# Integration of mechanistic and repeat dose toxicity data in the derivation of an oral reference dose for HFPO-DA

**DOI:** 10.1093/toxsci/kfag045

**Published:** 2026-04-10

**Authors:** Chad M Thompson, Melissa M Heintz, Sarah I Rogers, Melissa J Vincent, Laurie C Haws

**Affiliations:** ToxStrategies, LLC, Katy, TX, 77494, United States; ToxStrategies, LLC, Asheville, NC, 28801, United States; ToxStrategies, LLC, Asheville, NC, 28801, United States; ToxStrategies, LLC, Asheville, NC, 28801, United States; ToxStrategies, LLC, Austin, TX, 78731, United States

**Keywords:** HFPO-DA (GenX), PFAS, risk assessment

## Abstract

HFPO-DA (ammonium 2,3,3,3‐tetrafluoro‐2‐(heptafluoropropoxy)‐propanoate) is a short-chain perfluoroether carboxylic acid used as a polymerization aid in the manufacture of some types of fluorinated polymers. Existing toxicity criteria for HFPO-DA include chronic and subchronic oral reference dose (RfD) values based on liver effects in mice. New mechanistic data demonstrate that these liver effects are incontrovertibly linked to rodent-specific peroxisome proliferator-activated receptor alpha (PPARα) signaling pathways that lack human relevance. Therefore, it was critical to reevaluate existing HFPO-DA RfD values. A literature review was conducted to identify human and animal studies that might serve as the basis of updated toxicity criteria. Relevant studies were considered along with a newly completed chronic bioassay in mice. No epidemiological studies were determined acceptable for use in toxicity value development. The only neoplasms in the chronic mouse bioassay were in the liver and were considered PPARα related. The most sensitive extrahepatic noncancer endpoints in rodents involved placental lesions in reproductive and developmental toxicity studies and reduced testicular cellularity in mice following chronic exposure. Both effects resulted in RfD values of 0.001 mg/kg-d. Also presented are probabilistic risk assessment methods that resulted in a similar probabilistic RfD. These results indicate the need to update existing toxicity criteria for HFPO-DA.

HFPO-DA (ammonium 2,3,3,3-tetrafluoro-2-(heptafluoropropoxy)-propanoate; CASRN 62037-80-3) is used as a polymerization aid in the manufacture of some fluorinated polymers that are used in various applications including semiconductor fluid handling, aerospace and telecommunications cabling, renewable hydrogen production, and lithium-ion batteries (https://www.chemours.com/en/about-chemours/genx; https://www.teflon.com/en/industries-and-solutions/industries). HFPO-DA is one of several perfluorooctanoate acid (PFOA) replacements, with different fluoropolymer manufacturers having developed their own polymerization aid technologies to replace PFOA (Wang et al. 2013). In contrast to the historical uses of per- and polyfluorinated alkyl substances (PFAS) such as PFOA, HFPO-DA is not itself used in firefighting foam, carpets, textiles, paper, or other industrial consumer products. In addition, compared with long-chain PFAS, HFPO-DA is rapidly eliminated and does not bioaccumulate in tissues (Gannon et al. 2016; Abraham et al. 2024).

HFPO-DA has been evaluated in short- and long-term toxicity studies, as well as specialty assays such as immunotoxicity studies (Thompson et al. 2019; U.S. EPA 2021). Like other PFAS with widely differing toxicity criteria (Burgoon et al. 2023), the toxicity criteria proposed for HFPO-DA differ by orders of magnitude. For example, Thompson et al. (2019) proposed a chronic reference dose (RfD) of 0.01 mg/kg-d based on cystic focal degeneration in a 2-yr rat bioassay, whereas the EPA developed a chronic RfD of 0.000003 mg/kg-d based on liver effects in subchronic mouse studies (U.S. EPA 2021). Since the conduct of these assessments, considerable evidence has amassed indicating that most liver effects induced by HFPO-DA in mice are part of the upstream key events of the rodent-specific peroxisome proliferator-activated receptor alpha (PPARα) mode of action (MOA) for hepatocarcinogenesis that is widely regarded to lack human relevance (Heintz et al. 2023a). New information includes in vitro and in vivo studies utilizing wild-type and PPARα-null mice that have demonstrated the dependence of HFPO-DA liver effects on the PPARα receptor (Heintz et al. 2024a, 2024b, 2025; Ren et al. 2024).

The purpose of the current assessment is to re-evaluate existing toxicity data on HFPO-DA in light of this new mechanistic evidence, as well as to evaluate newly published data on HFPO-DA, including a newly completed chronic study in mice (Thompson et al. 2026), to determine if updated toxicity criteria are warranted. Systematic review methods were used to facilitate the identification of relevant studies, including the publication of a protocol describing the approach to systematically identify and select experimental animal and epidemiological studies assessing apical outcomes for use in toxicity value development (Heintz et al. 2023b). Identified studies were reviewed against predefined inclusion/exclusion criteria for consideration of noncancer and cancer apical outcomes. Mechanistic data were used to assess apical outcome biological plausibility and human relevance. Relevant endpoints were then subjected to quantitative dose–response modeling (if feasible) and arrayed to determine the most sensitive effects to be carried forward for toxicity criteria derivation.

Given the aforementioned disparity in published toxicity criteria for HFPO-DA, the newly derived toxicity criteria were compared with alternative methods of safety evaluation, such as the threshold for toxicological concern (TTC) and probabilistic-based RfD derivation. Overall, this work attempts to leverage best available science and the best available risk assessment practices to develop toxicity criteria for HFPO-DA that are scientifically sound and protective of human health.

## Materials and methods

Development of updated toxicity values associated with oral exposures to HFPO-DA for use in human health risk assessment was conducted using systematic review methodology consistent with international recommendations (NTP 2015; WHO 2021). A protocol describing the approach to systematically identify and select experimental animal and epidemiological studies assessing apical outcomes for use in toxicity value development was developed a priori based on previous toxicity assessments by Thompson et al. (2019) and U.S. EPA (2021). The protocol was posted publicly to Open Source Framework (OSF), an online data repository (Heintz et al. 2023b).

### Evidence identification and selection

Detailed information regarding evidence identification and selection is described in the protocol (Heintz et al. 2023b). Briefly, the search strategy for this updated assessment employed the same search syntax used by USEPA in its most recent toxicity assessment of HFPO-DA (USEPA 2021) (see Table S1; Heintz et al. 2023b). PubMed and Embase were queried using database-specific search syntax to identify evidence (i.e. candidate studies) published in the peer-reviewed literature through January 14, 2025. Unpublished studies were identified and obtained from USEPA’s Health and Environmental Research Online (HERO) database for “Gen X” (https://hero.epa.gov/hero/index.cfm/project/page/project_id/2627). Studies identified from these searches were reviewed against inclusion/exclusion criteria defined by the PECO (Population, Exposure, Comparator and Outcome) statement in Table S2 (Heintz et al. 2023b) as follows, P: In vivo experimental animal (mammalian) or epidemiological studies, E: Controlled or measured oral exposure to HFPO-DA, C: Untreated or vehicle-exposed negative control or a comparison or reference population exposed to lower levels of HFPO-DA, O: Noncancer and cancer apical outcomes. Studies using in vitro, ex vivo, or nonmammalian models, and those investigating mechanistic effects of HFPO-DA exposure were excluded as candidates for toxicity value development. However, these study types were categorized during evidence identification for downstream targeted assessments of apical outcome biological plausibility and human relevance.

### Data extraction and critical appraisal

Data were extracted from studies that met inclusion criteria at full text as described in Heintz et al. (2023b). Briefly, included animal studies were critically appraised for internal validity and risk of bias using ToxRTool (Toxicological data Reliability Assessment Tool) (NTP 2015), whereas epidemiological studies were assessed as described previously (Money et al. 2013). The output from both appraisal methods corresponds to a Klimisch categorization (1, 2, 3, or 4) of reliability. The potential impact of risk of bias associated with methodological attributes was considered within the context of the impact on reliability and reliance in risk assessment (see File S1). Studies that achieved reliability scores of 1 or 2 were carried forward for consideration in toxicity value derivation.

### Dose–response analysis

Dose‐response modeling was conducted with US EPA’s Benchmark Dose Software (BMDS) using the suite of frequentist dichotomous and continuous models. Initial benchmark dose (BMD) modeling was conducted with BMDS v2.7; however, all effects described herein were remodeled in the latest version BMDS Desktop 25.1. For dichotomous datasets, a benchmark response (BMR) of 10% extra risk was used effects in adults, whereas a BMR of 5% extra risk was used for effects in offspring. For continuous datasets, a BMR of 1 standard deviation (1SD) was used except for growth-related parameters where 10% and 5% relative deviation was used for adults and offspring, respectively. These BMR values are generally consistent with EPA BMD guidance (USEPA 2012) and many other EPA risk assessments. Models were used to obtain BMD values and their corresponding 95% lower confidence limit (BMDL) values. Model fits were judged using criteria such as *P*‐values, scaled residuals, Akaike information criterion, parsimony, visual inspection, and BMDS recommendations, as well as warnings regarding BMD/BMDL ratio and distance below the lowest study dose (USEPA 2012). Model output reports from BMDS (including plots) are shown in Supplementary Material. The model selection was largely consistent with the model recommended by BMDS and user rationale for different model selection is recorded in the reports. Only the results for the appropriate distribution and variance option (e.g. normal and constant variance) are contained in the reports. Model results are not provided for datasets that did not provide suitable fits; instead, for these datasets no-observed-adverse-effect-level (NOAEL) or lowest-observed-adverse-effect-level (LOAEL) values served as points of departure (PODs).

### Reference dose derivation

POD values were converted to human equivalent dose (HED) by allometric scaling using generic dosimetric adjustment factor (DAF) values of 4 and 7 for rats and mice, respectively (U.S. EPA 2002; USEPA 2011). Although there is some uncertainty in the use of allometric scaling for gestational exposure and early life exposure, HEDs were derived for these endpoints using the same DAFs (USEPA 2011). Notably, allometric scaling was used to adjust PODs in a recent HFPO-DA assessment by EPA (U.S. EPA 2021). Additional considerations for the interspecies adjustment of PODs for effects in female rats are described in the Results. Candidate reference dose (cRfD) values were derived for several endpoints by applying uncertainty factors to each HED value, including the interspecies UF (UF_A_) and intraspecies UF (UF_H_). Additional UFs considered included use of data from less‐than‐lifetime (i.e. subchronic) studies (UF_S_), use of a LOAEL instead of a NOAEL or BMDL (UF_L_), and overall completeness of database (UF_D_). The deterministic RfD calculation is as follows:


$$\text{RfD}\;\left(\text{mg}/\text{kg}-\text{day}\right)=\text{HED}÷[{\text{UF}}_{A}\times {\;\text{UF}}_{H}\times {\;\text{UF}}_{S}\times {\;\text{UF}}_{L}\times {\text{UF}}_{D}]$$

In addition to the deterministic RfD, a probabilistic RfD (pRfD) was also developed using the approximate probabilistic analysis (APROBA) spreadsheet (Chiu and Slob 2015; Chiu et al. 2018; WHO 2018). Using this approach, the pRfD is defined as the HD_M_^I^, where HD is the human dose (mg/kg-d), M is the target effect size (e.g. BMR of 10% extra risk), and I is the population incidence goal (e.g. 1%). In the APROBA spreadsheet, the upper and lower confidence limits on the BMD in animals (modeled in BMDS) were used to characterize the variability in the BMD, which is subsequently adjusted by an allometric scaling factor as well as interspecies and intraspecies uncertainty and variability factors based on historical data (Chiu et al. 2018). Using the M and I examples above, this would result in a random variable representing the human dose at which the most sensitive 1% of the population has a 10% extra risk of developing the adverse effect of interest (HD_10_^1%^). The fifth percentile of the distribution of the HD_10_^1%^ is designated the pRfD.

## Results

### Literature identification and selection

**Fig. 1.**
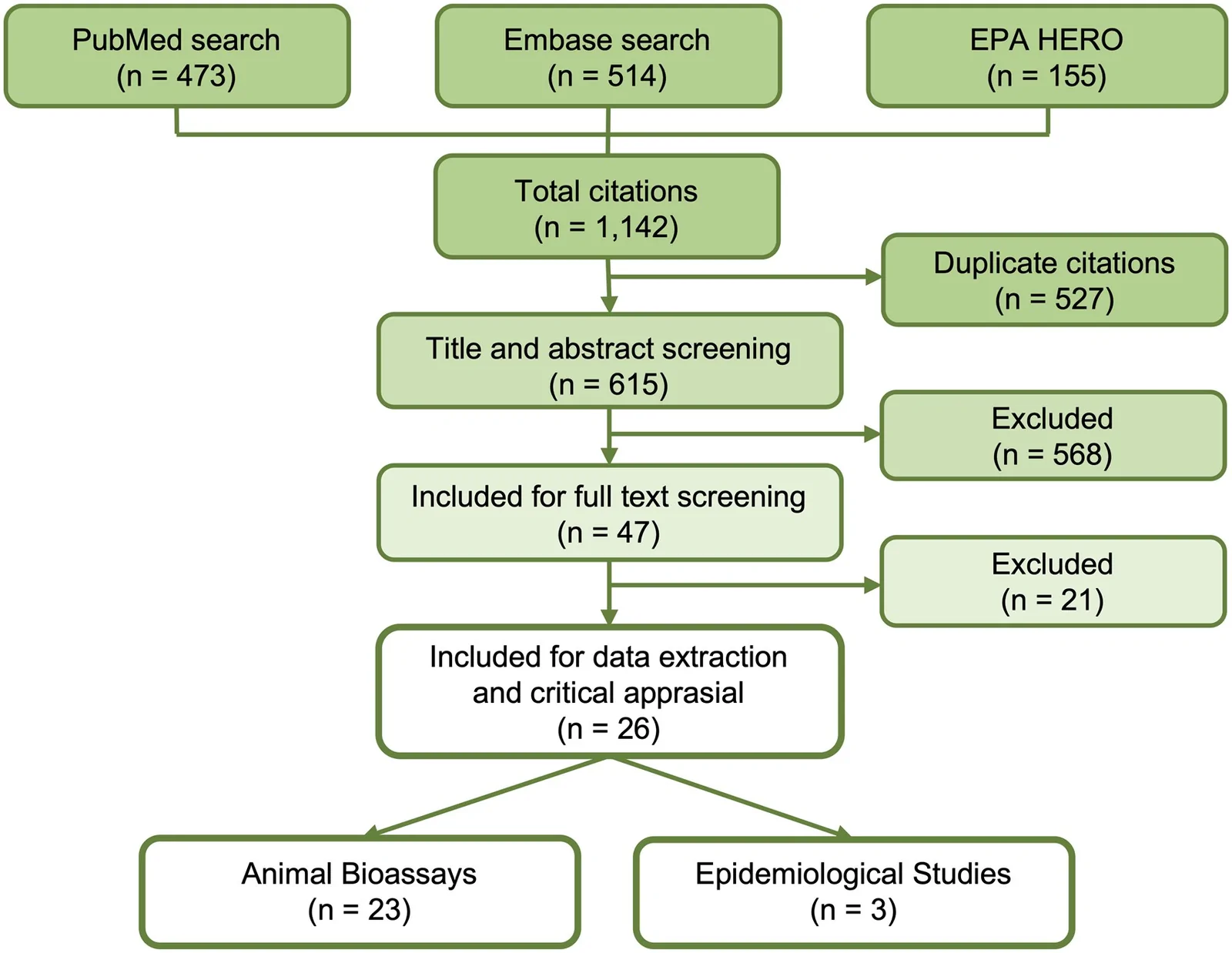
Flow diagram of literature search and screening results for HFPO-DA toxicity studies suitable for toxicity value development.

Following systematic review of the literature, 26 studies met the inclusion criteria at full text, consisting of 23 experimental animal studies and 3 epidemiological studies (Fig. 1). Studies excluded at each level of screening are listed in File S1.

### Data extraction and critical appraisal

#### Epidemiological studies

Each of the 3 epidemiological studies included for data extraction and critical appraisal assessed health effects associated with PFAS, including HFPO-DA, measured in participant serum or plasma. Health effects evaluated in these studies included serum lipid levels (e.g. cholesterol, triglycerides) (Rosen et al. 2022), unexplained recurrent spontaneous abortion (Nian et al. 2022), and polycystic ovarian syndrome (Zhan et al. 2023). The epidemiological study evaluating lipid outcomes did not detect HFPO-DA in the serum of any participant (Rosen et al. 2022).

**Fig. 2.**
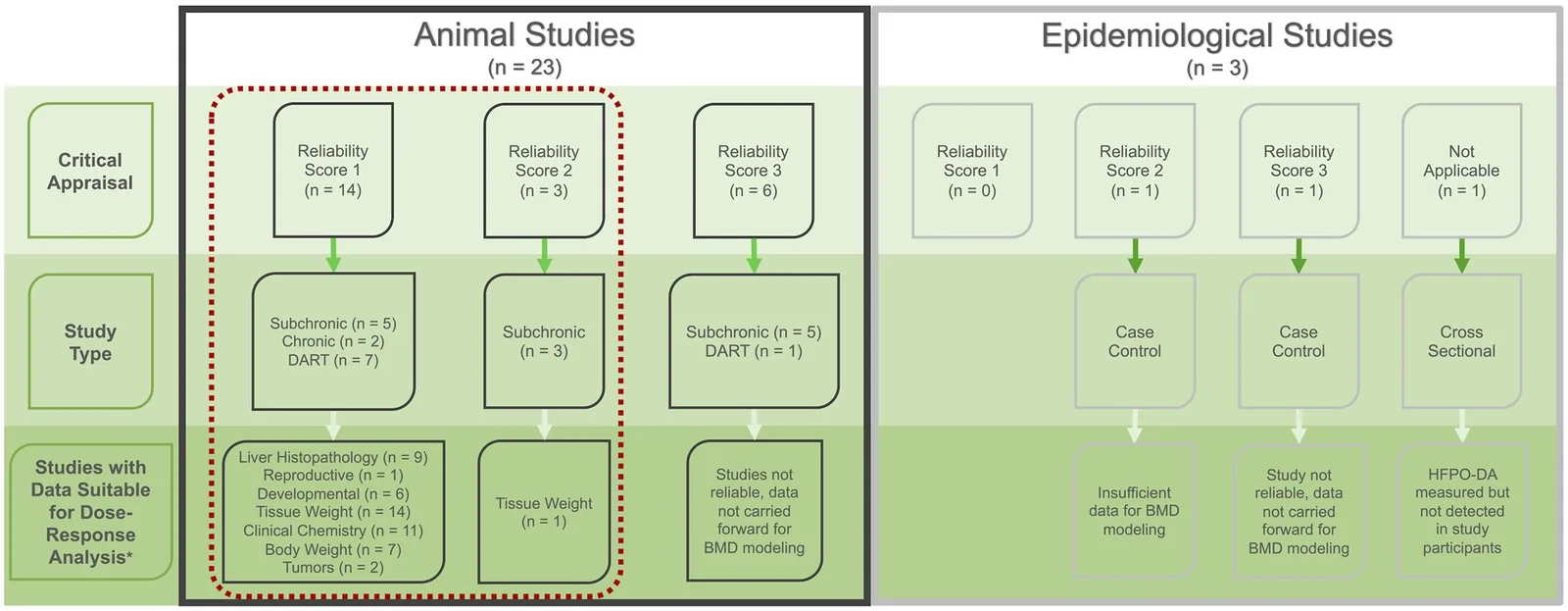
Evaluation of the evidence base for HFPO-DA toxicity value development. Each row indicates the number of animal or human studies according to reliability score, study type, and endpoints suitable for benchmark dose (BMD) modeling, respectively. A total of 17 animal studies (highlighted by the red dotted box) and 0 human studies met both inclusion and reliability criteria (i.e. reliability score of 1 or 2) and were carried forward for consideration in toxicity value development. *n* = number of studies; asterisk (*) indicates data from a single study may have been modeled for multiple endpoints.

Assessment of study reliability found that all three epidemiological studies had critical limitations that precluded their utility for assessing causal relationships and use in dose–response assessment (Fig. 2; see File S1 for details). Specifically, in each study, serum or plasma PFAS measurements were taken as a single measurement during study enrollment and therefore may not be reflective of PFAS concentrations at the time of disease onset. In addition, risks reported by study authors did not adequately account for co-exposures to other correlated PFAS compounds.

#### Animal studies

Out of the 23 animal studies included for data extraction and critical appraisal, 7 were unpublished laboratory reports and 16 were published studies from the peer-reviewed literature. Common outcomes evaluated in these studies were related to liver or developmental toxicity (Fig. 2**;** see File S1 for details). Assessment of study reliability found that 14 studies were reliable without restrictions (i.e. reliability score = 1), 3 studies were reliable with restrictions (i.e. reliability score = 2), and 6 studies were determined to be not reliable (i.e. reliability score = 3). Based on results from critical appraisal, a total of 17 animal studies met reliability criteria (i.e. reliability score of 1 or 2) and were carried forward for consideration in toxicity value development.

### Toxicity and dose–response analysis

#### Mode of action considerations

Existing US toxicity values for HFPO-DA are based on liver effects in rodents. Thompson et al. (2019) posited that some of the liver effects observed in a chronic bioassay in rats were likely the result of a PPARα MOA but available data at the time were insufficient for a definitive conclusion. EPA (2021) posited that the liver effects in rodents might be the result of the PPARα MOA for rodent hepatocarcinogenesis, stating, “the available data indicate that a PPARα MOA is plausible in the liver in response to GenX chemical exposure…however, there are not yet enough data to conclude that PPARα activation is the sole mechanism underlying the liver effects associated with exposure to GenX chemicals. For example, there are no studies investigating GenX chemical exposure in PPARα-null mice.” EPA (2021) also posited that other MOAs involving cytotoxicity, PPARγ activation, or mitochondrial toxicity might be operational. The deficiency in PPARα-null studies has been addressed in a series of in vitro and in vivo studies that clearly demonstrate the absence or mitigation of HFPO-DA phenotypic and transcriptomic effects in PPARα-null mice compared with wild-type mice (Heintz et al. 2024b, 2025). Moreover, studies comparing the phenotypic and transcriptomic responses of HFPO-DA to prototypical PPARα and PPARγ agonists as well as cytotoxic agents have demonstrated high concordance with the PPARα agonist but not the other prototypical chemicals (Heintz et al. 2024a, 2024b, 2025).

These findings corroborate earlier in vivo transcriptomic studies showing PPARα-related gene expression changes and enrichment of peroxisomal proliferator signaling pathways among the most sensitive responses to HFPO-DA in the mouse liver (Chappell et al. 2020; Heintz et al. 2022). These and other data indicate that HFPO-DA-induced liver tumors in mice are the result of a PPARα/peroxisomal proliferation MOA (Heintz et al. 2023a). This MOA is widely regarded to have limited human relevance (Corton et al. 2014, 2018; Felter et al. 2018), and provides important context for many of the effects discussed in subsequent sections.

#### Toxicokinetic considerations

Toxicokinetic analyses indicate that the alpha phase elimination (from central to peripheral compartments) half-lives ranged from 1.9 to 5.8 h in cynomolgus monkeys, mice, and male rats, whereas the half-life was ∼10-fold faster in female rats than male rats (0.2 vs 2.8 h). The slower beta elimination phase was comparable between sexes and species (Gannon et al. 2016). Consistent with these data, HFPO-DA studies have generally included higher doses for female rats than male rats and adverse effects are typically observed at higher doses in female rats than male rats or mice. The half-life of HFPO-DA in nonhuman primates is similar in both sexes and comparable to that of mice and male rats (Gannon et al. 2016). HFPO-DA has not been widely detected in human samples (Pritchett et al. 2019; Kotlarz et al. 2020; Petriello et al. 2022) (Note: there are multiple errata to Kotlarz et al. (2020)), and we are unaware of data sufficient to compare human HFPO-DA pharmacokinetics with those in rodents or monkeys, although data from a single male human volunteer report a half-life of 2.9 d (Abraham et al. 2024) that is comparable to the ∼72 h half-life in male and female monkeys (Gannon et al. 2016). According to EPA (2021), an analysis of 25 workers exposed to HFPO-DA exhibited a half-life of ∼81 h; however, EPA concluded the data were insufficient for derivation of interspecies extrapolation factors. Given the unique pharmacokinetics in female rats and the availability of chronic data in mice and male rats, we focused on effects in female rats observed in specialty studies such as reproductive and developmental toxicity endpoints (see “Reproductive and developmental toxicity” section). The section “Deterministic RfD derivation” includes additional discussion of interspecies extrapolation of effects in female rats.

#### Cancer endpoints

Genotoxicity data for HFPO-DA do not indicate genotoxic risk (RIVM 2016; Thompson et al. 2019; U.S. EPA 2021). One study claiming genotoxicity in a rat thyroid cell line has several deficiencies, including no apparent use of positive controls in the comet and micronucleus assays (Coperchini et al. 2020). More recently, transcriptomic analyses of several PFAS indicated a lack of evidence for DNA damage in human liver spheroids (Rowan-Carroll et al. 2025). Among several transcriptomic analyses in hepatocytes (Heintz et al. 2024a, 2024b), mice (Chappell et al. 2020; Heintz et al. 2022, 2025), and rats (Heintz et al., in review), there is no enrichment of DNA damage pathways. There is overwhelming evidence that HFPO-DA causes liver effects via the PPARα MOA for rodent liver tumors (Heintz et al. 2023a, 2024a, 2024b, 2025), and thus genotoxicity is unlikely to play a role in the limited carcinogenicity of HFPO-DA. Chronic exposure to HFPO-DA significantly increased adenomas and carcinomas of the liver in female rats and pancreatic acinar cell adenomas/carcinomas in male rats; a nonsignificant increase in Leydig cell tumors was also observed in male rats (Caverly Rae et al. 2015). Chronic exposure of mice to HFPO-DA significantly increased liver adenomas and carcinomas in male and female mice, with no evidence of tumorigenicity in any other organs (Chemours 2024; Thompson et al. 2026) (Table 1). These tumor locations are associated with peroxisome proliferators and referred to as the tumor triad consisting of liver adenomas/carcinomas (mice and rats), testicular Leydig cell tumors (rats), and pancreatic acinar cell tumors (rats) (Klaunig et al. 2003; Corton et al. 2018; Felter et al. 2018). It is widely accepted that liver tumors induced by nongenotoxic PPARα activators are not relevant to humans (Klaunig et al. 2003; Corton et al. 2018). The MOAs for pancreatic and Leydig cell tumors are less understood but might also relate to PPARα mechanisms (Klaunig et al. 2003).

**Table 1.** Tumors in rats and mice exposed to HFPO-DA.

	9 mo					18 mo					24 mo			
*Male mice*														
Dose (mg/kg-d):	0	0.05	0.1	0.5	5	0	0.05	0.1	0.5	5	–	–	–	–
No. in group:	20	20	19	20	20	50	50	50	50	50	–	–	–	–
Hepatocellular adenoma	0	0	0	0	9*	3	2	4	8	35*	–	–	–	–
Hepatocellular carcinoma	0	0	0	0	0	0	0	0	0	18*	–	–	–	–
Combined adenoma/carcinoma	0	0	0	0	9*	3	2	4	8	46*	–	–	–	–
*Female mice*														
Dose (mg/kg-d):	0	0.05	0.1	0.5	5	0	0.05	0.1	0.5	5	–	–	–	–
No. in group:	20	20	20	20	20	50	50	50	50	50	–	–	–	–
Hepatocellular adenoma	0	0	0	0	0	0	0	0	0	8*	–	–	–	–
Hepatocellular carcinoma	0	0	0	0	0	0	0	0	0	2	–	–	–	–
Combined adenoma/carcinoma	0	0	0	0	0	0	0	0	0	9*	–	–	–	–
*Male rats*														
Dose (mg/kg-d):	–	–	–	–	–	–	–	–	–	–	0	0.1	1	50
No. in group:	–	–	–	–	–	–	–	–	–	–	70	70	70	70
Pancreas acinar cell adenoma	–	–	–	–	–	–	–	–	–	–	0	1	0	3
Pancreas acinar cell carcinoma	–	–	–	–	–	–	–	–	–	–	0	0	0	2
Combined adenoma/carcinoma	–	–	–	–	–	–	–	–	–	–	0	1	0	5*
*Female rats*														
Dose (mg/kg-d):	–	–	–	–	–	–	–	–	–	–	0	1	50	500
No. in group:	–	–	–	–	–	–	–	–	–	–	70	70	70	70
Hepatocellular adenoma	–	–	–	–	–	–	–	–	–	–	0	0	0	11*
Hepatocellular carcinoma	–	–	–	–	–	–	–	–	–	–	0	0	0	4*

–, indicates exposure duration was not done; *, indicates significantly different from control.

As indicated in the section “Toxicokinetic considerations,” female rats exhibit rapid clearance of HFPO-DA and thus liver tumors were only elevated in female rats exposed to a near limit dose (1000 mg/kg-d is a limit dose for an OECD TG 452 chronic bioassay; OECD 2018) of 500 mg/kg-d. In contrast, liver tumors were significantly increased in mice exposed to 5 mg/kg-d (Table 1). The liver tumors in mice were anticipated based on clear evidence of a PPARα/peroxisomal proliferator MOA for HFPO-DA-mediated liver effects in rodents (see “Mode of action considerations” section). As such, dose–response analysis was not conducted on liver tumors in mice or rats due to lack of relevance for human health risk assessment. Nor was dose–response analysis conducted on the weak pancreatic acinar cell tumor response in male rats at the highest dose (50 mg/kg-d). This is consistent with EPA (2021) who characterized the evidence for carcinogenicity as only *suggestive*, which EPA guidance considers insufficient for dose–response analysis (U.S. EPA 2005). Given the lack of human relevance for liver tumors in mice and female rats and the weak tumor response in the rat pancreas, no cancer slope factor (CSF) value(s) were derived for HFPO-DA.

#### Noncancer endpoints

Noncancer effects evaluated included organ toxicity (i.e. histopathology), immunotoxicity, reproductive, and developmental toxicity. Many of the datasets were subjected to BMD modeling; when modeling was not feasible the endpoints were based on NOAEL or LOAEL values. With only a few exceptions, statistical significance was based on the analyses presented in the original studies. The sections below focus on the most sensitive effects of potential concern for human health risk assessment.

##### Organ toxicity

###### Liver

**Fig. 3.**
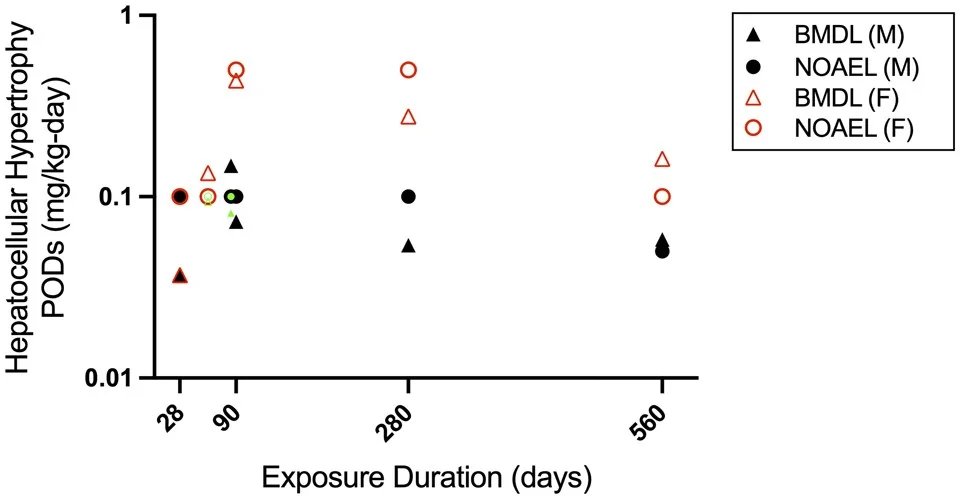
Evaluation of sensitive liver changes in mice across studies of multiple duration (28–560 d; 4–80 wk). Solid and open triangles represent BMDL_10_ values for males and female, respectively. Solid and open circles represent NOAEL values for males and female, respectively. In many cases, the BMDL_10_ values are lower than the NOAEL due to the abrupt change in incidence from 0 to 100%, which is not ideal for BMD modeling. Black and red symbols are hepatocellular hypertrophy as diagnosed in study reports, whereas the smaller green symbols represent NOAEL and BMDL_10_ values for EPA’s “constellation of lesions” that includes hepatocellular hypertrophy. Correlation coefficient (*r*) = 0.026. M = male; F = female.

The liver has consistently been identified as the most sensitive target organ in both rats and mice. EPA’s draft (2018) and EPA’s final (2021) toxicological reviews of HFPO-DA based their RfD values on liver changes observed in subchronic mouse studies (U.S. EPA 2018, 2021). In EPA (2021), EPA combined the incidence of several related liver lesions in parental mice in a reproductive/developmental study (DuPont 2010c) into a so-called “constellation of lesions” comprised of (i) cytoplasmic alteration (consistent with hepatocellular hypertrophy), (ii) apoptosis, (iii) single cell necrosis, and (iv) focal necrosis. Notably, the constellation was not a specific diagnosis in the original DuPont studies or in the Pathology Working Group report which served as a cornerstone of the EPA (2021) evaluation, but rather the incidence data for the constellation was aggregated subsequently by EPA. Although the term constellation has been used to encompass several diagnostic terms to describe a more generalized condition (Thoolen et al. 2010), it is unclear whether aggregating multiple lesions into a single incidence dataset is appropriate for dose–response modeling. More importantly, in this case, all of the lesions in EPA’s constellation are related to the upstream key events in the PPARα MOA for rodent liver tumors as evidenced by their absence in PPARα null mice (Heintz et al. 2025). These key events do not occur in humans and therefore, the lesions comprising the constellation are not relevant to humans. Because hepatocellular hypertrophy (or cytoplasmic alteration) was the most sensitive histopathological effect in the liver and one of the effects in the constellation, the PODs (i.e. BMDLs, NOAELs) for hepatocellular hypertrophy are very similar to the PODs for the constellation. As shown in Fig. 3, the BMDL_10_ and NOAEL values for hepatocellular hypertrophy in 4-wk, 13-wk, 40-wk, and 80-wk studies as well as parental mice in a reproductive study all have values of ∼0.1 mg/kg-d regardless of study duration. As is evident in Fig. 3, there is no significant correlation between exposure duration and the PODs (*r* = 0.026).

A recently completed chronic bioassay in mice concluded that “[s]ince hepatocellular hypertrophy resulted in parenchymal damage (i.e. single cell necrosis and/or hepatocellular necrosis, pigmented macrophages, and mononuclear/mixed cell infiltrate), and was associated with elevation of liver enzymes (ALT, AST and/or ALP), the hypertrophy was considered adverse in the 0.5 and 5 mg/kg/d males and females…”; further, “[t]he nature of the hypertrophy…was consistent with peroxisome proliferation…” (Chemours 2024). The term “adverse” here does not imply relevance for human health risk assessment, but rather refers to potential harm to the test animal in the confines of the study design (Pandiri et al. 2017; Gopinath and Mowat 2019). Given the compelling evidence for the rodent-specific PPARα MOA (see “Mode of action considerations” section), we did not consider these changes in the mouse liver relevant for human health risk assessment. This issue has been discussed in detail elsewhere (Heintz et al. 2023a). Nevertheless, we include a cRfD based on liver hepatocellular hypertrophy (the most sensitive histopathological effect in rodent livers) in our comparison to other cRfD values in the section “Comparison of new deterministic RfD to existing toxicity criteria”.

It has been argued that reports of necrosis are (or could be) evidence for a PPARα-independent MOA that might have relevance to humans. EPA (2021) acknowledged that data gaps in the PPARα MOA, specifically data in PPARα null models (see “Mode of action considerations” section), precluded conclusions about a PPARα MOA and hypothesized that reports of necrosis might be evidence of a non-PPARα MOA. Some of this concern for a non-PPARα cytotoxic MOA is, in part, a result of the term “single cell necrosis” that has been synonymous with apoptosis (Thoolen et al. 2010). In fact, some of the original DuPont toxicity studies on HFPO-DA scored lesions as single cell necrosis but described the lesions in the text as having characteristics consistent with apoptosis (some of this nomenclature confusion might be a result of electronic pathology data recording systems not having up to date or nuanced entries for certain lesions). There has also been debate about whether some of the liver lesions represent necrotic or apoptotic cell death (Chappell et al. 2020; U.S. EPA 2021; Heintz et al. 2022; Thompson et al. 2023), and studies have demonstrated that the some putative necrotic lesions following HFPO-DA exposure stain positive for markers of apoptosis (Thompson et al. 2023). It has also been argued that regardless of whether the cell death is apoptotic or necrotic, an increase in cell death is not part of the PPARα MOA because the seminal PPARα MOA reviews include suppression of apoptosis as a key event (Klaunig et al. 2003; Corton et al. 2014, 2018). However, this is largely based on acute in vitro and in vivo evidence, whereas longer-term studies with potent PPARα activators report increased apoptosis that is likely present to counterbalance increased hepatomegaly (Corton et al. 2014):

“*Suppression of apoptosis by PPARα activators occurs under acute exposure conditions when the liver is adapting to chemical exposure and increasing in size. However, once a steady state of liver enlargement is reached, levels of apoptosis likely return to background levels or to levels that balance the low level of cell proliferation that occurs for potent PPARα activators. Consistent with this, two reports show that chronic exposure of rats and mice to the PPARα activator WY under conditions that result in chronic low level hepatocyte proliferation leads to increases in apoptosis…The data indicate that in the intact animal a physiological function of PPARα activation is to balance the levels of hepatocyte proliferation and apoptosis in an effort by the system to adapt to chemical exposure, i.e. an initial increase in the size and number of hepatocytes followed by maintenance of the system in the new steady state in which low levels of hepatocyte proliferation are balanced by low levels of apoptosis.”*

As such, the argument that increased cell death in HFPO-DA studies is inconsistent with a PPARα MOA and instead evidence of a cytotoxic MOA that deserves consideration for human health risk assessment is tenuous.

Minimal to mild biliary hyperplasia was observed in male mice in the 5 mg/kg-d group following 18 mo of exposure (Table 2). This was not observed in males at 9 mo or in females at any timepoint. These lesions might to be associated with hepatocellular hypertrophy; however, the biliary hyperplasia also corresponded to increases in serum ALP, AST, and ALT as well as serum total bilirubin (Thompson et al. 2026). Together, these changes can be associated with cholestasis (Ye et al. 2022); however, staining for bile in the liver with Hall’s stain was negative (Thompson et al. 2026). Several studies have demonstrated mild biliary hyperplasia and cholestasis in rodents exposed to PPARα activators (Gyamfi and Wan 2009; Hailey et al. 2014). For example, exposure to WY-14,643 in wild-type mice increased serum bile acids and liver enzymes, but exposure to mice null for the PPARα heterodimeric partner retinoid X receptor had minimal responses (Gyamfi and Wan 2009). It is generally established that PPARα regulates bile acid synthesis and transport by downregulating expression of transporters responsible for bile acid absorption from blood, upregulating expression of transporters responsible for bile efflux from the liver to the blood and bile ducts, and also downregulating expression of rate limiting enzymes in bile acid synthesis within the liver (Xie et al. 2019; Ye et al. 2022). These PPARα-mediated perturbations tend to reduce bile acid levels within the liver which is the antithesis of cholestasis where increased bile acids within the liver induce toxicity. Indeed, PPARα-activating fibrates have been investigated for alleviating cholestasis in humans (Ye et al. 2022). Notably, exposure to HFPO-DA in mice altered the expression of several enzymes and transporters involved in bile acid homeostasis (Thompson et al. 2026). Clearly, there is a potential nexus between biliary hyperplasia, cholestasis, serum liver enzymes (particularly ALP), and PPARα-mediated regulation of many aspects of bile acid homeostasis. The biliary hyperplasia could be a downstream result of PPARα-mediated hepatomegaly and toxicity or potentially PPARα-mediated alterations in bile acid homeostasis. Given this uncertainty in the etiology, biliary hyperplasia was carried forward for cRfD derivation.

**Table 2.** Non-neoplastic liver lesions in male and female mice following chronic exposure.

	9 mo					18 mo				
Dose (mg/kg-d):	0	0.05	0.1	0.5	5	0	0.05	0.1	0.5	5
*Males*										
**Liver (no. examined)**	20	20	18	20	19	44	43	43	42	34
hypertrophy, hepatocellular	0	1	1	15^a^	19^a^	2	2	9^a^	38^a^	33^a^
Minimal	*0*	*0*	*1*	*0*	*0*	*0*	*2*	*5*	*5*	*0*
Mild	*0*	*1*	*0*	*15*	*0*	*2*	*0*	*4*	*24*	*3*
Moderate	*0*	*0*	*0*	*0*	*19*	*0*	*0*	*0*	*9*	*25*
Marked	*0*	*0*	*0*	*0*	*0*	*0*	*0*	*0*	*0*	*5*
Pigmented macrophage	0	0	0	3	19^a^	0	0	2	13^a^	33^a^
Minimal	0	0	0	*3*	*0*	*0*	*0*	*2*	*7*	*3*
Mild	0	0	0	*0*	*2*	*0*	*0*	*0*	*2*	*9*
Moderate	0	0	0	*0*	*17*	*0*	*0*	*0*	*4*	*21*
Focus of cellular alteration, basophilic	0	0	0	1	5^a^	0	1	1	1	9^a^
Mild	*0*	*0*	*0*	*1*	*2*	*0*	*1*	*1*	*0*	*5*
Moderate	*0*	*0*	*0*	*0*	*3*	*0*	*0*	*0*	*1*	*4*
Single cell necrosis, hepatocellular^a^	0	0	0	0	6^a^	–	–	–	–	–
Minimal	*0*	*0*	*0*	*0*	*6*	–	–	–	–	–
Hyperplasia, biliary	–	–	–	–	–	0	0	0	0	7^a^
Minimal	–	–	–	–	–	*0*	*0*	*0*	*0*	*5*
Mild	–	–	–	–	–	*0*	*0*	*0*	*0*	*2*
Infiltration, mononuclear cell	0	0	0	1	4^a^	6	1	5	11	12^a^
Minimal	*0*	*0*	*0*	*1*	*4*	*5*	*1*	*3*	*6*	*8*
Mild	0	0	0	0	0	*1*	*0*	*2*	*4*	*3*
Moderate	0	0	0	0	0	*0*	*0*	*0*	*1*	*1*
Infiltration, mixed cell	–	–	–	–	–	5	4	10	15^a^	22^a^
Minimal	–	–	–	–	–	*2*	*2*	*8*	*9*	*14*
Mild	–	–	–	–	–	*3*	*2*	*2*	*6*	*7*
Moderate	–	–	–	–	–	*0*	*0*	*0*	*0*	*1*
Vacuolation, hepatocellular	0	1	0	0	5^a^	–	–	–	–	–
Minimal	*0*	*1*	*0*	*0*	*5*	–	–	–	–	–
*Females*										
**Liver (No. examined)**	20	18	20	20	20	43	40	41	43	37
hypertrophy, hepatocellular	0	0	0	2	18^a^	0	1	0	14^a^	37^a^
Minimal	0	0	0	0	0	*0*	*0*	*0*	*9*	*2*
Mild	*0*	*0*	*0*	*2*	*10*	*0*	*1*	*0*	*5*	*6*
Moderate	*0*	*0*	*0*	*0*	*8*	*0*	*0*	*0*	*0*	*29*
Pigmented macrophage	1	1	1	3	19^a^	5	1	4	13^a^	36^a^
Minimal	*1*	*1*	*1*	*2*	*12*	*2*	*0*	*2*	*9*	*6*
Mild	*0*	*0*	*0*	*1*	*7*	*2*	*1*	*2*	*3*	*17*
Moderate	0	0	0	0	0	*1*	*0*	*0*	*1*	*12*
Marked	0	0	0	0	0	0	0	0	0	1
Necrosis, hepatocellular^a^	1	0	1	3	7^a^	–	–	–	–	–
Minimal	*1*	*0*	*1*	*3*	*7*	–	–	–	–	–
Infiltration, mononuclear cell	–	–	–	–	–	7	2	7	6	23^a^
Minimal	–	–	–	–	–	*2*	*2*	*6*	*4*	*5*
Mild	–	–	–	–	–	*5*	*0*	*1*	*2*	*17*
Moderate	–	–	–	–	–	*0*	*0*	*0*	*0*	*1*
Infiltration, mixed cell	–	–	–	–	–	15	7	8	24^a^	24^a^
Minimal	–	–	–	–	–	*11*	*7*	*8*	*17*	*16*
Mild	–	–	–	–	–	*4*	*0*	*0*	*7*	*6*
Moderate	–	–	–	–	–	*0*	*0*	*0*	*0*	*2*
Cellularity, increased; Ito cell	–	–	–	–	–	0	0	0	2	5^a^
Mild	–	–	–	–	–	*0*	*0*	*0*	*1*	*4*
Moderate	–	–	–	–	–	*0*	*0*	*0*	*0*	*1*
Marked	–	–	–	–	–	*0*	*0*	*0*	*1*	*0*

– indicates no significant differences for this lesion at indicated timepoint.

a Significantly different from control (*P* < 0.05).

As already indicated, the POD for most lesions in adult, nongravid female rats were higher than male rats and therefore were not considered for cRfD derivation. Following chronic exposure of male rats to HFPO-DA, liver histopathological changes included centrilobular hypertrophy, centrilobular necrosis, and focal cystic degeneration, which had BMDL_10_ values ranging from 6.5 to 38.2 mg/kg-d. Although there were no significant differences in absolute or relative liver weight at study termination (24 mo), a slight (16%) but statistically significant increase in relative liver weight was observed in the high dose group at the interim sacrifice (12 mo). Notably, excessive hypertrophy can lead to hepatocellular degeneration and necrosis and zonal hepatocellular necrosis such as the centrilobular necrosis observed in rats exposed to 50 mg/kg-d HFPO-DA and can be the result of either direct xenobiotic toxicity or indirect damage from enzyme induction (Thoolen et al. 2010). Although many of the effects observed in the chronic rat bioassay are consistent with PPARα activation, the etiology of cystic liver degeneration (also referred to as spongiosis hepatis), common among aging rats, is unknown (Thoolen et al. 2010). This lesion was clearly present in all groups but increased significantly at the highest dose: 24/70, 24/70, 19/70, and 42/70 at 0, 0.1, 1, and 50 mg/kg-d, respectively. Given the unknown MOA, this lesion was carried forward for cRfD derivation.

###### Adrenal gland

In male mice, extrahepatic non-neoplastic lesions included adrenal gland cortical hypertrophy at 5 mg/kg-d at 9 and 18 mo (Table 3). These histopathological changes correlated with higher adrenal gland weights at 5 mg/kg-d (data not shown). As described in Thompson et al. (2026), these changes could have been due to stress; however, PPARα activators have been shown to increase relative adrenal weight, adrenal and serum corticosterone levels, and adrenal cortex hyperplasia in wild-type mice but not PPARα null mice (Wang et al. 2010). These changes were posited to be the result of PPARα agonist induction of fibroblast growth factor 21 (*fgf21*) in the liver and subsequent activation of the hypothalamic–pituitary–adrenal (HPA) axis resulting in stimulation of adrenal cortex hyperplasia and hypercortisolism (Wang et al. 2010; Liang et al. 2014). Expression levels of *fgf21* are elevated following in vitro and in vivo exposure to HFPO-DA (Heintz et al. 2024a, 2024b, 2025), including in mice exposed to HFPO-DA for 9 or 18 mo (Thompson et al. 2026). Although the adrenal changes might be the result of PPARα activation in the liver, the effects were nevertheless considered for cRfD derivation.

**Table 3.** Non-neoplastic extrahepatic lesions in male mice at 9 and 18 mo.

	9 mo					18 mo				
Dose (mg/kg-d):	0	0.05	0.1	0.5	5	0	0.05	0.1	0.5	5
**Adrenal gland (No. examined)**	20	20	18	20	20	43	41	43	42	34
hypertrophy, cortical	1	1	1	0	17^a^	10	10	6	3	27^a^
Minimal	*1*	*1*	*1*	*0*	*12*	*4*	*5*	*3*	*0*	*5*
Mild	*0*	*0*	*0*	*0*	*4*	*5*	*5*	*2*	*3*	*16*
Moderate	*0*	*0*	*0*	*0*	*1*	*1*	*0*	*1*	*0*	*6*
**Testis (No. examined)**	–	–	–	–	–	44	43	43	42	34
cellularity, decreased; germ cell	–	–	–	–	–	0	0	0	9^a^	22^a^
Minimal	–	–	–	–	–	*0*	*0*	*0*	*1*	*2*
Mild	–	–	–	–	–	*0*	*0*	*0*	*8*	*18*
Moderate	–	–	–	–	–	*0*	*0*	*0*	*0*	*2*

a Significantly different from control (*P* < 0.05).

###### Testes

Decreased germ cell cellularity in the mouse testes was observed at 18 mo (Chemours 2024; Thompson et al. 2026) (Table 3). Concomitant with these changes were decreased cellularity of spermatids in the epididymis, increased atrophy of the prostate, and depletion of secretion in the seminal vesicles—all considered secondary to the decreased cellularity of the testes (data not shown). The decreased germ cell cellularity was considered adverse to the test animal (Chemours 2024; Thompson et al. 2026).

Studies with other PFAS (e.g. PFOA) have reported reductions in testes and epididymis weight, reduced numbers of germ cells, and lower plasma testosterone in wild-type but not PPARα null mice (Li et al. 2011). Some effects were also observed in humanized PPARα (hPPARα) mice that express human PPARα in the liver of the PPARα-null mouse strain that lacks extrahepatic expression of PPARα (*ibid*). Changes in the testes and plasma testosterone in wild-type mice and hPPARα mice therefore indicate that the effects are potentially secondary to changes in the liver. Whether the changes in the mouse testes of wild-type and hPPARα mice are relevant to humans is uncertain, as there are species differences in testes biology (Steinbach et al. 2015). Given the lack of mechanistic data for HFPO-DA in the mouse testes to inform human relevance, this effect was carried forward for cRfD derivation.

###### Immunotoxicity

Concerns related to potential immunosuppression by PFAS have served as the basis for some PFAS toxicity criteria (USEPA 2024b). EPA (2021) concluded that HFPO-DA can induce immunosuppression in female mice based on results in a T cell-dependent antibody response (TDAR) assay (Rushing et al. 2017) and “supportive findings” for decreased spleen weight and globulin levels. Rushing et al. (2017) reported statistically significant reductions in serum IgM antibody production in female mice exposed to 100 mg/kg-d HFPO-DA for 28 d. These results were presented graphically as mean log2 titer values thereby making data extraction and dose–response modeling uncertain. Statistically significant decreases in relative spleen weight were also reported in female mice at 100 mg/kg-d (Rushing et al. 2017); however, our own ANOVA (ANOVA with Dunnett’s multiple comparison test; Prism v10.2.0) indicated that there was no difference between the control (5.34 ± 0.39) and 100 mg/kg-d (4.65 ± 0.56) groups (*P* = 0.1329). Statistically significant increases in CD8^+^ cell counts as well as CD4^−^/CD8^−^ and CD4^+^/CD8^+^ cell count ratios were observed in male mice at 100 mg/kg-d without changes in spleen weight or TDAR. Due to the minimal effects at 100 mg/kg-d and the absence of effects at 10 mg/kg-d (a dose twice as high as those in chronic mouse bioassays) changes in Rushing et al. (2017) were not considered further for cRfD derivation.

EPA (2021) also cites evidence for reduced relative spleen weight in female mice exposed to ≥0.5 mg/kg-d in a 90-d subchronic study as further evidence for immunotoxicity (DuPont 2010a); however, the study states, “These changes did not occur in a dose-related manner, were not associated with changes in mean absolute spleen weight, and were not associated with test substance-related microscopic changes in the spleen…Therefore, the changes in mean spleen weight relative to brain and body weight in females were considered spurious and unrelated to treatment.” A 28-d study in mice exposed up to 30 mg/kg-d HFPO-DA made similar conclusions, where a significant *increase* in absolute and relative spleen weight was observed at 0.1 mg/kg-d but not 3 or 30 mg/kg-d (DuPont 2008). The chronic bioassay in mice found no changes in absolute spleen weight, spleen weight relative to bodyweight, spleen weight relative to brain weight, or treatment-related changes in spleen histopathology in either sex after 9 or 18 mo of exposure (Chemours 2024; Thompson et al. 2026).

EPA (2021) also cites evidence for reductions in serum globulin and concomitant increases in albumin/globulin ratio (AGR) as evidence for immunotoxicity based on associations between globulin levels and reduced antibody production. The single source cited for this association is to a 2002 FDA document that appears to have been withdrawn and replaced with *Nonclinical Evaluation of the Immunotoxic Potential of Pharmaceuticals Guidance for Industry* (CDER 2023). It is not clear what was contained in the FDA document cited in EPA (2021) but CDER (2023) mentions neither albumin nor globulin. RIVM (2016) proposed a toxicity value for HFPO-DA based on changes in the AGR; however, the increase in AGR noted in RIVM was driven more by increased serum albumin, which is normally produced and released by hepatocytes. RIVM cited a single study as evidence for PPARα-induced increases in serum albumin following fenofibrate intake (Gervois et al. 2004); however, the changes reported therein were minimal based on measurements before and after 4 wk of treatment (63.1 g/L vs 64.0 g/L). We were unable to find any other sources reporting an association between PPARα agonist exposure and serum albumin changes in humans. Both chronic HFPO-DA studies considered the changes in albumin, globulin, and AGR nonadverse (Caverly Rae et al. 2015; Chemours 2024). As such, these changes were not considered further for cRfD derivation due to uncertain toxicological relevance.

As discussed elsewhere (Thompson et al. 2019), changes in hematological parameters in male rats were transient and not observed following lifetime exposure. At 12 mo, red blood cell (RBC), hemoglobin, and hematocrit in female rats were significantly decreased at 500 mg/kg group; however, these high doses are not drivers for an HFPO-DA risk assessment. In the new chronic mouse bioassay, no significant differences in hematological parameters were observed between high dose (5 mg/kg-d) and control female mice at 18 mo (Chemours 2024; Thompson et al. 2026). In male mice, statistically significant albeit ≤10% reductions in hemoglobin and hematocrit were observed in the 5 mg/kg-d group. Increases in red cell distribution width and increased large unstained cells were observed in male mice only at 5 mg/kg-d. These hematological changes did not correlate with macroscopic or microscopic pathology findings and were not considered adverse by the study authors considering their uncertain toxicological relevance, appearance in only one sex, and their occurrence only at the highest doses. Hematological changes were not considered further for cRfD.

###### Reproductive and developmental toxicity

**Fig. 4.**
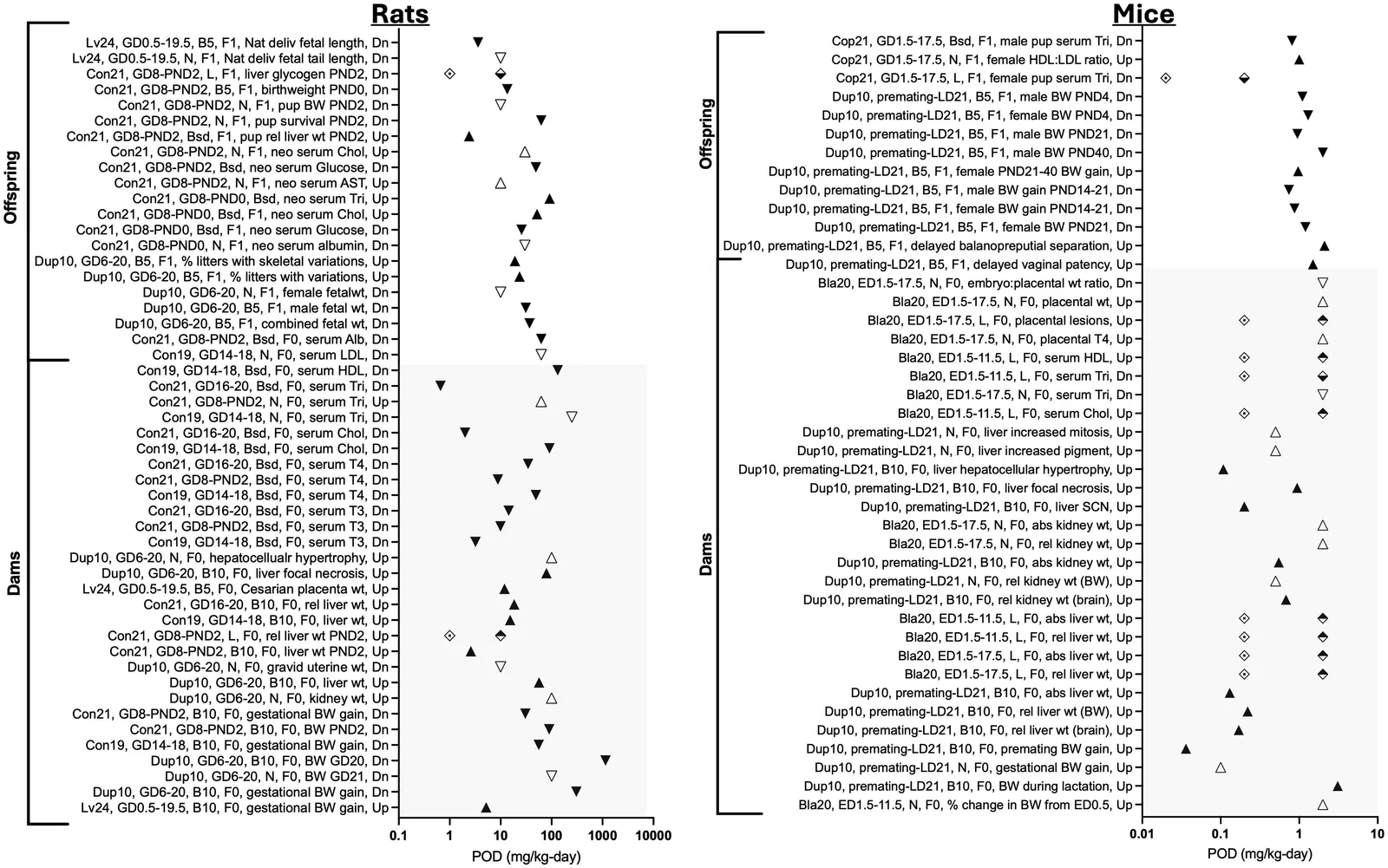
Array of adverse effects reported in rat and mouse dams and offspring. Solid and open triangles represent BMDL and NOAEL values, respectively; the direction of the triangle (up or down) indicates the direction of the effect. Diamonds indicate LOAELs, with the shaded half indicating the direction of the effect; diamonds with dots represent a 10-fold reduction of the LOAEL values to facilitate dose comparisons. Each effect is coded by study, timing of exposure, POD type (N = NOAEL, L = LOAEL, B10 = BMDL_10_, B5 = BMDL_5_, Bsd = BMD_1SD_), effect, and direction. Symbols in the lightly shaded region are for dams. Lv24, Lv et al. (2024); Con21, Conley et al. (2021); Con19, Conley et al. (2019), Dup10, DuPont (2010 rats or mice), Bla20, Blake et al. (2020); Cop21, Cope et al. (21). See text and Tables 4 and 5 for additional details.

An important motivation for this updated RfD is the number of existing and new reproductive and developmental toxicity studies conducted with HFPO-DA. Although EPA (2021) did not develop any candidate RfD values for reproductive and developmental effects, concern for such was stated as rationale for EPA increasing their UF_D_ from 3-fold to 10-fold between their draft (2018) and final (2021) toxicological reviews (U.S. EPA 2018, 2021). For this reason, reproductive and developmental studies on HFPO-DA are summarized below by species and endpoint and integrated in the section “Integration of reproductive and developmental toxicity in rats and mice.” Main effects in rats and mice are summarized semiquantitatively in Tables 4 and 5, respectively; and PODs for each effect are summarized in Fig. 4 to facilitate comparison of effects within and between species. The BMD modeling output results for rats and mice are shown in Files S2 and S3, respectively.

**Table 4.** Summary of select developmental effects in rats.

Study	Maternal BW	Placenta	Fetal metrics	Maternal and neonatal clinical chemistry
DuPont (2010b) GD6–20 0, 10, 100, 1,000 mg/kg	↓ BW gain @1,000 mg/kg	ND	↓ fetal wt @≥100 mg/kg (natural delivery) ↑ 14th rudimentary ribs @1,000 mg/kg	ND
Lv et al. (2024) GD0.5–19.5 0, 1, 10, 100 mg/kg Cesarian and natural delivery	↑ BW gain @≥10 mg/kg (Cesarian and natural)	↑ placenta wt @≥10 mg/kg (Cesarian delivery) ↑ placental histopathological changes (↑neutrophils, ↑cellular degeneration, ↑congestion) @≥10 mg/kg ↓ fetal: Placenta weight ratio @100 mg/kg (Cesarian delivery)	↓ fetal wt @≥10 mg/kg (natural delivery) ↓ fetal length @≥10 mg/kg (Cesarian and natural) ↓ fetal length tail length @100 mg/kg (Cesarian and natural)	ND
Conley et al. (2019) GD14–18 0, 62.5, 125, 250, 500 mg/kg	↓ BW gain @≥250 mg/kg	ND	ND	GD18 ↓ mat serum triglycerides @500 mg/kg ↓ mat serum LDL @≥125 mg/kg ↓ mat serum HDL @≥250 mg/kg ↓ mat serum Chol @≥250 mg/kg ↓ mat serum T3 @≥30 mg/kg ↓ mat serum T4 @≥125 mg/kg
Conley et al. (2021) GD8-PND2 0, 10, 30, 62.5, 125, 500 mg/kg GD16–20 0, 1, 3, 10, 30, 62.5, 125, mg/kg	GD8-PND2 ↓ BW gain @≥125 mg/kg	ND	PND0 ↓ birthweight @≥30 mg/kg PND2 ↓ pup wt @≥30 mg/kg ↓ survival @≥62.5 mg/kg ↑ rel liver weight @≥10 mg/kg	GD8-PND2 *PND0* ↑ neonatal serum triglycerides @≥125 mg/kg ↑ neonatal serum Chol @≥125 mg/kg ↓ neonatal serum glucose @≥62.5 mg/kg *PND2* ↑ mat serum triglycerides @≥125 mg/kg ↓ mat serum T3 and T4 @≥62.5 mg/kg ↑ neonatal serum Chol @≥62.5 mg/kg ↓ neonatal serum glucose @≥125 mg/kg ↓ neonatal liver glycogen @≥10 mg/kg ↑ neonatal serum AST @≥30 mg/kg GD16–20 ↓ mat serum triglycerides @≥10 mg/kg ↓ mat serum Chol @≥30 mg/kg ↓ mat serum T3 and T4 @≥62.5 mg/kg

**Table 5.** Summary of select reproductive and developmental changes in mice.

Study	Maternal BW	Placenta	Fetal metrics	Maternal and neonatal clinical chemistry
DuPont (2010c) Mating through lactation (∼60 d) 0, 0.1, 0.5, 5 mg/kg	↑ BW gain @≥0.5 mg/kg	ND	↓ pup wt PND4–21 @5 mg/kg ↓ pup survival @5 mg/kg (not significant)	ND
Blake et al. (2020) E1.5–11.5 0, 2, 10 mg/kg E1.5–17.5 0, 2, 10 mg/kg	E11.5 ↑ BW gain @10 mg/kg E17.5 No change in BW gain ↑ BW gain by mixed effect model @10 mg/kg	↑ placenta wt @10 mg/kg ↓ embryo: Placenta weight ratio @10 mg/kg E17.5 ↑ labyrinth atrophy @≥2 mg/kg ↑ labyrinth congestion, labyrinth necrosis, and fibrin clots @10 mg/kg.	No change in fetal wt	E11.5 ↓ mat serum triglycerides @≥2 mg/kg E17.5 ↓ mat serum triglycerides @10 mg/kg (possibly 2 mg/kg as well)
Cope et al. (2021) GD1.5–17.5 0, 0.2, 1, 2 mg/kg	No change	ND	PND0.5 No change in pup wt PND5.5 Mixed effect model estimated ∼6–7% decreases in pup wt @≥1 mg/kg PND22 No change in pup wt	PND22 ↓ female pup serum triglycerides @≥0.2 mg/kg ↓ male pup serum triglycerides @≥1.0 mg/kg

###### Reproductive and developmental toxicity in rats

No reproductive toxicity studies were identified in rats. Four developmental toxicity studies were evaluated in rats (DuPont 2010b; Conley et al. 2019, 2021; Lv et al. 2024). DuPont (2010b), an OECD TG 414 Prenatal Developmental Toxicity Study, exposed dams to 0, 10, 100, or 1,000 mg/kg-d from GD6-20 (15 d); fetuses were examined at study termination on GD20. Conley et al. (2019) exposed rats to 0, 62.5, 125, 250, or 500 mg/kg-d from GD14–18 (5 d); dams and fetuses were examined at study termination (GD18). Conley et al. (2021) exposed rats to 0, 10, 30, 62.5, 125, or 250 mg/kg-d from GD8-PND2 (∼15 d) in one experiment and to 0, 1, 3, 10, 30, 62.5, or 125 mg/kg-d from GD16-20 (5 d) in a second experiment. Lv et al. (2024) exposed rats to 0, 1, 10, or 100 mg/kg-d from GD0.5–19.5 (19 d), covering the longest portion of the gestation period among the rat studies. Half the dams underwent Cesarean section 2 hr after dosing on GD 19.5 and half the dams delivered naturally. Placenta were examined in the Cesarean dams only. The main findings in these four studies were grouped into four broad categories: Maternal bodyweight, placental changes, fetal metrics, and clinical chemistry changes in dams and offspring (Table 4). These and other effects were subjected to BMD modeling (see Materials and methods) and arrayed in Fig. 4 to help identify the most sensitive endpoints in dams and offspring.

###### Maternal changes in bodyweight and organ weight in rats

DuPont (2010b) reported statistically significant decreases in *bodyweight gain* at 1,000 mg/kg-d, with no indication of weight gain changes at 10 or 100 mg/kg-d. Maternal *bodyweight* was also significantly reduced at 1,000 mg/kg-d on GD20 and GD21. Conley et al. (2021) reported statistically significant decreases in gestational weight gain and bodyweight on PND2 in dams exposed 15 d (GD8-PND2) at ≥125 mg/kg-d, whereas no significant changes were observed in dams exposed only 5 d (GD16–20) up to 125 mg/kg-d. In contrast, Conley et al. (2019) reported significant decreases in gestational weight gain at ≥250 mg/kg-d after 5 d of exposure (GD14–18). Lv et al. (2024) reported statistically significant *increases* in gestational weight gain at 10 and 100 mg/kg-d. Based on DuPont (2010b) and the Conley et al. studies, exposures above 62.5 mg/kg-d can decrease gestational weight gain. Lv et al. (2024) reported increases in gestational weight gain at ≥10 mg/kg-d after 19 d of exposure (GD0.5–19.5). The difference in direction of weight changes reported in Lv et al. might relate to duration and/or timing of exposure (see “Integration of reproductive and developmental toxicity in rats and mice” section). In DuPont (2010b), kidney weight was increased at 1,000 mg/kg-d; however, the study authors did not consider this adverse due to the absence of microscopic changes. Decreases in uterine weight were attributed to lower fetal bodyweight by the study authors.

###### Placental effects in rats

DuPont (2010b) states that placentae were examined although there were no weight or histopathological results presented. Lv et al. (2024) was the only rat study to report effects on placenta, and only in rats that delivered by Cesarean. Statistically significant increases in placental weight were observed at ≥10 mg/kg-d, with values in the control and highest dose groups of 0.35 ± 0.07 g and 0.46 ± 0.04 g, respectively. Increases in placental histopathological changes (increased neutrophils, increased cellular degeneration, increased congestion) were reported at ≥10 mg/kg-d; however, incidence data were not provided. Statistically significant decreases in the fetal: Placenta weight ratio occurred at 100 mg/kg-d.

###### Offspring metrics in rats

Significant reductions in fetal weight were observed at ≥100 mg/kg-d but not at 10 mg/kg-d in DuPont (2010b). Fetal weight following natural delivery was significantly decreased at ≥10 mg/kg-d in Lv et al. (2024), whereas fetal weight was not affected in fetuses delivered by Cesarian section on GD19.5. This might indicate that fetal growth retardation (FGR) occurs late in gestation; however, fetal length was significantly decreased at ≥10 mg/kg-d in both Cesarian and natural delivery groups. Similarly, fetal tail length was decreased in Cesarian and natural delivery groups exposed to 100 mg/kg-d. Taking data from DuPont (2010b) and Lv et al. (2024) together, reductions in fetal weight likely occur somewhere around 10 mg/kg-d. Fetal malformations (14th rudimentary ribs) were increased at 1,000 mg/kg-d (albeit not significantly) and the study authors did not consider the affect adverse (DuPont 2010b).

Pup birthweight and bodyweight on PND2 were significantly reduced at ≥30 mg/kg-d in Conley et al. (2021). Survival was reduced at ≥62.5 mg/kg-d, the same dose where neonatal serum glucose was significantly reduced. Increased relative liver weight was observed in pups at ≥10 mg/kg-d HFPO-DA on PND2 (Table 4; Fig. 4).

###### Maternal and neonatal clinical chemistry changes in rats

Serum triglyceride levels were decreased in dams exposed to HFPO-DA from GD14–18 (5 d) albeit significantly only at 500 mg/kg-d (Conley et al. 2019). Serum triglyceride levels were significantly decreased at ≥10 mg/kg-d in dams exposed to HFPO-DA from GD16–20, sampled on GD20 (Conley et al. 2021). However, by PND2, the serum triglycerides were significantly higher at ≥125 mg/kg. Importantly, maternal serum triglycerides normally increase in rodents and humans during gestation (Cortés-Vásquez et al. 2018; Chen et al. 2022). Although hypertriglyceridemia can result in adverse pregnancy outcomes, natural increase in triglycerides during pregnancy serves to as an energy source or substrate for fetal development (*ibid*). As such, the dose-dependent decrease in maternal serum triglycerides opposes normal gestational trends. Considering that one of the primary functions of pharmaceutical PPARα activators such as fibrates is to decrease serum triglycerides (Kraja et al. 2010), exposure to PPARα activators during gestation may pose a risk. Indeed, PPARα activators are only recommended during pregnancy when elevated serum triglycerides pose maternal health risks (Cortés-Vásquez et al. 2018). Neonatal serum triglycerides were significantly increased at ≥125 mg/kg-d at PND0 but not PND2 (Conley et al. 2021).

Decreases in serum cholesterol were observed in dams exposed to ≥30 mg/kg-d HFPO-DA from GD16–20 (Conley et al. 2021). Serum cholesterol and HDL were decreased in dams exposed to ≥125 mg/kg-d from GD14–18 (Conley et al. 2019). LDL was decreased at ≥125 mg/kg-d (*ibid*). PPARα activation is associated with decreases in HDL-cholesterol in rodents, whereas fibrates typically increase HDL-cholesterol in humans (Kersten 2008). Moreover, PPARα activation generally increases APOA1 (a gene that codes for portions of HDL) in human liver but decreases *Apoa1* expression in rodents (Berthou et al. 1996; de la Rosa Rodriguez et al. 2018). HFPO-DA has been shown to reduce *Apoa1* expression in mouse liver (Chappell et al. 2020) and nongravid rat liver (Heintz et al. 2026), as well as in mouse and rat hepatocytes (Heintz et al. 2024a, 2024b). Neonatal serum cholesterol was increased at birth (PND0) and at PND2 from dams exposed from GD8-PND2 whereas *Apoa1* gene expression was decreased at PND0 in pup livers from dams exposed from GD8-PND2 (Conley et al. 2021) (Table 4; Fig. 4).

HFPO-DA consistently decreased serum T3 and T4 levels in dams in Conley et al. (2012, 2019) (see Table 4; Fig. 4). PPARα activators have been shown to decrease thyroid hormone levels (Miller et al. 2001), possibly through PPARα-dependent induction of CAR and subsequent induction of hepatic enzymes involved in the T3 catabolism (Wieneke et al. 2009). In addition, activated PPARα competes with the thyroid hormone receptor for their shared heterodimer partner RXR. PFAS can also bind to the T4 carrier protein transthyretin and thus displace T4 (Miller et al. 2001). These mechanisms are anticipated to reduce serum T4 levels in rodents; however, species differences in thyroid hormone catabolism and serum transport proteins suggest that rodents are more sensitive to these effects compared with humans (Rogers et al. 2023) but were considered for cRfD derivation.

HFPO-DA decreased neonatal serum glucose levels at PND0 at ≥62.5 mg/kg-d and PND2 at ≥125 mg/kg-d; these changes were likely related to glycogen depletion in pup livers at ≥10 mg/kg-d resulting in decreased early postnatal survival (Conley et al. 2021) (Table 4; Fig. 4). Hepatic glycogen is the neonatal glucose energy source prior to the onset of gluconeogenesis and fatty acid oxidation and thus depletion of glycogen stores can rapidly lead to hypoglycemia. Notably, experimentally induced reductions in neonatal glycogen reserves and hypoglycemia from maternal dietary glucose restriction have been shown to increase early postnatal mortality (Lanoue et al. 1999). Studies in wild-type and PPARα null mice exposed to other PPARα-activating PFAS (e.g. PFOA) exhibited increased postnatal mortality in wild-type but not PPARα null mice (Abbott et al. 2007). However, litter resorption was increased at ≥5 mg/kg-d PFOA in both genotypes (*ibid*). A subsequent study for PFOA demonstrated increased postnatal mortality in wild-type but not PPARα null mice or transgenic mice expressing human PPARα (Albrecht et al. 2013). This study by Albrecht et al. (2013) reported no effects on resorption—likely because the highest dose (3 mg/kg-d PFOA) was below the dose where resorptions were increased in Abbott et al. (2007). Notably, increased resorptions or other indications of reduced fetal survival have not been observed in studies on HFPO-DA (DuPont 2010b, 2010c; Conley et al. 2021). These findings suggest the increased postnatal mortality following HFPO-DA may be dependent on PPARα signaling pathways, whereas resorptions observed with PFOA (Abbott et al. 2007) may be independent of PPARα (note the New Jersey Drinking Water Quality Institute Health Effects Subcommittee (2017) has levied criticisms of Albrecht et al. (2013) study that may be of interest to some readers: https://nj.gov/dep/watersupply/pdf/pfoa-appendixa.pdf). Additionally, differences in human glycogen storage indicate that human neonates are not susceptible to the same energy depletion as rodents (Rogers et al. 2023). Once glycogen is depleted, rodents may be susceptible to death because newborn rats are highly dependent on their environment for thermoregulation but do exhibit high metabolic rates capable of doubling to offset any cooling (Gordon 1990). As such, low glycogen stores in newborn rodents might be quickly depleted attempting to maintain core body temperature and basic metabolic functions. Based on the above, Rogers et al. (2023) concluded that humans may be less susceptible to the early perinatal effects and neonatal mortality observed in HFPO-DA-exposed rats. Finally, increased serum AST was observed in pups at ≥30 mg/kg-d HFPO-DA in dams exposed from GD8-PND2 (Conley et al. 2021) (Table 4; Fig. 4).

###### Reproductive and developmental toxicity in mice

A single reproductive toxicity study was identified in mice. DuPont (2010c), an OECD TG 421 Reproduction/Development Toxicity Screening Test, exposed female mice to 0, 0.1, 0.5, or 5 mg/kg-d HFPO-DA for 14 d prior to mating, throughout gestation, and through lactation day 20 (∼60 d). Male mice were exposed for ∼90 d. No adverse effects on reproductive measures (mating, fertility, copulation indices, number of days between pairing and coitus, and gestation length) were observed.

Three developmental toxicity studies were evaluated in mice (DuPont 2010c; Blake et al. 2020; Cope et al. 2021). Following the exposure paradigm described above for DuPont (2010c), 1 male and 1 female from each litter were selected on PND21 for further exposure via oral gavage to the same HFPO-DA doses as the dams until PND40. Blake et al. (2020) exposed mice to 0, 2, or 10 mg/kg HFPO-DA from embryonic days 1.5 to 11.5 or 17.5. Cope et al. (2021) exposed mice to 0, 0.2, 1, or 2 mg/kg HFPO-DA from GD1.5–17.5; offspring were then fed a high- or low-fat diet at weaning until necropsy at 6 or 18 wk (this postnatal phase was not considered due to the dietary conditions). As with rats, effects were grouped into four broad categories covering toxicity in dams and offspring (Table 5); these and other effects were subjected to BMD modeling, converted by HED values by allometric scaling, and arrayed in Fig. 4 to help identify the most sensitive endpoints in dams and offspring.

###### Maternal changes in bodyweight and organ weight in mice

In DuPont (2010c), gestational weight gain was significantly increased at ≥0.5 mg/kg-d. Bodyweight gain was also increased in premating and postnatal phases relative to controls. Increased absolute and relative maternal liver weight was similar to those reported in nongravid mice (DuPont 2008, 2010a) and were consistent with peroxisomal proliferator phenotype. As in rats, increases in absolute and relative kidney weight were not associated with histopathological changes.

In Blake et al. (2020), gestational weight gain was significantly increased at 10 mg/kg-d at E11.5 but not E17.5. However, the authors reported significant increases at both timepoints at 10 mg/kg-d using mixed effect model accounting for litter size, etc. Kidney weight and relative kidney weight were increased at 10 mg/kg-d at E17.5 (no significant change was observed at E11.5). Histopathological evaluations of treated and control kidneys were considered “within normal limits” (Blake et al. 2020). Increases in maternal liver weight are consistent with peroxisomal proliferator phenotype, whereas the increase in maternal kidney weight without evidence of histopathological changes was not considered adverse (consistent with findings in DuPont 2010c). No significant changes in maternal bodyweight or organ weights were noted in Cope et al. (2021) with exposure only up to 2 mg/kg-d.

###### Placental effects in mice

Blake et al. (2020) was the only mouse study to report effects on placenta. Statistically significant increases in placental weight were observed at 10 mg/kg-d, along with a significant decrease in the embryo: Placental weight ratio. At E17.5, placental abnormalities were observed in all treatment groups (≥2 mg/kg-d) and tended to occur as litter-specific effects (e.g. most or all placenta within one litter were affected). Labyrinth atrophy was common at both 2 and 10 mg/kg-d, whereas labyrinth congestion, labyrinth necrosis, and fibrin clots were present at 10 mg/kg. No placental lesions were observed at E11.5.

###### Offspring metrics in mice

In DuPont (2010c), no significant differences in pup weights were observed at PND1; however, weights of male and female pups were significantly lower in the 5 mg/kg-d group by PND4 and remained lower at PND21. Consistent with these observations, bodyweight gain was significantly lower in the 5 mg/kg-d group from PND1–4 and at several intervals as late as PND14–21. By PND40, the female pup weight did not differ significantly from control mice, whereas the male pup weight remained significantly albeit ≤10% lower than controls. A slight (not statistically significant) decrease in pup survival on PND0, PND0–1, and PND0–4 was observed (all from a single litter in the 5 mg/kg-d group). Although the study authors did not consider this treatment related, subsequent information on the early postnatal survival in rats exposed to HFPO-DA (Conley et al. 2021) and other PFAS-related early mortality data (e.g. Albrecht et al., 2012) suggest that there might have been an effect on pup glycogen storage as reported in rats (Conley et al. 2021). In male and female mice, delayed balanopreputial separation and vaginal patency were observed in the 5 mg/kg-d group, respectively; however, the study authors attributed these changes to lower bodyweight in these groups (DuPont 2010c). Embryo weight was not significantly altered by HFPO-DA exposure in Blake et al. (2020). Cope reported no significant differences in pup weight at PND0.5 but estimated a 6.3% and 6.7% decrease in pup weight at 1 and 2 mg/kg-d at PND5.5 using a mixed effects model. No significant differences in pup weight were observed at PND22 (these mice were not exposed to HFPO-DA postnatally), indicating that the effects on pup weight were not persistent.

###### Maternal and neonatal clinical chemistry changes in mice

Triglyceride levels in maternal mice were significantly decreased in mice at ≥2 mg/kg-d at E11.5 and at 10 mg/kg-d at E17.5 (Blake et al. 2020) (triglyceride also appear to be significantly decreased at 2 mg/kg (257±120.3 vs 472.5 ± 78.9)). Cope et al. (2021) reported statistically significant decreases in serum triglycerides at ≥0.2 mg/kg-d and ≥1 mg/kg-d in female and male mice at PND22, respectively. Notably, these mice were not exposed to HFPO-DA postnatally.

###### Integration of reproductive and developmental toxicity in rats and mice

Exposure to HFPO-DA has mixed effects on maternal gestational weight gain. In mice, studies consistently showed an increase in gestational weight gain with increasing HFPO-DA exposure. Notably, exposure in the mouse studies started before gestation or by E1.5. In rats, gestational weight was increased in Lv et al. (2024), which began exposure at E0.5. All other rat studies began exposure on GD6, GD8, or GD14, and were associated with decreases in gestational weight gain. This suggests that duration and/or timing of exposure influences the direction of gestational weight changes. Increases in placental weight, histological placental lesions, and reductions in fetal: Placental weight ratios were observed in both rats and mice (Blake et al. 2020; Lv et al. 2024).

Maternal serum triglyceride levels were also reduced in both species (Blake et al. 2020; Conley et al. 2021). Serum triglyceride levels in offspring differed by species. Conley et al. (2021) reported increases in neonatal serum triglycerides, whereas Cope et al. (2021) reported significant decreases in pup serum triglycerides at PND22 despite the dams were not exposed to HFPO-DA after GD17.5. According to Cope et al. the decrease in triglycerides was “transient and attenuated over time.” It is well established that serum triglyceride levels increase several fold in rodents and humans during gestation with an apparent rapid return to pregestational levels (Cortés-Vásquez et al. 2018; Chen et al. 2022). Based on maternal serum triglyceride levels in control dams from Conley et al. (2021), it appears that the median triglyceride levels at PND0 are ∼350 mg/dL and rapidly fall to ∼30 mg/dL by PND2, presumably near pregestational levels. Notably, the significant reductions in maternal triglycerides at ≥10 mg/kg-d HFPO-DA plateau at ∼150 mg/dL, which is still several fold higher than postpartum levels and presumably pregestational levels. Whether these ∼2-fold lower triglyceride levels pose a risk to the developing fetus is unknown. The apparent treatment-related *increase* in serum triglycerides at ≥125 mg/kg-d at PND2 are around 60–70 mg/dL, i.e. lower than on PND0 (∼150 mg/dL) but higher than unexposed (30 mg/dL). Thus, the “increase” at PND2 may simply reflect a slower return to baseline levels in highly exposed rats.

Examination of Fig. 4 indicates that reductions in serum triglycerides in dams and offspring are among the most sensitive effects. Increased placental weight and histopathological changes (e.g. increased congestion) were also observed on both species. The reproductive and developmental toxicity endpoints carried forward for RfD derivation are described in the next section.

### Deterministic RfD derivation

All chronic or reproductive/developmental critical effects were subjected to BMD modeling using appropriate BMR values (see Materials and methods) or the LOAEL/NOAEL approach if not amendable to modeling. Numerous POD (BMDL, NOAEL, LOAEL) values were compared to identify the most critical effects in each species, sex, and lifestage. For reproductive and developmental toxicity PODs presented in Fig. 4, only the most sensitive effects in mice and rats were carried forward for cRfD derivations as these PODs would naturally result in the lowest cRfD values for these toxicity domains (note: LOAEL values were decreased 10-fold in Fig. 4 to facilitate comparisons). For mice, these effects included placental lesions, reduced pup weight, and decreases in maternal and offspring serum triglyceride levels. Changes in other clinical chemistry endpoints in mice (e.g. cholesterol) resulted the same POD as serum triglycerides. Given that serum triglycerides normally increase during gestation, mitigation of this increase may have implications for growth and/or development in offspring and therefore effects on maternal and offspring serum triglyceride levels were considered for RfD derivation. Increases in placental weight and placental histopathological changes have been observed in both mice and rats, which may also have implications for fetal growth and development and therefore considered for RfD derivation. Increased kidney weight in dams resulted in slightly higher PODs; however, there were no apparent microscopic lesions (DuPont 2010b). A Society of Toxicologic Pathology position paper states that organ weight changes without macroscopic or microscopic correlation may not necessarily be adverse and should be interpreted with caution (Sellers et al. 2007). Kidney weight changes were not considered for cRfD derivation as these effects were not the most sensitive or severe effects. Parameters related to increased liver weight and bodyweight in dams were considered part of the PPARα-induced hepatomegaly. For rats, decreases in maternal serum triglycerides and relative liver weight and decreased pup liver glycogen were the most sensitive endpoints. As already noted, changes in liver glycogen storage likely affects postnatal growth and survival in rodents but its relevance to humans is uncertain (Rogers et al. 2023); nevertheless, this effect was caried forward for cRfD derivation. As in mice, maternal changes in liver weight parameters were considered part of the PPARα-related hepatomegaly and not carried forward for cRfD derivation. Overall, reductions in fetal weight and birthweight were observed in mice and rats and could be related to the effects on the placenta, serum lipids, or glycogen storage.

Data continue to indicate that the liver is one of the most sensitive target organs following HFPO-DA exposure; however, most effects are clearly mediated by PPARα in the mouse liver and likely mediated by PPARα in the rat liver. Given the overwhelming evidence that liver effects such as hepatocellular hypertrophy (also called cytoplasmic alteration), apoptosis, and necrotic cell death in both short-term and longer-term studies in mice are the result of the rodent-specific PPARα MOA for liver tumors (Heintz et al. 2023a, 2025), most of the liver lesions in mice and rats were concluded to have limited relevance for human health risk assessment (these are discussed further in the section “Comparison of new deterministic RfD to existing toxicity criteria”). However, as a result of the unknown etiology, the biliary hyperplasia observed in male mice following 18 mo of exposure was considered potentially relevant for human health risk assessment. The cystic focal necrosis observed in male rats has an unknown etiology and therefore was also considered for RfD derivation. EPA recently reached a similar conclusion about this liver lesion in rats in their assessment of the PPARα activator di-isononyl phthalate (USEPA 2024a). New chronic data in mice indicate heretofore unrecognized histopathological effects in the male mouse adrenal gland and testes after prolonged exposure to high doses and were considered for RfD derivation.

**Fig. 5.**
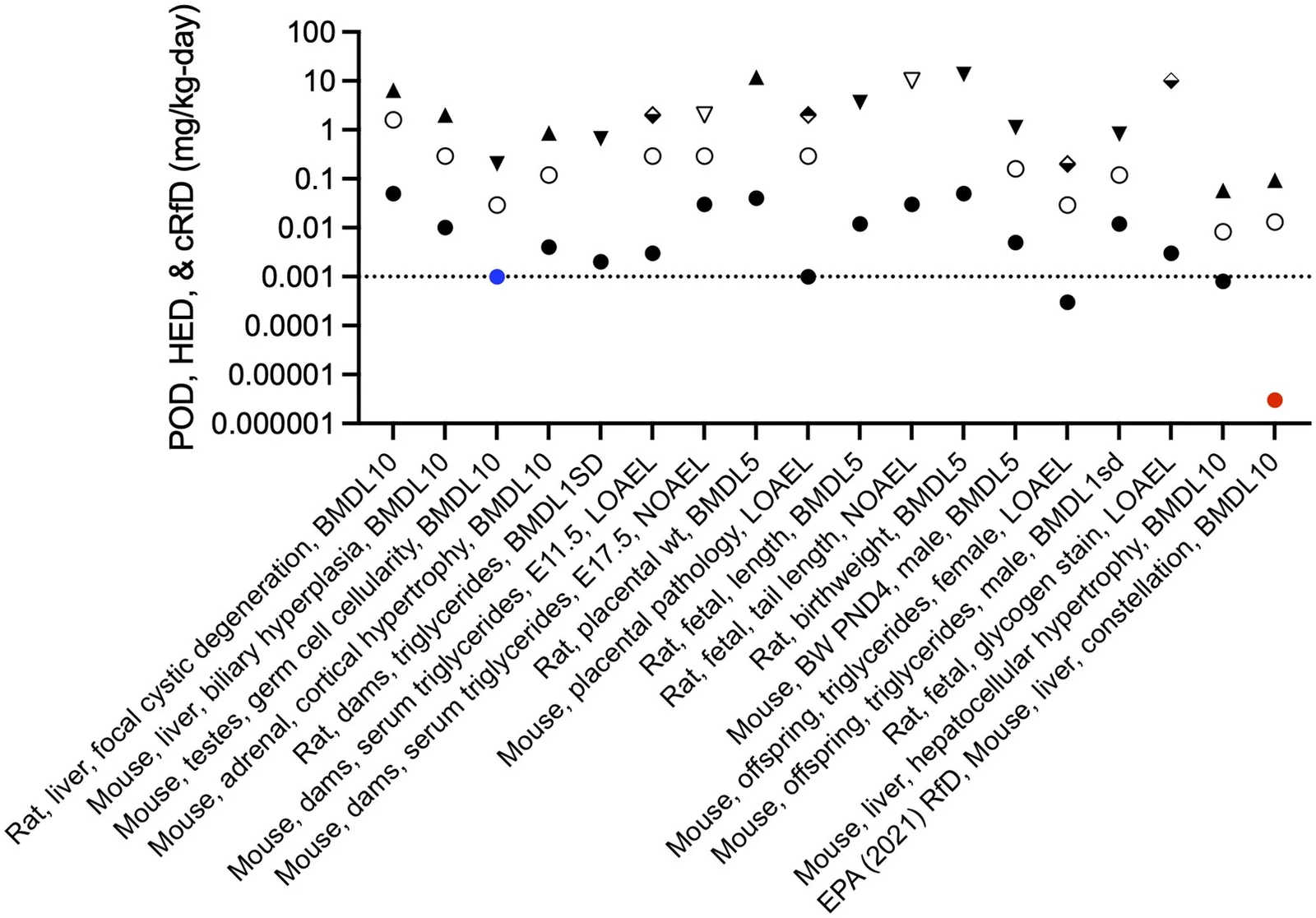
Array of cRfD values. Closed and open triangle represent BMDL and NOAEL values, respectively; the direction of the triangles (up or down) indicates the direction of the effect. Diamonds indicate LOAELs, with the shaded half indicating the direction of the effect. Open and closed circles indicate HEDs and cRfDs, respectively (see Table 5 for UFs). The solid blue circle represents the cRfD selected as the basis of the updated RfD. The solid red circle represents the EPA (2021) RfD based on a constellation of liver lesions and includes a 3,000-fold composite uncertainty factor. Also shown is a cRfD based on hepatocellular hypertrophy for comparison purposes only (see text).

The critical effects selected for cRfD derivation were subjected to BMD modeling (File S4), summarized in Table 6, and depicted in Fig. 5. The selected endpoints and POD values were converted to HED values using allometric scaling factors of 7 and 4 for mice and rats, respectively (U.S. EPA 2002). Additional considerations for female rats are described below when discussing the interspecies uncertainty factors.

**Table 6.** Candidate RfD values.

Endpoint and Study	POD (mg/kg-d)	POD Type	**HED** ^a^ **(mg/kg-d)**	UF_A_	UF_H_	UF_L_	UF_S_	UF_D_	UF_C_	cRfD (mg/kg-d)
Liver focal cystic degeneration in male rats; Rae (2015)	6.5	BMDL_10_	1.6	3	10	1	1	1	30	0.05
Biliary hyperplasia in male mice; Chemours (2024)	2.0	BMDL_10_	0.29	3	10	1	1	1	30	0.01
Reduced testes germ cell cellularity in mice; Chemours (2024)	0.2	BMDL_10_	0.029	3	10	1	1	1	30	**0.001**
Adrenal gland hypertrophy in male mice; Chemours (2024)	0.87	BMDL_10_	0.12	3	10	1	1	1	30	0.004
Reproductive and Developmental Endpoints										
Decreased serum triglycerides in rat dams; (GD16–20); Conley et al. (2021)	0.66	BMDL_1SD_	0.66^b^	30^b^	10	1	1	1	300	0.002
Decreased serum triglycerides in mouse dams, E11.5; Blake et al. (2020)	2	LOAEL	0.29	1^c^	10	10	1	1	100	0.003
Decreased serum triglycerides in mouse dams, E17.5; Blake et al. (2020)	2	NOAEL	0.29	1	10	1	1	1	10	0.03
Increased placental weight in female rats; Lv et al. (2024)	11.9	BMDL_5_	11.9	30	10	1	1	1	300	0.04
Increased placental lesions in female mice; Blake et al. (2020)	2	LOAEL	0.29	3	10	10	1	1	300	**0.001**
Decreased fetal length; Lv et al. (2024)	3.6	BMDL_5_	3.6	30	10	1	1	1	300	0.012
Decreased fetal tail length; Lv et al. (2024)	10	NOAEL	10	30	10	1	1	1	300	0.03
Decreased rat birthweight; Conley et al. (2021)	13.5	BMDL_5_	13.5	30	10	1	1	1	300	0.05
Decreased male mouse pup weight on PND4; DuPont (2010)	1.1	BMDL_5_	0.16	3	10	1	1	1	30	0.005
Decreased serum triglycerides in female mouse offspring; Cope et al. (2021)	0.2	LOAEL	0.029	1	10	10	1	1	100	0.0003
Decreased serum triglycerides in male mouse offspring; Cope et al. (2021)	0.81	BMDL_1SD_	0.12	1	10	1	1	1	10	0.01
Decreased pup liver glycogen; Conley et al.(2021)	10	LOAEL	10	30	10	10	1	1	3,000	0.003

a The HED was derived using an allometric scaling factor of 7 for mice and 4 for male rats. In lieu of allometric scaling, a 30-fold UF_A_ was applied to female rat dam and offspring endpoints (see text).

b Due to apparent differences in the clearance of HFPO-DA and the much higher doses administered to female rats, allometric scaling was not used. Instead, a 10-fold interspecies pharmacokinetic adjustment was applied (see text) along with a 3-fold interspecies pharmacodynamic adjustment.

c The pharmacodynamic portion of the UF_A_ was set to 1 for all serum triglyceride endpoints in mice based on evidence for reduced sensitivity to PPARα-mediated effects in humans (see text).

The uncertainty factors applied in the RfD derivation were as follows. A full 10-fold UF_L_ was applied to all endpoints that were not amenable to BMD modeling and that lacked a study NOAEL. Because all endpoints considered for cRfD derivation were based on either chronic studies or reproductive and developmental studies, a UF_S_ of 1 was used for all endpoints. A full 10-fold UF_H_ was applied to all endpoints, even though many of the endpoints were derived from sensitive lifestages (i.e. reproductive and developmental toxicity studies). The interspecies UF_A_ varied depending on endpoint. Although allometric scaling is widely considered to account for pharmacokinetic differences across species, some organizations consider allometric scaling to also account for toxicodynamic differences (TCEQ 2011) because (i) allometric scaling predicts toxicity (Schneider et al. 2004), which is a combination of toxicokinetics and toxicodynamics, and (ii) omission of the UF_A_ harmonizes with cancer assessments that take no specific account of toxicodynamics after allometric scaling (U.S. EPA 2005). Nevertheless, the default 3-fold UF_A_ was applied to endpoints that could not reasonably be attributed to PPARα activation at this time, including effects in the mouse adrenal gland and testes, cystic focal degeneration in rats, placental effects, and reduced bodyweight in offspring. For effects linked to PPARα activation, allometric scaling was considered sufficient to account for interspecies extrapolation as data indicate that humans are not likely to be more sensitive to PPARα activators than rodents. This is based on evidence that PPARα expression and activity levels are lower in humans than rodents (Palmer et al. 1998; Takacs and Abbott 2007; McMullen et al. 2020). A recent in vitro study with HFPO-DA and the PPARα agonist GW7647 reported ∼10-fold higher BMD values for PPARα genes in human primary hepatocytes as compared with mouse and rat hepatocytes (Heintz et al. 2024a, 2024b). In addition, HFPO-DA- and GW7647-exposed mouse hepatocytes had a greater number of concentration-responsive PPARα-mediated genes involved in bile acid homeostasis compared with human hepatocyte counterparts (Heintz et al. 2024a). These findings support the omission of a 3-fold UF_A_ for potential increased toxicodynamic sensitivity in humans, as the available data support *reduced* sensitivity in humans as compared with rodents. As such, a UF_A_ of 1 was applied to endpoints clearly related to PPARα (e.g. serum triglyceride levels).

Given the difference in HFPO-DA clearance in female rats (see “Toxicokinetic considerations” section), alternatives to allometric scaling were considered. EPA (2021) explored deriving HED values based on the ratio of clearance half-life in animals and humans but ultimately concluded that the half-life data for humans was insufficient. Furthermore, we note that EPA guidance on data-derived extrapolation factors explicitly states that half-life is not an acceptable basis for extrapolation factor derivation (U.S. EPA 2014). A recent toxicological review of perfluorohexanoic acid (PFHxA) used ratios of clearance data to derive species and sex specific dosimetric adjustment factors (DAFs) to derive HEDs for PFHxA (U.S. EPA 2023). Using a similar approach to that described therein, pharmacokinetic data from Gannon et al. (2016) and human half-life estimates described in the “Toxicokinetic considerations” section were used to explore an alternative to allometric scaling for female rats. As shown in Table S3, these analyses resulted in a DAF of 3.5 for male rats and 5.5 and 7.7 for male and female mice, respectively. These values are remarkably similar to the default allometric scaling factors of 4 and 7 for rats and mice, respectively (U.S. EPA 2002). As such, these results support the use of allometric scaling of PODs for mice and male rats. For female rats, the estimated DAF was 30.9, indicating that some adjustment other than allometric scaling may be warranted; however, the exact value is uncertain given the limited data. Consistent with the ∼10-fold higher doses used in female rat studies and the female rat DAF estimate, a 30-fold UF_A_ (10 for pharmacokinetics; 3 for pharmacodynamics) was applied to female rat endpoints (including offspring) in lieu of allometric scaling.

Deciding on an appropriate UF_D_ is complicated by the fact that the EPA has no clear guidance on the UF_D_ and review of recent EPA assessments indicates no discernable internal consistency. The subjective nature of the UF_D_ is exemplified in EPA toxicological reviews of HFPO-DA. The EPA (2018) draft toxicological review of HFPO-DA applied a UF_D_ of 3 that was justified, in part, based on the “lack of a full two-generation reproductive toxicity study evaluating exposures during early organogenesis (i.e. GD 0 to GD 6) and studies evaluating additional developmental endpoints that have been observed following exposure to other PFAS…” and the “lack of a chronic study in the mouse, which appears to be more sensitive than the rat…”. In the EPA (2021) final review, the UF_D_ was *increased* to 10 despite the availability of more mechanistic and toxicity studies.

The UF_D_ herein was set to 1. This is largely supported by the availability of chronic bioassays in two species, several subchronic studies, the availability of specialty studies (e.g. Rushing et al., 2017), and multiple guideline-based contract laboratory reproductive and developmental toxicity studies as well as academic reproductive and developmental toxicity studies (DuPont 2010b, 2010c; Conley et al. 2019, 2021; Blake et al. 2020; Cope et al. 2021; Lv et al. 2024). It was also concluded that additional reproductive studies in rats would likely be uninformative for risk assessment given the much higher doses needed to overcome the faster clearance of HFPO-DA in female rats and the fact that mice are highly sensitive to the PPARα effects of HFPO-DA and any multigenerational studies would likely be either confounded by or a consequence of PPARα signaling in the liver. In addition, HFPO-DA does not bioaccumulate. As such, the absence of a two-generation study was not deemed a deficiency in the HFPO-DA database.

Figure 5 depicts an array of cRfD values for the aforementioned outcomes; the POD, HED, UFs, and cRfD values are presented in Table 6. For reference, the EPA (2021) RfD based on EPA’s constellation of liver lesions in mice is shown in Fig. 5 on the far right. The lowest cRfD was based on serum triglyceride levels in female offspring (0.0003 mg/kg-d); however, this was due to the use of a LOAEL of 0.2 mg/kg-d necessitating a 10-fold UF_L_. Considering the overall similarity in response in male and female offspring reported in Cope et al. (2021) and the ability to model the male data, the cRfD for male offspring (0.01 mg/kg-d) was considered to have less uncertainty and therefore was considered to better represent this outcome. Reduced maternal serum triglycerides cRfD values ranged from 0.002 to 0.03 mg/kg-d. The next lowest cRfD was based on placental lesions in mice (0.001 mg/kg-d). Reduced cellularity in the mouse testes results in an identical cRfD of 0.001 mg/kg-d. Decreased offspring weight resulted in higher cRfD values, as did adrenal hypertrophy and billiary hyperplasia in male mice and focal cystic degeneration in male rats.

Overall, the cRfD values for placental lesions in mice and reduced cellularity in mouse testes, both 0.001 mg/kg-d, were considered the most sensitive outcomes for HFPO-DA. Because the testes effects in mice were based on BMD modeling, whereas the placental effects in mice were based on a LOAEL, and because of the overall lower UF_C_ (30 vs 300) the final RfD was based on reduced cellularity in the testes.

### Probabilistic RfD derivation

Thompson et al. (2019) derived deterministic and probabilistic RfD (pRfD) values for HFPO-DA that were within 2-fold of one another. The pRfD was proposed as it was anticipated that EPA would begin implementing probabilistic methods into risk assessment. Indeed, several EPA authors (Blessinger et al. 2020) applied a so-called unified probabilistic framework described elsewhere (Chiu and Slob 2015; Chiu et al. 2018; WHO 2018) in a case study assessment of acrolein. Although EPA has yet to codify a probabilistic approach for risk assessment, for consistency with Thompson et al. (2019), we sought to compare our updated deterministic RfD of 0.001 mg/kg-d to pRfD values derived with the WHO approximate probabilistic analysis (APROBA) spreadsheet. The inputs of this analysis are described in Table 7 along with the computed pRfD of 0.0005 mg/kg-d.

**Table 7.** Probabilistic RfD values for reduced cellularity in testes.

Description	Value	Notes
	*Input*	
Type of endpoint	Quantile-deterministic	Histopathological endpoint
Target BMR (M)	10% extra risk	Effect size
BMDL (POD)	0.20 mg/kg-d	BMDS modeling result
BMDU	0.53 mg/kg-d	BMDS modeling result
Population incidence goal (I)	1%	User target
Allometric oral scaling (LCL; UCL)	8.02; 15.21	APROBA spreadsheet
Remaining interspecies UF (LCL; UCL)	0.33; 3	APROBA spreadsheet
Intraspecies UF (LCL; UCL)	2.24; 41.88	APROBA spreadsheet
	*Output*	
Target human dose (HD_M_^I^; HD_10%_^1%^) LCL	0.0005 mg/kg-d	APROBA spreadsheet
Target human dose (HD_M_^I^; HD_10%_^1%^) UCL	0.02 mg/kg-d	APROBA spreadsheet

### Comparison of new deterministic RfD to existing toxicity criteria

The proposed RfD of 0.001 mg/kg-d is more than 300-fold higher than the EPA (2021) RfD of 0.000003 mg/kg-d that was based on a constellation of liver lesions in subchronic mouse studies (red dot in Fig. 5). As was shown in Fig. 3, the POD for EPA’s constellation of liver lesions was comparable to the most sensitive lesion, hepatocellular hypertrophy. Importantly, PODs for hepatocellular hypertrophy did not change meaningfully with increased exposure duration. Moreover, some of the lesions comprising EPA’s constellation of liver lesions (e.g. single cell necrosis) were less evident with chronic exposure. It is our contention that EPA’s constellation of liver lesions are all part of the sequela associated with the PPARα MOA for which most of the key events leading to tumor formation in rodents lack human relevance (Corton et al. 2018; Felter et al. 2018). Conservatism inherent to EPA’s selection of a sensitive endpoint with limited human relevance was compounded by the application of a 3000-fold UF_C_ after applying an ∼7-fold allometric scaling factor. This large UF_C_ was driven by the application of a 10-fold UF_S_ that is now unwarranted based on new data in Fig. 3 as well as the 10-fold UF_D_ that was applied based on EPA’s concerns about reproductive/developmental toxicity rather than deriving cRfD values based on available reproductive/developmental data.

Conservatism in the EPA (2021) RfD for HFPO-DA was objectively assessed by Pham et al. (2020) who demonstrated that RfD values tend to be approximately 6-fold higher than their respective TTC value—indicating that, as expected, TTC values are conservative surrogates of toxicity. Pham et al. proposed that chemicals with a log(RfD: TTC ratio) more than ±2 standard deviations from a mean of 0.79 (−1.1, 2.7) might warrant re-evaluation due to potential over- or underconservatism in the RfD. The EPA (2021) RfD for HFPO-DA (0.000003 mg/kg-d) is 500-fold *lower* than the Cramer Class III TTC (0.0015 mg/kg-d) and the log(RfD: TTC ratio) is more than 2 standard deviations from the mean. These potential indicators of overconservatism are consistent with the above discussion related to EPA’s choice of PPARα-related liver lesions and application of a 3000-fold UF_C_ in EPA (2021). Although PFAS were not included in the original Munro dataset, it has been shown that the addition of PFAS among Cramer Class III Munro database significantly expanded the chemical space without significantly altering the Cramer Class III TTC value (Lea et al. 2022). Our prosed RfD of 0.001 mg/kg-d is still slightly lower than the TTC but has a log(RfD: TTC ratio) within ±2 standard deviations of mean log(RfD: TTC ratio). These findings indicate that our proposed RfD is conservative, yet within the normal variation of IRIS RfD: TTC ratios. Finally, it is notable that a cRfD based on the BMDL_10_ for the sensitive, rodent-specific, and PPARα-related effect of hepatocellular hypertrophy in male mice at 18 mo of exposure would result in a value of 0.0008 mg/kg-d (cRfD = 0.058 mg/kg-day ÷ 7 ÷ 10 (UF_A_ = 1; UF_H_ = 10) = 0.0008 mg/kg-day), which is similar to the proposed RfD of 0.001 mg/kg-d.

## Discussion

Short-chain alternatives to long-chain PFAS such as PFOA and perfluorooctanesulfonic acid (PFOS) were developed to avoid bioaccumulation in mammals and thus reduce their potential toxicity. However, some of these alternatives may be more or less potent on a molar concentration basis, i.e. internal concentration versus applied or ingested dose. Indeed, HFPO-DA is a potent activator of PPARα but its rapid clearance and the lack of bioaccumulation in tissues limits its toxicity profile. As reviewed herein, the most sensitive effects occur in the liver. The direct action of HFPO-DA on the liver has been unequivocally demonstrated to be PPARα dependent (Heintz et al. 2024a, 2024b, 2025), with effects including hepatocellular hypertrophy (also called cytoplasmic alteration), apoptosis, and necrotic cell death consistent with the upstream key events (KEs) of the PPARα MOA for rodent liver tumors (Heintz et al. 2023a). As such, these liver endpoints were not considered in the derivation of noncancer toxicity criteria. Additionally, it is well established in the peer-reviewed literature that liver tumors occurring via the PPARα MOA are not relevant to humans (Corton et al. 2018; Yamada et al. 2025). As such, we did not develop a CSF for HFPO-DA, a decision consistent with those recently made by EPA for HFPO-DA (U.S. EPA 2021) and other PPARα activators (USEPA 2024a).

Many newer studies on HFPO-DA have focused on reproductive and developmental toxicity. Broadly, there is no evidence for adverse structural abnormalities. Some studies reported changes in fetal liver transcriptomics in response to HFPO-DA (Conley et al. 2021; Blake et al. 2022). For example, Conley et al. (2021) reported that both maternal and fetal liver gene changes included PPAR signaling. Nevertheless, there is no precedent for the use of such endpoints for human health risk assessment. Observed lower birthweight and/or increased perinatal death could be due to reductions in fetal glycogen storage leading to energy depletion, however, as discussed in Rogers et al. (2023), there are critical species differences that attenuate the relevance of these effects for humans (see “Reproductive and developmental toxicity in rats” section). Changes in placental weight and/or histopathology could be independent of PPARα activation in the liver and thus have potential human relevance (*ibid*). Moreover, as discussed in the section “Reproductive and developmental toxicity in rats”, the changes in maternal and pup serum triglyceride levels could have implications for offspring development. Notably, several studies have reported associations between exposure to longer chain PFAS like PFOA and PFOS and low birthweight in humans (U.S. EPA 2024a, 2024b).

As with the developmental effects, the chronic effects in the male mouse adrenal cortex and testes might also be secondary to PPARα activation in the liver, as both effects have been observed in wild-type but not PPARα null mice exposed to PPARα activators (Wang et al. 2010; Li et al. 2011). At present, however, more research is needed to understand the etiology and human relevance of these changes. As such, these endpoints were considered for development of an update RfD.

Overall, the testicular effects in the mouse chronic bioassay were determined to be among the most sensitive effects and was observed in a robust OECD TG 453 chronic bioassay. This endpoint resulted in an RfD of 0.001 mg/kg-d. As in Thompson et al. (2019), this deterministic RfD is ∼2-fold higher than the probabilistic RfD. As EPA has yet to fully implement probabilistic RfD values in their risk assessments, the deterministic RfD is proposed as the updated RfD for HFPO-DA. This RfD is 10-fold lower than the RfD published in Thompson et al. (2019)—a difference largely driven by effects observed in the new mouse chronic bioassay and new reproductive/developmental studies in mice. The updated RfD is more than 300-fold higher than the RfD derived in EPA (2021). This difference is largely driven by the conservative assumptions made in the derivation of the EPA (2021) RfD, including the use of the constellation of liver lesions and the application of a 10-fold UF_S_ that, as shown in Fig. 3, is not supported because the POD does not meaningfully decrease with longer exposure duration. Additionally, the availability of new studies addressing chronic toxicity in a second and more sensitive species (i.e. mice) and reproductive/developmental toxicity warrant reductions to the UF_D_.

The new mechanistic evidence related to the liver effects observed in rodents and the lack of human relevance of such, along new data from a chronic study in a second species, demonstrates a need to re-evaluate the RfD for HFPO-DA developed in EPA (2021) that was based on early PAPRα liver effects driven largely by hepatocellular hypertrophy in a subchronic study. The need for re-evaluation is further demonstrated by the analyses in the “Comparison of new deterministic RfD to existing toxicity criteria” section which indicate that the EPA (2021) RfD of 0.003 µg/kg-d (0.000003 mg/kg-d) is 500-fold lower than the Cramer Class III TTC of 1.5 µg/kg-d when, on average, RfD values are higher than TTC values (Pham et al. 2020). In contrast, the updated RfD herein is similar to the TTC (1 vs 1.5 µg/kg-d). In conclusion, the new information warrants an update to the RfD for HFPO-DA. The RfD of 0.001 mg/kg-d presented herein represents the current best available science.

## Supplementary Material

kfag045_Supplementary_Data
