# Supplementary material for: Integration of mechanistic and repeat dose toxicity data in the derivation of an oral reference dose for HFPO-DA
Source: Toxicol Sci. 2026 Apr 10;209(5):kfag045. doi: 10.1093/toxsci/kfag045 (PMC13176454; doi:10.1093/toxsci/kfag045)
Supplement: kfag045_Supplementary_Data [file kfag045_supplementary_data.zip › Supplementary File S3.docx]

Table of Contents

[Delayed Balanopreputial Separation, Ms, F1, M, Premating-LD21, Dupont (2010) 3](#_Toc223538032)

[Delayed vaginal latency, Ms, F1, F, Premating-LD21, Dupont (2010) 7](#_Toc223538033)

[Serum HDL:LDL ratio, Ms, F1, F, Cope (2021) 11](#_Toc223538034)

[Offspring BW, Ms, M, PND4, Dupont (2010) 15](#_Toc223538035)

[Offspring BW, Ms, M, PND21, Dupont (2010) 18](#_Toc223538036)

[Offspring BW, Ms, M, PND40, Dupont (2010) 22](#_Toc223538037)

[Offspring BW gain, Ms, M, PND14-21, Dupont (2010) 25](#_Toc223538038)

[Offspring BW, Ms, F, PND4, DuPont (2010) 30](#_Toc223538039)

[Offspring BW, Ms, F, PND21, DuPont (2010) 33](#_Toc223538040)

[Offspring BW gain, Ms, F, PND14-21, DuPont (2010) 37](#_Toc223538041)

[Offspring BW gain, Ms, F, PND21-40, DuPont (2010) 40](#_Toc223538042)

[Liver single cell necrosis, Ms, F0, Dams, Premating-LD21, DuPont (2010) 44](#_Toc223538043)

[Liver focal necrosis, Ms, F0, Dams, Premating-LD21, DuPont (2010) 45](#_Toc223538044)

[Liver hepatocellular hypertrophy, Ms, F0, Dams, Premating-LD21, DuPont (2010) 48](#_Toc223538045)

[Abs kidney wt, Ms, F0, Dams, Premating-LD21, DuPont (2010) 52](#_Toc223538046)

[Rel kidney wt (brain), Ms, F0, Dams, Premating-LD21, DuPont (2010) 55](#_Toc223538047)

[Abs liver wt, Ms, F0, Dams, Premating-LD21, DuPont (2010) 59](#_Toc223538048)

[Rel liver wt (BW), Ms, F0, Dams, Premating-LD21, DuPont (2010) 63](#_Toc223538049)

[Rel liver wt (brain), Ms, F0, Dams, Premating-LD21, DuPont (2010)_drop 66](#_Toc223538050)

[premating BW change, Ms, F0, Dams, DuPont (2010) 70](#_Toc223538051)

[lactational BW, Ms, F0, Dams, LD21, DuPont (2010) 73](#_Toc223538052)

[Serum Triglyceride, Ms., M Offspring, Cope et al. (2021) 77](#_Toc223538053)

[Serum Triglyceride, Ms., F Offspring, Cope et al. (2021) 83](#_Toc223538054)

[Serum triglycerides, dams, E11.5, Ms, F, Blake et al. (2020) 88](#_Toc223538055)

[Serum triglycerides, dams, E17.5, Ms, F, Blake et al. (2020) 93](#_Toc223538056)

# Delayed Balanopreputial Separation, Ms, F1, M, Premating-LD21, Dupont (2010)

## Dataset

**Name:** Balanopreputial Separation, Ms, F1, M, Premating-LD21, Dupont(2010)

| Dose | N | Mean | Std. Dev. |
| --- | --- | --- | --- |
| 0 | 21 | 27.5 | 1.4 |
| 0.1 | 18 | 27.6 | 1.72 |
| 0.5 | 23 | 27.7 | 1.61 |
| 5 | 19 | 30.1 | 1.27 |

Test 1 Dose Response: <0.0001

Test 2 Homogeneity of Variance: 0.5499

Test 3 Variance Model Selection: 0.5499

## Settings

| Setting | Value |
| --- | --- |
| BMR | 5% Relative Deviation |
| Distribution | Normal + Constant variance |
| Adverse Direction | Up (↑) |
| Maximum Polynomial Degree | 3 |
| Confidence Level (one sided) | 0.95 |

## Maximum Likelihood Approach

| Model | BMDL | BMD | BMDU | *P*-Value | AIC | Scaled Residual at Control | Scaled Residual near BMD | Recommendation and Notes |
| --- | --- | --- | --- | --- | --- | --- | --- | --- |
| Exponential 3 | 2.164 | 2.931 | 4.913 | 0.878 | 300.544 | -0.085 | <0.001 | **Viable** |
| Exponential 5 | 0.52 | 2.905 | 4.964 | - | 302.544 | -0.087 | <0.001 | **Questionable** Zero degrees of freedom; saturated model BMD/BMDL ratio > 3.0 |
| Hill | 0.678 | 2.831 | 4.63 | - | 302.546 | -0.091 | <0.001 | **Questionable** Zero degrees of freedom; saturated model BMD/BMDL ratio > 3.0 |
| Polynomial 2 | 2.091 | 3.12 | 4.294 | 0.886 | 300.541 | -0.098 | 0.009 | **Viable** |
| Polynomial 3 | 2.091 | 3.413 | 4.527 | 0.881 | 300.543 | -0.114 | -0. | **Viable** |
| Power | 2.092 | 2.906 | 4.964 | 0.876 | 300.544 | -0.087 | <0.001 | **Viable** |
| Linear^ab^ | 2.089 | 2.642 | 3.576 | 0.972 | 298.576 | 0.021 | -0.174 | **Recommended - Lowest AIC** |

^a^ BMDS recommended best fitting model

^b^ User selected best fitting model


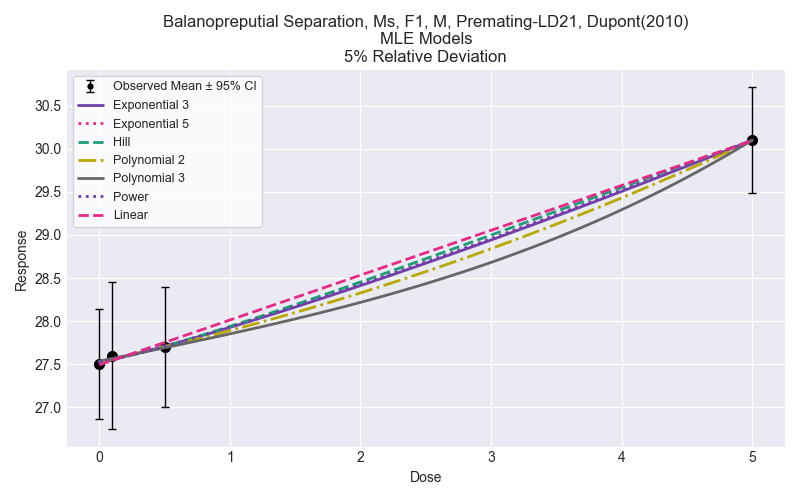


## Selected Model: Linear


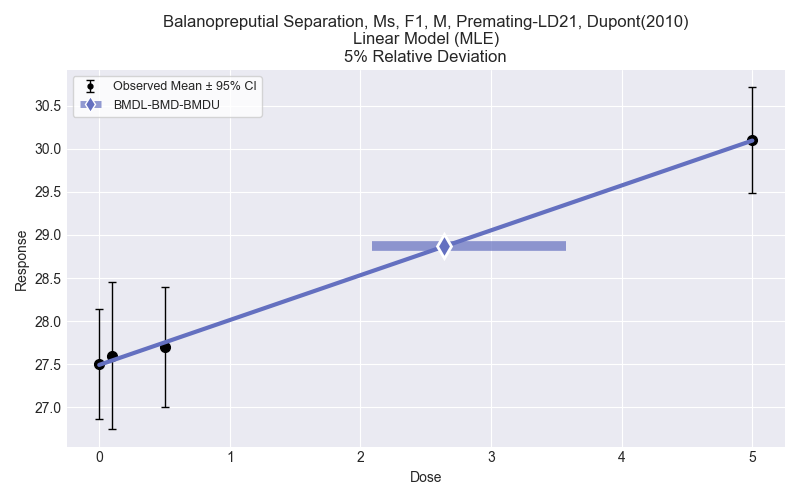


Linear Model
══════════════════════════════

Version: pybmds 25.1 (bmdscore 25.1)

Input Summary:
╒══════════════════════════════╤════════════════════════════╕
│ BMR │ 5% Relative Deviation │
│ Distribution │ Normal + Constant variance │
│ Modeling Direction │ Up (↑) │
│ Confidence Level (one sided) │ 0.95 │
│ Modeling Approach │ MLE │
│ Degree │ 1 │
╘══════════════════════════════╧════════════════════════════╛

Parameter Settings:
╒═════════════╤═══════════╤═════════╤════════╕
│ Parameter │ Initial │ Min │ Max │
╞═════════════╪═══════════╪═════════╪════════╡
│ g │ 0 │ -1e+06 │ 1e+06 │
│ b1 │ 0 │ -1e+06 │ 1e+06 │
│ alpha │ 0 │ -18 │ 18 │
╘═════════════╧═══════════╧═════════╧════════╛

Modeling Summary:
╒════════════════╤═════════════╕
│ BMD │ 2.64228 │
│ BMDL │ 2.0887 │
│ BMDU │ 3.5762 │
│ AIC │ 298.576 │
│ Log-Likelihood │ -146.288 │
│ P-Value │ 0.972489 │
│ Model d.f. │ 2 │
╘════════════════╧═════════════╛

Model Parameters:
╒════════════╤════════════╤════════════╤═════════════╕
│ Variable │ Estimate │ On Bound │ Std Error │
╞════════════╪════════════╪════════════╪═════════════╡
│ g │ 27.4933 │ no │ 0.195732 │
│ b1 │ 0.520257 │ no │ 0.0803303 │
│ alpha │ 2.16879 │ no │ 0.739142 │
╘════════════╧════════════╧════════════╧═════════════╛

Goodness of Fit:
╒════════╤═════╤═══════════════╤═════════════════════╤═══════════════════╕
│ Dose │ N │ Sample Mean │ Model Fitted Mean │ Scaled Residual │
╞════════╪═════╪═══════════════╪═════════════════════╪═══════════════════╡
│ 0 │ 21 │ 27.5 │ 27.4933 │ 0.0208911 │
│ 0.1 │ 18 │ 27.6 │ 27.5453 │ 0.15755 │
│ 0.5 │ 23 │ 27.7 │ 27.7534 │ -0.173947 │
│ 5 │ 19 │ 30.1 │ 30.0946 │ 0.0160713 │
╘════════╧═════╧═══════════════╧═════════════════════╧═══════════════════╛
╒════════╤═════╤═════════════╤═══════════════════╕
│ Dose │ N │ Sample SD │ Model Fitted SD │
╞════════╪═════╪═════════════╪═══════════════════╡
│ 0 │ 21 │ 1.4 │ 1.47268 │
│ 0.1 │ 18 │ 1.72 │ 1.47268 │
│ 0.5 │ 23 │ 1.61 │ 1.47268 │
│ 5 │ 19 │ 1.27 │ 1.47268 │
╘════════╧═════╧═════════════╧═══════════════════╛

Likelihoods:
╒═════════╤══════════════════╤════════════╤═════════╕
│ Model │ Log-Likelihood │ # Params │ AIC │
╞═════════╪══════════════════╪════════════╪═════════╡
│ A1 │ -146.26 │ 5 │ 302.52 │
│ A2 │ -145.205 │ 8 │ 306.41 │
│ A3 │ -146.26 │ 5 │ 302.52 │
│ fitted │ -146.288 │ 3 │ 298.576 │
│ reduced │ -163.189 │ 2 │ 330.379 │
╘═════════╧══════════════════╧════════════╧═════════╛

Tests of Mean and Variance Fits:
╒════════╤══════════════════════════════╤═════════════╤═════════════╕
│ Name │ -2 * Log(Likelihood Ratio) │ Test d.f. │ P-Value │
╞════════╪══════════════════════════════╪═════════════╪═════════════╡
│ Test 1 │ 35.9691 │ 6 │ 2.79505e-06 │
│ Test 2 │ 2.1102 │ 3 │ 0.549852 │
│ Test 3 │ 2.1102 │ 3 │ 0.549852 │
│ Test 4 │ 0.0557934 │ 2 │ 0.972489 │
╘════════╧══════════════════════════════╧═════════════╧═════════════╛
Test 1: Test the null hypothesis that responses and variances don't differ among dose levels
(A2 vs R). If this test fails to reject the null hypothesis (p-value > 0.05), there may not be
a dose-response.

Test 2: Test the null hypothesis that variances are homogenous (A1 vs A2). If this test fails to
reject the null hypothesis (p-value > 0.05), the simpler constant variance model may be appropriate.

Test 3: Test the null hypothesis that the variances are adequately modeled (A3 vs A2). If this test
fails to reject the null hypothesis (p-value > 0.05), it may be inferred that the variances have
been modeled appropriately.

Test 4: Test the null hypothesis that the model for the mean fits the data (Fitted vs A3). If this
test fails to reject the null hypothesis (p-value > 0.1), the user has support for use of the
selected model.

# Delayed vaginal latency, Ms, F1, F, Premating-LD21, Dupont (2010)

## Dataset

**Name:** Delayed vaginal latency, Ms, F1, F, Premating-LD21, Dupont(2010)

| Dose | N | Mean | Std. Dev. |
| --- | --- | --- | --- |
| 0 | 20 | 26.6 | 2.63 |
| 0.1 | 18 | 27.6 | 3.16 |
| 0.5 | 23 | 26 | 3.15 |
| 5 | 18 | 30 | 2.52 |

Test 1 Dose Response: 0.0014

Test 2 Homogeneity of Variance: 0.6471

Test 3 Variance Model Selection: 0.6471

## Settings

| Setting | Value |
| --- | --- |
| BMR | 5% Relative Deviation |
| Distribution | Normal + Constant variance |
| Adverse Direction | Up (↑) |
| Maximum Polynomial Degree | 3 |
| Confidence Level (one sided) | 0.95 |

## Maximum Likelihood Approach

| Model | BMDL | BMD | BMDU | *P*-Value | AIC | Scaled Residual at Control | Scaled Residual near BMD | Recommendation and Notes |
| --- | --- | --- | --- | --- | --- | --- | --- | --- |
| Exponential 3 | 1.578 | 4.489 | 4.92 | 0.073 | 399.101 | -0.107 | <0.001 | **Questionable** Goodness of fit p-value < 0.1 |
| Exponential 5 | 0.527 | 4.463 | 4.909 | - | 401.101 | -0.107 | <0.001 | **Questionable** Zero degrees of freedom; saturated model BMD/BMDL ratio > 3.0 |
| Hill | 0.632 | 4.487 | 4.771 | - | 401.101 | -0.107 | -0. | **Questionable** Zero degrees of freedom; saturated model BMD/BMDL ratio > 3.0 |
| Polynomial 2 | 1.49 | 3.162 | 4.058 | 0.189 | 397.222 | -0.091 | 0.013 | **Viable** |
| Polynomial 3^ab^ | 1.502 | 3.685 | 4.352 | 0.2 | 397.113 | -0.106 | 0.002 | **Recommended - Lowest AIC** |
| Power | 4.19 | 4.745 | 4.89 | 0.073 | 399.101 | -0.107 | <0.001 | **Questionable** Goodness of fit p-value < 0.1 |
| Linear | 1.393 | 1.978 | 3.339 | 0.113 | 398.259 | 0.077 | -1.466 | **Viable** |

^a^ BMDS recommended best fitting model

^b^ User selected best fitting model


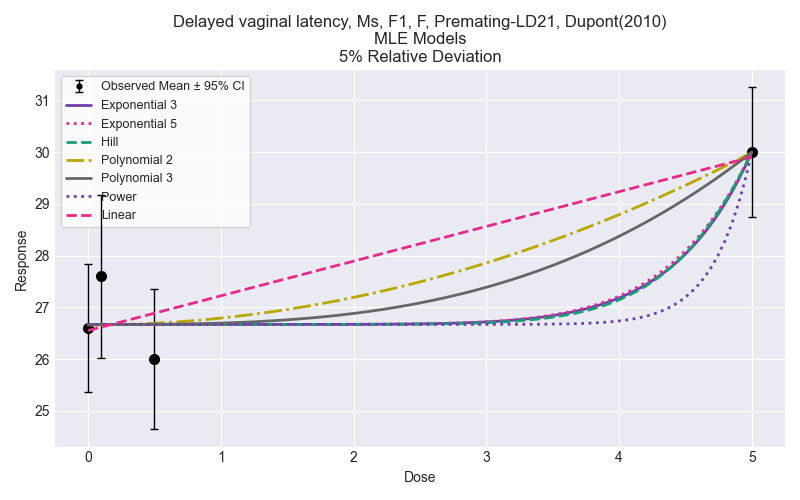


## Selected Model: Polynomial 3


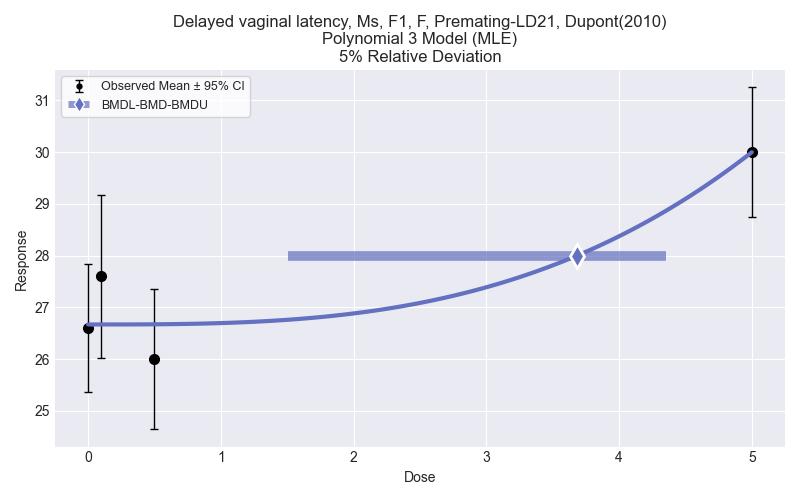


Polynomial 3 Model
══════════════════════════════

Version: pybmds 25.1 (bmdscore 25.1)

Input Summary:
╒══════════════════════════════╤════════════════════════════╕
│ BMR │ 5% Relative Deviation │
│ Distribution │ Normal + Constant variance │
│ Modeling Direction │ Up (↑) │
│ Confidence Level (one sided) │ 0.95 │
│ Modeling Approach │ MLE │
│ Degree │ 3 │
╘══════════════════════════════╧════════════════════════════╛

Parameter Settings:
╒═════════════╤═══════════╤═════════╤════════╕
│ Parameter │ Initial │ Min │ Max │
╞═════════════╪═══════════╪═════════╪════════╡
│ g │ 0 │ -1e+06 │ 1e+06 │
│ b1 │ 0 │ 0 │ 1e+06 │
│ b2 │ 0 │ 0 │ 1e+06 │
│ b3 │ 0 │ 0 │ 1e+06 │
│ alpha │ 0 │ -18 │ 18 │
╘═════════════╧═══════════╧═════════╧════════╛

Modeling Summary:
╒════════════════╤═════════════╕
│ BMD │ 3.68521 │
│ BMDL │ 1.50224 │
│ BMDU │ 4.3515 │
│ AIC │ 397.113 │
│ Log-Likelihood │ -195.557 │
│ P-Value │ 0.200001 │
│ Model d.f. │ 2 │
╘════════════════╧═════════════╛

Model Parameters:
╒════════════╤════════════╤════════════╤══════════════╕
│ Variable │ Estimate │ On Bound │ Std Error │
╞════════════╪════════════╪════════════╪══════════════╡
│ g │ 26.6681 │ no │ 0.368395 │
│ b1 │ 0 │ yes │ Not Reported │
│ b2 │ 0 │ yes │ Not Reported │
│ b3 │ 0.0266425 │ no │ 0.00617403 │
│ alpha │ 8.2725 │ no │ 10.8886 │
╘════════════╧════════════╧════════════╧══════════════╛
Standard errors estimates are not generated for parameters estimated on corresponding bounds,
although sampling error is present for all parameters, as a rule. Standard error estimates may not
be reliable as a basis for confidence intervals or tests when one or more parameters are on bounds.


Goodness of Fit:
╒════════╤═════╤═══════════════╤═════════════════════╤═══════════════════╕
│ Dose │ N │ Sample Mean │ Model Fitted Mean │ Scaled Residual │
╞════════╪═════╪═══════════════╪═════════════════════╪═══════════════════╡
│ 0 │ 20 │ 26.6 │ 26.6681 │ -0.105838 │
│ 0.1 │ 18 │ 27.6 │ 26.6681 │ 1.37464 │
│ 0.5 │ 23 │ 26 │ 26.6714 │ -1.11951 │
│ 5 │ 18 │ 30 │ 29.9984 │ 0.00239754 │
╘════════╧═════╧═══════════════╧═════════════════════╧═══════════════════╛
╒════════╤═════╤═════════════╤═══════════════════╕
│ Dose │ N │ Sample SD │ Model Fitted SD │
╞════════╪═════╪═════════════╪═══════════════════╡
│ 0 │ 20 │ 2.63 │ 2.87619 │
│ 0.1 │ 18 │ 3.16 │ 2.87619 │
│ 0.5 │ 23 │ 3.15 │ 2.87619 │
│ 5 │ 18 │ 2.52 │ 2.87619 │
╘════════╧═════╧═════════════╧═══════════════════╛

Likelihoods:
╒═════════╤══════════════════╤════════════╤═════════╕
│ Model │ Log-Likelihood │ # Params │ AIC │
╞═════════╪══════════════════╪════════════╪═════════╡
│ A1 │ -193.947 │ 5 │ 397.895 │
│ A2 │ -193.12 │ 8 │ 402.24 │
│ A3 │ -193.947 │ 5 │ 397.895 │
│ fitted │ -195.557 │ 3 │ 397.113 │
│ reduced │ -203.921 │ 2 │ 411.842 │
╘═════════╧══════════════════╧════════════╧═════════╛

Tests of Mean and Variance Fits:
╒════════╤══════════════════════════════╤═════════════╤════════════╕
│ Name │ -2 * Log(Likelihood Ratio) │ Test d.f. │ P-Value │
╞════════╪══════════════════════════════╪═════════════╪════════════╡
│ Test 1 │ 21.6017 │ 6 │ 0.00142942 │
│ Test 2 │ 1.6545 │ 3 │ 0.647097 │
│ Test 3 │ 1.6545 │ 3 │ 0.647097 │
│ Test 4 │ 3.21887 │ 2 │ 0.200001 │
╘════════╧══════════════════════════════╧═════════════╧════════════╛
Test 1: Test the null hypothesis that responses and variances don't differ among dose levels
(A2 vs R). If this test fails to reject the null hypothesis (p-value > 0.05), there may not be
a dose-response.

Test 2: Test the null hypothesis that variances are homogenous (A1 vs A2). If this test fails to
reject the null hypothesis (p-value > 0.05), the simpler constant variance model may be appropriate.

Test 3: Test the null hypothesis that the variances are adequately modeled (A3 vs A2). If this test
fails to reject the null hypothesis (p-value > 0.05), it may be inferred that the variances have
been modeled appropriately.

Test 4: Test the null hypothesis that the model for the mean fits the data (Fitted vs A3). If this
test fails to reject the null hypothesis (p-value > 0.1), the user has support for use of the
selected model.

# Serum HDL:LDL ratio, Ms, F1, F, Cope (2021)

## Dataset

**Name:** Serum HDL:LDL ratio, Ms, F1, F, Cope(2021)

| Dose | N | Mean | Std. Dev. |
| --- | --- | --- | --- |
| 0 | 8.5 | 3 | 0.3 |
| 0.2 | 8.5 | 3.4 | 0.3 |
| 1 | 8.5 | 3.2 | 0.2 |
| 2 | 8.5 | 3.5 | 0.4 |

Test 1 Dose Response: 0.0125

Test 2 Homogeneity of Variance: 0.2792

Test 3 Variance Model Selection: 0.2792

## Settings

| Setting | Value |
| --- | --- |
| BMR | 1.0 Standard Deviation |
| Distribution | Normal + Constant variance |
| Adverse Direction | Up (↑) |
| Maximum Polynomial Degree | 3 |
| Confidence Level (one sided) | 0.95 |

## Maximum Likelihood Approach

| Model | BMDL | BMD | BMDU | *P*-Value | AIC | Scaled Residual at Control | Scaled Residual near BMD | Recommendation and Notes |
| --- | --- | --- | --- | --- | --- | --- | --- | --- |
| Exponential 3 | 1.205 | 2.012 | 2.058 | 0.007 | 27.476 | -1.809 | <0.001 | **Questionable** Goodness of fit p-value < 0.1 BMD/highest dose ratio > 1.0 |
| Exponential 5 | 1.339 | 2.005 | 2.551 | 0.006 | 27.719 | -1.316 | 0.284 | **Questionable** Goodness of fit p-value < 0.1 BMD/highest dose ratio > 1.0 |
| Hill | 1.062 | 2.012 | 2.06 | - | 29.476 | -1.81 | <0.001 | **Questionable** Zero degrees of freedom; saturated model BMD/highest dose ratio > 1.0 |
| Polynomial 2 | 1.157 | 2.003 | 7.488 | 0.006 | 27.627 | -1.443 | 0.162 | **Questionable** Goodness of fit p-value < 0.1 BMD/highest dose ratio > 1.0 |
| Polynomial 3 | 1.176 | 2. | 6.777 | 0.007 | 27.431 | -1.538 | 0.074 | **Questionable** Goodness of fit p-value < 0.1 BMD/highest dose ratio > 1.0 |
| Power | 1.963 | 2.008 | 6.93 | 0.007 | 27.476 | -1.81 | -0. | **Questionable** Goodness of fit p-value < 0.1 BMD/highest dose ratio > 1.0 |
| Linear | 1.148 | 2.005 | 7.862 | 0.023 | 25.719 | -1.316 | 0.284 | **Questionable** Goodness of fit p-value < 0.1 BMD/highest dose ratio > 1.0 |


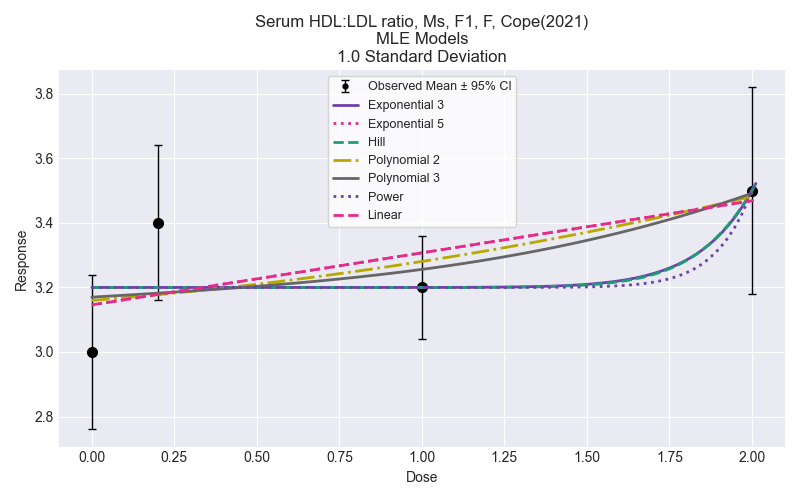


## Selected Model

No model was selected as a best-fitting model.

## Session for Serum HDL:LDL ratio, Ms, F1, F, Cope(2021)

## Dataset

**Name:** Serum HDL:LDL ratio, Ms, F1, F, Cope(2021)

| Dose | N | Mean | Std. Dev. |
| --- | --- | --- | --- |
| 0 | 8.5 | 3 | 0.3 |
| 0.2 | 8.5 | 3.4 | 0.3 |
| 1 | 8.5 | 3.2 | 0.2 |
| 2 | 8.5 | 3.5 | 0.4 |

## Settings

| Setting | Value |
| --- | --- |
| BMR | 1.0 Standard Deviation |
| Distribution | Normal + Nonconstant variance |
| Adverse Direction | Up (↑) |
| Maximum Polynomial Degree | 3 |
| Confidence Level (one sided) | 0.95 |

## Maximum Likelihood Approach

| Model | BMDL | BMD | BMDU | *P*-Value | AIC | Scaled Residual at Control | Scaled Residual near BMD | Recommendation and Notes |
| --- | --- | --- | --- | --- | --- | --- | --- | --- |
| Exponential 3 | - | 0.055 | - | -1 | - | <0.001 | <0.001 | **Unusable** Did not successfully execute. |
| Exponential 5 | 0.012 | 0.036 | - | - | 28.456 | <0.001 | <0.001 | **Questionable** lowest dose/BMDL ratio > 3.0 lowest dose/BMDL ratio > 10.0 lowest dose/BMD ratio > 3.0 Zero degrees of freedom; saturated model |
| Hill | 1.077 | 2.001 | - | 0.005 | 28.83 | -1.929 | -0. | **Questionable** Goodness of fit p-value < 0.1 BMD/highest dose ratio > 1.0 |
| Polynomial 2 | 1.077 | 1.986 | 7.456 | 0.004 | 29.506 | -1.585 | 0.132 | **Questionable** Goodness of fit p-value < 0.1 |
| Polynomial 3 | 1.108 | 1.976 | 2.017 | 0.004 | 29.225 | -1.641 | 0.015 | **Questionable** Goodness of fit p-value < 0.1 |
| Power | 1.158 | 2.001 | 5.302 | 0.021 | 26.831 | -1.924 | -0.007 | **Questionable** Goodness of fit p-value < 0.1 BMD/highest dose ratio > 1.0 |
| Linear | 1.061 | 1.958 | 8.159 | 0.014 | 27.661 | -1.359 | 0.28 | **Questionable** Goodness of fit p-value < 0.1 |

## Selected Model

No model was selected as a best-fitting model.

## Session for Serum HDL:LDL ratio, Ms, F1, F, Cope(2021)

## Dataset

**Name:** Serum HDL:LDL ratio, Ms, F1, F, Cope(2021)

| Dose | N | Mean | Std. Dev. |
| --- | --- | --- | --- |
| 0 | 8.5 | 3 | 0.3 |
| 0.2 | 8.5 | 3.4 | 0.3 |
| 1 | 8.5 | 3.2 | 0.2 |
| 2 | 8.5 | 3.5 | 0.4 |

Test 1 Dose Response: 0.0153

Test 2 Homogeneity of Variance: 0.3847

Test 3 Variance Model Selection: 0.3847

## Settings

| Setting | Value |
| --- | --- |
| BMR | 1.0 Standard Deviation |
| Distribution | Lognormal + Constant variance |
| Adverse Direction | Up (↑) |
| Maximum Polynomial Degree | 3 |
| Confidence Level (one sided) | 0.95 |

## Maximum Likelihood Approach

| Model | BMDL | BMD | BMDU | *P*-Value | AIC | Scaled Residual at Control | Scaled Residual near BMD | Recommendation and Notes |
| --- | --- | --- | --- | --- | --- | --- | --- | --- |
| Exponential 3 | 1.144 | 2.043 | 2.242 | 0.005 | 26.855 | -0.474 | 0.059 | **Questionable** Goodness of fit p-value < 0.1 BMD/highest dose ratio > 1.0 |
| Exponential 5 | 1.101 | 2.045 | 4.43 | - | 28.855 | -0.474 | 0.059 | **Questionable** Zero degrees of freedom; saturated model BMD/highest dose ratio > 1.0 |


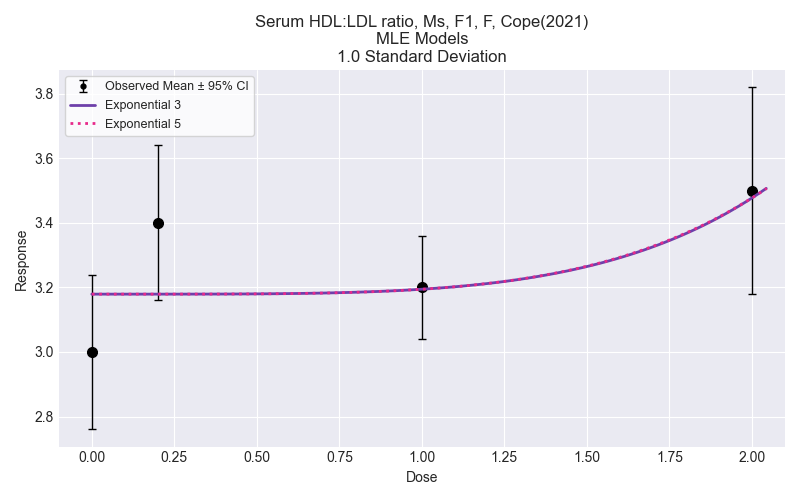


## Selected Model

No model was selected as a best-fitting model.

# Offspring BW, Ms, M, PND4, Dupont (2010)

## Dataset

**Name:** Offspring BW, Ms, M, PND4, Dupont (2010)

| Dose | N | Mean | Std. Dev. |
| --- | --- | --- | --- |
| 0 | 22 | 3.17 | 0.39 |
| 0.1 | 21 | 3.11 | 0.43 |
| 0.5 | 24 | 3.3 | 0.421 |
| 5 | 21 | 2.57 | 0.359 |

Test 1 Dose Response: <0.0001

Test 2 Homogeneity of Variance: 0.8361

Test 3 Variance Model Selection: 0.8361

## Settings

| Setting | Value |
| --- | --- |
| BMR | 5% Relative Deviation |
| Distribution | Normal + Constant variance |
| Adverse Direction | Down (↓) |
| Maximum Polynomial Degree | 3 |
| Confidence Level (one sided) | 0.95 |

## Maximum Likelihood Approach

| Model | BMDL | BMD | BMDU | *P*-Value | AIC | Scaled Residual at Control | Scaled Residual near BMD | Recommendation and Notes |
| --- | --- | --- | --- | --- | --- | --- | --- | --- |
| Exponential 3 | 1.046 | 4.261 | 4.701 | 0.097 | 95.813 | -0.327 | <0.001 | **Questionable** Goodness of fit p-value < 0.1 BMD/BMDL ratio > 3.0 |
| Exponential 5 | 1.046 | 4.275 | 4.712 | - | 97.813 | -0.327 | -0. | **Questionable** Zero degrees of freedom; saturated model BMD/BMDL ratio > 3.0 |
| Hill | 4.525 | 4.602 | 4.686 | - | 97.813 | -0.327 | <0.001 | **Questionable** Zero degrees of freedom; saturated model |
| Polynomial 2 | 1.105 | 2.523 | 2.923 | 0.23 | 94.005 | -0.349 | 1.309 | **Viable** |
| Polynomial 3^ab^ | 1.119 | 3.17 | 3.495 | 0.251 | 93.832 | -0.329 | -0.002 | **Recommended - Lowest AIC** |
| Power | 1.121 | 4.632 | 4.739 | 0.097 | 95.813 | -0.327 | -0. | **Questionable** Goodness of fit p-value < 0.1 BMD/BMDL ratio > 3.0 |
| Linear | 1.008 | 1.267 | 1.719 | 0.093 | 95.824 | -0.582 | 1.745 | **Questionable** Goodness of fit p-value < 0.1 |

^a^ BMDS recommended best fitting model

^b^ User selected best fitting model


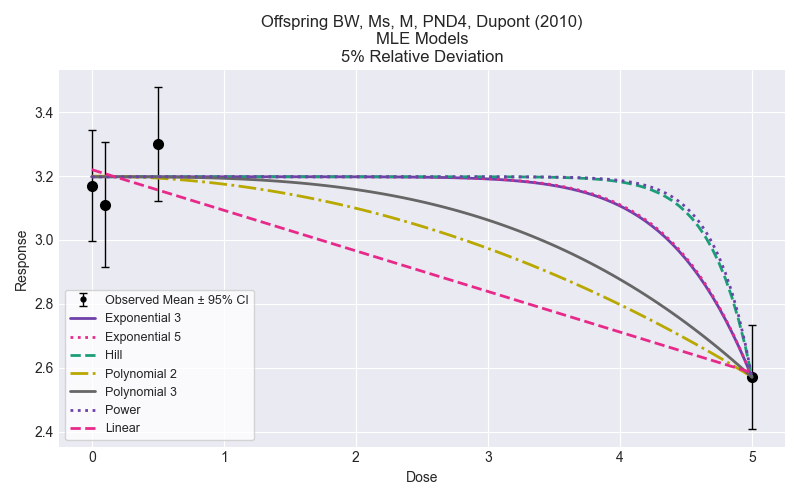


## Selected Model: Polynomial 3


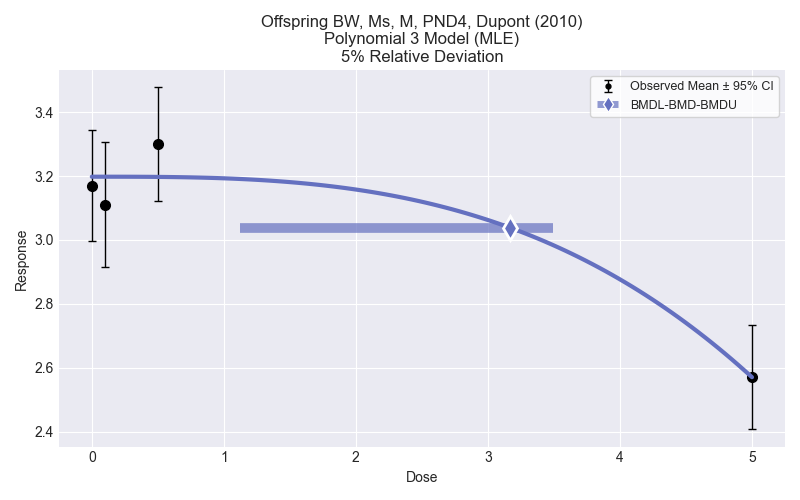


Polynomial 3 Model
══════════════════════════════

Version: pybmds 25.1 (bmdscore 25.1)

Input Summary:
╒══════════════════════════════╤════════════════════════════╕
│ BMR │ 5% Relative Deviation │
│ Distribution │ Normal + Constant variance │
│ Modeling Direction │ Down (↓) │
│ Confidence Level (one sided) │ 0.95 │
│ Modeling Approach │ MLE │
│ Degree │ 3 │
╘══════════════════════════════╧════════════════════════════╛

Parameter Settings:
╒═════════════╤═══════════╤═════════╤════════╕
│ Parameter │ Initial │ Min │ Max │
╞═════════════╪═══════════╪═════════╪════════╡
│ g │ 0 │ -1e+06 │ 1e+06 │
│ b1 │ 0 │ -1e+06 │ 0 │
│ b2 │ 0 │ -1e+06 │ 0 │
│ b3 │ 0 │ -1e+06 │ 0 │
│ alpha │ 0 │ -18 │ 18 │
╘═════════════╧═══════════╧═════════╧════════╛

Modeling Summary:
╒════════════════╤════════════╕
│ BMD │ 3.16953 │
│ BMDL │ 1.11924 │
│ BMDU │ 3.49497 │
│ AIC │ 93.8318 │
│ Log-Likelihood │ -43.9159 │
│ P-Value │ 0.250756 │
│ Model d.f. │ 2 │
╘════════════════╧════════════╛

Model Parameters:
╒════════════╤═════════════╤════════════╤══════════════╕
│ Variable │ Estimate │ On Bound │ Std Error │
╞════════════╪═════════════╪════════════╪══════════════╡
│ g │ 3.19794 │ no │ 0.0487114 │
│ b1 │ 0 │ yes │ Not Reported │
│ b2 │ 0 │ yes │ Not Reported │
│ b3 │ -0.00502178 │ no │ 0.000797727 │
│ alpha │ 0.158863 │ no │ 0.00380482 │
╘════════════╧═════════════╧════════════╧══════════════╛
Standard errors estimates are not generated for parameters estimated on corresponding bounds,
although sampling error is present for all parameters, as a rule. Standard error estimates may not
be reliable as a basis for confidence intervals or tests when one or more parameters are on bounds.


Goodness of Fit:
╒════════╤═════╤═══════════════╤═════════════════════╤═══════════════════╕
│ Dose │ N │ Sample Mean │ Model Fitted Mean │ Scaled Residual │
╞════════╪═════╪═══════════════╪═════════════════════╪═══════════════════╡
│ 0 │ 22 │ 3.17 │ 3.19794 │ -0.328773 │
│ 0.1 │ 21 │ 3.11 │ 3.19793 │ -1.011 │
│ 0.5 │ 24 │ 3.3 │ 3.19731 │ 1.26218 │
│ 5 │ 21 │ 2.57 │ 2.57022 │ -0.00248353 │
╘════════╧═════╧═══════════════╧═════════════════════╧═══════════════════╛
╒════════╤═════╤═════════════╤═══════════════════╕
│ Dose │ N │ Sample SD │ Model Fitted SD │
╞════════╪═════╪═════════════╪═══════════════════╡
│ 0 │ 22 │ 0.39 │ 0.398576 │
│ 0.1 │ 21 │ 0.43 │ 0.398576 │
│ 0.5 │ 24 │ 0.421 │ 0.398576 │
│ 5 │ 21 │ 0.359 │ 0.398576 │
╘════════╧═════╧═════════════╧═══════════════════╛

Likelihoods:
╒═════════╤══════════════════╤════════════╤══════════╕
│ Model │ Log-Likelihood │ # Params │ AIC │
╞═════════╪══════════════════╪════════════╪══════════╡
│ A1 │ -42.5326 │ 5 │ 95.0653 │
│ A2 │ -42.1048 │ 8 │ 100.21 │
│ A3 │ -42.5326 │ 5 │ 95.0653 │
│ fitted │ -43.9159 │ 3 │ 93.8318 │
│ reduced │ -60.2805 │ 2 │ 124.561 │
╘═════════╧══════════════════╧════════════╧══════════╛

Tests of Mean and Variance Fits:
╒════════╤══════════════════════════════╤═════════════╤═════════════╕
│ Name │ -2 * Log(Likelihood Ratio) │ Test d.f. │ P-Value │
╞════════╪══════════════════════════════╪═════════════╪═════════════╡
│ Test 1 │ 36.3515 │ 6 │ 2.35514e-06 │
│ Test 2 │ 0.855739 │ 3 │ 0.836094 │
│ Test 3 │ 0.855739 │ 3 │ 0.836094 │
│ Test 4 │ 2.76655 │ 2 │ 0.250756 │
╘════════╧══════════════════════════════╧═════════════╧═════════════╛
Test 1: Test the null hypothesis that responses and variances don't differ among dose levels
(A2 vs R). If this test fails to reject the null hypothesis (p-value > 0.05), there may not be
a dose-response.

Test 2: Test the null hypothesis that variances are homogenous (A1 vs A2). If this test fails to
reject the null hypothesis (p-value > 0.05), the simpler constant variance model may be appropriate.

Test 3: Test the null hypothesis that the variances are adequately modeled (A3 vs A2). If this test
fails to reject the null hypothesis (p-value > 0.05), it may be inferred that the variances have
been modeled appropriately.

Test 4: Test the null hypothesis that the model for the mean fits the data (Fitted vs A3). If this
test fails to reject the null hypothesis (p-value > 0.1), the user has support for use of the
selected model.

# Offspring BW, Ms, M, PND21, Dupont (2010)

## Dataset

**Name:** Offspring BW, Ms, M, PND21, Dupont (2010)

| Dose | N | Mean | Std. Dev. |
| --- | --- | --- | --- |
| 0 | 21 | 13.52 | 1.376 |
| 0.1 | 18 | 13.78 | 1.37 |
| 0.5 | 23 | 13.87 | 1.284 |
| 5 | 20 | 10.56 | 1.908 |

Test 1 Dose Response: <0.0001

Test 2 Homogeneity of Variance: 0.2325

Test 3 Variance Model Selection: 0.2325

## Settings

| Setting | Value |
| --- | --- |
| BMR | 5% Relative Deviation |
| Distribution | Normal + Constant variance |
| Adverse Direction | Down (↓) |
| Maximum Polynomial Degree | 3 |
| Confidence Level (one sided) | 0.95 |

## Maximum Likelihood Approach

| Model | BMDL | BMD | BMDU | *P*-Value | AIC | Scaled Residual at Control | Scaled Residual near BMD | Recommendation and Notes |
| --- | --- | --- | --- | --- | --- | --- | --- | --- |
| Exponential 3^a^ | 0.861 | 4.138 | 4.671 | 0.416 | 303.63 | -0.641 | <0.001 | **Recommended - Lowest BMDL** BMD/BMDL ratio > 3.0 |
| Exponential 5 | 0.861 | 4.142 | 4.675 | - | 305.63 | -0.641 | -0. | **Questionable** Zero degrees of freedom; saturated model BMD/BMDL ratio > 3.0 |
| Hill | 3.521 | 3.858 | 4.107 | - | 305.63 | -0.641 | -0. | **Questionable** Zero degrees of freedom; saturated model |
| Polynomial 2^b^ | 0.949 | 2.325 | 2.586 | 0.681 | 301.736 | -0.678 | 0.537 | **Viable** |
| Polynomial 3 | 0.953 | 3.003 | 3.225 | 0.715 | 301.64 | -0.647 | 0.006 | **Viable** BMD/BMDL ratio > 3.0 |
| Power | 3.948 | 4.592 | 4.697 | 0.416 | 303.63 | -0.641 | -0. | **Viable** |
| Linear | 0.896 | 1.065 | 1.319 | 0.311 | 303.301 | -1.027 | 1.11 | **Viable** |

^a^ BMDS recommended best fitting model

^b^ lower AIC; no warnings; similar BMDL


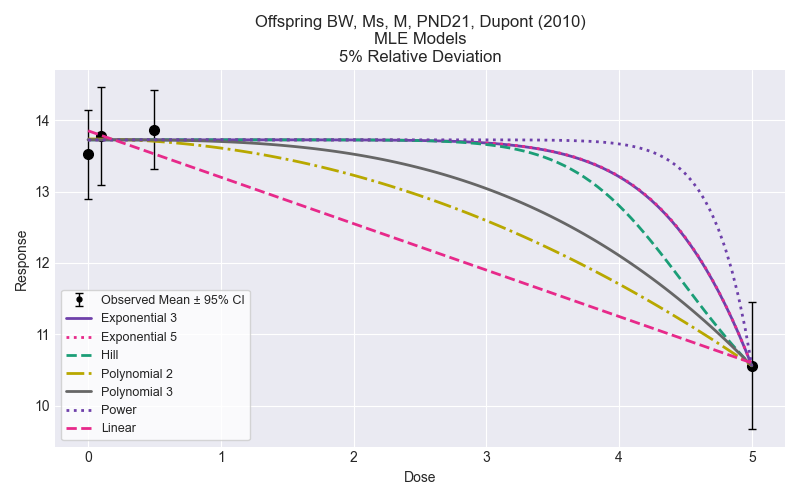


## Selected Model: Polynomial 2


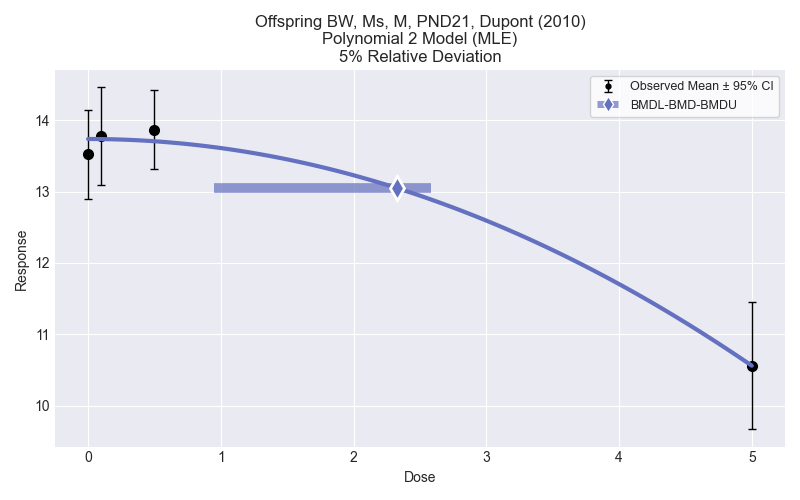


Polynomial 2 Model
══════════════════════════════

Version: pybmds 25.1 (bmdscore 25.1)

Input Summary:
╒══════════════════════════════╤════════════════════════════╕
│ BMR │ 5% Relative Deviation │
│ Distribution │ Normal + Constant variance │
│ Modeling Direction │ Down (↓) │
│ Confidence Level (one sided) │ 0.95 │
│ Modeling Approach │ MLE │
│ Degree │ 2 │
╘══════════════════════════════╧════════════════════════════╛

Parameter Settings:
╒═════════════╤═══════════╤═════════╤════════╕
│ Parameter │ Initial │ Min │ Max │
╞═════════════╪═══════════╪═════════╪════════╡
│ g │ 0 │ -1e+06 │ 1e+06 │
│ b1 │ 0 │ -1e+06 │ 0 │
│ b2 │ 0 │ -1e+06 │ 0 │
│ alpha │ 0 │ -18 │ 18 │
╘═════════════╧═══════════╧═════════╧════════╛

Modeling Summary:
╒════════════════╤═════════════╕
│ BMD │ 2.32499 │
│ BMDL │ 0.948656 │
│ BMDU │ 2.58597 │
│ AIC │ 301.736 │
│ Log-Likelihood │ -147.868 │
│ P-Value │ 0.681162 │
│ Model d.f. │ 2 │
╘════════════════╧═════════════╛

Model Parameters:
╒════════════╤════════════╤════════════╤══════════════╕
│ Variable │ Estimate │ On Bound │ Std Error │
╞════════════╪════════════╪════════════╪══════════════╡
│ g │ 13.7373 │ no │ 0.187235 │
│ b1 │ 0 │ yes │ Not Reported │
│ b2 │ -0.127065 │ no │ 0.015164 │
│ alpha │ 2.15689 │ no │ 0.726566 │
╘════════════╧════════════╧════════════╧══════════════╛
Standard errors estimates are not generated for parameters estimated on corresponding bounds,
although sampling error is present for all parameters, as a rule. Standard error estimates may not
be reliable as a basis for confidence intervals or tests when one or more parameters are on bounds.


Goodness of Fit:
╒════════╤═════╤═══════════════╤═════════════════════╤═══════════════════╕
│ Dose │ N │ Sample Mean │ Model Fitted Mean │ Scaled Residual │
╞════════╪═════╪═══════════════╪═════════════════════╪═══════════════════╡
│ 0 │ 21 │ 13.52 │ 13.7373 │ -0.677946 │
│ 0.1 │ 18 │ 13.78 │ 13.736 │ 0.127111 │
│ 0.5 │ 23 │ 13.87 │ 13.7055 │ 0.537164 │
│ 5 │ 20 │ 10.56 │ 10.5606 │ -0.00195115 │
╘════════╧═════╧═══════════════╧═════════════════════╧═══════════════════╛
╒════════╤═════╤═════════════╤═══════════════════╕
│ Dose │ N │ Sample SD │ Model Fitted SD │
╞════════╪═════╪═════════════╪═══════════════════╡
│ 0 │ 21 │ 1.376 │ 1.46864 │
│ 0.1 │ 18 │ 1.37 │ 1.46864 │
│ 0.5 │ 23 │ 1.284 │ 1.46864 │
│ 5 │ 20 │ 1.908 │ 1.46864 │
╘════════╧═════╧═════════════╧═══════════════════╛

Likelihoods:
╒═════════╤══════════════════╤════════════╤═════════╕
│ Model │ Log-Likelihood │ # Params │ AIC │
╞═════════╪══════════════════╪════════════╪═════════╡
│ A1 │ -147.484 │ 5 │ 304.968 │
│ A2 │ -145.342 │ 8 │ 306.684 │
│ A3 │ -147.484 │ 5 │ 304.968 │
│ fitted │ -147.868 │ 3 │ 301.736 │
│ reduced │ -173.209 │ 2 │ 350.418 │
╘═════════╧══════════════════╧════════════╧═════════╛

Tests of Mean and Variance Fits:
╒════════╤══════════════════════════════╤═════════════╤═════════════╕
│ Name │ -2 * Log(Likelihood Ratio) │ Test d.f. │ P-Value │
╞════════╪══════════════════════════════╪═════════════╪═════════════╡
│ Test 1 │ 55.7341 │ 6 │ 3.29448e-10 │
│ Test 2 │ 4.28333 │ 3 │ 0.23245 │
│ Test 3 │ 4.28333 │ 3 │ 0.23245 │
│ Test 4 │ 0.767911 │ 2 │ 0.681162 │
╘════════╧══════════════════════════════╧═════════════╧═════════════╛
Test 1: Test the null hypothesis that responses and variances don't differ among dose levels
(A2 vs R). If this test fails to reject the null hypothesis (p-value > 0.05), there may not be
a dose-response.

Test 2: Test the null hypothesis that variances are homogenous (A1 vs A2). If this test fails to
reject the null hypothesis (p-value > 0.05), the simpler constant variance model may be appropriate.

Test 3: Test the null hypothesis that the variances are adequately modeled (A3 vs A2). If this test
fails to reject the null hypothesis (p-value > 0.05), it may be inferred that the variances have
been modeled appropriately.

Test 4: Test the null hypothesis that the model for the mean fits the data (Fitted vs A3). If this
test fails to reject the null hypothesis (p-value > 0.1), the user has support for use of the
selected model.

# Offspring BW, Ms, M, PND40, Dupont (2010)

## Dataset

**Name:** Offspring BW, Ms, M, PND40, Dupont (2010)

| Dose | N | Mean | Std. Dev. |
| --- | --- | --- | --- |
| 0 | 21 | 29.6 | 2.77 |
| 0.1 | 18 | 29.8 | 2.07 |
| 0.5 | 23 | 30 | 2.86 |
| 5 | 19 | 27.2 | 2.3 |

Test 1 Dose Response: 0.0073

Test 2 Homogeneity of Variance: 0.4231

Test 3 Variance Model Selection: 0.4231

## Settings

| Setting | Value |
| --- | --- |
| BMR | 5% Relative Deviation |
| Distribution | Normal + Constant variance |
| Adverse Direction | Down (↓) |
| Maximum Polynomial Degree | 3 |
| Confidence Level (one sided) | 0.95 |

## Maximum Likelihood Approach

| Model | BMDL | BMD | BMDU | *P*-Value | AIC | Scaled Residual at Control | Scaled Residual near BMD | Recommendation and Notes |
| --- | --- | --- | --- | --- | --- | --- | --- | --- |
| Exponential 3 | 1.976 | 4.658 | 5.011 | 0.595 | 385.822 | -0.38 | 0 | **Viable** |
| Exponential 5 | 1.975 | 4.61 | 5.04 | - | 387.822 | -0.38 | -0. | **Questionable** Zero degrees of freedom; saturated model |
| Hill | 0.571 | 4.162 | 4.988 | - | 387.822 | -0.38 | 0 | **Questionable** Zero degrees of freedom; saturated model BMD/BMDL ratio > 3.0 |
| Polynomial 2 | 2.044 | 3.759 | 4.927 | 0.564 | 385.872 | -0.4 | -0.011 | **Viable** |
| Polynomial 3^ab^ | 2.05 | 4.144 | 4.954 | 0.866 | 383.827 | -0.386 | 0.015 | **Recommended - Lowest AIC** |
| Power | 2.05 | 4.845 | 5.02 | 0.595 | 385.822 | -0.38 | -0. | **Viable** |
| Linear | 1.988 | 2.805 | 4.825 | 0.667 | 384.349 | -0.564 | -0.074 | **Viable** |

^a^ BMDS recommended best fitting model

^b^ User selected best fitting model


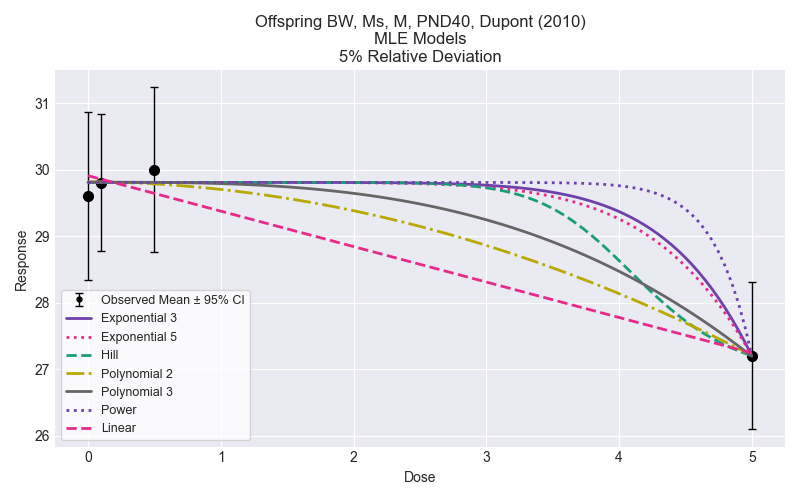


## Selected Model: Polynomial 3


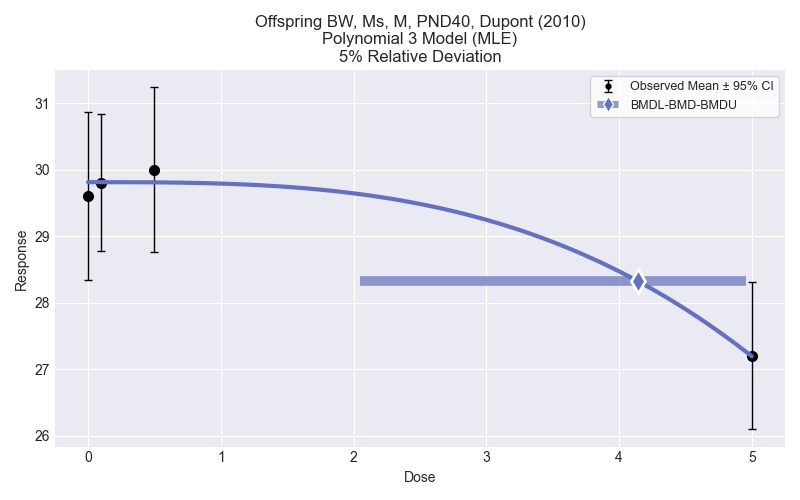


Polynomial 3 Model
══════════════════════════════

Version: pybmds 25.1 (bmdscore 25.1)

Input Summary:
╒══════════════════════════════╤════════════════════════════╕
│ BMR │ 5% Relative Deviation │
│ Distribution │ Normal + Constant variance │
│ Modeling Direction │ Down (↓) │
│ Confidence Level (one sided) │ 0.95 │
│ Modeling Approach │ MLE │
│ Degree │ 3 │
╘══════════════════════════════╧════════════════════════════╛

Parameter Settings:
╒═════════════╤═══════════╤═════════╤════════╕
│ Parameter │ Initial │ Min │ Max │
╞═════════════╪═══════════╪═════════╪════════╡
│ g │ 0 │ -1e+06 │ 1e+06 │
│ b1 │ 0 │ -1e+06 │ 0 │
│ b2 │ 0 │ -1e+06 │ 0 │
│ b3 │ 0 │ -1e+06 │ 0 │
│ alpha │ 0 │ -18 │ 18 │
╘═════════════╧═══════════╧═════════╧════════╛

Modeling Summary:
╒════════════════╤═════════════╕
│ BMD │ 4.14378 │
│ BMDL │ 2.04957 │
│ BMDU │ 4.95367 │
│ AIC │ 383.827 │
│ Log-Likelihood │ -188.913 │
│ P-Value │ 0.866135 │
│ Model d.f. │ 2 │
╘════════════════╧═════════════╛

Model Parameters:
╒════════════╤════════════╤════════════╤══════════════╕
│ Variable │ Estimate │ On Bound │ Std Error │
╞════════════╪════════════╪════════════╪══════════════╡
│ g │ 29.81 │ no │ 0.316736 │
│ b1 │ 0 │ yes │ Not Reported │
│ b2 │ 0 │ yes │ Not Reported │
│ b3 │ -0.020948 │ no │ 0.00523196 │
│ alpha │ 6.21517 │ no │ 6.07095 │
╘════════════╧════════════╧════════════╧══════════════╛
Standard errors estimates are not generated for parameters estimated on corresponding bounds,
although sampling error is present for all parameters, as a rule. Standard error estimates may not
be reliable as a basis for confidence intervals or tests when one or more parameters are on bounds.


Goodness of Fit:
╒════════╤═════╤═══════════════╤═════════════════════╤═══════════════════╕
│ Dose │ N │ Sample Mean │ Model Fitted Mean │ Scaled Residual │
╞════════╪═════╪═══════════════╪═════════════════════╪═══════════════════╡
│ 0 │ 21 │ 29.6 │ 29.81 │ -0.386078 │
│ 0.1 │ 18 │ 29.8 │ 29.81 │ -0.0170424 │
│ 0.5 │ 23 │ 30 │ 29.8074 │ 0.370472 │
│ 5 │ 19 │ 27.2 │ 27.1915 │ 0.0147894 │
╘════════╧═════╧═══════════════╧═════════════════════╧═══════════════════╛
╒════════╤═════╤═════════════╤═══════════════════╕
│ Dose │ N │ Sample SD │ Model Fitted SD │
╞════════╪═════╪═════════════╪═══════════════════╡
│ 0 │ 21 │ 2.77 │ 2.49302 │
│ 0.1 │ 18 │ 2.07 │ 2.49302 │
│ 0.5 │ 23 │ 2.86 │ 2.49302 │
│ 5 │ 19 │ 2.3 │ 2.49302 │
╘════════╧═════╧═════════════╧═══════════════════╛

Likelihoods:
╒═════════╤══════════════════╤════════════╤═════════╕
│ Model │ Log-Likelihood │ # Params │ AIC │
╞═════════╪══════════════════╪════════════╪═════════╡
│ A1 │ -188.77 │ 5 │ 387.539 │
│ A2 │ -187.368 │ 8 │ 390.737 │
│ A3 │ -188.77 │ 5 │ 387.539 │
│ fitted │ -188.913 │ 3 │ 383.827 │
│ reduced │ -196.171 │ 2 │ 396.343 │
╘═════════╧══════════════════╧════════════╧═════════╛

Tests of Mean and Variance Fits:
╒════════╤══════════════════════════════╤═════════════╤════════════╕
│ Name │ -2 * Log(Likelihood Ratio) │ Test d.f. │ P-Value │
╞════════╪══════════════════════════════╪═════════════╪════════════╡
│ Test 1 │ 17.606 │ 6 │ 0.00729604 │
│ Test 2 │ 2.8025 │ 3 │ 0.423088 │
│ Test 3 │ 2.8025 │ 3 │ 0.423088 │
│ Test 4 │ 0.287429 │ 2 │ 0.866135 │
╘════════╧══════════════════════════════╧═════════════╧════════════╛
Test 1: Test the null hypothesis that responses and variances don't differ among dose levels
(A2 vs R). If this test fails to reject the null hypothesis (p-value > 0.05), there may not be
a dose-response.

Test 2: Test the null hypothesis that variances are homogenous (A1 vs A2). If this test fails to
reject the null hypothesis (p-value > 0.05), the simpler constant variance model may be appropriate.

Test 3: Test the null hypothesis that the variances are adequately modeled (A3 vs A2). If this test
fails to reject the null hypothesis (p-value > 0.05), it may be inferred that the variances have
been modeled appropriately.

Test 4: Test the null hypothesis that the model for the mean fits the data (Fitted vs A3). If this
test fails to reject the null hypothesis (p-value > 0.1), the user has support for use of the
selected model.

# Offspring BW gain, Ms, M, PND14-21, Dupont (2010)

## Dataset

**Name:** Offspring BW gain, Ms, M, PND14-21, Dupont (2010)

| Dose | N | Mean | Std. Dev. |
| --- | --- | --- | --- |
| 0 | 21 | 4.87 | 1.027 |
| 0.1 | 18 | 4.92 | 0.839 |
| 0.5 | 23 | 5.21 | 0.807 |
| 5 | 20 | 3.52 | 0.982 |

Test 1 Dose Response: <0.0001

Test 2 Homogeneity of Variance: 0.6295

Test 3 Variance Model Selection: 0.6295

## Settings

| Setting | Value |
| --- | --- |
| BMR | 5% Relative Deviation |
| Distribution | Normal + Constant variance |
| Adverse Direction | Down (↓) |
| Maximum Polynomial Degree | 3 |
| Confidence Level (one sided) | 0.95 |

## Maximum Likelihood Approach

| Model | BMDL | BMD | BMDU | *P*-Value | AIC | Scaled Residual at Control | Scaled Residual near BMD | Recommendation and Notes |
| --- | --- | --- | --- | --- | --- | --- | --- | --- |
| Exponential 3 | 0.654 | 4.109 | 4.617 | 0.177 | 224.37 | -0.712 | -0. | **Viable** BMD/BMDL ratio > 3.0 |
| Exponential 5 | 0.654 | 4.107 | 4.617 | - | 226.37 | -0.712 | -0. | **Questionable** Zero degrees of freedom; saturated model BMD/BMDL ratio > 3.0 |
| Hill | 0.518 | 3.06 | 4.322 | - | 226.37 | -0.712 | -0. | **Questionable** Zero degrees of freedom; saturated model BMD/BMDL ratio > 3.0 |
| Polynomial 2 | 0.731 | 2.049 | 2.363 | 0.369 | 222.538 | -0.736 | 1.108 | **Viable** |
| Polynomial 3^ab^ | 0.739 | 2.759 | 3.035 | 0.399 | 222.386 | -0.714 | -0.001 | **Recommended - Lowest AIC** BMD/BMDL ratio > 3.0 |
| Power | 0.74 | 4.526 | 4.603 | 0.177 | 224.37 | -0.712 | 0 | **Viable** BMD/BMDL ratio > 3.0 |
| Linear | 0.672 | 0.837 | 1.12 | 0.153 | 224.297 | -0.977 | 1.55 | **Viable** |

^a^ BMDS recommended best fitting model

^b^ User selected best fitting model


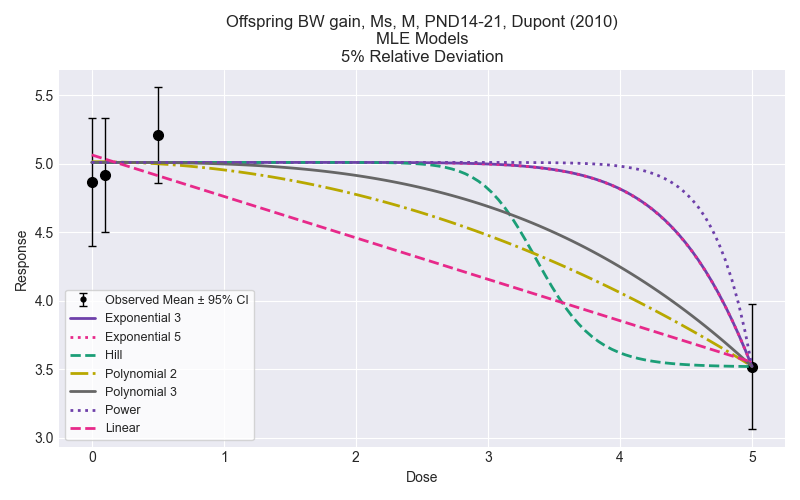


## Selected Model: Polynomial 3


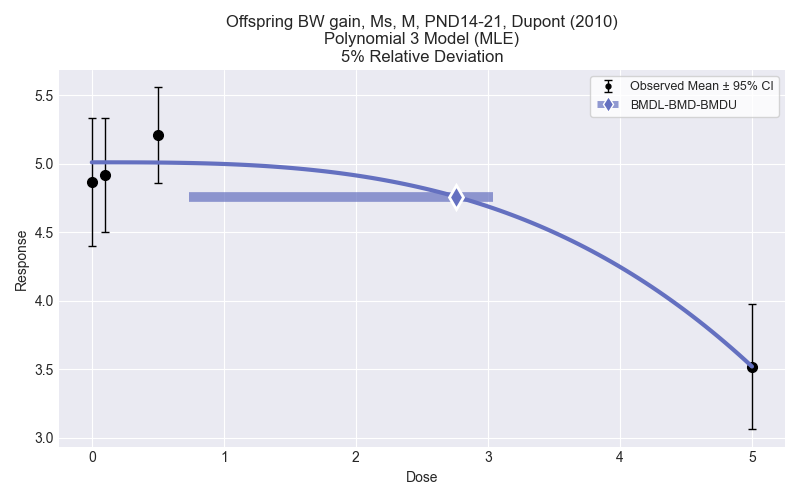


Polynomial 3 Model
══════════════════════════════

Version: pybmds 25.1 (bmdscore 25.1)

Input Summary:
╒══════════════════════════════╤════════════════════════════╕
│ BMR │ 5% Relative Deviation │
│ Distribution │ Normal + Constant variance │
│ Modeling Direction │ Down (↓) │
│ Confidence Level (one sided) │ 0.95 │
│ Modeling Approach │ MLE │
│ Degree │ 3 │
╘══════════════════════════════╧════════════════════════════╛

Parameter Settings:
╒═════════════╤═══════════╤═════════╤════════╕
│ Parameter │ Initial │ Min │ Max │
╞═════════════╪═══════════╪═════════╪════════╡
│ g │ 0 │ -1e+06 │ 1e+06 │
│ b1 │ 0 │ -1e+06 │ 0 │
│ b2 │ 0 │ -1e+06 │ 0 │
│ b3 │ 0 │ -1e+06 │ 0 │
│ alpha │ 0 │ -18 │ 18 │
╘═════════════╧═══════════╧═════════╧════════╛

Modeling Summary:
╒════════════════╤═════════════╕
│ BMD │ 2.75925 │
│ BMDL │ 0.738743 │
│ BMDU │ 3.03515 │
│ AIC │ 222.386 │
│ Log-Likelihood │ -108.193 │
│ P-Value │ 0.398547 │
│ Model d.f. │ 2 │
╘════════════════╧═════════════╛

Model Parameters:
╒════════════╤═════════════╤════════════╤══════════════╕
│ Variable │ Estimate │ On Bound │ Std Error │
╞════════════╪═════════════╪════════════╪══════════════╡
│ g │ 5.01112 │ no │ 0.115015 │
│ b1 │ -4.2166e-16 │ yes │ Not Reported │
│ b2 │ 0 │ yes │ Not Reported │
│ b3 │ -0.011927 │ no │ 0.0018631 │
│ alpha │ 0.819548 │ no │ 0.104895 │
╘════════════╧═════════════╧════════════╧══════════════╛
Standard errors estimates are not generated for parameters estimated on corresponding bounds,
although sampling error is present for all parameters, as a rule. Standard error estimates may not
be reliable as a basis for confidence intervals or tests when one or more parameters are on bounds.


Goodness of Fit:
╒════════╤═════╤═══════════════╤═════════════════════╤═══════════════════╕
│ Dose │ N │ Sample Mean │ Model Fitted Mean │ Scaled Residual │
╞════════╪═════╪═══════════════╪═════════════════════╪═══════════════════╡
│ 0 │ 21 │ 4.87 │ 5.01112 │ -0.714375 │
│ 0.1 │ 18 │ 4.92 │ 5.01111 │ -0.427001 │
│ 0.5 │ 23 │ 5.21 │ 5.00963 │ 1.06145 │
│ 5 │ 20 │ 3.52 │ 3.52025 │ -0.00124313 │
╘════════╧═════╧═══════════════╧═════════════════════╧═══════════════════╛
╒════════╤═════╤═════════════╤═══════════════════╕
│ Dose │ N │ Sample SD │ Model Fitted SD │
╞════════╪═════╪═════════════╪═══════════════════╡
│ 0 │ 21 │ 1.027 │ 0.905289 │
│ 0.1 │ 18 │ 0.839 │ 0.905289 │
│ 0.5 │ 23 │ 0.807 │ 0.905289 │
│ 5 │ 20 │ 0.982 │ 0.905289 │
╘════════╧═════╧═════════════╧═══════════════════╛

Likelihoods:
╒═════════╤══════════════════╤════════════╤═════════╕
│ Model │ Log-Likelihood │ # Params │ AIC │
╞═════════╪══════════════════╪════════════╪═════════╡
│ A1 │ -107.273 │ 5 │ 224.547 │
│ A2 │ -106.407 │ 8 │ 228.813 │
│ A3 │ -107.273 │ 5 │ 224.547 │
│ fitted │ -108.193 │ 3 │ 222.386 │
│ reduced │ -124.812 │ 2 │ 253.624 │
╘═════════╧══════════════════╧════════════╧═════════╛

Tests of Mean and Variance Fits:
╒════════╤══════════════════════════════╤═════════════╤═════════════╕
│ Name │ -2 * Log(Likelihood Ratio) │ Test d.f. │ P-Value │
╞════════╪══════════════════════════════╪═════════════╪═════════════╡
│ Test 1 │ 36.8106 │ 6 │ 1.91706e-06 │
│ Test 2 │ 1.73346 │ 3 │ 0.629521 │
│ Test 3 │ 1.73346 │ 3 │ 0.629521 │
│ Test 4 │ 1.83986 │ 2 │ 0.398547 │
╘════════╧══════════════════════════════╧═════════════╧═════════════╛
Test 1: Test the null hypothesis that responses and variances don't differ among dose levels
(A2 vs R). If this test fails to reject the null hypothesis (p-value > 0.05), there may not be
a dose-response.

Test 2: Test the null hypothesis that variances are homogenous (A1 vs A2). If this test fails to
reject the null hypothesis (p-value > 0.05), the simpler constant variance model may be appropriate.

Test 3: Test the null hypothesis that the variances are adequately modeled (A3 vs A2). If this test
fails to reject the null hypothesis (p-value > 0.05), it may be inferred that the variances have
been modeled appropriately.

Test 4: Test the null hypothesis that the model for the mean fits the data (Fitted vs A3). If this
test fails to reject the null hypothesis (p-value > 0.1), the user has support for use of the
selected model.

# Offspring BW, Ms, F, PND4, DuPont (2010)

## Dataset

**Name:** Offspring BW, Ms, F, PND4, DuPont (2010)

| Dose | N | Mean | Std. Dev. |
| --- | --- | --- | --- |
| 0 | 22 | 3.04 | 0.392 |
| 0.1 | 21 | 3.05 | 0.473 |
| 0.5 | 24 | 3.17 | 0.338 |
| 5 | 20 | 2.59 | 0.34 |

Test 1 Dose Response: <0.0001

Test 2 Homogeneity of Variance: 0.3414

Test 3 Variance Model Selection: 0.3414

## Settings

| Setting | Value |
| --- | --- |
| BMR | 5% Relative Deviation |
| Distribution | Normal + Constant variance |
| Adverse Direction | Down (↓) |
| Maximum Polynomial Degree | 3 |
| Confidence Level (one sided) | 0.95 |

## Maximum Likelihood Approach

| Model | BMDL | BMD | BMDU | *P*-Value | AIC | Scaled Residual at Control | Scaled Residual near BMD | Recommendation and Notes |
| --- | --- | --- | --- | --- | --- | --- | --- | --- |
| Exponential 3 | 1.182 | 4.302 | 4.801 | 0.197 | 87.943 | -0.609 | <0.001 | **Viable** BMD/BMDL ratio > 3.0 |
| Exponential 5 | 1.182 | 4.379 | 4.799 | - | 89.943 | -0.609 | <0.001 | **Questionable** Zero degrees of freedom; saturated model BMD/BMDL ratio > 3.0 |
| Hill | 3.194 | 4.317 | 4.536 | - | 89.943 | -0.609 | -0. | **Questionable** Zero degrees of freedom; saturated model |
| Polynomial 2 | 1.254 | 2.779 | 3.365 | 0.407 | 86.075 | -0.627 | -0.012 | **Viable** |
| Polynomial 3^ab^ | 1.264 | 3.381 | 3.839 | 0.432 | 85.957 | -0.61 | -0.001 | **Recommended - Lowest AIC** |
| Power | 1.265 | 4.683 | 4.795 | 0.197 | 87.943 | -0.609 | <0.001 | **Viable** BMD/BMDL ratio > 3.0 |
| Linear | 1.168 | 1.54 | 2.287 | 0.21 | 87.395 | -0.816 | 1.437 | **Viable** |

^a^ BMDS recommended best fitting model

^b^ User selected best fitting model


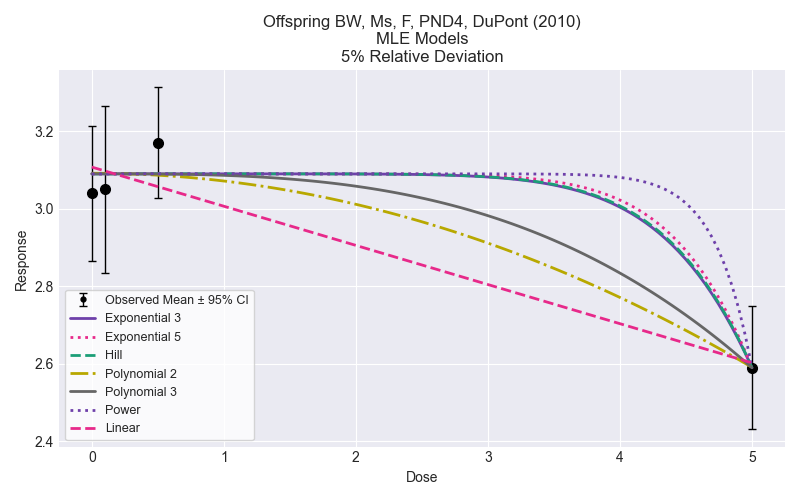


## Selected Model: Polynomial 3


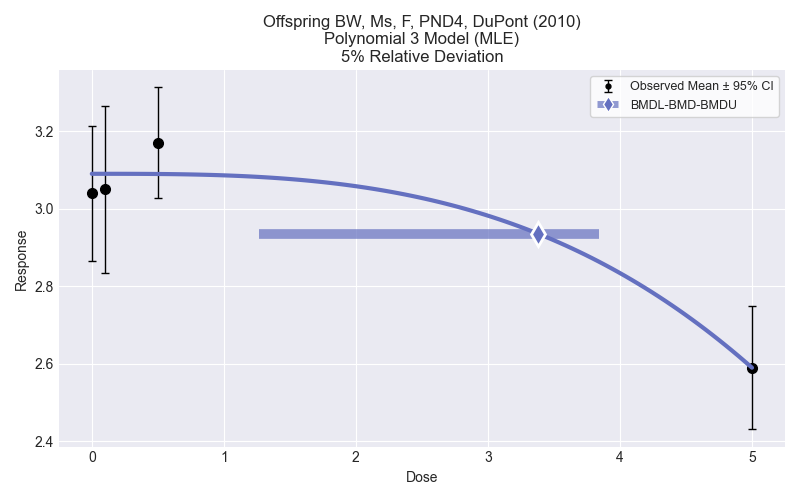


Polynomial 3 Model
══════════════════════════════

Version: pybmds 25.1 (bmdscore 25.1)

Input Summary:
╒══════════════════════════════╤════════════════════════════╕
│ BMR │ 5% Relative Deviation │
│ Distribution │ Normal + Constant variance │
│ Modeling Direction │ Down (↓) │
│ Confidence Level (one sided) │ 0.95 │
│ Modeling Approach │ MLE │
│ Degree │ 3 │
╘══════════════════════════════╧════════════════════════════╛

Parameter Settings:
╒═════════════╤═══════════╤═════════╤════════╕
│ Parameter │ Initial │ Min │ Max │
╞═════════════╪═══════════╪═════════╪════════╡
│ g │ 0 │ -1e+06 │ 1e+06 │
│ b1 │ 0 │ -1e+06 │ 0 │
│ b2 │ 0 │ -1e+06 │ 0 │
│ b3 │ 0 │ -1e+06 │ 0 │
│ alpha │ 0 │ -18 │ 18 │
╘═════════════╧═══════════╧═════════╧════════╛

Modeling Summary:
╒════════════════╤════════════╕
│ BMD │ 3.38083 │
│ BMDL │ 1.26407 │
│ BMDU │ 3.83854 │
│ AIC │ 85.9565 │
│ Log-Likelihood │ -39.9783 │
│ P-Value │ 0.431865 │
│ Model d.f. │ 2 │
╘════════════════╧════════════╛

Model Parameters:
╒════════════╤═════════════╤════════════╤══════════════╕
│ Variable │ Estimate │ On Bound │ Std Error │
╞════════════╪═════════════╪════════════╪══════════════╡
│ g │ 3.08985 │ no │ 0.046822 │
│ b1 │ 0 │ yes │ Not Reported │
│ b2 │ 0 │ yes │ Not Reported │
│ b3 │ -0.00399794 │ no │ 0.000781245 │
│ alpha │ 0.146777 │ no │ 0.00326643 │
╘════════════╧═════════════╧════════════╧══════════════╛
Standard errors estimates are not generated for parameters estimated on corresponding bounds,
although sampling error is present for all parameters, as a rule. Standard error estimates may not
be reliable as a basis for confidence intervals or tests when one or more parameters are on bounds.


Goodness of Fit:
╒════════╤═════╤═══════════════╤═════════════════════╤═══════════════════╕
│ Dose │ N │ Sample Mean │ Model Fitted Mean │ Scaled Residual │
╞════════╪═════╪═══════════════╪═════════════════════╪═══════════════════╡
│ 0 │ 22 │ 3.04 │ 3.08985 │ -0.610282 │
│ 0.1 │ 21 │ 3.05 │ 3.08984 │ -0.476589 │
│ 0.5 │ 24 │ 3.17 │ 3.08935 │ 1.03131 │
│ 5 │ 20 │ 2.59 │ 2.59011 │ -0.00122749 │
╘════════╧═════╧═══════════════╧═════════════════════╧═══════════════════╛
╒════════╤═════╤═════════════╤═══════════════════╕
│ Dose │ N │ Sample SD │ Model Fitted SD │
╞════════╪═════╪═════════════╪═══════════════════╡
│ 0 │ 22 │ 0.392 │ 0.383115 │
│ 0.1 │ 21 │ 0.473 │ 0.383115 │
│ 0.5 │ 24 │ 0.338 │ 0.383115 │
│ 5 │ 20 │ 0.34 │ 0.383115 │
╘════════╧═════╧═════════════╧═══════════════════╛

Likelihoods:
╒═════════╤══════════════════╤════════════╤══════════╕
│ Model │ Log-Likelihood │ # Params │ AIC │
╞═════════╪══════════════════╪════════════╪══════════╡
│ A1 │ -39.1386 │ 5 │ 88.2772 │
│ A2 │ -37.4661 │ 8 │ 90.9321 │
│ A3 │ -39.1386 │ 5 │ 88.2772 │
│ fitted │ -39.9783 │ 3 │ 85.9565 │
│ reduced │ -51.4256 │ 2 │ 106.851 │
╘═════════╧══════════════════╧════════════╧══════════╛

Tests of Mean and Variance Fits:
╒════════╤══════════════════════════════╤═════════════╤═════════════╕
│ Name │ -2 * Log(Likelihood Ratio) │ Test d.f. │ P-Value │
╞════════╪══════════════════════════════╪═════════════╪═════════════╡
│ Test 1 │ 27.919 │ 6 │ 9.73194e-05 │
│ Test 2 │ 3.34513 │ 3 │ 0.341411 │
│ Test 3 │ 3.34513 │ 3 │ 0.341411 │
│ Test 4 │ 1.67928 │ 2 │ 0.431865 │
╘════════╧══════════════════════════════╧═════════════╧═════════════╛
Test 1: Test the null hypothesis that responses and variances don't differ among dose levels
(A2 vs R). If this test fails to reject the null hypothesis (p-value > 0.05), there may not be
a dose-response.

Test 2: Test the null hypothesis that variances are homogenous (A1 vs A2). If this test fails to
reject the null hypothesis (p-value > 0.05), the simpler constant variance model may be appropriate.

Test 3: Test the null hypothesis that the variances are adequately modeled (A3 vs A2). If this test
fails to reject the null hypothesis (p-value > 0.05), it may be inferred that the variances have
been modeled appropriately.

Test 4: Test the null hypothesis that the model for the mean fits the data (Fitted vs A3). If this
test fails to reject the null hypothesis (p-value > 0.1), the user has support for use of the
selected model.

# Offspring BW, Ms, F, PND21, DuPont (2010)

## Dataset

**Name:** Offspring BW, Ms, F, PND21, DuPont (2010)

| Dose | N | Mean | Std. Dev. |
| --- | --- | --- | --- |
| 0 | 21 | 13.04 | 1.149 |
| 0.1 | 18 | 13.22 | 1.258 |
| 0.5 | 23 | 13.45 | 1.127 |
| 5 | 18 | 10.73 | 1.54 |

Test 1 Dose Response: <0.0001

Test 2 Homogeneity of Variance: 0.4862

Test 3 Variance Model Selection: 0.4862

## Settings

| Setting | Value |
| --- | --- |
| BMR | 5% Relative Deviation |
| Distribution | Normal + Constant variance |
| Adverse Direction | Down (↓) |
| Maximum Polynomial Degree | 3 |
| Confidence Level (one sided) | 0.95 |

## Maximum Likelihood Approach

| Model | BMDL | BMD | BMDU | *P*-Value | AIC | Scaled Residual at Control | Scaled Residual near BMD | Recommendation and Notes |
| --- | --- | --- | --- | --- | --- | --- | --- | --- |
| Exponential 3 | 1.105 | 4.169 | 4.709 | 0.27 | 269.721 | -0.754 | -0. | **Viable** BMD/BMDL ratio > 3.0 |
| Exponential 5 | 1.105 | 4.169 | 4.709 | - | 271.721 | -0.754 | -0. | **Questionable** Zero degrees of freedom; saturated model BMD/BMDL ratio > 3.0 |
| Hill | 1.066 | 4.205 | 4.499 | - | 271.721 | -0.754 | <0.001 | **Questionable** Zero degrees of freedom; saturated model BMD/BMDL ratio > 3.0 |
| Polynomial 2^ab^ | 1.165 | 2.564 | 2.892 | 0.503 | 267.88 | -0.786 | 0.856 | **Recommended - Lowest AIC** |
| Polynomial 3 | 1.176 | 3.192 | 3.478 | 0.267 | 269.739 | -0.755 | -0.009 | **Viable** |
| Power | 1.178 | 4.635 | 4.741 | 0.27 | 269.721 | -0.754 | 0 | **Viable** BMD/BMDL ratio > 3.0 |
| Linear | 1.07 | 1.3 | 1.662 | 0.193 | 269.794 | -1.097 | 1.392 | **Viable** |

^a^ BMDS recommended best fitting model

^b^ User selected best fitting model


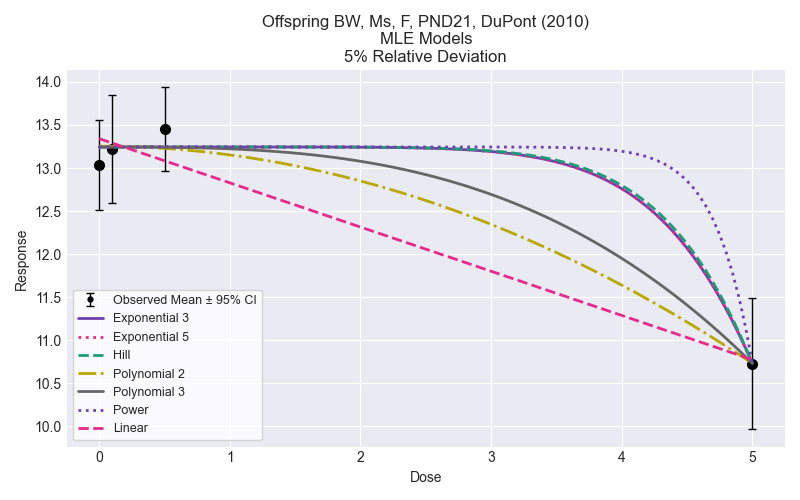


## Selected Model: Polynomial 2


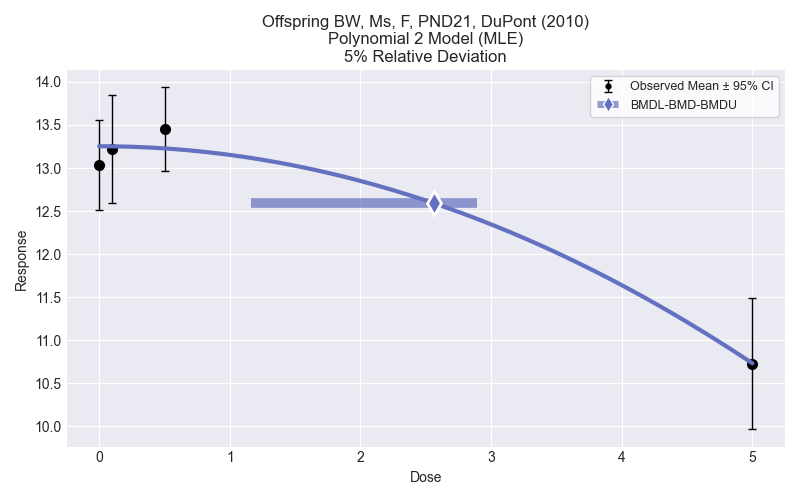


Polynomial 2 Model
══════════════════════════════

Version: pybmds 25.1 (bmdscore 25.1)

Input Summary:
╒══════════════════════════════╤════════════════════════════╕
│ BMR │ 5% Relative Deviation │
│ Distribution │ Normal + Constant variance │
│ Modeling Direction │ Down (↓) │
│ Confidence Level (one sided) │ 0.95 │
│ Modeling Approach │ MLE │
│ Degree │ 2 │
╘══════════════════════════════╧════════════════════════════╛

Parameter Settings:
╒═════════════╤═══════════╤═════════╤════════╕
│ Parameter │ Initial │ Min │ Max │
╞═════════════╪═══════════╪═════════╪════════╡
│ g │ 0 │ -1e+06 │ 1e+06 │
│ b1 │ 0 │ -1e+06 │ 0 │
│ b2 │ 0 │ -1e+06 │ 0 │
│ alpha │ 0 │ -18 │ 18 │
╘═════════════╧═══════════╧═════════╧════════╛

Modeling Summary:
╒════════════════╤═════════════╕
│ BMD │ 2.56379 │
│ BMDL │ 1.16511 │
│ BMDU │ 2.89201 │
│ AIC │ 267.88 │
│ Log-Likelihood │ -130.94 │
│ P-Value │ 0.502929 │
│ Model d.f. │ 2 │
╘════════════════╧═════════════╛

Model Parameters:
╒════════════╤════════════╤════════════╤══════════════╕
│ Variable │ Estimate │ On Bound │ Std Error │
╞════════════╪════════════╪════════════╪══════════════╡
│ g │ 13.2532 │ no │ 0.158514 │
│ b1 │ 0 │ yes │ Not Reported │
│ b2 │ -0.100815 │ no │ 0.0133661 │
│ alpha │ 1.54593 │ no │ 0.377889 │
╘════════════╧════════════╧════════════╧══════════════╛
Standard errors estimates are not generated for parameters estimated on corresponding bounds,
although sampling error is present for all parameters, as a rule. Standard error estimates may not
be reliable as a basis for confidence intervals or tests when one or more parameters are on bounds.


Goodness of Fit:
╒════════╤═════╤═══════════════╤═════════════════════╤═══════════════════╕
│ Dose │ N │ Sample Mean │ Model Fitted Mean │ Scaled Residual │
╞════════╪═════╪═══════════════╪═════════════════════╪═══════════════════╡
│ 0 │ 21 │ 13.04 │ 13.2532 │ -0.78571 │
│ 0.1 │ 18 │ 13.22 │ 13.2522 │ -0.10978 │
│ 0.5 │ 23 │ 13.45 │ 13.228 │ 0.856381 │
│ 5 │ 18 │ 10.73 │ 10.7328 │ -0.00959871 │
╘════════╧═════╧═══════════════╧═════════════════════╧═══════════════════╛
╒════════╤═════╤═════════════╤═══════════════════╕
│ Dose │ N │ Sample SD │ Model Fitted SD │
╞════════╪═════╪═════════════╪═══════════════════╡
│ 0 │ 21 │ 1.149 │ 1.24336 │
│ 0.1 │ 18 │ 1.258 │ 1.24336 │
│ 0.5 │ 23 │ 1.127 │ 1.24336 │
│ 5 │ 18 │ 1.54 │ 1.24336 │
╘════════╧═════╧═════════════╧═══════════════════╛

Likelihoods:
╒═════════╤══════════════════╤════════════╤═════════╕
│ Model │ Log-Likelihood │ # Params │ AIC │
╞═════════╪══════════════════╪════════════╪═════════╡
│ A1 │ -130.253 │ 5 │ 270.506 │
│ A2 │ -129.033 │ 8 │ 274.066 │
│ A3 │ -130.253 │ 5 │ 270.506 │
│ fitted │ -130.94 │ 3 │ 267.88 │
│ reduced │ -152.426 │ 2 │ 308.851 │
╘═════════╧══════════════════╧════════════╧═════════╛

Tests of Mean and Variance Fits:
╒════════╤══════════════════════════════╤═════════════╤═════════════╕
│ Name │ -2 * Log(Likelihood Ratio) │ Test d.f. │ P-Value │
╞════════╪══════════════════════════════╪═════════════╪═════════════╡
│ Test 1 │ 46.7856 │ 6 │ 2.06472e-08 │
│ Test 2 │ 2.44004 │ 3 │ 0.486225 │
│ Test 3 │ 2.44004 │ 3 │ 0.486225 │
│ Test 4 │ 1.37461 │ 2 │ 0.502929 │
╘════════╧══════════════════════════════╧═════════════╧═════════════╛
Test 1: Test the null hypothesis that responses and variances don't differ among dose levels
(A2 vs R). If this test fails to reject the null hypothesis (p-value > 0.05), there may not be
a dose-response.

Test 2: Test the null hypothesis that variances are homogenous (A1 vs A2). If this test fails to
reject the null hypothesis (p-value > 0.05), the simpler constant variance model may be appropriate.

Test 3: Test the null hypothesis that the variances are adequately modeled (A3 vs A2). If this test
fails to reject the null hypothesis (p-value > 0.05), it may be inferred that the variances have
been modeled appropriately.

Test 4: Test the null hypothesis that the model for the mean fits the data (Fitted vs A3). If this
test fails to reject the null hypothesis (p-value > 0.1), the user has support for use of the
selected model.

# Offspring BW gain, Ms, F, PND14-21, DuPont (2010)

## Dataset

**Name:** Offspring BW gain, Ms, F, PND14-21, DuPont (2010)

| Dose | N | Mean | Std. Dev. |
| --- | --- | --- | --- |
| 0 | 21 | 4.44 | 0.973 |
| 0.1 | 18 | 4.53 | 0.666 |
| 0.5 | 23 | 4.76 | 0.701 |
| 5 | 18 | 3.48 | 0.787 |

Test 1 Dose Response: <0.0001

Test 2 Homogeneity of Variance: 0.2986

Test 3 Variance Model Selection: 0.2986

## Settings

| Setting | Value |
| --- | --- |
| BMR | 5% Relative Deviation |
| Distribution | Normal + Constant variance |
| Adverse Direction | Down (↓) |
| Maximum Polynomial Degree | 3 |
| Confidence Level (one sided) | 0.95 |

## Maximum Likelihood Approach

| Model | BMDL | BMD | BMDU | *P*-Value | AIC | Scaled Residual at Control | Scaled Residual near BMD | Recommendation and Notes |
| --- | --- | --- | --- | --- | --- | --- | --- | --- |
| Exponential 3 | 0.784 | 4.057 | 4.688 | 0.159 | 195.839 | -0.848 | -0. | **Viable** BMD/BMDL ratio > 3.0 |
| Exponential 5 | 0.784 | 4.057 | 4.688 | - | 197.839 | -0.848 | 0 | **Questionable** Zero degrees of freedom; saturated model BMD/BMDL ratio > 3.0 |
| Hill | 0.516 | 3.525 | 3.694 | - | 197.839 | -0.848 | -0. | **Questionable** Zero degrees of freedom; saturated model BMD/BMDL ratio > 3.0 |
| Polynomial 2 | 0.862 | 2.252 | 2.299 | 0.142 | 196.01 | -0.877 | 1.103 | **Viable** |
| Polynomial 3^ab^ | 0.872 | 2.96 | 3.342 | 0.368 | 193.853 | -0.85 | -0.001 | **Recommended - Lowest AIC** BMD/BMDL ratio > 3.0 |
| Power | 0.873 | 4.576 | 4.685 | 0.159 | 195.839 | -0.848 | -0. | **Viable** BMD/BMDL ratio > 3.0 |
| Linear | 0.795 | 1.038 | 1.515 | 0.162 | 195.498 | -1.065 | 1.501 | **Viable** |

^a^ BMDS recommended best fitting model

^b^ User selected best fitting model


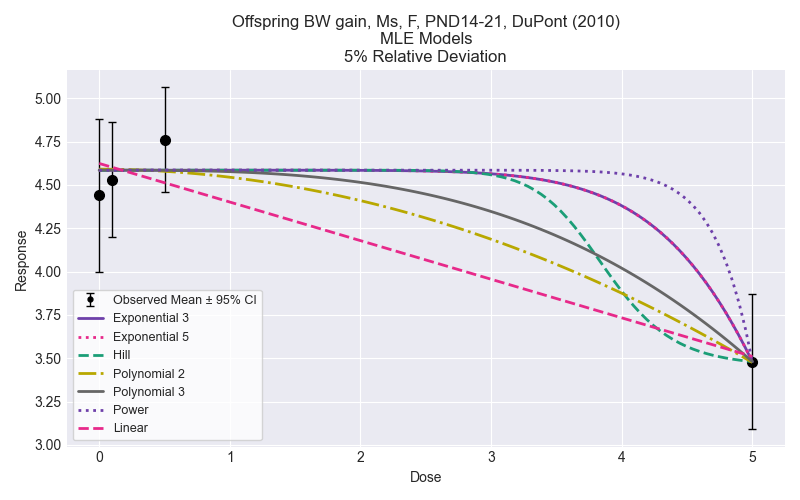


## Selected Model: Polynomial 3


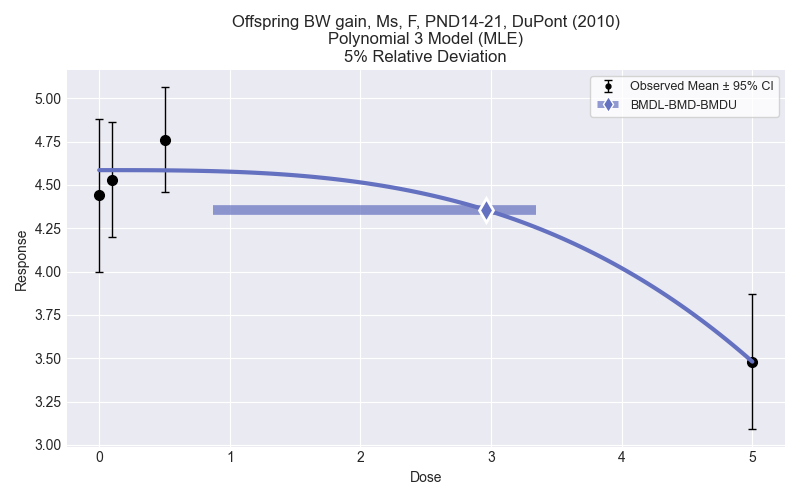


Polynomial 3 Model
══════════════════════════════

Version: pybmds 25.1 (bmdscore 25.1)

Input Summary:
╒══════════════════════════════╤════════════════════════════╕
│ BMR │ 5% Relative Deviation │
│ Distribution │ Normal + Constant variance │
│ Modeling Direction │ Down (↓) │
│ Confidence Level (one sided) │ 0.95 │
│ Modeling Approach │ MLE │
│ Degree │ 3 │
╘══════════════════════════════╧════════════════════════════╛

Parameter Settings:
╒═════════════╤═══════════╤═════════╤════════╕
│ Parameter │ Initial │ Min │ Max │
╞═════════════╪═══════════╪═════════╪════════╡
│ g │ 0 │ -1e+06 │ 1e+06 │
│ b1 │ 0 │ -1e+06 │ 0 │
│ b2 │ 0 │ -1e+06 │ 0 │
│ b3 │ 0 │ -1e+06 │ 0 │
│ alpha │ 0 │ -18 │ 18 │
╘═════════════╧═══════════╧═════════╧════════╛

Modeling Summary:
╒════════════════╤════════════╕
│ BMD │ 2.96 │
│ BMDL │ 0.871713 │
│ BMDU │ 3.34162 │
│ AIC │ 193.853 │
│ Log-Likelihood │ -93.9267 │
│ P-Value │ 0.368238 │
│ Model d.f. │ 2 │
╘════════════════╧════════════╛

Model Parameters:
╒════════════╤═════════════╤════════════╤══════════════╕
│ Variable │ Estimate │ On Bound │ Std Error │
╞════════════╪═════════════╪════════════╪══════════════╡
│ g │ 4.5852 │ no │ 0.0994553 │
│ b1 │ 0 │ yes │ Not Reported │
│ b2 │ 0 │ yes │ Not Reported │
│ b3 │ -0.00884002 │ no │ 0.00167737 │
│ alpha │ 0.612805 │ no │ 0.0593748 │
╘════════════╧═════════════╧════════════╧══════════════╛
Standard errors estimates are not generated for parameters estimated on corresponding bounds,
although sampling error is present for all parameters, as a rule. Standard error estimates may not
be reliable as a basis for confidence intervals or tests when one or more parameters are on bounds.


Goodness of Fit:
╒════════╤═════╤═══════════════╤═════════════════════╤═══════════════════╕
│ Dose │ N │ Sample Mean │ Model Fitted Mean │ Scaled Residual │
╞════════╪═════╪═══════════════╪═════════════════════╪═══════════════════╡
│ 0 │ 21 │ 4.44 │ 4.5852 │ -0.849965 │
│ 0.1 │ 18 │ 4.53 │ 4.58519 │ -0.299094 │
│ 0.5 │ 23 │ 4.76 │ 4.58409 │ 1.07769 │
│ 5 │ 18 │ 3.48 │ 3.48019 │ -0.0010431 │
╘════════╧═════╧═══════════════╧═════════════════════╧═══════════════════╛
╒════════╤═════╤═════════════╤═══════════════════╕
│ Dose │ N │ Sample SD │ Model Fitted SD │
╞════════╪═════╪═════════════╪═══════════════════╡
│ 0 │ 21 │ 0.973 │ 0.782819 │
│ 0.1 │ 18 │ 0.666 │ 0.782819 │
│ 0.5 │ 23 │ 0.701 │ 0.782819 │
│ 5 │ 18 │ 0.787 │ 0.782819 │
╘════════╧═════╧═════════════╧═══════════════════╛

Likelihoods:
╒═════════╤══════════════════╤════════════╤═════════╕
│ Model │ Log-Likelihood │ # Params │ AIC │
╞═════════╪══════════════════╪════════════╪═════════╡
│ A1 │ -92.9277 │ 5 │ 195.855 │
│ A2 │ -91.0894 │ 8 │ 198.179 │
│ A3 │ -92.9277 │ 5 │ 195.855 │
│ fitted │ -93.9267 │ 3 │ 193.853 │
│ reduced │ -105.847 │ 2 │ 215.693 │
╘═════════╧══════════════════╧════════════╧═════════╛

Tests of Mean and Variance Fits:
╒════════╤══════════════════════════════╤═════════════╤═════════════╕
│ Name │ -2 * Log(Likelihood Ratio) │ Test d.f. │ P-Value │
╞════════╪══════════════════════════════╪═════════════╪═════════════╡
│ Test 1 │ 29.5147 │ 6 │ 4.86008e-05 │
│ Test 2 │ 3.67663 │ 3 │ 0.298566 │
│ Test 3 │ 3.67663 │ 3 │ 0.298566 │
│ Test 4 │ 1.99805 │ 2 │ 0.368238 │
╘════════╧══════════════════════════════╧═════════════╧═════════════╛
Test 1: Test the null hypothesis that responses and variances don't differ among dose levels
(A2 vs R). If this test fails to reject the null hypothesis (p-value > 0.05), there may not be
a dose-response.

Test 2: Test the null hypothesis that variances are homogenous (A1 vs A2). If this test fails to
reject the null hypothesis (p-value > 0.05), the simpler constant variance model may be appropriate.

Test 3: Test the null hypothesis that the variances are adequately modeled (A3 vs A2). If this test
fails to reject the null hypothesis (p-value > 0.05), it may be inferred that the variances have
been modeled appropriately.

Test 4: Test the null hypothesis that the model for the mean fits the data (Fitted vs A3). If this
test fails to reject the null hypothesis (p-value > 0.1), the user has support for use of the
selected model.

# Offspring BW gain, Ms, F, PND21-40, DuPont (2010)

## Dataset

**Name:** Offspring BW gain, Ms, F, PND21-40, DuPont (2010)

| Dose | N | Mean | Std. Dev. |
| --- | --- | --- | --- |
| 0 | 20 | 11.2 | 1.29 |
| 0.1 | 18 | 10.4 | 1.34 |
| 0.5 | 23 | 10.7 | 1.14 |
| 5 | 18 | 12.9 | 1.93 |

Test 1 Dose Response: <0.0001

Test 2 Homogeneity of Variance: 0.0917

Test 3 Variance Model Selection: 0.0917

## Settings

| Setting | Value |
| --- | --- |
| BMR | 5% Relative Deviation |
| Distribution | Normal + Constant variance |
| Adverse Direction | Up (↑) |
| Maximum Polynomial Degree | 3 |
| Confidence Level (one sided) | 0.95 |

## Maximum Likelihood Approach

| Model | BMDL | BMD | BMDU | *P*-Value | AIC | Scaled Residual at Control | Scaled Residual near BMD | Recommendation and Notes |
| --- | --- | --- | --- | --- | --- | --- | --- | --- |
| Exponential 3 | 1.062 | 4.287 | 4.745 | 0.076 | 288.108 | 1.333 | <0.001 | **Questionable** Goodness of fit p-value < 0.1 BMD/BMDL ratio > 3.0 |
| Exponential 5 | 0.501 | 3.684 | 4.741 | - | 290.108 | 1.333 | <0.001 | **Questionable** Zero degrees of freedom; saturated model BMD/BMDL ratio > 3.0 |
| Hill | 0.507 | 4.338 | 4.649 | - | 290.108 | 1.333 | -0. | **Questionable** Zero degrees of freedom; saturated model BMD/BMDL ratio > 3.0 |
| Polynomial 2^ab^ | 0.97 | 2.513 | 3.025 | 0.202 | 286.153 | 1.358 | -0.299 | **Recommended - Lowest AIC** |
| Polynomial 3 | 0.972 | 3.157 | 3.579 | 0.076 | 288.112 | 1.335 | 0.003 | **Questionable** Goodness of fit p-value < 0.1 BMD/BMDL ratio > 3.0 |
| Power | 0.973 | 4.63 | 4.736 | 0.076 | 288.108 | 1.333 | -0. | **Questionable** Goodness of fit p-value < 0.1 BMD/BMDL ratio > 3.0 |
| Linear | 0.924 | 1.228 | 1.797 | 0.128 | 287.059 | 1.59 | -0.696 | **Viable** |

^a^ BMDS recommended best fitting model

^b^ User selected best fitting model


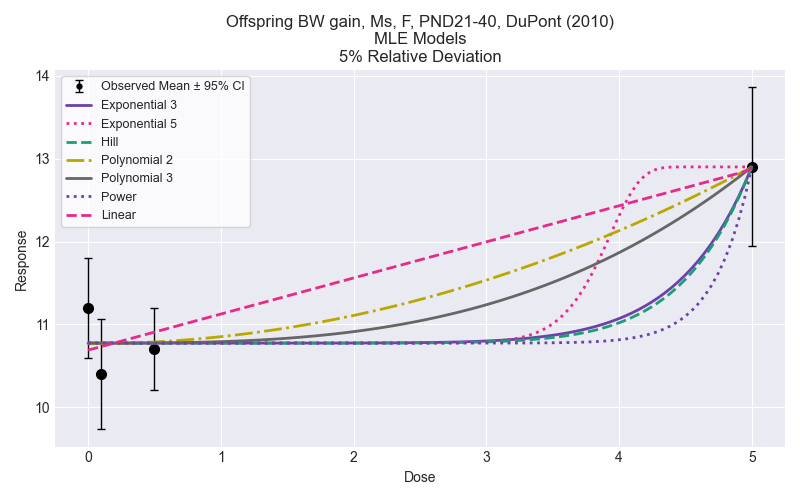


## Selected Model: Polynomial 2


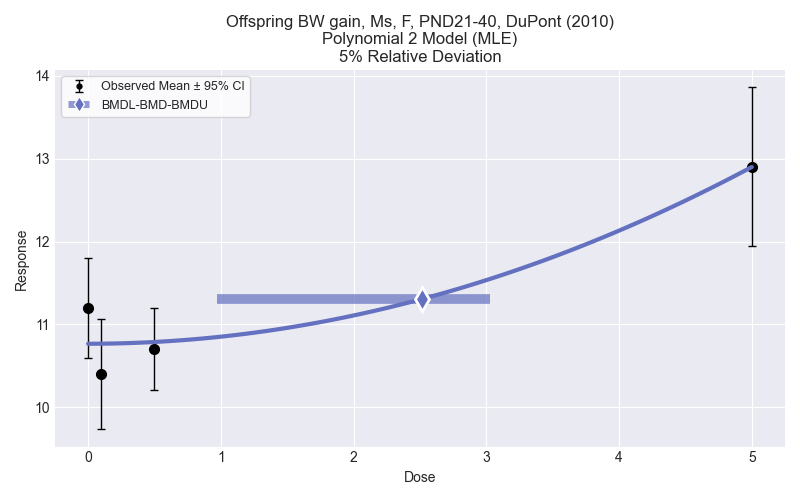


Polynomial 2 Model
══════════════════════════════

Version: pybmds 25.1 (bmdscore 25.1)

Input Summary:
╒══════════════════════════════╤════════════════════════════╕
│ BMR │ 5% Relative Deviation │
│ Distribution │ Normal + Constant variance │
│ Modeling Direction │ Up (↑) │
│ Confidence Level (one sided) │ 0.95 │
│ Modeling Approach │ MLE │
│ Degree │ 2 │
╘══════════════════════════════╧════════════════════════════╛

Parameter Settings:
╒═════════════╤═══════════╤═════════╤════════╕
│ Parameter │ Initial │ Min │ Max │
╞═════════════╪═══════════╪═════════╪════════╡
│ g │ 0 │ -1e+06 │ 1e+06 │
│ b1 │ 0 │ 0 │ 1e+06 │
│ b2 │ 0 │ 0 │ 1e+06 │
│ alpha │ 0 │ -18 │ 18 │
╘═════════════╧═══════════╧═════════╧════════╛

Modeling Summary:
╒════════════════╤═════════════╕
│ BMD │ 2.51285 │
│ BMDL │ 0.969745 │
│ BMDU │ 3.02465 │
│ AIC │ 286.153 │
│ Log-Likelihood │ -140.076 │
│ P-Value │ 0.202073 │
│ Model d.f. │ 2 │
╘════════════════╧═════════════╛

Model Parameters:
╒════════════╤══════════════╤════════════╤══════════════╕
│ Variable │ Estimate │ On Bound │ Std Error │
╞════════════╪══════════════╪════════════╪══════════════╡
│ g │ 10.7674 │ no │ 0.18317 │
│ b1 │ 1.22357e-14 │ yes │ Not Reported │
│ b2 │ 0.0852608 │ no │ 0.0153488 │
│ alpha │ 2.03066 │ no │ 0.656097 │
╘════════════╧══════════════╧════════════╧══════════════╛
Standard errors estimates are not generated for parameters estimated on corresponding bounds,
although sampling error is present for all parameters, as a rule. Standard error estimates may not
be reliable as a basis for confidence intervals or tests when one or more parameters are on bounds.


Goodness of Fit:
╒════════╤═════╤═══════════════╤═════════════════════╤═══════════════════╕
│ Dose │ N │ Sample Mean │ Model Fitted Mean │ Scaled Residual │
╞════════╪═════╪═══════════════╪═════════════════════╪═══════════════════╡
│ 0 │ 20 │ 11.2 │ 10.7674 │ 1.35754 │
│ 0.1 │ 18 │ 10.4 │ 10.7683 │ -1.09648 │
│ 0.5 │ 23 │ 10.7 │ 10.7887 │ -0.298673 │
│ 5 │ 18 │ 12.9 │ 12.899 │ 0.0031256 │
╘════════╧═════╧═══════════════╧═════════════════════╧═══════════════════╛
╒════════╤═════╤═════════════╤═══════════════════╕
│ Dose │ N │ Sample SD │ Model Fitted SD │
╞════════╪═════╪═════════════╪═══════════════════╡
│ 0 │ 20 │ 1.29 │ 1.42501 │
│ 0.1 │ 18 │ 1.34 │ 1.42501 │
│ 0.5 │ 23 │ 1.14 │ 1.42501 │
│ 5 │ 18 │ 1.93 │ 1.42501 │
╘════════╧═════╧═════════════╧═══════════════════╛

Likelihoods:
╒═════════╤══════════════════╤════════════╤═════════╕
│ Model │ Log-Likelihood │ # Params │ AIC │
╞═════════╪══════════════════╪════════════╪═════════╡
│ A1 │ -138.477 │ 5 │ 286.955 │
│ A2 │ -135.253 │ 8 │ 286.506 │
│ A3 │ -138.477 │ 5 │ 286.955 │
│ fitted │ -140.076 │ 3 │ 286.153 │
│ reduced │ -153.098 │ 2 │ 310.197 │
╘═════════╧══════════════════╧════════════╧═════════╛

Tests of Mean and Variance Fits:
╒════════╤══════════════════════════════╤═════════════╤═════════════╕
│ Name │ -2 * Log(Likelihood Ratio) │ Test d.f. │ P-Value │
╞════════╪══════════════════════════════╪═════════════╪═════════════╡
│ Test 1 │ 35.6904 │ 6 │ 3.16605e-06 │
│ Test 2 │ 6.44856 │ 3 │ 0.0917133 │
│ Test 3 │ 6.44856 │ 3 │ 0.0917133 │
│ Test 4 │ 3.19826 │ 2 │ 0.202073 │
╘════════╧══════════════════════════════╧═════════════╧═════════════╛
Test 1: Test the null hypothesis that responses and variances don't differ among dose levels
(A2 vs R). If this test fails to reject the null hypothesis (p-value > 0.05), there may not be
a dose-response.

Test 2: Test the null hypothesis that variances are homogenous (A1 vs A2). If this test fails to
reject the null hypothesis (p-value > 0.05), the simpler constant variance model may be appropriate.

Test 3: Test the null hypothesis that the variances are adequately modeled (A3 vs A2). If this test
fails to reject the null hypothesis (p-value > 0.05), it may be inferred that the variances have
been modeled appropriately.

Test 4: Test the null hypothesis that the model for the mean fits the data (Fitted vs A3). If this
test fails to reject the null hypothesis (p-value > 0.1), the user has support for use of the
selected model.

# Liver single cell necrosis, Ms, F0, Dams, Premating-LD21, DuPont (2010)

## Dataset

**Name:** Liver single cell necrosis, Ms, F0, Dams, Premating-LD21, DuPont (2010)

| Dose | N | Incidence |
| --- | --- | --- |
| 0 | 24 | 1 |
| 0.1 | 22 | 3 |
| 0.5 | 24 | 2 |
| 5 | 24 | 21 |

## Settings

| Setting | Value |
| --- | --- |
| BMR | 10% Extra Risk |
| Confidence Level (one sided) | 0.95 |
| Maximum Multistage Degree | 3 |

## Maximum Likelihood Approach

| Model | BMDL | BMD | BMDU | *P*-Value | AIC | Scaled Residual at Control | Scaled Residual near BMD | Recommendation and Notes |
| --- | --- | --- | --- | --- | --- | --- | --- | --- |
| Hill | 0.341 | 3.505 | 4.229 | - | 67.036 | -0.771 | <0.001 | **Questionable** Zero degrees of freedom; saturated model BMD/BMDL ratio > 3.0 |
| Gamma | 0.254 | 2.828 | 3.185 | 0.251 | 65.036 | -0.771 | <0.001 | **Viable** BMD/BMDL ratio > 3.0 |
| LogLogistic | 0.341 | 3.982 | 4.205 | 0.251 | 65.036 | -0.771 | -0. | **Viable** BMD/BMDL ratio > 3.0 |
| Multistage 1^a^ | 0.202 | 0.298 | 0.462 | 0.192 | 65.308 | -0.118 | 0.978 | **Recommended - Lowest BMDL** |
| Multistage 2 | 0.251 | 1.151 | 1.453 | 0.483 | 63.101 | -0.694 | -0.245 | **Viable** BMD/BMDL ratio > 3.0 |
| Multistage 3 | 0.255 | 1.587 | 2.192 | 0.244 | 65.015 | -0.689 | -0.192 | **Viable** BMD/BMDL ratio > 3.0 |
| Weibull | 0.254 | 4.111 | 4.359 | 0.518 | 63.036 | -0.771 | -0. | **Viable** BMD/BMDL ratio > 3.0 |
| Logistic | 0.731 | 1.041 | 1.46 | 0.467 | 63.105 | -0.572 | -0.391 | **Viable** |
| LogProbit | 0.385 | 2.861 | 4.513 | 0.251 | 65.036 | -0.771 | -0. | **Viable** BMD/BMDL ratio > 3.0 |
| Probit | 0.696 | 0.943 | 1.277 | 0.454 | 63.148 | -0.542 | -0.448 | **Viable** |
| Quantal Linear | 0.202 | 0.298 | 0.462 | 0.192 | 65.308 | -0.118 | 0.978 | **Viable** |

^a^ BMDS recommended best fitting model


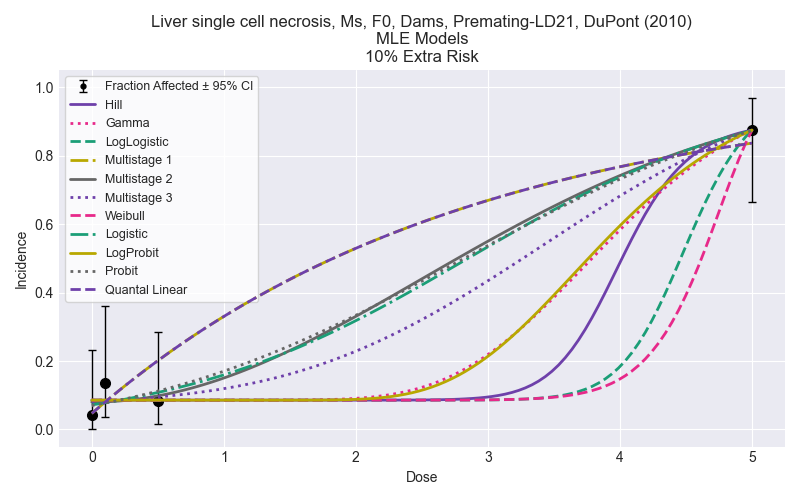


## Selected Model

No model was selected as a best-fitting model.

# Liver focal necrosis, Ms, F0, Dams, Premating-LD21, DuPont (2010)

## Dataset

**Name:** Liver focal necrosis, Ms, F0, Dams, Premating-LD21, DuPont (2010)

| Dose | N | Incidence |
| --- | --- | --- |
| 0 | 24 | 1 |
| 0.1 | 22 | 0 |
| 0.5 | 24 | 3 |
| 5 | 24 | 5 |

## Settings

| Setting | Value |
| --- | --- |
| BMR | 10% Extra Risk |
| Confidence Level (one sided) | 0.95 |
| Maximum Multistage Degree | 3 |

## Maximum Likelihood Approach

| Model | BMDL | BMD | BMDU | *P*-Value | AIC | Scaled Residual at Control | Scaled Residual near BMD | Recommendation and Notes |
| --- | --- | --- | --- | --- | --- | --- | --- | --- |
| Hill | 0.134 | 0.492 | 4.64 | - | 60.284 | 0.669 | <0.001 | **Questionable** Zero degrees of freedom; saturated model BMD/BMDL ratio > 3.0 |
| Gamma | 1.151 | 2.455 | 11.113 | 0.256 | 58.357 | -0.007 | 1.271 | **Viable** |
| LogLogistic^a^ | 0.935 | 2.247 | 10.934 | 0.267 | 58.272 | 0.038 | 1.241 | **Viable** |
| Multistage 1 | 1.151 | 2.455 | 11.115 | 0.256 | 58.357 | -0.007 | 1.271 | **Viable** |
| Multistage 2 | 1.151 | 2.455 | 11.114 | 0.256 | 58.357 | -0.007 | 1.271 | **Viable** |
| Multistage 3 | 1.151 | 2.455 | 11.114 | 0.256 | 58.357 | -0.007 | 1.271 | **Viable** |
| Weibull | 1.151 | 2.455 | 11.113 | 0.256 | 58.357 | -0.007 | 1.271 | **Viable** |
| Logistic | 2.38 | 3.569 | 11.405 | 0.216 | 58.814 | -0.225 | -0.066 | **Viable** |
| LogProbit^b^ | 0.182 | 1.324 | - | 0.178 | 59.652 | 0.378 | 0.789 | **Recommended - Lowest BMDL** BMD/BMDL ratio > 3.0 |
| Probit | 2.21 | 3.434 | 11.51 | 0.22 | 58.768 | -0.208 | -0.079 | **Viable** |
| Quantal Linear | 1.151 | 2.455 | 11.113 | 0.256 | 58.357 | -0.007 | 1.271 | **Viable** |

^a^ lowest AIC; no warnings; LogProbit is outlier model and is not consistent with data

^b^ BMDS recommended best fitting model


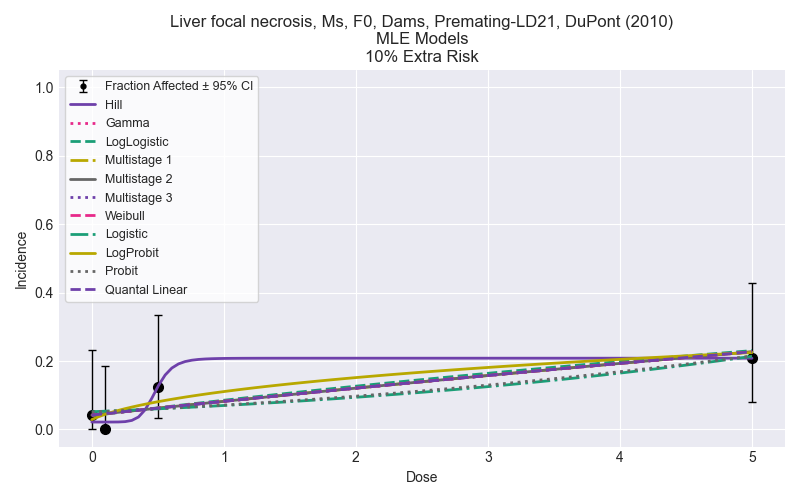


## Selected Model: LogLogistic


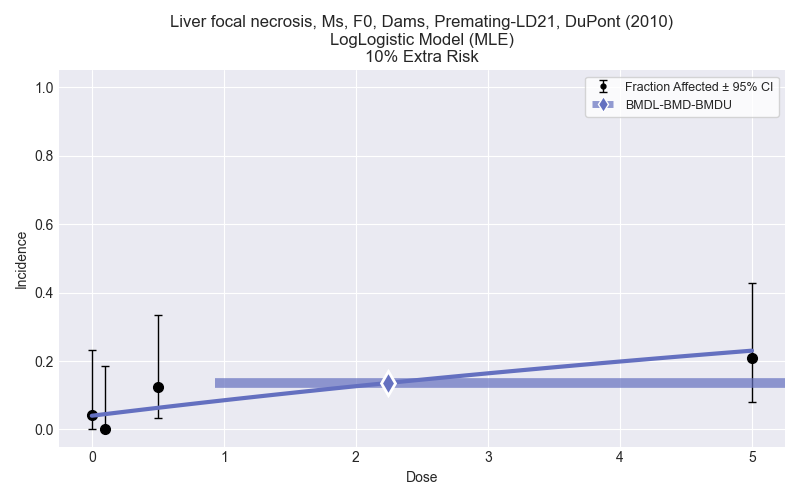


LogLogistic Model
══════════════════════════════

Version: pybmds 25.1 (bmdscore 25.1)

Input Summary:
╒══════════════════════════════╤════════════════════════╕
│ BMR │ 10% Extra Risk │
│ Confidence Level (one sided) │ 0.95 │
│ Modeling approach │ frequentist_restricted │
╘══════════════════════════════╧════════════════════════╛

Parameter Settings:
╒═════════════╤═══════════╤═══════╤═══════╕
│ Parameter │ Initial │ Min │ Max │
╞═════════════╪═══════════╪═══════╪═══════╡
│ g │ 0 │ -18 │ 18 │
│ a │ 0 │ -18 │ 18 │
│ b │ 1 │ 1 │ 18 │
╘═════════════╧═══════════╧═══════╧═══════╛

Modeling Summary:
╒════════════════╤════════════╕
│ BMD │ 2.2469 │
│ BMDL │ 0.934954 │
│ BMDU │ 10.9338 │
│ AIC │ 58.2717 │
│ Log-Likelihood │ -27.1359 │
│ P-Value │ 0.266927 │
│ Overall d.f. │ 2 │
│ Chi² │ 2.64156 │
╘════════════════╧════════════╛

Model Parameters:
╒════════════╤════════════╤════════════╤══════════════╕
│ Variable │ Estimate │ On Bound │ Std Error │
╞════════════╪════════════╪════════════╪══════════════╡
│ g │ 0.0401438 │ no │ 0.0477477 │
│ a │ -3.00678 │ no │ 0.636993 │
│ b │ 1 │ yes │ Not Reported │
╘════════════╧════════════╧════════════╧══════════════╛
Standard errors estimates are not generated for parameters estimated on corresponding bounds,
although sampling error is present for all parameters, as a rule. Standard error estimates may not
be reliable as a basis for confidence intervals or tests when one or more parameters are on bounds.


Goodness of Fit:
╒════════╤════════╤════════════╤════════════╤════════════╤═══════════════════╕
│ Dose │ Size │ Observed │ Expected │ Est Prob │ Scaled Residual │
╞════════╪════════╪════════════╪════════════╪════════════╪═══════════════════╡
│ 0 │ 24 │ 1 │ 0.96345 │ 0.0401438 │ 0.0380072 │
│ 0.1 │ 22 │ 0 │ 0.987073 │ 0.044867 │ -1.01658 │
│ 0.5 │ 24 │ 3 │ 1.51929 │ 0.0633039 │ 1.24122 │
│ 5 │ 24 │ 5 │ 5.53018 │ 0.230424 │ -0.256998 │
╘════════╧════════╧════════════╧════════════╧════════════╧═══════════════════╛

Analysis of Deviance:
╒═══════════════╤══════════════════╤════════════╤════════════╤═════════════╤═══════════╕
│ Model │ Log-Likelihood │ # Params │ Deviance │ Test d.f. │ P-Value │
╞═══════════════╪══════════════════╪════════════╪════════════╪═════════════╪═══════════╡
│ Full model │ -25.4812 │ 4 │ - │ - │ - │
│ Fitted model │ -27.1359 │ 2 │ 3.30937 │ 2 │ 0.191152 │
│ Reduced model │ -29.6693 │ 1 │ 8.37632 │ 3 │ 0.038842 │
╘═══════════════╧══════════════════╧════════════╧════════════╧═════════════╧═══════════╛

# Liver hepatocellular hypertrophy, Ms, F0, Dams, Premating-LD21, DuPont (2010)

## Dataset

**Name:** Liver hepatocellular hypertrophy, Ms, F0, Dams, Premating-LD21, DuPont (2010)

| Dose | N | Incidence |
| --- | --- | --- |
| 0 | 24 | 0 |
| 0.1 | 22 | 0 |
| 0.5 | 24 | 14 |
| 5 | 24 | 24 |

## Settings

| Setting | Value |
| --- | --- |
| BMR | 10% Extra Risk |
| Confidence Level (one sided) | 0.95 |
| Maximum Multistage Degree | 3 |

## Maximum Likelihood Approach

| Model | BMDL | BMD | BMDU | *P*-Value | AIC | Scaled Residual at Control | Scaled Residual near BMD | Recommendation and Notes |
| --- | --- | --- | --- | --- | --- | --- | --- | --- |
| Hill | 0.14 | 0.359 | 0.396 | 1. | 36.602 | -0.001 | <0.001 | **Viable** |
| Gamma | 0.135 | 0.298 | 0.351 | 0.999 | 36.605 | -0.001 | -0.042 | **Viable** |
| LogLogistic | 0.14 | 0.359 | 0.396 | 1. | 36.602 | -0.001 | <0.001 | **Viable** |
| Multistage 1^a^ | 0.051 | 0.077 | 0.119 | 0.264 | 41.417 | -0.001 | -1.801 | **Recommended - Lowest BMDL** |
| Multistage 2^b^ | 0.108 | 0.178 | 0.227 | 0.854 | 36.099 | -0.001 | -0.86 | **Viable** |
| Multistage 3 | 0.135 | 0.248 | 0.291 | 0.985 | 34.908 | -0.001 | -0.391 | **Viable** |
| Weibull | 0 | 0.189 | 0.254 | 0.703 | 37.909 | -0.001 | -0.787 | **Unusable** BMDL does not exist |
| Logistic | 0.224 | 0.373 | 0.407 | 0.994 | 36.625 | -0.039 | 0.005 | **Viable** |
| LogProbit | 0.13 | 0.36 | 0.436 | 1. | 36.601 | -0.001 | -0. | **Viable** |
| Probit | 0.149 | 0.207 | 0.283 | 0.167 | 42.264 | -0.853 | -1.217 | **Viable** |
| Quantal Linear | 0.051 | 0.077 | 0.119 | 0.264 | 41.417 | -0.001 | -1.801 | **Viable** |

^a^ BMDS recommended best fitting model

^b^ lowest AIC; better visual fit; smaller scaled residual; similar BMDL


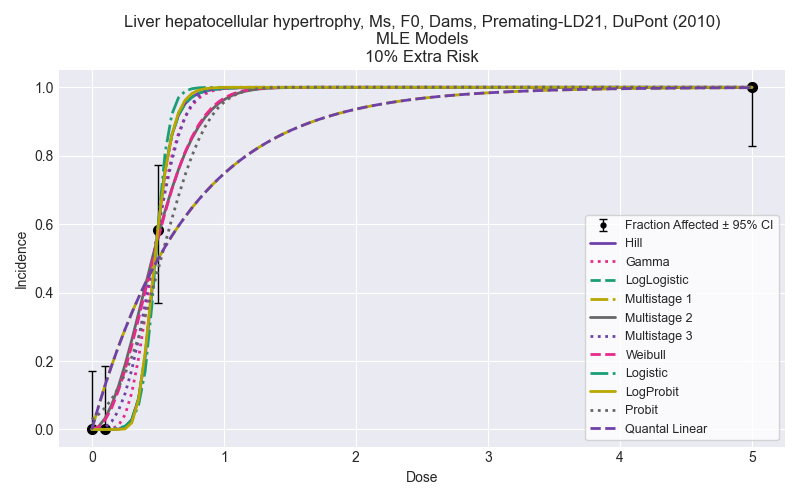


## Selected Model: Multistage 2


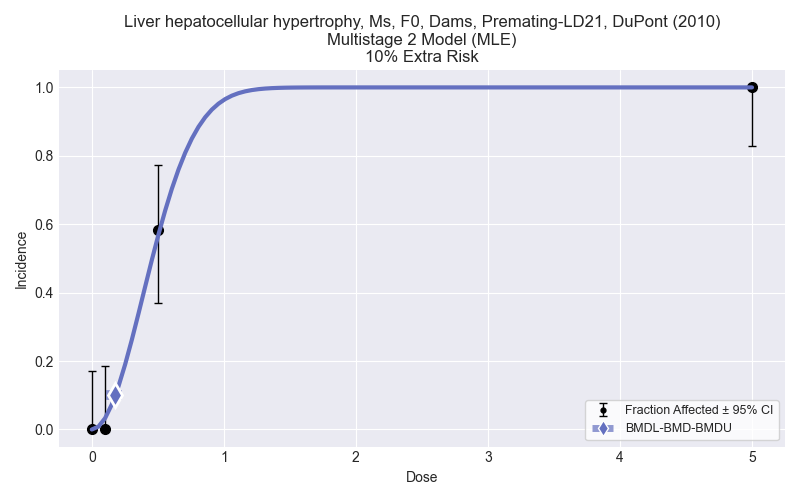


Multistage 2 Model
══════════════════════════════

Version: pybmds 25.1 (bmdscore 25.1)

Input Summary:
╒══════════════════════════════╤════════════════════════╕
│ BMR │ 10% Extra Risk │
│ Confidence Level (one sided) │ 0.95 │
│ Modeling approach │ frequentist_restricted │
│ Degree │ 2 │
╘══════════════════════════════╧════════════════════════╛

Parameter Settings:
╒═════════════╤═══════════╤═══════╤═══════╕
│ Parameter │ Initial │ Min │ Max │
╞═════════════╪═══════════╪═══════╪═══════╡
│ g │ 0 │ -18 │ 18 │
│ b1 │ 0 │ 0 │ 10000 │
│ b2 │ 0 │ 0 │ 10000 │
╘═════════════╧═══════════╧═══════╧═══════╛

Modeling Summary:
╒════════════════╤════════════╕
│ BMD │ 0.178451 │
│ BMDL │ 0.108389 │
│ BMDU │ 0.22744 │
│ AIC │ 36.0987 │
│ Log-Likelihood │ -17.0494 │
│ P-Value │ 0.853868 │
│ Overall d.f. │ 3 │
│ Chi² │ 0.781577 │
╘════════════════╧════════════╛

Model Parameters:
╒════════════╤═════════════╤════════════╤══════════════╕
│ Variable │ Estimate │ On Bound │ Std Error │
╞════════════╪═════════════╪════════════╪══════════════╡
│ g │ 1.523e-08 │ yes │ Not Reported │
│ b1 │ 2.09284e-17 │ yes │ Not Reported │
│ b2 │ 3.30855 │ no │ 22.7414 │
╘════════════╧═════════════╧════════════╧══════════════╛
Standard errors estimates are not generated for parameters estimated on corresponding bounds,
although sampling error is present for all parameters, as a rule. Standard error estimates may not
be reliable as a basis for confidence intervals or tests when one or more parameters are on bounds.


Goodness of Fit:
╒════════╤════════╤════════════╤═════════════╤════════════╤═══════════════════╕
│ Dose │ Size │ Observed │ Expected │ Est Prob │ Scaled Residual │
╞════════╪════════╪════════════╪═════════════╪════════════╪═══════════════════╡
│ 0 │ 24 │ 0 │ 3.6552e-07 │ 1.523e-08 │ -0.000604582 │
│ 0.1 │ 22 │ 0 │ 0.715972 │ 0.0325442 │ -0.860266 │
│ 0.5 │ 24 │ 14 │ 13.5048 │ 0.562701 │ 0.203764 │
│ 5 │ 24 │ 24 │ 24 │ 1 │ 0 │
╘════════╧════════╧════════════╧═════════════╧════════════╧═══════════════════╛

Analysis of Deviance:
╒═══════════════╤══════════════════╤════════════╤════════════╤═════════════╤═══════════╕
│ Model │ Log-Likelihood │ # Params │ Deviance │ Test d.f. │ P-Value │
╞═══════════════╪══════════════════╪════════════╪════════════╪═════════════╪═══════════╡
│ Full model │ -16.3006 │ 4 │ - │ - │ - │
│ Fitted model │ -17.0494 │ 1 │ 1.49744 │ 3 │ 0.682861 │
│ Reduced model │ -63.4217 │ 1 │ 94.2422 │ 3 │ 0 │
╘═══════════════╧══════════════════╧════════════╧════════════╧═════════════╧═══════════╛

# Abs kidney wt, Ms, F0, Dams, Premating-LD21, DuPont (2010)

## Dataset

**Name:** Abs kidney wt, Ms, F0, Dams, Premating-LD21, DuPont (2010)

| Dose | N | Mean | Std. Dev. |
| --- | --- | --- | --- |
| 0 | 21 | 0.477 | 0.044 |
| 0.1 | 18 | 0.501 | 0.039 |
| 0.5 | 23 | 0.505 | 0.046 |
| 5 | 20 | 0.577 | 0.05 |

Test 1 Dose Response: <0.0001

Test 2 Homogeneity of Variance: 0.7325

Test 3 Variance Model Selection: 0.7325

## Settings

| Setting | Value |
| --- | --- |
| BMR | 10% Relative Deviation |
| Distribution | Normal + Constant variance |
| Adverse Direction | Up (↑) |
| Maximum Polynomial Degree | 3 |
| Confidence Level (one sided) | 0.95 |

## Maximum Likelihood Approach

| Model | BMDL | BMD | BMDU | *P*-Value | AIC | Scaled Residual at Control | Scaled Residual near BMD | Recommendation and Notes |
| --- | --- | --- | --- | --- | --- | --- | --- | --- |
| Exponential 3 | 2.392 | 2.916 | 4.938 | 0.217 | -270.733 | -1.372 | -0.071 | **Viable** |
| Exponential 5^ab^ | 0.554 | 1.328 | 3.982 | 0.167 | -269.877 | -0.776 | -0.247 | **Recommended - Lowest BMDL** |
| Hill | 0.465 | 2.547 | 4.113 | 0.099 | -269.074 | -1.269 | 0.489 | **Questionable** Goodness of fit p-value < 0.1 BMD/BMDL ratio > 3.0 |
| Polynomial 2 | 2.255 | 2.803 | 4.193 | 0.228 | -270.837 | -1.342 | -0.077 | **Viable** |
| Polynomial 3 | 2.255 | 2.802 | 4.436 | 0.228 | -270.837 | -1.341 | -0.079 | **Viable** |
| Power | 2.255 | 2.802 | 4.874 | 0.228 | -270.837 | -1.34 | -0.079 | **Viable** |
| Linear | 2.255 | 2.802 | 3.666 | 0.228 | -270.837 | -1.34 | -0.079 | **Viable** |

^a^ BMDS recommended best fitting model

^b^ User selected best fitting model


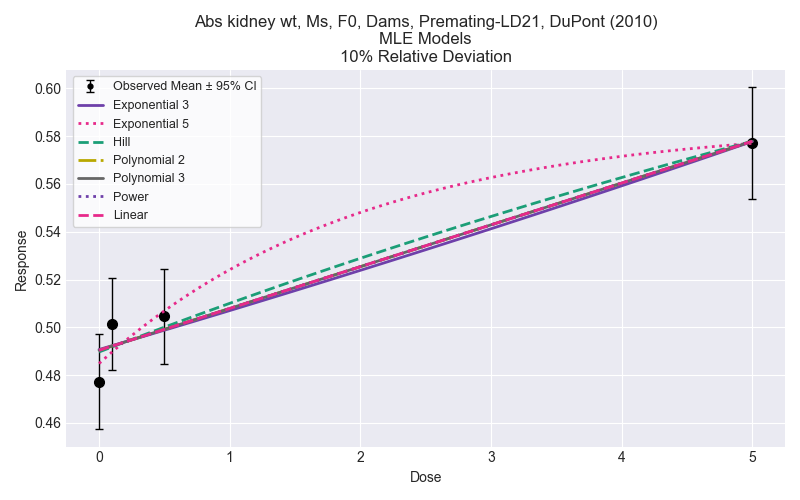


## Selected Model: Exponential 5


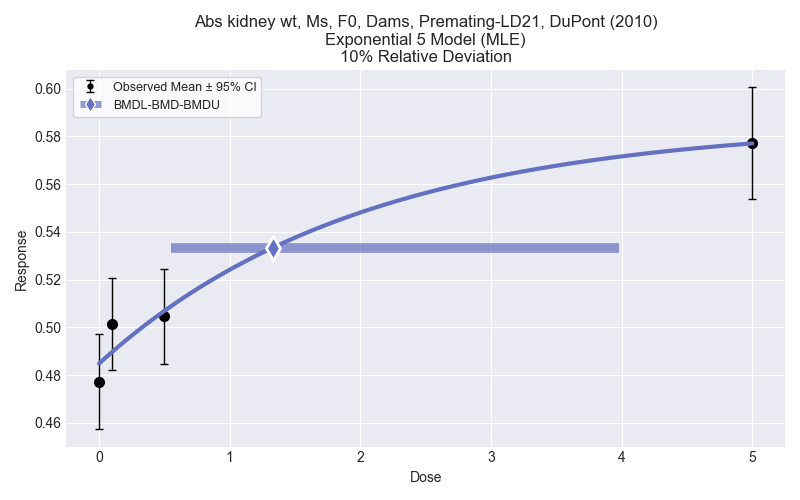


Exponential 5 Model
══════════════════════════════

Version: pybmds 25.1 (bmdscore 25.1)

Input Summary:
╒══════════════════════════════╤════════════════════════════╕
│ BMR │ 10% Relative Deviation │
│ Distribution │ Normal + Constant variance │
│ Modeling Direction │ Up (↑) │
│ Confidence Level (one sided) │ 0.95 │
│ Modeling Approach │ MLE │
╘══════════════════════════════╧════════════════════════════╛

Parameter Settings:
╒═════════════╤═══════════╤═══════╤═══════╕
│ Parameter │ Initial │ Min │ Max │
╞═════════════╪═══════════╪═══════╪═══════╡
│ a │ 0 │ 0 │ 100 │
│ b │ 0 │ 0 │ 100 │
│ c │ 0 │ 0 │ 20 │
│ d │ 1 │ 1 │ 18 │
│ log-alpha │ 0 │ -18 │ 18 │
╘═════════════╧═══════════╧═══════╧═══════╛

Modeling Summary:
╒════════════════╤═════════════╕
│ BMD │ 1.32757 │
│ BMDL │ 0.55425 │
│ BMDU │ 3.98237 │
│ AIC │ -269.877 │
│ Log-Likelihood │ 138.939 │
│ P-Value │ 0.166537 │
│ Model d.f. │ 1 │
╘════════════════╧═════════════╛

Model Parameters:
╒════════════╤════════════╤════════════╤══════════════╕
│ Variable │ Estimate │ On Bound │ Std Error │
╞════════════╪════════════╪════════════╪══════════════╡
│ a │ 0.484823 │ no │ 0.00807401 │
│ b │ 0.494836 │ no │ 0.414025 │
│ c │ 1.20766 │ no │ 0.0354171 │
│ d │ 1 │ yes │ Not Reported │
│ log-alpha │ -6.22663 │ no │ 0.156174 │
╘════════════╧════════════╧════════════╧══════════════╛
Standard errors estimates are not generated for parameters estimated on corresponding bounds,
although sampling error is present for all parameters, as a rule. Standard error estimates may not
be reliable as a basis for confidence intervals or tests when one or more parameters are on bounds.


Goodness of Fit:
╒════════╤═════╤═══════════════╤═════════════════════╤═══════════════════╕
│ Dose │ N │ Sample Mean │ Model Fitted Mean │ Scaled Residual │
╞════════╪═════╪═══════════════╪═════════════════════╪═══════════════════╡
│ 0 │ 21 │ 0.4773 │ 0.484823 │ -0.775566 │
│ 0.1 │ 18 │ 0.5013 │ 0.489684 │ 1.10863 │
│ 0.5 │ 23 │ 0.5046 │ 0.506891 │ -0.24711 │
│ 5 │ 20 │ 0.5771 │ 0.577021 │ 0.00797834 │
╘════════╧═════╧═══════════════╧═════════════════════╧═══════════════════╛
╒════════╤═════╤═════════════╤═══════════════════╕
│ Dose │ N │ Sample SD │ Model Fitted SD │
╞════════╪═════╪═════════════╪═══════════════════╡
│ 0 │ 21 │ 0.04412 │ 0.0444534 │
│ 0.1 │ 18 │ 0.03868 │ 0.0444534 │
│ 0.5 │ 23 │ 0.04599 │ 0.0444534 │
│ 5 │ 20 │ 0.04995 │ 0.0444534 │
╘════════╧═════╧═════════════╧═══════════════════╛

Likelihoods:
╒═════════╤══════════════════╤════════════╤══════════╕
│ Model │ Log-Likelihood │ # Params │ AIC │
╞═════════╪══════════════════╪════════════╪══════════╡
│ A1 │ 139.896 │ 5 │ -269.791 │
│ A2 │ 140.539 │ 8 │ -265.077 │
│ A3 │ 139.896 │ 5 │ -269.791 │
│ fitted │ 138.939 │ 4 │ -269.877 │
│ reduced │ 117.834 │ 2 │ -231.668 │
╘═════════╧══════════════════╧════════════╧══════════╛

Tests of Mean and Variance Fits:
╒════════╤══════════════════════════════╤═════════════╤═════════════╕
│ Name │ -2 * Log(Likelihood Ratio) │ Test d.f. │ P-Value │
╞════════╪══════════════════════════════╪═════════════╪═════════════╡
│ Test 1 │ 45.409 │ 6 │ 3.88124e-08 │
│ Test 2 │ 1.28603 │ 3 │ 0.732453 │
│ Test 3 │ 1.28603 │ 3 │ 0.732453 │
│ Test 4 │ 1.91385 │ 1 │ 0.166537 │
╘════════╧══════════════════════════════╧═════════════╧═════════════╛
Test 1: Test the null hypothesis that responses and variances don't differ among dose levels
(A2 vs R). If this test fails to reject the null hypothesis (p-value > 0.05), there may not be
a dose-response.

Test 2: Test the null hypothesis that variances are homogenous (A1 vs A2). If this test fails to
reject the null hypothesis (p-value > 0.05), the simpler constant variance model may be appropriate.

Test 3: Test the null hypothesis that the variances are adequately modeled (A3 vs A2). If this test
fails to reject the null hypothesis (p-value > 0.05), it may be inferred that the variances have
been modeled appropriately.

Test 4: Test the null hypothesis that the model for the mean fits the data (Fitted vs A3). If this
test fails to reject the null hypothesis (p-value > 0.1), the user has support for use of the
selected model.

# Rel kidney wt (brain), Ms, F0, Dams, Premating-LD21, DuPont (2010)

## Dataset

**Name:** Rel kidney wt (brain), Ms, F0, Dams, Premating-LD21, DuPont (2010)

| Dose | N | Mean | Std. Dev. |
| --- | --- | --- | --- |
| 0 | 21 | 97.006 | 8.243 |
| 0.1 | 18 | 100.797 | 9.529 |
| 0.5 | 23 | 101.248 | 9.523 |
| 5 | 20 | 116.698 | 8.478 |

Test 1 Dose Response: <0.0001

Test 2 Homogeneity of Variance: 0.8698

Test 3 Variance Model Selection: 0.8698

## Settings

| Setting | Value |
| --- | --- |
| BMR | 10% Relative Deviation |
| Distribution | Normal + Constant variance |
| Adverse Direction | Up (↑) |
| Maximum Polynomial Degree | 3 |
| Confidence Level (one sided) | 0.95 |

## Maximum Likelihood Approach

| Model | BMDL | BMD | BMDU | *P*-Value | AIC | Scaled Residual at Control | Scaled Residual near BMD | Recommendation and Notes |
| --- | --- | --- | --- | --- | --- | --- | --- | --- |
| Exponential 3 | 2.373 | 2.874 | 4.917 | 0.429 | 595.929 | -1.005 | -0.044 | **Viable** |
| Exponential 5^ab^ | 0.675 | 1.751 | 4.897 | 0.264 | 597.484 | -0.635 | -0.188 | **Recommended - Lowest BMDL** |
| Hill | 1.934 | 2.508 | 3.859 | 0.223 | 597.72 | -0.901 | 0.21 | **Viable** |
| Polynomial 2 | 2.238 | 2.753 | 4.197 | 0.444 | 595.862 | -0.968 | -0.065 | **Viable** |
| Polynomial 3 | 2.238 | 2.76 | 4.446 | 0.444 | 595.861 | -0.974 | -0.048 | **Viable** |
| Power | 2.238 | 2.76 | 4.951 | 0.444 | 595.861 | -0.974 | -0.048 | **Viable** |
| Linear | 2.238 | 2.76 | 3.575 | 0.444 | 595.861 | -0.974 | -0.048 | **Viable** |

^a^ BMDS recommended best fitting model

^b^ User selected best fitting model


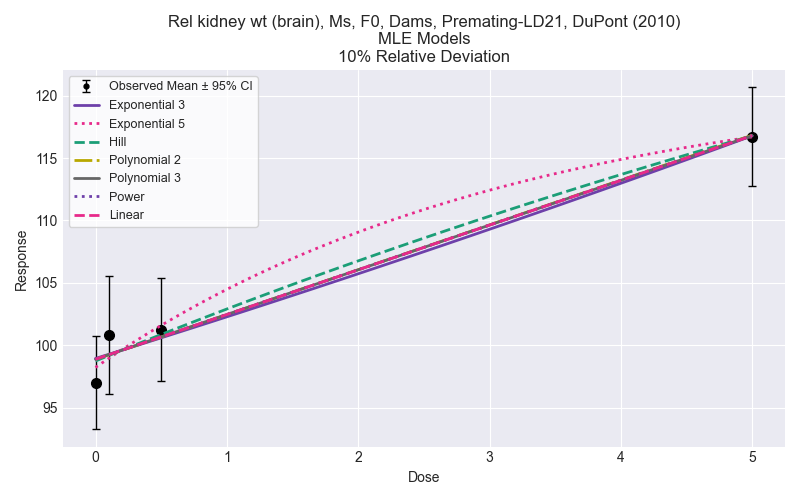


## Selected Model: Exponential 5


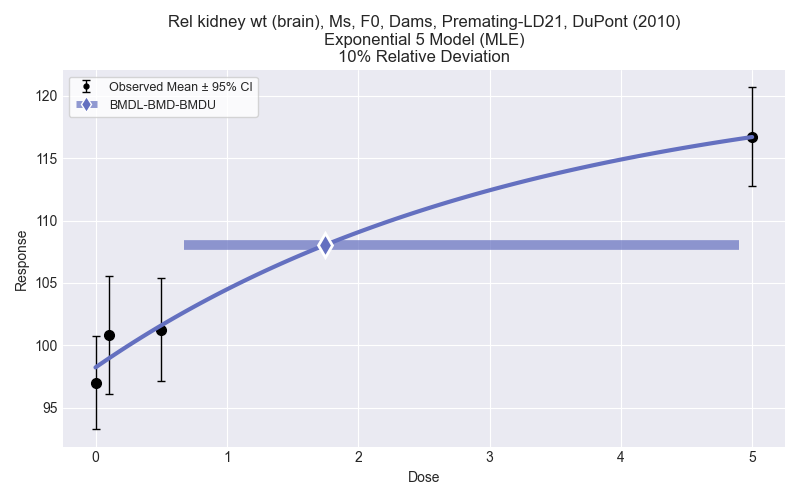


Exponential 5 Model
══════════════════════════════

Version: pybmds 25.1 (bmdscore 25.1)

Input Summary:
╒══════════════════════════════╤════════════════════════════╕
│ BMR │ 10% Relative Deviation │
│ Distribution │ Normal + Constant variance │
│ Modeling Direction │ Up (↑) │
│ Confidence Level (one sided) │ 0.95 │
│ Modeling Approach │ MLE │
╘══════════════════════════════╧════════════════════════════╛

Parameter Settings:
╒═════════════╤═══════════╤═══════╤═══════╕
│ Parameter │ Initial │ Min │ Max │
╞═════════════╪═══════════╪═══════╪═══════╡
│ a │ 0 │ 0 │ 100 │
│ b │ 0 │ 0 │ 100 │
│ c │ 0 │ 0 │ 20 │
│ d │ 1 │ 1 │ 18 │
│ log-alpha │ 0 │ -18 │ 18 │
╘═════════════╧═══════════╧═══════╧═══════╛

Modeling Summary:
╒════════════════╤═════════════╕
│ BMD │ 1.75091 │
│ BMDL │ 0.67538 │
│ BMDU │ 4.89718 │
│ AIC │ 597.484 │
│ Log-Likelihood │ -294.742 │
│ P-Value │ 0.264202 │
│ Model d.f. │ 1 │
╘════════════════╧═════════════╛

Model Parameters:
╒════════════╤════════════╤════════════╤══════════════╕
│ Variable │ Estimate │ On Bound │ Std Error │
╞════════════╪════════════╪════════════╪══════════════╡
│ a │ 98.2255 │ no │ 1.58693 │
│ b │ 0.310403 │ no │ 0.436729 │
│ c │ 1.2385 │ no │ 0.106983 │
│ d │ 1 │ yes │ Not Reported │
│ log-alpha │ 4.35095 │ no │ 0.156173 │
╘════════════╧════════════╧════════════╧══════════════╛
Standard errors estimates are not generated for parameters estimated on corresponding bounds,
although sampling error is present for all parameters, as a rule. Standard error estimates may not
be reliable as a basis for confidence intervals or tests when one or more parameters are on bounds.


Goodness of Fit:
╒════════╤═════╤═══════════════╤═════════════════════╤═══════════════════╕
│ Dose │ N │ Sample Mean │ Model Fitted Mean │ Scaled Residual │
╞════════╪═════╪═══════════════╪═════════════════════╪═══════════════════╡
│ 0 │ 21 │ 97.006 │ 98.2255 │ -0.634594 │
│ 0.1 │ 18 │ 100.797 │ 98.9415 │ 0.893915 │
│ 0.5 │ 23 │ 101.248 │ 101.593 │ -0.188064 │
│ 5 │ 20 │ 116.698 │ 116.69 │ 0.00389924 │
╘════════╧═════╧═══════════════╧═════════════════════╧═══════════════════╛
╒════════╤═════╤═════════════╤═══════════════════╕
│ Dose │ N │ Sample SD │ Model Fitted SD │
╞════════╪═════╪═════════════╪═══════════════════╡
│ 0 │ 21 │ 8.2431 │ 8.80636 │
│ 0.1 │ 18 │ 9.5287 │ 8.80636 │
│ 0.5 │ 23 │ 9.5233 │ 8.80636 │
│ 5 │ 20 │ 8.4777 │ 8.80636 │
╘════════╧═════╧═════════════╧═══════════════════╛

Likelihoods:
╒═════════╤══════════════════╤════════════╤═════════╕
│ Model │ Log-Likelihood │ # Params │ AIC │
╞═════════╪══════════════════╪════════════╪═════════╡
│ A1 │ -294.119 │ 5 │ 598.237 │
│ A2 │ -293.761 │ 8 │ 603.523 │
│ A3 │ -294.119 │ 5 │ 598.237 │
│ fitted │ -294.742 │ 4 │ 597.484 │
│ reduced │ -316.729 │ 2 │ 637.457 │
╘═════════╧══════════════════╧════════════╧═════════╛

Tests of Mean and Variance Fits:
╒════════╤══════════════════════════════╤═════════════╤═════════════╕
│ Name │ -2 * Log(Likelihood Ratio) │ Test d.f. │ P-Value │
╞════════╪══════════════════════════════╪═════════════╪═════════════╡
│ Test 1 │ 45.9346 │ 6 │ 3.05069e-08 │
│ Test 2 │ 0.714271 │ 3 │ 0.869842 │
│ Test 3 │ 0.714271 │ 3 │ 0.869842 │
│ Test 4 │ 1.2466 │ 1 │ 0.264202 │
╘════════╧══════════════════════════════╧═════════════╧═════════════╛
Test 1: Test the null hypothesis that responses and variances don't differ among dose levels
(A2 vs R). If this test fails to reject the null hypothesis (p-value > 0.05), there may not be
a dose-response.

Test 2: Test the null hypothesis that variances are homogenous (A1 vs A2). If this test fails to
reject the null hypothesis (p-value > 0.05), the simpler constant variance model may be appropriate.

Test 3: Test the null hypothesis that the variances are adequately modeled (A3 vs A2). If this test
fails to reject the null hypothesis (p-value > 0.05), it may be inferred that the variances have
been modeled appropriately.

Test 4: Test the null hypothesis that the model for the mean fits the data (Fitted vs A3). If this
test fails to reject the null hypothesis (p-value > 0.1), the user has support for use of the
selected model.

# Abs liver wt, Ms, F0, Dams, Premating-LD21, DuPont (2010)

## Dataset

**Name:** Abs liver wt, Ms, F0, Dams, Premating-LD21, DuPont (2010)

| Dose | N | Mean | Std. Dev. |
| --- | --- | --- | --- |
| 0 | 21 | 2.103 | 0.275 |
| 0.1 | 18 | 2.269 | 0.214 |
| 0.5 | 23 | 2.613 | 0.393 |
| 5 | 20 | 4.27 | 0.487 |

Test 1 Dose Response: 0

Test 2 Homogeneity of Variance: 0.0022

Test 3 Variance Model Selection: 0.1054

## Settings

| Setting | Value |
| --- | --- |
| BMR | 10% Relative Deviation |
| Distribution | Normal + Nonconstant variance |
| Adverse Direction | Up (↑) |
| Maximum Polynomial Degree | 3 |
| Confidence Level (one sided) | 0.95 |

## Maximum Likelihood Approach

| Model | BMDL | BMD | BMDU | *P*-Value | AIC | Scaled Residual at Control | Scaled Residual near BMD | Recommendation and Notes |
| --- | --- | --- | --- | --- | --- | --- | --- | --- |
| Exponential 3 | 0.686 | 0.746 | 0.916 | <0.001 | 76.221 | -2.308 | 2.737 | **Questionable** \|Residual near BMD\| > 2.0 Residual at control > 2.0 Goodness of fit p-value < 0.1 |
| Exponential 5 | 0.138 | 0.19 | 0.392 | 0.661 | 61.815 | -0.255 | 0.569 | **Viable** |
| Hill^ab^ | 0.128 | 0.183 | 0.361 | 0.7 | 61.771 | -0.22 | 0.526 | **Recommended - Lowest AIC** |
| Polynomial 2 | 0.48 | 0.539 | 0.668 | 0.002 | 71.981 | -1.985 | 2.404 | **Questionable** \|Residual near BMD\| > 2.0 Goodness of fit p-value < 0.1 |
| Polynomial 3 | 0.48 | 0.539 | 0.671 | 0.002 | 71.981 | -1.985 | 2.404 | **Questionable** \|Residual near BMD\| > 2.0 Goodness of fit p-value < 0.1 |
| Power | 0.48 | 0.539 | 0.661 | 0.002 | 71.981 | -1.985 | 2.404 | **Questionable** \|Residual near BMD\| > 2.0 Goodness of fit p-value < 0.1 |
| Linear | 0.48 | 0.539 | 0.609 | 0.002 | 71.981 | -1.985 | 2.404 | **Questionable** \|Residual near BMD\| > 2.0 Goodness of fit p-value < 0.1 |

^a^ BMDS recommended best fitting model

^b^ User selected best fitting model


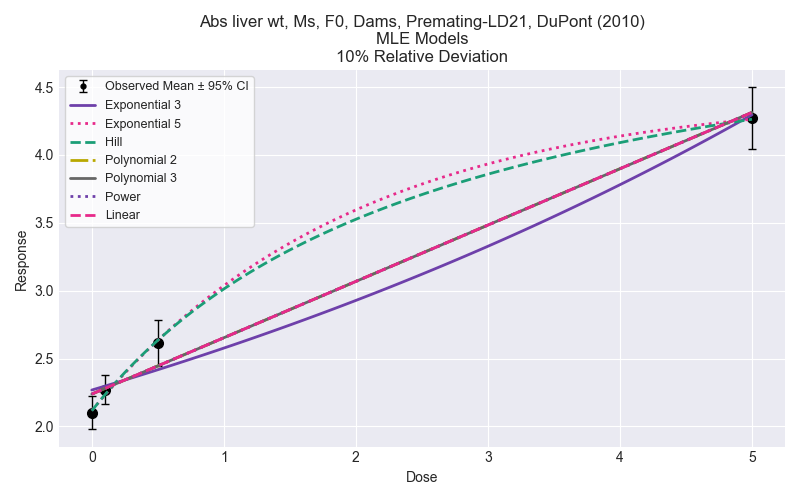


## Selected Model: Hill


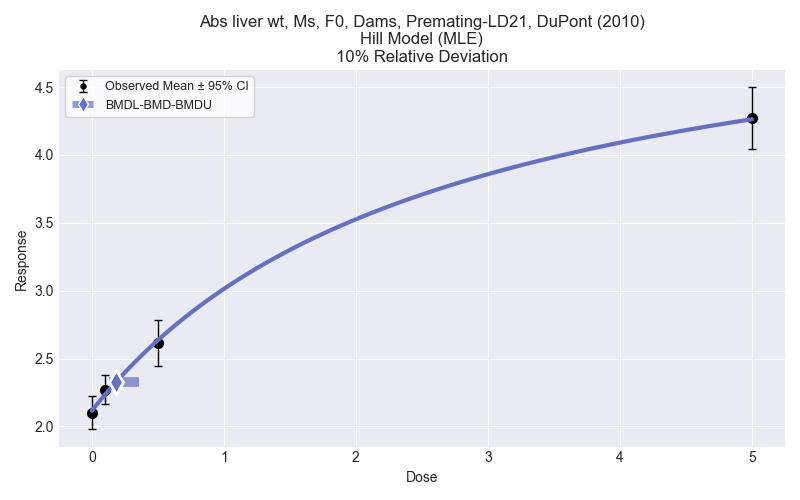


Hill Model
══════════════════════════════

Version: pybmds 25.1 (bmdscore 25.1)

Input Summary:
╒══════════════════════════════╤═══════════════════════════════╕
│ BMR │ 10% Relative Deviation │
│ Distribution │ Normal + Nonconstant variance │
│ Modeling Direction │ Up (↑) │
│ Confidence Level (one sided) │ 0.95 │
│ Modeling Approach │ MLE │
╘══════════════════════════════╧═══════════════════════════════╛

Parameter Settings:
╒═════════════╤═══════════╤═══════╤═══════╕
│ Parameter │ Initial │ Min │ Max │
╞═════════════╪═══════════╪═══════╪═══════╡
│ g │ 0 │ -100 │ 100 │
│ v │ 0 │ -100 │ 100 │
│ k │ 0 │ 0 │ 5 │
│ n │ 1 │ 1 │ 18 │
│ rho │ 0 │ -18 │ 18 │
│ alpha │ 0 │ -18 │ 18 │
╘═════════════╧═══════════╧═══════╧═══════╛

Modeling Summary:
╒════════════════╤════════════╕
│ BMD │ 0.183317 │
│ BMDL │ 0.128454 │
│ BMDU │ 0.36074 │
│ AIC │ 61.7707 │
│ Log-Likelihood │ -25.8853 │
│ P-Value │ 0.699928 │
│ Model d.f. │ 1 │
╘════════════════╧════════════╛

Model Parameters:
╒════════════╤════════════╤════════════╤══════════════╕
│ Variable │ Estimate │ On Bound │ Std Error │
╞════════════╪════════════╪════════════╪══════════════╡
│ g │ 2.11531 │ no │ 0.0490281 │
│ v │ 3.29506 │ no │ 0.437781 │
│ k │ 2.67226 │ no │ 0.884506 │
│ n │ 1 │ yes │ Not Reported │
│ rho │ 1.84357 │ no │ 0.621855 │
│ alpha │ 0.0176261 │ no │ 0.000198219 │
╘════════════╧════════════╧════════════╧══════════════╛
Standard errors estimates are not generated for parameters estimated on corresponding bounds,
although sampling error is present for all parameters, as a rule. Standard error estimates may not
be reliable as a basis for confidence intervals or tests when one or more parameters are on bounds.


Goodness of Fit:
╒════════╤═════╤═══════════════╤═════════════════════╤═══════════════════╕
│ Dose │ N │ Sample Mean │ Model Fitted Mean │ Scaled Residual │
╞════════╪═════╪═══════════════╪═════════════════════╪═══════════════════╡
│ 0 │ 21 │ 2.1026 │ 2.11531 │ -0.219853 │
│ 0.1 │ 18 │ 2.2687 │ 2.23416 │ 0.52603 │
│ 0.5 │ 23 │ 2.6128 │ 2.63466 │ -0.323342 │
│ 5 │ 20 │ 4.2703 │ 4.26269 │ 0.0673252 │
╘════════╧═════╧═══════════════╧═════════════════════╧═══════════════════╛
╒════════╤═════╤═════════════╤═══════════════════╕
│ Dose │ N │ Sample SD │ Model Fitted SD │
╞════════╪═════╪═════════════╪═══════════════════╡
│ 0 │ 21 │ 0.27466 │ 0.264852 │
│ 0.1 │ 18 │ 0.21414 │ 0.27854 │
│ 0.5 │ 23 │ 0.39338 │ 0.324262 │
│ 5 │ 20 │ 0.48662 │ 0.505256 │
╘════════╧═════╧═════════════╧═══════════════════╛

Likelihoods:
╒═════════╤══════════════════╤════════════╤══════════╕
│ Model │ Log-Likelihood │ # Params │ AIC │
╞═════════╪══════════════════╪════════════╪══════════╡
│ A1 │ -30.863 │ 5 │ 71.726 │
│ A2 │ -23.5612 │ 8 │ 63.1225 │
│ A3 │ -25.8111 │ 6 │ 63.6221 │
│ fitted │ -25.8853 │ 5 │ 61.7707 │
│ reduced │ -109.583 │ 2 │ 223.166 │
╘═════════╧══════════════════╧════════════╧══════════╛

Tests of Mean and Variance Fits:
╒════════╤══════════════════════════════╤═════════════╤════════════╕
│ Name │ -2 * Log(Likelihood Ratio) │ Test d.f. │ P-Value │
╞════════╪══════════════════════════════╪═════════════╪════════════╡
│ Test 1 │ 172.043 │ 6 │ 0 │
│ Test 2 │ 14.6035 │ 3 │ 0.00218887 │
│ Test 3 │ 4.49962 │ 2 │ 0.105419 │
│ Test 4 │ 0.148547 │ 1 │ 0.699928 │
╘════════╧══════════════════════════════╧═════════════╧════════════╛
Test 1: Test the null hypothesis that responses and variances don't differ among dose levels
(A2 vs R). If this test fails to reject the null hypothesis (p-value > 0.05), there may not be
a dose-response.

Test 2: Test the null hypothesis that variances are homogenous (A1 vs A2). If this test fails to
reject the null hypothesis (p-value > 0.05), the simpler constant variance model may be appropriate.

Test 3: Test the null hypothesis that the variances are adequately modeled (A3 vs A2). If this test
fails to reject the null hypothesis (p-value > 0.05), it may be inferred that the variances have
been modeled appropriately.

Test 4: Test the null hypothesis that the model for the mean fits the data (Fitted vs A3). If this
test fails to reject the null hypothesis (p-value > 0.1), the user has support for use of the
selected model.

# Rel liver wt (BW), Ms, F0, Dams, Premating-LD21, DuPont (2010)

## Dataset

**Name:** Rel liver wt (BW), Ms, F0, Dams, Premating-LD21, DuPont (2010)

| Dose | N | Mean | Std. Dev. |
| --- | --- | --- | --- |
| 0 | 21 | 6.006 | 0.546 |
| 0.1 | 18 | 6.456 | 0.407 |
| 0.5 | 23 | 7.05 | 0.803 |
| 5 | 20 | 10.767 | 1.068 |

Test 1 Dose Response: 0

Test 2 Homogeneity of Variance: 0.0838

Test 3 Variance Model Selection: 0.0838

## Settings

| Setting | Value |
| --- | --- |
| BMR | 10% Relative Deviation |
| Distribution | Lognormal + Constant variance |
| Adverse Direction | Up (↑) |
| Maximum Polynomial Degree | 3 |
| Confidence Level (one sided) | 0.95 |

## Maximum Likelihood Approach

| Model | BMDL | BMD | BMDU | *P*-Value | AIC | Scaled Residual at Control | Scaled Residual near BMD | Recommendation and Notes |
| --- | --- | --- | --- | --- | --- | --- | --- | --- |
| Exponential 3 | 0.822 | 0.891 | 1.079 | 0.001 | 188.14 | -1.397 | 1.552 | **Questionable** Goodness of fit p-value < 0.1 |
| Exponential 5^ab^ | 0.216 | 0.303 | 0.46 | 0.161 | 178.804 | -0.293 | 0.033 | **Recommended - Lowest AIC** |

^a^ BMDS recommended best fitting model

^b^ User selected best fitting model


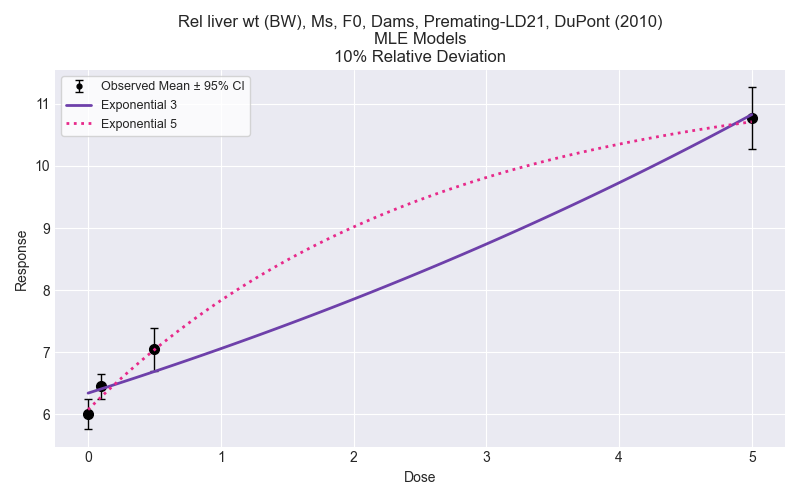


## Selected Model: Exponential 5


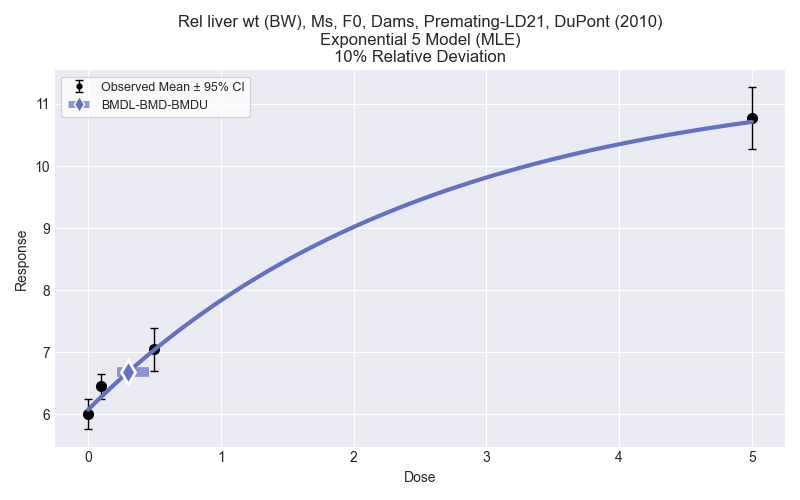


Exponential 5 Model
══════════════════════════════

Version: pybmds 25.1 (bmdscore 25.1)

Input Summary:
╒══════════════════════════════╤═══════════════════════════════╕
│ BMR │ 10% Relative Deviation │
│ Distribution │ Lognormal + Constant variance │
│ Modeling Direction │ Up (↑) │
│ Confidence Level (one sided) │ 0.95 │
│ Modeling Approach │ MLE │
╘══════════════════════════════╧═══════════════════════════════╛

Parameter Settings:
╒═════════════╤═══════════╤═══════╤═══════╕
│ Parameter │ Initial │ Min │ Max │
╞═════════════╪═══════════╪═══════╪═══════╡
│ a │ 0 │ 0 │ 100 │
│ b │ 0 │ 0 │ 100 │
│ c │ 0 │ 0 │ 20 │
│ d │ 1 │ 1 │ 18 │
│ log-alpha │ 0 │ -18 │ 18 │
╘═════════════╧═══════════╧═══════╧═══════╛

Modeling Summary:
╒════════════════╤════════════╕
│ BMD │ 0.302728 │
│ BMDL │ 0.216404 │
│ BMDU │ 0.460454 │
│ AIC │ 178.804 │
│ Log-Likelihood │ -85.4022 │
│ P-Value │ 0.161264 │
│ Model d.f. │ 1 │
╘════════════════╧════════════╛

Model Parameters:
╒════════════╤════════════╤════════════╤══════════════╕
│ Variable │ Estimate │ On Bound │ Std Error │
╞════════════╪════════════╪════════════╪══════════════╡
│ a │ 6.07605 │ no │ 0.103865 │
│ b │ 0.395967 │ no │ 0.122107 │
│ c │ 1.88523 │ no │ 0.0503862 │
│ d │ 1 │ yes │ Not Reported │
│ log-alpha │ -4.73781 │ no │ 0.156174 │
╘════════════╧════════════╧════════════╧══════════════╛
Standard errors estimates are not generated for parameters estimated on corresponding bounds,
although sampling error is present for all parameters, as a rule. Standard error estimates may not
be reliable as a basis for confidence intervals or tests when one or more parameters are on bounds.


Goodness of Fit:
╒════════╤═════╤═══════════════╤═════════════════════════════╤═══════════════════════╤═══════════════════╕
│ Dose │ N │ Sample Mean │ Approximate Sample Median │ Model Fitted Median │ Scaled Residual │
╞════════╪═════╪═══════════════╪═════════════════════════════╪═══════════════════════╪═══════════════════╡
│ 0 │ 21 │ 6.006 │ 5.98136 │ 6.07629 │ -0.293317 │
│ 0.1 │ 18 │ 6.456 │ 6.44323 │ 6.28511 │ 0.660243 │
│ 0.5 │ 23 │ 7.05 │ 7.00474 │ 7.04243 │ 0.0330524 │
│ 5 │ 20 │ 10.767 │ 10.7144 │ 10.7124 │ 0.222227 │
╘════════╧═════╧═══════════════╧═════════════════════════════╧═══════════════════════╧═══════════════════╛
╒════════╤═════╤═════════════╤══════════════════════════╤════════════════════╤═══════════════════╕
│ Dose │ N │ Sample SD │ Approximate Sample GSD │ Model Fitted GSD │ Scaled Residual │
╞════════╪═════╪═════════════╪══════════════════════════╪════════════════════╪═══════════════════╡
│ 0 │ 21 │ 0.5457 │ 1.09491 │ 1.0981 │ -0.293317 │
│ 0.1 │ 18 │ 0.4066 │ 1.06494 │ 1.0981 │ 0.660243 │
│ 0.5 │ 23 │ 0.8027 │ 1.12018 │ 1.0981 │ 0.0330524 │
│ 5 │ 20 │ 1.0681 │ 1.10402 │ 1.0981 │ 0.222227 │
╘════════╧═════╧═════════════╧══════════════════════════╧════════════════════╧═══════════════════╛

Likelihoods:
╒═════════╤══════════════════╤════════════╤═════════╕
│ Model │ Log-Likelihood │ # Params │ AIC │
╞═════════╪══════════════════╪════════════╪═════════╡
│ A1 │ -84.421 │ 5 │ 178.842 │
│ A2 │ -81.0947 │ 8 │ 178.189 │
│ A3 │ -84.421 │ 5 │ 178.842 │
│ fitted │ -85.4022 │ 4 │ 178.804 │
│ reduced │ -163.274 │ 2 │ 330.548 │
╘═════════╧══════════════════╧════════════╧═════════╛

Tests of Mean and Variance Fits:
╒════════╤══════════════════════════════╤═════════════╤═══════════╕
│ Name │ -2 * Log(Likelihood Ratio) │ Test d.f. │ P-Value │
╞════════╪══════════════════════════════╪═════════════╪═══════════╡
│ Test 1 │ 164.359 │ 6 │ 0 │
│ Test 2 │ 6.65267 │ 3 │ 0.0838323 │
│ Test 3 │ 6.65267 │ 3 │ 0.0838323 │
│ Test 4 │ 1.96234 │ 1 │ 0.161264 │
╘════════╧══════════════════════════════╧═════════════╧═══════════╛
Test 1: Test the null hypothesis that responses and variances don't differ among dose levels
(A2 vs R). If this test fails to reject the null hypothesis (p-value > 0.05), there may not be
a dose-response.

Test 2: Test the null hypothesis that variances are homogenous (A1 vs A2). If this test fails to
reject the null hypothesis (p-value > 0.05), the simpler constant variance model may be appropriate.

Test 3: Test the null hypothesis that the variances are adequately modeled (A3 vs A2). If this test
fails to reject the null hypothesis (p-value > 0.05), it may be inferred that the variances have
been modeled appropriately.

Test 4: Test the null hypothesis that the model for the mean fits the data (Fitted vs A3). If this
test fails to reject the null hypothesis (p-value > 0.1), the user has support for use of the
selected model.

# Rel liver wt (brain), Ms, F0, Dams, Premating-LD21, DuPont (2010)_drop

## Dataset

**Name:** Rel liver wt (brain), Ms, F0, Dams, Premating-LD21, DuPont (2010)_drop

| Dose | N | Mean | Std. Dev. |
| --- | --- | --- | --- |
| 0 | 21 | 427.097 | 51.199 |
| 0.1 | 18 | 455.91 | 46.886 |
| 0.5 | 23 | 526.053 | 94.229 |

Test 1 Dose Response: <0.0001

Test 2 Homogeneity of Variance: 0.0015

Test 3 Variance Model Selection: 0.2278

## Settings

| Setting | Value |
| --- | --- |
| BMR | 10% Relative Deviation |
| Distribution | Normal + Nonconstant variance |
| Adverse Direction | Up (↑) |
| Maximum Polynomial Degree | 2 |
| Confidence Level (one sided) | 0.95 |

## Maximum Likelihood Approach

| Model | BMDL | BMD | BMDU | *P*-Value | AIC | Scaled Residual at Control | Scaled Residual near BMD | Recommendation and Notes |
| --- | --- | --- | --- | --- | --- | --- | --- | --- |
| Exponential 3^ab^ | 0.167 | 0.216 | 0.483 | 0.532 | 694.513 | -0.126 | 0.609 | **Recommended - Lowest AIC** |
| Exponential 5 | 0.077 | 0.132 | 0.483 | - | 698.433 | 0.029 | 0.397 | **Questionable** Zero degrees of freedom; saturated model |
| Hill | 0.076 | 0.17 | 0.45 | - | 700.433 | 0.029 | 0.397 | **Questionable** Zero degrees of freedom; saturated model |
| Polynomial 2 | 0.15 | 0.201 | 0.375 | 0.271 | 696.459 | -0.055 | 0.519 | **Viable** |
| Power | 0.15 | 0.201 | 0.426 | 0.271 | 696.459 | -0.056 | 0.519 | **Viable** |
| Linear | 0.15 | 0.201 | - | 0.271 | 696.459 | -0.055 | 0.519 | **Viable** |

^a^ BMDS recommended best fitting model

^b^ User selected best fitting model


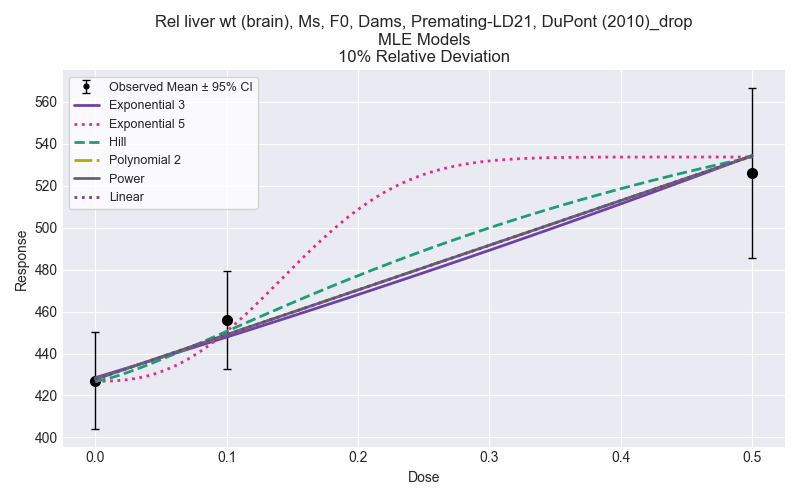


## Selected Model: Exponential 3


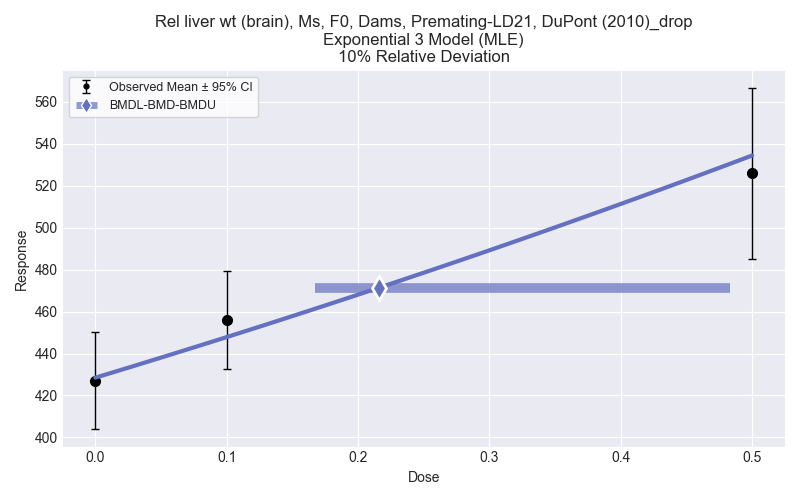


Exponential 3 Model
══════════════════════════════

Version: pybmds 25.1 (bmdscore 25.1)

Input Summary:
╒══════════════════════════════╤═══════════════════════════════╕
│ BMR │ 10% Relative Deviation │
│ Distribution │ Normal + Nonconstant variance │
│ Modeling Direction │ Up (↑) │
│ Confidence Level (one sided) │ 0.95 │
│ Modeling Approach │ MLE │
╘══════════════════════════════╧═══════════════════════════════╛

Parameter Settings:
╒═════════════╤═══════════╤═══════╤═══════╕
│ Parameter │ Initial │ Min │ Max │
╞═════════════╪═══════════╪═══════╪═══════╡
│ a │ 0 │ 0 │ 100 │
│ b │ 0 │ 0 │ 100 │
│ c │ 0 │ 0 │ 20 │
│ d │ 1 │ 1 │ 18 │
│ rho │ 0 │ 0 │ 18 │
│ log-alpha │ 0 │ -18 │ 18 │
╘═════════════╧═══════════╧═══════╧═══════╛

Modeling Summary:
╒════════════════╤═════════════╕
│ BMD │ 0.215702 │
│ BMDL │ 0.167102 │
│ BMDU │ 0.482819 │
│ AIC │ 694.513 │
│ Log-Likelihood │ -344.256 │
│ P-Value │ 0.531625 │
│ Model d.f. │ 2 │
╘════════════════╧═════════════╛

Model Parameters:
╒════════════╤════════════╤════════════╤══════════════╕
│ Variable │ Estimate │ On Bound │ Std Error │
╞════════════╪════════════╪════════════╪══════════════╡
│ a │ 428.495 │ no │ 8.85242 │
│ b │ 0.441861 │ no │ 0.0753804 │
│ d │ 1 │ yes │ Not Reported │
│ rho │ 4.26807 │ no │ 0.0314443 │
│ log-alpha │ -18 │ yes │ Not Reported │
╘════════════╧════════════╧════════════╧══════════════╛
Standard errors estimates are not generated for parameters estimated on corresponding bounds,
although sampling error is present for all parameters, as a rule. Standard error estimates may not
be reliable as a basis for confidence intervals or tests when one or more parameters are on bounds.


Goodness of Fit:
╒════════╤═════╤═══════════════╤═════════════════════╤═══════════════════╕
│ Dose │ N │ Sample Mean │ Model Fitted Mean │ Scaled Residual │
╞════════╪═════╪═══════════════╪═════════════════════╪═══════════════════╡
│ 0 │ 21 │ 427.097 │ 428.495 │ -0.125523 │
│ 0.1 │ 18 │ 455.91 │ 447.853 │ 0.609274 │
│ 0.5 │ 23 │ 526.053 │ 534.435 │ -0.491412 │
╘════════╧═════╧═══════════════╧═════════════════════╧═══════════════════╛
╒════════╤═════╤═════════════╤═══════════════════╕
│ Dose │ N │ Sample SD │ Model Fitted SD │
╞════════╪═════╪═════════════╪═══════════════════╡
│ 0 │ 21 │ 51.1989 │ 51.0528 │
│ 0.1 │ 18 │ 46.8862 │ 56.101 │
│ 0.5 │ 23 │ 94.2293 │ 81.8046 │
╘════════╧═════╧═════════════╧═══════════════════╛

Likelihoods:
╒═════════╤══════════════════╤════════════╤═════════╕
│ Model │ Log-Likelihood │ # Params │ AIC │
╞═════════╪══════════════════╪════════════╪═════════╡
│ A1 │ -349.416 │ 4 │ 706.832 │
│ A2 │ -342.897 │ 6 │ 697.794 │
│ A3 │ -343.625 │ 5 │ 697.249 │
│ fitted │ -344.256 │ 3 │ 694.513 │
│ reduced │ -359.858 │ 2 │ 723.715 │
╘═════════╧══════════════════╧════════════╧═════════╛

Tests of Mean and Variance Fits:
╒════════╤══════════════════════════════╤═════════════╤═════════════╕
│ Name │ -2 * Log(Likelihood Ratio) │ Test d.f. │ P-Value │
╞════════╪══════════════════════════════╪═════════════╪═════════════╡
│ Test 1 │ 33.9208 │ 4 │ 7.73588e-07 │
│ Test 2 │ 13.0372 │ 2 │ 0.00147577 │
│ Test 3 │ 1.45483 │ 1 │ 0.227754 │
│ Test 4 │ 1.26363 │ 2 │ 0.531625 │
╘════════╧══════════════════════════════╧═════════════╧═════════════╛
Test 1: Test the null hypothesis that responses and variances don't differ among dose levels
(A2 vs R). If this test fails to reject the null hypothesis (p-value > 0.05), there may not be
a dose-response.

Test 2: Test the null hypothesis that variances are homogenous (A1 vs A2). If this test fails to
reject the null hypothesis (p-value > 0.05), the simpler constant variance model may be appropriate.

Test 3: Test the null hypothesis that the variances are adequately modeled (A3 vs A2). If this test
fails to reject the null hypothesis (p-value > 0.05), it may be inferred that the variances have
been modeled appropriately.

Test 4: Test the null hypothesis that the model for the mean fits the data (Fitted vs A3). If this
test fails to reject the null hypothesis (p-value > 0.1), the user has support for use of the
selected model.

# premating BW change, Ms, F0, Dams, DuPont (2010)

## Dataset

**Name:** premating BW change, Ms, F0, Dams, DuPont (2010)

| Dose | N | Mean | Std. Dev. |
| --- | --- | --- | --- |
| 0 | 25 | 1 | 0.81 |
| 0.1 | 25 | 1.4 | 0.85 |
| 0.5 | 25 | 2 | 0.84 |

Test 1 Dose Response: 0.0019

Test 2 Homogeneity of Variance: 0.9692

Test 3 Variance Model Selection: 0.9692

## Settings

| Setting | Value |
| --- | --- |
| BMR | 10% Relative Deviation |
| Distribution | Normal + Constant variance |
| Adverse Direction | Up (↑) |
| Maximum Polynomial Degree | 2 |
| Confidence Level (one sided) | 0.95 |

## Maximum Likelihood Approach

| Model | BMDL | BMD | BMDU | *P*-Value | AIC | Scaled Residual at Control | Scaled Residual near BMD | Recommendation and Notes |
| --- | --- | --- | --- | --- | --- | --- | --- | --- |
| Exponential 3 | 0.058 | 0.081 | 0.466 | 0.259 | 189.737 | -0.749 | 0.833 | **Viable** |
| Exponential 5 | 0.006 | 0.028 | 0.227 | - | 192.462 | <0.001 | <0.001 | **Questionable** lowest dose/BMDL ratio > 3.0 lowest dose/BMDL ratio > 10.0 lowest dose/BMD ratio > 3.0 Zero degrees of freedom; saturated model BMD/BMDL ratio > 3.0 |
| Hill | 0.004 | 0.039 | 0.225 | - | 192.462 | <0.001 | <0.001 | **Questionable** lowest dose/BMDL ratio > 3.0 lowest dose/BMDL ratio > 10.0 Zero degrees of freedom; saturated model BMD/BMDL ratio > 3.0 |
| Polynomial 2 | 0.036 | 0.059 | 0.228 | 0.346 | 189.349 | -0.58 | 0.725 | **Viable** |
| Power | 0.036 | 0.059 | 0.456 | 0.346 | 189.349 | -0.58 | 0.725 | **Viable** |
| Linear^ab^ | 0.036 | 0.059 | 0.111 | 0.346 | 189.349 | -0.58 | 0.725 | **Recommended - Lowest AIC** |

^a^ BMDS recommended best fitting model

^b^ User selected best fitting model


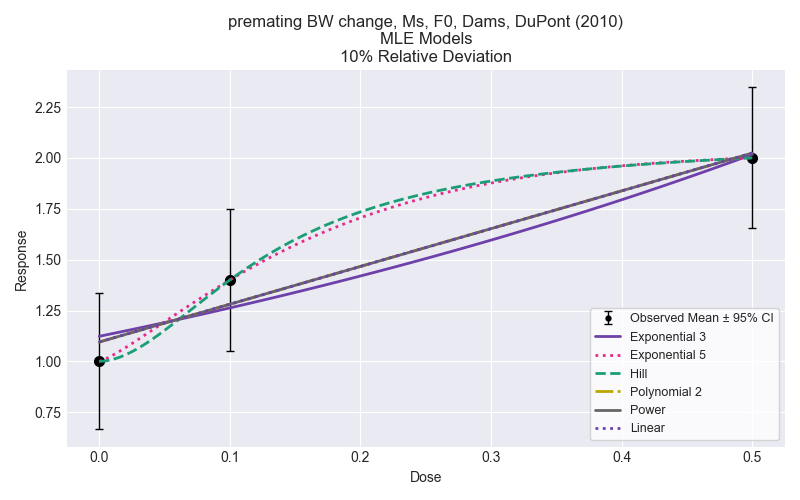


## Selected Model: Linear


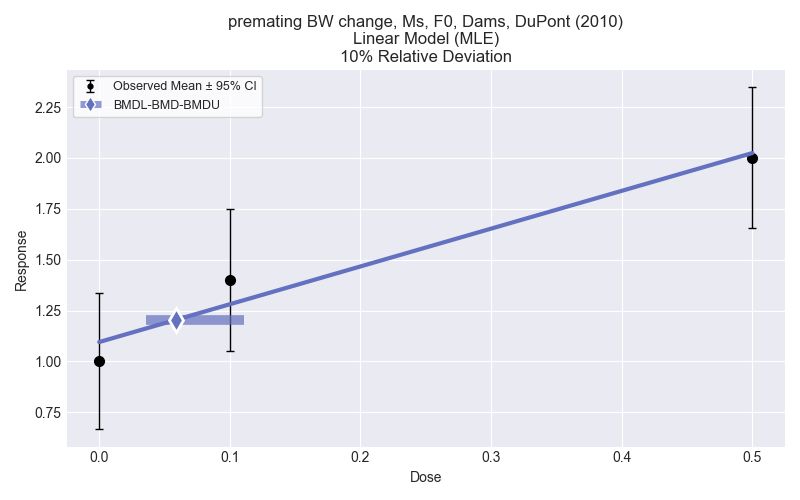


Linear Model
══════════════════════════════

Version: pybmds 25.1 (bmdscore 25.1)

Input Summary:
╒══════════════════════════════╤════════════════════════════╕
│ BMR │ 10% Relative Deviation │
│ Distribution │ Normal + Constant variance │
│ Modeling Direction │ Up (↑) │
│ Confidence Level (one sided) │ 0.95 │
│ Modeling Approach │ MLE │
│ Degree │ 1 │
╘══════════════════════════════╧════════════════════════════╛

Parameter Settings:
╒═════════════╤═══════════╤═════════╤════════╕
│ Parameter │ Initial │ Min │ Max │
╞═════════════╪═══════════╪═════════╪════════╡
│ g │ 0 │ -1e+06 │ 1e+06 │
│ b1 │ 0 │ -1e+06 │ 1e+06 │
│ alpha │ 0 │ -18 │ 18 │
╘═════════════╧═══════════╧═════════╧════════╛

Modeling Summary:
╒════════════════╤═════════════╕
│ BMD │ 0.0589744 │
│ BMDL │ 0.0355738 │
│ BMDU │ 0.111133 │
│ AIC │ 189.349 │
│ Log-Likelihood │ -91.6747 │
│ P-Value │ 0.346232 │
│ Model d.f. │ 1 │
╘════════════════╧═════════════╛

Model Parameters:
╒════════════╤════════════╤════════════╤═════════════╕
│ Variable │ Estimate │ On Bound │ Std Error │
╞════════════╪════════════╪════════════╪═════════════╡
│ g │ 1.09524 │ no │ 0.129272 │
│ b1 │ 1.85714 │ no │ 0.439114 │
│ alpha │ 0.674881 │ no │ 0.0743702 │
╘════════════╧════════════╧════════════╧═════════════╛

Goodness of Fit:
╒════════╤═════╤═══════════════╤═════════════════════╤═══════════════════╕
│ Dose │ N │ Sample Mean │ Model Fitted Mean │ Scaled Residual │
╞════════╪═════╪═══════════════╪═════════════════════╪═══════════════════╡
│ 0 │ 25 │ 1 │ 1.09524 │ -0.579652 │
│ 0.1 │ 25 │ 1.4 │ 1.28095 │ 0.724565 │
│ 0.5 │ 25 │ 2 │ 2.02381 │ -0.144913 │
╘════════╧═════╧═══════════════╧═════════════════════╧═══════════════════╛
╒════════╤═════╤═════════════╤═══════════════════╕
│ Dose │ N │ Sample SD │ Model Fitted SD │
╞════════╪═════╪═════════════╪═══════════════════╡
│ 0 │ 25 │ 0.81 │ 0.821511 │
│ 0.1 │ 25 │ 0.85 │ 0.821511 │
│ 0.5 │ 25 │ 0.84 │ 0.821511 │
╘════════╧═════╧═════════════╧═══════════════════╛

Likelihoods:
╒═════════╤══════════════════╤════════════╤═════════╕
│ Model │ Log-Likelihood │ # Params │ AIC │
╞═════════╪══════════════════╪════════════╪═════════╡
│ A1 │ -91.231 │ 4 │ 190.462 │
│ A2 │ -91.1997 │ 6 │ 194.399 │
│ A3 │ -91.231 │ 4 │ 190.462 │
│ fitted │ -91.6747 │ 3 │ 189.349 │
│ reduced │ -99.6956 │ 2 │ 203.391 │
╘═════════╧══════════════════╧════════════╧═════════╛

Tests of Mean and Variance Fits:
╒════════╤══════════════════════════════╤═════════════╤════════════╕
│ Name │ -2 * Log(Likelihood Ratio) │ Test d.f. │ P-Value │
╞════════╪══════════════════════════════╪═════════════╪════════════╡
│ Test 1 │ 16.9918 │ 4 │ 0.00194003 │
│ Test 2 │ 0.0626274 │ 2 │ 0.969172 │
│ Test 3 │ 0.0626274 │ 2 │ 0.969172 │
│ Test 4 │ 0.887217 │ 1 │ 0.346232 │
╘════════╧══════════════════════════════╧═════════════╧════════════╛
Test 1: Test the null hypothesis that responses and variances don't differ among dose levels
(A2 vs R). If this test fails to reject the null hypothesis (p-value > 0.05), there may not be
a dose-response.

Test 2: Test the null hypothesis that variances are homogenous (A1 vs A2). If this test fails to
reject the null hypothesis (p-value > 0.05), the simpler constant variance model may be appropriate.

Test 3: Test the null hypothesis that the variances are adequately modeled (A3 vs A2). If this test
fails to reject the null hypothesis (p-value > 0.05), it may be inferred that the variances have
been modeled appropriately.

Test 4: Test the null hypothesis that the model for the mean fits the data (Fitted vs A3). If this
test fails to reject the null hypothesis (p-value > 0.1), the user has support for use of the
selected model.

# lactational BW, Ms, F0, Dams, LD21, DuPont (2010)

## Dataset

**Name:** lactational BW, Ms, F0, Dams, LD21, DuPont (2010)

| Dose | N | Mean | Std. Dev. |
| --- | --- | --- | --- |
| 0 | 21 | 34.9 | 2.24 |
| 0.1 | 18 | 35.2 | 2.57 |
| 0.5 | 23 | 37 | 3.11 |
| 5 | 20 | 39.8 | 3.9 |

Test 1 Dose Response: <0.0001

Test 2 Homogeneity of Variance: 0.068

Test 3 Variance Model Selection: 0.068

## Settings

| Setting | Value |
| --- | --- |
| BMR | 10% Relative Deviation |
| Distribution | Normal + Constant variance |
| Adverse Direction | Up (↑) |
| Maximum Polynomial Degree | 3 |
| Confidence Level (one sided) | 0.95 |

## Maximum Likelihood Approach

| Model | BMDL | BMD | BMDU | *P*-Value | AIC | Scaled Residual at Control | Scaled Residual near BMD | Recommendation and Notes |
| --- | --- | --- | --- | --- | --- | --- | --- | --- |
| Exponential 3 | 3.184 | 4.136 | 5.935 | 0.125 | 420.374 | -0.995 | -0.147 | **Viable** |
| Exponential 5 | 0.462 | 0.902 | 3.898 | - | 420.215 | <0.001 | <0.001 | **Questionable** Zero degrees of freedom; saturated model |
| Hill | 3.598 | 3.863 | 3.943 | 0.055 | 421.906 | -0.915 | -0.183 | **Questionable** Goodness of fit p-value < 0.1 |
| Polynomial 2 | 3.079 | 4.072 | 5.942 | 0.132 | 420.268 | -0.975 | -0.161 | **Viable** |
| Polynomial 3 | 2.869 | 4.656 | 4.753 | 0.016 | 424.054 | -1.243 | -0.036 | **Questionable** Goodness of fit p-value < 0.1 |
| Power | 3.079 | 4.07 | 5.942 | 0.132 | 420.268 | -0.975 | -0.162 | **Viable** |
| Linear^ab^ | 3.079 | 4.07 | 5.942 | 0.132 | 420.268 | -0.975 | -0.162 | **Recommended - Lowest AIC** |

^a^ BMDS recommended best fitting model

^b^ User selected best fitting model


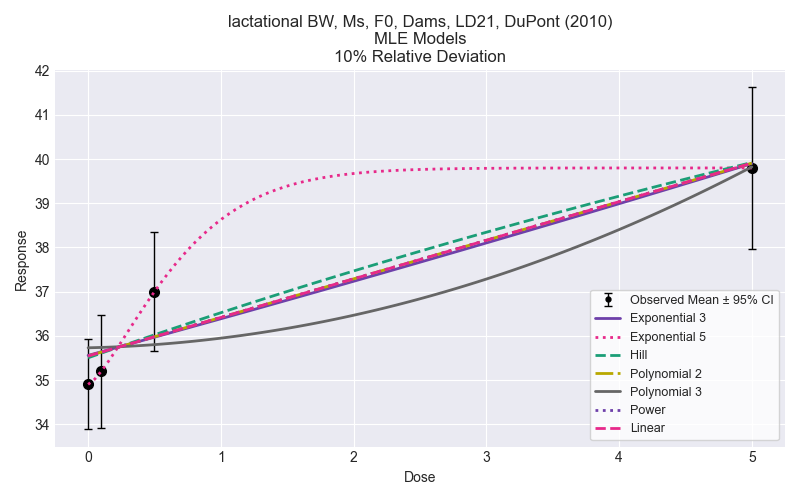


## Selected Model: Linear


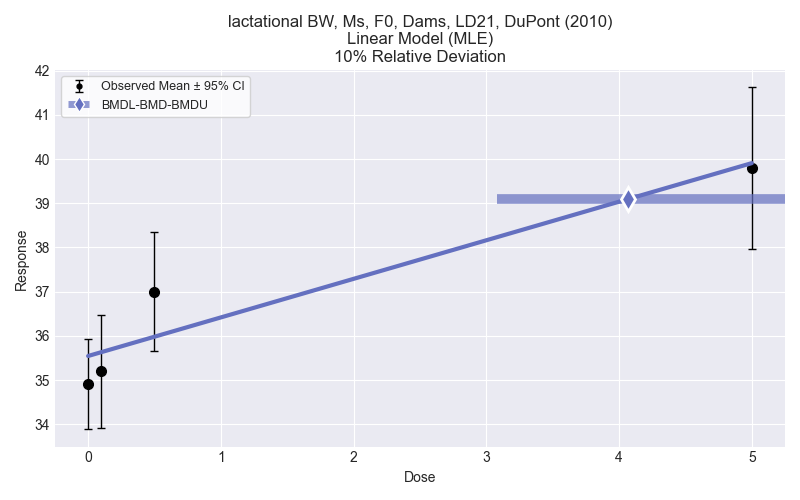


Linear Model
══════════════════════════════

Version: pybmds 25.1 (bmdscore 25.1)

Input Summary:
╒══════════════════════════════╤════════════════════════════╕
│ BMR │ 10% Relative Deviation │
│ Distribution │ Normal + Constant variance │
│ Modeling Direction │ Up (↑) │
│ Confidence Level (one sided) │ 0.95 │
│ Modeling Approach │ MLE │
│ Degree │ 1 │
╘══════════════════════════════╧════════════════════════════╛

Parameter Settings:
╒═════════════╤═══════════╤═════════╤════════╕
│ Parameter │ Initial │ Min │ Max │
╞═════════════╪═══════════╪═════════╪════════╡
│ g │ 0 │ -1e+06 │ 1e+06 │
│ b1 │ 0 │ -1e+06 │ 1e+06 │
│ alpha │ 0 │ -18 │ 18 │
╘═════════════╧═══════════╧═════════╧════════╛

Modeling Summary:
╒════════════════╤═════════════╕
│ BMD │ 4.07043 │
│ BMDL │ 3.07919 │
│ BMDU │ 5.94157 │
│ AIC │ 420.268 │
│ Log-Likelihood │ -207.134 │
│ P-Value │ 0.131776 │
│ Model d.f. │ 2 │
╘════════════════╧═════════════╛

Model Parameters:
╒════════════╤════════════╤════════════╤═════════════╕
│ Variable │ Estimate │ On Bound │ Std Error │
╞════════════╪════════════╪════════════╪═════════════╡
│ g │ 35.5435 │ no │ 0.402052 │
│ b1 │ 0.873212 │ no │ 0.161856 │
│ alpha │ 9.15381 │ no │ 13.0861 │
╘════════════╧════════════╧════════════╧═════════════╛

Goodness of Fit:
╒════════╤═════╤═══════════════╤═════════════════════╤═══════════════════╕
│ Dose │ N │ Sample Mean │ Model Fitted Mean │ Scaled Residual │
╞════════╪═════╪═══════════════╪═════════════════════╪═══════════════════╡
│ 0 │ 21 │ 34.9 │ 35.5435 │ -0.974634 │
│ 0.1 │ 18 │ 35.2 │ 35.6308 │ -0.6041 │
│ 0.5 │ 23 │ 37 │ 35.9801 │ 1.61669 │
│ 5 │ 20 │ 39.8 │ 39.9095 │ -0.161909 │
╘════════╧═════╧═══════════════╧═════════════════════╧═══════════════════╛
╒════════╤═════╤═════════════╤═══════════════════╕
│ Dose │ N │ Sample SD │ Model Fitted SD │
╞════════╪═════╪═════════════╪═══════════════════╡
│ 0 │ 21 │ 2.24 │ 3.02553 │
│ 0.1 │ 18 │ 2.57 │ 3.02553 │
│ 0.5 │ 23 │ 3.11 │ 3.02553 │
│ 5 │ 20 │ 3.9 │ 3.02553 │
╘════════╧═════╧═════════════╧═══════════════════╛

Likelihoods:
╒═════════╤══════════════════╤════════════╤═════════╕
│ Model │ Log-Likelihood │ # Params │ AIC │
╞═════════╪══════════════════╪════════════╪═════════╡
│ A1 │ -205.107 │ 5 │ 420.215 │
│ A2 │ -201.544 │ 8 │ 419.089 │
│ A3 │ -205.107 │ 5 │ 420.215 │
│ fitted │ -207.134 │ 3 │ 420.268 │
│ reduced │ -219.587 │ 2 │ 443.174 │
╘═════════╧══════════════════╧════════════╧═════════╛

Tests of Mean and Variance Fits:
╒════════╤══════════════════════════════╤═════════════╤═════════════╕
│ Name │ -2 * Log(Likelihood Ratio) │ Test d.f. │ P-Value │
╞════════╪══════════════════════════════╪═════════════╪═════════════╡
│ Test 1 │ 36.086 │ 6 │ 2.65258e-06 │
│ Test 2 │ 7.12607 │ 3 │ 0.0679864 │
│ Test 3 │ 7.12607 │ 3 │ 0.0679864 │
│ Test 4 │ 4.05331 │ 2 │ 0.131776 │
╘════════╧══════════════════════════════╧═════════════╧═════════════╛
Test 1: Test the null hypothesis that responses and variances don't differ among dose levels
(A2 vs R). If this test fails to reject the null hypothesis (p-value > 0.05), there may not be
a dose-response.

Test 2: Test the null hypothesis that variances are homogenous (A1 vs A2). If this test fails to
reject the null hypothesis (p-value > 0.05), the simpler constant variance model may be appropriate.

Test 3: Test the null hypothesis that the variances are adequately modeled (A3 vs A2). If this test
fails to reject the null hypothesis (p-value > 0.05), it may be inferred that the variances have
been modeled appropriately.

Test 4: Test the null hypothesis that the model for the mean fits the data (Fitted vs A3). If this
test fails to reject the null hypothesis (p-value > 0.1), the user has support for use of the
selected model.

# Serum Triglyceride, Ms., M Offspring, Cope et al. (2021)

## Dataset

**Name:** Serum Triglyceride, Ms., M Offspring, Cope et al. (2021)

| Dose (mg/kg-day) | N | Mean | Std. Dev. |
| --- | --- | --- | --- |
| 0 | 7 | 174.6 | 67.2 |
| 0.2 | 7 | 124 | 38 |
| 1 | 7 | 114.9 | 43.5 |
| 2 | 7 | 106.6 | 27.6 |

Test 1 Dose Response: 0.0219

Test 2 Homogeneity of Variance: 0.1221

Test 3 Variance Model Selection: 0.1221

## Settings

| Setting | Value |
| --- | --- |
| BMR | 1.0 Standard Deviation |
| Distribution | Normal + Constant variance |
| Adverse Direction | Down (↓) |
| Maximum Polynomial Degree | 3 |
| Confidence Level (one sided) | 0.95 |

## Maximum Likelihood Approach

| Model | BMDL | BMD | BMDU | *P*-Value | AIC | Scaled Residual at Control | Scaled Residual near BMD | Recommendation and Notes |
| --- | --- | --- | --- | --- | --- | --- | --- | --- |
| Exponential 3^ab^ | 0.806 | 1.645 | 7.27 | 0.144 | 299.914 | 1.268 | 0.463 | **Recommended - Lowest AIC** |
| Exponential 5 | 0.013 | 0.143 | - | 0.719 | 298.169 | <0.001 | -0.002 | **Questionable** lowest dose/BMDL ratio > 3.0 lowest dose/BMDL ratio > 10.0 BMD/BMDL ratio > 3.0 |
| Hill | <0.001 | 0.123 | - | 0.786 | 298.114 | 0.002 | -0.037 | **Questionable** lowest dose/BMDL ratio > 3.0 lowest dose/BMDL ratio > 10.0 BMD/BMDL ratio > 3.0 BMD/BMDL ratio > 20.0 |
| Polynomial 2 | 1.038 | 1.821 | 7.26 | 0.123 | 300.228 | 1.385 | 0.405 | **Viable** |
| Polynomial 3 | 1.038 | 1.799 | 7.263 | 0.123 | 300.228 | 1.37 | 0.426 | **Viable** |
| Power | 1.038 | 1.817 | 7.261 | 0.123 | 300.228 | 1.383 | 0.408 | **Viable** |
| Linear | 1.038 | 1.817 | 7.261 | 0.123 | 300.228 | 1.383 | 0.408 | **Viable** |

^a^ BMDS recommended best fitting model

^b^ User selected best fitting model


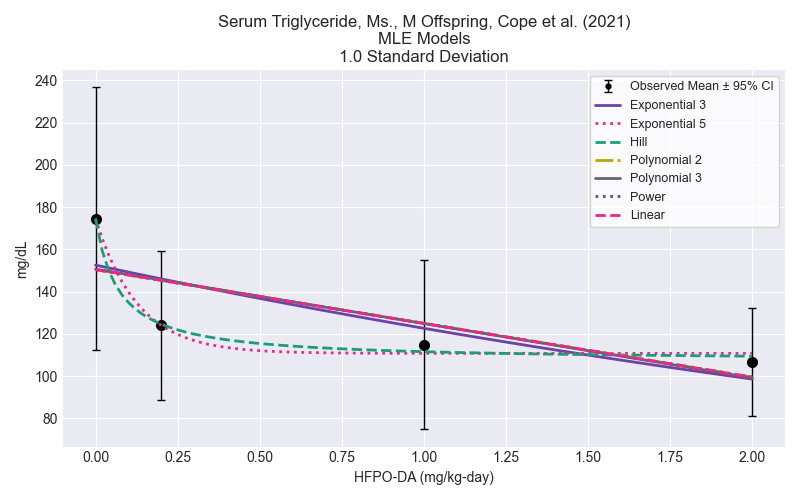


## Selected Model: Exponential 3


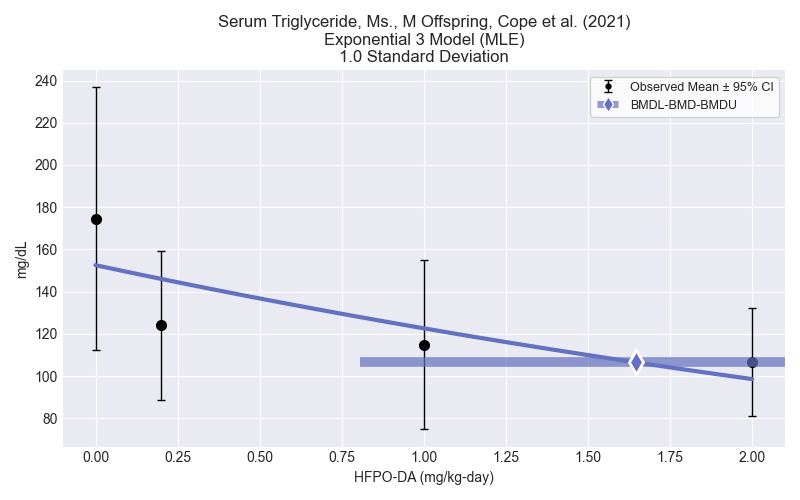


Exponential 3 Model
══════════════════════════════

Version: pybmds 25.1 (bmdscore 25.1)

Input Summary:
╒══════════════════════════════╤════════════════════════════╕
│ BMR │ 1.0 Standard Deviation │
│ Distribution │ Normal + Constant variance │
│ Modeling Direction │ Down (↓) │
│ Confidence Level (one sided) │ 0.95 │
│ Modeling Approach │ MLE │
╘══════════════════════════════╧════════════════════════════╛

Parameter Settings:
╒═════════════╤═══════════╤═══════╤═══════╕
│ Parameter │ Initial │ Min │ Max │
╞═════════════╪═══════════╪═══════╪═══════╡
│ a │ 0 │ 0 │ 100 │
│ b │ 0 │ 0 │ 100 │
│ c │ 0 │ -20 │ 0 │
│ d │ 1 │ 1 │ 18 │
│ log-alpha │ 0 │ -18 │ 18 │
╘═════════════╧═══════════╧═══════╧═══════╛

Modeling Summary:
╒════════════════╤═════════════╕
│ BMD │ 1.64481 │
│ BMDL │ 0.806237 │
│ BMDU │ 7.27 │
│ AIC │ 299.914 │
│ Log-Likelihood │ -146.957 │
│ P-Value │ 0.1441 │
│ Model d.f. │ 2 │
╘════════════════╧═════════════╛

Model Parameters:
╒════════════╤════════════╤════════════╤══════════════╕
│ Variable │ Estimate │ On Bound │ Std Error │
╞════════════╪════════════╪════════════╪══════════════╡
│ a │ 152.537 │ no │ 13.1331 │
│ b │ 0.218444 │ no │ 0.0988134 │
│ d │ 1 │ yes │ Not Reported │
│ log-alpha │ 7.65906 │ no │ 0.267261 │
╘════════════╧════════════╧════════════╧══════════════╛
Standard errors estimates are not generated for parameters estimated on corresponding bounds,
although sampling error is present for all parameters, as a rule. Standard error estimates may not
be reliable as a basis for confidence intervals or tests when one or more parameters are on bounds.


Goodness of Fit:
╒════════╤═════╤═══════════════╤═════════════════════╤═══════════════════╕
│ Dose │ N │ Sample Mean │ Model Fitted Mean │ Scaled Residual │
╞════════╪═════╪═══════════════╪═════════════════════╪═══════════════════╡
│ 0 │ 7 │ 174.6 │ 152.537 │ 1.26788 │
│ 0.2 │ 7 │ 124 │ 146.016 │ -1.26515 │
│ 1 │ 7 │ 114.9 │ 122.604 │ -0.44272 │
│ 2 │ 7 │ 106.6 │ 98.5454 │ 0.462862 │
╘════════╧═════╧═══════════════╧═════════════════════╧═══════════════════╛
╒════════╤═════╤═════════════╤═══════════════════╕
│ Dose │ N │ Sample SD │ Model Fitted SD │
╞════════╪═════╪═════════════╪═══════════════════╡
│ 0 │ 7 │ 67.2 │ 46.0408 │
│ 0.2 │ 7 │ 38 │ 46.0408 │
│ 1 │ 7 │ 43.5 │ 46.0408 │
│ 2 │ 7 │ 27.6 │ 46.0408 │
╘════════╧═════╧═════════════╧═══════════════════╛

Likelihoods:
╒═════════╤══════════════════╤════════════╤═════════╕
│ Model │ Log-Likelihood │ # Params │ AIC │
╞═════════╪══════════════════╪════════════╪═════════╡
│ A1 │ -145.02 │ 5 │ 300.04 │
│ A2 │ -142.123 │ 8 │ 300.246 │
│ A3 │ -145.02 │ 5 │ 300.04 │
│ fitted │ -146.957 │ 3 │ 299.914 │
│ reduced │ -149.522 │ 2 │ 303.044 │
╘═════════╧══════════════════╧════════════╧═════════╛

Tests of Mean and Variance Fits:
╒════════╤══════════════════════════════╤═════════════╤═══════════╕
│ Name │ -2 * Log(Likelihood Ratio) │ Test d.f. │ P-Value │
╞════════╪══════════════════════════════╪═════════════╪═══════════╡
│ Test 1 │ 14.7984 │ 6 │ 0.0218841 │
│ Test 2 │ 5.7936 │ 3 │ 0.122095 │
│ Test 3 │ 5.7936 │ 3 │ 0.122095 │
│ Test 4 │ 3.8745 │ 2 │ 0.1441 │
╘════════╧══════════════════════════════╧═════════════╧═══════════╛
Test 1: Test the null hypothesis that responses and variances don't differ among dose levels
(A2 vs R). If this test fails to reject the null hypothesis (p-value > 0.05), there may not be
a dose-response.

Test 2: Test the null hypothesis that variances are homogenous (A1 vs A2). If this test fails to
reject the null hypothesis (p-value > 0.05), the simpler constant variance model may be appropriate.

Test 3: Test the null hypothesis that the variances are adequately modeled (A3 vs A2). If this test
fails to reject the null hypothesis (p-value > 0.05), it may be inferred that the variances have
been modeled appropriately.

Test 4: Test the null hypothesis that the model for the mean fits the data (Fitted vs A3). If this
test fails to reject the null hypothesis (p-value > 0.1), the user has support for use of the
selected model.

## Serum Triglyceride, Ms., M Offspring, Cope et al. (2021)

## Dataset

**Name:** Serum Triglyceride, Ms., M Offspring, Cope et al. (2021)

| Dose (mg/kg-day) | N | Mean | Std. Dev. |
| --- | --- | --- | --- |
| 0 | 7 | 174.6 | 67.2 |
| 0.2 | 7 | 124 | 38 |
| 1 | 7 | 114.9 | 43.5 |
| 2 | 7 | 106.6 | 27.6 |

## Settings

| Setting | Value |
| --- | --- |
| BMR | 1.0 Standard Deviation |
| Distribution | Normal + Nonconstant variance |
| Adverse Direction | Down (↓) |
| Maximum Polynomial Degree | 3 |
| Confidence Level (one sided) | 0.95 |

## Maximum Likelihood Approach

| Model | BMDL | BMD | BMDU | *P*-Value | AIC | Scaled Residual at Control | Scaled Residual near BMD | Recommendation and Notes |
| --- | --- | --- | --- | --- | --- | --- | --- | --- |
| Exponential 3 | - | - | - | - | - | 0 | - | **Unusable** Did not successfully execute. |
| Exponential 5 | 0.018 | 0.301 | 0.331 | - | 297.959 | -0.031 | 0.135 | **Questionable** lowest dose/BMDL ratio > 3.0 lowest dose/BMDL ratio > 10.0 Zero degrees of freedom; saturated model BMD/BMDL ratio > 3.0 |
| Hill | <0.001 | 0.682 | - | 0.348 | 295.749 | -0.016 | 0.259 | **Questionable** lowest dose/BMDL ratio > 3.0 lowest dose/BMDL ratio > 10.0 BMD/BMDL ratio > 3.0 BMD/BMDL ratio > 20.0 |
| Polynomial 2^a^ | 1.445 | 2.807 | 5.278 | 0.11 | 297.279 | 1.316 | 0.249 | **Recommended - Lowest AIC** BMD/highest dose ratio > 1.0 |
| Polynomial 3 | 1.445 | 2.866 | 5.28 | 0.11 | 297.281 | 1.333 | 0.224 | **Viable** BMD/highest dose ratio > 1.0 |
| Power | 1.445 | 2.734 | 2.79 | 0.11 | 297.283 | 1.3 | 0.28 | **Viable** BMD/highest dose ratio > 1.0 |
| Linear | 1.445 | 2.807 | 5.278 | 0.11 | 297.279 | 1.316 | 0.249 | **Viable** BMD/highest dose ratio > 1.0 |

^a^ BMDS recommended best fitting model

## Selected Model

No model was selected as a best-fitting model.

## Serum Triglyceride, Ms., M Offspring, Cope et al. (2021)

## Dataset

**Name:** Serum Triglyceride, Ms., M Offspring, Cope et al. (2021)

| Dose (mg/kg-day) | N | Mean | Std. Dev. |
| --- | --- | --- | --- |
| 0 | 7 | 174.6 | 67.2 |
| 0.2 | 7 | 124 | 38 |
| 1 | 7 | 114.9 | 43.5 |
| 2 | 7 | 106.6 | 27.6 |

Test 1 Dose Response: 0.1372

Test 2 Homogeneity of Variance: 0.732

Test 3 Variance Model Selection: 0.732

## Settings

| Setting | Value |
| --- | --- |
| BMR | 1.0 Standard Deviation |
| Distribution | Lognormal + Constant variance |
| Adverse Direction | Down (↓) |
| Maximum Polynomial Degree | 3 |
| Confidence Level (one sided) | 0.95 |

## Maximum Likelihood Approach

| Model | BMDL | BMD | BMDU | *P*-Value | AIC | Scaled Residual at Control | Scaled Residual near BMD | Recommendation and Notes |
| --- | --- | --- | --- | --- | --- | --- | --- | --- |
| Exponential 3 | 1.037 | 2.695 | 7.209 | 0.166 | 290.636 | 65.9 | 15.939 | **Questionable** \|Residual near BMD\| > 2.0 Residual at control > 2.0 BMD/highest dose ratio > 1.0 |
| Exponential 5 | 0.495 | 0.506 | - | - | 291.101 | 21.436 | 9.662 | **Questionable** \|Residual near BMD\| > 2.0 Zero degrees of freedom; saturated model Residual at control > 2.0 |


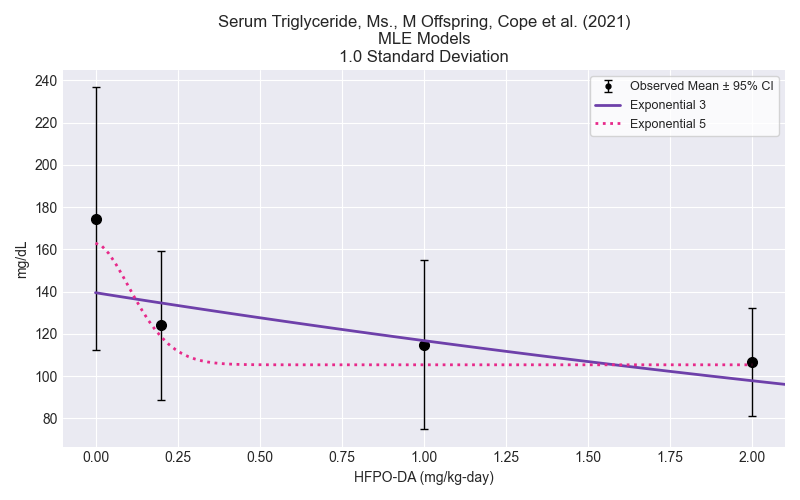


## Selected Model

## No model was selected as a best-fitting model.

# Serum Triglyceride, Ms., F Offspring, Cope et al. (2021)

## Dataset

**Name:** Serum Triglyceride, Ms., F Offspring, Cope et al. (2021)

| Dose (mg/kg-day) | N | Mean | Std. Dev. |
| --- | --- | --- | --- |
| 0 | 7 | 194.4 | 44.8 |
| 0.2 | 7 | 112.2 | 30.8 |
| 1 | 7 | 106 | 26.5 |
| 2 | 7 | 109.9 | 26.8 |

Test 1 Dose Response: <0.0001

Test 2 Homogeneity of Variance: 0.4235

Test 3 Variance Model Selection: 0.4235

## Settings

| Setting | Value |
| --- | --- |
| BMR | 1.0 Standard Deviation |
| Distribution | Normal + Constant variance |
| Adverse Direction | Down (↓) |
| Maximum Polynomial Degree | 3 |
| Confidence Level (one sided) | 0.95 |

## Maximum Likelihood Approach

| Model | BMDL | BMD | BMDU | *P*-Value | AIC | Scaled Residual at Control | Scaled Residual near BMD | Recommendation and Notes |
| --- | --- | --- | --- | --- | --- | --- | --- | --- |
| Exponential 3 | 0.651 | 1.213 | 3.593 | <0.001 | 294.577 | 2.371 | -0.978 | **Questionable** Residual at control > 2.0 Goodness of fit p-value < 0.1 |
| Exponential 5 | 0.006 | 0.046 | 0.191 | - | 281.135 | <0.001 | <0.001 | **Questionable** lowest dose/BMDL ratio > 3.0 lowest dose/BMDL ratio > 10.0 lowest dose/BMD ratio > 3.0 Zero degrees of freedom; saturated model BMD/BMDL ratio > 3.0 |
| Hill | 0.001 | 0.152 | 0.179 | - | 281.135 | <0.001 | <0.001 | **Questionable** lowest dose/BMDL ratio > 3.0 lowest dose/BMDL ratio > 10.0 Zero degrees of freedom; saturated model BMD/BMDL ratio > 3.0 BMD/BMDL ratio > 20.0 |
| Polynomial 2 | 0.918 | 1.674 | 4.238 | <0.001 | 295.529 | 2.706 | 0.607 | **Questionable** Residual at control > 2.0 Goodness of fit p-value < 0.1 |
| Polynomial 3 | 0.923 | 1.534 | 4.143 | <0.001 | 295.459 | 2.585 | 0.781 | **Questionable** Residual at control > 2.0 Goodness of fit p-value < 0.1 |
| Power | 0.923 | 1.512 | 4.141 | <0.001 | 295.457 | 2.568 | 0.811 | **Questionable** Residual at control > 2.0 Goodness of fit p-value < 0.1 |
| Linear | 0.923 | 1.512 | 4.141 | <0.001 | 295.457 | 2.568 | 0.811 | **Questionable** Residual at control > 2.0 Goodness of fit p-value < 0.1 |


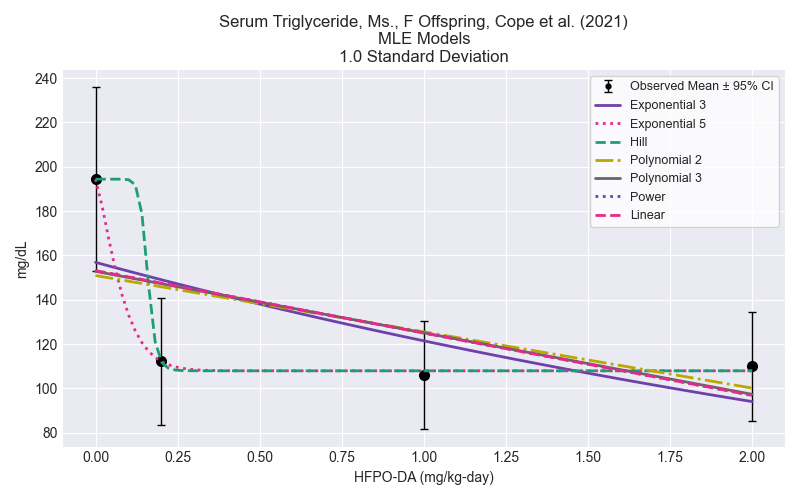


## Selected Model

No model was selected as a best-fitting model.

## Serum Triglyceride, Ms., F Offspring, Cope et al. (2021)

## Dataset

**Name:** Serum Triglyceride, Ms., F Offspring, Cope et al. (2021)

| Dose (mg/kg-day) | N | Mean | Std. Dev. |
| --- | --- | --- | --- |
| 0 | 7 | 194.4 | 44.8 |
| 0.2 | 7 | 112.2 | 30.8 |
| 1 | 7 | 106 | 26.5 |
| 2 | 7 | 109.9 | 26.8 |

Test 1 Dose Response: <0.0001

Test 2 Homogeneity of Variance: 0.4235

Test 3 Variance Model Selection: 0.9442

## Settings

| Setting | Value |
| --- | --- |
| BMR | 1.0 Standard Deviation |
| Distribution | Normal + Nonconstant variance |
| Adverse Direction | Down (↓) |
| Maximum Polynomial Degree | 3 |
| Confidence Level (one sided) | 0.95 |

## Maximum Likelihood Approach

| Model | BMDL | BMD | BMDU | *P*-Value | AIC | Scaled Residual at Control | Scaled Residual near BMD | Recommendation and Notes |
| --- | --- | --- | --- | --- | --- | --- | --- | --- |
| Exponential 3 | 1.085 | 2.625 | 4.292 | <0.001 | 291.828 | 2.356 | 0.63 | **Questionable** Residual at control > 2.0 Goodness of fit p-value < 0.1 BMD/highest dose ratio > 1.0 |
| Exponential 5 | 0.008 | 0.062 | 0.201 | - | 280.473 | 0.006 | 0.006 | **Questionable** lowest dose/BMDL ratio > 3.0 lowest dose/BMDL ratio > 10.0 lowest dose/BMD ratio > 3.0 Zero degrees of freedom; saturated model BMD/BMDL ratio > 3.0 |
| Hill | <0.001 | 0.172 | 0.187 | 0.774 | 278.475 | -0.009 | -0.073 | **Questionable** lowest dose/BMDL ratio > 3.0 lowest dose/BMDL ratio > 10.0 BMD/BMDL ratio > 3.0 BMD/BMDL ratio > 20.0 |
| Polynomial 2 | 1.363 | 2.595 | 2.648 | <0.001 | 292.459 | 2.446 | 0.563 | **Questionable** Residual at control > 2.0 Goodness of fit p-value < 0.1 BMD/highest dose ratio > 1.0 |
| Polynomial 3 | 1.365 | 2.653 | 2.708 | <0.001 | 292.447 | 2.442 | 0.535 | **Questionable** Residual at control > 2.0 Goodness of fit p-value < 0.1 BMD/highest dose ratio > 1.0 |
| Power | 1.366 | 2.71 | 3.476 | <0.001 | 292.441 | 2.47 | 0.514 | **Questionable** Residual at control > 2.0 Goodness of fit p-value < 0.1 BMD/highest dose ratio > 1.0 |
| Linear | 1.366 | 2.74 | 4.602 | <0.001 | 292.439 | 2.474 | 0.502 | **Questionable** Residual at control > 2.0 Goodness of fit p-value < 0.1 BMD/highest dose ratio > 1.0 |


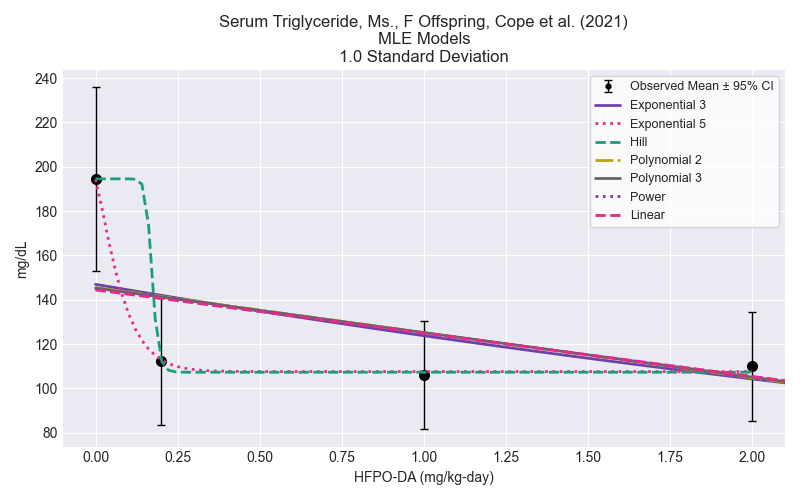


## Selected Model

No model was selected as a best-fitting model.

## Serum Triglyceride, Ms., F Offspring, Cope et al. (2021)

## Dataset

**Name:** Serum Triglyceride, Ms., F Offspring, Cope et al. (2021)

| Dose (mg/kg-day) | N | Mean | Std. Dev. |
| --- | --- | --- | --- |
| 0 | 7 | 194.4 | 44.8 |
| 0.2 | 7 | 112.2 | 30.8 |
| 1 | 7 | 106 | 26.5 |
| 2 | 7 | 109.9 | 26.8 |

Test 1 Dose Response: 0.0009

Test 2 Homogeneity of Variance: 0.9754

Test 3 Variance Model Selection: 0.9754

## Settings

| Setting | Value |
| --- | --- |
| BMR | 1.0 Standard Deviation |
| Distribution | Lognormal + Constant variance |
| Adverse Direction | Down (↓) |
| Maximum Polynomial Degree | 3 |
| Confidence Level (one sided) | 0.95 |

## Maximum Likelihood Approach

| Model | BMDL | BMD | BMDU | *P*-Value | AIC | Scaled Residual at Control | Scaled Residual near BMD | Recommendation and Notes |
| --- | --- | --- | --- | --- | --- | --- | --- | --- |
| Exponential 3 | 0.957 | 2.308 | 4.783 | <0.001 | 288.232 | 99.561 | 23.608 | **Questionable** \|Residual near BMD\| > 2.0 Residual at control > 2.0 Goodness of fit p-value < 0.1 BMD/highest dose ratio > 1.0 |
| Exponential 5 | 0.009 | 0.083 | 0.085 | - | 276.032 | 9.91 | 9.91 | **Questionable** \|Residual near BMD\| > 2.0 lowest dose/BMDL ratio > 3.0 lowest dose/BMDL ratio > 10.0 Zero degrees of freedom; saturated model Residual at control > 2.0 BMD/BMDL ratio > 3.0 |


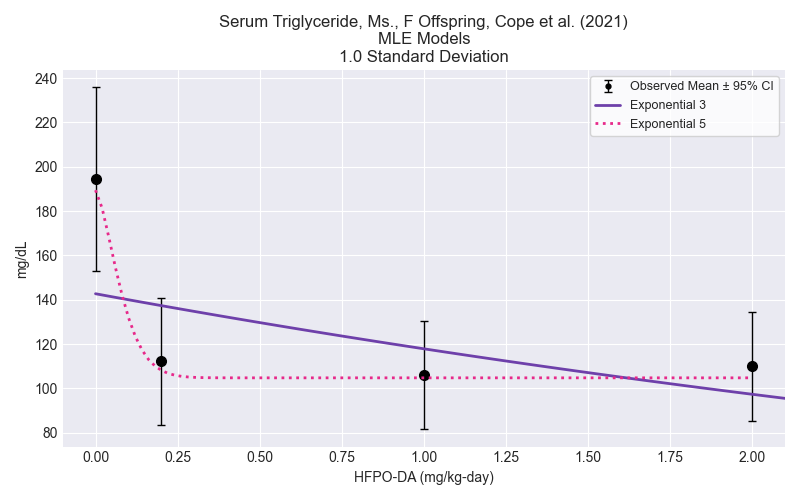


## Selected Model

No model was selected as a best-fitting model.

# Serum triglycerides, dams, E11.5, Ms, F, Blake et al. (2020)

## Dataset

**Name:** Serum triglycerides, dams, E11.5, Ms, F, Blake et al. (2020)

| Dose (mg/kg-day) | N | Mean | Std. Dev. |
| --- | --- | --- | --- |
| 0 | 5 | 205.6 | 56 |
| 2 | 5 | 117.6 | 33.9 |
| 10 | 4 | 80.3 | 14.4 |

Test 1 Dose Response: 0.0001

Test 2 Homogeneity of Variance: 0.0369

Test 3 Variance Model Selection: 0.0369

## Settings

| Setting | Value |
| --- | --- |
| BMR | 1.0 Standard Deviation |
| Distribution | Normal + Constant variance |
| Adverse Direction | Down (↓) |
| Maximum Polynomial Degree | 2 |
| Confidence Level (one sided) | 0.95 |

## Maximum Likelihood Approach

| Model | BMDL | BMD | BMDU | *P*-Value | AIC | Scaled Residual at Control | Scaled Residual near BMD | Recommendation and Notes |
| --- | --- | --- | --- | --- | --- | --- | --- | --- |
| Exponential 3 | 1.144 | 2.392 | 6.611 | 0.021 | 151.131 | 1.036 | -1.616 | **Questionable** Goodness of fit p-value < 0.1 Constant variance test failed (Test 2 p-value < 0.05) |
| Exponential 5 | 0.037 | 1.276 | 1.979 | - | 149.772 | <0.001 | <0.001 | **Questionable** lowest dose/BMDL ratio > 3.0 lowest dose/BMDL ratio > 10.0 Zero degrees of freedom; saturated model Constant variance test failed (Test 2 p-value < 0.05) BMD/BMDL ratio > 3.0 BMD/BMDL ratio > 20.0 |
| Hill | <0.001 | 0.588 | 0.853 | - | 149.772 | <0.001 | <0.001 | **Questionable** lowest dose/BMDL ratio > 3.0 lowest dose/BMDL ratio > 10.0 lowest dose/BMD ratio > 3.0 Zero degrees of freedom; saturated model Constant variance test failed (Test 2 p-value < 0.05) BMD/BMDL ratio > 3.0 BMD/BMDL ratio > 20.0 |
| Polynomial 2 | 2.8 | 4.364 | 9.795 | 0.008 | 152.866 | 1.441 | -1.816 | **Questionable** Goodness of fit p-value < 0.1 Constant variance test failed (Test 2 p-value < 0.05) |
| Power | 2.8 | 4.378 | 9.795 | 0.008 | 152.866 | 1.452 | -1.815 | **Questionable** Goodness of fit p-value < 0.1 Constant variance test failed (Test 2 p-value < 0.05) |
| Linear | 2.8 | 4.378 | 9.795 | 0.008 | 152.866 | 1.452 | -1.815 | **Questionable** Goodness of fit p-value < 0.1 Constant variance test failed (Test 2 p-value < 0.05) |


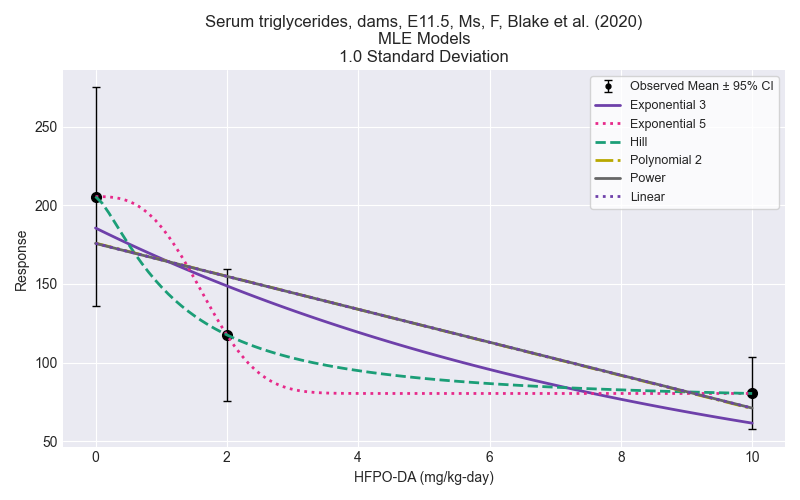


## Selected Model

No model was selected as a best-fitting model.

## Serum triglycerides, dams, E11.5, Ms, F, Blake et al. (2020)

## Dataset

**Name:** Serum triglycerides, dams, E11.5, Ms, F, Blake et al. (2020)

| Dose (mg/kg-day) | N | Mean | Std. Dev. |
| --- | --- | --- | --- |
| 0 | 5 | 205.6 | 56 |
| 2 | 5 | 117.6 | 33.9 |
| 10 | 4 | 80.3 | 14.4 |

Test 1 Dose Response: 0.0001

Test 2 Homogeneity of Variance: 0.0369

Test 3 Variance Model Selection: 0.4517

## Settings

| Setting | Value |
| --- | --- |
| BMR | 1.0 Standard Deviation |
| Distribution | Normal + Nonconstant variance |
| Adverse Direction | Down (↓) |
| Maximum Polynomial Degree | 2 |
| Confidence Level (one sided) | 0.95 |

## Maximum Likelihood Approach

| Model | BMDL | BMD | BMDU | *P*-Value | AIC | Scaled Residual at Control | Scaled Residual near BMD | Recommendation and Notes |
| --- | --- | --- | --- | --- | --- | --- | --- | --- |
| Exponential 3 | 2.855 | 5.682 | 12.431 | 0.031 | 146.417 | 1.357 | -1.442 | **Questionable** Goodness of fit p-value < 0.1 |
| Exponential 5 | 0.447 | 1.102 | 10.386 | - | 145.74 | 0.138 | -0.247 | **Questionable** lowest dose/BMDL ratio > 3.0 Zero degrees of freedom; saturated model |
| Hill | 0.262 | 1.861 | 10.523 | - | 145.74 | 0.138 | -0.247 | **Questionable** lowest dose/BMDL ratio > 3.0 Zero degrees of freedom; saturated model BMD/BMDL ratio > 3.0 |
| Polynomial 2 | 4.237 | 6.904 | 7.046 | 0.018 | 147.324 | 1.481 | 0.097 | **Questionable** Goodness of fit p-value < 0.1 |
| Power | 4.233 | 6.789 | 6.929 | 0.018 | 147.334 | 1.467 | 0.119 | **Questionable** Goodness of fit p-value < 0.1 |
| Linear | 4.239 | 6.999 | 12.679 | 0.018 | 147.322 | 1.49 | 0.079 | **Questionable** Goodness of fit p-value < 0.1 |


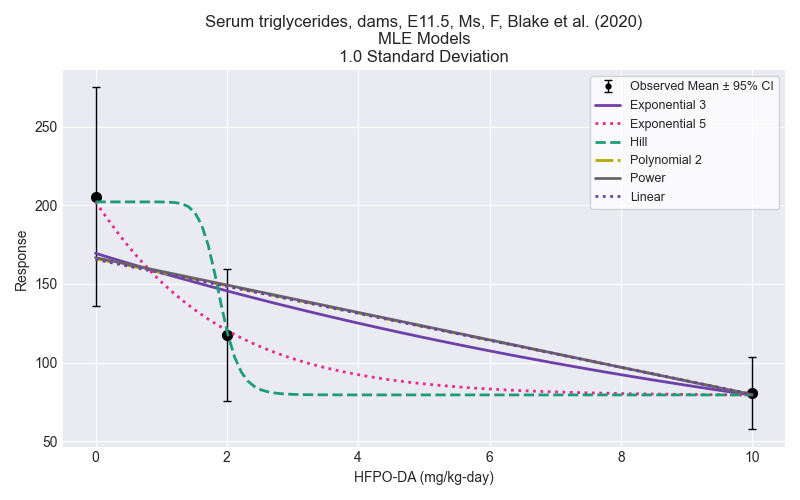


## Selected Model

No model was selected as a best-fitting model.

## Serum triglycerides, dams, E11.5, Ms, F, Blake et al. (2020)

## Dataset

**Name:** Serum triglycerides, dams, E11.5, Ms, F, Blake et al. (2020)

| Dose (mg/kg-day) | N | Mean | Std. Dev. |
| --- | --- | --- | --- |
| 0 | 5 | 205.6 | 56 |
| 2 | 5 | 117.6 | 33.9 |
| 10 | 4 | 80.3 | 14.4 |

Test 1 Dose Response: 0.0006

Test 2 Homogeneity of Variance: 0.58

Test 3 Variance Model Selection: 0.58

## Settings

| Setting | Value |
| --- | --- |
| BMR | 1.0 Standard Deviation |
| Distribution | Lognormal + Constant variance |
| Adverse Direction | Down (↓) |
| Maximum Polynomial Degree | 0 |
| Confidence Level (one sided) | 0.95 |

## Maximum Likelihood Approach

| Model | BMDL | BMD | BMDU | *P*-Value | AIC | Scaled Residual at Control | Scaled Residual near BMD | Recommendation and Notes |
| --- | --- | --- | --- | --- | --- | --- | --- | --- |
| Exponential 3 | 2.38 | 4.982 | 6.855 | 0.01 | 145.566 | 66.102 | -40.825 | **Questionable** \|Residual near BMD\| > 2.0 Residual at control > 2.0 Goodness of fit p-value < 0.1 |
| Exponential 5 | 0.359 | 1.225 | 1.304 | - | 142.935 | 12.476 | 7.973 | **Questionable** \|Residual near BMD\| > 2.0 lowest dose/BMDL ratio > 3.0 Zero degrees of freedom; saturated model Residual at control > 2.0 BMD/BMDL ratio > 3.0 |


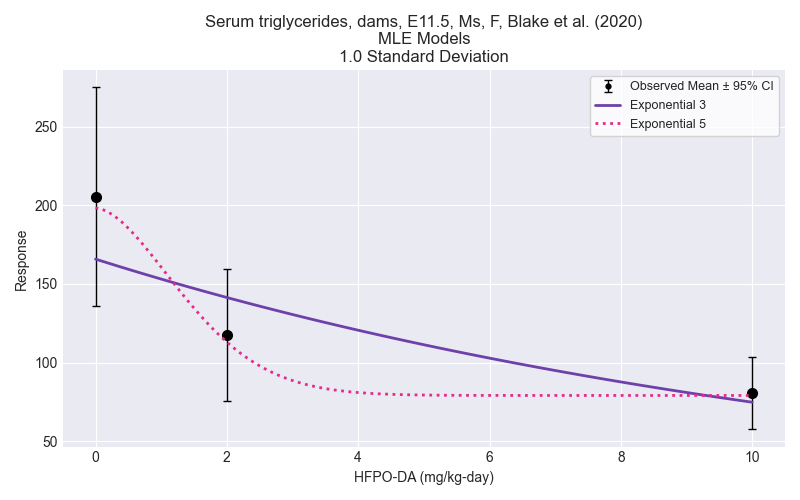


## Selected Model

No model was selected as a best-fitting model.

# Serum triglycerides, dams, E17.5, Ms, F, Blake et al. (2020)

## Dataset

**Name:** Serum triglycerides, dams, E17.5, Ms, F, Blake et al. (2020)

| Dose (mg/kg-day) | N | Mean | Std. Dev. |
| --- | --- | --- | --- |
| 0 | 4 | 472.5 | 78.9 |
| 2 | 5 | 257 | 120.3 |
| 10 | 5 | 120.6 | 31.7 |

Test 1 Dose Response: <0.0001

Test 2 Homogeneity of Variance: 0.0275

Test 3 Variance Model Selection: 0.0275

## Settings

| Setting | Value |
| --- | --- |
| BMR | 1.0 Standard Deviation |
| Distribution | Normal + Constant variance |
| Adverse Direction | Down (↓) |
| Maximum Polynomial Degree | 2 |
| Confidence Level (one sided) | 0.95 |

## Maximum Likelihood Approach

| Model | BMDL | BMD | BMDU | *P*-Value | AIC | Scaled Residual at Control | Scaled Residual near BMD | Recommendation and Notes |
| --- | --- | --- | --- | --- | --- | --- | --- | --- |
| Exponential 3 | 0.645 | 1.359 | 1.387 | 0.041 | 171.12 | 0.948 | -1.281 | **Questionable** lowest dose/BMDL ratio > 3.0 Goodness of fit p-value < 0.1 Constant variance test failed (Test 2 p-value < 0.05) |
| Exponential 5 | 0.283 | 0.662 | 1.954 | - | 170.942 | <0.001 | <0.001 | **Questionable** lowest dose/BMDL ratio > 3.0 lowest dose/BMD ratio > 3.0 Zero degrees of freedom; saturated model Constant variance test failed (Test 2 p-value < 0.05) |
| Hill | 0.158 | 0.78 | 1.008 | - | 170.942 | <0.001 | <0.001 | **Questionable** lowest dose/BMDL ratio > 3.0 lowest dose/BMDL ratio > 10.0 Zero degrees of freedom; saturated model Constant variance test failed (Test 2 p-value < 0.05) BMD/BMDL ratio > 3.0 |
| Polynomial 2 | 2.331 | 3.437 | 6.705 | 0.006 | 174.454 | 1.59 | -1.777 | **Questionable** Goodness of fit p-value < 0.1 Constant variance test failed (Test 2 p-value < 0.05) |
| Power | 2.33 | 3.435 | 6.439 | 0.006 | 174.454 | 1.59 | -1.778 | **Questionable** Goodness of fit p-value < 0.1 Constant variance test failed (Test 2 p-value < 0.05) |
| Linear | 2.33 | 3.435 | 6.358 | 0.006 | 174.454 | 1.59 | -1.778 | **Questionable** Goodness of fit p-value < 0.1 Constant variance test failed (Test 2 p-value < 0.05) |


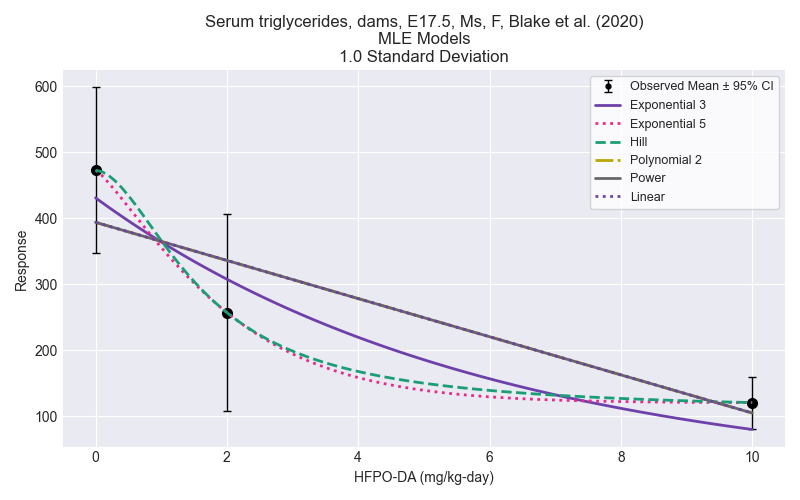


## Selected Model

No model was selected as a best-fitting model.

## Serum triglycerides, dams, E17.5, Ms, F, Blake et al. (2020)

## Dataset

**Name:** Serum triglycerides, dams, E17.5, Ms, F, Blake et al. (2020)

| Dose (mg/kg-day) | N | Mean | Std. Dev. |
| --- | --- | --- | --- |
| 0 | 4 | 472.5 | 78.9 |
| 2 | 5 | 257 | 120.3 |
| 10 | 5 | 120.6 | 31.7 |

Test 1 Dose Response: <0.0001

Test 2 Homogeneity of Variance: 0.0275

Test 3 Variance Model Selection: 0.0432

## Settings

| Setting | Value |
| --- | --- |
| BMR | 1.0 Standard Deviation |
| Distribution | Normal + Nonconstant variance |
| Adverse Direction | Down (↓) |
| Maximum Polynomial Degree | 2 |
| Confidence Level (one sided) | 0.95 |

## Maximum Likelihood Approach

| Model | BMDL | BMD | BMDU | *P*-Value | AIC | Scaled Residual at Control | Scaled Residual near BMD | Recommendation and Notes |
| --- | --- | --- | --- | --- | --- | --- | --- | --- |
| Exponential 3 | 1.708 | 3.4 | 3.47 | 0.259 | 167.118 | 1.016 | -1.298 | **Questionable** Nonconstant variance test failed (Test 3 p-value < 0.05) Control stdev. fit > 1.5 |
| Exponential 5 | 0.446 | 1.475 | 7.562 | - | 169.845 | 0.395 | -0.552 | **Questionable** lowest dose/BMDL ratio > 3.0 Zero degrees of freedom; saturated model Nonconstant variance test failed (Test 3 p-value < 0.05) Control stdev. fit > 1.5 BMD/BMDL ratio > 3.0 |
| Hill | 0.332 | 1.349 | 7.917 | - | 169.845 | 0.395 | -0.552 | **Questionable** lowest dose/BMDL ratio > 3.0 Zero degrees of freedom; saturated model Nonconstant variance test failed (Test 3 p-value < 0.05) Control stdev. fit > 1.5 BMD/BMDL ratio > 3.0 |
| Polynomial 2 | 4.153 | 5.459 | 7.31 | 0.129 | 168.144 | 1.413 | -1.306 | **Questionable** Nonconstant variance test failed (Test 3 p-value < 0.05) Control stdev. fit > 1.5 |
| Power | 3.388 | 5.548 | 11.439 | 0.129 | 168.146 | 1.419 | -1.275 | **Questionable** Nonconstant variance test failed (Test 3 p-value < 0.05) Control stdev. fit > 1.5 |
| Linear | 3.39 | 5.459 | 11.434 | 0.129 | 168.144 | 1.413 | -1.306 | **Questionable** Nonconstant variance test failed (Test 3 p-value < 0.05) Control stdev. fit > 1.5 |


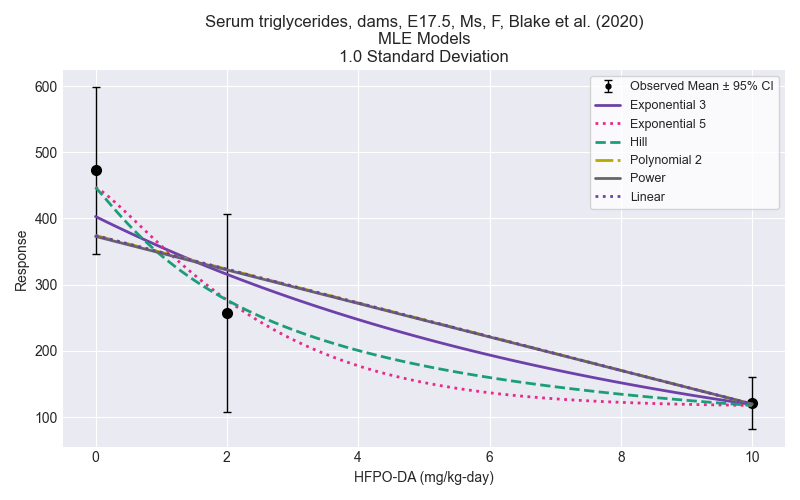


## Selected Model

No model was selected as a best-fitting model.

## Serum triglycerides, dams, E17.5, Ms, F, Blake et al. (2020)

## Dataset

**Name:** Serum triglycerides, dams, E17.5, Ms, F, Blake et al. (2020)

| Dose (mg/kg-day) | N | Mean | Std. Dev. |
| --- | --- | --- | --- |
| 0 | 4 | 472.5 | 78.9 |
| 2 | 5 | 257 | 120.3 |
| 10 | 5 | 120.6 | 31.7 |

Test 1 Dose Response: <0.0001

Test 2 Homogeneity of Variance: 0.1168

Test 3 Variance Model Selection: 0.1168

## Settings

| Setting | Value |
| --- | --- |
| BMR | 1.0 Standard Deviation |
| Distribution | Lognormal + Constant variance |
| Adverse Direction | Down (↓) |
| Maximum Polynomial Degree | 0 |
| Confidence Level (one sided) | 0.95 |

## Maximum Likelihood Approach

| Model | BMDL | BMD | BMDU | *P*-Value | AIC | Scaled Residual at Control | Scaled Residual near BMD | Recommendation and Notes |
| --- | --- | --- | --- | --- | --- | --- | --- | --- |
| Exponential 3 | 1.972 | 4.306 | 5.293 | 0.028 | 166.742 | 140.301 | -58.835 | **Questionable** \|Residual near BMD\| > 2.0 Residual at control > 2.0 Goodness of fit p-value < 0.1 |
| Exponential 5 | 0.436 | 1.307 | 1.339 | - | 165.89 | 7.364 | 39.428 | **Questionable** \|Residual near BMD\| > 2.0 lowest dose/BMDL ratio > 3.0 Zero degrees of freedom; saturated model Residual at control > 2.0 |


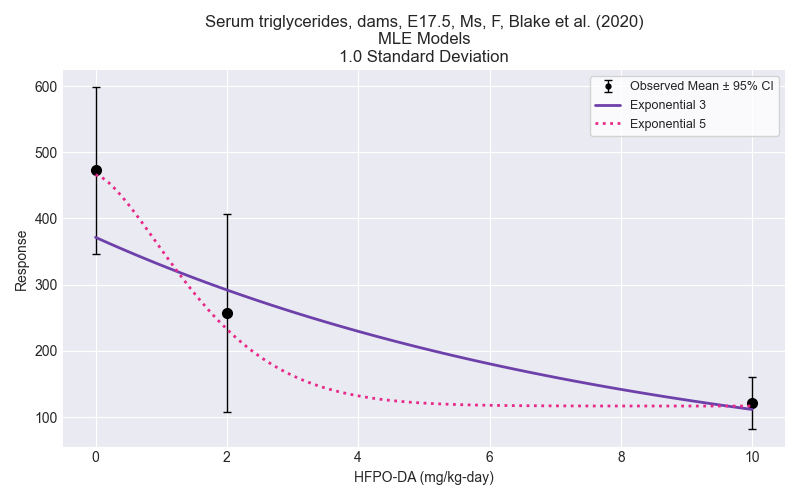


## Selected Model

No model was selected as a best-fitting model.
