# Supplementary material for: Integration of mechanistic and repeat dose toxicity data in the derivation of an oral reference dose for HFPO-DA
Source: Toxicol Sci. 2026 Apr 10;209(5):kfag045. doi: 10.1093/toxsci/kfag045 (PMC13176454; doi:10.1093/toxsci/kfag045)
Supplement: kfag045_Supplementary_Data [file kfag045_supplementary_data.zip › Supplementary File S4.docx]

Table of Contents

[Summary Table of BMD Modeling Results 3](#_Toc223075420)

[Testes, reduced germ cell cellularity, Ms, M, 18-mo, CRL (2024) 4](#_Toc223075421)

[Liver, hepatocellular hypertrophy, Ms, M, 18-mo, CRL (2024) 7](#_Toc223075422)

[Liver, hepatocellular hypertrophy, Ms, F, 18-mo, CRL (2024) 10](#_Toc223075423)

[Liver, biliary hyperplasia, Ms, M, 18-mo, CRL (2024) 13](#_Toc223075424)

[Liver, Cystic focal degen, Rt, M, 24-mo, Rae (2015) 16](#_Toc223075425)

[Liver, EPA constellation, Ms, M, Repro 19](#_Toc223075426)

[Liver, EPA constellation, Ms, F, Repro 22](#_Toc223075427)

[Adrenal gland, cortical hypertrophy, Ms, M, 18-mo, CRL (2024) 25](#_Toc223075428)

[Serum Triglyceride, Ms., M Offspring, Cope et al. (2021) 29](#_Toc223075429)

[Serum Triglyceride, Ms., F Offspring, Cope et al. (2021) 35](#_Toc223075430)

[Placenta weight, Rt, F, Lv et al. (2024) 40](#_Toc223075431)

[Pup weight, Ms, M, PND4, DuPont (2010) 43](#_Toc223075432)

[Pup birthweight GD8-PND2, Rt, F, Conley et al. (2021) 47](#_Toc223075433)

[Serum triglycerides, Rt, F, Conley et al. (2021) 51](#_Toc223075434)

[Serum triglycerides, dams, E11.5, Ms, F, Blake et al. (2020) 55](#_Toc223075435)

[Serum triglycerides, dams, E17.5, Ms, F, Blake et al. (2020) 60](#_Toc223075436)

[Fetal length (ND), Rt, F, GD0.5-19.5, Lv et al. (2024) 65](#_Toc223075437)

[Fetal tail length (ND), Rt, F, GD0.5-19.5, Lv et al. (2024) 69](#_Toc223075438)

# Summary Table of BMD Modeling Results

| **Endpoint in Main Text Table 5** | **Page** | | **BMR** | **BMD**  **(mg/kg-day)** | **BMDL**  **(mg/kg-day)** | **Model** |
| --- | --- | --- | --- | --- | --- | --- |
| Liver focal cystic degeneration in male rats; Rae (2015) | 16 | | 10%er | 9.9 | 6.5 | Multistage 1^a^ |
| Biliary hyperplasia in male mice; (CRL, 2024) | 13 | | 10%er | 4.8 | 2.0 | Weibull |
| Reduced testes germ cell cellularity in mice; CRL (2024) | 4 | | 10%er | 0.34 | 0.20 | LogProbit^a^ |
| Adrenal gland hypertrophy in male mice; CRL (2024) | 25 | | 10%er | 2.1 | 0.87 | Multistage 3^a^ |
| Decreased serum triglycerides, rat dams GD16-20 Conley et al. (2021) | | 51 | 1SD | 5.9 | 0.66 | Hill  N-CV |
| Decreased serum triglycerides in mouse dams, E11.5; Blake et al. (2020) | | 55 | 1SD | NA | NA | LOAEL |
| Decreased serum triglycerides in mouse dams, E17.5; Blake et al. (2020) | | 60 | 1SD | NA | NA | NOAEL |
| Increased placental weight in female rats; Lv et al. (2024) | | 40 | 5%rd | 16.6 | 11.9 | Linear  N-CV |
| Increased placental lesions in female mice; Blake et al. (2020) | | NA | NA | NA | NA | LOAEL |
| Decreased fetal length; Lv et al. (2024) | | 65 | 5%rd | 6.0 | 3.6 | Exponential 5  N-NCV |
| Decreased fetal tail length; Lv et al. (2024) | | 69 | 5%rd | NA | NA | NOAEL |
| Decreased rat birthweight; Conley et al. (2021) | | 47 | 5%rd | 24.2 | 13.5 | Hill  N-CV |
| Decreased male mouse pup weight on PND4; DuPont (2010) | | 43 | 5%rd | 3.2 | 1.1 | Polynomial 3  N-CV |
| Decreased serum triglycerides in female mouse offspring; Cope et al. (2021) | | 35 | 5%rd | NA | NA | LOAEL |
| Decreased serum triglycerides in male mouse offspring; Cope et al. (2021) | | 29 | 1SD | 1.6 | 0.81 | Exponential 3  N-CV |
| Decreased pup liver glycogen;  Conley et al. (2021) | | NA | NA | NA | NA | LOAEL |

^a^ Selected model differed from BMDS recommendation; see model output for rationale.

er = extra risk; rd = relative deviation; sd = standard deviation; N = normal; CV = constant variance; NCV = non-constant variance

# Testes, reduced germ cell cellularity, Ms, M, 18-mo, CRL (2024)

## Dataset

**Name:** Testes, reduced germ cell cellularity, Ms, M, 18-mo

| Dose (mg/kg-day) | N | Incidence |
| --- | --- | --- |
| 0 | 44 | 0 |
| 0.05 | 43 | 0 |
| 0.1 | 43 | 0 |
| 0.5 | 42 | 9 |
| 5 | 34 | 22 |

## Settings

| Setting | Value |
| --- | --- |
| BMR | 10% Extra Risk |
| Confidence Level (one sided) | 0.95 |
| Maximum Multistage Degree | 3 |

## Maximum Likelihood Approach

| Model | BMDL | BMD | BMDU | *P*-Value | AIC | Scaled Residual at Control | Scaled Residual near BMD | Recommendation and Notes |
| --- | --- | --- | --- | --- | --- | --- | --- | --- |
| Hill^a^ | 0.251 | 0.442 | 0.485 | 1. | 93.794 | -0.001 | <0.001 | **Recommended - Lowest AIC** |
| Gamma | 0.327 | 0.44 | 0.656 | 0.178 | 96.819 | -0.001 | 2.077 | **Questionable** \|Residual near BMD\| > 2.0 |
| LogLogistic | 0.206 | 0.349 | 0.572 | 0.291 | 97.01 | -0.001 | 1.333 | **Viable** |
| Multistage 1 | 0.327 | 0.44 | 0.607 | 0.178 | 96.819 | -0.001 | 2.077 | **Questionable** \|Residual near BMD\| > 2.0 |
| Multistage 2 | 0.327 | 0.44 | 0.646 | 0.178 | 96.819 | -0.001 | 2.077 | **Questionable** \|Residual near BMD\| > 2.0 |
| Multistage 3 | 0.327 | 0.44 | 0.648 | 0.178 | 96.819 | -0.001 | 2.077 | **Questionable** \|Residual near BMD\| > 2.0 |
| Weibull | 0.327 | 0.44 | 0.654 | 0.178 | 96.819 | -0.001 | 2.077 | **Questionable** \|Residual near BMD\| > 2.0 |
| Logistic | 1.337 | 1.684 | 2.097 | <0.001 | 114.425 | -1.409 | 4.104 | **Questionable** \|Residual near BMD\| > 2.0 Goodness of fit p-value < 0.1 |
| LogProbit^b^ | 0.202 | 0.34 | 0.529 | 0.467 | 95.261 | -0.001 | 1.126 | **Viable** |
| Probit | 1.199 | 1.491 | 1.851 | <0.001 | 113.467 | -1.354 | 4.05 | **Questionable** \|Residual near BMD\| > 2.0 Goodness of fit p-value < 0.1 |
| Quantal Linear | 0.327 | 0.44 | 0.607 | 0.178 | 96.819 | -0.001 | 2.077 | **Questionable** \|Residual near BMD\| > 2.0 |

^a^ BMDS recommended best fitting model

^b^ viable alternate; better visual fit than the Hill model provided similar BMDL and next lowest AIC


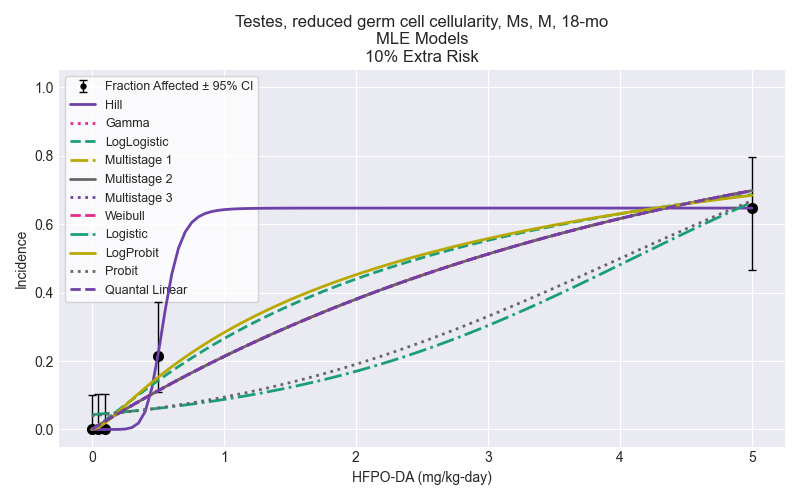


## Selected Model: LogProbit


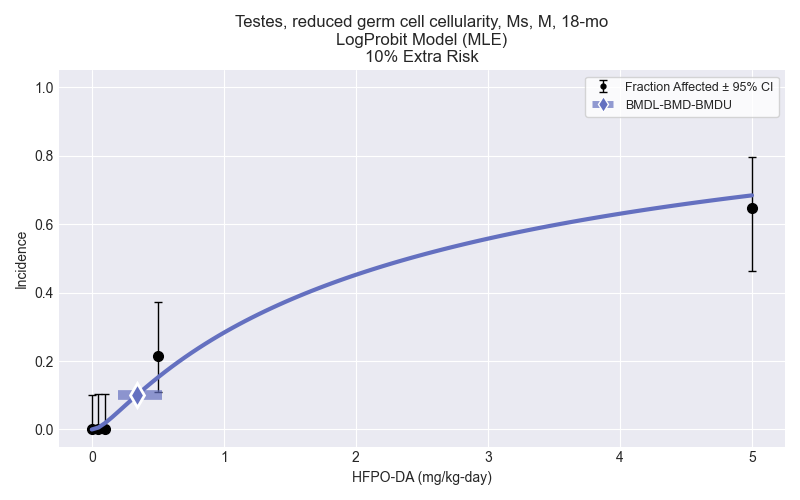


LogProbit Model
══════════════════════════════

Version: pybmds 25.1 (bmdscore 25.1)

Input Summary:
╒══════════════════════════════╤══════════════════════════╕
│ BMR │ 10% Extra Risk │
│ Confidence Level (one sided) │ 0.95 │
│ Modeling approach │ frequentist_unrestricted │
╘══════════════════════════════╧══════════════════════════╛

Parameter Settings:
╒═════════════╤═══════════╤══════════╤═══════╕
│ Parameter │ Initial │ Min │ Max │
╞═════════════╪═══════════╪══════════╪═══════╡
│ g │ 0 │ -18 │ 18 │
│ a │ 0 │ -18 │ 18 │
│ b │ 0.0001 │ 0.0001 │ 18 │
╘═════════════╧═══════════╧══════════╧═══════╛

Modeling Summary:
╒════════════════╤════════════╕
│ BMD │ 0.339623 │
│ BMDL │ 0.202159 │
│ BMDU │ 0.528728 │
│ AIC │ 95.2606 │
│ Log-Likelihood │ -45.6303 │
│ P-Value │ 0.466955 │
│ Overall d.f. │ 3 │
│ Chi² │ 2.54645 │
╘════════════════╧════════════╛

Model Parameters:
╒════════════╤════════════╤════════════╤══════════════╕
│ Variable │ Estimate │ On Bound │ Std Error │
╞════════════╪════════════╪════════════╪══════════════╡
│ g │ 1.523e-08 │ yes │ Not Reported │
│ a │ -0.574193 │ no │ 0.148736 │
│ b │ 0.655011 │ no │ 0.100253 │
╘════════════╧════════════╧════════════╧══════════════╛
Standard errors estimates are not generated for parameters estimated on corresponding bounds,
although sampling error is present for all parameters, as a rule. Standard error estimates may not
be reliable as a basis for confidence intervals or tests when one or more parameters are on bounds.


Goodness of Fit:
╒════════╤════════╤════════════╤══════════════╤════════════╤═══════════════════╕
│ Dose │ Size │ Observed │ Expected │ Est Prob │ Scaled Residual │
╞════════╪════════╪════════════╪══════════════╪════════════╪═══════════════════╡
│ 0 │ 44 │ 0 │ 6.70119e-07 │ 1.523e-08 │ -0.000818608 │
│ 0.05 │ 43 │ 0 │ 0.240778 │ 0.00559949 │ -0.492071 │
│ 0.1 │ 43 │ 0 │ 0.802058 │ 0.0186525 │ -0.904048 │
│ 0.5 │ 42 │ 9 │ 6.38086 │ 0.151925 │ 1.12591 │
│ 5 │ 34 │ 22 │ 23.2692 │ 0.684388 │ -0.468345 │
╘════════╧════════╧════════════╧══════════════╧════════════╧═══════════════════╛

Analysis of Deviance:
╒═══════════════╤══════════════════╤════════════╤════════════╤═════════════╤═══════════╕
│ Model │ Log-Likelihood │ # Params │ Deviance │ Test d.f. │ P-Value │
╞═══════════════╪══════════════════╪════════════╪════════════╪═════════════╪═══════════╡
│ Full model │ -43.8968 │ 5 │ - │ - │ - │
│ Fitted model │ -45.6303 │ 2 │ 3.46703 │ 3 │ 0.325063 │
│ Reduced model │ -87.2513 │ 1 │ 86.7091 │ 4 │ 0 │
╘═══════════════╧══════════════════╧════════════╧════════════╧═════════════╧═══════════╛

# Liver, hepatocellular hypertrophy, Ms, M, 18-mo, CRL (2024)

## Dataset

**Name:** Liver, hepatocellular hypertrophy, Mm, M, 18-mo

| Dose (mg/kg-day) | N | Incidence |
| --- | --- | --- |
| 0 | 44 | 2 |
| 0.05 | 43 | 2 |
| 0.1 | 43 | 9 |
| 0.5 | 42 | 38 |
| 5 | 34 | 33 |

## Settings

| Setting | Value |
| --- | --- |
| BMR | 10% Extra Risk |
| Confidence Level (one sided) | 0.95 |
| Maximum Multistage Degree | 3 |

## Maximum Likelihood Approach

| Model | BMDL | BMD | BMDU | *P*-Value | AIC | Scaled Residual at Control | Scaled Residual near BMD | Recommendation and Notes |
| --- | --- | --- | --- | --- | --- | --- | --- | --- |
| Hill^ab^ | 0.058 | 0.081 | 0.116 | 0.513 | 120.462 | 0.283 | 0.249 | **Recommended - Lowest AIC** |
| Gamma | 0.036 | 0.047 | 0.07 | 0 | 145.099 | 0.518 | -1.693 | **Questionable** Goodness of fit p-value < 0.1 |
| LogLogistic | 0.049 | 0.071 | 0.102 | <0.001 | 123.929 | 0.48 | -0.856 | **Questionable** Goodness of fit p-value < 0.1 |
| Multistage 1 | 0.036 | 0.047 | 0.062 | 0 | 145.099 | 0.518 | -1.693 | **Questionable** Goodness of fit p-value < 0.1 |
| Multistage 2 | 0.036 | 0.047 | 0.062 | 0 | 145.099 | 0.518 | -1.693 | **Questionable** Goodness of fit p-value < 0.1 |
| Multistage 3 | 0.036 | 0.047 | 0.062 | 0 | 145.099 | 0.518 | -1.693 | **Questionable** Goodness of fit p-value < 0.1 |
| Weibull | 0.05 | 0.076 | 0.1 | 0.556 | 147.131 | 0.366 | 0.476 | **Viable** |
| Logistic | 0.082 | 0.105 | 0.134 | 0.448 | 146.549 | -0.321 | 1.21 | **Viable** |
| LogProbit | 0.045 | 0.065 | 0.091 | <0.001 | 128.498 | 0.537 | -0.985 | **Questionable** Goodness of fit p-value < 0.1 |
| Probit | 0.223 | 0.293 | 0.414 | 0 | 199.64 | -2.979 | -0.773 | **Questionable** Residual at control > 2.0 Goodness of fit p-value < 0.1 |
| Quantal Linear | 0.036 | 0.047 | 0.062 | 0 | 145.099 | 0.518 | -1.693 | **Questionable** Goodness of fit p-value < 0.1 |

^a^ BMDS recommended best fitting model

^b^ User selected best fitting model


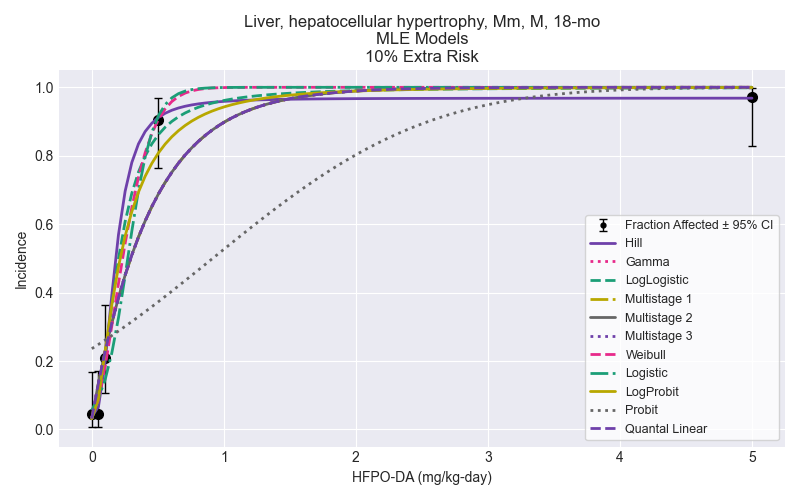


## Selected Model: Hill


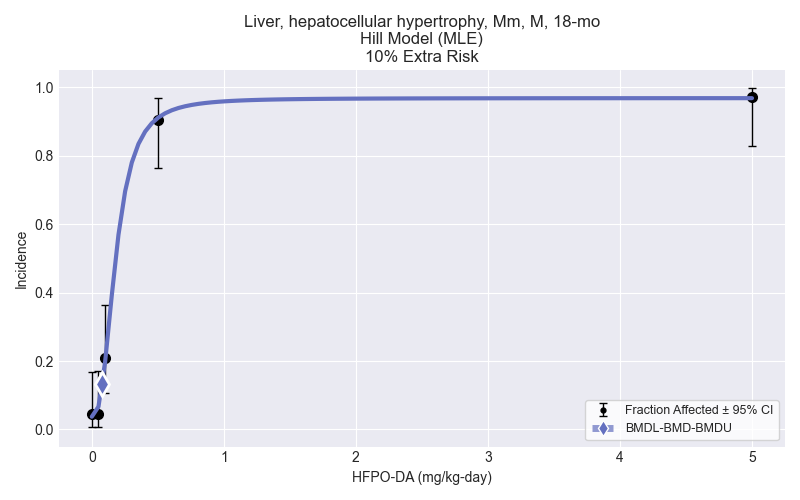


Hill Model
══════════════════════════════

Version: pybmds 25.1 (bmdscore 25.1)

Input Summary:
╒══════════════════════════════╤════════════════════════╕
│ BMR │ 10% Extra Risk │
│ Confidence Level (one sided) │ 0.95 │
│ Modeling approach │ frequentist_restricted │
╘══════════════════════════════╧════════════════════════╛

Parameter Settings:
╒═════════════╤═══════════╤═══════╤═══════╕
│ Parameter │ Initial │ Min │ Max │
╞═════════════╪═══════════╪═══════╪═══════╡
│ g │ 0 │ -18 │ 18 │
│ v │ 0 │ -18 │ 18 │
│ a │ 0 │ -18 │ 18 │
│ b │ 1 │ 1 │ 18 │
╘═════════════╧═══════════╧═══════╧═══════╛

Modeling Summary:
╒════════════════╤═════════════╕
│ BMD │ 0.081006 │
│ BMDL │ 0.0584821 │
│ BMDU │ 0.116216 │
│ AIC │ 120.462 │
│ Log-Likelihood │ -56.2308 │
│ P-Value │ 0.512501 │
│ Overall d.f. │ 1 │
│ Chi² │ 0.428957 │
╘════════════════╧═════════════╛

Model Parameters:
╒════════════╤════════════╤════════════╤═════════════╕
│ Variable │ Estimate │ On Bound │ Std Error │
╞════════════╪════════════╪════════════╪═════════════╡
│ g │ 0.0373585 │ no │ 0.0417457 │
│ v │ 0.967206 │ no │ 0.0413724 │
│ a │ 4.57052 │ no │ 1.38088 │
│ b │ 2.67807 │ no │ 0.621461 │
╘════════════╧════════════╧════════════╧═════════════╛

Goodness of Fit:
╒════════╤════════╤════════════╤════════════╤════════════╤═══════════════════╕
│ Dose │ Size │ Observed │ Expected │ Est Prob │ Scaled Residual │
╞════════╪════════╪════════════╪════════════╪════════════╪═══════════════════╡
│ 0 │ 44 │ 2 │ 1.64378 │ 0.0373585 │ 0.283185 │
│ 0.05 │ 43 │ 2 │ 2.83557 │ 0.0659434 │ -0.513423 │
│ 0.1 │ 43 │ 9 │ 8.35429 │ 0.194286 │ 0.248881 │
│ 0.5 │ 42 │ 38 │ 38.2441 │ 0.910574 │ -0.132005 │
│ 5 │ 34 │ 33 │ 32.9222 │ 0.968301 │ 0.0761145 │
╘════════╧════════╧════════════╧════════════╧════════════╧═══════════════════╛

Analysis of Deviance:
╒═══════════════╤══════════════════╤════════════╤════════════╤═════════════╤═══════════╕
│ Model │ Log-Likelihood │ # Params │ Deviance │ Test d.f. │ P-Value │
╞═══════════════╪══════════════════╪════════════╪════════════╪═════════════╪═══════════╡
│ Full model │ -56.0053 │ 5 │ - │ - │ - │
│ Fitted model │ -56.2308 │ 4 │ 0.450988 │ 1 │ 0.501866 │
│ Reduced model │ -139.263 │ 1 │ 166.516 │ 4 │ 0 │
╘═══════════════╧══════════════════╧════════════╧════════════╧═════════════╧═══════════╛

# Liver, hepatocellular hypertrophy, Ms, F, 18-mo, CRL (2024)

## Dataset

**Name:** Liver, hepatocellular hypertrophy, Mm, F, 18-mo

| Dose (mg/kg) | N | Incidence |
| --- | --- | --- |
| 0 | 43 | 0 |
| 0.05 | 40 | 1 |
| 0.1 | 41 | 0 |
| 0.5 | 43 | 14 |
| 5 | 37 | 37 |

## Settings

| Setting | Value |
| --- | --- |
| BMR | 10% Extra Risk |
| Confidence Level (one sided) | 0.95 |
| Maximum Multistage Degree | 3 |

## Maximum Likelihood Approach

| Model | BMDL | BMD | BMDU | *P*-Value | AIC | Scaled Residual at Control | Scaled Residual near BMD | Recommendation and Notes |
| --- | --- | --- | --- | --- | --- | --- | --- | --- |
| Hill | 0.213 | 0.419 | 0.45 | 0.347 | 71.899 | -0.591 | <0.001 | **Viable** |
| Gamma | 0.161 | 0.385 | 0.431 | 0.347 | 71.899 | -0.591 | <0.001 | **Viable** |
| LogLogistic | 0.213 | 0.419 | 0.45 | 0.347 | 71.899 | -0.591 | <0.001 | **Viable** |
| Multistage 1 | 0.101 | 0.138 | 0.19 | 0.371 | 73.732 | -0.001 | -1.802 | **Viable** |
| Multistage 2 | 0.154 | 0.244 | 0.335 | 0.531 | 70.419 | -0.001 | -1.005 | **Viable** |
| Multistage 3^ab^ | 0.162 | 0.287 | 0.382 | 0.551 | 70.02 | -0.001 | -0.879 | **Recommended - Lowest AIC** |
| Weibull | 0.154 | 0.295 | 0.371 | 0.315 | 72.295 | -0.538 | -0.771 | **Viable** |
| Logistic | 0.28 | 0.345 | 0.407 | 0.524 | 70.023 | -0.464 | 0.011 | **Viable** |
| LogProbit | 0.207 | 0.402 | 0.472 | 0.347 | 71.899 | -0.591 | <0.001 | **Viable** |
| Probit | 0.248 | 0.309 | 0.38 | 0.595 | 70.83 | -0.64 | 0.486 | **Viable** |
| Quantal Linear | 0.101 | 0.138 | 0.19 | 0.371 | 73.732 | -0.001 | -1.802 | **Viable** |

^a^ BMDS recommended best fitting model

^b^ User selected best fitting model


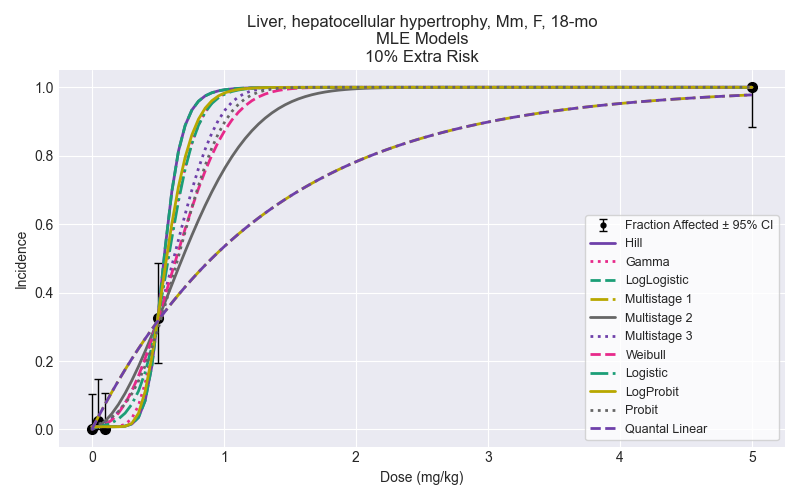


## Selected Model: Multistage 3


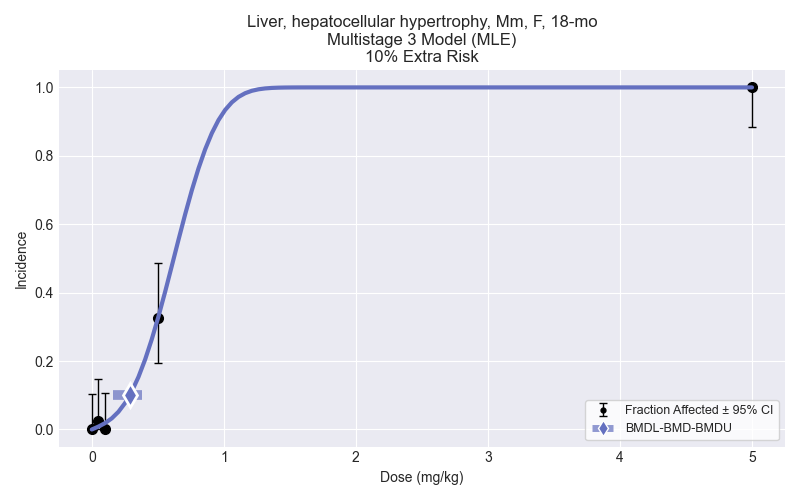


Multistage 3 Model
══════════════════════════════

Version: pybmds 25.1 (bmdscore 25.1)

Input Summary:
╒══════════════════════════════╤════════════════════════╕
│ BMR │ 10% Extra Risk │
│ Confidence Level (one sided) │ 0.95 │
│ Modeling approach │ frequentist_restricted │
│ Degree │ 3 │
╘══════════════════════════════╧════════════════════════╛

Parameter Settings:
╒═════════════╤═══════════╤═══════╤═══════╕
│ Parameter │ Initial │ Min │ Max │
╞═════════════╪═══════════╪═══════╪═══════╡
│ g │ 0 │ -18 │ 18 │
│ b1 │ 0 │ 0 │ 10000 │
│ b2 │ 0 │ 0 │ 10000 │
│ b3 │ 0 │ 0 │ 10000 │
╘═════════════╧═══════════╧═══════╧═══════╛

Modeling Summary:
╒════════════════╤════════════╕
│ BMD │ 0.287315 │
│ BMDL │ 0.162412 │
│ BMDU │ 0.382268 │
│ AIC │ 70.02 │
│ Log-Likelihood │ -33.01 │
│ P-Value │ 0.550518 │
│ Overall d.f. │ 3 │
│ Chi² │ 2.1069 │
╘════════════════╧════════════╛

Model Parameters:
╒════════════╤════════════╤════════════╤══════════════╕
│ Variable │ Estimate │ On Bound │ Std Error │
╞════════════╪════════════╪════════════╪══════════════╡
│ g │ 1.523e-08 │ yes │ Not Reported │
│ b1 │ 0.161842 │ no │ 0.729359 │
│ b2 │ 0 │ yes │ Not Reported │
│ b3 │ 2.48171 │ no │ 80.5512 │
╘════════════╧════════════╧════════════╧══════════════╛
Standard errors estimates are not generated for parameters estimated on corresponding bounds,
although sampling error is present for all parameters, as a rule. Standard error estimates may not
be reliable as a basis for confidence intervals or tests when one or more parameters are on bounds.


Goodness of Fit:
╒════════╤════════╤════════════╤══════════════╤════════════╤═══════════════════╕
│ Dose │ Size │ Observed │ Expected │ Est Prob │ Scaled Residual │
╞════════╪════════╪════════════╪══════════════╪════════════╪═══════════════════╡
│ 0 │ 43 │ 0 │ 6.54889e-07 │ 1.523e-08 │ -0.000809252 │
│ 0.05 │ 40 │ 1 │ 0.334686 │ 0.00836714 │ 1.15487 │
│ 0.1 │ 41 │ 0 │ 0.758205 │ 0.0184928 │ -0.878915 │
│ 0.5 │ 43 │ 14 │ 13.9196 │ 0.323711 │ 0.0262167 │
│ 5 │ 37 │ 37 │ 37 │ 1 │ 0 │
╘════════╧════════╧════════════╧══════════════╧════════════╧═══════════════════╛

Analysis of Deviance:
╒═══════════════╤══════════════════╤════════════╤════════════╤═════════════╤═══════════╕
│ Model │ Log-Likelihood │ # Params │ Deviance │ Test d.f. │ P-Value │
╞═══════════════╪══════════════════╪════════════╪════════════╪═════════════╪═══════════╡
│ Full model │ -31.8095 │ 5 │ - │ - │ - │
│ Fitted model │ -33.01 │ 2 │ 2.40102 │ 3 │ 0.493445 │
│ Reduced model │ -115.802 │ 1 │ 167.985 │ 4 │ 0 │
╘═══════════════╧══════════════════╧════════════╧════════════╧═════════════╧═══════════╛

# Liver, biliary hyperplasia, Ms, M, 18-mo, CRL (2024)

## Dataset

**Name:** Liver, biliary hyperplasia, Mm,. M, 18-mo

| Dose (mg/kg-day) | N | Incidence |
| --- | --- | --- |
| 0 | 44 | 0 |
| 0.05 | 43 | 0 |
| 0.1 | 43 | 0 |
| 0.5 | 42 | 0 |
| 5 | 34 | 7 |

## Settings

| Setting | Value |
| --- | --- |
| BMR | 10% Extra Risk |
| Confidence Level (one sided) | 0.95 |
| Maximum Multistage Degree | 3 |

## Maximum Likelihood Approach

| Model | BMDL | BMD | BMDU | *P*-Value | AIC | Scaled Residual at Control | Scaled Residual near BMD | Recommendation and Notes |
| --- | --- | --- | --- | --- | --- | --- | --- | --- |
| Hill | 1.936 | 4.768 | 4.989 | 1. | 40.575 | -0.001 | <0.001 | **Viable** |
| Gamma | 1.995 | 4.394 | 4.933 | 1. | 38.575 | -0.001 | <0.001 | **Viable** |
| LogLogistic | 1.936 | 4.768 | 4.978 | 1. | 38.575 | -0.001 | <0.001 | **Viable** |
| Multistage 1 | 1.535 | 2.7 | 5.408 | 0.866 | 38.901 | -0.001 | -0.91 | **Viable** |
| Multistage 2 | 2.05 | 3.405 | 4.819 | 0.999 | 36.777 | -0.001 | 0.038 | **Viable** |
| Multistage 3 | 2.135 | 3.853 | 4.857 | 1. | 36.594 | -0.001 | 0.004 | **Viable** |
| Weibull^ab^ | 2.019 | 4.787 | 4.975 | 1. | 36.575 | -0.001 | -0. | **Recommended - Lowest AIC** |
| Logistic | 3.658 | 4.746 | 4.973 | 1. | 36.575 | -0.001 | <0.001 | **Viable** |
| LogProbit | 1.678 | 4.58 | 5.015 | 1. | 38.575 | -0.001 | -0. | **Viable** |
| Probit | 3.353 | 4.575 | 5.003 | 1. | 38.575 | -0. | -0. | **Viable** |
| Quantal Linear | 1.535 | 2.7 | 5.405 | 0.866 | 38.901 | -0.001 | -0.91 | **Viable** |

^a^ BMDS recommended best fitting model

^b^ User selected best fitting model


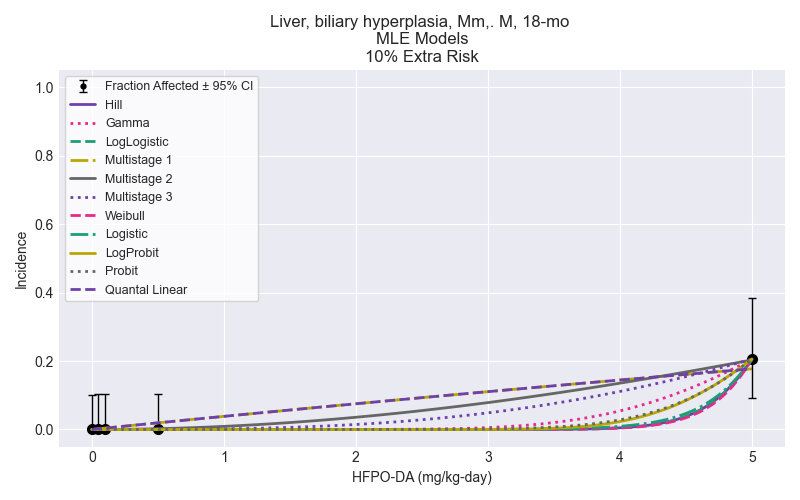


## Selected Model: Weibull


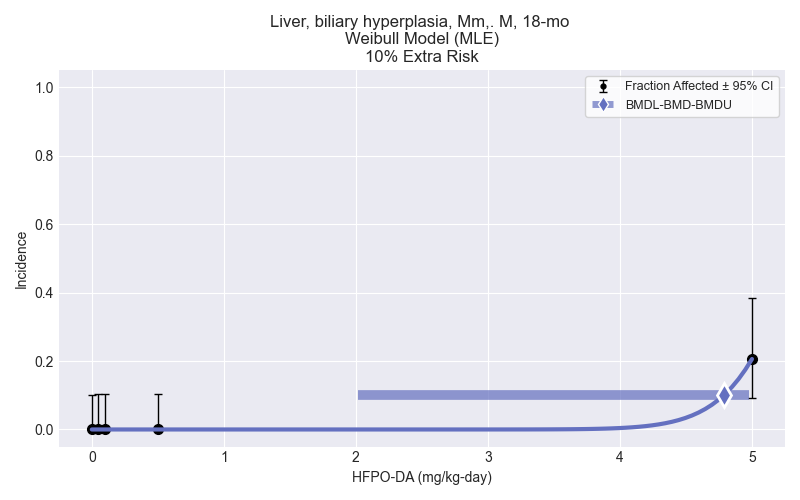


Weibull Model
══════════════════════════════

Version: pybmds 25.1 (bmdscore 25.1)

Input Summary:
╒══════════════════════════════╤════════════════════════╕
│ BMR │ 10% Extra Risk │
│ Confidence Level (one sided) │ 0.95 │
│ Modeling approach │ frequentist_restricted │
╘══════════════════════════════╧════════════════════════╛

Parameter Settings:
╒═════════════╤═══════════╤═════════╤═══════╕
│ Parameter │ Initial │ Min │ Max │
╞═════════════╪═══════════╪═════════╪═══════╡
│ g │ 0 │ -18 │ 18 │
│ a │ 1 │ 1 │ 18 │
│ b │ 1e-06 │ 1e-06 │ 100 │
╘═════════════╧═══════════╧═════════╧═══════╛

Modeling Summary:
╒════════════════╤═══════════════╕
│ BMD │ 4.78653 │
│ BMDL │ 2.0192 │
│ BMDU │ 4.9753 │
│ AIC │ 36.5746 │
│ Log-Likelihood │ -17.2873 │
│ P-Value │ 1 │
│ Overall d.f. │ 4 │
│ Chi² │ 2.61956e-06 │
╘════════════════╧═══════════════╛

Model Parameters:
╒════════════╤══════════════╤════════════╤══════════════╕
│ Variable │ Estimate │ On Bound │ Std Error │
╞════════════╪══════════════╪════════════╪══════════════╡
│ g │ 1.523e-08 │ yes │ Not Reported │
│ a │ 17.9446 │ no │ -9999 │
│ b │ 6.60633e-14 │ yes │ Not Reported │
╘════════════╧══════════════╧════════════╧══════════════╛
Standard errors estimates are not generated for parameters estimated on corresponding bounds,
although sampling error is present for all parameters, as a rule. Standard error estimates may not
be reliable as a basis for confidence intervals or tests when one or more parameters are on bounds.


Goodness of Fit:
╒════════╤════════╤════════════╤═════════════╤════════════╤═══════════════════╕
│ Dose │ Size │ Observed │ Expected │ Est Prob │ Scaled Residual │
╞════════╪════════╪════════════╪═════════════╪════════════╪═══════════════════╡
│ 0 │ 44 │ 0 │ 6.70119e-07 │ 1.523e-08 │ -0.000818608 │
│ 0.05 │ 43 │ 0 │ 6.54889e-07 │ 1.523e-08 │ -0.000809252 │
│ 0.1 │ 43 │ 0 │ 6.54889e-07 │ 1.523e-08 │ -0.000809252 │
│ 0.5 │ 42 │ 0 │ 6.39659e-07 │ 1.523e-08 │ -0.000799787 │
│ 5 │ 34 │ 7 │ 7 │ 0.205882 │ -5.98919e-08 │
╘════════╧════════╧════════════╧═════════════╧════════════╧═══════════════════╛

Analysis of Deviance:
╒═══════════════╤══════════════════╤════════════╤═════════════╤═════════════╤═════════════╕
│ Model │ Log-Likelihood │ # Params │ Deviance │ Test d.f. │ P-Value │
╞═══════════════╪══════════════════╪════════════╪═════════════╪═════════════╪═════════════╡
│ Full model │ -17.2873 │ 5 │ - │ - │ - │
│ Fitted model │ -17.2873 │ 1 │ 5.23911e-06 │ 4 │ 1 │
│ Reduced model │ -30.5535 │ 1 │ 26.5323 │ 4 │ 2.47106e-05 │
╘═══════════════╧══════════════════╧════════════╧═════════════╧═════════════╧═════════════╛

# Liver, Cystic focal degen, Rt, M, 24-mo, Rae (2015)

## Dataset

**Name:** Liver, Cystic focal degen, Rt, M, 24 mo

| Dose (mg/kg-day) | N | Incidence |
| --- | --- | --- |
| 0 | 70 | 24 |
| 0.1 | 70 | 24 |
| 1 | 70 | 19 |
| 50 | 70 | 42 |

## Settings

| Setting | Value |
| --- | --- |
| BMR | 10% Extra Risk |
| Confidence Level (one sided) | 0.95 |
| Maximum Multistage Degree | 3 |

## Maximum Likelihood Approach

| Model | BMDL | BMD | BMDU | *P*-Value | AIC | Scaled Residual at Control | Scaled Residual near BMD | Recommendation and Notes |
| --- | --- | --- | --- | --- | --- | --- | --- | --- |
| Hill | 1.029 | 38.987 | 46.999 | - | 365.204 | 0.427 | -0. | **Questionable** Zero degrees of freedom; saturated model BMD/BMDL ratio > 3.0 BMD/BMDL ratio > 20.0 |
| Gamma | 6.65 | 33.45 | 42.533 | 0.295 | 363.204 | 0.427 | -0. | **Viable** BMD/BMDL ratio > 3.0 |
| LogLogistic | 4.667 | 42.45 | 46.949 | 0.295 | 363.204 | 0.427 | -0. | **Viable** BMD/BMDL ratio > 3.0 |
| Multistage 1^a^ | 6.548 | 9.935 | 17.827 | 0.519 | 361.425 | 0.463 | -0.945 | **Viable** |
| Multistage 2 | 6.648 | 22.251 | 29.749 | 0.577 | 361.209 | 0.428 | -0.857 | **Viable** BMD/BMDL ratio > 3.0 |
| Multistage 3 | 6.649 | 29.144 | 35.367 | 0.578 | 361.205 | 0.427 | <0.001 | **Viable** BMD/BMDL ratio > 3.0 |
| Weibull^b^ | 6.65 | 43.436 | 47.258 | 0.578 | 361.204 | 0.427 | <0.001 | **Recommended - Lowest AIC** BMD/BMDL ratio > 3.0 |
| Logistic | 9.526 | 12.86 | 20.177 | 0.537 | 361.358 | 0.456 | -0.916 | **Viable** |
| LogProbit | 0.997 | 32.564 | 48.309 | 0.295 | 363.204 | 0.427 | -0. | **Questionable** BMD/BMDL ratio > 3.0 BMD/BMDL ratio > 20.0 |
| Probit | 9.431 | 12.701 | 20.025 | 0.536 | 361.361 | 0.456 | -0.917 | **Viable** |
| Quantal Linear | 6.548 | 9.935 | 17.825 | 0.519 | 361.425 | 0.463 | -0.945 | **Viable** |

^a^ Viable alternate; BMD/BMDL ratio <3; similar AIC and BMDL

^b^ BMDS recommended best fitting model


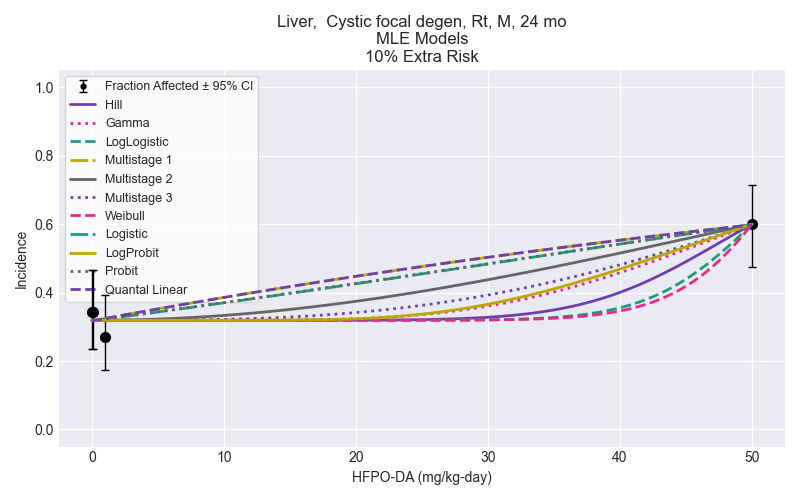


## Selected Model: Multistage 1


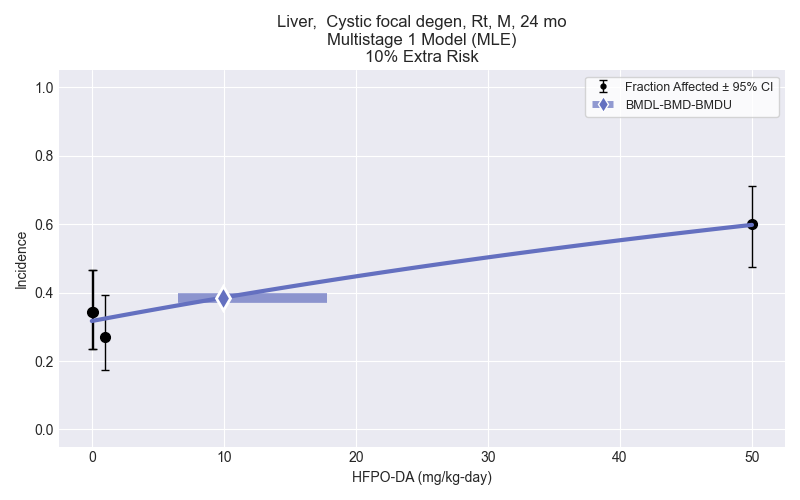


Multistage 1 Model
══════════════════════════════

Version: pybmds 25.1 (bmdscore 25.1)

Input Summary:
╒══════════════════════════════╤════════════════════════╕
│ BMR │ 10% Extra Risk │
│ Confidence Level (one sided) │ 0.95 │
│ Modeling approach │ frequentist_restricted │
│ Degree │ 1 │
╘══════════════════════════════╧════════════════════════╛

Parameter Settings:
╒═════════════╤═══════════╤═══════╤═══════╕
│ Parameter │ Initial │ Min │ Max │
╞═════════════╪═══════════╪═══════╪═══════╡
│ g │ 0 │ -18 │ 18 │
│ b1 │ 0 │ 0 │ 10000 │
╘═════════════╧═══════════╧═══════╧═══════╛

Modeling Summary:
╒════════════════╤═════════════╕
│ BMD │ 9.93508 │
│ BMDL │ 6.54798 │
│ BMDU │ 17.827 │
│ AIC │ 361.425 │
│ Log-Likelihood │ -178.713 │
│ P-Value │ 0.519214 │
│ Overall d.f. │ 2 │
│ Chi² │ 1.31088 │
╘════════════════╧═════════════╛

Model Parameters:
╒════════════╤════════════╤════════════╤═════════════╕
│ Variable │ Estimate │ On Bound │ Std Error │
╞════════════╪════════════╪════════════╪═════════════╡
│ g │ 0.317106 │ no │ 0.00892132 │
│ b1 │ 0.0106049 │ no │ 0.153008 │
╘════════════╧════════════╧════════════╧═════════════╛

Goodness of Fit:
╒════════╤════════╤════════════╤════════════╤════════════╤═══════════════════╕
│ Dose │ Size │ Observed │ Expected │ Est Prob │ Scaled Residual │
╞════════╪════════╪════════════╪════════════╪════════════╪═══════════════════╡
│ 0 │ 70 │ 24 │ 22.1974 │ 0.317106 │ 0.46299 │
│ 0.1 │ 70 │ 24 │ 22.2481 │ 0.31783 │ 0.449702 │
│ 1 │ 70 │ 19 │ 22.7017 │ 0.324309 │ -0.945135 │
│ 50 │ 70 │ 42 │ 41.8701 │ 0.598144 │ 0.0316802 │
╘════════╧════════╧════════════╧════════════╧════════════╧═══════════════════╛

Analysis of Deviance:
╒═══════════════╤══════════════════╤════════════╤════════════╤═════════════╤═════════════╕
│ Model │ Log-Likelihood │ # Params │ Deviance │ Test d.f. │ P-Value │
╞═══════════════╪══════════════════╪════════════╪════════════╪═════════════╪═════════════╡
│ Full model │ -178.046 │ 4 │ - │ - │ - │
│ Fitted model │ -178.713 │ 2 │ 1.33375 │ 2 │ 0.513309 │
│ Reduced model │ -187.16 │ 1 │ 18.2278 │ 3 │ 0.000394728 │
╘═══════════════╧══════════════════╧════════════╧════════════╧═════════════╧═════════════╛

# Liver, EPA constellation, Ms, M, Repro

## Dataset

**Name:** Liver, constellation, Ms, M, Repro

| Dose (mg/kg-day) | N | Incidence |
| --- | --- | --- |
| 0 | 25 | 1 |
| 0.1 | 24 | 1 |
| 0.5 | 24 | 13 |
| 5 | 24 | 24 |

## Settings

| Setting | Value |
| --- | --- |
| BMR | 10% Extra Risk |
| Confidence Level (one sided) | 0.95 |
| Maximum Multistage Degree | 3 |

## Maximum Likelihood Approach

| Model | BMDL | BMD | BMDU | *P*-Value | AIC | Scaled Residual at Control | Scaled Residual near BMD | Recommendation and Notes |
| --- | --- | --- | --- | --- | --- | --- | --- | --- |
| Hill | 0.108 | 0.338 | 0.412 | 0.977 | 55.816 | -0.02 | -0. | **Viable** BMD/BMDL ratio > 3.0 |
| Gamma | 0.087 | 0.266 | 0.376 | 1. | 55.815 | -0. | <0.001 | **Viable** BMD/BMDL ratio > 3.0 |
| LogLogistic | 0.108 | 0.338 | 0.412 | 0.977 | 55.816 | -0.02 | -0. | **Viable** BMD/BMDL ratio > 3.0 |
| Multistage 1 | 0.055 | 0.083 | 0.133 | 0.279 | 57.026 | 0.452 | -1.402 | **Viable** |
| Multistage 2^a^ | 0.081 | 0.189 | 0.249 | 0.905 | 54.026 | 0.248 | -0.368 | **Viable** |
| Multistage 3^b^ | 0.085 | 0.261 | 0.314 | 0.997 | 53.82 | 0.047 | -0.053 | **Recommended - Lowest AIC** BMD/BMDL ratio > 3.0 |
| Weibull | 0.084 | 0.202 | 0.275 | 0.717 | 55.951 | 0.205 | -0.29 | **Viable** |
| Logistic | 0.154 | 0.216 | 0.293 | 0.888 | 54.048 | 0.359 | -0.327 | **Viable** |
| LogProbit | 0.101 | 0.249 | 0.447 | 0.99 | 55.816 | -0.006 | 0.006 | **Viable** |
| Probit | 0.138 | 0.189 | 0.256 | 0.758 | 54.444 | 0.093 | -0.635 | **Viable** |
| Quantal Linear | 0.055 | 0.083 | 0.133 | 0.279 | 57.026 | 0.452 | -1.402 | **Viable** |

^a^ viable alternate; BMD/BMDL <3; similar AIC and BMDL

^b^ BMDS recommended best fitting model


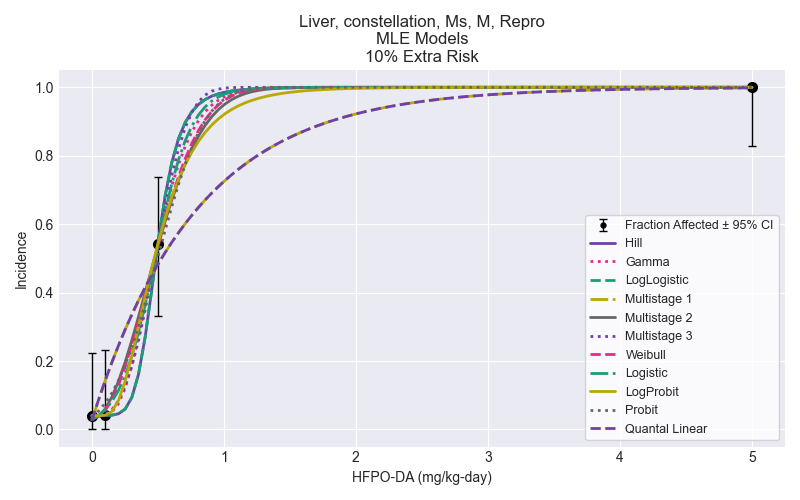


## Selected Model: Multistage 2


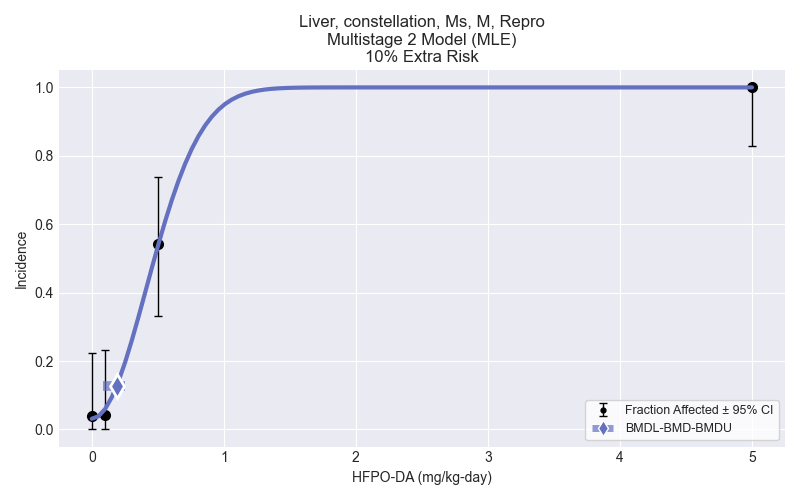


Multistage 2 Model
══════════════════════════════

Version: pybmds 25.1 (bmdscore 25.1)

Input Summary:
╒══════════════════════════════╤════════════════════════╕
│ BMR │ 10% Extra Risk │
│ Confidence Level (one sided) │ 0.95 │
│ Modeling approach │ frequentist_restricted │
│ Degree │ 2 │
╘══════════════════════════════╧════════════════════════╛

Parameter Settings:
╒═════════════╤═══════════╤═══════╤═══════╕
│ Parameter │ Initial │ Min │ Max │
╞═════════════╪═══════════╪═══════╪═══════╡
│ g │ 0 │ -18 │ 18 │
│ b1 │ 0 │ 0 │ 10000 │
│ b2 │ 0 │ 0 │ 10000 │
╘═════════════╧═══════════╧═══════╧═══════╛

Modeling Summary:
╒════════════════╤═════════════╕
│ BMD │ 0.189379 │
│ BMDL │ 0.0813716 │
│ BMDU │ 0.249072 │
│ AIC │ 54.0259 │
│ Log-Likelihood │ -25.0129 │
│ P-Value │ 0.904625 │
│ Overall d.f. │ 2 │
│ Chi² │ 0.20047 │
╘════════════════╧═════════════╛

Model Parameters:
╒════════════╤════════════╤════════════╤══════════════╕
│ Variable │ Estimate │ On Bound │ Std Error │
╞════════════╪════════════╪════════════╪══════════════╡
│ g │ 0.0313669 │ no │ 0.0568442 │
│ b1 │ 4.5008e-17 │ yes │ Not Reported │
│ b2 │ 2.93774 │ no │ 21.4496 │
╘════════════╧════════════╧════════════╧══════════════╛
Standard errors estimates are not generated for parameters estimated on corresponding bounds,
although sampling error is present for all parameters, as a rule. Standard error estimates may not
be reliable as a basis for confidence intervals or tests when one or more parameters are on bounds.


Goodness of Fit:
╒════════╤════════╤════════════╤════════════╤════════════╤═══════════════════╕
│ Dose │ Size │ Observed │ Expected │ Est Prob │ Scaled Residual │
╞════════╪════════╪════════════╪════════════╪════════════╪═══════════════════╡
│ 0 │ 25 │ 1 │ 0.784171 │ 0.0313669 │ 0.247642 │
│ 0.1 │ 24 │ 1 │ 1.42581 │ 0.0594088 │ -0.367694 │
│ 0.5 │ 24 │ 13 │ 12.8465 │ 0.535272 │ 0.0628072 │
│ 5 │ 24 │ 24 │ 24 │ 1 │ 0 │
╘════════╧════════╧════════════╧════════════╧════════════╧═══════════════════╛

Analysis of Deviance:
╒═══════════════╤══════════════════╤════════════╤════════════╤═════════════╤═══════════╕
│ Model │ Log-Likelihood │ # Params │ Deviance │ Test d.f. │ P-Value │
╞═══════════════╪══════════════════╪════════════╪════════════╪═════════════╪═══════════╡
│ Full model │ -24.9076 │ 4 │ - │ - │ - │
│ Fitted model │ -25.0129 │ 2 │ 0.210598 │ 2 │ 0.900055 │
│ Reduced model │ -65.3624 │ 1 │ 80.9095 │ 3 │ 0 │
╘═══════════════╧══════════════════╧════════════╧════════════╧═════════════╧═══════════╛

# Liver, EPA constellation, Ms, F, Repro

## Dataset

**Name:** Liver, constellation, Ms, F, Repro

| Dose (mg/kg-day) | N | Incidence |
| --- | --- | --- |
| 0 | 24 | 2 |
| 0.1 | 22 | 3 |
| 0.5 | 24 | 17 |
| 5 | 24 | 24 |

## Settings

| Setting | Value |
| --- | --- |
| BMR | 10% Extra Risk |
| Confidence Level (one sided) | 0.95 |
| Maximum Multistage Degree | 3 |

## Maximum Likelihood Approach

| Model | BMDL | BMD | BMDU | *P*-Value | AIC | Scaled Residual at Control | Scaled Residual near BMD | Recommendation and Notes |
| --- | --- | --- | --- | --- | --- | --- | --- | --- |
| Hill | 0.054 | 0.144 | 0.379 | 0.804 | 66.371 | -0.077 | 0.108 | **Viable** |
| Gamma | 0.041 | 0.132 | 0.325 | 1. | 66.268 | <0.001 | -0. | **Viable** BMD/BMDL ratio > 3.0 |
| LogLogistic | 0.054 | 0.144 | 0.379 | 0.804 | 66.371 | -0.077 | 0.108 | **Viable** |
| Multistage 1 | 0.035 | 0.052 | 0.084 | 0.448 | 66.021 | 0.393 | -1.087 | **Viable** |
| Multistage 2 | 0.041 | 0.139 | 0.196 | 1. | 66.268 | -0. | <0.001 | **Viable** BMD/BMDL ratio > 3.0 |
| Multistage 3 | 0.041 | 0.139 | 0.268 | - | 68.268 | <0.001 | -0. | **Questionable** Zero degrees of freedom; saturated model BMD/BMDL ratio > 3.0 |
| Weibull | 0.041 | 0.136 | 0.211 | 1. | 66.268 | <0.001 | -0. | **Viable** BMD/BMDL ratio > 3.0 |
| Logistic^ab^ | 0.094 | 0.132 | 0.183 | 0.993 | 64.283 | 0.085 | -0.086 | **Recommended - Lowest AIC** |
| LogProbit | 0.054 | 0.128 | 0.425 | 0.932 | 66.282 | -0.015 | 0.024 | **Viable** |
| Probit | 0.089 | 0.12 | 0.163 | 0.888 | 64.514 | -0.101 | -0.328 | **Viable** |
| Quantal Linear | 0.035 | 0.052 | 0.084 | 0.448 | 66.021 | 0.393 | -1.087 | **Viable** |

^a^ BMDS recommended best fitting model

^b^ User selected best fitting model


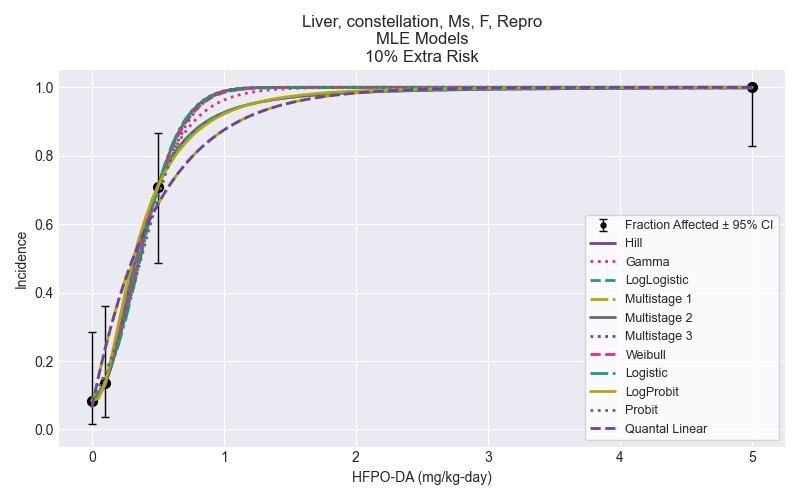


## Selected Model: Logistic


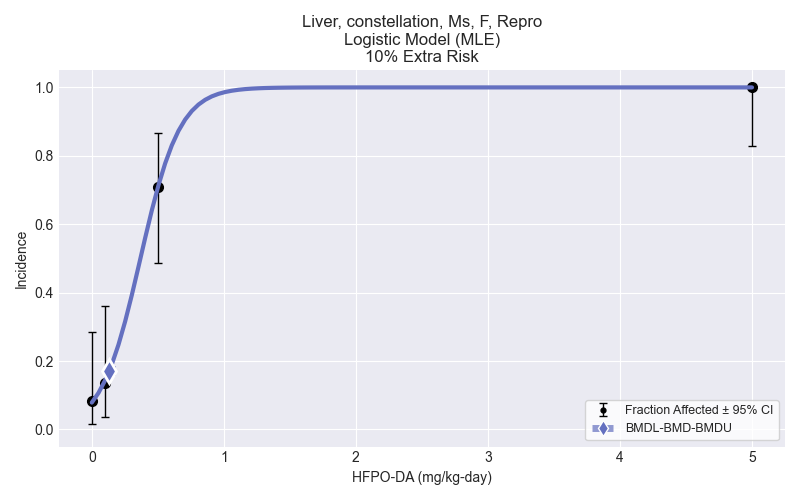


Logistic Model
══════════════════════════════

Version: pybmds 25.1 (bmdscore 25.1)

Input Summary:
╒══════════════════════════════╤══════════════════════════╕
│ BMR │ 10% Extra Risk │
│ Confidence Level (one sided) │ 0.95 │
│ Modeling approach │ frequentist_unrestricted │
╘══════════════════════════════╧══════════════════════════╛

Parameter Settings:
╒═════════════╤═══════════╤═══════╤═══════╕
│ Parameter │ Initial │ Min │ Max │
╞═════════════╪═══════════╪═══════╪═══════╡
│ a │ 0 │ -18 │ 18 │
│ b │ 0 │ 0 │ 100 │
╘═════════════╧═══════════╧═══════╧═══════╛

Modeling Summary:
╒════════════════╤═════════════╕
│ BMD │ 0.131761 │
│ BMDL │ 0.0940909 │
│ BMDU │ 0.183369 │
│ AIC │ 64.283 │
│ Log-Likelihood │ -30.1415 │
│ P-Value │ 0.992608 │
│ Overall d.f. │ 2 │
│ Chi² │ 0.0148397 │
╘════════════════╧═════════════╛

Model Parameters:
╒════════════╤════════════╤════════════╤═════════════╕
│ Variable │ Estimate │ On Bound │ Std Error │
╞════════════╪════════════╪════════════╪═════════════╡
│ a │ -2.46102 │ no │ 0.540289 │
│ b │ 6.68528 │ no │ 1.47034 │
╘════════════╧════════════╧════════════╧═════════════╛

Goodness of Fit:
╒════════╤════════╤════════════╤════════════╤════════════╤═══════════════════╕
│ Dose │ Size │ Observed │ Expected │ Est Prob │ Scaled Residual │
╞════════╪════════╪════════════╪════════════╪════════════╪═══════════════════╡
│ 0 │ 24 │ 2 │ 1.88728 │ 0.0786367 │ 0.0854792 │
│ 0.1 │ 22 │ 3 │ 3.1409 │ 0.142768 │ -0.0858674 │
│ 0.5 │ 24 │ 17 │ 16.9718 │ 0.707159 │ 0.0126402 │
│ 5 │ 24 │ 24 │ 24 │ 1 │ 9.23391e-07 │
╘════════╧════════╧════════════╧════════════╧════════════╧═══════════════════╛

Analysis of Deviance:
╒═══════════════╤══════════════════╤════════════╤════════════╤═════════════╤═════════════╕
│ Model │ Log-Likelihood │ # Params │ Deviance │ Test d.f. │ P-Value │
╞═══════════════╪══════════════════╪════════════╪════════════╪═════════════╪═════════════╡
│ Full model │ -30.1341 │ 4 │ - │ - │ - │
│ Fitted model │ -30.1415 │ 2 │ 0.0148047 │ 2 │ 0.992625 │
│ Reduced model │ -65.1346 │ 1 │ 70.0009 │ 3 │ 4.21885e-15 │
╘═══════════════╧══════════════════╧════════════╧════════════╧═════════════╧═════════════╛

# Adrenal gland, cortical hypertrophy, Ms, M, 18-mo, CRL (2024)

## Dataset

**Name:** Adrenal gland, cortical hypertrophy, Ms, M, 18-mo

| Dose (mg/kg-day) | N | Incidence |
| --- | --- | --- |
| 0 | 43 | 10 |
| 0.05 | 41 | 10 |
| 0.1 | 43 | 6 |
| 0.5 | 42 | 3 |
| 5 | 34 | 27 |

## Settings

| Setting | Value |
| --- | --- |
| BMR | 10% Extra Risk |
| Confidence Level (one sided) | 0.95 |
| Maximum Multistage Degree | 3 |

## Maximum Likelihood Approach

| Model | BMDL | BMD | BMDU | *P*-Value | AIC | Scaled Residual at Control | Scaled Residual near BMD | Recommendation and Notes |
| --- | --- | --- | --- | --- | --- | --- | --- | --- |
| Hill | 0.544 | 4.081 | 4.373 | 0.015 | 197.517 | 1.06 | <0.001 | **Questionable** Goodness of fit p-value < 0.1 BMD/BMDL ratio > 3.0 |
| Gamma | 0.795 | 3.101 | 3.428 | 0.052 | 195.517 | 1.06 | <0.001 | **Questionable** Goodness of fit p-value < 0.1 BMD/BMDL ratio > 3.0 |
| LogLogistic | 0.761 | 4.15 | 4.336 | 0.052 | 195.517 | 1.06 | <0.001 | **Questionable** Goodness of fit p-value < 0.1 BMD/BMDL ratio > 3.0 |
| Multistage 1 | 0.322 | 0.466 | 0.717 | 0.009 | 200.5 | 1.273 | -2.682 | **Questionable** \|Residual near BMD\| > 2.0 Goodness of fit p-value < 0.1 |
| Multistage 2 | 0.763 | 1.389 | 1.722 | 0.091 | 194.211 | 1.08 | -1.856 | **Questionable** Goodness of fit p-value < 0.1 |
| Multistage 3^a^ | 0.871 | 2.116 | 2.442 | 0.113 | 193.586 | 1.062 | -1.736 | **Viable** |
| Weibull^b^ | 0.823 | 4.247 | 4.449 | 0.116 | 193.517 | 1.06 | <0.001 | **Recommended - Lowest AIC** BMD/BMDL ratio > 3.0 |
| Logistic | 0.705 | 0.903 | 1.166 | 0.039 | 196.338 | 1.274 | -2.136 | **Questionable** \|Residual near BMD\| > 2.0 Goodness of fit p-value < 0.1 |
| LogProbit | 0.713 | 3.032 | 4.594 | 0.052 | 195.517 | 1.06 | <0.001 | **Questionable** Goodness of fit p-value < 0.1 BMD/BMDL ratio > 3.0 |
| Probit | 0.682 | 0.854 | 1.097 | 0.036 | 196.57 | 1.271 | -2.178 | **Questionable** \|Residual near BMD\| > 2.0 Goodness of fit p-value < 0.1 |
| Quantal Linear | 0.322 | 0.466 | 0.717 | 0.009 | 200.5 | 1.273 | -2.682 | **Questionable** \|Residual near BMD\| > 2.0 Goodness of fit p-value < 0.1 |

^a^ Viable alternative; BMD/BMDL ration < 3; avoids steep turn of Weibull model; similar AIC and BMDL

^b^ BMDS recommended best fitting model


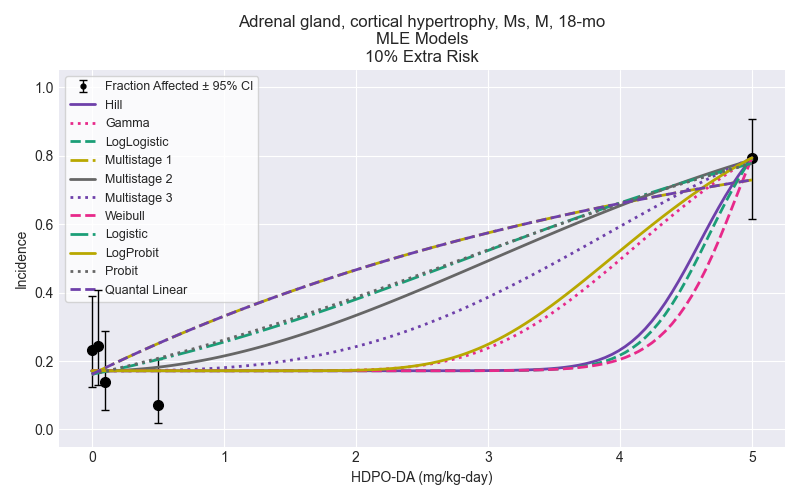


## Selected Model: Multistage 3


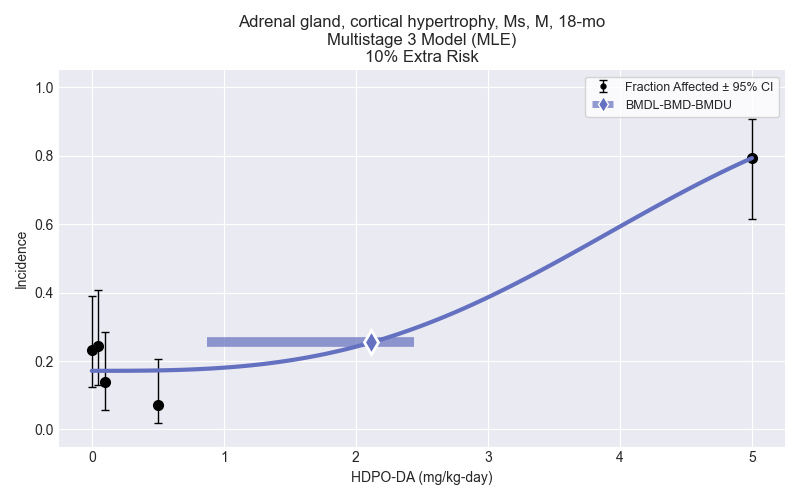


Multistage 3 Model
══════════════════════════════

Version: pybmds 25.1 (bmdscore 25.1)

Input Summary:
╒══════════════════════════════╤════════════════════════╕
│ BMR │ 10% Extra Risk │
│ Confidence Level (one sided) │ 0.95 │
│ Modeling approach │ frequentist_restricted │
│ Degree │ 3 │
╘══════════════════════════════╧════════════════════════╛

Parameter Settings:
╒═════════════╤═══════════╤═══════╤═══════╕
│ Parameter │ Initial │ Min │ Max │
╞═════════════╪═══════════╪═══════╪═══════╡
│ g │ 0 │ -18 │ 18 │
│ b1 │ 0 │ 0 │ 10000 │
│ b2 │ 0 │ 0 │ 10000 │
│ b3 │ 0 │ 0 │ 10000 │
╘═════════════╧═══════════╧═══════╧═══════╛

Modeling Summary:
╒════════════════╤════════════╕
│ BMD │ 2.11629 │
│ BMDL │ 0.870804 │
│ BMDU │ 2.44227 │
│ AIC │ 193.586 │
│ Log-Likelihood │ -94.7929 │
│ P-Value │ 0.113434 │
│ Overall d.f. │ 3 │
│ Chi² │ 5.9628 │
╘════════════════╧════════════╛

Model Parameters:
╒════════════╤═════════════╤════════════╤══════════════╕
│ Variable │ Estimate │ On Bound │ Std Error │
╞════════════╪═════════════╪════════════╪══════════════╡
│ g │ 0.171502 │ no │ 0.0125758 │
│ b1 │ 2.52427e-17 │ yes │ Not Reported │
│ b2 │ 2.17301e-16 │ yes │ Not Reported │
│ b3 │ 0.0111161 │ no │ 0.337781 │
╘════════════╧═════════════╧════════════╧══════════════╛
Standard errors estimates are not generated for parameters estimated on corresponding bounds,
although sampling error is present for all parameters, as a rule. Standard error estimates may not
be reliable as a basis for confidence intervals or tests when one or more parameters are on bounds.


Goodness of Fit:
╒════════╤════════╤════════════╤════════════╤════════════╤═══════════════════╕
│ Dose │ Size │ Observed │ Expected │ Est Prob │ Scaled Residual │
╞════════╪════════╪════════════╪════════════╪════════════╪═══════════════════╡
│ 0 │ 43 │ 10 │ 7.37459 │ 0.171502 │ 1.06214 │
│ 0.05 │ 41 │ 10 │ 7.03163 │ 0.171503 │ 1.22983 │
│ 0.1 │ 43 │ 6 │ 7.37499 │ 0.171511 │ -0.556255 │
│ 0.5 │ 42 │ 3 │ 7.2514 │ 0.172652 │ -1.73571 │
│ 5 │ 34 │ 27 │ 26.9804 │ 0.793542 │ 0.00829481 │
╘════════╧════════╧════════════╧════════════╧════════════╧═══════════════════╛

Analysis of Deviance:
╒═══════════════╤══════════════════╤════════════╤════════════╤═════════════╤═════════════╕
│ Model │ Log-Likelihood │ # Params │ Deviance │ Test d.f. │ P-Value │
╞═══════════════╪══════════════════╪════════════╪════════════╪═════════════╪═════════════╡
│ Full model │ -91.5698 │ 5 │ - │ - │ - │
│ Fitted model │ -94.7929 │ 2 │ 6.44626 │ 3 │ 0.0918061 │
│ Reduced model │ -119.568 │ 1 │ 55.9955 │ 4 │ 2.00951e-11 │
╘═══════════════╧══════════════════╧════════════╧════════════╧═════════════╧═════════════╛

# Serum Triglyceride, Ms., M Offspring, Cope et al. (2021)

## Dataset

**Name:** Serum Triglyceride, Ms., M Offspring, Cope et al. (2021)

| Dose (mg/kg-day) | N | Mean | Std. Dev. |
| --- | --- | --- | --- |
| 0 | 7 | 174.6 | 67.2 |
| 0.2 | 7 | 124 | 38 |
| 1 | 7 | 114.9 | 43.5 |
| 2 | 7 | 106.6 | 27.6 |

Test 1 Dose Response: 0.0219

Test 2 Homogeneity of Variance: 0.1221

Test 3 Variance Model Selection: 0.1221

## Settings

| Setting | Value |
| --- | --- |
| BMR | 1.0 Standard Deviation |
| Distribution | Normal + Constant variance |
| Adverse Direction | Down (↓) |
| Maximum Polynomial Degree | 3 |
| Confidence Level (one sided) | 0.95 |

## Maximum Likelihood Approach

| Model | BMDL | BMD | BMDU | *P*-Value | AIC | Scaled Residual at Control | Scaled Residual near BMD | Recommendation and Notes |
| --- | --- | --- | --- | --- | --- | --- | --- | --- |
| Exponential 3^ab^ | 0.806 | 1.645 | 7.27 | 0.144 | 299.914 | 1.268 | 0.463 | **Recommended - Lowest AIC** |
| Exponential 5 | 0.013 | 0.143 | - | 0.719 | 298.169 | <0.001 | -0.002 | **Questionable** lowest dose/BMDL ratio > 3.0 lowest dose/BMDL ratio > 10.0 BMD/BMDL ratio > 3.0 |
| Hill | <0.001 | 0.123 | - | 0.786 | 298.114 | 0.002 | -0.037 | **Questionable** lowest dose/BMDL ratio > 3.0 lowest dose/BMDL ratio > 10.0 BMD/BMDL ratio > 3.0 BMD/BMDL ratio > 20.0 |
| Polynomial 2 | 1.038 | 1.821 | 7.26 | 0.123 | 300.228 | 1.385 | 0.405 | **Viable** |
| Polynomial 3 | 1.038 | 1.799 | 7.263 | 0.123 | 300.228 | 1.37 | 0.426 | **Viable** |
| Power | 1.038 | 1.817 | 7.261 | 0.123 | 300.228 | 1.383 | 0.408 | **Viable** |
| Linear | 1.038 | 1.817 | 7.261 | 0.123 | 300.228 | 1.383 | 0.408 | **Viable** |

^a^ BMDS recommended best fitting model

^b^ User selected best fitting model


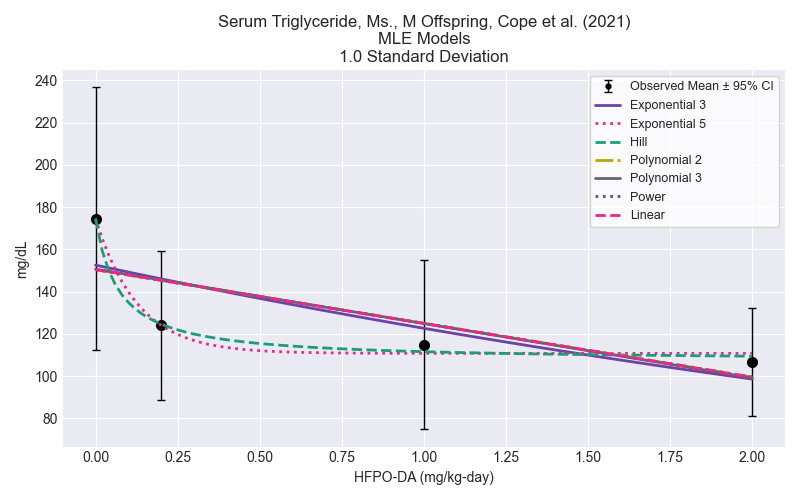


## Selected Model: Exponential 3


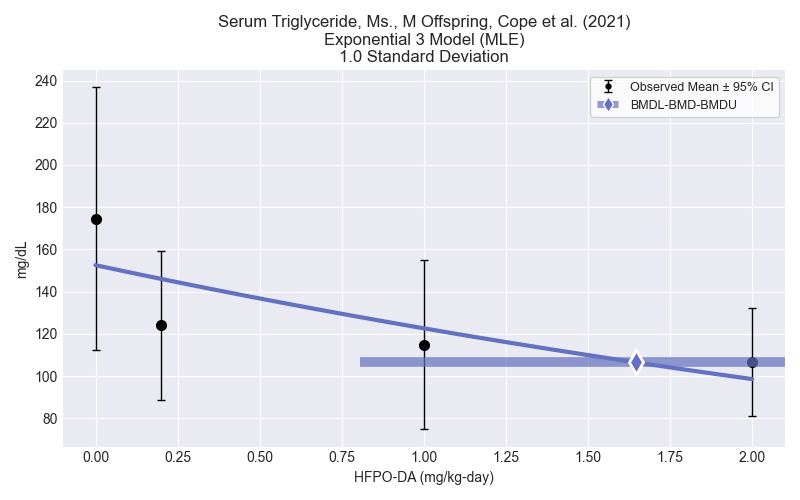


Exponential 3 Model
══════════════════════════════

Version: pybmds 25.1 (bmdscore 25.1)

Input Summary:
╒══════════════════════════════╤════════════════════════════╕
│ BMR │ 1.0 Standard Deviation │
│ Distribution │ Normal + Constant variance │
│ Modeling Direction │ Down (↓) │
│ Confidence Level (one sided) │ 0.95 │
│ Modeling Approach │ MLE │
╘══════════════════════════════╧════════════════════════════╛

Parameter Settings:
╒═════════════╤═══════════╤═══════╤═══════╕
│ Parameter │ Initial │ Min │ Max │
╞═════════════╪═══════════╪═══════╪═══════╡
│ a │ 0 │ 0 │ 100 │
│ b │ 0 │ 0 │ 100 │
│ c │ 0 │ -20 │ 0 │
│ d │ 1 │ 1 │ 18 │
│ log-alpha │ 0 │ -18 │ 18 │
╘═════════════╧═══════════╧═══════╧═══════╛

Modeling Summary:
╒════════════════╤═════════════╕
│ BMD │ 1.64481 │
│ BMDL │ 0.806237 │
│ BMDU │ 7.27 │
│ AIC │ 299.914 │
│ Log-Likelihood │ -146.957 │
│ P-Value │ 0.1441 │
│ Model d.f. │ 2 │
╘════════════════╧═════════════╛

Model Parameters:
╒════════════╤════════════╤════════════╤══════════════╕
│ Variable │ Estimate │ On Bound │ Std Error │
╞════════════╪════════════╪════════════╪══════════════╡
│ a │ 152.537 │ no │ 13.1331 │
│ b │ 0.218444 │ no │ 0.0988134 │
│ d │ 1 │ yes │ Not Reported │
│ log-alpha │ 7.65906 │ no │ 0.267261 │
╘════════════╧════════════╧════════════╧══════════════╛
Standard errors estimates are not generated for parameters estimated on corresponding bounds,
although sampling error is present for all parameters, as a rule. Standard error estimates may not
be reliable as a basis for confidence intervals or tests when one or more parameters are on bounds.


Goodness of Fit:
╒════════╤═════╤═══════════════╤═════════════════════╤═══════════════════╕
│ Dose │ N │ Sample Mean │ Model Fitted Mean │ Scaled Residual │
╞════════╪═════╪═══════════════╪═════════════════════╪═══════════════════╡
│ 0 │ 7 │ 174.6 │ 152.537 │ 1.26788 │
│ 0.2 │ 7 │ 124 │ 146.016 │ -1.26515 │
│ 1 │ 7 │ 114.9 │ 122.604 │ -0.44272 │
│ 2 │ 7 │ 106.6 │ 98.5454 │ 0.462862 │
╘════════╧═════╧═══════════════╧═════════════════════╧═══════════════════╛
╒════════╤═════╤═════════════╤═══════════════════╕
│ Dose │ N │ Sample SD │ Model Fitted SD │
╞════════╪═════╪═════════════╪═══════════════════╡
│ 0 │ 7 │ 67.2 │ 46.0408 │
│ 0.2 │ 7 │ 38 │ 46.0408 │
│ 1 │ 7 │ 43.5 │ 46.0408 │
│ 2 │ 7 │ 27.6 │ 46.0408 │
╘════════╧═════╧═════════════╧═══════════════════╛

Likelihoods:
╒═════════╤══════════════════╤════════════╤═════════╕
│ Model │ Log-Likelihood │ # Params │ AIC │
╞═════════╪══════════════════╪════════════╪═════════╡
│ A1 │ -145.02 │ 5 │ 300.04 │
│ A2 │ -142.123 │ 8 │ 300.246 │
│ A3 │ -145.02 │ 5 │ 300.04 │
│ fitted │ -146.957 │ 3 │ 299.914 │
│ reduced │ -149.522 │ 2 │ 303.044 │
╘═════════╧══════════════════╧════════════╧═════════╛

Tests of Mean and Variance Fits:
╒════════╤══════════════════════════════╤═════════════╤═══════════╕
│ Name │ -2 * Log(Likelihood Ratio) │ Test d.f. │ P-Value │
╞════════╪══════════════════════════════╪═════════════╪═══════════╡
│ Test 1 │ 14.7984 │ 6 │ 0.0218841 │
│ Test 2 │ 5.7936 │ 3 │ 0.122095 │
│ Test 3 │ 5.7936 │ 3 │ 0.122095 │
│ Test 4 │ 3.8745 │ 2 │ 0.1441 │
╘════════╧══════════════════════════════╧═════════════╧═══════════╛
Test 1: Test the null hypothesis that responses and variances don't differ among dose levels
(A2 vs R). If this test fails to reject the null hypothesis (p-value > 0.05), there may not be
a dose-response.

Test 2: Test the null hypothesis that variances are homogenous (A1 vs A2). If this test fails to
reject the null hypothesis (p-value > 0.05), the simpler constant variance model may be appropriate.

Test 3: Test the null hypothesis that the variances are adequately modeled (A3 vs A2). If this test
fails to reject the null hypothesis (p-value > 0.05), it may be inferred that the variances have
been modeled appropriately.

Test 4: Test the null hypothesis that the model for the mean fits the data (Fitted vs A3). If this
test fails to reject the null hypothesis (p-value > 0.1), the user has support for use of the
selected model.

## Serum Triglyceride, Ms., M Offspring, Cope et al. (2021)

## Dataset

**Name:** Serum Triglyceride, Ms., M Offspring, Cope et al. (2021)

| Dose (mg/kg-day) | N | Mean | Std. Dev. |
| --- | --- | --- | --- |
| 0 | 7 | 174.6 | 67.2 |
| 0.2 | 7 | 124 | 38 |
| 1 | 7 | 114.9 | 43.5 |
| 2 | 7 | 106.6 | 27.6 |

## Settings

| Setting | Value |
| --- | --- |
| BMR | 1.0 Standard Deviation |
| Distribution | Normal + Nonconstant variance |
| Adverse Direction | Down (↓) |
| Maximum Polynomial Degree | 3 |
| Confidence Level (one sided) | 0.95 |

## Maximum Likelihood Approach

| Model | BMDL | BMD | BMDU | *P*-Value | AIC | Scaled Residual at Control | Scaled Residual near BMD | Recommendation and Notes |
| --- | --- | --- | --- | --- | --- | --- | --- | --- |
| Exponential 3 | - | - | - | - | - | 0 | - | **Unusable** Did not successfully execute. |
| Exponential 5 | 0.018 | 0.301 | 0.331 | - | 297.959 | -0.031 | 0.135 | **Questionable** lowest dose/BMDL ratio > 3.0 lowest dose/BMDL ratio > 10.0 Zero degrees of freedom; saturated model BMD/BMDL ratio > 3.0 |
| Hill | <0.001 | 0.682 | - | 0.348 | 295.749 | -0.016 | 0.259 | **Questionable** lowest dose/BMDL ratio > 3.0 lowest dose/BMDL ratio > 10.0 BMD/BMDL ratio > 3.0 BMD/BMDL ratio > 20.0 |
| Polynomial 2^a^ | 1.445 | 2.807 | 5.278 | 0.11 | 297.279 | 1.316 | 0.249 | **Recommended - Lowest AIC** BMD/highest dose ratio > 1.0 |
| Polynomial 3 | 1.445 | 2.866 | 5.28 | 0.11 | 297.281 | 1.333 | 0.224 | **Viable** BMD/highest dose ratio > 1.0 |
| Power | 1.445 | 2.734 | 2.79 | 0.11 | 297.283 | 1.3 | 0.28 | **Viable** BMD/highest dose ratio > 1.0 |
| Linear | 1.445 | 2.807 | 5.278 | 0.11 | 297.279 | 1.316 | 0.249 | **Viable** BMD/highest dose ratio > 1.0 |

^a^ BMDS recommended best fitting model

## Selected Model

No model was selected as a best-fitting model.

## Serum Triglyceride, Ms., M Offspring, Cope et al. (2021)

## Dataset

**Name:** Serum Triglyceride, Ms., M Offspring, Cope et al. (2021)

| Dose (mg/kg-day) | N | Mean | Std. Dev. |
| --- | --- | --- | --- |
| 0 | 7 | 174.6 | 67.2 |
| 0.2 | 7 | 124 | 38 |
| 1 | 7 | 114.9 | 43.5 |
| 2 | 7 | 106.6 | 27.6 |

Test 1 Dose Response: 0.1372

Test 2 Homogeneity of Variance: 0.732

Test 3 Variance Model Selection: 0.732

## Settings

| Setting | Value |
| --- | --- |
| BMR | 1.0 Standard Deviation |
| Distribution | Lognormal + Constant variance |
| Adverse Direction | Down (↓) |
| Maximum Polynomial Degree | 3 |
| Confidence Level (one sided) | 0.95 |

## Maximum Likelihood Approach

| Model | BMDL | BMD | BMDU | *P*-Value | AIC | Scaled Residual at Control | Scaled Residual near BMD | Recommendation and Notes |
| --- | --- | --- | --- | --- | --- | --- | --- | --- |
| Exponential 3 | 1.037 | 2.695 | 7.209 | 0.166 | 290.636 | 65.9 | 15.939 | **Questionable** \|Residual near BMD\| > 2.0 Residual at control > 2.0 BMD/highest dose ratio > 1.0 |
| Exponential 5 | 0.495 | 0.506 | - | - | 291.101 | 21.436 | 9.662 | **Questionable** \|Residual near BMD\| > 2.0 Zero degrees of freedom; saturated model Residual at control > 2.0 |


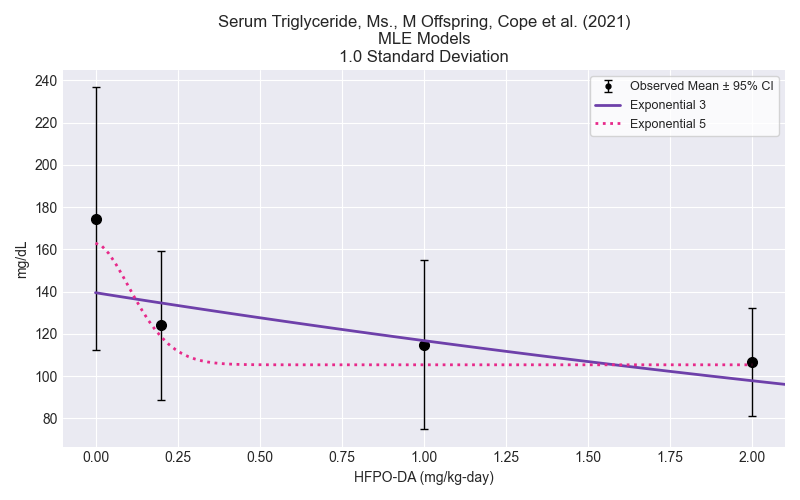


## Selected Model

No model was selected as a best-fitting model.

# Serum Triglyceride, Ms., F Offspring, Cope et al. (2021)

## Dataset

**Name:** Serum Triglyceride, Ms., F Offspring, Cope et al. (2021)

| Dose (mg/kg-day) | N | Mean | Std. Dev. |
| --- | --- | --- | --- |
| 0 | 7 | 194.4 | 44.8 |
| 0.2 | 7 | 112.2 | 30.8 |
| 1 | 7 | 106 | 26.5 |
| 2 | 7 | 109.9 | 26.8 |

Test 1 Dose Response: <0.0001

Test 2 Homogeneity of Variance: 0.4235

Test 3 Variance Model Selection: 0.4235

## Settings

| Setting | Value |
| --- | --- |
| BMR | 1.0 Standard Deviation |
| Distribution | Normal + Constant variance |
| Adverse Direction | Down (↓) |
| Maximum Polynomial Degree | 3 |
| Confidence Level (one sided) | 0.95 |

## Maximum Likelihood Approach

| Model | BMDL | BMD | BMDU | *P*-Value | AIC | Scaled Residual at Control | Scaled Residual near BMD | Recommendation and Notes |
| --- | --- | --- | --- | --- | --- | --- | --- | --- |
| Exponential 3 | 0.651 | 1.213 | 3.593 | <0.001 | 294.577 | 2.371 | -0.978 | **Questionable** Residual at control > 2.0 Goodness of fit p-value < 0.1 |
| Exponential 5 | 0.006 | 0.046 | 0.191 | - | 281.135 | <0.001 | <0.001 | **Questionable** lowest dose/BMDL ratio > 3.0 lowest dose/BMDL ratio > 10.0 lowest dose/BMD ratio > 3.0 Zero degrees of freedom; saturated model BMD/BMDL ratio > 3.0 |
| Hill | 0.001 | 0.152 | 0.179 | - | 281.135 | <0.001 | <0.001 | **Questionable** lowest dose/BMDL ratio > 3.0 lowest dose/BMDL ratio > 10.0 Zero degrees of freedom; saturated model BMD/BMDL ratio > 3.0 BMD/BMDL ratio > 20.0 |
| Polynomial 2 | 0.918 | 1.674 | 4.238 | <0.001 | 295.529 | 2.706 | 0.607 | **Questionable** Residual at control > 2.0 Goodness of fit p-value < 0.1 |
| Polynomial 3 | 0.923 | 1.534 | 4.143 | <0.001 | 295.459 | 2.585 | 0.781 | **Questionable** Residual at control > 2.0 Goodness of fit p-value < 0.1 |
| Power | 0.923 | 1.512 | 4.141 | <0.001 | 295.457 | 2.568 | 0.811 | **Questionable** Residual at control > 2.0 Goodness of fit p-value < 0.1 |
| Linear | 0.923 | 1.512 | 4.141 | <0.001 | 295.457 | 2.568 | 0.811 | **Questionable** Residual at control > 2.0 Goodness of fit p-value < 0.1 |


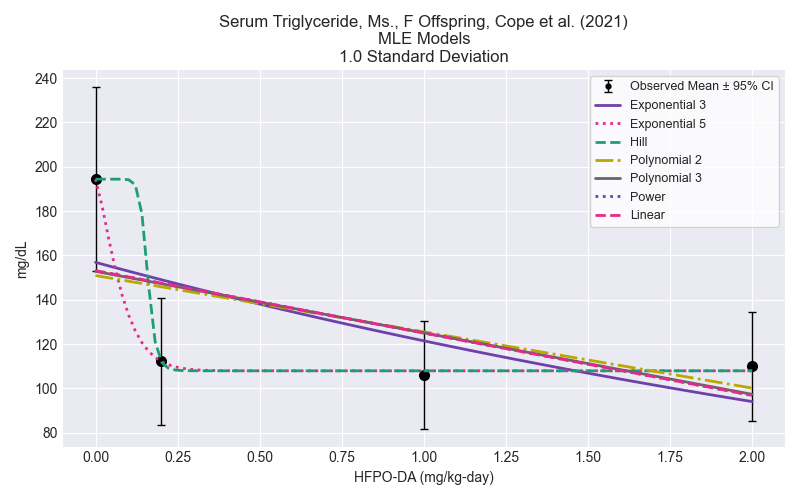


## Selected Model

No model was selected as a best-fitting model.

## Serum Triglyceride, Ms., F Offspring, Cope et al. (2021)

## Dataset

**Name:** Serum Triglyceride, Ms., F Offspring, Cope et al. (2021)

| Dose (mg/kg-day) | N | Mean | Std. Dev. |
| --- | --- | --- | --- |
| 0 | 7 | 194.4 | 44.8 |
| 0.2 | 7 | 112.2 | 30.8 |
| 1 | 7 | 106 | 26.5 |
| 2 | 7 | 109.9 | 26.8 |

Test 1 Dose Response: <0.0001

Test 2 Homogeneity of Variance: 0.4235

Test 3 Variance Model Selection: 0.9442

## Settings

| Setting | Value |
| --- | --- |
| BMR | 1.0 Standard Deviation |
| Distribution | Normal + Nonconstant variance |
| Adverse Direction | Down (↓) |
| Maximum Polynomial Degree | 3 |
| Confidence Level (one sided) | 0.95 |

## Maximum Likelihood Approach

| Model | BMDL | BMD | BMDU | *P*-Value | AIC | Scaled Residual at Control | Scaled Residual near BMD | Recommendation and Notes |
| --- | --- | --- | --- | --- | --- | --- | --- | --- |
| Exponential 3 | 1.085 | 2.625 | 4.292 | <0.001 | 291.828 | 2.356 | 0.63 | **Questionable** Residual at control > 2.0 Goodness of fit p-value < 0.1 BMD/highest dose ratio > 1.0 |
| Exponential 5 | 0.008 | 0.062 | 0.201 | - | 280.473 | 0.006 | 0.006 | **Questionable** lowest dose/BMDL ratio > 3.0 lowest dose/BMDL ratio > 10.0 lowest dose/BMD ratio > 3.0 Zero degrees of freedom; saturated model BMD/BMDL ratio > 3.0 |
| Hill | <0.001 | 0.172 | 0.187 | 0.774 | 278.475 | -0.009 | -0.073 | **Questionable** lowest dose/BMDL ratio > 3.0 lowest dose/BMDL ratio > 10.0 BMD/BMDL ratio > 3.0 BMD/BMDL ratio > 20.0 |
| Polynomial 2 | 1.363 | 2.595 | 2.648 | <0.001 | 292.459 | 2.446 | 0.563 | **Questionable** Residual at control > 2.0 Goodness of fit p-value < 0.1 BMD/highest dose ratio > 1.0 |
| Polynomial 3 | 1.365 | 2.653 | 2.708 | <0.001 | 292.447 | 2.442 | 0.535 | **Questionable** Residual at control > 2.0 Goodness of fit p-value < 0.1 BMD/highest dose ratio > 1.0 |
| Power | 1.366 | 2.71 | 3.476 | <0.001 | 292.441 | 2.47 | 0.514 | **Questionable** Residual at control > 2.0 Goodness of fit p-value < 0.1 BMD/highest dose ratio > 1.0 |
| Linear | 1.366 | 2.74 | 4.602 | <0.001 | 292.439 | 2.474 | 0.502 | **Questionable** Residual at control > 2.0 Goodness of fit p-value < 0.1 BMD/highest dose ratio > 1.0 |


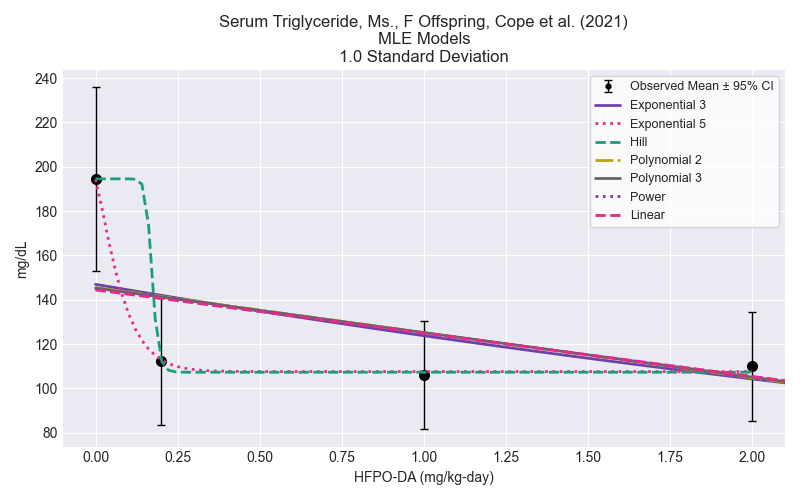


## Selected Model

No model was selected as a best-fitting model.

## Serum Triglyceride, Ms., F Offspring, Cope et al. (2021)

## Dataset

**Name:** Serum Triglyceride, Ms., F Offspring, Cope et al. (2021)

| Dose (mg/kg-day) | N | Mean | Std. Dev. |
| --- | --- | --- | --- |
| 0 | 7 | 194.4 | 44.8 |
| 0.2 | 7 | 112.2 | 30.8 |
| 1 | 7 | 106 | 26.5 |
| 2 | 7 | 109.9 | 26.8 |

Test 1 Dose Response: 0.0009

Test 2 Homogeneity of Variance: 0.9754

Test 3 Variance Model Selection: 0.9754

## Settings

| Setting | Value |
| --- | --- |
| BMR | 1.0 Standard Deviation |
| Distribution | Lognormal + Constant variance |
| Adverse Direction | Down (↓) |
| Maximum Polynomial Degree | 3 |
| Confidence Level (one sided) | 0.95 |

## Maximum Likelihood Approach

| Model | BMDL | BMD | BMDU | *P*-Value | AIC | Scaled Residual at Control | Scaled Residual near BMD | Recommendation and Notes |
| --- | --- | --- | --- | --- | --- | --- | --- | --- |
| Exponential 3 | 0.957 | 2.308 | 4.783 | <0.001 | 288.232 | 99.561 | 23.608 | **Questionable** \|Residual near BMD\| > 2.0 Residual at control > 2.0 Goodness of fit p-value < 0.1 BMD/highest dose ratio > 1.0 |
| Exponential 5 | 0.009 | 0.083 | 0.085 | - | 276.032 | 9.91 | 9.91 | **Questionable** \|Residual near BMD\| > 2.0 lowest dose/BMDL ratio > 3.0 lowest dose/BMDL ratio > 10.0 Zero degrees of freedom; saturated model Residual at control > 2.0 BMD/BMDL ratio > 3.0 |


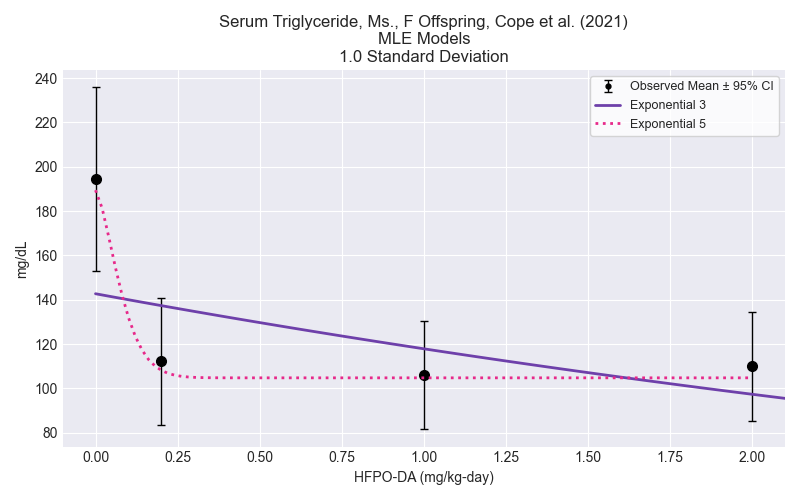


## Selected Model

No model was selected as a best-fitting model.

# Placenta weight, Rt, F, Lv et al. (2024)

## Dataset

**Name:** Placenta weight, Rt., F, Lv et al. (2024)

| Dose (mg/kg-day) | N | Mean | Std. Dev. |
| --- | --- | --- | --- |
| 0 | 6 | 0.35 | 0.07 |
| 1 | 6 | 0.34 | 0.03 |
| 10 | 6 | 0.39 | 0.04 |
| 100 | 6 | 0.46 | 0.04 |

## Settings

| Setting | Value |
| --- | --- |
| BMR | 5% Relative Deviation |
| Distribution | Normal + Constant variance |
| Adverse Direction | Up (↑) |
| Maximum Polynomial Degree | 3 |
| Confidence Level (one sided) | 0.95 |

## Maximum Likelihood Approach

| Model | BMDL | BMD | BMDU | *P*-Value | AIC | Scaled Residual at Control | Scaled Residual near BMD | Recommendation and Notes |
| --- | --- | --- | --- | --- | --- | --- | --- | --- |
| Exponential 3 | - | 6.419 | - | - | - | -2.043 | -139.476 | **Unusable** Did not successfully execute. |
| Exponential 5 | 1.302 | 8.722 | 19.436 | - | -72.431 | 0.282 | <0.001 | **Questionable** Zero degrees of freedom; saturated model BMD/BMDL ratio > 3.0 |
| Hill | 6.841 | 8.234 | 9.927 | - | -72.431 | 0.282 | <0.001 | **Questionable** Zero degrees of freedom; saturated model |
| Polynomial 2 | 11.877 | 16.612 | 49.837 | 0.265 | -73.935 | -0.285 | 1.284 | **Viable** |
| Polynomial 3 | 11.876 | 16.635 | 62.652 | 0.265 | -73.935 | -0.287 | 1.282 | **Viable** |
| Power | 11.877 | 16.62 | 91.005 | 0.265 | -73.935 | -0.286 | 1.283 | **Viable** |
| Linear^ab^ | 11.877 | 16.62 | 26.489 | 0.265 | -73.935 | -0.286 | 1.283 | **Recommended - Lowest AIC** |

^a^ BMDS recommended best fitting model

^b^ User selected best fitting model

## Selected Model: Linear


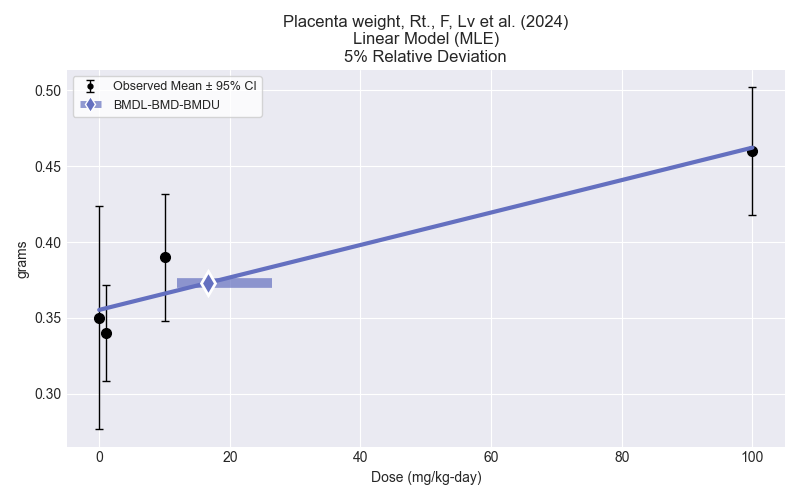


Linear Model
══════════════════════════════

Version: pybmds 25.1 (bmdscore 25.1)

Input Summary:
╒══════════════════════════════╤════════════════════════════╕
│ BMR │ 5% Relative Deviation │
│ Distribution │ Normal + Constant variance │
│ Modeling Direction │ Up (↑) │
│ Confidence Level (one sided) │ 0.95 │
│ Modeling Approach │ MLE │
│ Degree │ 1 │
╘══════════════════════════════╧════════════════════════════╛

Parameter Settings:
╒═════════════╤═══════════╤═════════╤════════╕
│ Parameter │ Initial │ Min │ Max │
╞═════════════╪═══════════╪═════════╪════════╡
│ g │ 0 │ -1e+06 │ 1e+06 │
│ b1 │ 0 │ -1e+06 │ 1e+06 │
│ alpha │ 0 │ -18 │ 18 │
╘═════════════╧═══════════╧═════════╧════════╛

Modeling Summary:
╒════════════════╤════════════╕
│ BMD │ 16.6204 │
│ BMDL │ 11.877 │
│ BMDU │ 26.4893 │
│ AIC │ -73.9354 │
│ Log-Likelihood │ 39.9677 │
│ P-Value │ 0.265128 │
│ Model d.f. │ 2 │
╘════════════════╧════════════╛

Model Parameters:
╒════════════╤════════════╤════════════╤═════════════╕
│ Variable │ Estimate │ On Bound │ Std Error │
╞════════════╪════════════╪════════════╪═════════════╡
│ g │ 0.355336 │ no │ 0.0112049 │
│ b1 │ 0.00106897 │ no │ 0.000222974 │
│ alpha │ 0.00209434 │ no │ 1.2662e-06 │
╘════════════╧════════════╧════════════╧═════════════╛

Goodness of Fit:
╒════════╤═════╤═══════════════╤═════════════════════╤═══════════════════╕
│ Dose │ N │ Sample Mean │ Model Fitted Mean │ Scaled Residual │
╞════════╪═════╪═══════════════╪═════════════════════╪═══════════════════╡
│ 0 │ 6 │ 0.35 │ 0.355336 │ -0.285605 │
│ 1 │ 6 │ 0.34 │ 0.356405 │ -0.878066 │
│ 10 │ 6 │ 0.39 │ 0.366026 │ 1.28321 │
│ 100 │ 6 │ 0.46 │ 0.462233 │ -0.11954 │
╘════════╧═════╧═══════════════╧═════════════════════╧═══════════════════╛
╒════════╤═════╤═════════════╤═══════════════════╕
│ Dose │ N │ Sample SD │ Model Fitted SD │
╞════════╪═════╪═════════════╪═══════════════════╡
│ 0 │ 6 │ 0.07 │ 0.0457639 │
│ 1 │ 6 │ 0.03 │ 0.0457639 │
│ 10 │ 6 │ 0.04 │ 0.0457639 │
│ 100 │ 6 │ 0.04 │ 0.0457639 │
╘════════╧═════╧═════════════╧═══════════════════╛

Likelihoods:
╒═════════╤══════════════════╤════════════╤══════════╕
│ Model │ Log-Likelihood │ # Params │ AIC │
╞═════════╪══════════════════╪════════════╪══════════╡
│ A1 │ 41.2952 │ 5 │ -72.5905 │
│ A2 │ 43.7548 │ 8 │ -71.5095 │
│ A3 │ 41.2952 │ 5 │ -72.5905 │
│ fitted │ 39.9677 │ 3 │ -73.9354 │
│ reduced │ 31.9067 │ 2 │ -59.8134 │
╘═════════╧══════════════════╧════════════╧══════════╛

Tests of Mean and Variance Fits:
╒════════╤══════════════════════════════╤═════════════╤═════════════╕
│ Name │ -2 * Log(Likelihood Ratio) │ Test d.f. │ P-Value │
╞════════╪══════════════════════════════╪═════════════╪═════════════╡
│ Test 1 │ 23.6961 │ 6 │ 0.000593912 │
│ Test 2 │ 4.91903 │ 3 │ 0.177823 │
│ Test 3 │ 4.91903 │ 3 │ 0.177823 │
│ Test 4 │ 2.65508 │ 2 │ 0.265128 │
╘════════╧══════════════════════════════╧═════════════╧═════════════╛
Test 1: Test the null hypothesis that responses and variances don't differ among dose levels
(A2 vs R). If this test fails to reject the null hypothesis (p-value > 0.05), there may not be
a dose-response.

Test 2: Test the null hypothesis that variances are homogenous (A1 vs A2). If this test fails to
reject the null hypothesis (p-value > 0.05), the simpler constant variance model may be appropriate.

Test 3: Test the null hypothesis that the variances are adequately modeled (A3 vs A2). If this test
fails to reject the null hypothesis (p-value > 0.05), it may be inferred that the variances have
been modeled appropriately.

Test 4: Test the null hypothesis that the model for the mean fits the data (Fitted vs A3). If this
test fails to reject the null hypothesis (p-value > 0.1), the user has support for use of the
selected model.

# Pup weight, Ms, M, PND4, DuPont (2010)

## Dataset

**Name:** Pup weight, Ms, M, PND4, DuPont (2010)

| Dose (mg/kg-day) | N | Mean | Std. Dev. |
| --- | --- | --- | --- |
| 0 | 22 | 3.17 | 0.39 |
| 0.1 | 21 | 3.11 | 0.43 |
| 0.5 | 24 | 3.3 | 0.421 |
| 5 | 21 | 2.57 | 0.359 |

Test 1 Dose Response: <0.0001

Test 2 Homogeneity of Variance: 0.8361

Test 3 Variance Model Selection: 0.8361

## Settings

| Setting | Value |
| --- | --- |
| BMR | 5% Relative Deviation |
| Distribution | Normal + Constant variance |
| Adverse Direction | Down (↓) |
| Maximum Polynomial Degree | 3 |
| Confidence Level (one sided) | 0.95 |

## Maximum Likelihood Approach

| Model | BMDL | BMD | BMDU | *P*-Value | AIC | Scaled Residual at Control | Scaled Residual near BMD | Recommendation and Notes |
| --- | --- | --- | --- | --- | --- | --- | --- | --- |
| Exponential 3 | 1.046 | 4.261 | 4.701 | 0.097 | 95.813 | -0.327 | <0.001 | **Questionable** Goodness of fit p-value < 0.1 BMD/BMDL ratio > 3.0 |
| Exponential 5 | 1.046 | 4.275 | 4.712 | - | 97.813 | -0.327 | -0. | **Questionable** Zero degrees of freedom; saturated model BMD/BMDL ratio > 3.0 |
| Hill | 4.525 | 4.602 | 4.686 | - | 97.813 | -0.327 | <0.001 | **Questionable** Zero degrees of freedom; saturated model |
| Polynomial 2 | 1.105 | 2.523 | 2.923 | 0.23 | 94.005 | -0.349 | 1.309 | **Viable** |
| Polynomial 3^ab^ | 1.119 | 3.17 | 3.495 | 0.251 | 93.832 | -0.329 | -0.002 | **Recommended - Lowest AIC** |
| Power | 1.121 | 4.632 | 4.739 | 0.097 | 95.813 | -0.327 | -0. | **Questionable** Goodness of fit p-value < 0.1 BMD/BMDL ratio > 3.0 |
| Linear | 1.008 | 1.267 | 1.719 | 0.093 | 95.824 | -0.582 | 1.745 | **Questionable** Goodness of fit p-value < 0.1 |

^a^ BMDS recommended best fitting model

^b^ User selected best fitting model


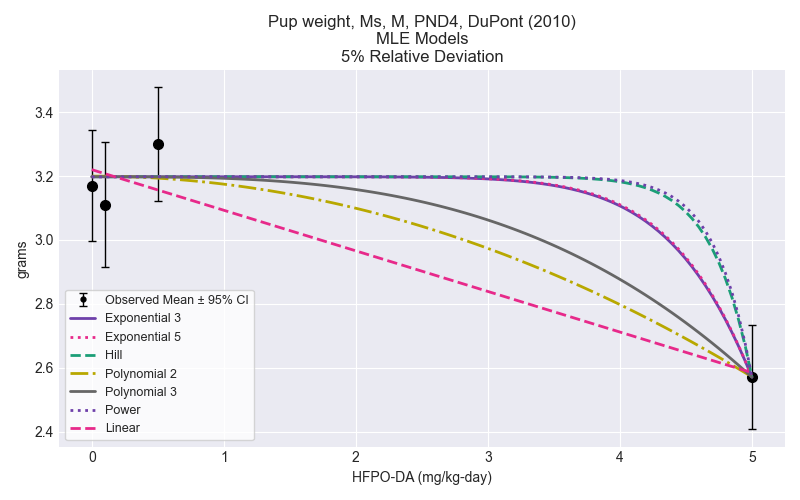


## Selected Model: Polynomial 3


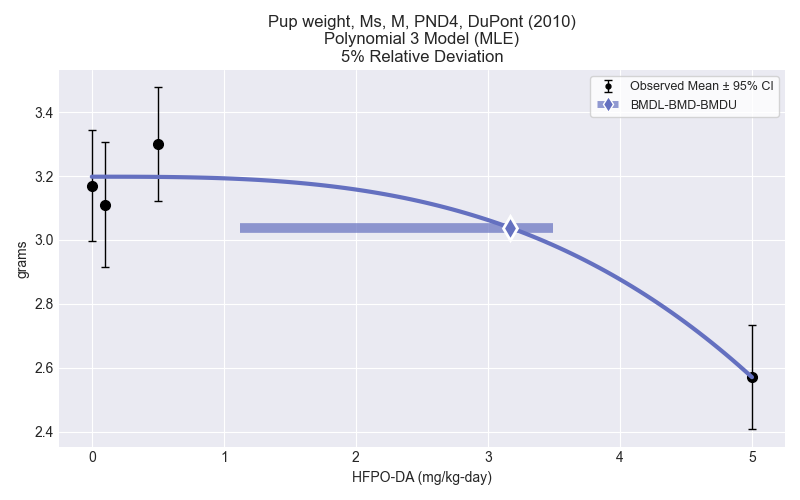


Polynomial 3 Model
══════════════════════════════

Version: pybmds 25.1 (bmdscore 25.1)

Input Summary:
╒══════════════════════════════╤════════════════════════════╕
│ BMR │ 5% Relative Deviation │
│ Distribution │ Normal + Constant variance │
│ Modeling Direction │ Down (↓) │
│ Confidence Level (one sided) │ 0.95 │
│ Modeling Approach │ MLE │
│ Degree │ 3 │
╘══════════════════════════════╧════════════════════════════╛

Parameter Settings:
╒═════════════╤═══════════╤═════════╤════════╕
│ Parameter │ Initial │ Min │ Max │
╞═════════════╪═══════════╪═════════╪════════╡
│ g │ 0 │ -1e+06 │ 1e+06 │
│ b1 │ 0 │ -1e+06 │ 0 │
│ b2 │ 0 │ -1e+06 │ 0 │
│ b3 │ 0 │ -1e+06 │ 0 │
│ alpha │ 0 │ -18 │ 18 │
╘═════════════╧═══════════╧═════════╧════════╛

Modeling Summary:
╒════════════════╤════════════╕
│ BMD │ 3.16953 │
│ BMDL │ 1.11924 │
│ BMDU │ 3.49497 │
│ AIC │ 93.8318 │
│ Log-Likelihood │ -43.9159 │
│ P-Value │ 0.250756 │
│ Model d.f. │ 2 │
╘════════════════╧════════════╛

Model Parameters:
╒════════════╤═════════════╤════════════╤══════════════╕
│ Variable │ Estimate │ On Bound │ Std Error │
╞════════════╪═════════════╪════════════╪══════════════╡
│ g │ 3.19794 │ no │ 0.0487114 │
│ b1 │ 0 │ yes │ Not Reported │
│ b2 │ 0 │ yes │ Not Reported │
│ b3 │ -0.00502178 │ no │ 0.000797727 │
│ alpha │ 0.158863 │ no │ 0.00380482 │
╘════════════╧═════════════╧════════════╧══════════════╛
Standard errors estimates are not generated for parameters estimated on corresponding bounds,
although sampling error is present for all parameters, as a rule. Standard error estimates may not
be reliable as a basis for confidence intervals or tests when one or more parameters are on bounds.


Goodness of Fit:
╒════════╤═════╤═══════════════╤═════════════════════╤═══════════════════╕
│ Dose │ N │ Sample Mean │ Model Fitted Mean │ Scaled Residual │
╞════════╪═════╪═══════════════╪═════════════════════╪═══════════════════╡
│ 0 │ 22 │ 3.17 │ 3.19794 │ -0.328773 │
│ 0.1 │ 21 │ 3.11 │ 3.19793 │ -1.011 │
│ 0.5 │ 24 │ 3.3 │ 3.19731 │ 1.26218 │
│ 5 │ 21 │ 2.57 │ 2.57022 │ -0.00248353 │
╘════════╧═════╧═══════════════╧═════════════════════╧═══════════════════╛
╒════════╤═════╤═════════════╤═══════════════════╕
│ Dose │ N │ Sample SD │ Model Fitted SD │
╞════════╪═════╪═════════════╪═══════════════════╡
│ 0 │ 22 │ 0.39 │ 0.398576 │
│ 0.1 │ 21 │ 0.43 │ 0.398576 │
│ 0.5 │ 24 │ 0.421 │ 0.398576 │
│ 5 │ 21 │ 0.359 │ 0.398576 │
╘════════╧═════╧═════════════╧═══════════════════╛

Likelihoods:
╒═════════╤══════════════════╤════════════╤══════════╕
│ Model │ Log-Likelihood │ # Params │ AIC │
╞═════════╪══════════════════╪════════════╪══════════╡
│ A1 │ -42.5326 │ 5 │ 95.0653 │
│ A2 │ -42.1048 │ 8 │ 100.21 │
│ A3 │ -42.5326 │ 5 │ 95.0653 │
│ fitted │ -43.9159 │ 3 │ 93.8318 │
│ reduced │ -60.2805 │ 2 │ 124.561 │
╘═════════╧══════════════════╧════════════╧══════════╛

Tests of Mean and Variance Fits:
╒════════╤══════════════════════════════╤═════════════╤═════════════╕
│ Name │ -2 * Log(Likelihood Ratio) │ Test d.f. │ P-Value │
╞════════╪══════════════════════════════╪═════════════╪═════════════╡
│ Test 1 │ 36.3515 │ 6 │ 2.35514e-06 │
│ Test 2 │ 0.855739 │ 3 │ 0.836094 │
│ Test 3 │ 0.855739 │ 3 │ 0.836094 │
│ Test 4 │ 2.76655 │ 2 │ 0.250756 │
╘════════╧══════════════════════════════╧═════════════╧═════════════╛
Test 1: Test the null hypothesis that responses and variances don't differ among dose levels
(A2 vs R). If this test fails to reject the null hypothesis (p-value > 0.05), there may not be
a dose-response.

Test 2: Test the null hypothesis that variances are homogenous (A1 vs A2). If this test fails to
reject the null hypothesis (p-value > 0.05), the simpler constant variance model may be appropriate.

Test 3: Test the null hypothesis that the variances are adequately modeled (A3 vs A2). If this test
fails to reject the null hypothesis (p-value > 0.05), it may be inferred that the variances have
been modeled appropriately.

Test 4: Test the null hypothesis that the model for the mean fits the data (Fitted vs A3). If this
test fails to reject the null hypothesis (p-value > 0.1), the user has support for use of the
selected model.

# Pup birthweight GD8-PND2, Rt, F, Conley et al. (2021)

## Dataset

**Name:** Pup birthweight, Rt, F, Conley et al. (2021)

| Dose (mg/kg-day) | N | Mean | Std. Dev. |
| --- | --- | --- | --- |
| 0 | 5 | 6.38 | 0.4 |
| 10 | 5 | 6.33 | 0.2 |
| 30 | 5 | 5.81 | 0.27 |
| 62.5 | 5 | 5.63 | 0.13 |
| 125 | 5 | 5.41 | 0.25 |
| 250 | 5 | 4.71 | 0.42 |

Test 1 Dose Response: <0.0001

Test 2 Homogeneity of Variance: 0.1347

Test 3 Variance Model Selection: 0.1347

## Settings

| Setting | Value |
| --- | --- |
| BMR | 5% Relative Deviation |
| Distribution | Normal + Constant variance |
| Adverse Direction | Down (↓) |
| Maximum Polynomial Degree | 3 |
| Confidence Level (one sided) | 0.95 |

## Maximum Likelihood Approach

| Model | BMDL | BMD | BMDU | *P*-Value | AIC | Scaled Residual at Control | Scaled Residual near BMD | Recommendation and Notes |
| --- | --- | --- | --- | --- | --- | --- | --- | --- |
| Exponential 3 | 36.725 | 43.427 | 58.365 | 0.103 | 19.283 | 1.017 | -1.595 | **Viable** |
| Exponential 5 | 17.349 | 27.471 | 45.99 | 0.16 | 18.739 | 0.285 | -1.464 | **Viable** |
| Hill^ab^ | 13.512 | 24.223 | 43.581 | 0.197 | 18.252 | 0.098 | -1.351 | **Recommended - Lowest AIC** |
| Polynomial 2 | 42.493 | 49.271 | 63.757 | 0.062 | 20.535 | 1.207 | -1.374 | **Questionable** Goodness of fit p-value < 0.1 |
| Polynomial 3 | 42.497 | 49.315 | 63.758 | 0.062 | 20.535 | 1.213 | -1.375 | **Questionable** Goodness of fit p-value < 0.1 |
| Power | 42.491 | 49.245 | 64.434 | 0.062 | 20.535 | 1.206 | -1.374 | **Questionable** Goodness of fit p-value < 0.1 |
| Linear | 42.491 | 49.245 | 58.787 | 0.062 | 20.535 | 1.206 | -1.374 | **Questionable** Goodness of fit p-value < 0.1 |

^a^ BMDS recommended best fitting model

^b^ User selected best fitting model


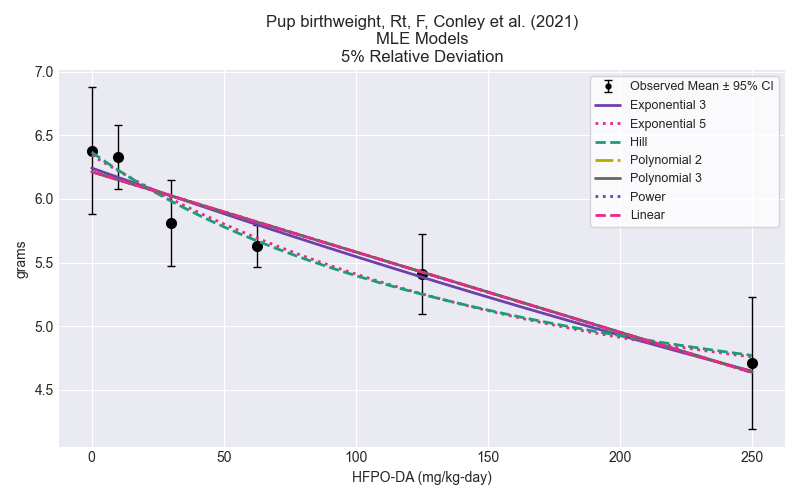


## Selected Model: Hill


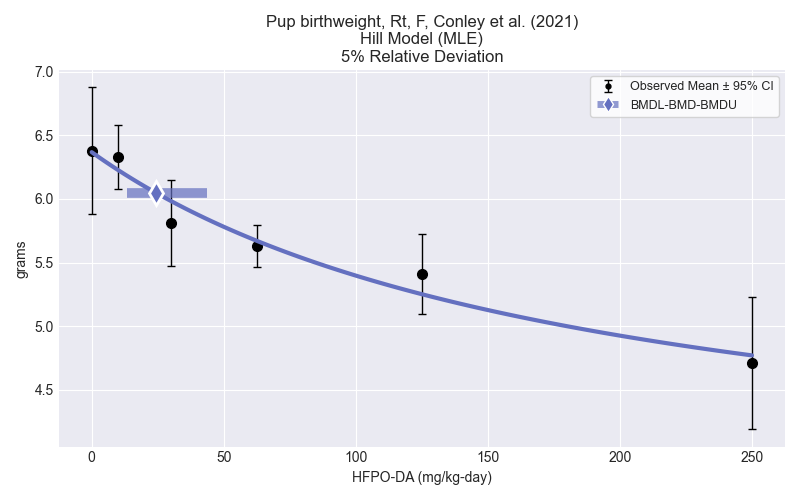


Hill Model
══════════════════════════════

Version: pybmds 25.1 (bmdscore 25.1)

Input Summary:
╒══════════════════════════════╤════════════════════════════╕
│ BMR │ 5% Relative Deviation │
│ Distribution │ Normal + Constant variance │
│ Modeling Direction │ Down (↓) │
│ Confidence Level (one sided) │ 0.95 │
│ Modeling Approach │ MLE │
╘══════════════════════════════╧════════════════════════════╛

Parameter Settings:
╒═════════════╤═══════════╤═══════╤═══════╕
│ Parameter │ Initial │ Min │ Max │
╞═════════════╪═══════════╪═══════╪═══════╡
│ g │ 0 │ -100 │ 100 │
│ v │ 0 │ -100 │ 100 │
│ k │ 0 │ 0 │ 5 │
│ n │ 1 │ 1 │ 18 │
│ alpha │ 0 │ -18 │ 18 │
╘═════════════╧═══════════╧═══════╧═══════╛

Modeling Summary:
╒════════════════╤═══════════╕
│ BMD │ 24.2225 │
│ BMDL │ 13.5124 │
│ BMDU │ 43.5813 │
│ AIC │ 18.2518 │
│ Log-Likelihood │ -5.12591 │
│ P-Value │ 0.196567 │
│ Model d.f. │ 3 │
╘════════════════╧═══════════╛

Model Parameters:
╒════════════╤═════════════╤════════════╤══════════════╕
│ Variable │ Estimate │ On Bound │ Std Error │
╞════════════╪═════════════╪════════════╪══════════════╡
│ g │ 6.36736 │ no │ 0.107416 │
│ v │ -2.80554 │ no │ 0.8777 │
│ k │ 189.233 │ no │ 128.763 │
│ n │ 1 │ yes │ Not Reported │
│ alpha │ 0.0824017 │ no │ 0.00175318 │
╘════════════╧═════════════╧════════════╧══════════════╛
Standard errors estimates are not generated for parameters estimated on corresponding bounds,
although sampling error is present for all parameters, as a rule. Standard error estimates may not
be reliable as a basis for confidence intervals or tests when one or more parameters are on bounds.


Goodness of Fit:
╒════════╤═════╤═══════════════╤═════════════════════╤═══════════════════╕
│ Dose │ N │ Sample Mean │ Model Fitted Mean │ Scaled Residual │
╞════════╪═════╪═══════════════╪═════════════════════╪═══════════════════╡
│ 0 │ 5 │ 6.38 │ 6.36736 │ 0.0984658 │
│ 10 │ 5 │ 6.33 │ 6.22654 │ 0.805899 │
│ 30 │ 5 │ 5.81 │ 5.98345 │ -1.35109 │
│ 62.5 │ 5 │ 5.63 │ 5.6708 │ -0.317834 │
│ 125 │ 5 │ 5.41 │ 5.25133 │ 1.23597 │
│ 250 │ 5 │ 4.71 │ 4.77052 │ -0.471413 │
╘════════╧═════╧═══════════════╧═════════════════════╧═══════════════════╛
╒════════╤═════╤═════════════╤═══════════════════╕
│ Dose │ N │ Sample SD │ Model Fitted SD │
╞════════╪═════╪═════════════╪═══════════════════╡
│ 0 │ 5 │ 0.4 │ 0.287057 │
│ 10 │ 5 │ 0.2 │ 0.287057 │
│ 30 │ 5 │ 0.27 │ 0.287057 │
│ 62.5 │ 5 │ 0.13 │ 0.287057 │
│ 125 │ 5 │ 0.25 │ 0.287057 │
│ 250 │ 5 │ 0.42 │ 0.287057 │
╘════════╧═════╧═════════════╧═══════════════════╛

Likelihoods:
╒═════════╤══════════════════╤════════════╤═════════╕
│ Model │ Log-Likelihood │ # Params │ AIC │
╞═════════╪══════════════════╪════════════╪═════════╡
│ A1 │ -2.7846 │ 7 │ 19.5692 │
│ A2 │ 1.42439 │ 12 │ 21.1512 │
│ A3 │ -2.7846 │ 7 │ 19.5692 │
│ fitted │ -5.12591 │ 4 │ 18.2518 │
│ reduced │ -28.5885 │ 2 │ 61.177 │
╘═════════╧══════════════════╧════════════╧═════════╛

Tests of Mean and Variance Fits:
╒════════╤══════════════════════════════╤═════════════╤═════════════╕
│ Name │ -2 * Log(Likelihood Ratio) │ Test d.f. │ P-Value │
╞════════╪══════════════════════════════╪═════════════╪═════════════╡
│ Test 1 │ 60.0257 │ 10 │ 3.58389e-09 │
│ Test 2 │ 8.41797 │ 5 │ 0.134655 │
│ Test 3 │ 8.41797 │ 5 │ 0.134655 │
│ Test 4 │ 4.68263 │ 3 │ 0.196567 │
╘════════╧══════════════════════════════╧═════════════╧═════════════╛
Test 1: Test the null hypothesis that responses and variances don't differ among dose levels
(A2 vs R). If this test fails to reject the null hypothesis (p-value > 0.05), there may not be
a dose-response.

Test 2: Test the null hypothesis that variances are homogenous (A1 vs A2). If this test fails to
reject the null hypothesis (p-value > 0.05), the simpler constant variance model may be appropriate.

Test 3: Test the null hypothesis that the variances are adequately modeled (A3 vs A2). If this test
fails to reject the null hypothesis (p-value > 0.05), it may be inferred that the variances have
been modeled appropriately.

Test 4: Test the null hypothesis that the model for the mean fits the data (Fitted vs A3). If this
test fails to reject the null hypothesis (p-value > 0.1), the user has support for use of the
selected model.

# Serum triglycerides, Rt, F, Conley et al. (2021)

## Dataset

**Name:** Serum triglycerides, Rt, F, Conley(2021)

| Dose (mg/kg-day) | N | Mean | Std. Dev. |
| --- | --- | --- | --- |
| 0 | 6 | 390 | 157 |
| 1 | 4 | 268 | 102 |
| 3 | 4 | 256 | 74 |
| 10 | 4 | 197 | 114 |
| 30 | 4 | 181 | 40 |
| 62.5 | 4 | 196 | 54 |
| 125 | 4 | 165 | 30 |

Test 1 Dose Response: 0.0004

Test 2 Homogeneity of Variance: 0.0086

Test 3 Variance Model Selection: 0.5513

## Settings

| Setting | Value |
| --- | --- |
| BMR | 1.0 Standard Deviation |
| Distribution | Normal + Nonconstant variance |
| Adverse Direction | Down (↓) |
| Maximum Polynomial Degree | 3 |
| Confidence Level (one sided) | 0.95 |

## Maximum Likelihood Approach

| Model | BMDL | BMD | BMDU | *P*-Value | AIC | Scaled Residual at Control | Scaled Residual near BMD | Recommendation and Notes |
| --- | --- | --- | --- | --- | --- | --- | --- | --- |
| Exponential 3 | 66.408 | 97.649 | 99.67 | 0.011 | 362.645 | 2.126 | 0.943 | **Questionable** Residual at control > 2.0 Goodness of fit p-value < 0.1 |
| Exponential 5 | 0.946 | 20.567 | - | 0.244 | 355.208 | 1.083 | 0.07 | **Questionable** BMD/BMDL ratio > 3.0 BMD/BMDL ratio > 20.0 |
| Hill^ab^ | 0.662 | 5.872 | - | 0.421 | 353.646 | 0.407 | 0.125 | **Recommended - Lowest AIC** BMD/BMDL ratio > 3.0 |
| Polynomial 2 | 92.676 | 126.716 | 129.339 | 0.014 | 362.08 | 2.302 | 0.633 | **Questionable** Residual at control > 2.0 Goodness of fit p-value < 0.1 BMD/highest dose ratio > 1.0 |
| Polynomial 3 | 110.052 | 127.544 | 186.466 | 0.014 | 362.027 | 2.307 | 0.621 | **Questionable** Residual at control > 2.0 Goodness of fit p-value < 0.1 BMD/highest dose ratio > 1.0 |
| Power | 92.981 | 127.409 | 160.963 | 0.014 | 362.03 | 2.303 | 0.619 | **Questionable** Residual at control > 2.0 Goodness of fit p-value < 0.1 BMD/highest dose ratio > 1.0 |
| Linear | 92.996 | 127.544 | 186.466 | 0.014 | 362.027 | 2.307 | 0.621 | **Questionable** Residual at control > 2.0 Goodness of fit p-value < 0.1 BMD/highest dose ratio > 1.0 |

^a^ BMDS recommended best fitting model

^b^ User selected best fitting model


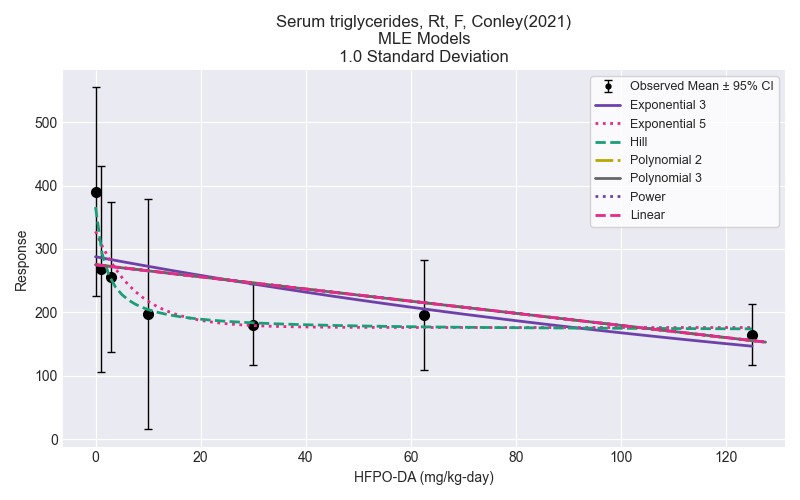


## Selected Model: Hill


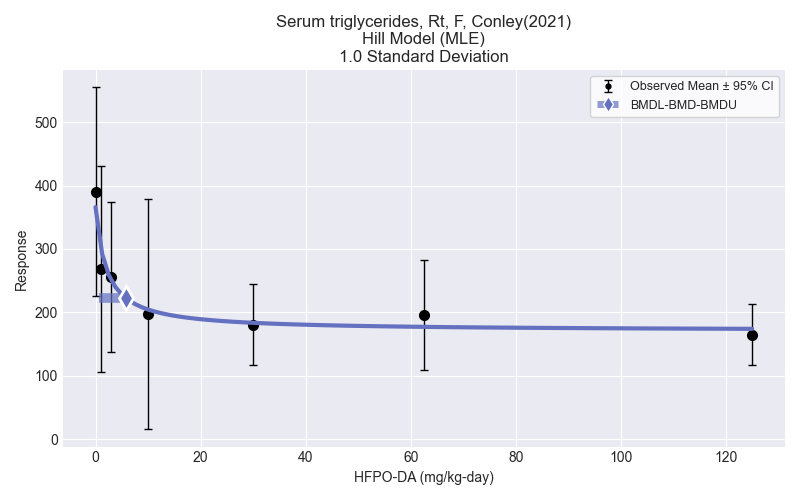


Hill Model
══════════════════════════════

Version: pybmds 25.1 (bmdscore 25.1)

Input Summary:
╒══════════════════════════════╤═══════════════════════════════╕
│ BMR │ 1.0 Standard Deviation │
│ Distribution │ Normal + Nonconstant variance │
│ Modeling Direction │ Down (↓) │
│ Confidence Level (one sided) │ 0.95 │
│ Modeling Approach │ MLE │
╘══════════════════════════════╧═══════════════════════════════╛

Parameter Settings:
╒═════════════╤═══════════╤═══════╤═══════╕
│ Parameter │ Initial │ Min │ Max │
╞═════════════╪═══════════╪═══════╪═══════╡
│ g │ 0 │ -100 │ 100 │
│ v │ 0 │ -100 │ 100 │
│ k │ 0 │ 0 │ 5 │
│ n │ 1 │ 1 │ 18 │
│ rho │ 0 │ -18 │ 18 │
│ alpha │ 0 │ -18 │ 18 │
╘═════════════╧═══════════╧═══════╧═══════╛

Modeling Summary:
╒════════════════╤══════════════╕
│ BMD │ 5.87209 │
│ BMDL │ 0.661727 │
│ BMDU │ -9999 │
│ AIC │ 353.646 │
│ Log-Likelihood │ -171.823 │
│ P-Value │ 0.420919 │
│ Model d.f. │ 4 │
╘════════════════╧══════════════╛

Model Parameters:
╒════════════╤════════════════╤════════════╤══════════════╕
│ Variable │ Estimate │ On Bound │ Std Error │
╞════════════╪════════════════╪════════════╪══════════════╡
│ g │ 366.089 │ no │ 63.4142 │
│ v │ -195.069 │ no │ 60.9608 │
│ k │ 2.08007 │ no │ 2.43521 │
│ n │ 1 │ yes │ Not Reported │
│ rho │ 3.00111 │ no │ 1.41508 │
│ alpha │ 0.000420135 │ no │ 1.36095e-06 │
╘════════════╧════════════════╧════════════╧══════════════╛
Standard errors estimates are not generated for parameters estimated on corresponding bounds,
although sampling error is present for all parameters, as a rule. Standard error estimates may not
be reliable as a basis for confidence intervals or tests when one or more parameters are on bounds.


Goodness of Fit:
╒════════╤═════╤═══════════════╤═════════════════════╤═══════════════════╕
│ Dose │ N │ Sample Mean │ Model Fitted Mean │ Scaled Residual │
╞════════╪═════╪═══════════════╪═════════════════════╪═══════════════════╡
│ 0 │ 6 │ 390 │ 366.089 │ 0.406613 │
│ 1 │ 4 │ 268 │ 302.756 │ -0.641727 │
│ 3 │ 4 │ 256 │ 250.892 │ 0.125033 │
│ 10 │ 4 │ 197 │ 204.609 │ -0.252913 │
│ 30 │ 4 │ 181 │ 183.668 │ -0.104277 │
│ 62.5 │ 4 │ 196 │ 177.303 │ 0.770544 │
│ 125 │ 4 │ 165 │ 174.212 │ -0.389807 │
╘════════╧═════╧═══════════════╧═════════════════════╧═══════════════════╛
╒════════╤═════╤═════════════╤═══════════════════╕
│ Dose │ N │ Sample SD │ Model Fitted SD │
╞════════╪═════╪═════════════╪═══════════════════╡
│ 0 │ 6 │ 157 │ 144.044 │
│ 1 │ 4 │ 102 │ 108.32 │
│ 3 │ 4 │ 74 │ 81.7064 │
│ 10 │ 4 │ 114 │ 60.1675 │
│ 30 │ 4 │ 40 │ 51.1682 │
│ 62.5 │ 4 │ 54 │ 48.5305 │
│ 125 │ 4 │ 30 │ 47.2669 │
╘════════╧═════╧═════════════╧═══════════════════╛

Likelihoods:
╒═════════╤══════════════════╤════════════╤═════════╕
│ Model │ Log-Likelihood │ # Params │ AIC │
╞═════════╪══════════════════╪════════════╪═════════╡
│ A1 │ -176.483 │ 8 │ 368.965 │
│ A2 │ -167.884 │ 14 │ 363.767 │
│ A3 │ -169.877 │ 9 │ 357.754 │
│ fitted │ -171.823 │ 5 │ 353.646 │
│ reduced │ -185.613 │ 2 │ 375.226 │
╘═════════╧══════════════════╧════════════╧═════════╛

Tests of Mean and Variance Fits:
╒════════╤══════════════════════════════╤═════════════╤═════════════╕
│ Name │ -2 * Log(Likelihood Ratio) │ Test d.f. │ P-Value │
╞════════╪══════════════════════════════╪═════════════╪═════════════╡
│ Test 1 │ 35.4586 │ 12 │ 0.000395685 │
│ Test 2 │ 17.1978 │ 6 │ 0.00858315 │
│ Test 3 │ 3.98696 │ 5 │ 0.551296 │
│ Test 4 │ 3.89128 │ 4 │ 0.420919 │
╘════════╧══════════════════════════════╧═════════════╧═════════════╛
Test 1: Test the null hypothesis that responses and variances don't differ among dose levels
(A2 vs R). If this test fails to reject the null hypothesis (p-value > 0.05), there may not be
a dose-response.

Test 2: Test the null hypothesis that variances are homogenous (A1 vs A2). If this test fails to
reject the null hypothesis (p-value > 0.05), the simpler constant variance model may be appropriate.

Test 3: Test the null hypothesis that the variances are adequately modeled (A3 vs A2). If this test
fails to reject the null hypothesis (p-value > 0.05), it may be inferred that the variances have
been modeled appropriately.

Test 4: Test the null hypothesis that the model for the mean fits the data (Fitted vs A3). If this
test fails to reject the null hypothesis (p-value > 0.1), the user has support for use of the
selected model.

# Serum triglycerides, dams, E11.5, Ms, F, Blake et al. (2020)

## Dataset

**Name:** Serum triglycerides, dams, E11.5, Ms, F, Blake et al. (2020)

| Dose (mg/kg-day) | N | Mean | Std. Dev. |
| --- | --- | --- | --- |
| 0 | 5 | 205.6 | 56 |
| 2 | 5 | 117.6 | 33.9 |
| 10 | 4 | 80.3 | 14.4 |

Test 1 Dose Response: 0.0001

Test 2 Homogeneity of Variance: 0.0369

Test 3 Variance Model Selection: 0.0369

## Settings

| Setting | Value |
| --- | --- |
| BMR | 1.0 Standard Deviation |
| Distribution | Normal + Constant variance |
| Adverse Direction | Down (↓) |
| Maximum Polynomial Degree | 2 |
| Confidence Level (one sided) | 0.95 |

## Maximum Likelihood Approach

| Model | BMDL | BMD | BMDU | *P*-Value | AIC | Scaled Residual at Control | Scaled Residual near BMD | Recommendation and Notes |
| --- | --- | --- | --- | --- | --- | --- | --- | --- |
| Exponential 3 | 1.144 | 2.392 | 6.611 | 0.021 | 151.131 | 1.036 | -1.616 | **Questionable** Goodness of fit p-value < 0.1 Constant variance test failed (Test 2 p-value < 0.05) |
| Exponential 5 | 0.037 | 1.276 | 1.979 | - | 149.772 | <0.001 | <0.001 | **Questionable** lowest dose/BMDL ratio > 3.0 lowest dose/BMDL ratio > 10.0 Zero degrees of freedom; saturated model Constant variance test failed (Test 2 p-value < 0.05) BMD/BMDL ratio > 3.0 BMD/BMDL ratio > 20.0 |
| Hill | <0.001 | 0.588 | 0.853 | - | 149.772 | <0.001 | <0.001 | **Questionable** lowest dose/BMDL ratio > 3.0 lowest dose/BMDL ratio > 10.0 lowest dose/BMD ratio > 3.0 Zero degrees of freedom; saturated model Constant variance test failed (Test 2 p-value < 0.05) BMD/BMDL ratio > 3.0 BMD/BMDL ratio > 20.0 |
| Polynomial 2 | 2.8 | 4.364 | 9.795 | 0.008 | 152.866 | 1.441 | -1.816 | **Questionable** Goodness of fit p-value < 0.1 Constant variance test failed (Test 2 p-value < 0.05) |
| Power | 2.8 | 4.378 | 9.795 | 0.008 | 152.866 | 1.452 | -1.815 | **Questionable** Goodness of fit p-value < 0.1 Constant variance test failed (Test 2 p-value < 0.05) |
| Linear | 2.8 | 4.378 | 9.795 | 0.008 | 152.866 | 1.452 | -1.815 | **Questionable** Goodness of fit p-value < 0.1 Constant variance test failed (Test 2 p-value < 0.05) |


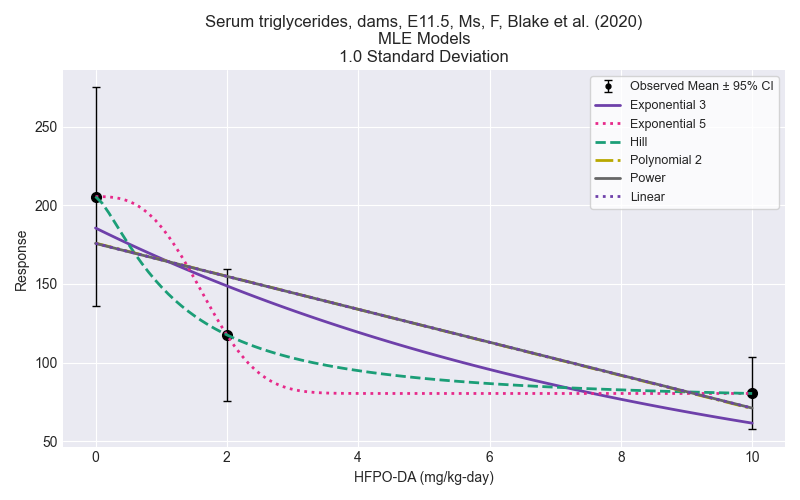


## Selected Model

No model was selected as a best-fitting model.

## Serum triglycerides, dams, E11.5, Ms, F, Blake et al. (2020)

## Dataset

**Name:** Serum triglycerides, dams, E11.5, Ms, F, Blake et al. (2020)

| Dose (mg/kg-day) | N | Mean | Std. Dev. |
| --- | --- | --- | --- |
| 0 | 5 | 205.6 | 56 |
| 2 | 5 | 117.6 | 33.9 |
| 10 | 4 | 80.3 | 14.4 |

Test 1 Dose Response: 0.0001

Test 2 Homogeneity of Variance: 0.0369

Test 3 Variance Model Selection: 0.4517

## Settings

| Setting | Value |
| --- | --- |
| BMR | 1.0 Standard Deviation |
| Distribution | Normal + Nonconstant variance |
| Adverse Direction | Down (↓) |
| Maximum Polynomial Degree | 2 |
| Confidence Level (one sided) | 0.95 |

## Maximum Likelihood Approach

| Model | BMDL | BMD | BMDU | *P*-Value | AIC | Scaled Residual at Control | Scaled Residual near BMD | Recommendation and Notes |
| --- | --- | --- | --- | --- | --- | --- | --- | --- |
| Exponential 3 | 2.855 | 5.682 | 12.431 | 0.031 | 146.417 | 1.357 | -1.442 | **Questionable** Goodness of fit p-value < 0.1 |
| Exponential 5 | 0.447 | 1.102 | 10.386 | - | 145.74 | 0.138 | -0.247 | **Questionable** lowest dose/BMDL ratio > 3.0 Zero degrees of freedom; saturated model |
| Hill | 0.262 | 1.861 | 10.523 | - | 145.74 | 0.138 | -0.247 | **Questionable** lowest dose/BMDL ratio > 3.0 Zero degrees of freedom; saturated model BMD/BMDL ratio > 3.0 |
| Polynomial 2 | 4.237 | 6.904 | 7.046 | 0.018 | 147.324 | 1.481 | 0.097 | **Questionable** Goodness of fit p-value < 0.1 |
| Power | 4.233 | 6.789 | 6.929 | 0.018 | 147.334 | 1.467 | 0.119 | **Questionable** Goodness of fit p-value < 0.1 |
| Linear | 4.239 | 6.999 | 12.679 | 0.018 | 147.322 | 1.49 | 0.079 | **Questionable** Goodness of fit p-value < 0.1 |


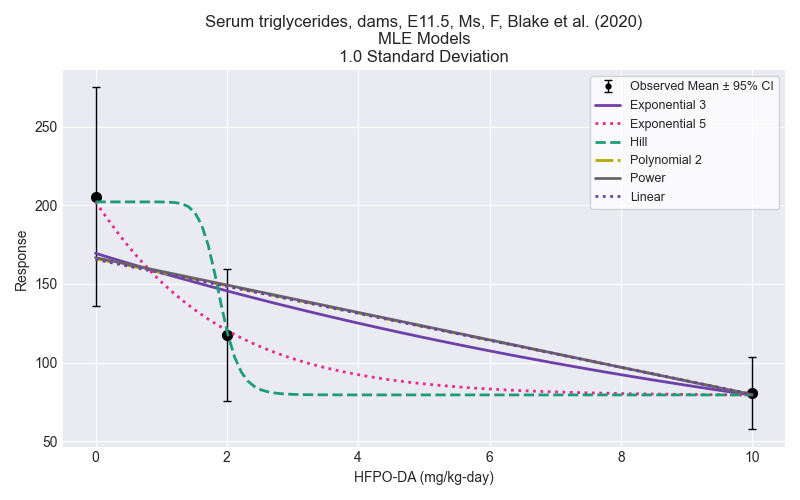


## Selected Model

No model was selected as a best-fitting model.

## Serum triglycerides, dams, E11.5, Ms, F, Blake et al. (2020)

## Dataset

**Name:** Serum triglycerides, dams, E11.5, Ms, F, Blake et al. (2020)

| Dose (mg/kg-day) | N | Mean | Std. Dev. |
| --- | --- | --- | --- |
| 0 | 5 | 205.6 | 56 |
| 2 | 5 | 117.6 | 33.9 |
| 10 | 4 | 80.3 | 14.4 |

Test 1 Dose Response: 0.0006

Test 2 Homogeneity of Variance: 0.58

Test 3 Variance Model Selection: 0.58

## Settings

| Setting | Value |
| --- | --- |
| BMR | 1.0 Standard Deviation |
| Distribution | Lognormal + Constant variance |
| Adverse Direction | Down (↓) |
| Maximum Polynomial Degree | 0 |
| Confidence Level (one sided) | 0.95 |

## Maximum Likelihood Approach

| Model | BMDL | BMD | BMDU | *P*-Value | AIC | Scaled Residual at Control | Scaled Residual near BMD | Recommendation and Notes |
| --- | --- | --- | --- | --- | --- | --- | --- | --- |
| Exponential 3 | 2.38 | 4.982 | 6.855 | 0.01 | 145.566 | 66.102 | -40.825 | **Questionable** \|Residual near BMD\| > 2.0 Residual at control > 2.0 Goodness of fit p-value < 0.1 |
| Exponential 5 | 0.359 | 1.225 | 1.304 | - | 142.935 | 12.476 | 7.973 | **Questionable** \|Residual near BMD\| > 2.0 lowest dose/BMDL ratio > 3.0 Zero degrees of freedom; saturated model Residual at control > 2.0 BMD/BMDL ratio > 3.0 |


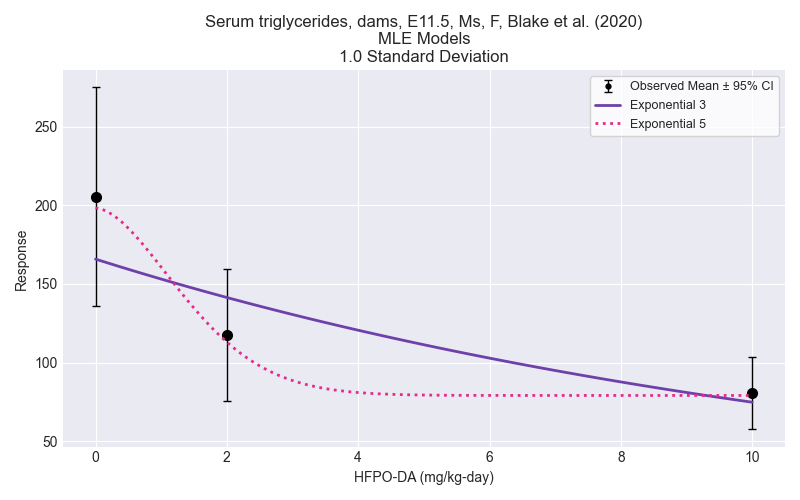


## Selected Model

No model was selected as a best-fitting model.

# Serum triglycerides, dams, E17.5, Ms, F, Blake et al. (2020)

## Dataset

**Name:** Serum triglycerides, dams, E17.5, Ms, F, Blake et al. (2020)

| Dose (mg/kg-day) | N | Mean | Std. Dev. |
| --- | --- | --- | --- |
| 0 | 4 | 472.5 | 78.9 |
| 2 | 5 | 257 | 120.3 |
| 10 | 5 | 120.6 | 31.7 |

Test 1 Dose Response: <0.0001

Test 2 Homogeneity of Variance: 0.0275

Test 3 Variance Model Selection: 0.0275

## Settings

| Setting | Value |
| --- | --- |
| BMR | 1.0 Standard Deviation |
| Distribution | Normal + Constant variance |
| Adverse Direction | Down (↓) |
| Maximum Polynomial Degree | 2 |
| Confidence Level (one sided) | 0.95 |

## Maximum Likelihood Approach

| Model | BMDL | BMD | BMDU | *P*-Value | AIC | Scaled Residual at Control | Scaled Residual near BMD | Recommendation and Notes |
| --- | --- | --- | --- | --- | --- | --- | --- | --- |
| Exponential 3 | 0.645 | 1.359 | 1.387 | 0.041 | 171.12 | 0.948 | -1.281 | **Questionable** lowest dose/BMDL ratio > 3.0 Goodness of fit p-value < 0.1 Constant variance test failed (Test 2 p-value < 0.05) |
| Exponential 5 | 0.283 | 0.662 | 1.954 | - | 170.942 | <0.001 | <0.001 | **Questionable** lowest dose/BMDL ratio > 3.0 lowest dose/BMD ratio > 3.0 Zero degrees of freedom; saturated model Constant variance test failed (Test 2 p-value < 0.05) |
| Hill | 0.158 | 0.78 | 1.008 | - | 170.942 | <0.001 | <0.001 | **Questionable** lowest dose/BMDL ratio > 3.0 lowest dose/BMDL ratio > 10.0 Zero degrees of freedom; saturated model Constant variance test failed (Test 2 p-value < 0.05) BMD/BMDL ratio > 3.0 |
| Polynomial 2 | 2.331 | 3.437 | 6.705 | 0.006 | 174.454 | 1.59 | -1.777 | **Questionable** Goodness of fit p-value < 0.1 Constant variance test failed (Test 2 p-value < 0.05) |
| Power | 2.33 | 3.435 | 6.439 | 0.006 | 174.454 | 1.59 | -1.778 | **Questionable** Goodness of fit p-value < 0.1 Constant variance test failed (Test 2 p-value < 0.05) |
| Linear | 2.33 | 3.435 | 6.358 | 0.006 | 174.454 | 1.59 | -1.778 | **Questionable** Goodness of fit p-value < 0.1 Constant variance test failed (Test 2 p-value < 0.05) |


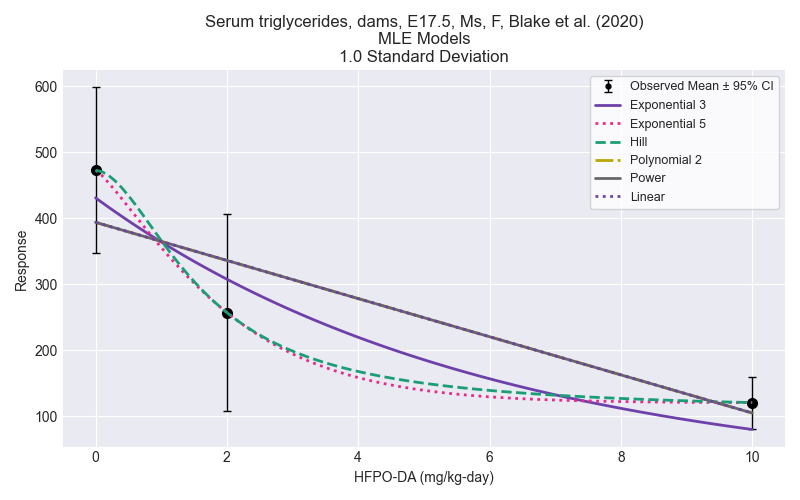


## Selected Model

No model was selected as a best-fitting model.

## Serum triglycerides, dams, E17.5, Ms, F, Blake et al. (2020)

## Dataset

**Name:** Serum triglycerides, dams, E17.5, Ms, F, Blake et al. (2020)

| Dose (mg/kg-day) | N | Mean | Std. Dev. |
| --- | --- | --- | --- |
| 0 | 4 | 472.5 | 78.9 |
| 2 | 5 | 257 | 120.3 |
| 10 | 5 | 120.6 | 31.7 |

Test 1 Dose Response: <0.0001

Test 2 Homogeneity of Variance: 0.0275

Test 3 Variance Model Selection: 0.0432

## Settings

| Setting | Value |
| --- | --- |
| BMR | 1.0 Standard Deviation |
| Distribution | Normal + Nonconstant variance |
| Adverse Direction | Down (↓) |
| Maximum Polynomial Degree | 2 |
| Confidence Level (one sided) | 0.95 |

## Maximum Likelihood Approach

| Model | BMDL | BMD | BMDU | *P*-Value | AIC | Scaled Residual at Control | Scaled Residual near BMD | Recommendation and Notes |
| --- | --- | --- | --- | --- | --- | --- | --- | --- |
| Exponential 3 | 1.708 | 3.4 | 3.47 | 0.259 | 167.118 | 1.016 | -1.298 | **Questionable** Nonconstant variance test failed (Test 3 p-value < 0.05) Control stdev. fit > 1.5 |
| Exponential 5 | 0.446 | 1.475 | 7.562 | - | 169.845 | 0.395 | -0.552 | **Questionable** lowest dose/BMDL ratio > 3.0 Zero degrees of freedom; saturated model Nonconstant variance test failed (Test 3 p-value < 0.05) Control stdev. fit > 1.5 BMD/BMDL ratio > 3.0 |
| Hill | 0.332 | 1.349 | 7.917 | - | 169.845 | 0.395 | -0.552 | **Questionable** lowest dose/BMDL ratio > 3.0 Zero degrees of freedom; saturated model Nonconstant variance test failed (Test 3 p-value < 0.05) Control stdev. fit > 1.5 BMD/BMDL ratio > 3.0 |
| Polynomial 2 | 4.153 | 5.459 | 7.31 | 0.129 | 168.144 | 1.413 | -1.306 | **Questionable** Nonconstant variance test failed (Test 3 p-value < 0.05) Control stdev. fit > 1.5 |
| Power | 3.388 | 5.548 | 11.439 | 0.129 | 168.146 | 1.419 | -1.275 | **Questionable** Nonconstant variance test failed (Test 3 p-value < 0.05) Control stdev. fit > 1.5 |
| Linear | 3.39 | 5.459 | 11.434 | 0.129 | 168.144 | 1.413 | -1.306 | **Questionable** Nonconstant variance test failed (Test 3 p-value < 0.05) Control stdev. fit > 1.5 |


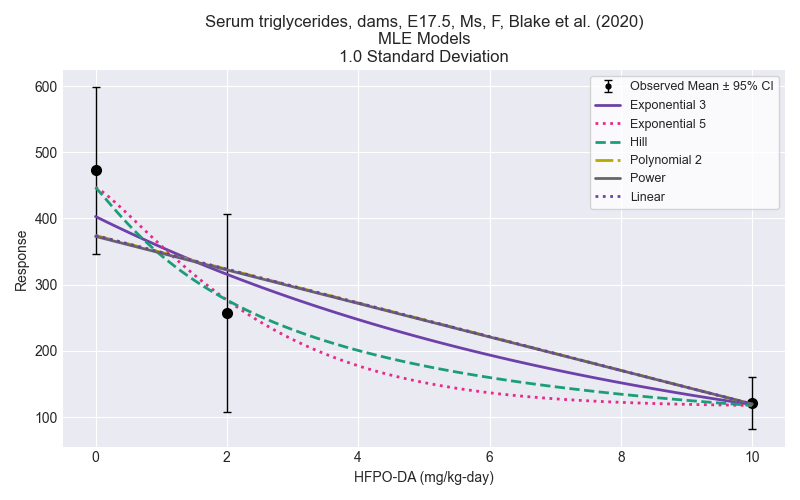


## Selected Model

No model was selected as a best-fitting model.

## Serum triglycerides, dams, E17.5, Ms, F, Blake et al. (2020)

## Dataset

**Name:** Serum triglycerides, dams, E17.5, Ms, F, Blake et al. (2020)

| Dose (mg/kg-day) | N | Mean | Std. Dev. |
| --- | --- | --- | --- |
| 0 | 4 | 472.5 | 78.9 |
| 2 | 5 | 257 | 120.3 |
| 10 | 5 | 120.6 | 31.7 |

Test 1 Dose Response: <0.0001

Test 2 Homogeneity of Variance: 0.1168

Test 3 Variance Model Selection: 0.1168

## Settings

| Setting | Value |
| --- | --- |
| BMR | 1.0 Standard Deviation |
| Distribution | Lognormal + Constant variance |
| Adverse Direction | Down (↓) |
| Maximum Polynomial Degree | 0 |
| Confidence Level (one sided) | 0.95 |

## Maximum Likelihood Approach

| Model | BMDL | BMD | BMDU | *P*-Value | AIC | Scaled Residual at Control | Scaled Residual near BMD | Recommendation and Notes |
| --- | --- | --- | --- | --- | --- | --- | --- | --- |
| Exponential 3 | 1.972 | 4.306 | 5.293 | 0.028 | 166.742 | 140.301 | -58.835 | **Questionable** \|Residual near BMD\| > 2.0 Residual at control > 2.0 Goodness of fit p-value < 0.1 |
| Exponential 5 | 0.436 | 1.307 | 1.339 | - | 165.89 | 7.364 | 39.428 | **Questionable** \|Residual near BMD\| > 2.0 lowest dose/BMDL ratio > 3.0 Zero degrees of freedom; saturated model Residual at control > 2.0 |


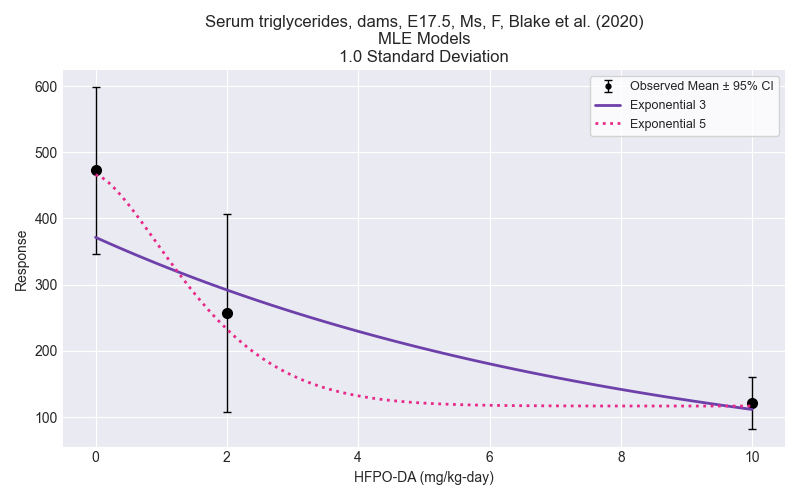


## Selected Model

No model was selected as a best-fitting model.

# Fetal length (ND), Rt, F, GD0.5-19.5, Lv et al. (2024)

## Dataset

**Name:** Fetal length (ND), Rt, F, GD0.5-19.5, Lv et al. (2024)

| Dose | N | Mean | Std. Dev. |
| --- | --- | --- | --- |
| 0 | 18 | 53.77 | 1.8 |
| 1 | 18 | 53.29 | 2.28 |
| 10 | 18 | 49.88 | 2.74 |
| 100 | 18 | 47.5 | 2.09 |

Test 1 Dose Response: <0.0001

Test 2 Homogeneity of Variance: 0.3412

Test 3 Variance Model Selection: 0.2267

## Settings

| Setting | Value |
| --- | --- |
| BMR | 5% Relative Deviation |
| Distribution | Normal + Nonconstant variance |
| Adverse Direction | Down (↓) |
| Maximum Polynomial Degree | 3 |
| Confidence Level (one sided) | 0.95 |

## Maximum Likelihood Approach

| Model | BMDL | BMD | BMDU | *P*-Value | AIC | Scaled Residual at Control | Scaled Residual near BMD | Recommendation and Notes |
| --- | --- | --- | --- | --- | --- | --- | --- | --- |
| Exponential 3 | 39.129 | 47.936 | 48.928 | <0.001 | 346.326 | 1.891 | -3.668 | **Questionable** \|Residual near BMD\| > 2.0 Goodness of fit p-value < 0.1 |
| Exponential 5^ab^ | 3.621 | 6.035 | 10.017 | 0.539 | 327.213 | <0.001 | -0. | **Recommended - Lowest AIC** |
| Hill | 23.326 | 23.819 | 30.168 | <0.001 | 339.8 | 1.705 | -2.528 | **Questionable** \|Residual near BMD\| > 2.0 Goodness of fit p-value < 0.1 |
| Polynomial 2 | 41.265 | 49.843 | 50.874 | <0.001 | 346.268 | 1.961 | -3.576 | **Questionable** \|Residual near BMD\| > 2.0 Goodness of fit p-value < 0.1 |
| Polynomial 3 | 41.011 | 49.748 | 50.777 | <0.001 | 346.472 | 1.929 | -3.61 | **Questionable** \|Residual near BMD\| > 2.0 Goodness of fit p-value < 0.1 |
| Power | 42.159 | 50.775 | 64.33 | <0.001 | 345.596 | 1.927 | -3.447 | **Questionable** \|Residual near BMD\| > 2.0 Goodness of fit p-value < 0.1 Control stdev. fit > 1.5 |
| Linear | 42.159 | 50.781 | 63.276 | <0.001 | 345.596 | 1.926 | -3.445 | **Questionable** \|Residual near BMD\| > 2.0 Goodness of fit p-value < 0.1 Control stdev. fit > 1.5 |

^a^ BMDS recommended best fitting model

^b^ User selected best fitting model


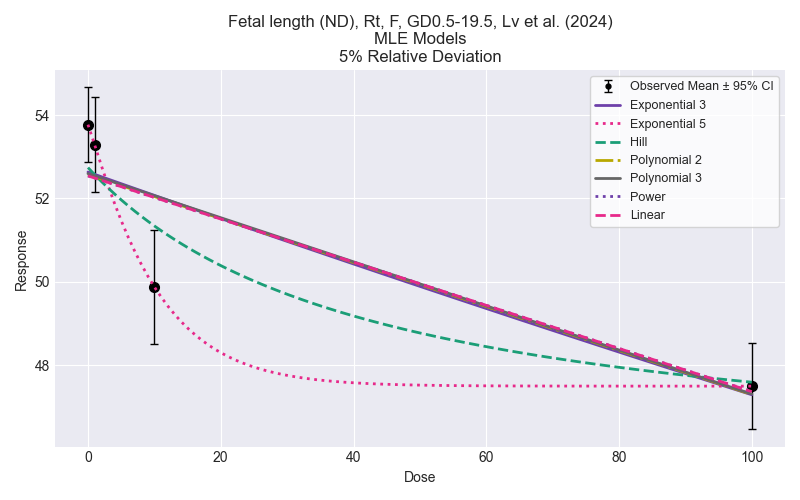
Selected Model: Exponential 5


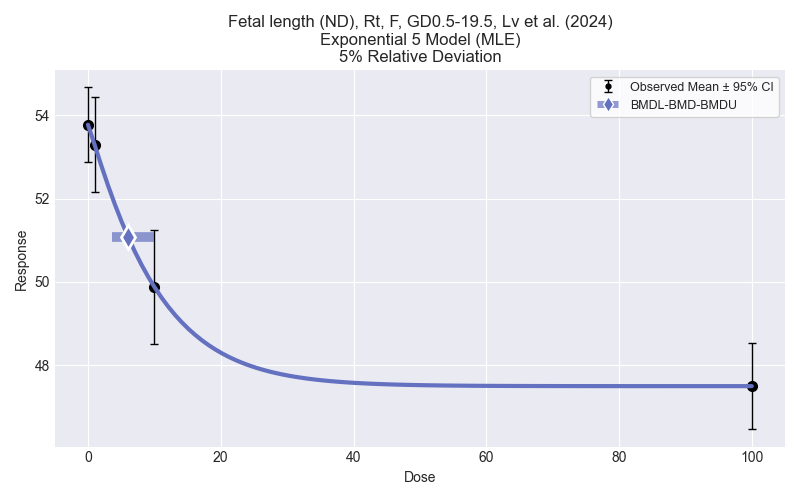


Exponential 5 Model
══════════════════════════════

Version: pybmds 25.1 (bmdscore 25.1)

Input Summary:
╒══════════════════════════════╤═══════════════════════════════╕
│ BMR │ 5% Relative Deviation │
│ Distribution │ Normal + Nonconstant variance │
│ Modeling Direction │ Down (↓) │
│ Confidence Level (one sided) │ 0.95 │
│ Modeling Approach │ MLE │
╘══════════════════════════════╧═══════════════════════════════╛

Parameter Settings:
╒═════════════╤═══════════╤═══════╤═══════╕
│ Parameter │ Initial │ Min │ Max │
╞═════════════╪═══════════╪═══════╪═══════╡
│ a │ 0 │ 0 │ 100 │
│ b │ 0 │ 0 │ 100 │
│ c │ 0 │ -20 │ 0 │
│ d │ 1 │ 1 │ 18 │
│ rho │ 0 │ 0 │ 18 │
│ log-alpha │ 0 │ -18 │ 18 │
╘═════════════╧═══════════╧═══════╧═══════╛

Modeling Summary:
╒════════════════╤═════════════╕
│ BMD │ 6.0347 │
│ BMDL │ 3.62063 │
│ BMDU │ 10.0174 │
│ AIC │ 327.213 │
│ Log-Likelihood │ -158.607 │
│ P-Value │ 0.538779 │
│ Model d.f. │ 1 │
╘════════════════╧═════════════╛

Model Parameters:
╒════════════╤════════════╤════════════╤══════════════╕
│ Variable │ Estimate │ On Bound │ Std Error │
╞════════════╪════════════╪════════════╪══════════════╡
│ a │ 53.77 │ no │ 0.516109 │
│ b │ 0.0971083 │ no │ 0.0247446 │
│ c │ 0.883391 │ no │ 0.0145075 │
│ d │ 1.08502 │ no │ 0.644827 │
│ rho │ 0 │ yes │ Not Reported │
│ log-alpha │ 1.56786 │ no │ 0.166663 │
╘════════════╧════════════╧════════════╧══════════════╛
Standard errors estimates are not generated for parameters estimated on corresponding bounds,
although sampling error is present for all parameters, as a rule. Standard error estimates may not
be reliable as a basis for confidence intervals or tests when one or more parameters are on bounds.


Goodness of Fit:
╒════════╤═════╤═══════════════╤═════════════════════╤═══════════════════╕
│ Dose │ N │ Sample Mean │ Model Fitted Mean │ Scaled Residual │
╞════════╪═════╪═══════════════╪═════════════════════╪═══════════════════╡
│ 0 │ 18 │ 53.77 │ 53.77 │ 2.129e-07 │
│ 1 │ 18 │ 53.29 │ 53.29 │ -5.9578e-08 │
│ 10 │ 18 │ 49.88 │ 49.88 │ -1.22988e-07 │
│ 100 │ 18 │ 47.5 │ 47.5 │ 9.65577e-08 │
╘════════╧═════╧═══════════════╧═════════════════════╧═══════════════════╛
╒════════╤═════╤═════════════╤═══════════════════╕
│ Dose │ N │ Sample SD │ Model Fitted SD │
╞════════╪═════╪═════════════╪═══════════════════╡
│ 0 │ 18 │ 1.8 │ 2.19007 │
│ 1 │ 18 │ 2.28 │ 2.19007 │
│ 10 │ 18 │ 2.74 │ 2.19007 │
│ 100 │ 18 │ 2.09 │ 2.19007 │
╘════════╧═════╧═════════════╧═══════════════════╛

Likelihoods:
╒═════════╤══════════════════╤════════════╤═════════╕
│ Model │ Log-Likelihood │ # Params │ AIC │
╞═════════╪══════════════════╪════════════╪═════════╡
│ A1 │ -158.607 │ 5 │ 327.213 │
│ A2 │ -156.933 │ 8 │ 329.867 │
│ A3 │ -158.418 │ 6 │ 328.835 │
│ fitted │ -158.607 │ 5 │ 327.213 │
│ reduced │ -189.741 │ 2 │ 383.482 │
╘═════════╧══════════════════╧════════════╧═════════╛

Tests of Mean and Variance Fits:
╒════════╤══════════════════════════════╤═════════════╤════════════╕
│ Name │ -2 * Log(Likelihood Ratio) │ Test d.f. │ P-Value │
╞════════╪══════════════════════════════╪═════════════╪════════════╡
│ Test 1 │ 65.615 │ 6 │ 3.2303e-12 │
│ Test 2 │ 3.34647 │ 3 │ 0.341226 │
│ Test 3 │ 2.96867 │ 2 │ 0.226653 │
│ Test 4 │ 0.377807 │ 1 │ 0.538779 │
╘════════╧══════════════════════════════╧═════════════╧════════════╛
Test 1: Test the null hypothesis that responses and variances don't differ among dose levels
(A2 vs R). If this test fails to reject the null hypothesis (p-value > 0.05), there may not be
a dose-response.

Test 2: Test the null hypothesis that variances are homogenous (A1 vs A2). If this test fails to
reject the null hypothesis (p-value > 0.05), the simpler constant variance model may be appropriate.

Test 3: Test the null hypothesis that the variances are adequately modeled (A3 vs A2). If this test
fails to reject the null hypothesis (p-value > 0.05), it may be inferred that the variances have
been modeled appropriately.

Test 4: Test the null hypothesis that the model for the mean fits the data (Fitted vs A3). If this
test fails to reject the null hypothesis (p-value > 0.1), the user has support for use of the
selected model.

# Fetal tail length (ND), Rt, F, GD0.5-19.5, Lv et al. (2024)

## Dataset

**Name:** Fetal tail length (ND), Rt, F, GD0.5-19.5, Lv et al. (2024)

| Dose | N | Mean | Std. Dev. |
| --- | --- | --- | --- |
| 0 | 18 | 16.38 | 1.28 |
| 1 | 18 | 16.58 | 2.34 |
| 10 | 18 | 15.5 | 0.7 |
| 100 | 18 | 14.38 | 0.97 |

Test 1 Dose Response: <0.0001

Test 2 Homogeneity of Variance: <0.0001

Test 3 Variance Model Selection: <0.0001

## Settings

| Setting | Value |
| --- | --- |
| BMR | 5% Relative Deviation |
| Distribution | Normal + Constant variance |
| Adverse Direction | Down (↓) |
| Maximum Polynomial Degree | 3 |
| Confidence Level (one sided) | 0.95 |

## Maximum Likelihood Approach

| Model | BMDL | BMD | BMDU | *P*-Value | AIC | Scaled Residual at Control | Scaled Residual near BMD | Recommendation and Notes |
| --- | --- | --- | --- | --- | --- | --- | --- | --- |
| Exponential 3 | 8.88 | 9.803 | 10.275 | 0 | 499.158 | -0.058 | -0. | **Questionable** Goodness of fit p-value < 0.1 Control stdev. fit > 1.5 Constant variance test failed (Test 2 p-value < 0.05) |
| Exponential 5 | 3.489 | 9.758 | 28.359 | - | 265.041 | -0.298 | <0.001 | **Questionable** Zero degrees of freedom; saturated model Constant variance test failed (Test 2 p-value < 0.05) |
| Hill | 91.934 | 94.186 | 97.632 | - | 270.525 | 0.651 | <0.001 | **Questionable** Zero degrees of freedom; saturated model Constant variance test failed (Test 2 p-value < 0.05) |
| Polynomial 2 | 31.638 | 42.482 | 77.057 | 0.14 | 264.79 | 0.406 | -1.596 | **Questionable** Constant variance test failed (Test 2 p-value < 0.05) |
| Polynomial 3 | 31.639 | 42.565 | 83.649 | 0.14 | 264.79 | 0.409 | -1.594 | **Questionable** Constant variance test failed (Test 2 p-value < 0.05) |
| Power | 31.638 | 42.472 | 90.905 | 0.14 | 264.79 | 0.405 | -1.597 | **Questionable** Constant variance test failed (Test 2 p-value < 0.05) |
| Linear | 31.638 | 42.472 | 65.28 | 0.14 | 264.79 | 0.405 | -1.597 | **Questionable** Constant variance test failed (Test 2 p-value < 0.05) |


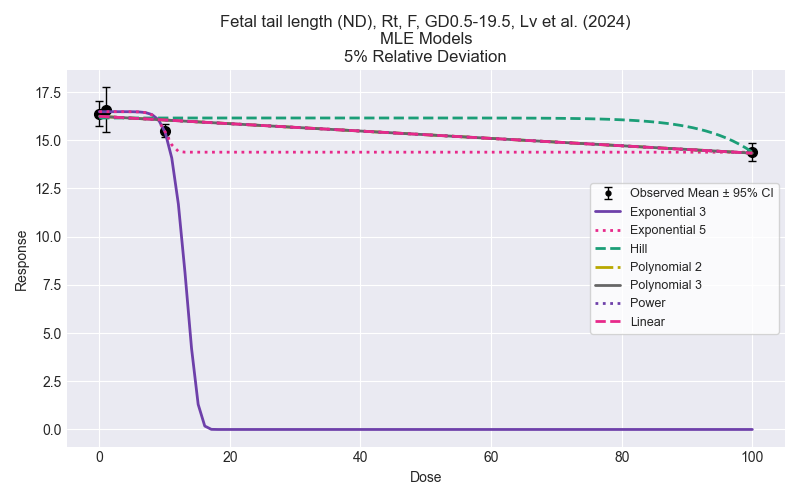


## Selected Model

No model was selected as a best-fitting model.

## Session for Fetal tail length (ND), Rt, F, GD0.5-19.5, Lv et al. (2024)

## Dataset

**Name:** Fetal tail length (ND), Rt, F, GD0.5-19.5, Lv et al. (2024)

| Dose | N | Mean | Std. Dev. |
| --- | --- | --- | --- |
| 0 | 18 | 16.38 | 1.28 |
| 1 | 18 | 16.58 | 2.34 |
| 10 | 18 | 15.5 | 0.7 |
| 100 | 18 | 14.38 | 0.97 |

## Settings

| Setting | Value |
| --- | --- |
| BMR | 5% Relative Deviation |
| Distribution | Normal + Nonconstant variance |
| Adverse Direction | Down (↓) |
| Maximum Polynomial Degree | 3 |
| Confidence Level (one sided) | 0.95 |

## Maximum Likelihood Approach

| Model | BMDL | BMD | BMDU | *P*-Value | AIC | Scaled Residual at Control | Scaled Residual near BMD | Recommendation and Notes |
| --- | --- | --- | --- | --- | --- | --- | --- | --- |
| Exponential 3 | - | 6.32 | - | - | - | 0 | 750.39 | **Unusable** Did not successfully execute. |
| Exponential 5 | 3.328 | 9.123 | 10.47 | 0.013 | 252.785 | -0.73 | 0.723 | **Questionable** Nonconstant variance test failed (Test 3 p-value < 0.05) Goodness of fit p-value < 0.1 |
| Hill | 7.783 | 8.865 | 9.067 | - | 254.785 | -0.73 | 0.723 | **Questionable** Zero degrees of freedom; saturated model Nonconstant variance test failed (Test 3 p-value < 0.05) |
| Polynomial 2 | 33.751 | 41.678 | 70.155 | <0.001 | 260.111 | 0.324 | -1.588 | **Questionable** Nonconstant variance test failed (Test 3 p-value < 0.05) Goodness of fit p-value < 0.1 |
| Polynomial 3 | - | 72.879 | - | - | 271.679 | 0.613 | 0.069 | **Unusable** Did not successfully execute. |
| Power | - | 41.904 | - | <0.001 | 266.054 | 0.383 | -1.609 | **Unusable** Did not successfully execute. |
| Linear | 33.751 | 41.678 | 56.166 | <0.001 | 260.111 | 0.324 | -1.588 | **Questionable** Nonconstant variance test failed (Test 3 p-value < 0.05) Goodness of fit p-value < 0.1 |

## Selected Model

No model was selected as a best-fitting model.

## Session for Fetal tail length (ND), Rt, F, GD0.5-19.5, Lv et al. (2024)

## Dataset

**Name:** Fetal tail length (ND), Rt, F, GD0.5-19.5, Lv et al. (2024)

| Dose | N | Mean | Std. Dev. |
| --- | --- | --- | --- |
| 0 | 18 | 16.38 | 1.28 |
| 1 | 18 | 16.58 | 2.34 |
| 10 | 18 | 15.5 | 0.7 |
| 100 | 18 | 14.38 | 0.97 |

Test 1 Dose Response: <0.0001

Test 2 Homogeneity of Variance: <0.0001

Test 3 Variance Model Selection: <0.0001

## Settings

| Setting | Value |
| --- | --- |
| BMR | 5% Relative Deviation |
| Distribution | Lognormal + Constant variance |
| Adverse Direction | Down (↓) |
| Maximum Polynomial Degree | 3 |
| Confidence Level (one sided) | 0.95 |

## Maximum Likelihood Approach

| Model | BMDL | BMD | BMDU | *P*-Value | AIC | Scaled Residual at Control | Scaled Residual near BMD | Recommendation and Notes |
| --- | --- | --- | --- | --- | --- | --- | --- | --- |
| Exponential 3 | 31.353 | 42.115 | 96.984 | 0.214 | 258.339 | 0.855 | -1.8 | **Questionable** Constant variance test failed (Test 2 p-value < 0.05) |
| Exponential 5 | 3.722 | 9.838 | 37.013 | - | 259.285 | 0.023 | 0.06 | **Questionable** Zero degrees of freedom; saturated model Constant variance test failed (Test 2 p-value < 0.05) |


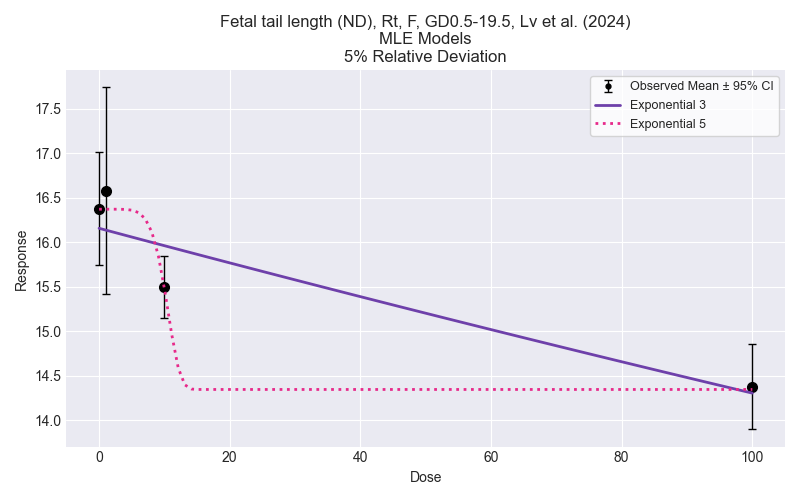


## Selected Model

No model was selected as a best-fitting model.
