# Supplementary material for: Integration of mechanistic and repeat dose toxicity data in the derivation of an oral reference dose for HFPO-DA
Source: Toxicol Sci. 2026 Apr 10;209(5):kfag045. doi: 10.1093/toxsci/kfag045 (PMC13176454; doi:10.1093/toxsci/kfag045)
Supplement: kfag045_Supplementary_Data [file kfag045_supplementary_data.zip › Supplementary Tables.docx]

**Supplementary Tables**

**Table S1. Literature search strategy and results**

| **Database** | **Syntax** | **Results from Search Date:**  **9-8-2023** | **Results from Search Date:**  **1-14-2025^b^** |
| --- | --- | --- | --- |
| PubMed | GenX OR "HFPO DA" OR "2,3,3,3‐tetrafluoro‐2‐(heptafluoropropoxy)‐propanoate" OR "2,3,3,3‐tetrafluoro‐2‐(heptafluoropropoxy) propionic acid" OR "Ammonium 2,3,3,3-tetrafluoro-2-(heptafluoropropoxy)propanoate" OR "Hexafluoropropylene Oxide Dimer Acid" OR heptafluoropropyl 1,2,2,2-tetrafluoroethyl ether OR ("Perfluoro(2-methyl-3-oxahexanoate)" OR "Perfluoro(2-methyl-3-oxahexanoic) acid" OR "2,3,3,3-tetrafluoro-2-(heptafluoropropoxy)propanoic acid" OR "perfluoro-2-propoxypropanoic acid" AND (acid OR acids)) OR (GenX AND (fluorocarbon* OR fluorotelomer* OR polyfluoro* OR perfluoro-* OR perfluoroa* OR perfluorob* OR perfluoroc* OR perfluorod* OR perfluoroe* OR perfluoroh* OR perfluoron* OR perfluoroo* OR perfluorop* OR perfluoros* OR perfluorou* OR perfluorinated OR fluorinated)) OR "Ammonium perfluoro-2-methyl-3-oxahexanoate" OR ((Undecafluoro AND oxahexanoic) OR (Ammonium AND perfluoro AND oxahexanoic) OR (Tetrafluoro AND heptafluoropropoxy) AND (salt OR salts OR acid OR acids)) AND (English[Filter]) | 307 | 166 |
| Embase | 'genx OR 'hfpo da' OR '2,3,3,3‐tetrafluoro‐2‐(heptafluoropropoxy)‐propanoate' OR 'hexafluoropropylene oxide dimer acid' OR 'heptafluoropropyl 1,2,2,2-tetrafluoroethyl ether' OR 'perfluoro(2-methyl-3-oxahexanoate)' OR 'perfluoro(2-methyl-3-oxahexanoic) acid' OR 'perfluoro-2-propoxypropanoic acid' OR 'ammonium perfluoro-2-methyl-3-oxahexanoate' OR ((undecafluoro AND oxahexanoic OR (ammonium AND perfluoro AND oxahexanoic) OR (tetrafluoro AND heptafluoropropoxy)) AND (salt OR salts OR acid OR acids)) AND [English]/lim | 336 | 178 |
| EPA HERO^a^ | GenX Chemicals (CASRN 13252-13-6 and CASRN 62037-80-3) project | 155 | Not performed |

^a^ HERO GenX database: <https://hero.epa.gov/hero/index.cfm/project/page/project_id/2627>

^b^ Date-limited from previous search date (9-8-2023)

| Table S2: Inclusion/Exclusion Criteria | | |
| --- | --- | --- |
| **PECO Element** | **Inclusion Criteria** | **Exclusion Criteria** |
| *Population (P)* | - *In vivo* experimental animal models (mammalian models only) - Epidemiological studies | - Non-mammalian models (*in vivo* and *in vitro*­)^a^ - *In vitro* models^a^ - *Ex vivo* models^a^ |
| *Exposures (E)* | - Controlled or measured exposure to HFPO-DA via oral route | - Exposure via routes of exposure other than oral - Doses (concentrations, exposure duration and frequency) of HFPO-DA are not clearly reported - Studies using only one dose/concentration - Exposure lasted less than 28 days - Co-exposure to other test materials in experimental animal studies^b^ |
| *Comparator (C)* | - Studies that include an untreated, sham, or vehicle-exposed negative control - A comparison or reference population exposed to lower levels (or no exposure/   exposure below detection limits) of HFPO-DA | - Studies that do not include an appropriate comparator - Studies that do not report results of the negative controls |
| *Outcome (O)* | - Non-cancer and cancer apical outcomes | - Mechanistic effects; experimental assessment of non-apical activity^a^ |
| *Study type/ Other Criteria* | - Published in English - Full text available - Primary (i.e., empirical) research | - Published in language other than English - Conference abstracts, letter to the editor, opinions - Mode of action assessments, systematic reviews, meta-analysis^a^ |
| ^a^ Studies of *in vitro, ex vivo* or non-mammalian models, mechanistic effects, or mode of action assessments, systematic review and meta-analysis were excluded from data extraction and synthesis. However, if identified during evidence identification, these study types were categorized for consideration during the mode of action analysis or targeted assessment of apical outcome biological plausibility and human relevance.  ^b^ Co-exposure in observational studies in humans were assessed for impact on dose response as part of critical appraisal. | | |

**Table S3. Dosimetric Adjustment Factor (DAF) Estimates for HFPO-DA^e^**

|  | CL, L/h^a^ | V_d_, L/kg | Half-life^b^, h | CL_h_/CL_a-sex_ | DAF, (1/ CL_h_/CL_a-sex_) |
| --- | --- | --- | --- | --- | --- |
| Rat, male | 0.0029 | 0.303 | 72.2 | 0.28792629 | 3.5^c^ |
| Rat, female | 0.0259 | 2.519 | 67.4 | 0.03233095 | 30.9 |
| Mouse, male | 0.0046 | 0.247 | 36.9 | 0.18051619 | 5.5^d^ |
| Mouse, female | 0.0065 | 0.226 | 24.2 | 0.12938791 | 7.7^d^ |
| Monkey, male | 0.0010 | 0.097 | 64.1 |  |  |
| Monkey, female | 0.0007 | 0.077 | 79.6 |  |  |
| Human | 0.0008 | 0.087^f^ | 72^g^ |  |  |

^a^ CL = V_d_ x (ln(2)/t_1/2_)

^b^ β phase half-life

^c^ similar to default rat allometric scaling factor

^d^ similar to default mouse allometric scaling factor

^e^ rat, mouse, and monkey data are from Gannon et al. (2016); human half-life based on limited human data (see main text); V_d_ based on the average of male and female monkeys

^f^ average based on male and female monkey values

^g^ see main text
