# Supplementary material for: Integration of mechanistic and repeat dose toxicity data in the derivation of an oral reference dose for HFPO-DA
Source: Toxicol Sci. 2026 Apr 10;209(5):kfag045. doi: 10.1093/toxsci/kfag045 (PMC13176454; doi:10.1093/toxsci/kfag045)
Supplement: kfag045_Supplementary_Data [file kfag045_supplementary_data.zip › Supplementary File S2.docx]

Table of Contents

[Fetal length (ND), Rt, F, GD0.5-19.5, Lv et al. (2024) 3](#_Toc223541286)

[Fetal tail length (ND), Rt, F, GD0.5-19.5, Lv et al. (2024) 7](#_Toc223541287)

[Fetal weight, Rt, GD6-20, DuPont (2010) 11](#_Toc223541288)

[Male fetal weight, Rt, GD6-20, DuPont (2010) 15](#_Toc223541289)

[Litters w/ skeletal variations (%), Rt, GD6-20, DuPont (2010) 19](#_Toc223541290)

[Litters w/ variations (%), Rt, GD6-20, DuPont (2010) 22](#_Toc223541291)

[Serum cholesterol, Rt, GD8-PND0, Conley et al. (2021) 26](#_Toc223541292)

[Serum triglycerides, Rt, GD8-PND0, Conley et al. (2021) 30](#_Toc223541293)

[Serum glucose, Rt, GD8-PND0, Conley et al. (2021) 34](#_Toc223541294)

[Pup birthweight GD8-PND2, Rt, F, Conley et al. (2021) 38](#_Toc223541295)

[Serum glucose, Rt, GD8-PND2, Conley et al. (2021)_drop 42](#_Toc223541296)

[Rel liver wt, Rt, GD8-PND2, Conley et al. (2021)_drop 46](#_Toc223541297)

[Placenta wt (CD), Rt, F, GD0.5-19.5, Lv et al. (2024) 50](#_Toc223541298)

[Gestational BW gain, Rt, F, GD0.5-19.5, Lv et al. (2024)_drop 53](#_Toc223541299)

[Liver focal necrosis, Rt, F, GD6-20, DuPont (2010)_drop 57](#_Toc223541300)

[Gestational BW gain, Rt, F, GD6-20, DuPont (2010) 60](#_Toc223541301)

[Gestational BW GD20, Rt, F, GD6-20, DuPont (2010) 64](#_Toc223541302)

[Maternal liver wt, Rt, F, GD6-20, DuPont (2010) 69](#_Toc223541303)

[HFPO-DA_Conley19_Dev_Rt_Maternal 73](#_Toc223541304)

[Gestational BW gain, Rt, F, GD14-18, Conley et al. (2019) 73](#_Toc223541305)

[Liver wt, Rt, F, GD14-18, Conley et al. (2019) 77](#_Toc223541306)

[Serum T3, Rt, F, GD14-18, Conley et al. (2019) 81](#_Toc223541307)

[Serum T4, Rt, F, GD14-18, Conley et al. (2019)_drop 85](#_Toc223541308)

[Serum cholesterol, Rt, F, GD14-18, Conley et al. (2019) 89](#_Toc223541309)

[Serum HDL, Rt, F, GD14-18, Conley et al. (2019) 93](#_Toc223541310)

[Serum triglycerides, Rt, F, GD16-20, Conley et al. (2021) 97](#_Toc223541311)

[Serum cholesterol, Rt, F, GD16-20, Conley et al. (2021)_drop 102](#_Toc223541312)

[Serum T4, Rt, F, GD16-20, Conley et al. (2021) 106](#_Toc223541313)

[Serum T3, Rt, F, GD16-20, Conley et al. (2021) 110](#_Toc223541314)

[Relative liver wt, Rt, F, GD16-20, Conley et al. (2021) 114](#_Toc223541315)

[Serum albumin, Rt, F, GD8-PND2, Conley et al. (2021) 118](#_Toc223541316)

[Serum T4, Rt, F, GD8-PND2, Conley et al. (2021) 122](#_Toc223541317)

[Serum T3, Rt, F, GD8-PND2, Conley et al. (2021) 126](#_Toc223541318)

[Liver wt, Rt, F, GD8-PND2, Conley et al. (2021) 130](#_Toc223541319)

[Gestational BW gain, Rt, F, GD8-PND2, Conley et al. (2021) 134](#_Toc223541320)

[BW PND2, Rt, F, GD8-PND2, Conley et al. (2021) 138](#_Toc223541321)

# Fetal length (ND), Rt, F, GD0.5-19.5, Lv et al. (2024)

## Dataset

**Name:** Fetal length (ND), Rt, F, GD0.5-19.5, Lv et al. (2024)

| Dose | N | Mean | Std. Dev. |
| --- | --- | --- | --- |
| 0 | 18 | 53.77 | 1.8 |
| 1 | 18 | 53.29 | 2.28 |
| 10 | 18 | 49.88 | 2.74 |
| 100 | 18 | 47.5 | 2.09 |

Test 1 Dose Response: <0.0001

Test 2 Homogeneity of Variance: 0.3412

Test 3 Variance Model Selection: 0.2267

## Settings

| Setting | Value |
| --- | --- |
| BMR | 5% Relative Deviation |
| Distribution | Normal + Nonconstant variance |
| Adverse Direction | Down (↓) |
| Maximum Polynomial Degree | 3 |
| Confidence Level (one sided) | 0.95 |

## Maximum Likelihood Approach

| Model | BMDL | BMD | BMDU | *P*-Value | AIC | Scaled Residual at Control | Scaled Residual near BMD | Recommendation and Notes |
| --- | --- | --- | --- | --- | --- | --- | --- | --- |
| Exponential 3 | 39.129 | 47.936 | 48.928 | <0.001 | 346.326 | 1.891 | -3.668 | **Questionable** \|Residual near BMD\| > 2.0 Goodness of fit p-value < 0.1 |
| Exponential 5^ab^ | 3.621 | 6.035 | 10.017 | 0.539 | 327.213 | <0.001 | -0. | **Recommended - Lowest AIC** |
| Hill | 23.326 | 23.819 | 30.168 | <0.001 | 339.8 | 1.705 | -2.528 | **Questionable** \|Residual near BMD\| > 2.0 Goodness of fit p-value < 0.1 |
| Polynomial 2 | 41.265 | 49.843 | 50.874 | <0.001 | 346.268 | 1.961 | -3.576 | **Questionable** \|Residual near BMD\| > 2.0 Goodness of fit p-value < 0.1 |
| Polynomial 3 | 41.011 | 49.748 | 50.777 | <0.001 | 346.472 | 1.929 | -3.61 | **Questionable** \|Residual near BMD\| > 2.0 Goodness of fit p-value < 0.1 |
| Power | 42.159 | 50.775 | 64.33 | <0.001 | 345.596 | 1.927 | -3.447 | **Questionable** \|Residual near BMD\| > 2.0 Goodness of fit p-value < 0.1 Control stdev. fit > 1.5 |
| Linear | 42.159 | 50.781 | 63.276 | <0.001 | 345.596 | 1.926 | -3.445 | **Questionable** \|Residual near BMD\| > 2.0 Goodness of fit p-value < 0.1 Control stdev. fit > 1.5 |

^a^ BMDS recommended best fitting model

^b^ User selected best fitting model


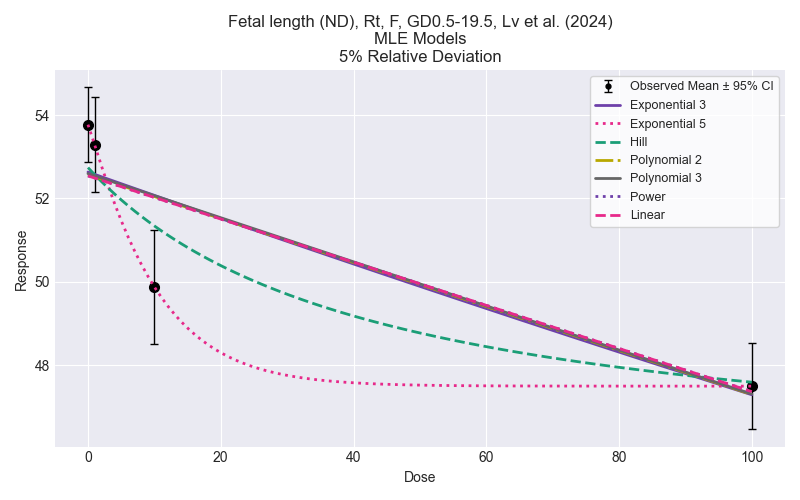
Selected Model: Exponential 5


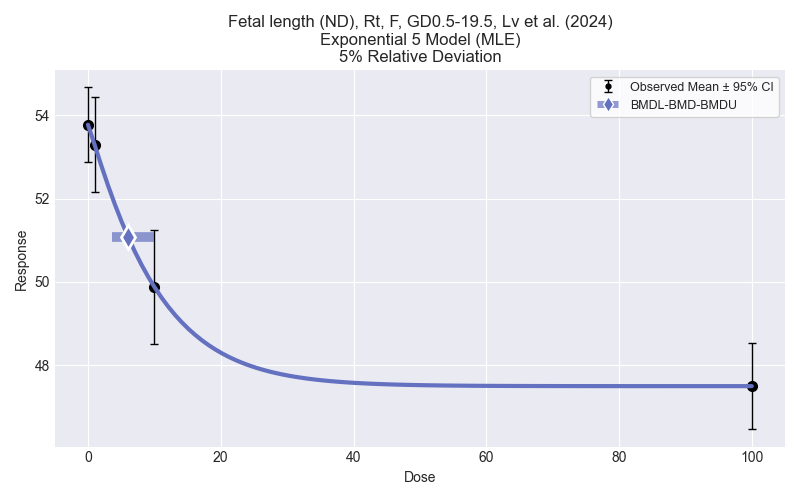


Exponential 5 Model
══════════════════════════════

Version: pybmds 25.1 (bmdscore 25.1)

Input Summary:
╒══════════════════════════════╤═══════════════════════════════╕
│ BMR │ 5% Relative Deviation │
│ Distribution │ Normal + Nonconstant variance │
│ Modeling Direction │ Down (↓) │
│ Confidence Level (one sided) │ 0.95 │
│ Modeling Approach │ MLE │
╘══════════════════════════════╧═══════════════════════════════╛

Parameter Settings:
╒═════════════╤═══════════╤═══════╤═══════╕
│ Parameter │ Initial │ Min │ Max │
╞═════════════╪═══════════╪═══════╪═══════╡
│ a │ 0 │ 0 │ 100 │
│ b │ 0 │ 0 │ 100 │
│ c │ 0 │ -20 │ 0 │
│ d │ 1 │ 1 │ 18 │
│ rho │ 0 │ 0 │ 18 │
│ log-alpha │ 0 │ -18 │ 18 │
╘═════════════╧═══════════╧═══════╧═══════╛

Modeling Summary:
╒════════════════╤═════════════╕
│ BMD │ 6.0347 │
│ BMDL │ 3.62063 │
│ BMDU │ 10.0174 │
│ AIC │ 327.213 │
│ Log-Likelihood │ -158.607 │
│ P-Value │ 0.538779 │
│ Model d.f. │ 1 │
╘════════════════╧═════════════╛

Model Parameters:
╒════════════╤════════════╤════════════╤══════════════╕
│ Variable │ Estimate │ On Bound │ Std Error │
╞════════════╪════════════╪════════════╪══════════════╡
│ a │ 53.77 │ no │ 0.516109 │
│ b │ 0.0971083 │ no │ 0.0247446 │
│ c │ 0.883391 │ no │ 0.0145075 │
│ d │ 1.08502 │ no │ 0.644827 │
│ rho │ 0 │ yes │ Not Reported │
│ log-alpha │ 1.56786 │ no │ 0.166663 │
╘════════════╧════════════╧════════════╧══════════════╛
Standard errors estimates are not generated for parameters estimated on corresponding bounds,
although sampling error is present for all parameters, as a rule. Standard error estimates may not
be reliable as a basis for confidence intervals or tests when one or more parameters are on bounds.


Goodness of Fit:
╒════════╤═════╤═══════════════╤═════════════════════╤═══════════════════╕
│ Dose │ N │ Sample Mean │ Model Fitted Mean │ Scaled Residual │
╞════════╪═════╪═══════════════╪═════════════════════╪═══════════════════╡
│ 0 │ 18 │ 53.77 │ 53.77 │ 2.129e-07 │
│ 1 │ 18 │ 53.29 │ 53.29 │ -5.9578e-08 │
│ 10 │ 18 │ 49.88 │ 49.88 │ -1.22988e-07 │
│ 100 │ 18 │ 47.5 │ 47.5 │ 9.65577e-08 │
╘════════╧═════╧═══════════════╧═════════════════════╧═══════════════════╛
╒════════╤═════╤═════════════╤═══════════════════╕
│ Dose │ N │ Sample SD │ Model Fitted SD │
╞════════╪═════╪═════════════╪═══════════════════╡
│ 0 │ 18 │ 1.8 │ 2.19007 │
│ 1 │ 18 │ 2.28 │ 2.19007 │
│ 10 │ 18 │ 2.74 │ 2.19007 │
│ 100 │ 18 │ 2.09 │ 2.19007 │
╘════════╧═════╧═════════════╧═══════════════════╛

Likelihoods:
╒═════════╤══════════════════╤════════════╤═════════╕
│ Model │ Log-Likelihood │ # Params │ AIC │
╞═════════╪══════════════════╪════════════╪═════════╡
│ A1 │ -158.607 │ 5 │ 327.213 │
│ A2 │ -156.933 │ 8 │ 329.867 │
│ A3 │ -158.418 │ 6 │ 328.835 │
│ fitted │ -158.607 │ 5 │ 327.213 │
│ reduced │ -189.741 │ 2 │ 383.482 │
╘═════════╧══════════════════╧════════════╧═════════╛

Tests of Mean and Variance Fits:
╒════════╤══════════════════════════════╤═════════════╤════════════╕
│ Name │ -2 * Log(Likelihood Ratio) │ Test d.f. │ P-Value │
╞════════╪══════════════════════════════╪═════════════╪════════════╡
│ Test 1 │ 65.615 │ 6 │ 3.2303e-12 │
│ Test 2 │ 3.34647 │ 3 │ 0.341226 │
│ Test 3 │ 2.96867 │ 2 │ 0.226653 │
│ Test 4 │ 0.377807 │ 1 │ 0.538779 │
╘════════╧══════════════════════════════╧═════════════╧════════════╛
Test 1: Test the null hypothesis that responses and variances don't differ among dose levels
(A2 vs R). If this test fails to reject the null hypothesis (p-value > 0.05), there may not be
a dose-response.

Test 2: Test the null hypothesis that variances are homogenous (A1 vs A2). If this test fails to
reject the null hypothesis (p-value > 0.05), the simpler constant variance model may be appropriate.

Test 3: Test the null hypothesis that the variances are adequately modeled (A3 vs A2). If this test
fails to reject the null hypothesis (p-value > 0.05), it may be inferred that the variances have
been modeled appropriately.

Test 4: Test the null hypothesis that the model for the mean fits the data (Fitted vs A3). If this
test fails to reject the null hypothesis (p-value > 0.1), the user has support for use of the
selected model.

# Fetal tail length (ND), Rt, F, GD0.5-19.5, Lv et al. (2024)

## Dataset

**Name:** Fetal tail length (ND), Rt, F, GD0.5-19.5, Lv et al. (2024)

| Dose | N | Mean | Std. Dev. |
| --- | --- | --- | --- |
| 0 | 18 | 16.38 | 1.28 |
| 1 | 18 | 16.58 | 2.34 |
| 10 | 18 | 15.5 | 0.7 |
| 100 | 18 | 14.38 | 0.97 |

Test 1 Dose Response: <0.0001

Test 2 Homogeneity of Variance: <0.0001

Test 3 Variance Model Selection: <0.0001

## Settings

| Setting | Value |
| --- | --- |
| BMR | 5% Relative Deviation |
| Distribution | Normal + Constant variance |
| Adverse Direction | Down (↓) |
| Maximum Polynomial Degree | 3 |
| Confidence Level (one sided) | 0.95 |

## Maximum Likelihood Approach

| Model | BMDL | BMD | BMDU | *P*-Value | AIC | Scaled Residual at Control | Scaled Residual near BMD | Recommendation and Notes |
| --- | --- | --- | --- | --- | --- | --- | --- | --- |
| Exponential 3 | 8.88 | 9.803 | 10.275 | 0 | 499.158 | -0.058 | -0. | **Questionable** Goodness of fit p-value < 0.1 Control stdev. fit > 1.5 Constant variance test failed (Test 2 p-value < 0.05) |
| Exponential 5 | 3.489 | 9.758 | 28.359 | - | 265.041 | -0.298 | <0.001 | **Questionable** Zero degrees of freedom; saturated model Constant variance test failed (Test 2 p-value < 0.05) |
| Hill | 91.934 | 94.186 | 97.632 | - | 270.525 | 0.651 | <0.001 | **Questionable** Zero degrees of freedom; saturated model Constant variance test failed (Test 2 p-value < 0.05) |
| Polynomial 2 | 31.638 | 42.482 | 77.057 | 0.14 | 264.79 | 0.406 | -1.596 | **Questionable** Constant variance test failed (Test 2 p-value < 0.05) |
| Polynomial 3 | 31.639 | 42.565 | 83.649 | 0.14 | 264.79 | 0.409 | -1.594 | **Questionable** Constant variance test failed (Test 2 p-value < 0.05) |
| Power | 31.638 | 42.472 | 90.905 | 0.14 | 264.79 | 0.405 | -1.597 | **Questionable** Constant variance test failed (Test 2 p-value < 0.05) |
| Linear | 31.638 | 42.472 | 65.28 | 0.14 | 264.79 | 0.405 | -1.597 | **Questionable** Constant variance test failed (Test 2 p-value < 0.05) |


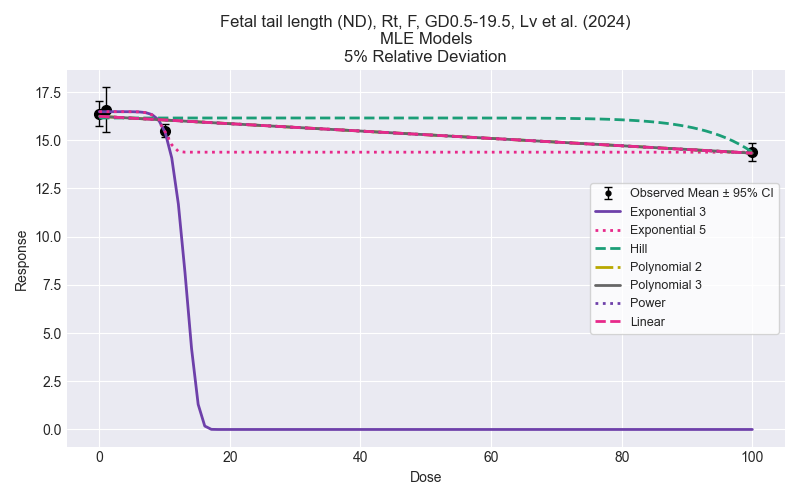


## Selected Model

No model was selected as a best-fitting model.

## Session for Fetal tail length (ND), Rt, F, GD0.5-19.5, Lv et al. (2024)

## Dataset

**Name:** Fetal tail length (ND), Rt, F, GD0.5-19.5, Lv et al. (2024)

| Dose | N | Mean | Std. Dev. |
| --- | --- | --- | --- |
| 0 | 18 | 16.38 | 1.28 |
| 1 | 18 | 16.58 | 2.34 |
| 10 | 18 | 15.5 | 0.7 |
| 100 | 18 | 14.38 | 0.97 |

## Settings

| Setting | Value |
| --- | --- |
| BMR | 5% Relative Deviation |
| Distribution | Normal + Nonconstant variance |
| Adverse Direction | Down (↓) |
| Maximum Polynomial Degree | 3 |
| Confidence Level (one sided) | 0.95 |

## Maximum Likelihood Approach

| Model | BMDL | BMD | BMDU | *P*-Value | AIC | Scaled Residual at Control | Scaled Residual near BMD | Recommendation and Notes |
| --- | --- | --- | --- | --- | --- | --- | --- | --- |
| Exponential 3 | - | 6.32 | - | - | - | 0 | 750.39 | **Unusable** Did not successfully execute. |
| Exponential 5 | 3.328 | 9.123 | 10.47 | 0.013 | 252.785 | -0.73 | 0.723 | **Questionable** Nonconstant variance test failed (Test 3 p-value < 0.05) Goodness of fit p-value < 0.1 |
| Hill | 7.783 | 8.865 | 9.067 | - | 254.785 | -0.73 | 0.723 | **Questionable** Zero degrees of freedom; saturated model Nonconstant variance test failed (Test 3 p-value < 0.05) |
| Polynomial 2 | 33.751 | 41.678 | 70.155 | <0.001 | 260.111 | 0.324 | -1.588 | **Questionable** Nonconstant variance test failed (Test 3 p-value < 0.05) Goodness of fit p-value < 0.1 |
| Polynomial 3 | - | 72.879 | - | - | 271.679 | 0.613 | 0.069 | **Unusable** Did not successfully execute. |
| Power | - | 41.904 | - | <0.001 | 266.054 | 0.383 | -1.609 | **Unusable** Did not successfully execute. |
| Linear | 33.751 | 41.678 | 56.166 | <0.001 | 260.111 | 0.324 | -1.588 | **Questionable** Nonconstant variance test failed (Test 3 p-value < 0.05) Goodness of fit p-value < 0.1 |

## Selected Model

No model was selected as a best-fitting model.

## Session for Fetal tail length (ND), Rt, F, GD0.5-19.5, Lv et al. (2024)

## Dataset

**Name:** Fetal tail length (ND), Rt, F, GD0.5-19.5, Lv et al. (2024)

| Dose | N | Mean | Std. Dev. |
| --- | --- | --- | --- |
| 0 | 18 | 16.38 | 1.28 |
| 1 | 18 | 16.58 | 2.34 |
| 10 | 18 | 15.5 | 0.7 |
| 100 | 18 | 14.38 | 0.97 |

Test 1 Dose Response: <0.0001

Test 2 Homogeneity of Variance: <0.0001

Test 3 Variance Model Selection: <0.0001

## Settings

| Setting | Value |
| --- | --- |
| BMR | 5% Relative Deviation |
| Distribution | Lognormal + Constant variance |
| Adverse Direction | Down (↓) |
| Maximum Polynomial Degree | 3 |
| Confidence Level (one sided) | 0.95 |

## Maximum Likelihood Approach

| Model | BMDL | BMD | BMDU | *P*-Value | AIC | Scaled Residual at Control | Scaled Residual near BMD | Recommendation and Notes |
| --- | --- | --- | --- | --- | --- | --- | --- | --- |
| Exponential 3 | 31.353 | 42.115 | 96.984 | 0.214 | 258.339 | 0.855 | -1.8 | **Questionable** Constant variance test failed (Test 2 p-value < 0.05) |
| Exponential 5 | 3.722 | 9.838 | 37.013 | - | 259.285 | 0.023 | 0.06 | **Questionable** Zero degrees of freedom; saturated model Constant variance test failed (Test 2 p-value < 0.05) |


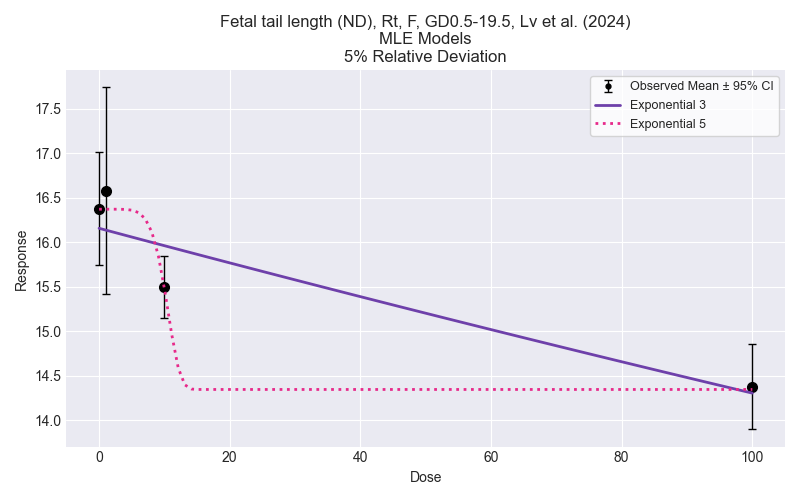


## Selected Model

No model was selected as a best-fitting model.

# Fetal weight, Rt, GD6-20, DuPont (2010)

## Dataset

**Name:** Fetal weight, Rt, GD6-20, DuPont (2010)

| Dose | N | Mean | Std. Dev. |
| --- | --- | --- | --- |
| 0 | 22 | 5.7 | 0.38 |
| 10 | 21 | 5.6 | 0.24 |
| 100 | 21 | 5.2 | 0.24 |
| 1000 | 21 | 4.1 | 0.29 |

Test 1 Dose Response: 0

Test 2 Homogeneity of Variance: 0.0881

Test 3 Variance Model Selection: 0.0881

## Settings

| Setting | Value |
| --- | --- |
| BMR | 5% Relative Deviation |
| Distribution | Normal + Constant variance |
| Adverse Direction | Down (↓) |
| Maximum Polynomial Degree | 3 |
| Confidence Level (one sided) | 0.95 |

## Maximum Likelihood Approach

| Model | BMDL | BMD | BMDU | *P*-Value | AIC | Scaled Residual at Control | Scaled Residual near BMD | Recommendation and Notes |
| --- | --- | --- | --- | --- | --- | --- | --- | --- |
| Exponential 3 | 148.552 | 163.648 | 194.407 | <0.001 | 49.252 | 1.901 | -2.96 | **Questionable** \|Residual near BMD\| > 2.0 Goodness of fit p-value < 0.1 |
| Exponential 5 | 40.466 | 54.091 | 99.501 | 0.614 | 37.474 | 0.316 | -0.382 | **Viable** |
| Hill^ab^ | 36.793 | 51.924 | 80.609 | 0.656 | 37.418 | 0.28 | -0.341 | **Recommended - Lowest AIC** |
| Polynomial 2 | 172.405 | 187.62 | 230.474 | <0.001 | 51.141 | 2.036 | -3.119 | **Questionable** \|Residual near BMD\| > 2.0 Residual at control > 2.0 Goodness of fit p-value < 0.1 |
| Polynomial 3 | 172.491 | 187.159 | 235.751 | <0.001 | 51.141 | 2.019 | -3.127 | **Questionable** \|Residual near BMD\| > 2.0 Residual at control > 2.0 Goodness of fit p-value < 0.1 |
| Power | 172.451 | 187.377 | 222.096 | <0.001 | 51.14 | 2.027 | -3.124 | **Questionable** \|Residual near BMD\| > 2.0 Residual at control > 2.0 Goodness of fit p-value < 0.1 |
| Linear | 172.449 | 187.377 | 205.36 | <0.001 | 51.14 | 2.027 | -3.124 | **Questionable** \|Residual near BMD\| > 2.0 Residual at control > 2.0 Goodness of fit p-value < 0.1 |

^a^ BMDS recommended best fitting model

^b^ User selected best fitting model


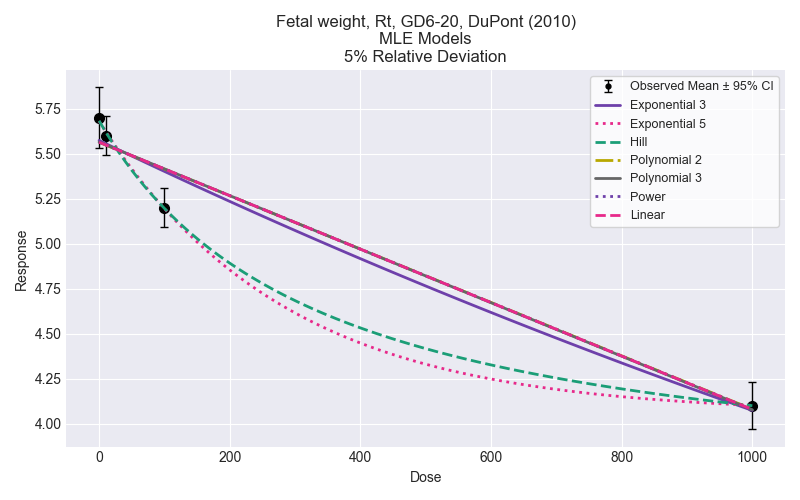


## Selected Model: Hill


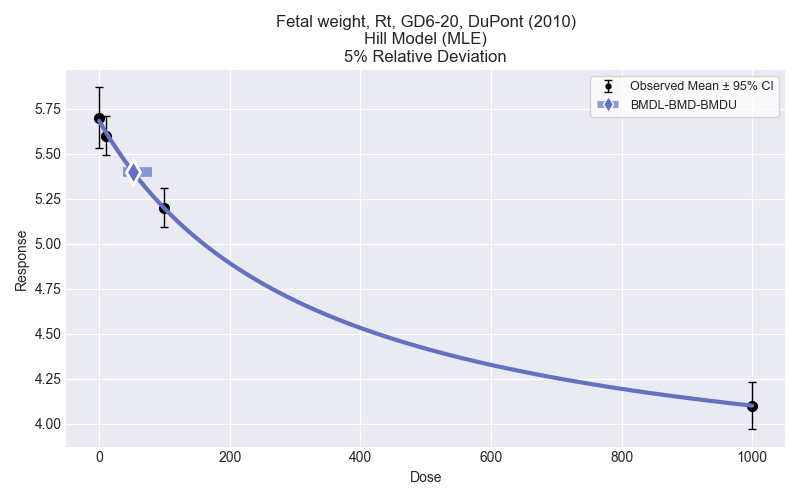


Hill Model
══════════════════════════════

Version: pybmds 25.1 (bmdscore 25.1)

Input Summary:
╒══════════════════════════════╤════════════════════════════╕
│ BMR │ 5% Relative Deviation │
│ Distribution │ Normal + Constant variance │
│ Modeling Direction │ Down (↓) │
│ Confidence Level (one sided) │ 0.95 │
│ Modeling Approach │ MLE │
╘══════════════════════════════╧════════════════════════════╛

Parameter Settings:
╒═════════════╤═══════════╤═══════╤═══════╕
│ Parameter │ Initial │ Min │ Max │
╞═════════════╪═══════════╪═══════╪═══════╡
│ g │ 0 │ -100 │ 100 │
│ v │ 0 │ -100 │ 100 │
│ k │ 0 │ 0 │ 5 │
│ n │ 1 │ 1 │ 18 │
│ alpha │ 0 │ -18 │ 18 │
╘═════════════╧═══════════╧═══════╧═══════╛

Modeling Summary:
╒════════════════╤════════════╕
│ BMD │ 51.9245 │
│ BMDL │ 36.7933 │
│ BMDU │ 80.6091 │
│ AIC │ 37.4185 │
│ Log-Likelihood │ -14.7092 │
│ P-Value │ 0.655721 │
│ Model d.f. │ 1 │
╘════════════════╧════════════╛

Model Parameters:
╒════════════╤═════════════╤════════════╤══════════════╕
│ Variable │ Estimate │ On Bound │ Std Error │
╞════════════╪═════════════╪════════════╪══════════════╡
│ g │ 5.68282 │ no │ 0.0477608 │
│ v │ -2.11073 │ no │ 0.180704 │
│ k │ 333.794 │ no │ 100.995 │
│ n │ 1 │ yes │ Not Reported │
│ alpha │ 0.0827627 │ no │ 0.00105069 │
╘════════════╧═════════════╧════════════╧══════════════╛
Standard errors estimates are not generated for parameters estimated on corresponding bounds,
although sampling error is present for all parameters, as a rule. Standard error estimates may not
be reliable as a basis for confidence intervals or tests when one or more parameters are on bounds.


Goodness of Fit:
╒════════╤═════╤═══════════════╤═════════════════════╤═══════════════════╕
│ Dose │ N │ Sample Mean │ Model Fitted Mean │ Scaled Residual │
╞════════╪═════╪═══════════════╪═════════════════════╪═══════════════════╡
│ 0 │ 22 │ 5.7 │ 5.68282 │ 0.280077 │
│ 10 │ 21 │ 5.6 │ 5.62143 │ -0.341304 │
│ 100 │ 21 │ 5.2 │ 5.19625 │ 0.0597733 │
│ 1000 │ 21 │ 4.1 │ 4.10032 │ -0.0051373 │
╘════════╧═════╧═══════════════╧═════════════════════╧═══════════════════╛
╒════════╤═════╤═════════════╤═══════════════════╕
│ Dose │ N │ Sample SD │ Model Fitted SD │
╞════════╪═════╪═════════════╪═══════════════════╡
│ 0 │ 22 │ 0.38 │ 0.287685 │
│ 10 │ 21 │ 0.24 │ 0.287685 │
│ 100 │ 21 │ 0.24 │ 0.287685 │
│ 1000 │ 21 │ 0.29 │ 0.287685 │
╘════════╧═════╧═════════════╧═══════════════════╛

Likelihoods:
╒═════════╤══════════════════╤════════════╤══════════╕
│ Model │ Log-Likelihood │ # Params │ AIC │
╞═════════╪══════════════════╪════════════╪══════════╡
│ A1 │ -14.6098 │ 5 │ 39.2197 │
│ A2 │ -11.3401 │ 8 │ 38.6801 │
│ A3 │ -14.6098 │ 5 │ 39.2197 │
│ fitted │ -14.7092 │ 4 │ 37.4185 │
│ reduced │ -89.7557 │ 2 │ 183.511 │
╘═════════╧══════════════════╧════════════╧══════════╛

Tests of Mean and Variance Fits:
╒════════╤══════════════════════════════╤═════════════╤═══════════╕
│ Name │ -2 * Log(Likelihood Ratio) │ Test d.f. │ P-Value │
╞════════╪══════════════════════════════╪═════════════╪═══════════╡
│ Test 1 │ 156.831 │ 6 │ 0 │
│ Test 2 │ 6.53956 │ 3 │ 0.0881155 │
│ Test 3 │ 6.53956 │ 3 │ 0.0881155 │
│ Test 4 │ 0.198763 │ 1 │ 0.655721 │
╘════════╧══════════════════════════════╧═════════════╧═══════════╛
Test 1: Test the null hypothesis that responses and variances don't differ among dose levels
(A2 vs R). If this test fails to reject the null hypothesis (p-value > 0.05), there may not be
a dose-response.

Test 2: Test the null hypothesis that variances are homogenous (A1 vs A2). If this test fails to
reject the null hypothesis (p-value > 0.05), the simpler constant variance model may be appropriate.

Test 3: Test the null hypothesis that the variances are adequately modeled (A3 vs A2). If this test
fails to reject the null hypothesis (p-value > 0.05), it may be inferred that the variances have
been modeled appropriately.

Test 4: Test the null hypothesis that the model for the mean fits the data (Fitted vs A3). If this
test fails to reject the null hypothesis (p-value > 0.1), the user has support for use of the
selected model.

# Male fetal weight, Rt, GD6-20, DuPont (2010)

## Dataset

**Name:** Male fetal weight, Rt, GD6-20, DuPont (2010)

| Dose | N | Mean | Std. Dev. |
| --- | --- | --- | --- |
| 0 | 21 | 5.9 | 0.3 |
| 10 | 21 | 5.8 | 0.28 |
| 100 | 21 | 5.3 | 0.21 |
| 1000 | 21 | 4.2 | 0.31 |

Test 1 Dose Response: 0

Test 2 Homogeneity of Variance: 0.3131

Test 3 Variance Model Selection: 0.3131

## Settings

| Setting | Value |
| --- | --- |
| BMR | 5% Relative Deviation |
| Distribution | Normal + Constant variance |
| Adverse Direction | Down (↓) |
| Maximum Polynomial Degree | 3 |
| Confidence Level (one sided) | 0.95 |

## Maximum Likelihood Approach

| Model | BMDL | BMD | BMDU | *P*-Value | AIC | Scaled Residual at Control | Scaled Residual near BMD | Recommendation and Notes |
| --- | --- | --- | --- | --- | --- | --- | --- | --- |
| Exponential 3 | 145.385 | 159.664 | 183.749 | <0.001 | 49.654 | 2.275 | -3.836 | **Questionable** \|Residual near BMD\| > 2.0 Residual at control > 2.0 Goodness of fit p-value < 0.1 |
| Exponential 5 | 35.125 | 44.759 | 97.913 | 0.723 | 27.2 | 0.229 | -0.266 | **Viable** |
| Hill^ab^ | 31.128 | 41.718 | 63.737 | 0.799 | 27.14 | 0.166 | -0.191 | **Recommended - Lowest AIC** |
| Polynomial 2 | 169.731 | 183.915 | 216.562 | <0.001 | 52.231 | 2.41 | -3.979 | **Questionable** \|Residual near BMD\| > 2.0 Residual at control > 2.0 Goodness of fit p-value < 0.1 |
| Polynomial 3 | 169.629 | 184.325 | 219.707 | <0.001 | 52.234 | 2.424 | -3.969 | **Questionable** \|Residual near BMD\| > 2.0 Residual at control > 2.0 Goodness of fit p-value < 0.1 |
| Power | 169.387 | 183.791 | 212.139 | <0.001 | 52.353 | 2.336 | -3.878 | **Questionable** \|Residual near BMD\| > 2.0 Residual at control > 2.0 Goodness of fit p-value < 0.1 |
| Linear | 169.764 | 183.784 | 200.738 | <0.001 | 52.231 | 2.405 | -3.982 | **Questionable** \|Residual near BMD\| > 2.0 Residual at control > 2.0 Goodness of fit p-value < 0.1 |

^a^ BMDS recommended best fitting model

^b^ User selected best fitting model


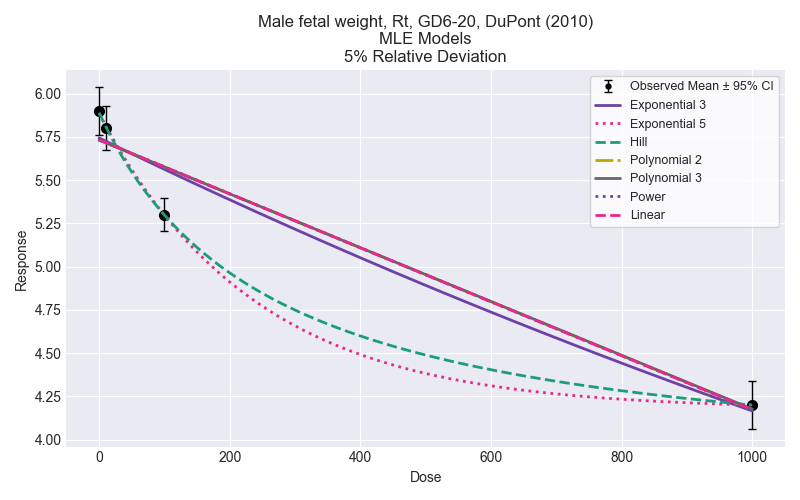


## Selected Model: Hill


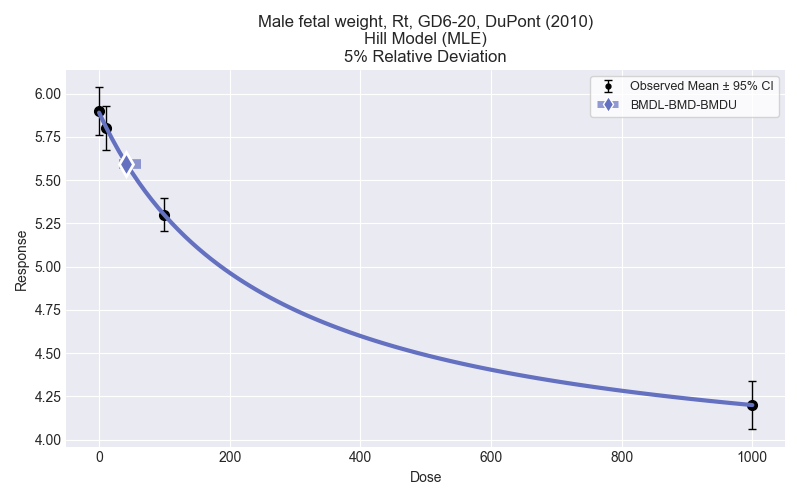


Hill Model
══════════════════════════════

Version: pybmds 25.1 (bmdscore 25.1)

Input Summary:
╒══════════════════════════════╤════════════════════════════╕
│ BMR │ 5% Relative Deviation │
│ Distribution │ Normal + Constant variance │
│ Modeling Direction │ Down (↓) │
│ Confidence Level (one sided) │ 0.95 │
│ Modeling Approach │ MLE │
╘══════════════════════════════╧════════════════════════════╛

Parameter Settings:
╒═════════════╤═══════════╤═══════╤═══════╕
│ Parameter │ Initial │ Min │ Max │
╞═════════════╪═══════════╪═══════╪═══════╡
│ g │ 0 │ -100 │ 100 │
│ v │ 0 │ -100 │ 100 │
│ k │ 0 │ 0 │ 5 │
│ n │ 1 │ 1 │ 18 │
│ alpha │ 0 │ -18 │ 18 │
╘═════════════╧═══════════╧═══════╧═══════╛

Modeling Summary:
╒════════════════╤═══════════╕
│ BMD │ 41.718 │
│ BMDL │ 31.1277 │
│ BMDU │ 63.7375 │
│ AIC │ 27.1396 │
│ Log-Likelihood │ -9.56979 │
│ P-Value │ 0.799392 │
│ Model d.f. │ 1 │
╘════════════════╧═══════════╛

Model Parameters:
╒════════════╤═════════════╤════════════╤══════════════╕
│ Variable │ Estimate │ On Bound │ Std Error │
╞════════════╪═════════════╪════════════╪══════════════╡
│ g │ 5.89018 │ no │ 0.0460471 │
│ v │ -2.1296 │ no │ 0.133811 │
│ k │ 259.946 │ no │ 61.6497 │
│ n │ 1 │ yes │ Not Reported │
│ alpha │ 0.0735523 │ no │ 0.000834887 │
╘════════════╧═════════════╧════════════╧══════════════╛
Standard errors estimates are not generated for parameters estimated on corresponding bounds,
although sampling error is present for all parameters, as a rule. Standard error estimates may not
be reliable as a basis for confidence intervals or tests when one or more parameters are on bounds.


Goodness of Fit:
╒════════╤═════╤═══════════════╤═════════════════════╤═══════════════════╕
│ Dose │ N │ Sample Mean │ Model Fitted Mean │ Scaled Residual │
╞════════╪═════╪═══════════════╪═════════════════════╪═══════════════════╡
│ 0 │ 21 │ 5.9 │ 5.89018 │ 0.165916 │
│ 10 │ 21 │ 5.8 │ 5.81129 │ -0.190782 │
│ 100 │ 21 │ 5.3 │ 5.29854 │ 0.0247401 │
│ 1000 │ 21 │ 4.2 │ 4.19995 │ 0.000905622 │
╘════════╧═════╧═══════════════╧═════════════════════╧═══════════════════╛
╒════════╤═════╤═════════════╤═══════════════════╕
│ Dose │ N │ Sample SD │ Model Fitted SD │
╞════════╪═════╪═════════════╪═══════════════════╡
│ 0 │ 21 │ 0.3 │ 0.271205 │
│ 10 │ 21 │ 0.28 │ 0.271205 │
│ 100 │ 21 │ 0.21 │ 0.271205 │
│ 1000 │ 21 │ 0.31 │ 0.271205 │
╘════════╧═════╧═════════════╧═══════════════════╛

Likelihoods:
╒═════════╤══════════════════╤════════════╤══════════╕
│ Model │ Log-Likelihood │ # Params │ AIC │
╞═════════╪══════════════════╪════════════╪══════════╡
│ A1 │ -9.53749 │ 5 │ 29.075 │
│ A2 │ -7.7575 │ 8 │ 31.515 │
│ A3 │ -9.53749 │ 5 │ 29.075 │
│ fitted │ -9.56979 │ 4 │ 27.1396 │
│ reduced │ -92.405 │ 2 │ 188.81 │
╘═════════╧══════════════════╧════════════╧══════════╛

Tests of Mean and Variance Fits:
╒════════╤══════════════════════════════╤═════════════╤═══════════╕
│ Name │ -2 * Log(Likelihood Ratio) │ Test d.f. │ P-Value │
╞════════╪══════════════════════════════╪═════════════╪═══════════╡
│ Test 1 │ 169.295 │ 6 │ 0 │
│ Test 2 │ 3.56 │ 3 │ 0.313064 │
│ Test 3 │ 3.56 │ 3 │ 0.313064 │
│ Test 4 │ 0.0645841 │ 1 │ 0.799392 │
╘════════╧══════════════════════════════╧═════════════╧═══════════╛
Test 1: Test the null hypothesis that responses and variances don't differ among dose levels
(A2 vs R). If this test fails to reject the null hypothesis (p-value > 0.05), there may not be
a dose-response.

Test 2: Test the null hypothesis that variances are homogenous (A1 vs A2). If this test fails to
reject the null hypothesis (p-value > 0.05), the simpler constant variance model may be appropriate.

Test 3: Test the null hypothesis that the variances are adequately modeled (A3 vs A2). If this test
fails to reject the null hypothesis (p-value > 0.05), it may be inferred that the variances have
been modeled appropriately.

Test 4: Test the null hypothesis that the model for the mean fits the data (Fitted vs A3). If this
test fails to reject the null hypothesis (p-value > 0.1), the user has support for use of the
selected model.

# Litters w/ skeletal variations (%), Rt, GD6-20, DuPont (2010)

## Dataset

**Name:** Litters w/ skeletal variations (%), Rt, GD6-20, DuPont (2010)

| Dose | N | Mean | Std. Dev. |
| --- | --- | --- | --- |
| 0 | 22 | 10.6 | 14.17 |
| 10 | 21 | 11.6 | 10.9 |
| 100 | 21 | 14.7 | 10.42 |
| 1000 | 21 | 30.3 | 27.49 |

## Settings

| Setting | Value |
| --- | --- |
| BMR | 5% Relative Deviation |
| Distribution | Normal + Nonconstant variance |
| Adverse Direction | Up (↑) |
| Maximum Polynomial Degree | 3 |
| Confidence Level (one sided) | 0.95 |

## Maximum Likelihood Approach

| Model | BMDL | BMD | BMDU | *P*-Value | AIC | Scaled Residual at Control | Scaled Residual near BMD | Recommendation and Notes |
| --- | --- | --- | --- | --- | --- | --- | --- | --- |
| Exponential 3 | - | 147.073 | - | -1 | - | -0.002 | 0.003 | **Unusable** Did not successfully execute. |
| Exponential 5 | 12.221 | 120.175 | 872 | - | 707.632 | -0.647 | 0.914 | **Questionable** Zero degrees of freedom; saturated model BMD/BMDL ratio > 3.0 |
| Hill | 89.035 | 118.566 | 263.544 | - | 707.632 | -0.647 | 0.914 | **Questionable** Zero degrees of freedom; saturated model |
| Polynomial 2 | 19.059 | 183.11 | 285.268 | 0.669 | 703.635 | -0.634 | 0.892 | **Viable** BMD/BMDL ratio > 3.0 |
| Polynomial 3^ab^ | 19.064 | 249.33 | 436.323 | 0.67 | 703.632 | -0.65 | 0.918 | **Recommended - Lowest AIC** BMD/BMDL ratio > 3.0 |
| Power | 19.066 | 233.841 | 873.334 | 0.371 | 705.632 | -0.649 | 0.918 | **Viable** BMD/BMDL ratio > 3.0 |
| Linear | 17.567 | 30.921 | 73.882 | 0.455 | 704.405 | -0.394 | -0.059 | **Viable** |

^a^ BMDS recommended best fitting model

^b^ User selected best fitting model

## Selected Model: Polynomial 3


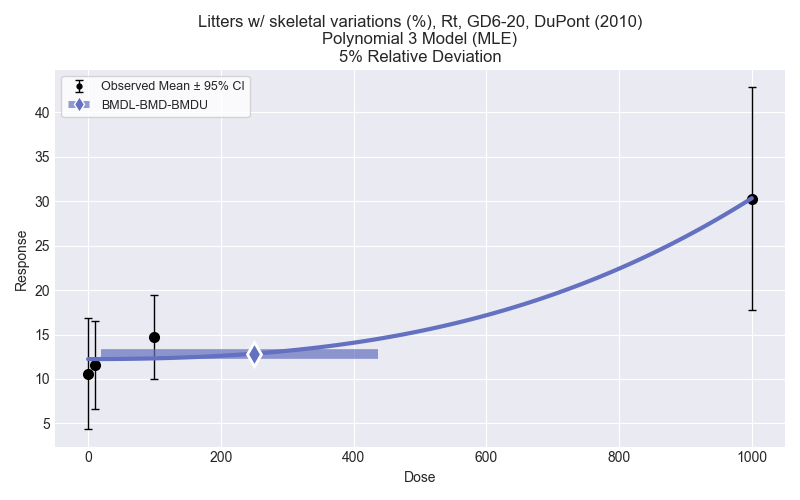


Polynomial 3 Model
══════════════════════════════

Version: pybmds 25.1 (bmdscore 25.1)

Input Summary:
╒══════════════════════════════╤═══════════════════════════════╕
│ BMR │ 5% Relative Deviation │
│ Distribution │ Normal + Nonconstant variance │
│ Modeling Direction │ Up (↑) │
│ Confidence Level (one sided) │ 0.95 │
│ Modeling Approach │ MLE │
│ Degree │ 3 │
╘══════════════════════════════╧═══════════════════════════════╛

Parameter Settings:
╒═════════════╤═══════════╤═══════╤══════════╕
│ Parameter │ Initial │ Min │ Max │
╞═════════════╪═══════════╪═══════╪══════════╡
│ g │ 0 │ 0 │ 1000 │
│ b1 │ 0 │ 0 │ 18 │
│ b2 │ 0 │ 0 │ 1e+06 │
│ b3 │ 0 │ 0 │ 1e+06 │
│ rho │ 0 │ 0 │ 18 │
│ alpha │ 0 │ -18 │ 18 │
╘═════════════╧═══════════╧═══════╧══════════╛

Modeling Summary:
╒════════════════╤═════════════╕
│ BMD │ 249.33 │
│ BMDL │ 19.0638 │
│ BMDU │ 436.323 │
│ AIC │ 703.632 │
│ Log-Likelihood │ -347.816 │
│ P-Value │ 0.669863 │
│ Model d.f. │ 2 │
╘════════════════╧═════════════╛

Model Parameters:
╒════════════╤══════════════╤════════════╤══════════════╕
│ Variable │ Estimate │ On Bound │ Std Error │
╞════════════╪══════════════╪════════════╪══════════════╡
│ g │ 12.2364 │ no │ 1.49273 │
│ b1 │ 0 │ yes │ Not Reported │
│ b2 │ 7.09898e-06 │ no │ 6.45044e-05 │
│ b3 │ 1.10008e-08 │ yes │ Not Reported │
│ rho │ 1.80533 │ no │ 0.582931 │
│ alpha │ 1.51708 │ no │ 3.72427 │
╘════════════╧══════════════╧════════════╧══════════════╛
Standard errors estimates are not generated for parameters estimated on corresponding bounds,
although sampling error is present for all parameters, as a rule. Standard error estimates may not
be reliable as a basis for confidence intervals or tests when one or more parameters are on bounds.


Goodness of Fit:
╒════════╤═════╤═══════════════╤═════════════════════╤═══════════════════╕
│ Dose │ N │ Sample Mean │ Model Fitted Mean │ Scaled Residual │
╞════════╪═════╪═══════════════╪═════════════════════╪═══════════════════╡
│ 0 │ 22 │ 10.6 │ 12.2364 │ -0.649851 │
│ 10 │ 21 │ 11.6 │ 12.2371 │ -0.247186 │
│ 100 │ 21 │ 14.7 │ 12.3184 │ 0.918485 │
│ 1000 │ 21 │ 30.3 │ 30.3361 │ -0.00618 │
╘════════╧═════╧═══════════════╧═════════════════════╧═══════════════════╛
╒════════╤═════╤═════════════╤═══════════════════╕
│ Dose │ N │ Sample SD │ Model Fitted SD │
╞════════╪═════╪═════════════╪═══════════════════╡
│ 0 │ 22 │ 14.17 │ 11.8111 │
│ 10 │ 21 │ 10.9 │ 11.8117 │
│ 100 │ 21 │ 10.42 │ 11.8825 │
│ 1000 │ 21 │ 27.49 │ 26.8049 │
╘════════╧═════╧═════════════╧═══════════════════╛

Likelihoods:
╒═════════╤══════════════════╤════════════╤═════════╕
│ Model │ Log-Likelihood │ # Params │ AIC │
╞═════════╪══════════════════╪════════════╪═════════╡
│ A1 │ -360.228 │ 5 │ 730.456 │
│ A2 │ -345.859 │ 8 │ 707.717 │
│ A3 │ -347.415 │ 6 │ 706.831 │
│ fitted │ -347.816 │ 4 │ 703.632 │
│ reduced │ -368.795 │ 2 │ 741.59 │
╘═════════╧══════════════════╧════════════╧═════════╛

Tests of Mean and Variance Fits:
╒════════╤══════════════════════════════╤═════════════╤═════════════╕
│ Name │ -2 * Log(Likelihood Ratio) │ Test d.f. │ P-Value │
╞════════╪══════════════════════════════╪═════════════╪═════════════╡
│ Test 1 │ 45.8734 │ 6 │ 3.13747e-08 │
│ Test 2 │ 28.7388 │ 3 │ 2.54105e-06 │
│ Test 3 │ 3.11388 │ 2 │ 0.21078 │
│ Test 4 │ 0.801365 │ 2 │ 0.669863 │
╘════════╧══════════════════════════════╧═════════════╧═════════════╛
Test 1: Test the null hypothesis that responses and variances don't differ among dose levels
(A2 vs R). If this test fails to reject the null hypothesis (p-value > 0.05), there may not be
a dose-response.

Test 2: Test the null hypothesis that variances are homogenous (A1 vs A2). If this test fails to
reject the null hypothesis (p-value > 0.05), the simpler constant variance model may be appropriate.

Test 3: Test the null hypothesis that the variances are adequately modeled (A3 vs A2). If this test
fails to reject the null hypothesis (p-value > 0.05), it may be inferred that the variances have
been modeled appropriately.

Test 4: Test the null hypothesis that the model for the mean fits the data (Fitted vs A3). If this
test fails to reject the null hypothesis (p-value > 0.1), the user has support for use of the
selected model.

# Litters w/ variations (%), Rt, GD6-20, DuPont (2010)

## Dataset

**Name:** Litters w/ variations (%), Rt, GD6-20, DuPont (2010)

| Dose | N | Mean | Std. Dev. |
| --- | --- | --- | --- |
| 0 | 22 | 11.9 | 14.31 |
| 10 | 21 | 12.8 | 10.92 |
| 100 | 21 | 15.3 | 9.86 |
| 1000 | 21 | 30.6 | 27.22 |

Test 1 Dose Response: <0.0001

Test 2 Homogeneity of Variance: <0.0001

Test 3 Variance Model Selection: 0.1917

## Settings

| Setting | Value |
| --- | --- |
| BMR | 5% Relative Deviation |
| Distribution | Normal + Nonconstant variance |
| Adverse Direction | Up (↑) |
| Maximum Polynomial Degree | 3 |
| Confidence Level (one sided) | 0.95 |

## Maximum Likelihood Approach

| Model | BMDL | BMD | BMDU | *P*-Value | AIC | Scaled Residual at Control | Scaled Residual near BMD | Recommendation and Notes |
| --- | --- | --- | --- | --- | --- | --- | --- | --- |
| Exponential 3 | 41.333 | 670.406 | 894.059 | 0.317 | 703.796 | -0.566 | -0. | **Viable** BMD/BMDL ratio > 3.0 |
| Exponential 5 | 17.756 | 535.347 | 878.857 | - | 705.796 | -0.566 | 0.779 | **Questionable** Zero degrees of freedom; saturated model BMD/BMDL ratio > 3.0 BMD/BMDL ratio > 20.0 |
| Hill | 83.33 | 526.761 | 861.416 | - | 705.796 | -0.566 | 0.779 | **Questionable** Zero degrees of freedom; saturated model BMD/BMDL ratio > 3.0 |
| Polynomial 2 | 23.309 | 195.023 | 309.703 | 0.588 | 701.857 | -0.538 | 0.732 | **Viable** BMD/BMDL ratio > 3.0 |
| Polynomial 3^ab^ | 23.481 | 337.509 | 460.302 | 0.8 | 699.801 | -0.563 | 0.774 | **Recommended - Lowest AIC** BMD/BMDL ratio > 3.0 |
| Power | 23.499 | 830.551 | 880.305 | 0.317 | 703.796 | -0.566 | -0. | **Questionable** BMD/BMDL ratio > 3.0 BMD/BMDL ratio > 20.0 |
| Linear | 20.429 | 35.824 | 89.823 | 0.323 | 703.059 | -0.319 | -0.022 | **Viable** |

^a^ BMDS recommended best fitting model

^b^ User selected best fitting model


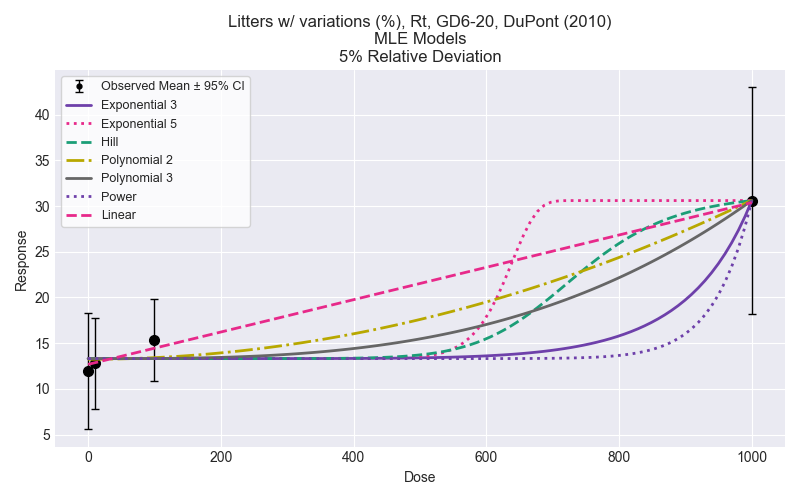


## Selected Model: Polynomial 3


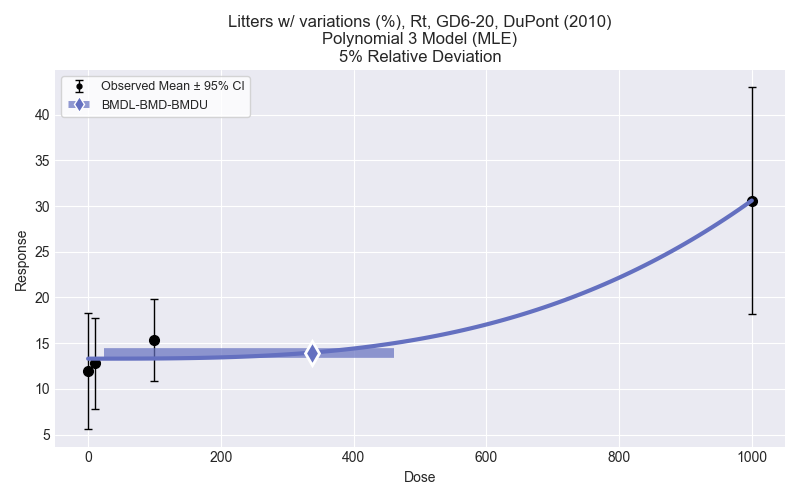


Polynomial 3 Model
══════════════════════════════

Version: pybmds 25.1 (bmdscore 25.1)

Input Summary:
╒══════════════════════════════╤═══════════════════════════════╕
│ BMR │ 5% Relative Deviation │
│ Distribution │ Normal + Nonconstant variance │
│ Modeling Direction │ Up (↑) │
│ Confidence Level (one sided) │ 0.95 │
│ Modeling Approach │ MLE │
│ Degree │ 3 │
╘══════════════════════════════╧═══════════════════════════════╛

Parameter Settings:
╒═════════════╤═══════════╤═══════╤══════════╕
│ Parameter │ Initial │ Min │ Max │
╞═════════════╪═══════════╪═══════╪══════════╡
│ g │ 0 │ 0 │ 1000 │
│ b1 │ 0 │ 0 │ 18 │
│ b2 │ 0 │ 0 │ 1e+06 │
│ b3 │ 0 │ 0 │ 1e+06 │
│ rho │ 0 │ 0 │ 18 │
│ alpha │ 0 │ -18 │ 18 │
╘═════════════╧═══════════╧═══════╧══════════╛

Modeling Summary:
╒════════════════╤═════════════╕
│ BMD │ 337.509 │
│ BMDL │ 23.4814 │
│ BMDU │ 460.302 │
│ AIC │ 699.801 │
│ Log-Likelihood │ -346.901 │
│ P-Value │ 0.800027 │
│ Model d.f. │ 3 │
╘════════════════╧═════════════╛

Model Parameters:
╒════════════╤══════════════╤════════════╤══════════════╕
│ Variable │ Estimate │ On Bound │ Std Error │
╞════════════╪══════════════╪════════════╪══════════════╡
│ g │ 13.3037 │ no │ 1.35613 │
│ b1 │ 0 │ yes │ Not Reported │
│ b2 │ 0 │ yes │ Not Reported │
│ b3 │ 1.73015e-08 │ yes │ Not Reported │
│ rho │ 1.96843 │ no │ 0.41001 │
│ alpha │ 0.838872 │ no │ 0.802151 │
╘════════════╧══════════════╧════════════╧══════════════╛
Standard errors estimates are not generated for parameters estimated on corresponding bounds,
although sampling error is present for all parameters, as a rule. Standard error estimates may not
be reliable as a basis for confidence intervals or tests when one or more parameters are on bounds.


Goodness of Fit:
╒════════╤═════╤═══════════════╤═════════════════════╤═══════════════════╕
│ Dose │ N │ Sample Mean │ Model Fitted Mean │ Scaled Residual │
╞════════╪═════╪═══════════════╪═════════════════════╪═══════════════════╡
│ 0 │ 22 │ 11.9 │ 13.3037 │ -0.562857 │
│ 10 │ 21 │ 12.8 │ 13.3037 │ -0.197331 │
│ 100 │ 21 │ 15.3 │ 13.321 │ 0.774328 │
│ 1000 │ 21 │ 30.6 │ 30.6052 │ -0.000899365 │
╘════════╧═════╧═══════════════╧═════════════════════╧═══════════════════╛
╒════════╤═════╤═════════════╤═══════════════════╕
│ Dose │ N │ Sample SD │ Model Fitted SD │
╞════════╪═════╪═════════════╪═══════════════════╡
│ 0 │ 22 │ 14.31 │ 11.6971 │
│ 10 │ 21 │ 10.92 │ 11.6972 │
│ 100 │ 21 │ 9.86 │ 11.7121 │
│ 1000 │ 21 │ 27.22 │ 26.5579 │
╘════════╧═════╧═════════════╧═══════════════════╛

Likelihoods:
╒═════════╤══════════════════╤════════════╤═════════╕
│ Model │ Log-Likelihood │ # Params │ AIC │
╞═════════╪══════════════════╪════════════╪═════════╡
│ A1 │ -359.455 │ 5 │ 728.911 │
│ A2 │ -344.746 │ 8 │ 705.492 │
│ A3 │ -346.398 │ 6 │ 704.796 │
│ fitted │ -346.901 │ 3 │ 699.801 │
│ reduced │ -367.459 │ 2 │ 738.917 │
╘═════════╧══════════════════╧════════════╧═════════╛

Tests of Mean and Variance Fits:
╒════════╤══════════════════════════════╤═════════════╤═════════════╕
│ Name │ -2 * Log(Likelihood Ratio) │ Test d.f. │ P-Value │
╞════════╪══════════════════════════════╪═════════════╪═════════════╡
│ Test 1 │ 45.4253 │ 6 │ 3.85227e-08 │
│ Test 2 │ 29.4189 │ 3 │ 1.82853e-06 │
│ Test 3 │ 3.30414 │ 2 │ 0.191653 │
│ Test 4 │ 1.00506 │ 3 │ 0.800027 │
╘════════╧══════════════════════════════╧═════════════╧═════════════╛
Test 1: Test the null hypothesis that responses and variances don't differ among dose levels
(A2 vs R). If this test fails to reject the null hypothesis (p-value > 0.05), there may not be
a dose-response.

Test 2: Test the null hypothesis that variances are homogenous (A1 vs A2). If this test fails to
reject the null hypothesis (p-value > 0.05), the simpler constant variance model may be appropriate.

Test 3: Test the null hypothesis that the variances are adequately modeled (A3 vs A2). If this test
fails to reject the null hypothesis (p-value > 0.05), it may be inferred that the variances have
been modeled appropriately.

Test 4: Test the null hypothesis that the model for the mean fits the data (Fitted vs A3). If this
test fails to reject the null hypothesis (p-value > 0.1), the user has support for use of the
selected model.

# Serum cholesterol, Rt, GD8-PND0, Conley et al. (2021)

## Dataset

**Name:** Serum cholesterol, Rt, GD8-PND0, Conley et al. (2021)

| Dose | N | Mean | Std. Dev. |
| --- | --- | --- | --- |
| 0 | 5 | 46 | 7 |
| 10 | 5 | 55 | 13 |
| 30 | 5 | 45 | 7 |
| 62.5 | 4 | 56 | 14 |
| 125 | 5 | 82 | 18 |
| 250 | 5 | 80 | 11 |

Test 1 Dose Response: <0.0001

Test 2 Homogeneity of Variance: 0.2493

Test 3 Variance Model Selection: 0.2493

## Settings

| Setting | Value |
| --- | --- |
| BMR | 1.0 Standard Deviation |
| Distribution | Normal + Constant variance |
| Adverse Direction | Up (↑) |
| Maximum Polynomial Degree | 3 |
| Confidence Level (one sided) | 0.95 |

## Maximum Likelihood Approach

| Model | BMDL | BMD | BMDU | *P*-Value | AIC | Scaled Residual at Control | Scaled Residual near BMD | Recommendation and Notes |
| --- | --- | --- | --- | --- | --- | --- | --- | --- |
| Exponential 3 | 87.694 | 114.411 | 172.497 | 0.009 | 240.256 | -0.738 | 2.651 | **Questionable** \|Residual near BMD\| > 2.0 Goodness of fit p-value < 0.1 Control stdev. fit > 1.5 |
| Exponential 5^ab^ | 51.783 | 64.338 | 117.074 | 0.47 | 231.298 | -0.524 | <0.001 | **Recommended - Lowest AIC** Control stdev. fit > 1.5 |
| Hill | 54.296 | 64.671 | 112.482 | 0.47 | 231.298 | -0.524 | -0. | **Viable** Control stdev. fit > 1.5 |
| Polynomial 2 | 65.254 | 90.257 | 146.23 | 0.018 | 238.731 | -0.492 | -0.33 | **Questionable** Goodness of fit p-value < 0.1 Control stdev. fit > 1.5 |
| Polynomial 3 | 65.25 | 90.089 | 146.231 | 0.018 | 238.731 | -0.488 | -0.329 | **Questionable** Goodness of fit p-value < 0.1 Control stdev. fit > 1.5 |
| Power | 65.22 | 88.727 | 90.564 | 0.018 | 238.746 | -0.495 | -0.333 | **Questionable** Goodness of fit p-value < 0.1 Control stdev. fit > 1.5 |
| Linear | 65.279 | 90.267 | 145.105 | 0.018 | 238.731 | -0.492 | -0.33 | **Questionable** Goodness of fit p-value < 0.1 Control stdev. fit > 1.5 |

^a^ BMDS recommended best fitting model

^b^ User selected best fitting model


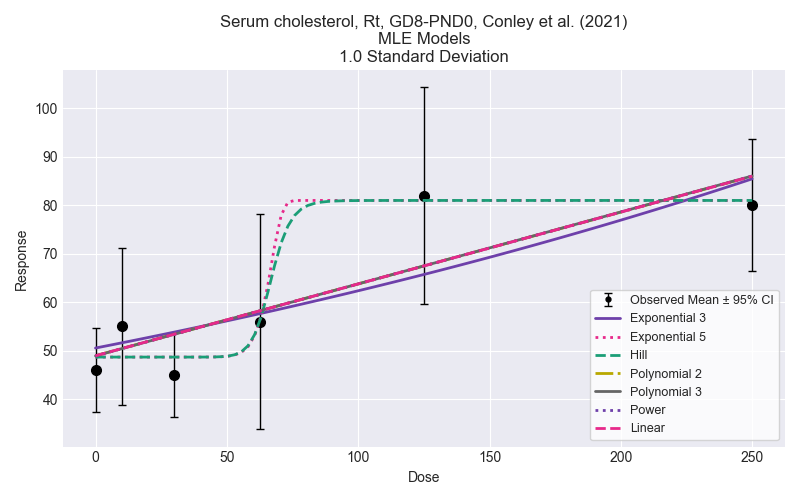


## Selected Model: Exponential 5


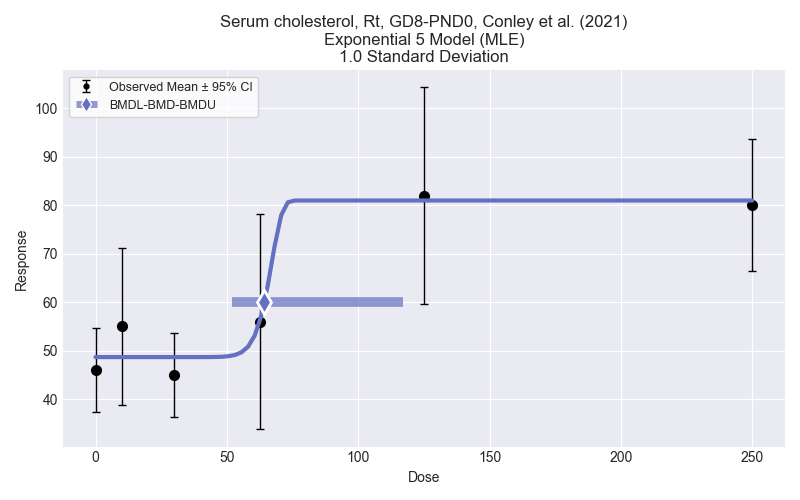


Exponential 5 Model
══════════════════════════════

Version: pybmds 25.1 (bmdscore 25.1)

Input Summary:
╒══════════════════════════════╤════════════════════════════╕
│ BMR │ 1.0 Standard Deviation │
│ Distribution │ Normal + Constant variance │
│ Modeling Direction │ Up (↑) │
│ Confidence Level (one sided) │ 0.95 │
│ Modeling Approach │ MLE │
╘══════════════════════════════╧════════════════════════════╛

Parameter Settings:
╒═════════════╤═══════════╤═══════╤═══════╕
│ Parameter │ Initial │ Min │ Max │
╞═════════════╪═══════════╪═══════╪═══════╡
│ a │ 0 │ 0 │ 100 │
│ b │ 0 │ 0 │ 100 │
│ c │ 0 │ 0 │ 20 │
│ d │ 1 │ 1 │ 18 │
│ log-alpha │ 0 │ -18 │ 18 │
╘═════════════╧═══════════╧═══════╧═══════╛

Modeling Summary:
╒════════════════╤═════════════╕
│ BMD │ 64.3376 │
│ BMDL │ 51.7832 │
│ BMDU │ 117.074 │
│ AIC │ 231.298 │
│ Log-Likelihood │ -111.649 │
│ P-Value │ 0.469744 │
│ Model d.f. │ 3 │
╘════════════════╧═════════════╛

Model Parameters:
╒════════════╤════════════╤════════════╤══════════════╕
│ Variable │ Estimate │ On Bound │ Std Error │
╞════════════╪════════════╪════════════╪══════════════╡
│ a │ 48.6667 │ no │ 2.93583 │
│ b │ 0.0148375 │ no │ 0.000791637 │
│ c │ 1.66438 │ no │ 0.0748976 │
│ d │ 18 │ yes │ Not Reported │
│ log-alpha │ 4.86204 │ no │ 0.262613 │
╘════════════╧════════════╧════════════╧══════════════╛
Standard errors estimates are not generated for parameters estimated on corresponding bounds,
although sampling error is present for all parameters, as a rule. Standard error estimates may not
be reliable as a basis for confidence intervals or tests when one or more parameters are on bounds.


Goodness of Fit:
╒════════╤═════╤═══════════════╤═════════════════════╤═══════════════════╕
│ Dose │ N │ Sample Mean │ Model Fitted Mean │ Scaled Residual │
╞════════╪═════╪═══════════════╪═════════════════════╪═══════════════════╡
│ 0 │ 5 │ 46 │ 48.6667 │ -0.524415 │
│ 10 │ 5 │ 55 │ 48.6667 │ 1.24549 │
│ 30 │ 5 │ 45 │ 48.6667 │ -0.721074 │
│ 62.5 │ 4 │ 56 │ 56 │ 1.86626e-06 │
│ 125 │ 5 │ 82 │ 81 │ 0.196656 │
│ 250 │ 5 │ 80 │ 81 │ -0.196656 │
╘════════╧═════╧═══════════════╧═════════════════════╧═══════════════════╛
╒════════╤═════╤═════════════╤═══════════════════╕
│ Dose │ N │ Sample SD │ Model Fitted SD │
╞════════╪═════╪═════════════╪═══════════════════╡
│ 0 │ 5 │ 7 │ 11.3705 │
│ 10 │ 5 │ 13 │ 11.3705 │
│ 30 │ 5 │ 7 │ 11.3705 │
│ 62.5 │ 4 │ 14 │ 11.3705 │
│ 125 │ 5 │ 18 │ 11.3705 │
│ 250 │ 5 │ 11 │ 11.3705 │
╘════════╧═════╧═════════════╧═══════════════════╛

Likelihoods:
╒═════════╤══════════════════╤════════════╤═════════╕
│ Model │ Log-Likelihood │ # Params │ AIC │
╞═════════╪══════════════════╪════════════╪═════════╡
│ A1 │ -110.383 │ 7 │ 234.767 │
│ A2 │ -107.066 │ 12 │ 238.132 │
│ A3 │ -110.383 │ 7 │ 234.767 │
│ fitted │ -111.649 │ 4 │ 231.298 │
│ reduced │ -126.061 │ 2 │ 256.122 │
╘═════════╧══════════════════╧════════════╧═════════╛

Tests of Mean and Variance Fits:
╒════════╤══════════════════════════════╤═════════════╤════════════╕
│ Name │ -2 * Log(Likelihood Ratio) │ Test d.f. │ P-Value │
╞════════╪══════════════════════════════╪═════════════╪════════════╡
│ Test 1 │ 37.9897 │ 10 │ 3.8108e-05 │
│ Test 2 │ 6.63475 │ 5 │ 0.249252 │
│ Test 3 │ 6.63475 │ 5 │ 0.249252 │
│ Test 4 │ 2.53084 │ 3 │ 0.469744 │
╘════════╧══════════════════════════════╧═════════════╧════════════╛
Test 1: Test the null hypothesis that responses and variances don't differ among dose levels
(A2 vs R). If this test fails to reject the null hypothesis (p-value > 0.05), there may not be
a dose-response.

Test 2: Test the null hypothesis that variances are homogenous (A1 vs A2). If this test fails to
reject the null hypothesis (p-value > 0.05), the simpler constant variance model may be appropriate.

Test 3: Test the null hypothesis that the variances are adequately modeled (A3 vs A2). If this test
fails to reject the null hypothesis (p-value > 0.05), it may be inferred that the variances have
been modeled appropriately.

Test 4: Test the null hypothesis that the model for the mean fits the data (Fitted vs A3). If this
test fails to reject the null hypothesis (p-value > 0.1), the user has support for use of the
selected model.

# Serum triglycerides, Rt, GD8-PND0, Conley et al. (2021)

## Dataset

**Name:** Serum triglycerides, Rt, GD8-PND0, Conley et al. (2021)

| Dose | N | Mean | Std. Dev. |
| --- | --- | --- | --- |
| 0 | 5 | 29 | 4 |
| 10 | 5 | 33 | 7 |
| 30 | 5 | 24 | 4 |
| 62.5 | 4 | 28 | 8 |
| 125 | 5 | 44 | 9 |
| 250 | 5 | 45 | 11 |

Test 1 Dose Response: 0.0002

Test 2 Homogeneity of Variance: 0.1694

Test 3 Variance Model Selection: 0.1694

## Settings

| Setting | Value |
| --- | --- |
| BMR | 1.0 Standard Deviation |
| Distribution | Normal + Constant variance |
| Adverse Direction | Up (↑) |
| Maximum Polynomial Degree | 3 |
| Confidence Level (one sided) | 0.95 |

## Maximum Likelihood Approach

| Model | BMDL | BMD | BMDU | *P*-Value | AIC | Scaled Residual at Control | Scaled Residual near BMD | Recommendation and Notes |
| --- | --- | --- | --- | --- | --- | --- | --- | --- |
| Exponential 3 | 95.806 | 127.945 | 211.499 | 0.022 | 210.574 | 0.11 | 2.005 | **Questionable** \|Residual near BMD\| > 2.0 Goodness of fit p-value < 0.1 Control stdev. fit > 1.5 |
| Exponential 5^ab^ | 91.32 | 114.54 | 123.215 | 0.244 | 205.281 | 0.146 | <0.001 | **Recommended - Lowest AIC** Control stdev. fit > 1.5 |
| Hill | 64.247 | 105.948 | 121.708 | 0.244 | 205.281 | 0.146 | <0.001 | **Viable** Control stdev. fit > 1.5 |
| Polynomial 2 | 75.727 | 111.748 | 200.106 | 0.026 | 210.162 | 0.226 | 1.832 | **Questionable** Goodness of fit p-value < 0.1 Control stdev. fit > 1.5 |
| Polynomial 3 | 75.786 | 108.815 | 199.829 | 0.026 | 210.149 | 0.27 | 1.805 | **Questionable** Goodness of fit p-value < 0.1 Control stdev. fit > 1.5 |
| Power | 75.811 | 110.557 | 203.302 | 0.012 | 212.147 | 0.252 | 1.822 | **Questionable** Goodness of fit p-value < 0.1 Control stdev. fit > 1.5 |
| Linear | 75.783 | 108.937 | 192.236 | 0.026 | 210.149 | 0.268 | 1.806 | **Questionable** Goodness of fit p-value < 0.1 Control stdev. fit > 1.5 |

^a^ BMDS recommended best fitting model

^b^ User selected best fitting model


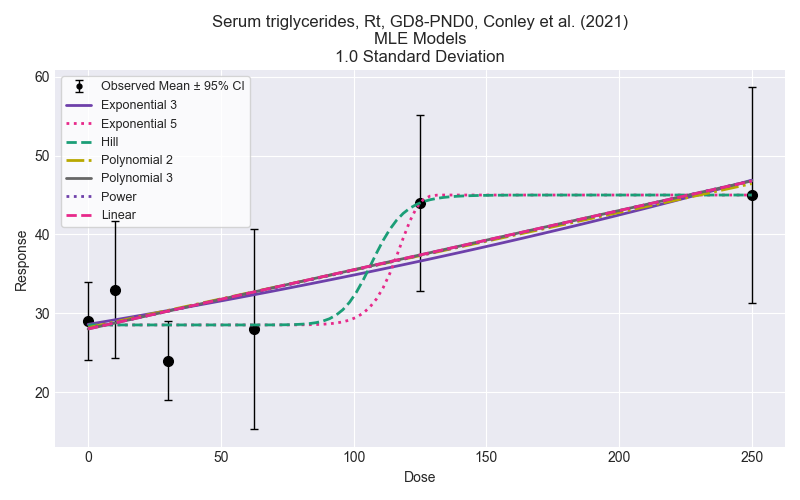


## Selected Model: Exponential 5


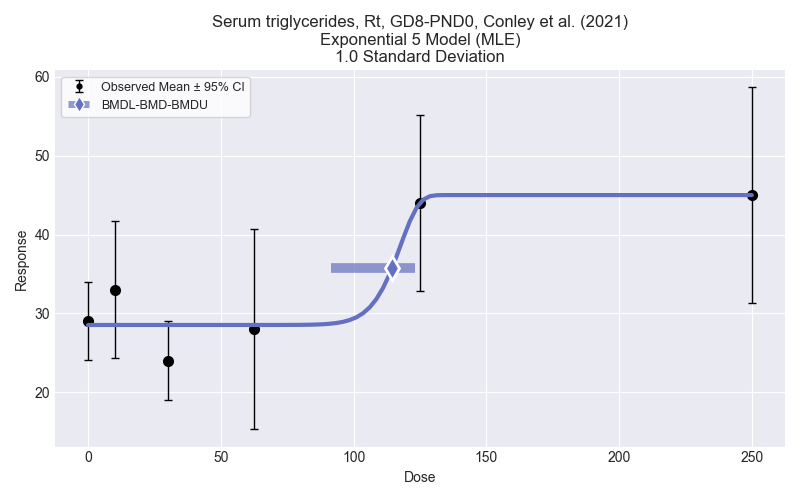


Exponential 5 Model
══════════════════════════════

Version: pybmds 25.1 (bmdscore 25.1)

Input Summary:
╒══════════════════════════════╤════════════════════════════╕
│ BMR │ 1.0 Standard Deviation │
│ Distribution │ Normal + Constant variance │
│ Modeling Direction │ Up (↑) │
│ Confidence Level (one sided) │ 0.95 │
│ Modeling Approach │ MLE │
╘══════════════════════════════╧════════════════════════════╛

Parameter Settings:
╒═════════════╤═══════════╤═══════╤═══════╕
│ Parameter │ Initial │ Min │ Max │
╞═════════════╪═══════════╪═══════╪═══════╡
│ a │ 0 │ 0 │ 100 │
│ b │ 0 │ 0 │ 100 │
│ c │ 0 │ 0 │ 20 │
│ d │ 1 │ 1 │ 18 │
│ log-alpha │ 0 │ -18 │ 18 │
╘═════════════╧═══════════╧═══════╧═══════╛

Modeling Summary:
╒════════════════╤════════════╕
│ BMD │ 114.54 │
│ BMDL │ 91.3201 │
│ BMDU │ 123.215 │
│ AIC │ 205.281 │
│ Log-Likelihood │ -98.6403 │
│ P-Value │ 0.243707 │
│ Model d.f. │ 3 │
╘════════════════╧════════════╛

Model Parameters:
╒════════════╤═════════════╤════════════╤══════════════╕
│ Variable │ Estimate │ On Bound │ Std Error │
╞════════════╪═════════════╪════════════╪══════════════╡
│ a │ 28.5263 │ no │ 1.66567 │
│ b │ 0.00847124 │ no │ 0.000748431 │
│ c │ 1.57749 │ no │ 0.0928198 │
│ d │ 18 │ yes │ Not Reported │
│ log-alpha │ 3.9649 │ no │ 0.262612 │
╘════════════╧═════════════╧════════════╧══════════════╛
Standard errors estimates are not generated for parameters estimated on corresponding bounds,
although sampling error is present for all parameters, as a rule. Standard error estimates may not
be reliable as a basis for confidence intervals or tests when one or more parameters are on bounds.


Goodness of Fit:
╒════════╤═════╤═══════════════╤═════════════════════╤═══════════════════╕
│ Dose │ N │ Sample Mean │ Model Fitted Mean │ Scaled Residual │
╞════════╪═════╪═══════════════╪═════════════════════╪═══════════════════╡
│ 0 │ 5 │ 29 │ 28.5263 │ 0.145895 │
│ 10 │ 5 │ 33 │ 28.5263 │ 1.3778 │
│ 30 │ 5 │ 24 │ 28.5263 │ -1.39399 │
│ 62.5 │ 4 │ 28 │ 28.5265 │ -0.145019 │
│ 125 │ 5 │ 44 │ 44 │ 8.17988e-06 │
│ 250 │ 5 │ 45 │ 45 │ -6.20702e-06 │
╘════════╧═════╧═══════════════╧═════════════════════╧═══════════════════╛
╒════════╤═════╤═════════════╤═══════════════════╕
│ Dose │ N │ Sample SD │ Model Fitted SD │
╞════════╪═════╪═════════════╪═══════════════════╡
│ 0 │ 5 │ 4 │ 7.26052 │
│ 10 │ 5 │ 7 │ 7.26052 │
│ 30 │ 5 │ 4 │ 7.26052 │
│ 62.5 │ 4 │ 8 │ 7.26052 │
│ 125 │ 5 │ 9 │ 7.26052 │
│ 250 │ 5 │ 11 │ 7.26052 │
╘════════╧═════╧═════════════╧═══════════════════╛

Likelihoods:
╒═════════╤══════════════════╤════════════╤═════════╕
│ Model │ Log-Likelihood │ # Params │ AIC │
╞═════════╪══════════════════╪════════════╪═════════╡
│ A1 │ -96.5554 │ 7 │ 207.111 │
│ A2 │ -92.6704 │ 12 │ 209.341 │
│ A3 │ -96.5554 │ 7 │ 207.111 │
│ fitted │ -98.6403 │ 4 │ 205.281 │
│ reduced │ -109.365 │ 2 │ 222.73 │
╘═════════╧══════════════════╧════════════╧═════════╛

Tests of Mean and Variance Fits:
╒════════╤══════════════════════════════╤═════════════╤═════════════╕
│ Name │ -2 * Log(Likelihood Ratio) │ Test d.f. │ P-Value │
╞════════╪══════════════════════════════╪═════════════╪═════════════╡
│ Test 1 │ 33.3892 │ 10 │ 0.000234252 │
│ Test 2 │ 7.76999 │ 5 │ 0.169376 │
│ Test 3 │ 7.76999 │ 5 │ 0.169376 │
│ Test 4 │ 4.16976 │ 3 │ 0.243707 │
╘════════╧══════════════════════════════╧═════════════╧═════════════╛
Test 1: Test the null hypothesis that responses and variances don't differ among dose levels
(A2 vs R). If this test fails to reject the null hypothesis (p-value > 0.05), there may not be
a dose-response.

Test 2: Test the null hypothesis that variances are homogenous (A1 vs A2). If this test fails to
reject the null hypothesis (p-value > 0.05), the simpler constant variance model may be appropriate.

Test 3: Test the null hypothesis that the variances are adequately modeled (A3 vs A2). If this test
fails to reject the null hypothesis (p-value > 0.05), it may be inferred that the variances have
been modeled appropriately.

Test 4: Test the null hypothesis that the model for the mean fits the data (Fitted vs A3). If this
test fails to reject the null hypothesis (p-value > 0.1), the user has support for use of the
selected model.

# Serum glucose, Rt, GD8-PND0, Conley et al. (2021)

## Dataset

**Name:** Serum glucose, Rt, GD8-PND0, Conley et al. (2021)

| Dose | N | Mean | Std. Dev. |
| --- | --- | --- | --- |
| 0 | 5 | 54 | 25 |
| 10 | 5 | 73 | 16 |
| 30 | 5 | 57 | 11 |
| 62.5 | 4 | 36 | 12 |
| 125 | 5 | 30 | 7 |
| 250 | 5 | 29 | 9 |

Test 1 Dose Response: <0.0001

Test 2 Homogeneity of Variance: 0.059

Test 3 Variance Model Selection: 0.3228

## Settings

| Setting | Value |
| --- | --- |
| BMR | 1.0 Standard Deviation |
| Distribution | Normal + Nonconstant variance |
| Adverse Direction | Down (↓) |
| Maximum Polynomial Degree | 3 |
| Confidence Level (one sided) | 0.95 |

## Maximum Likelihood Approach

| Model | BMDL | BMD | BMDU | *P*-Value | AIC | Scaled Residual at Control | Scaled Residual near BMD | Recommendation and Notes |
| --- | --- | --- | --- | --- | --- | --- | --- | --- |
| Exponential 3 | 61.541 | 123.261 | 291.323 | 0.014 | 246.005 | -0.512 | -1.563 | **Questionable** Goodness of fit p-value < 0.1 |
| Exponential 5 | 25.374 | 45.185 | 83.811 | 0.561 | 238.598 | -1.249 | 0.266 | **Viable** |
| Hill^ab^ | 25.778 | 42.26 | 84.691 | 0.582 | 238.525 | -1.256 | 0.326 | **Recommended - Lowest AIC** |
| Polynomial 2 | 106.363 | 175.908 | 253.286 | 0.005 | 248.46 | -0.154 | -1.828 | **Questionable** Goodness of fit p-value < 0.1 |
| Polynomial 3 | 106.366 | 175.146 | 335.5 | 0.005 | 248.46 | -0.16 | -1.827 | **Questionable** Goodness of fit p-value < 0.1 |
| Power | 105.861 | 165.142 | 168.56 | 0.005 | 248.51 | -0.224 | -1.806 | **Questionable** Goodness of fit p-value < 0.1 |
| Linear | 106.366 | 175.154 | 335.504 | 0.005 | 248.46 | -0.16 | -1.827 | **Questionable** Goodness of fit p-value < 0.1 |

^a^ BMDS recommended best fitting model

^b^ User selected best fitting model


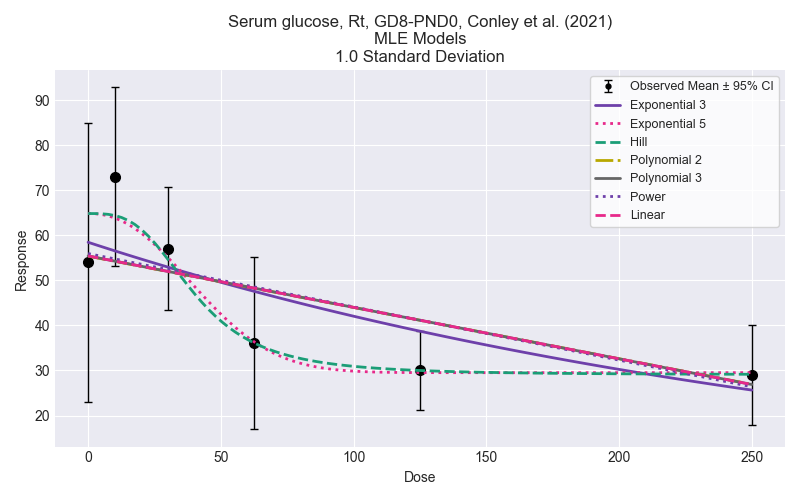


## Selected Model: Hill


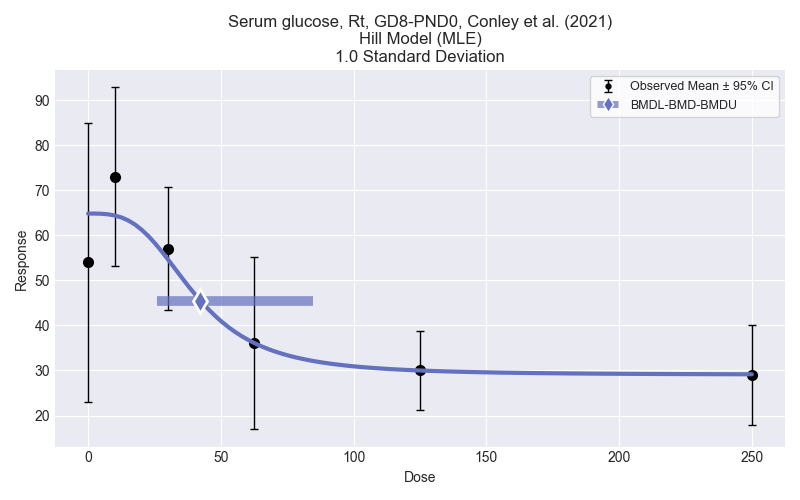


Hill Model
══════════════════════════════

Version: pybmds 25.1 (bmdscore 25.1)

Input Summary:
╒══════════════════════════════╤═══════════════════════════════╕
│ BMR │ 1.0 Standard Deviation │
│ Distribution │ Normal + Nonconstant variance │
│ Modeling Direction │ Down (↓) │
│ Confidence Level (one sided) │ 0.95 │
│ Modeling Approach │ MLE │
╘══════════════════════════════╧═══════════════════════════════╛

Parameter Settings:
╒═════════════╤═══════════╤═══════╤═══════╕
│ Parameter │ Initial │ Min │ Max │
╞═════════════╪═══════════╪═══════╪═══════╡
│ g │ 0 │ -100 │ 100 │
│ v │ 0 │ -100 │ 100 │
│ k │ 0 │ 0 │ 5 │
│ n │ 1 │ 1 │ 18 │
│ rho │ 0 │ -18 │ 18 │
│ alpha │ 0 │ -18 │ 18 │
╘═════════════╧═══════════╧═══════╧═══════╛

Modeling Summary:
╒════════════════╤═════════════╕
│ BMD │ 42.2597 │
│ BMDL │ 25.7778 │
│ BMDU │ 84.6913 │
│ AIC │ 238.525 │
│ Log-Likelihood │ -113.262 │
│ P-Value │ 0.582349 │
│ Model d.f. │ 2 │
╘════════════════╧═════════════╛

Model Parameters:
╒════════════╤═════════════╤════════════╤═════════════╕
│ Variable │ Estimate │ On Bound │ Std Error │
╞════════════╪═════════════╪════════════╪═════════════╡
│ g │ 64.8428 │ no │ 6.11557 │
│ v │ -35.792 │ no │ 6.78355 │
│ k │ 40.2126 │ no │ 9.69564 │
│ n │ 3.17748 │ no │ 1.52385 │
│ rho │ 2.48155 │ no │ 0.815742 │
│ alpha │ 0.0118881 │ no │ 0.000437738 │
╘════════════╧═════════════╧════════════╧═════════════╛

Goodness of Fit:
╒════════╤═════╤═══════════════╤═════════════════════╤═══════════════════╕
│ Dose │ N │ Sample Mean │ Model Fitted Mean │ Scaled Residual │
╞════════╪═════╪═══════════════╪═════════════════════╪═══════════════════╡
│ 0 │ 5 │ 54 │ 64.8428 │ -1.25591 │
│ 10 │ 5 │ 73 │ 64.4179 │ 1.0022 │
│ 30 │ 5 │ 57 │ 54.7232 │ 0.325517 │
│ 62.5 │ 4 │ 36 │ 36.1241 │ -0.0265594 │
│ 125 │ 5 │ 30 │ 29.9993 │ 0.000201698 │
│ 250 │ 5 │ 29 │ 29.1582 │ -0.049384 │
╘════════╧═════╧═══════════════╧═════════════════════╧═══════════════════╛
╒════════╤═════╤═════════════╤═══════════════════╕
│ Dose │ N │ Sample SD │ Model Fitted SD │
╞════════╪═════╪═════════════╪═══════════════════╡
│ 0 │ 5 │ 25 │ 19.3048 │
│ 10 │ 5 │ 16 │ 19.148 │
│ 30 │ 5 │ 11 │ 15.6399 │
│ 62.5 │ 4 │ 12 │ 9.34177 │
│ 125 │ 5 │ 7 │ 7.41852 │
│ 250 │ 5 │ 9 │ 7.1613 │
╘════════╧═════╧═════════════╧═══════════════════╛

Likelihoods:
╒═════════╤══════════════════╤════════════╤═════════╕
│ Model │ Log-Likelihood │ # Params │ AIC │
╞═════════╪══════════════════╪════════════╪═════════╡
│ A1 │ -115.706 │ 7 │ 245.413 │
│ A2 │ -110.387 │ 12 │ 244.773 │
│ A3 │ -112.722 │ 8 │ 241.444 │
│ fitted │ -113.262 │ 6 │ 238.525 │
│ reduced │ -129.263 │ 2 │ 262.526 │
╘═════════╧══════════════════╧════════════╧═════════╛

Tests of Mean and Variance Fits:
╒════════╤══════════════════════════════╤═════════════╤════════════╕
│ Name │ -2 * Log(Likelihood Ratio) │ Test d.f. │ P-Value │
╞════════╪══════════════════════════════╪═════════════╪════════════╡
│ Test 1 │ 37.7525 │ 10 │ 4.1906e-05 │
│ Test 2 │ 10.6392 │ 5 │ 0.0590217 │
│ Test 3 │ 4.67021 │ 4 │ 0.322839 │
│ Test 4 │ 1.08137 │ 2 │ 0.582349 │
╘════════╧══════════════════════════════╧═════════════╧════════════╛
Test 1: Test the null hypothesis that responses and variances don't differ among dose levels
(A2 vs R). If this test fails to reject the null hypothesis (p-value > 0.05), there may not be
a dose-response.

Test 2: Test the null hypothesis that variances are homogenous (A1 vs A2). If this test fails to
reject the null hypothesis (p-value > 0.05), the simpler constant variance model may be appropriate.

Test 3: Test the null hypothesis that the variances are adequately modeled (A3 vs A2). If this test
fails to reject the null hypothesis (p-value > 0.05), it may be inferred that the variances have
been modeled appropriately.

Test 4: Test the null hypothesis that the model for the mean fits the data (Fitted vs A3). If this
test fails to reject the null hypothesis (p-value > 0.1), the user has support for use of the
selected model.

# Pup birthweight GD8-PND2, Rt, F, Conley et al. (2021)

## Dataset

**Name:** Pup birthweight, Rt, F, Conley et al. (2021)

| Dose (mg/kg-day) | N | Mean | Std. Dev. |
| --- | --- | --- | --- |
| 0 | 5 | 6.38 | 0.4 |
| 10 | 5 | 6.33 | 0.2 |
| 30 | 5 | 5.81 | 0.27 |
| 62.5 | 5 | 5.63 | 0.13 |
| 125 | 5 | 5.41 | 0.25 |
| 250 | 5 | 4.71 | 0.42 |

Test 1 Dose Response: <0.0001

Test 2 Homogeneity of Variance: 0.1347

Test 3 Variance Model Selection: 0.1347

## Settings

| Setting | Value |
| --- | --- |
| BMR | 5% Relative Deviation |
| Distribution | Normal + Constant variance |
| Adverse Direction | Down (↓) |
| Maximum Polynomial Degree | 3 |
| Confidence Level (one sided) | 0.95 |

## Maximum Likelihood Approach

| Model | BMDL | BMD | BMDU | *P*-Value | AIC | Scaled Residual at Control | Scaled Residual near BMD | Recommendation and Notes |
| --- | --- | --- | --- | --- | --- | --- | --- | --- |
| Exponential 3 | 36.725 | 43.427 | 58.365 | 0.103 | 19.283 | 1.017 | -1.595 | **Viable** |
| Exponential 5 | 17.349 | 27.471 | 45.99 | 0.16 | 18.739 | 0.285 | -1.464 | **Viable** |
| Hill^ab^ | 13.512 | 24.223 | 43.581 | 0.197 | 18.252 | 0.098 | -1.351 | **Recommended - Lowest AIC** |
| Polynomial 2 | 42.493 | 49.271 | 63.757 | 0.062 | 20.535 | 1.207 | -1.374 | **Questionable** Goodness of fit p-value < 0.1 |
| Polynomial 3 | 42.497 | 49.315 | 63.758 | 0.062 | 20.535 | 1.213 | -1.375 | **Questionable** Goodness of fit p-value < 0.1 |
| Power | 42.491 | 49.245 | 64.434 | 0.062 | 20.535 | 1.206 | -1.374 | **Questionable** Goodness of fit p-value < 0.1 |
| Linear | 42.491 | 49.245 | 58.787 | 0.062 | 20.535 | 1.206 | -1.374 | **Questionable** Goodness of fit p-value < 0.1 |

^a^ BMDS recommended best fitting model

^b^ User selected best fitting model


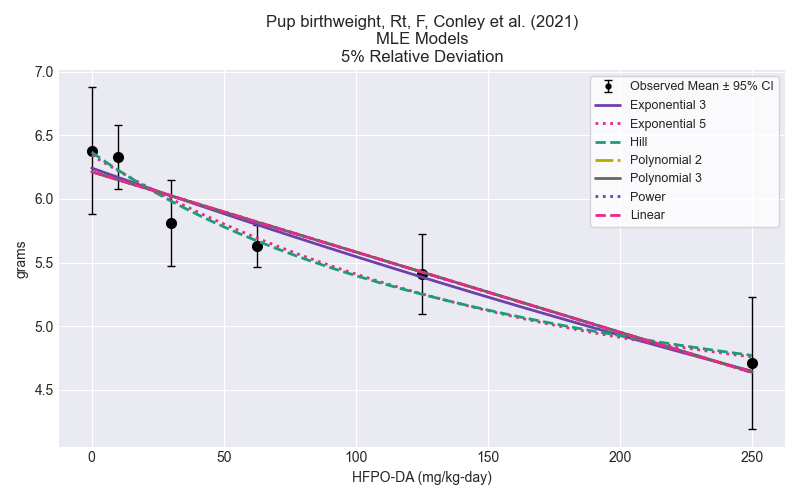


## Selected Model: Hill


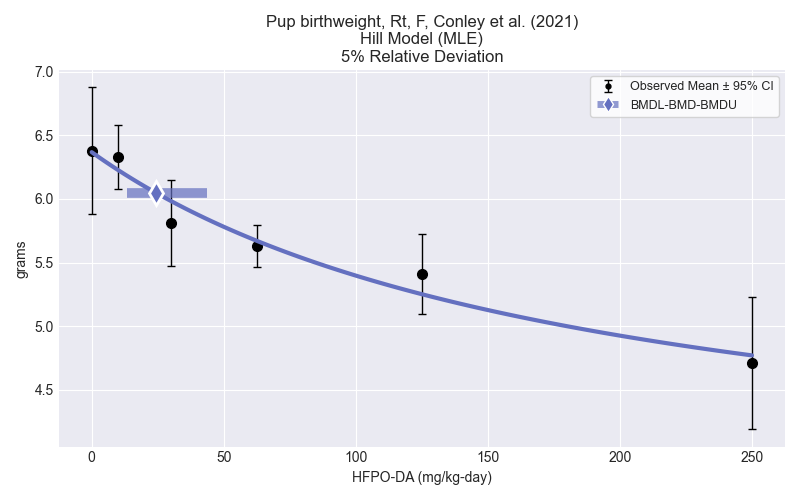


Hill Model
══════════════════════════════

Version: pybmds 25.1 (bmdscore 25.1)

Input Summary:
╒══════════════════════════════╤════════════════════════════╕
│ BMR │ 5% Relative Deviation │
│ Distribution │ Normal + Constant variance │
│ Modeling Direction │ Down (↓) │
│ Confidence Level (one sided) │ 0.95 │
│ Modeling Approach │ MLE │
╘══════════════════════════════╧════════════════════════════╛

Parameter Settings:
╒═════════════╤═══════════╤═══════╤═══════╕
│ Parameter │ Initial │ Min │ Max │
╞═════════════╪═══════════╪═══════╪═══════╡
│ g │ 0 │ -100 │ 100 │
│ v │ 0 │ -100 │ 100 │
│ k │ 0 │ 0 │ 5 │
│ n │ 1 │ 1 │ 18 │
│ alpha │ 0 │ -18 │ 18 │
╘═════════════╧═══════════╧═══════╧═══════╛

Modeling Summary:
╒════════════════╤═══════════╕
│ BMD │ 24.2225 │
│ BMDL │ 13.5124 │
│ BMDU │ 43.5813 │
│ AIC │ 18.2518 │
│ Log-Likelihood │ -5.12591 │
│ P-Value │ 0.196567 │
│ Model d.f. │ 3 │
╘════════════════╧═══════════╛

Model Parameters:
╒════════════╤═════════════╤════════════╤══════════════╕
│ Variable │ Estimate │ On Bound │ Std Error │
╞════════════╪═════════════╪════════════╪══════════════╡
│ g │ 6.36736 │ no │ 0.107416 │
│ v │ -2.80554 │ no │ 0.8777 │
│ k │ 189.233 │ no │ 128.763 │
│ n │ 1 │ yes │ Not Reported │
│ alpha │ 0.0824017 │ no │ 0.00175318 │
╘════════════╧═════════════╧════════════╧══════════════╛
Standard errors estimates are not generated for parameters estimated on corresponding bounds,
although sampling error is present for all parameters, as a rule. Standard error estimates may not
be reliable as a basis for confidence intervals or tests when one or more parameters are on bounds.


Goodness of Fit:
╒════════╤═════╤═══════════════╤═════════════════════╤═══════════════════╕
│ Dose │ N │ Sample Mean │ Model Fitted Mean │ Scaled Residual │
╞════════╪═════╪═══════════════╪═════════════════════╪═══════════════════╡
│ 0 │ 5 │ 6.38 │ 6.36736 │ 0.0984658 │
│ 10 │ 5 │ 6.33 │ 6.22654 │ 0.805899 │
│ 30 │ 5 │ 5.81 │ 5.98345 │ -1.35109 │
│ 62.5 │ 5 │ 5.63 │ 5.6708 │ -0.317834 │
│ 125 │ 5 │ 5.41 │ 5.25133 │ 1.23597 │
│ 250 │ 5 │ 4.71 │ 4.77052 │ -0.471413 │
╘════════╧═════╧═══════════════╧═════════════════════╧═══════════════════╛
╒════════╤═════╤═════════════╤═══════════════════╕
│ Dose │ N │ Sample SD │ Model Fitted SD │
╞════════╪═════╪═════════════╪═══════════════════╡
│ 0 │ 5 │ 0.4 │ 0.287057 │
│ 10 │ 5 │ 0.2 │ 0.287057 │
│ 30 │ 5 │ 0.27 │ 0.287057 │
│ 62.5 │ 5 │ 0.13 │ 0.287057 │
│ 125 │ 5 │ 0.25 │ 0.287057 │
│ 250 │ 5 │ 0.42 │ 0.287057 │
╘════════╧═════╧═════════════╧═══════════════════╛

Likelihoods:
╒═════════╤══════════════════╤════════════╤═════════╕
│ Model │ Log-Likelihood │ # Params │ AIC │
╞═════════╪══════════════════╪════════════╪═════════╡
│ A1 │ -2.7846 │ 7 │ 19.5692 │
│ A2 │ 1.42439 │ 12 │ 21.1512 │
│ A3 │ -2.7846 │ 7 │ 19.5692 │
│ fitted │ -5.12591 │ 4 │ 18.2518 │
│ reduced │ -28.5885 │ 2 │ 61.177 │
╘═════════╧══════════════════╧════════════╧═════════╛

Tests of Mean and Variance Fits:
╒════════╤══════════════════════════════╤═════════════╤═════════════╕
│ Name │ -2 * Log(Likelihood Ratio) │ Test d.f. │ P-Value │
╞════════╪══════════════════════════════╪═════════════╪═════════════╡
│ Test 1 │ 60.0257 │ 10 │ 3.58389e-09 │
│ Test 2 │ 8.41797 │ 5 │ 0.134655 │
│ Test 3 │ 8.41797 │ 5 │ 0.134655 │
│ Test 4 │ 4.68263 │ 3 │ 0.196567 │
╘════════╧══════════════════════════════╧═════════════╧═════════════╛
Test 1: Test the null hypothesis that responses and variances don't differ among dose levels
(A2 vs R). If this test fails to reject the null hypothesis (p-value > 0.05), there may not be
a dose-response.

Test 2: Test the null hypothesis that variances are homogenous (A1 vs A2). If this test fails to
reject the null hypothesis (p-value > 0.05), the simpler constant variance model may be appropriate.

Test 3: Test the null hypothesis that the variances are adequately modeled (A3 vs A2). If this test
fails to reject the null hypothesis (p-value > 0.05), it may be inferred that the variances have
been modeled appropriately.

Test 4: Test the null hypothesis that the model for the mean fits the data (Fitted vs A3). If this
test fails to reject the null hypothesis (p-value > 0.1), the user has support for use of the
selected model.

# Serum glucose, Rt, GD8-PND2, Conley et al. (2021)_drop

## Dataset

**Name:** Serum glucose, Rt, GD8-PND2, Conley et al. (2021)_drop

| Dose | N | Mean | Std. Dev. |
| --- | --- | --- | --- |
| 0 | 5 | 83 | 27 |
| 10 | 5 | 91 | 7 |
| 30 | 5 | 85 | 13 |
| 62.5 | 4 | 79 | 20 |
| 125 | 3.5 | 57 | 28.1 |

Test 1 Dose Response: 0.0355

Test 2 Homogeneity of Variance: 0.0535

Test 3 Variance Model Selection: 0.3152

## Settings

| Setting | Value |
| --- | --- |
| BMR | 1.0 Standard Deviation |
| Distribution | Normal + Nonconstant variance |
| Adverse Direction | Down (↓) |
| Maximum Polynomial Degree | 3 |
| Confidence Level (one sided) | 0.95 |

## Maximum Likelihood Approach

| Model | BMDL | BMD | BMDU | *P*-Value | AIC | Scaled Residual at Control | Scaled Residual near BMD | Recommendation and Notes |
| --- | --- | --- | --- | --- | --- | --- | --- | --- |
| Exponential 3 | 44.878 | 94.667 | 185.752 | 0.097 | 201.416 | -0.485 | 0.007 | **Questionable** Goodness of fit p-value < 0.1 |
| Exponential 5 | 42.633 | 90.438 | 185.33 | 0.043 | 203.41 | -0.473 | 0.009 | **Questionable** Goodness of fit p-value < 0.1 |
| Hill | 37.319 | 69.615 | 125.779 | 0.02 | 204.486 | -0.458 | -0.011 | **Questionable** Goodness of fit p-value < 0.1 |
| Polynomial 2^ab^ | 49.18 | 96.333 | 181.306 | 0.176 | 199.423 | -0.488 | 0.009 | **Recommended - Lowest AIC** |
| Polynomial 3 | 49.18 | 96.333 | 181.306 | 0.176 | 199.423 | -0.488 | 0.009 | **Viable** |
| Power | 49.18 | 96.366 | 181.303 | 0.097 | 201.423 | -0.488 | 0.009 | **Questionable** Goodness of fit p-value < 0.1 |
| Linear | 46.047 | 77.451 | 241.28 | 0.131 | 200.204 | -0.826 | 0.433 | **Viable** |

^a^ BMDS recommended best fitting model

^b^ User selected best fitting model


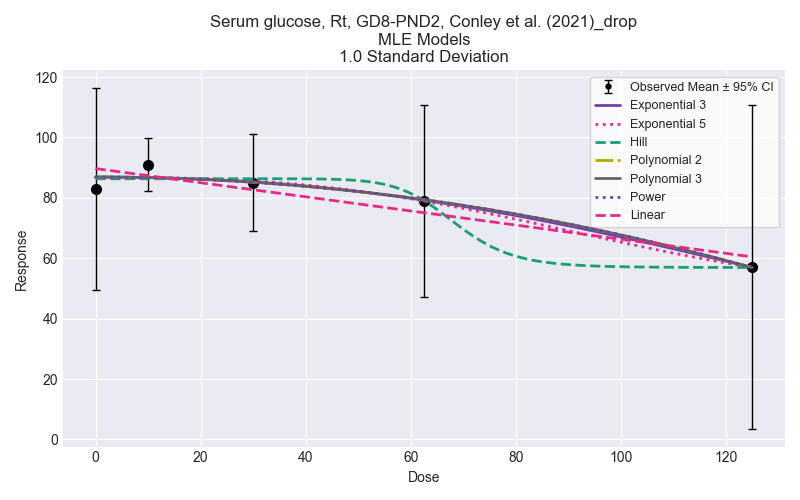


## Selected Model: Polynomial 2


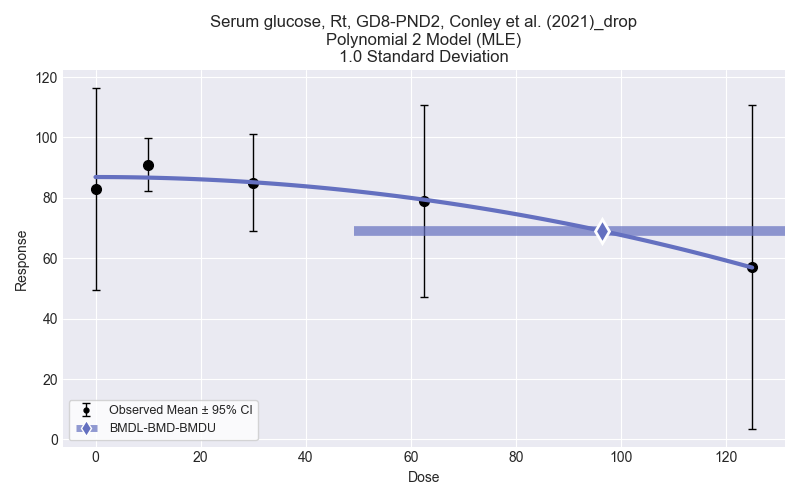


Polynomial 2 Model
══════════════════════════════

Version: pybmds 25.1 (bmdscore 25.1)

Input Summary:
╒══════════════════════════════╤═══════════════════════════════╕
│ BMR │ 1.0 Standard Deviation │
│ Distribution │ Normal + Nonconstant variance │
│ Modeling Direction │ Down (↓) │
│ Confidence Level (one sided) │ 0.95 │
│ Modeling Approach │ MLE │
│ Degree │ 2 │
╘══════════════════════════════╧═══════════════════════════════╛

Parameter Settings:
╒═════════════╤═══════════╤═════════╤═══════╕
│ Parameter │ Initial │ Min │ Max │
╞═════════════╪═══════════╪═════════╪═══════╡
│ g │ 0 │ 0 │ 1000 │
│ b1 │ 0 │ -18 │ 0 │
│ b2 │ 0 │ -1e+06 │ 0 │
│ rho │ 0 │ 0 │ 18 │
│ alpha │ 0 │ -18 │ 18 │
╘═════════════╧═══════════╧═════════╧═══════╛

Modeling Summary:
╒════════════════╤═══════════╕
│ BMD │ 96.3333 │
│ BMDL │ 49.1799 │
│ BMDU │ 181.306 │
│ AIC │ 199.423 │
│ Log-Likelihood │ -96.7114 │
│ P-Value │ 0.17649 │
│ Model d.f. │ 4 │
╘════════════════╧═══════════╛

Model Parameters:
╒════════════╤══════════════╤════════════╤══════════════╕
│ Variable │ Estimate │ On Bound │ Std Error │
╞════════════╪══════════════╪════════════╪══════════════╡
│ g │ 86.8879 │ no │ 4.40511 │
│ b1 │ 0 │ yes │ Not Reported │
│ b2 │ -0.00191836 │ no │ 0.000689023 │
│ rho │ 0 │ yes │ Not Reported │
│ alpha │ 316.933 │ no │ 29947.4 │
╘════════════╧══════════════╧════════════╧══════════════╛
Standard errors estimates are not generated for parameters estimated on corresponding bounds,
although sampling error is present for all parameters, as a rule. Standard error estimates may not
be reliable as a basis for confidence intervals or tests when one or more parameters are on bounds.


Goodness of Fit:
╒════════╤═════╤═══════════════╤═════════════════════╤═══════════════════╕
│ Dose │ N │ Sample Mean │ Model Fitted Mean │ Scaled Residual │
╞════════╪═════╪═══════════════╪═════════════════════╪═══════════════════╡
│ 0 │ 5 │ 83 │ 86.8879 │ -0.488327 │
│ 10 │ 5 │ 91 │ 86.696 │ 0.540594 │
│ 30 │ 5 │ 85 │ 85.1613 │ -0.0202631 │
│ 62.5 │ 4 │ 79 │ 79.3942 │ -0.044291 │
│ 125 │ 3.5 │ 57 │ 56.9134 │ 0.00909695 │
╘════════╧═════╧═══════════════╧═════════════════════╧═══════════════════╛
╒════════╤═════╤═════════════╤═══════════════════╕
│ Dose │ N │ Sample SD │ Model Fitted SD │
╞════════╪═════╪═════════════╪═══════════════════╡
│ 0 │ 5 │ 27 │ 17.8026 │
│ 10 │ 5 │ 7 │ 17.8026 │
│ 30 │ 5 │ 13 │ 17.8026 │
│ 62.5 │ 4 │ 20 │ 17.8026 │
│ 125 │ 3.5 │ 28.1 │ 17.8026 │
╘════════╧═════╧═════════════╧═══════════════════╛

Likelihoods:
╒═════════╤══════════════════╤════════════╤═════════╕
│ Model │ Log-Likelihood │ # Params │ AIC │
╞═════════╪══════════════════╪════════════╪═════════╡
│ A1 │ -96.4416 │ 6 │ 204.883 │
│ A2 │ -91.78 │ 10 │ 203.56 │
│ A3 │ -93.5514 │ 7 │ 201.103 │
│ fitted │ -96.7114 │ 3 │ 199.423 │
│ reduced │ -100.042 │ 2 │ 204.083 │
╘═════════╧══════════════════╧════════════╧═════════╛

Tests of Mean and Variance Fits:
╒════════╤══════════════════════════════╤═════════════╤═══════════╕
│ Name │ -2 * Log(Likelihood Ratio) │ Test d.f. │ P-Value │
╞════════╪══════════════════════════════╪═════════════╪═══════════╡
│ Test 1 │ 16.5234 │ 8 │ 0.0354731 │
│ Test 2 │ 9.32331 │ 4 │ 0.0535073 │
│ Test 3 │ 3.54287 │ 3 │ 0.315245 │
│ Test 4 │ 6.32002 │ 4 │ 0.17649 │
╘════════╧══════════════════════════════╧═════════════╧═══════════╛
Test 1: Test the null hypothesis that responses and variances don't differ among dose levels
(A2 vs R). If this test fails to reject the null hypothesis (p-value > 0.05), there may not be
a dose-response.

Test 2: Test the null hypothesis that variances are homogenous (A1 vs A2). If this test fails to
reject the null hypothesis (p-value > 0.05), the simpler constant variance model may be appropriate.

Test 3: Test the null hypothesis that the variances are adequately modeled (A3 vs A2). If this test
fails to reject the null hypothesis (p-value > 0.05), it may be inferred that the variances have
been modeled appropriately.

Test 4: Test the null hypothesis that the model for the mean fits the data (Fitted vs A3). If this
test fails to reject the null hypothesis (p-value > 0.1), the user has support for use of the
selected model.

# Rel liver wt, Rt, GD8-PND2, Conley et al. (2021)_drop

## Dataset

**Name:** Rel liver wt, Rt, GD8-PND2, Conley et al. (2021)_drop

| Dose | N | Mean | Std. Dev. |
| --- | --- | --- | --- |
| 0 | 5 | 39.2 | 3.6 |
| 10 | 5 | 48.8 | 2.7 |
| 30 | 5 | 55.9 | 5.1 |
| 62.5 | 4 | 60.8 | 3.6 |
| 125 | 4 | 67.8 | 5.6 |

Test 1 Dose Response: <0.0001

Test 2 Homogeneity of Variance: 0.5586

Test 3 Variance Model Selection: 0.5586

## Settings

| Setting | Value |
| --- | --- |
| BMR | 1.0 Standard Deviation |
| Distribution | Normal + Constant variance |
| Adverse Direction | Up (↑) |
| Maximum Polynomial Degree | 3 |
| Confidence Level (one sided) | 0.95 |

## Maximum Likelihood Approach

| Model | BMDL | BMD | BMDU | *P*-Value | AIC | Scaled Residual at Control | Scaled Residual near BMD | Recommendation and Notes |
| --- | --- | --- | --- | --- | --- | --- | --- | --- |
| Exponential 3 | 26.92 | 34.819 | 49.35 | <0.001 | 151.917 | -2.69 | 1.868 | **Questionable** Residual at control > 2.0 Goodness of fit p-value < 0.1 Control stdev. fit > 1.5 |
| Exponential 5 | 3.551 | 5.573 | 10.022 | 0.226 | 136.658 | -0.627 | 1.007 | **Viable** |
| Hill^ab^ | 2.384 | 4.047 | 8.226 | 0.522 | 134.983 | -0.259 | -0.259 | **Recommended - Lowest AIC** lowest dose/BMDL ratio > 3.0 |
| Polynomial 2 | 20.105 | 26.623 | 38.97 | <0.001 | 149.203 | -2.467 | 1.875 | **Questionable** Residual at control > 2.0 Goodness of fit p-value < 0.1 Control stdev. fit > 1.5 |
| Polynomial 3 | 19.448 | 30.495 | 31.126 | <0.001 | 151.969 | -2.796 | 1.748 | **Questionable** Residual at control > 2.0 Goodness of fit p-value < 0.1 Control stdev. fit > 1.5 |
| Power | 20.104 | 26.656 | 38.986 | <0.001 | 149.202 | -2.472 | 1.874 | **Questionable** Residual at control > 2.0 Goodness of fit p-value < 0.1 Control stdev. fit > 1.5 |
| Linear | 20.104 | 26.656 | 38.897 | <0.001 | 149.202 | -2.472 | 1.874 | **Questionable** Residual at control > 2.0 Goodness of fit p-value < 0.1 Control stdev. fit > 1.5 |

^a^ BMDS recommended best fitting model

^b^ User selected best fitting model; note: BMR of 5% resulted in warnings of BMD and BMDL >3-fold below lowest dose


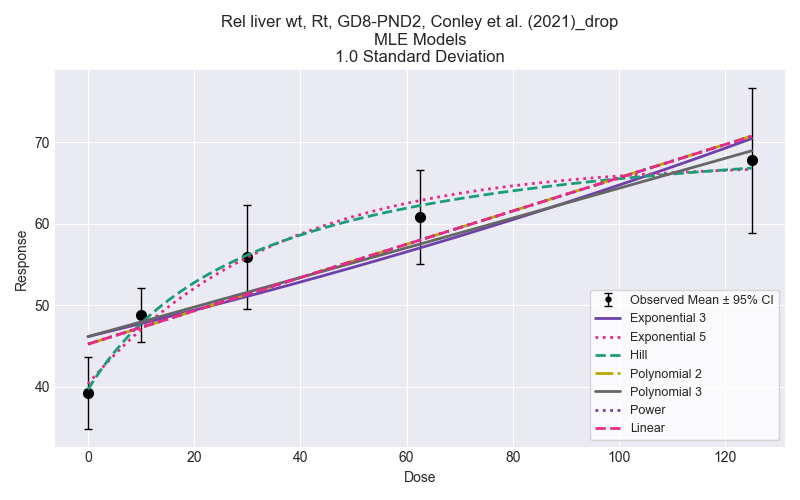


## Selected Model: Hill


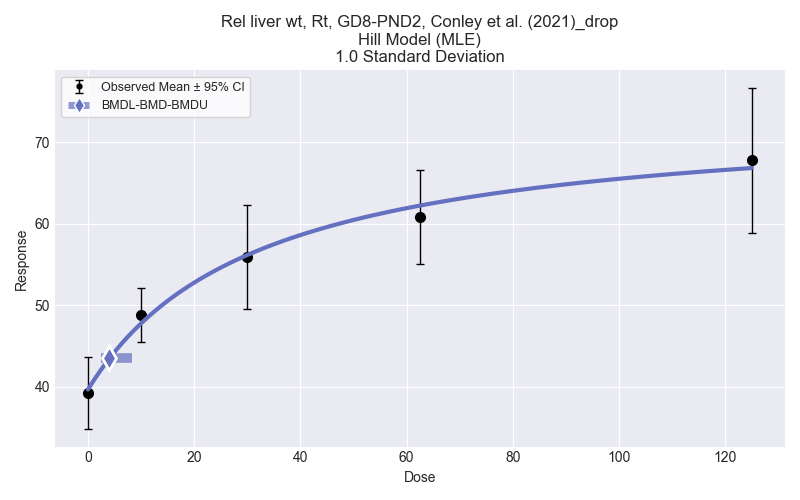


Hill Model
══════════════════════════════

Version: pybmds 25.1 (bmdscore 25.1)

Input Summary:
╒══════════════════════════════╤════════════════════════════╕
│ BMR │ 1.0 Standard Deviation │
│ Distribution │ Normal + Constant variance │
│ Modeling Direction │ Up (↑) │
│ Confidence Level (one sided) │ 0.95 │
│ Modeling Approach │ MLE │
╘══════════════════════════════╧════════════════════════════╛

Parameter Settings:
╒═════════════╤═══════════╤═══════╤═══════╕
│ Parameter │ Initial │ Min │ Max │
╞═════════════╪═══════════╪═══════╪═══════╡
│ g │ 0 │ -100 │ 100 │
│ v │ 0 │ -100 │ 100 │
│ k │ 0 │ 0 │ 5 │
│ n │ 1 │ 1 │ 18 │
│ alpha │ 0 │ -18 │ 18 │
╘═════════════╧═══════════╧═══════╧═══════╛

Modeling Summary:
╒════════════════╤════════════╕
│ BMD │ 4.04718 │
│ BMDL │ 2.38365 │
│ BMDU │ 8.22612 │
│ AIC │ 134.983 │
│ Log-Likelihood │ -63.4916 │
│ P-Value │ 0.521715 │
│ Model d.f. │ 2 │
╘════════════════╧════════════╛

Model Parameters:
╒════════════╤════════════╤════════════╤══════════════╕
│ Variable │ Estimate │ On Bound │ Std Error │
╞════════════╪════════════╪════════════╪══════════════╡
│ g │ 39.6424 │ no │ 1.68886 │
│ v │ 34.1665 │ no │ 4.07968 │
│ k │ 32.1036 │ no │ 12.6219 │
│ n │ 1 │ yes │ Not Reported │
│ alpha │ 14.6309 │ no │ 63.1236 │
╘════════════╧════════════╧════════════╧══════════════╛
Standard errors estimates are not generated for parameters estimated on corresponding bounds,
although sampling error is present for all parameters, as a rule. Standard error estimates may not
be reliable as a basis for confidence intervals or tests when one or more parameters are on bounds.


Goodness of Fit:
╒════════╤═════╤═══════════════╤═════════════════════╤═══════════════════╕
│ Dose │ N │ Sample Mean │ Model Fitted Mean │ Scaled Residual │
╞════════╪═════╪═══════════════╪═════════════════════╪═══════════════════╡
│ 0 │ 5 │ 39.2 │ 39.6424 │ -0.258627 │
│ 10 │ 5 │ 48.8 │ 47.7573 │ 0.609564 │
│ 30 │ 5 │ 55.9 │ 56.147 │ -0.144398 │
│ 62.5 │ 4 │ 60.8 │ 62.2145 │ -0.739627 │
│ 125 │ 4 │ 67.8 │ 66.8271 │ 0.508709 │
╘════════╧═════╧═══════════════╧═════════════════════╧═══════════════════╛
╒════════╤═════╤═════════════╤═══════════════════╕
│ Dose │ N │ Sample SD │ Model Fitted SD │
╞════════╪═════╪═════════════╪═══════════════════╡
│ 0 │ 5 │ 3.6 │ 3.82503 │
│ 10 │ 5 │ 2.7 │ 3.82503 │
│ 30 │ 5 │ 5.1 │ 3.82503 │
│ 62.5 │ 4 │ 3.6 │ 3.82503 │
│ 125 │ 4 │ 5.6 │ 3.82503 │
╘════════╧═════╧═════════════╧═══════════════════╛

Likelihoods:
╒═════════╤══════════════════╤════════════╤═════════╕
│ Model │ Log-Likelihood │ # Params │ AIC │
╞═════════╪══════════════════╪════════════╪═════════╡
│ A1 │ -62.841 │ 6 │ 137.682 │
│ A2 │ -61.3432 │ 10 │ 142.686 │
│ A3 │ -62.841 │ 6 │ 137.682 │
│ fitted │ -63.4916 │ 4 │ 134.983 │
│ reduced │ -86.6013 │ 2 │ 177.203 │
╘═════════╧══════════════════╧════════════╧═════════╛

Tests of Mean and Variance Fits:
╒════════╤══════════════════════════════╤═════════════╤═════════════╕
│ Name │ -2 * Log(Likelihood Ratio) │ Test d.f. │ P-Value │
╞════════╪══════════════════════════════╪═════════════╪═════════════╡
│ Test 1 │ 50.5162 │ 8 │ 3.25182e-08 │
│ Test 2 │ 2.99556 │ 4 │ 0.558569 │
│ Test 3 │ 2.99556 │ 4 │ 0.558569 │
│ Test 4 │ 1.30127 │ 2 │ 0.521715 │
╘════════╧══════════════════════════════╧═════════════╧═════════════╛
Test 1: Test the null hypothesis that responses and variances don't differ among dose levels
(A2 vs R). If this test fails to reject the null hypothesis (p-value > 0.05), there may not be
a dose-response.

Test 2: Test the null hypothesis that variances are homogenous (A1 vs A2). If this test fails to
reject the null hypothesis (p-value > 0.05), the simpler constant variance model may be appropriate.

Test 3: Test the null hypothesis that the variances are adequately modeled (A3 vs A2). If this test
fails to reject the null hypothesis (p-value > 0.05), it may be inferred that the variances have
been modeled appropriately.

Test 4: Test the null hypothesis that the model for the mean fits the data (Fitted vs A3). If this
test fails to reject the null hypothesis (p-value > 0.1), the user has support for use of the
selected model.

# Placenta wt (CD), Rt, F, GD0.5-19.5, Lv et al. (2024)

## Dataset

**Name:** Placenta wt (CD), Rt, F, GD0.5-19.5, Lv et al. (2024)

| Dose | N | Mean | Std. Dev. |
| --- | --- | --- | --- |
| 0 | 6 | 0.35 | 0.07 |
| 1 | 6 | 0.34 | 0.03 |
| 10 | 6 | 0.39 | 0.04 |
| 100 | 6 | 0.46 | 0.04 |

## Settings

| Setting | Value |
| --- | --- |
| BMR | 5% Relative Deviation |
| Distribution | Normal + Constant variance |
| Adverse Direction | Up (↑) |
| Maximum Polynomial Degree | 3 |
| Confidence Level (one sided) | 0.95 |

## Maximum Likelihood Approach

| Model | BMDL | BMD | BMDU | *P*-Value | AIC | Scaled Residual at Control | Scaled Residual near BMD | Recommendation and Notes |
| --- | --- | --- | --- | --- | --- | --- | --- | --- |
| Exponential 3 | - | 6.419 | - | - | - | -2.043 | -139.476 | **Unusable** Did not successfully execute. |
| Exponential 5 | 1.302 | 8.722 | 19.436 | - | -72.431 | 0.282 | <0.001 | **Questionable** Zero degrees of freedom; saturated model BMD/BMDL ratio > 3.0 |
| Hill | 6.841 | 8.234 | 9.927 | - | -72.431 | 0.282 | <0.001 | **Questionable** Zero degrees of freedom; saturated model |
| Polynomial 2 | 11.877 | 16.612 | 49.837 | 0.265 | -73.935 | -0.285 | 1.284 | **Viable** |
| Polynomial 3 | 11.876 | 16.635 | 62.652 | 0.265 | -73.935 | -0.287 | 1.282 | **Viable** |
| Power | 11.877 | 16.62 | 91.005 | 0.265 | -73.935 | -0.286 | 1.283 | **Viable** |
| Linear^ab^ | 11.877 | 16.62 | 26.489 | 0.265 | -73.935 | -0.286 | 1.283 | **Recommended - Lowest AIC** |

^a^ BMDS recommended best fitting model

^b^ User selected best fitting model

## Selected Model: Linear


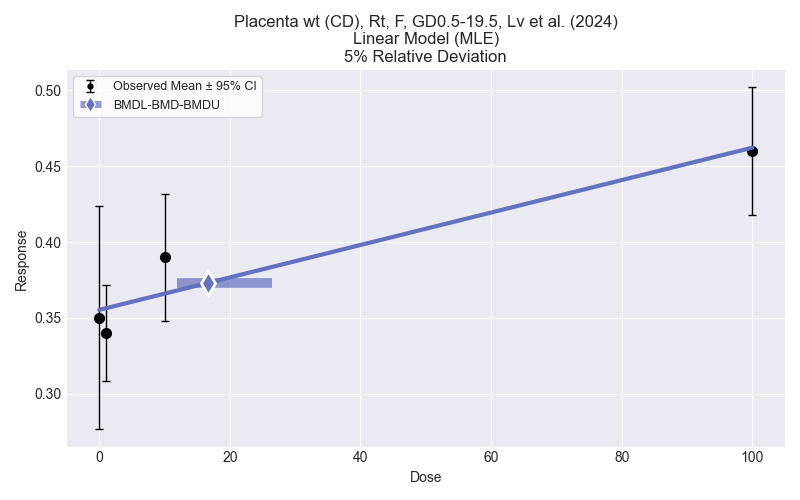


Linear Model
══════════════════════════════

Version: pybmds 25.1 (bmdscore 25.1)

Input Summary:
╒══════════════════════════════╤════════════════════════════╕
│ BMR │ 5% Relative Deviation │
│ Distribution │ Normal + Constant variance │
│ Modeling Direction │ Up (↑) │
│ Confidence Level (one sided) │ 0.95 │
│ Modeling Approach │ MLE │
│ Degree │ 1 │
╘══════════════════════════════╧════════════════════════════╛

Parameter Settings:
╒═════════════╤═══════════╤═════════╤════════╕
│ Parameter │ Initial │ Min │ Max │
╞═════════════╪═══════════╪═════════╪════════╡
│ g │ 0 │ -1e+06 │ 1e+06 │
│ b1 │ 0 │ -1e+06 │ 1e+06 │
│ alpha │ 0 │ -18 │ 18 │
╘═════════════╧═══════════╧═════════╧════════╛

Modeling Summary:
╒════════════════╤════════════╕
│ BMD │ 16.6204 │
│ BMDL │ 11.877 │
│ BMDU │ 26.4893 │
│ AIC │ -73.9354 │
│ Log-Likelihood │ 39.9677 │
│ P-Value │ 0.265128 │
│ Model d.f. │ 2 │
╘════════════════╧════════════╛

Model Parameters:
╒════════════╤════════════╤════════════╤═════════════╕
│ Variable │ Estimate │ On Bound │ Std Error │
╞════════════╪════════════╪════════════╪═════════════╡
│ g │ 0.355336 │ no │ 0.0112049 │
│ b1 │ 0.00106897 │ no │ 0.000222974 │
│ alpha │ 0.00209434 │ no │ 1.2662e-06 │
╘════════════╧════════════╧════════════╧═════════════╛

Goodness of Fit:
╒════════╤═════╤═══════════════╤═════════════════════╤═══════════════════╕
│ Dose │ N │ Sample Mean │ Model Fitted Mean │ Scaled Residual │
╞════════╪═════╪═══════════════╪═════════════════════╪═══════════════════╡
│ 0 │ 6 │ 0.35 │ 0.355336 │ -0.285605 │
│ 1 │ 6 │ 0.34 │ 0.356405 │ -0.878066 │
│ 10 │ 6 │ 0.39 │ 0.366026 │ 1.28321 │
│ 100 │ 6 │ 0.46 │ 0.462233 │ -0.11954 │
╘════════╧═════╧═══════════════╧═════════════════════╧═══════════════════╛
╒════════╤═════╤═════════════╤═══════════════════╕
│ Dose │ N │ Sample SD │ Model Fitted SD │
╞════════╪═════╪═════════════╪═══════════════════╡
│ 0 │ 6 │ 0.07 │ 0.0457639 │
│ 1 │ 6 │ 0.03 │ 0.0457639 │
│ 10 │ 6 │ 0.04 │ 0.0457639 │
│ 100 │ 6 │ 0.04 │ 0.0457639 │
╘════════╧═════╧═════════════╧═══════════════════╛

Likelihoods:
╒═════════╤══════════════════╤════════════╤══════════╕
│ Model │ Log-Likelihood │ # Params │ AIC │
╞═════════╪══════════════════╪════════════╪══════════╡
│ A1 │ 41.2952 │ 5 │ -72.5905 │
│ A2 │ 43.7548 │ 8 │ -71.5095 │
│ A3 │ 41.2952 │ 5 │ -72.5905 │
│ fitted │ 39.9677 │ 3 │ -73.9354 │
│ reduced │ 31.9067 │ 2 │ -59.8134 │
╘═════════╧══════════════════╧════════════╧══════════╛

Tests of Mean and Variance Fits:
╒════════╤══════════════════════════════╤═════════════╤═════════════╕
│ Name │ -2 * Log(Likelihood Ratio) │ Test d.f. │ P-Value │
╞════════╪══════════════════════════════╪═════════════╪═════════════╡
│ Test 1 │ 23.6961 │ 6 │ 0.000593912 │
│ Test 2 │ 4.91903 │ 3 │ 0.177823 │
│ Test 3 │ 4.91903 │ 3 │ 0.177823 │
│ Test 4 │ 2.65508 │ 2 │ 0.265128 │
╘════════╧══════════════════════════════╧═════════════╧═════════════╛
Test 1: Test the null hypothesis that responses and variances don't differ among dose levels
(A2 vs R). If this test fails to reject the null hypothesis (p-value > 0.05), there may not be
a dose-response.

Test 2: Test the null hypothesis that variances are homogenous (A1 vs A2). If this test fails to
reject the null hypothesis (p-value > 0.05), the simpler constant variance model may be appropriate.

Test 3: Test the null hypothesis that the variances are adequately modeled (A3 vs A2). If this test
fails to reject the null hypothesis (p-value > 0.05), it may be inferred that the variances have
been modeled appropriately.

Test 4: Test the null hypothesis that the model for the mean fits the data (Fitted vs A3). If this
test fails to reject the null hypothesis (p-value > 0.1), the user has support for use of the
selected model.

# Gestational BW gain, Rt, F, GD0.5-19.5, Lv et al. (2024)_drop

## Dataset

**Name:** Gestational BW gain, Rt, F, GD0.5-19.5, Lv et al. (2024)_drop

| Dose | N | Mean | Std. Dev. |
| --- | --- | --- | --- |
| 0 | 12 | 107.4 | 6.3 |
| 1 | 12 | 110.44 | 6.84 |
| 10 | 12 | 124.76 | 4.4 |

Test 1 Dose Response: <0.0001

Test 2 Homogeneity of Variance: 0.3013

Test 3 Variance Model Selection: 0.3013

## Settings

| Setting | Value |
| --- | --- |
| BMR | 10% Relative Deviation |
| Distribution | Normal + Constant variance |
| Adverse Direction | Up (↑) |
| Maximum Polynomial Degree | 2 |
| Confidence Level (one sided) | 0.95 |

## Maximum Likelihood Approach

| Model | BMDL | BMD | BMDU | *P*-Value | AIC | Scaled Residual at Control | Scaled Residual near BMD | Recommendation and Notes |
| --- | --- | --- | --- | --- | --- | --- | --- | --- |
| Exponential 3 | 5.483 | 6.626 | 10.005 | 0.525 | 233.712 | -0.426 | -0.041 | **Viable** |
| Exponential 5 | 1.031 | 4.271 | 9.804 | - | 237.309 | <0.001 | -0. | **Questionable** Zero degrees of freedom; saturated model BMD/BMDL ratio > 3.0 |
| Hill | 1.231 | 4.448 | 9.53 | - | 237.309 | -0. | <0.001 | **Questionable** Zero degrees of freedom; saturated model BMD/BMDL ratio > 3.0 |
| Polynomial 2 | 5.244 | 6.45 | 9.072 | 0.557 | 233.654 | -0.399 | -0.031 | **Viable** |
| Power^ab^ | 5.245 | 6.436 | 9.765 | 0.557 | 233.654 | -0.391 | -0.043 | **Recommended - Lowest AIC** |
| Linear | 5.245 | 6.436 | 8.265 | 0.557 | 233.654 | -0.391 | -0.043 | **Viable** |

^a^ BMDS recommended best fitting model

^b^ User selected best fitting model


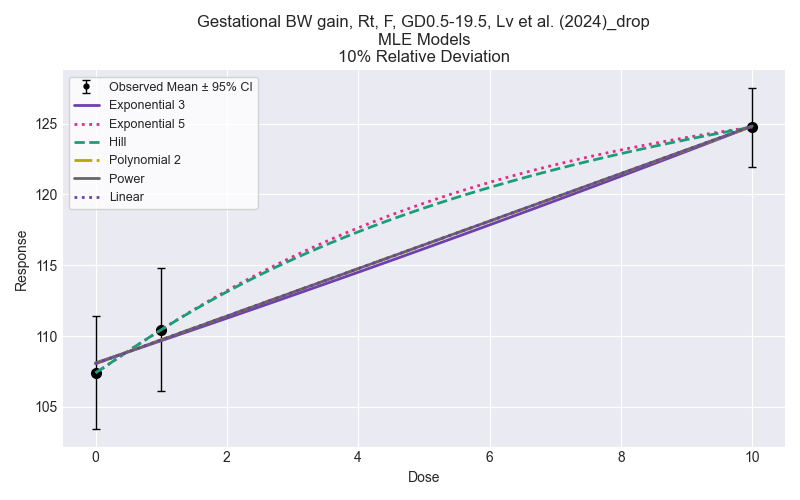


## Selected Model: Power


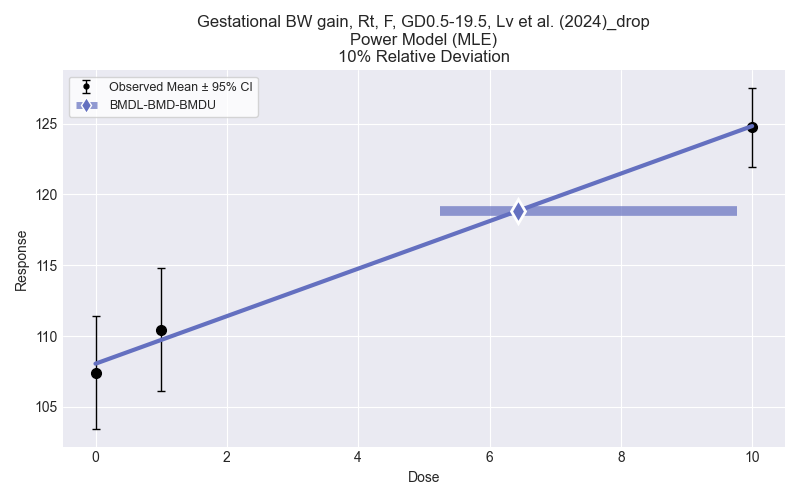


Power Model
══════════════════════════════

Version: pybmds 25.1 (bmdscore 25.1)

Input Summary:
╒══════════════════════════════╤════════════════════════════╕
│ BMR │ 10% Relative Deviation │
│ Distribution │ Normal + Constant variance │
│ Modeling Direction │ Up (↑) │
│ Confidence Level (one sided) │ 0.95 │
│ Modeling Approach │ MLE │
╘══════════════════════════════╧════════════════════════════╛

Parameter Settings:
╒═════════════╤═══════════╤═══════╤═══════╕
│ Parameter │ Initial │ Min │ Max │
╞═════════════╪═══════════╪═══════╪═══════╡
│ g │ 0 │ -100 │ 100 │
│ v │ 0 │ -100 │ 100 │
│ n │ 1 │ 1 │ 18 │
│ alpha │ 0 │ -18 │ 18 │
╘═════════════╧═══════════╧═══════╧═══════╛

Modeling Summary:
╒════════════════╤═════════════╕
│ BMD │ 6.43629 │
│ BMDL │ 5.24511 │
│ BMDU │ 9.76495 │
│ AIC │ 233.654 │
│ Log-Likelihood │ -113.827 │
│ P-Value │ 0.556939 │
│ Model d.f. │ 1 │
╘════════════════╧═════════════╛

Model Parameters:
╒════════════╤════════════╤════════════╤══════════════╕
│ Variable │ Estimate │ On Bound │ Std Error │
╞════════════╪════════════╪════════════╪══════════════╡
│ g │ 108.045 │ no │ 1.22878 │
│ v │ 1.67868 │ no │ 0.211772 │
│ n │ 1 │ yes │ Not Reported │
│ alpha │ 32.6501 │ no │ 251.265 │
╘════════════╧════════════╧════════════╧══════════════╛
Standard errors estimates are not generated for parameters estimated on corresponding bounds,
although sampling error is present for all parameters, as a rule. Standard error estimates may not
be reliable as a basis for confidence intervals or tests when one or more parameters are on bounds.


Goodness of Fit:
╒════════╤═════╤═══════════════╤═════════════════════╤═══════════════════╕
│ Dose │ N │ Sample Mean │ Model Fitted Mean │ Scaled Residual │
╞════════╪═════╪═══════════════╪═════════════════════╪═══════════════════╡
│ 0 │ 12 │ 107.4 │ 108.045 │ -0.390928 │
│ 1 │ 12 │ 110.44 │ 109.724 │ 0.434365 │
│ 10 │ 12 │ 124.76 │ 124.832 │ -0.0434365 │
╘════════╧═════╧═══════════════╧═════════════════════╧═══════════════════╛
╒════════╤═════╤═════════════╤═══════════════════╕
│ Dose │ N │ Sample SD │ Model Fitted SD │
╞════════╪═════╪═════════════╪═══════════════════╡
│ 0 │ 12 │ 6.3 │ 5.71403 │
│ 1 │ 12 │ 6.84 │ 5.71403 │
│ 10 │ 12 │ 4.4 │ 5.71403 │
╘════════╧═════╧═════════════╧═══════════════════╛

Likelihoods:
╒═════════╤══════════════════╤════════════╤═════════╕
│ Model │ Log-Likelihood │ # Params │ AIC │
╞═════════╪══════════════════╪════════════╪═════════╡
│ A1 │ -113.655 │ 4 │ 235.309 │
│ A2 │ -112.455 │ 6 │ 236.91 │
│ A3 │ -113.655 │ 4 │ 235.309 │
│ fitted │ -113.827 │ 3 │ 233.654 │
│ reduced │ -132.005 │ 2 │ 268.011 │
╘═════════╧══════════════════╧════════════╧═════════╛

Tests of Mean and Variance Fits:
╒════════╤══════════════════════════════╤═════════════╤═════════════╕
│ Name │ -2 * Log(Likelihood Ratio) │ Test d.f. │ P-Value │
╞════════╪══════════════════════════════╪═════════════╪═════════════╡
│ Test 1 │ 39.1009 │ 4 │ 6.63987e-08 │
│ Test 2 │ 2.39928 │ 2 │ 0.301302 │
│ Test 3 │ 2.39928 │ 2 │ 0.301302 │
│ Test 4 │ 0.345033 │ 1 │ 0.556939 │
╘════════╧══════════════════════════════╧═════════════╧═════════════╛
Test 1: Test the null hypothesis that responses and variances don't differ among dose levels
(A2 vs R). If this test fails to reject the null hypothesis (p-value > 0.05), there may not be
a dose-response.

Test 2: Test the null hypothesis that variances are homogenous (A1 vs A2). If this test fails to
reject the null hypothesis (p-value > 0.05), the simpler constant variance model may be appropriate.

Test 3: Test the null hypothesis that the variances are adequately modeled (A3 vs A2). If this test
fails to reject the null hypothesis (p-value > 0.05), it may be inferred that the variances have
been modeled appropriately.

Test 4: Test the null hypothesis that the model for the mean fits the data (Fitted vs A3). If this
test fails to reject the null hypothesis (p-value > 0.1), the user has support for use of the
selected model.

# Liver focal necrosis, Rt, F, GD6-20, DuPont (2010)_drop

## Dataset

**Name:** Liver focal necrosis, Rt, F, GD6-20, DuPont (2010)_drop

| Dose | N | Incidence |
| --- | --- | --- |
| 0 | 22 | 0 |
| 10 | 22 | 0 |
| 100 | 22 | 2 |

## Settings

| Setting | Value |
| --- | --- |
| BMR | 10% Extra Risk |
| Confidence Level (one sided) | 0.95 |
| Maximum Multistage Degree | 2 |

## Maximum Likelihood Approach

| Model | BMDL | BMD | BMDU | *P*-Value | AIC | Scaled Residual at Control | Scaled Residual near BMD | Recommendation and Notes |
| --- | --- | --- | --- | --- | --- | --- | --- | --- |
| Hill | - | - | - | - | 19.404 | -0.001 | - | **Unusable** Did not successfully execute. |
| Gamma | 314.85 | 612175 | - | - | 23.925 | -0.829 | 1.658 | **Questionable** Zero degrees of freedom; saturated model BMDL/highest dose ratio > 1.0 BMD/highest dose ratio > 1.0 BMD/BMDL ratio > 3.0 BMD/BMDL ratio > 20.0 |
| LogLogistic | 47.214 | 100.676 | - | 0.999 | 17.404 | -0.001 | 0 | **Viable** BMD/highest dose ratio > 1.0 |
| Multistage 1 | 46.04 | 122.142 | - | 0.9 | 15.803 | -0.001 | 0.141 | **Viable** BMD/highest dose ratio > 1.0 |
| Multistage 2 | 48.717 | 105.69 | - | 0.99 | 15.446 | -0.001 | 0.015 | **Viable** BMD/highest dose ratio > 1.0 |
| Weibull | 49.064 | 100.572 | - | 0.999 | 17.404 | -0.001 | <0.001 | **Viable** BMD/highest dose ratio > 1.0 |
| Logistic^ab^ | 79.151 | 100.671 | 232.874 | 1. | 15.404 | -0.001 | <0.001 | **Recommended - Lowest AIC** BMD/highest dose ratio > 1.0 |
| LogProbit | 41.466 | 101.106 | - | 0.999 | 17.404 | -0.001 | 0 | **Viable** BMD/highest dose ratio > 1.0 |
| Probit | 75.15 | 101.01 | - | 1. | 17.404 | -0. | -0. | **Viable** BMD/highest dose ratio > 1.0 |
| Quantal Linear | 46.041 | 122.142 | - | 0.9 | 15.803 | -0.001 | 0.141 | **Viable** BMD/highest dose ratio > 1.0 |

^a^ BMDS recommended best fitting model

^b^ User selected best fitting model; software crashed when including 1000 mg/kg group

## Selected Model: Logistic


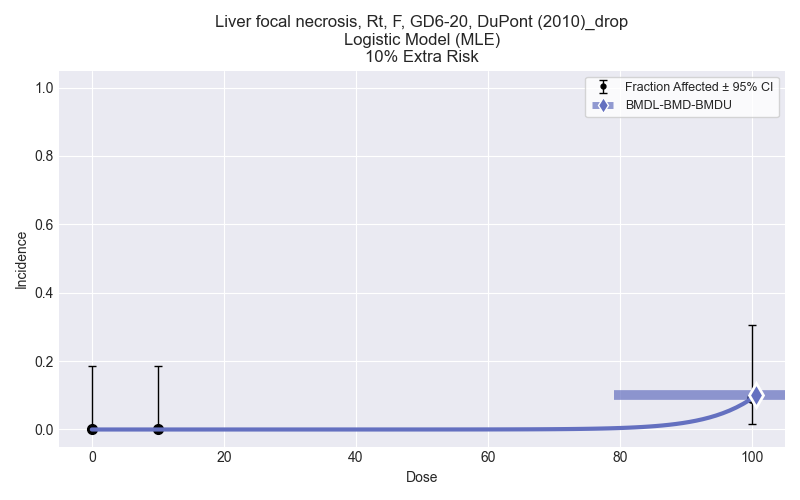


Logistic Model
══════════════════════════════

Version: pybmds 25.1 (bmdscore 25.1)

Input Summary:
╒══════════════════════════════╤══════════════════════════╕
│ BMR │ 10% Extra Risk │
│ Confidence Level (one sided) │ 0.95 │
│ Modeling approach │ frequentist_unrestricted │
╘══════════════════════════════╧══════════════════════════╛

Parameter Settings:
╒═════════════╤═══════════╤═══════╤═══════╕
│ Parameter │ Initial │ Min │ Max │
╞═════════════╪═══════════╪═══════╪═══════╡
│ a │ 0 │ -18 │ 18 │
│ b │ 0 │ 0 │ 100 │
╘═════════════╧═══════════╧═══════╧═══════╛

Modeling Summary:
╒════════════════╤═══════════════╕
│ BMD │ 100.671 │
│ BMDL │ 79.1514 │
│ BMDU │ 232.874 │
│ AIC │ 15.404 │
│ Log-Likelihood │ -6.702 │
│ P-Value │ 0.999999 │
│ Overall d.f. │ 2 │
│ Chi² │ 1.94516e-06 │
╘════════════════╧═══════════════╛

Model Parameters:
╒════════════╤════════════╤════════════╤══════════════╕
│ Variable │ Estimate │ On Bound │ Std Error │
╞════════════╪════════════╪════════════╪══════════════╡
│ a │ -18 │ yes │ Not Reported │
│ b │ 0.156974 │ no │ 0.0074162 │
╘════════════╧════════════╧════════════╧══════════════╛
Standard errors estimates are not generated for parameters estimated on corresponding bounds,
although sampling error is present for all parameters, as a rule. Standard error estimates may not
be reliable as a basis for confidence intervals or tests when one or more parameters are on bounds.


Goodness of Fit:
╒════════╤════════╤════════════╤════════════╤═════════════╤═══════════════════╕
│ Dose │ Size │ Observed │ Expected │ Est Prob │ Scaled Residual │
╞════════╪════════╪════════════╪════════════╪═════════════╪═══════════════════╡
│ 0 │ 22 │ 0 │ 3.3506e-07 │ 1.523e-08 │ -0.000578843 │
│ 10 │ 22 │ 0 │ 1.6101e-06 │ 7.31862e-08 │ -0.0012689 │
│ 100 │ 22 │ 2 │ 2 │ 0.0909091 │ 1.30725e-07 │
╘════════╧════════╧════════════╧════════════╧═════════════╧═══════════════════╛

Analysis of Deviance:
╒═══════════════╤══════════════════╤════════════╤═════════════╤═════════════╤═══════════╕
│ Model │ Log-Likelihood │ # Params │ Deviance │ Test d.f. │ P-Value │
╞═══════════════╪══════════════════╪════════════╪═════════════╪═════════════╪═══════════╡
│ Full model │ -6.70199 │ 3 │ - │ - │ - │
│ Fitted model │ -6.702 │ 1 │ 3.89031e-06 │ 2 │ 0.999998 │
│ Reduced model │ -8.9624 │ 1 │ 4.52081 │ 2 │ 0.104308 │
╘═══════════════╧══════════════════╧════════════╧═════════════╧═════════════╧═══════════╛

# Gestational BW gain, Rt, F, GD6-20, DuPont (2010)

## Dataset

**Name:** Gestational BW gain, Rt, F, GD6-20, DuPont (2010)

| Dose | N | Mean | Std. Dev. |
| --- | --- | --- | --- |
| 0 | 22 | 145 | 18.4 |
| 10 | 21 | 141 | 13.1 |
| 100 | 17 | 139 | 13.9 |
| 1000 | 14 | 109 | 17.7 |

Test 1 Dose Response: <0.0001

Test 2 Homogeneity of Variance: 0.3447

Test 3 Variance Model Selection: 0.3447

## Settings

| Setting | Value |
| --- | --- |
| BMR | 10% Relative Deviation |
| Distribution | Normal + Constant variance |
| Adverse Direction | Down (↓) |
| Maximum Polynomial Degree | 3 |
| Confidence Level (one sided) | 0.95 |

## Maximum Likelihood Approach

| Model | BMDL | BMD | BMDU | *P*-Value | AIC | Scaled Residual at Control | Scaled Residual near BMD | Recommendation and Notes |
| --- | --- | --- | --- | --- | --- | --- | --- | --- |
| Exponential 3^a^ | 306.107 | 385.568 | 964.437 | 0.744 | 621.936 | 0.559 | -0.077 | **Viable** |
| Exponential 5^b^ | 101.772 | 334.28 | 962.729 | 0.454 | 623.906 | 0.487 | 0.072 | **Recommended - Lowest BMDL** BMD/BMDL ratio > 3.0 |
| Hill | 121.981 | 374.378 | 916.754 | 0.447 | 623.924 | 0.544 | -0.045 | **Viable** BMD/BMDL ratio > 3.0 |
| Polynomial 2 | 344.135 | 419.288 | 729.162 | 0.729 | 621.977 | 0.601 | -0.159 | **Viable** |
| Polynomial 3 | 339.185 | 595.757 | 608.089 | 0.296 | 624.436 | 0.813 | 0.013 | **Viable** |
| Power | 344.136 | 419.305 | 965.782 | 0.729 | 621.977 | 0.601 | -0.159 | **Viable** |
| Linear | 344.136 | 419.305 | 539.045 | 0.729 | 621.977 | 0.601 | -0.159 | **Viable** |

^a^ lower AIC, no warnings, more consistent with data

^b^ BMDS recommended best fitting model


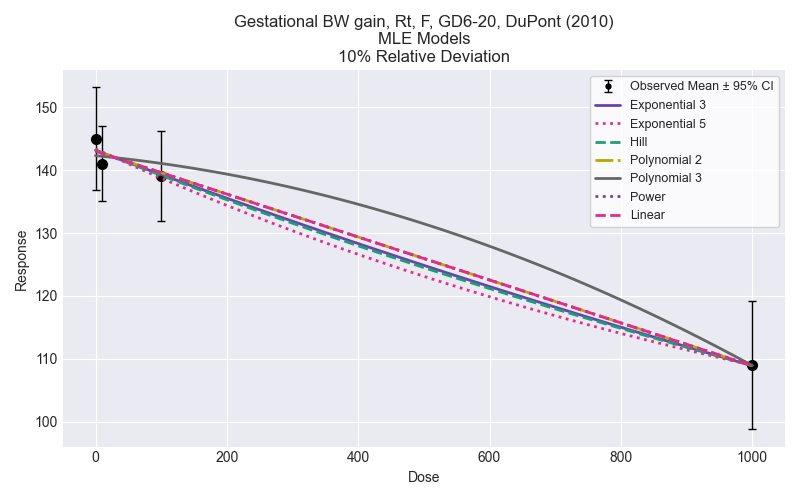


## Selected Model: Exponential 3


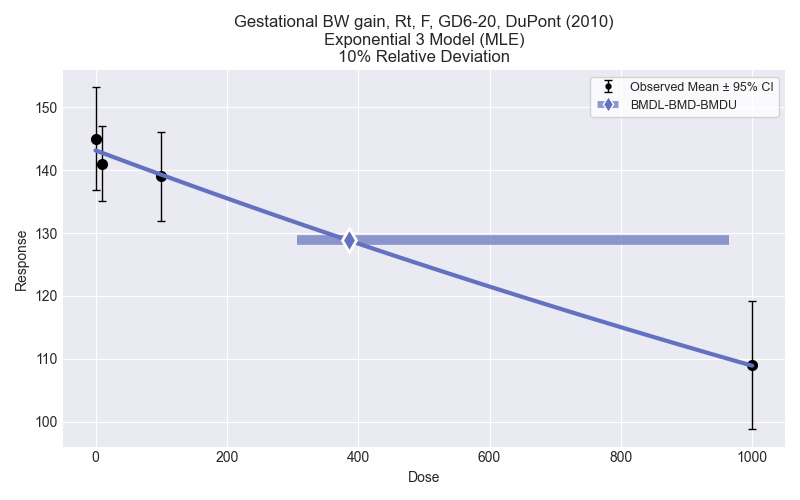


Exponential 3 Model
══════════════════════════════

Version: pybmds 25.1 (bmdscore 25.1)

Input Summary:
╒══════════════════════════════╤════════════════════════════╕
│ BMR │ 10% Relative Deviation │
│ Distribution │ Normal + Constant variance │
│ Modeling Direction │ Down (↓) │
│ Confidence Level (one sided) │ 0.95 │
│ Modeling Approach │ MLE │
╘══════════════════════════════╧════════════════════════════╛

Parameter Settings:
╒═════════════╤═══════════╤═══════╤═══════╕
│ Parameter │ Initial │ Min │ Max │
╞═════════════╪═══════════╪═══════╪═══════╡
│ a │ 0 │ 0 │ 100 │
│ b │ 0 │ 0 │ 100 │
│ c │ 0 │ -20 │ 0 │
│ d │ 1 │ 1 │ 18 │
│ log-alpha │ 0 │ -18 │ 18 │
╘═════════════╧═══════════╧═══════╧═══════╛

Modeling Summary:
╒════════════════╤═════════════╕
│ BMD │ 385.568 │
│ BMDL │ 306.107 │
│ BMDU │ 964.437 │
│ AIC │ 621.936 │
│ Log-Likelihood │ -307.968 │
│ P-Value │ 0.744393 │
│ Model d.f. │ 2 │
╘════════════════╧═════════════╛

Model Parameters:
╒════════════╤═══════════════╤════════════╤══════════════╕
│ Variable │ Estimate │ On Bound │ Std Error │
╞════════════╪═══════════════╪════════════╪══════════════╡
│ a │ 143.148 │ no │ 2.09088 │
│ b │ 0.000273261 │ no │ 4.16178e-05 │
│ d │ 1 │ yes │ Not Reported │
│ log-alpha │ 5.48558 │ no │ 0.164399 │
╘════════════╧═══════════════╧════════════╧══════════════╛
Standard errors estimates are not generated for parameters estimated on corresponding bounds,
although sampling error is present for all parameters, as a rule. Standard error estimates may not
be reliable as a basis for confidence intervals or tests when one or more parameters are on bounds.


Goodness of Fit:
╒════════╤═════╤═══════════════╤═════════════════════╤═══════════════════╕
│ Dose │ N │ Sample Mean │ Model Fitted Mean │ Scaled Residual │
╞════════╪═════╪═══════════════╪═════════════════════╪═══════════════════╡
│ 0 │ 22 │ 145 │ 143.148 │ 0.559332 │
│ 10 │ 21 │ 141 │ 142.757 │ -0.51856 │
│ 100 │ 17 │ 139 │ 139.289 │ -0.0768086 │
│ 1000 │ 14 │ 109 │ 108.921 │ 0.0191478 │
╘════════╧═════╧═══════════════╧═════════════════════╧═══════════════════╛
╒════════╤═════╤═════════════╤═══════════════════╕
│ Dose │ N │ Sample SD │ Model Fitted SD │
╞════════╪═════╪═════════════╪═══════════════════╡
│ 0 │ 22 │ 18.4 │ 15.5302 │
│ 10 │ 21 │ 13.1 │ 15.5302 │
│ 100 │ 17 │ 13.9 │ 15.5302 │
│ 1000 │ 14 │ 17.7 │ 15.5302 │
╘════════╧═════╧═════════════╧═══════════════════╛

Likelihoods:
╒═════════╤══════════════════╤════════════╤═════════╕
│ Model │ Log-Likelihood │ # Params │ AIC │
╞═════════╪══════════════════╪════════════╪═════════╡
│ A1 │ -307.673 │ 5 │ 625.345 │
│ A2 │ -306.012 │ 8 │ 628.024 │
│ A3 │ -307.673 │ 5 │ 625.345 │
│ fitted │ -307.968 │ 3 │ 621.936 │
│ reduced │ -327.629 │ 2 │ 659.258 │
╘═════════╧══════════════════╧════════════╧═════════╛

Tests of Mean and Variance Fits:
╒════════╤══════════════════════════════╤═════════════╤════════════╕
│ Name │ -2 * Log(Likelihood Ratio) │ Test d.f. │ P-Value │
╞════════╪══════════════════════════════╪═════════════╪════════════╡
│ Test 1 │ 43.2339 │ 6 │ 1.0485e-07 │
│ Test 2 │ 3.3213 │ 3 │ 0.344688 │
│ Test 3 │ 3.3213 │ 3 │ 0.344688 │
│ Test 4 │ 0.590372 │ 2 │ 0.744393 │
╘════════╧══════════════════════════════╧═════════════╧════════════╛
Test 1: Test the null hypothesis that responses and variances don't differ among dose levels
(A2 vs R). If this test fails to reject the null hypothesis (p-value > 0.05), there may not be
a dose-response.

Test 2: Test the null hypothesis that variances are homogenous (A1 vs A2). If this test fails to
reject the null hypothesis (p-value > 0.05), the simpler constant variance model may be appropriate.

Test 3: Test the null hypothesis that the variances are adequately modeled (A3 vs A2). If this test
fails to reject the null hypothesis (p-value > 0.05), it may be inferred that the variances have
been modeled appropriately.

Test 4: Test the null hypothesis that the model for the mean fits the data (Fitted vs A3). If this
test fails to reject the null hypothesis (p-value > 0.1), the user has support for use of the
selected model.

# Gestational BW GD20, Rt, F, GD6-20, DuPont (2010)

## Dataset

**Name:** Gestational BW GD20, Rt, F, GD6-20, DuPont (2010)

| Dose | N | Mean | Std. Dev. |
| --- | --- | --- | --- |
| 0 | 22 | 411 | 28.6 |
| 10 | 21 | 409 | 17.3 |
| 100 | 21 | 410 | 20.7 |
| 1000 | 21 | 392 | 20.3 |

Test 1 Dose Response: 0.0114

Test 2 Homogeneity of Variance: 0.105

Test 3 Variance Model Selection: 0.105

## Settings

| Setting | Value |
| --- | --- |
| BMR | 10% Relative Deviation |
| Distribution | Normal + Constant variance |
| Adverse Direction | Down (↓) |
| Maximum Polynomial Degree | 3 |
| Confidence Level (one sided) | 0.95 |

## Maximum Likelihood Approach

| Model | BMDL | BMD | BMDU | *P*-Value | AIC | Scaled Residual at Control | Scaled Residual near BMD | Recommendation and Notes |
| --- | --- | --- | --- | --- | --- | --- | --- | --- |
| Exponential 3 | 1024.219 | 1352.461 | 4555.065 | 0.762 | 772.345 | 0.211 | -0. | **Viable** BMDL/highest dose ratio > 1.0 BMD/highest dose ratio > 1.0 |
| Exponential 5 | 1024.481 | 1353.216 | 3821.454 | - | 774.345 | 0.211 | -0. | **Questionable** Zero degrees of freedom; saturated model BMDL/highest dose ratio > 1.0 BMD/highest dose ratio > 1.0 |
| Hill | 1047.153 | 1184.312 | 1336.112 | - | 774.345 | 0.213 | <0.001 | **Questionable** Zero degrees of freedom; saturated model BMDL/highest dose ratio > 1.0 BMD/highest dose ratio > 1.0 |
| Polynomial 2 | 1228.899 | 2135.448 | 4434.529 | 0.922 | 770.416 | 0.075 | -0.011 | **Viable** BMDL/highest dose ratio > 1.0 BMD/highest dose ratio > 1.0 |
| Polynomial 3^ab^ | 1149.795 | 1344.916 | 4374.991 | 0.956 | 770.345 | 0.189 | 0.002 | **Recommended - Lowest AIC** BMDL/highest dose ratio > 1.0 BMD/highest dose ratio > 1.0 |
| Power | 1021.777 | 1046.766 | 4375.253 | 0.762 | 772.345 | 0.213 | -0. | **Viable** BMDL/highest dose ratio > 1.0 BMD/highest dose ratio > 1.0 |
| Linear | 1481.223 | 2214.406 | 4438.337 | 0.92 | 770.421 | 0.076 | -0.022 | **Viable** BMDL/highest dose ratio > 1.0 BMD/highest dose ratio > 1.0 |

^a^ BMDS recommended best fitting model

^b^ User selected best fitting model; note BMDL > highest dose


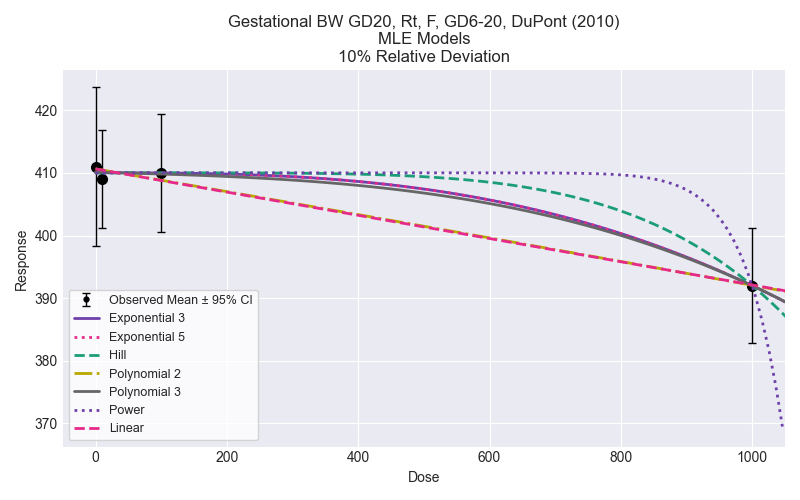


## Selected Model: Polynomial 3


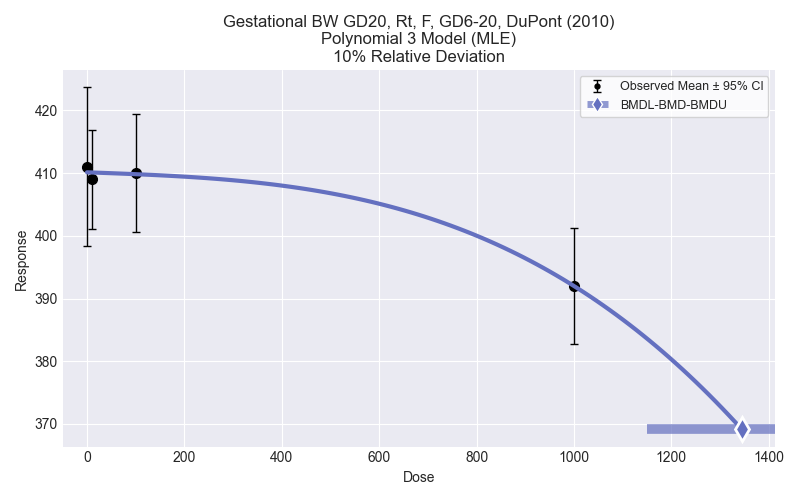


Polynomial 3 Model
══════════════════════════════

Version: pybmds 25.1 (bmdscore 25.1)

Input Summary:
╒══════════════════════════════╤════════════════════════════╕
│ BMR │ 10% Relative Deviation │
│ Distribution │ Normal + Constant variance │
│ Modeling Direction │ Down (↓) │
│ Confidence Level (one sided) │ 0.95 │
│ Modeling Approach │ MLE │
│ Degree │ 3 │
╘══════════════════════════════╧════════════════════════════╛

Parameter Settings:
╒═════════════╤═══════════╤═════════╤════════╕
│ Parameter │ Initial │ Min │ Max │
╞═════════════╪═══════════╪═════════╪════════╡
│ g │ 0 │ -1e+06 │ 1e+06 │
│ b1 │ 0 │ -1e+06 │ 0 │
│ b2 │ 0 │ -1e+06 │ 0 │
│ b3 │ 0 │ -1e+06 │ 0 │
│ alpha │ 0 │ -18 │ 18 │
╘═════════════╧═══════════╧═════════╧════════╛

Modeling Summary:
╒════════════════╤═════════════╕
│ BMD │ 1344.92 │
│ BMDL │ 1149.8 │
│ BMDU │ 4374.99 │
│ AIC │ 770.345 │
│ Log-Likelihood │ -382.172 │
│ P-Value │ 0.955599 │
│ Model d.f. │ 2 │
╘════════════════╧═════════════╛

Model Parameters:
╒════════════╤═══════════════╤════════════╤══════════════╕
│ Variable │ Estimate │ On Bound │ Std Error │
╞════════════╪═══════════════╪════════════╪══════════════╡
│ g │ 410.127 │ no │ 6.26048 │
│ b1 │ -0.00285081 │ no │ 0.157713 │
│ b2 │ -9.55912e-09 │ yes │ Not Reported │
│ b3 │ -1.52758e-08 │ yes │ Not Reported │
│ alpha │ 470.704 │ no │ 33984.3 │
╘════════════╧═══════════════╧════════════╧══════════════╛
Standard errors estimates are not generated for parameters estimated on corresponding bounds,
although sampling error is present for all parameters, as a rule. Standard error estimates may not
be reliable as a basis for confidence intervals or tests when one or more parameters are on bounds.


Goodness of Fit:
╒════════╤═════╤═══════════════╤═════════════════════╤═══════════════════╕
│ Dose │ N │ Sample Mean │ Model Fitted Mean │ Scaled Residual │
╞════════╪═════╪═══════════════╪═════════════════════╪═══════════════════╡
│ 0 │ 22 │ 411 │ 410.127 │ 0.188802 │
│ 10 │ 21 │ 409 │ 410.098 │ -0.231954 │
│ 100 │ 21 │ 410 │ 409.826 │ 0.0367026 │
│ 1000 │ 21 │ 392 │ 391.99 │ 0.00201189 │
╘════════╧═════╧═══════════════╧═════════════════════╧═══════════════════╛
╒════════╤═════╤═════════════╤═══════════════════╕
│ Dose │ N │ Sample SD │ Model Fitted SD │
╞════════╪═════╪═════════════╪═══════════════════╡
│ 0 │ 22 │ 28.6 │ 21.6957 │
│ 10 │ 21 │ 17.3 │ 21.6957 │
│ 100 │ 21 │ 20.7 │ 21.6957 │
│ 1000 │ 21 │ 20.3 │ 21.6957 │
╘════════╧═════╧═════════════╧═══════════════════╛

Likelihoods:
╒═════════╤══════════════════╤════════════╤═════════╕
│ Model │ Log-Likelihood │ # Params │ AIC │
╞═════════╪══════════════════╪════════════╪═════════╡
│ A1 │ -382.127 │ 5 │ 774.254 │
│ A2 │ -379.057 │ 8 │ 774.114 │
│ A3 │ -382.127 │ 5 │ 774.254 │
│ fitted │ -382.172 │ 3 │ 770.345 │
│ reduced │ -387.301 │ 2 │ 778.602 │
╘═════════╧══════════════════╧════════════╧═════════╛

Tests of Mean and Variance Fits:
╒════════╤══════════════════════════════╤═════════════╤═══════════╕
│ Name │ -2 * Log(Likelihood Ratio) │ Test d.f. │ P-Value │
╞════════╪══════════════════════════════╪═════════════╪═══════════╡
│ Test 1 │ 16.4879 │ 6 │ 0.0113613 │
│ Test 2 │ 6.14048 │ 3 │ 0.104972 │
│ Test 3 │ 6.14048 │ 3 │ 0.104972 │
│ Test 4 │ 0.0908333 │ 2 │ 0.955599 │
╘════════╧══════════════════════════════╧═════════════╧═══════════╛
Test 1: Test the null hypothesis that responses and variances don't differ among dose levels
(A2 vs R). If this test fails to reject the null hypothesis (p-value > 0.05), there may not be
a dose-response.

Test 2: Test the null hypothesis that variances are homogenous (A1 vs A2). If this test fails to
reject the null hypothesis (p-value > 0.05), the simpler constant variance model may be appropriate.

Test 3: Test the null hypothesis that the variances are adequately modeled (A3 vs A2). If this test
fails to reject the null hypothesis (p-value > 0.05), it may be inferred that the variances have
been modeled appropriately.

Test 4: Test the null hypothesis that the model for the mean fits the data (Fitted vs A3). If this
test fails to reject the null hypothesis (p-value > 0.1), the user has support for use of the
selected model.

# Maternal liver wt, Rt, F, GD6-20, DuPont (2010)

## Dataset

**Name:** Maternal liver wt, Rt, F, GD6-20, DuPont (2010)

| Dose | N | Mean | Std. Dev. |
| --- | --- | --- | --- |
| 0 | 22 | 14.82 | 1.552 |
| 10 | 21 | 14.93 | 1.308 |
| 100 | 21 | 16.61 | 1.946 |

Test 1 Dose Response: 0.001

Test 2 Homogeneity of Variance: 0.1872

Test 3 Variance Model Selection: 0.1872

## Settings

| Setting | Value |
| --- | --- |
| BMR | 10% Relative Deviation |
| Distribution | Normal + Constant variance |
| Adverse Direction | Up (↑) |
| Maximum Polynomial Degree | 2 |
| Confidence Level (one sided) | 0.95 |

## Maximum Likelihood Approach

| Model | BMDL | BMD | BMDU | *P*-Value | AIC | Scaled Residual at Control | Scaled Residual near BMD | Recommendation and Notes |
| --- | --- | --- | --- | --- | --- | --- | --- | --- |
| Exponential 3 | 59.037 | 85.992 | 136.761 | - | 248.482 | -0. | -0. | **Questionable** Zero degrees of freedom; saturated model |
| Exponential 5 | 10.366 | 83.788 | 138.337 | - | 250.482 | <0.001 | -0. | **Questionable** Zero degrees of freedom; saturated model BMD/BMDL ratio > 3.0 |
| Hill | 11.687 | 84.591 | - | - | 250.482 | -0. | -0. | **Questionable** Zero degrees of freedom; saturated model BMD/BMDL ratio > 3.0 |
| Polynomial 2 | 56.881 | 86.981 | 138.319 | - | 248.482 | 0.011 | -0.002 | **Questionable** Zero degrees of freedom; saturated model |
| Power | 56.895 | 85.569 | 138.348 | - | 248.482 | -0. | -0. | **Questionable** Zero degrees of freedom; saturated model |
| Linear^ab^ | 56.822 | 81.274 | 138.721 | 0.881 | 246.504 | 0.098 | 0.011 | **Recommended - Lowest AIC** |

^a^ BMDS recommended best fitting model

^b^ User selected best fitting model


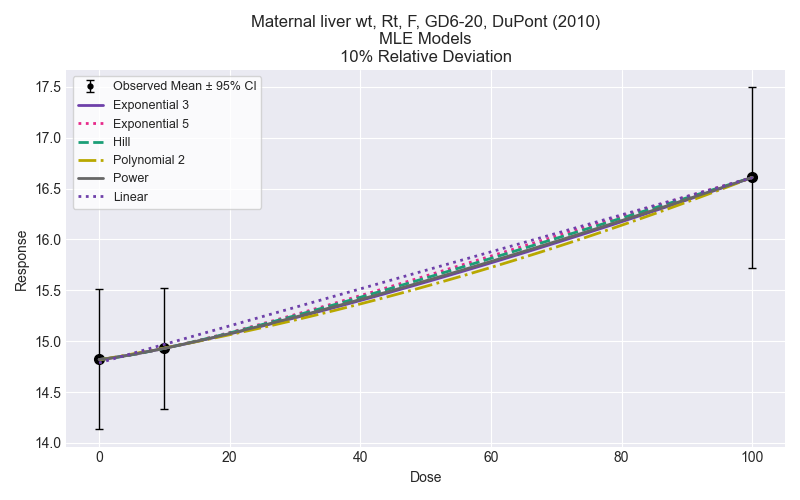


## Selected Model: Linear


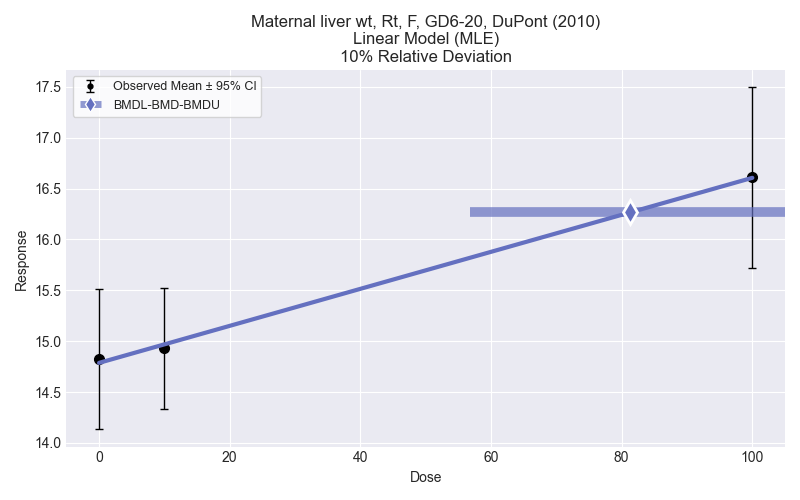


Linear Model
══════════════════════════════

Version: pybmds 25.1 (bmdscore 25.1)

Input Summary:
╒══════════════════════════════╤════════════════════════════╕
│ BMR │ 10% Relative Deviation │
│ Distribution │ Normal + Constant variance │
│ Modeling Direction │ Up (↑) │
│ Confidence Level (one sided) │ 0.95 │
│ Modeling Approach │ MLE │
│ Degree │ 1 │
╘══════════════════════════════╧════════════════════════════╛

Parameter Settings:
╒═════════════╤═══════════╤═════════╤════════╕
│ Parameter │ Initial │ Min │ Max │
╞═════════════╪═══════════╪═════════╪════════╡
│ g │ 0 │ -1e+06 │ 1e+06 │
│ b1 │ 0 │ -1e+06 │ 1e+06 │
│ alpha │ 0 │ -18 │ 18 │
╘═════════════╧═══════════╧═════════╧════════╛

Modeling Summary:
╒════════════════╤═════════════╕
│ BMD │ 81.2739 │
│ BMDL │ 56.8217 │
│ BMDU │ 138.721 │
│ AIC │ 246.504 │
│ Log-Likelihood │ -120.252 │
│ P-Value │ 0.881164 │
│ Model d.f. │ 1 │
╘════════════════╧═════════════╛

Model Parameters:
╒════════════╤════════════╤════════════╤═════════════╕
│ Variable │ Estimate │ On Bound │ Std Error │
╞════════════╪════════════╪════════════╪═════════════╡
│ g │ 14.7868 │ no │ 0.254165 │
│ b1 │ 0.0181937 │ no │ 0.00441489 │
│ alpha │ 2.50929 │ no │ 1.11307 │
╘════════════╧════════════╧════════════╧═════════════╛

Goodness of Fit:
╒════════╤═════╤═══════════════╤═════════════════════╤═══════════════════╕
│ Dose │ N │ Sample Mean │ Model Fitted Mean │ Scaled Residual │
╞════════╪═════╪═══════════════╪═════════════════════╪═══════════════════╡
│ 0 │ 22 │ 14.82 │ 14.7868 │ 0.0984303 │
│ 10 │ 21 │ 14.93 │ 14.9687 │ -0.111941 │
│ 100 │ 21 │ 16.61 │ 16.6061 │ 0.0111941 │
╘════════╧═════╧═══════════════╧═════════════════════╧═══════════════════╛
╒════════╤═════╤═════════════╤═══════════════════╕
│ Dose │ N │ Sample SD │ Model Fitted SD │
╞════════╪═════╪═════════════╪═══════════════════╡
│ 0 │ 22 │ 1.552 │ 1.58407 │
│ 10 │ 21 │ 1.308 │ 1.58407 │
│ 100 │ 21 │ 1.946 │ 1.58407 │
╘════════╧═════╧═════════════╧═══════════════════╛

Likelihoods:
╒═════════╤══════════════════╤════════════╤═════════╕
│ Model │ Log-Likelihood │ # Params │ AIC │
╞═════════╪══════════════════╪════════════╪═════════╡
│ A1 │ -120.241 │ 4 │ 248.482 │
│ A2 │ -118.566 │ 6 │ 249.131 │
│ A3 │ -120.241 │ 4 │ 248.482 │
│ fitted │ -120.252 │ 3 │ 246.504 │
│ reduced │ -127.782 │ 2 │ 259.565 │
╘═════════╧══════════════════╧════════════╧═════════╛

Tests of Mean and Variance Fits:
╒════════╤══════════════════════════════╤═════════════╤════════════╕
│ Name │ -2 * Log(Likelihood Ratio) │ Test d.f. │ P-Value │
╞════════╪══════════════════════════════╪═════════════╪════════════╡
│ Test 1 │ 18.4339 │ 4 │ 0.00101497 │
│ Test 2 │ 3.35071 │ 2 │ 0.187242 │
│ Test 3 │ 3.35071 │ 2 │ 0.187242 │
│ Test 4 │ 0.0223484 │ 1 │ 0.881164 │
╘════════╧══════════════════════════════╧═════════════╧════════════╛
Test 1: Test the null hypothesis that responses and variances don't differ among dose levels
(A2 vs R). If this test fails to reject the null hypothesis (p-value > 0.05), there may not be
a dose-response.

Test 2: Test the null hypothesis that variances are homogenous (A1 vs A2). If this test fails to
reject the null hypothesis (p-value > 0.05), the simpler constant variance model may be appropriate.

Test 3: Test the null hypothesis that the variances are adequately modeled (A3 vs A2). If this test
fails to reject the null hypothesis (p-value > 0.05), it may be inferred that the variances have
been modeled appropriately.

Test 4: Test the null hypothesis that the model for the mean fits the data (Fitted vs A3). If this
test fails to reject the null hypothesis (p-value > 0.1), the user has support for use of the
selected model.

# HFPO-DA_Conley19_Dev_Rt_Maternal

**Report Generated:** 2026-Feb-16 07:02 UTC

**BMDS Desktop Version:** 25.1 (pybmds 25.1; bmdscore 25.1)

# Gestational BW gain, Rt, F, GD14-18, Conley et al. (2019)

## Dataset

**Name:** Gestational BW gain, Rt, F, GD14-18, Conley et al. (2019)

| Dose | N | Mean | Std. Dev. |
| --- | --- | --- | --- |
| 0 | 9 | 33.7 | 9 |
| 1 | 6 | 32.8 | 10.8 |
| 3 | 6 | 37 | 13.7 |
| 10 | 6 | 33.3 | 6.9 |
| 30 | 6 | 33.6 | 3.9 |
| 62.5 | 3 | 33.4 | 7.4 |
| 125 | 3 | 35.5 | 2.6 |
| 250 | 3 | 20.9 | 6.1 |

Test 1 Dose Response: 0.0457

Test 2 Homogeneity of Variance: 0.0248

Test 3 Variance Model Selection: 0.0522

## Settings

| Setting | Value |
| --- | --- |
| BMR | 10% Relative Deviation |
| Distribution | Normal + Nonconstant variance |
| Adverse Direction | Down (↓) |
| Maximum Polynomial Degree | 3 |
| Confidence Level (one sided) | 0.95 |

## Maximum Likelihood Approach

| Model | BMDL | BMD | BMDU | *P*-Value | AIC | Scaled Residual at Control | Scaled Residual near BMD | Recommendation and Notes |
| --- | --- | --- | --- | --- | --- | --- | --- | --- |
| Exponential 3 | 50.362 | 178.845 | 240.009 | 0.608 | 303.801 | -0.166 | 0.432 | **Viable** BMD/BMDL ratio > 3.0 |
| Exponential 5 | 49.527 | 178.845 | 240.008 | 0.462 | 305.801 | -0.166 | 0.432 | **Viable** BMD/BMDL ratio > 3.0 |
| Hill | 140.781 | 180.077 | 238.044 | 0.463 | 305.8 | -0.168 | 0.446 | **Viable** |
| Polynomial 2 | 54.99 | 128.223 | 173.939 | 0.703 | 302.007 | -0.261 | 1.006 | **Viable** |
| Polynomial 3^ab^ | 55.995 | 147.242 | 194.476 | 0.734 | 301.77 | -0.246 | 0.779 | **Recommended - Lowest AIC** |
| Power | 55.862 | 180.111 | 219.104 | 0.608 | 303.8 | -0.169 | 0.446 | **Viable** BMD/BMDL ratio > 3.0 |
| Linear | 51.206 | 71.175 | 152.287 | 0.551 | 303.144 | -0.464 | 0.315 | **Viable** |

^a^ BMDS recommended best fitting model

^b^ User selected best fitting model


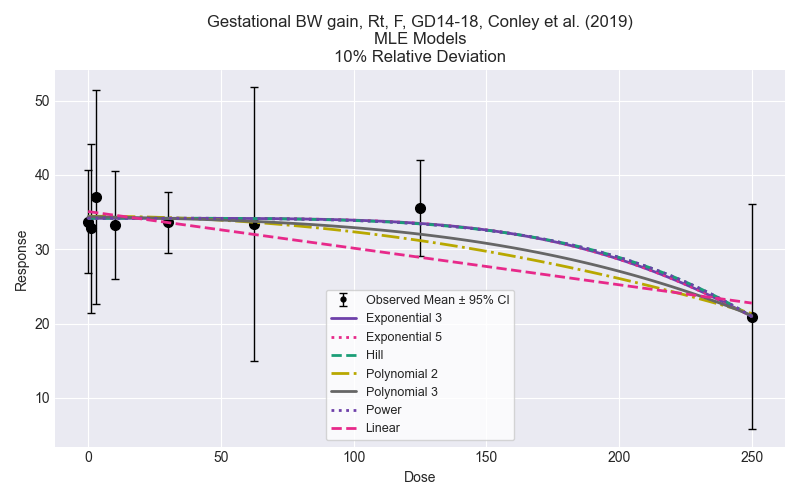


## Selected Model: Polynomial 3


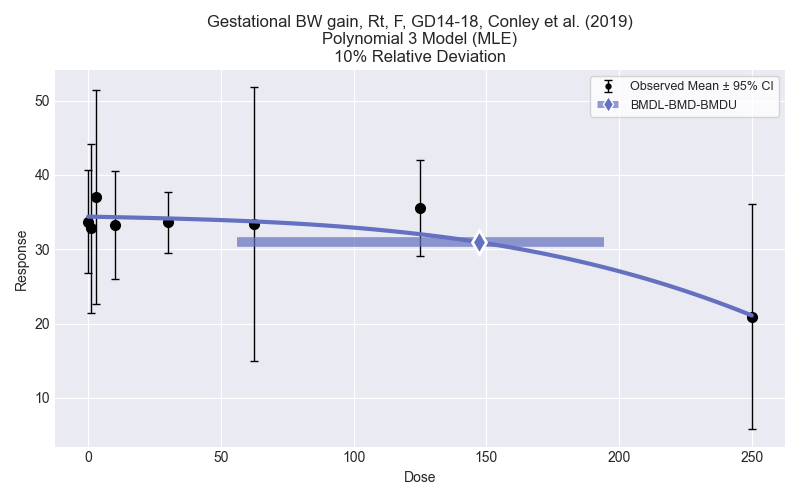


Polynomial 3 Model
══════════════════════════════

Version: pybmds 25.1 (bmdscore 25.1)

Input Summary:
╒══════════════════════════════╤═══════════════════════════════╕
│ BMR │ 10% Relative Deviation │
│ Distribution │ Normal + Nonconstant variance │
│ Modeling Direction │ Down (↓) │
│ Confidence Level (one sided) │ 0.95 │
│ Modeling Approach │ MLE │
│ Degree │ 3 │
╘══════════════════════════════╧═══════════════════════════════╛

Parameter Settings:
╒═════════════╤═══════════╤═════════╤═══════╕
│ Parameter │ Initial │ Min │ Max │
╞═════════════╪═══════════╪═════════╪═══════╡
│ g │ 0 │ 0 │ 1000 │
│ b1 │ 0 │ -18 │ 0 │
│ b2 │ 0 │ -1e+06 │ 0 │
│ b3 │ 0 │ -1e+06 │ 0 │
│ rho │ 0 │ 0 │ 18 │
│ alpha │ 0 │ -18 │ 18 │
╘═════════════╧═══════════╧═════════╧═══════╛

Modeling Summary:
╒════════════════╤═════════════╕
│ BMD │ 147.242 │
│ BMDL │ 55.9953 │
│ BMDU │ 194.476 │
│ AIC │ 301.77 │
│ Log-Likelihood │ -146.885 │
│ P-Value │ 0.734431 │
│ Model d.f. │ 6 │
╘════════════════╧═════════════╛

Model Parameters:
╒════════════╤══════════════╤════════════╤══════════════╕
│ Variable │ Estimate │ On Bound │ Std Error │
╞════════════╪══════════════╪════════════╪══════════════╡
│ g │ 34.3882 │ no │ 1.53536 │
│ b1 │ -0.00749901 │ no │ 0.0401928 │
│ b2 │ 0 │ yes │ Not Reported │
│ b3 │ -7.31351e-07 │ yes │ Not Reported │
│ rho │ 2.3203 │ no │ 1.88621 │
│ alpha │ 0.0192091 │ no │ 0.00243563 │
╘════════════╧══════════════╧════════════╧══════════════╛
Standard errors estimates are not generated for parameters estimated on corresponding bounds,
although sampling error is present for all parameters, as a rule. Standard error estimates may not
be reliable as a basis for confidence intervals or tests when one or more parameters are on bounds.


Goodness of Fit:
╒════════╤═════╤═══════════════╤═════════════════════╤═══════════════════╕
│ Dose │ N │ Sample Mean │ Model Fitted Mean │ Scaled Residual │
╞════════╪═════╪═══════════════╪═════════════════════╪═══════════════════╡
│ 0 │ 9 │ 33.7 │ 34.3882 │ -0.245814 │
│ 1 │ 6 │ 32.8 │ 34.3807 │ -0.461117 │
│ 3 │ 6 │ 37 │ 34.3657 │ 0.768876 │
│ 10 │ 6 │ 33.3 │ 34.3125 │ -0.296037 │
│ 30 │ 6 │ 33.6 │ 34.1435 │ -0.159819 │
│ 62.5 │ 3 │ 33.4 │ 33.7409 │ -0.0718783 │
│ 125 │ 3 │ 35.5 │ 32.0224 │ 0.778996 │
│ 250 │ 3 │ 20.9 │ 21.0861 │ -0.0676817 │
╘════════╧═════╧═══════════════╧═════════════════════╧═══════════════════╛
╒════════╤═════╤═════════════╤═══════════════════╕
│ Dose │ N │ Sample SD │ Model Fitted SD │
╞════════╪═════╪═════════════╪═══════════════════╡
│ 0 │ 9 │ 9 │ 8.39885 │
│ 1 │ 6 │ 10.8 │ 8.39672 │
│ 3 │ 6 │ 13.7 │ 8.39247 │
│ 10 │ 6 │ 6.9 │ 8.3774 │
│ 30 │ 6 │ 3.9 │ 8.32955 │
│ 62.5 │ 3 │ 7.4 │ 8.21573 │
│ 125 │ 3 │ 2.6 │ 7.73226 │
│ 250 │ 3 │ 6.1 │ 4.76199 │
╘════════╧═════╧═════════════╧═══════════════════╛

Likelihoods:
╒═════════╤══════════════════╤════════════╤═════════╕
│ Model │ Log-Likelihood │ # Params │ AIC │
╞═════════╪══════════════════╪════════════╪═════════╡
│ A1 │ -146.879 │ 9 │ 311.758 │
│ A2 │ -138.862 │ 16 │ 309.723 │
│ A3 │ -145.099 │ 10 │ 310.198 │
│ fitted │ -146.885 │ 4 │ 301.77 │
│ reduced │ -150.866 │ 2 │ 305.733 │
╘═════════╧══════════════════╧════════════╧═════════╛

Tests of Mean and Variance Fits:
╒════════╤══════════════════════════════╤═════════════╤═══════════╕
│ Name │ -2 * Log(Likelihood Ratio) │ Test d.f. │ P-Value │
╞════════╪══════════════════════════════╪═════════════╪═══════════╡
│ Test 1 │ 24.0098 │ 14 │ 0.0456977 │
│ Test 2 │ 16.0345 │ 7 │ 0.0248027 │
│ Test 3 │ 12.4752 │ 6 │ 0.0521687 │
│ Test 4 │ 3.57152 │ 6 │ 0.734431 │
╘════════╧══════════════════════════════╧═════════════╧═══════════╛
Test 1: Test the null hypothesis that responses and variances don't differ among dose levels
(A2 vs R). If this test fails to reject the null hypothesis (p-value > 0.05), there may not be
a dose-response.

Test 2: Test the null hypothesis that variances are homogenous (A1 vs A2). If this test fails to
reject the null hypothesis (p-value > 0.05), the simpler constant variance model may be appropriate.

Test 3: Test the null hypothesis that the variances are adequately modeled (A3 vs A2). If this test
fails to reject the null hypothesis (p-value > 0.05), it may be inferred that the variances have
been modeled appropriately.

Test 4: Test the null hypothesis that the model for the mean fits the data (Fitted vs A3). If this
test fails to reject the null hypothesis (p-value > 0.1), the user has support for use of the
selected model.

# Liver wt, Rt, F, GD14-18, Conley et al. (2019)

## Dataset

**Name:** Liver wt, Rt, F, GD14-18, Conley et al. (2019)

| Dose | N | Mean | Std. Dev. |
| --- | --- | --- | --- |
| 0 | 9 | 16.7 | 3 |
| 1 | 6 | 15.8 | 1.7 |
| 3 | 6 | 16.7 | 2.7 |
| 10 | 6 | 16.5 | 1.2 |
| 30 | 6 | 17.2 | 1.2 |
| 62.5 | 3 | 17.8 | 1.2 |
| 125 | 3 | 19.1 | 1 |
| 250 | 3 | 19.6 | 0.5 |
| 500 | 3 | 19.2 | 1 |

Test 1 Dose Response: 0.0011

Test 2 Homogeneity of Variance: 0.0028

Test 3 Variance Model Selection: 0.5021

## Settings

| Setting | Value |
| --- | --- |
| BMR | 10% Relative Deviation |
| Distribution | Normal + Nonconstant variance |
| Adverse Direction | Up (↑) |
| Maximum Polynomial Degree | 3 |
| Confidence Level (one sided) | 0.95 |

## Maximum Likelihood Approach

| Model | BMDL | BMD | BMDU | *P*-Value | AIC | Scaled Residual at Control | Scaled Residual near BMD | Recommendation and Notes |
| --- | --- | --- | --- | --- | --- | --- | --- | --- |
| Exponential 3 | 173.345 | 257.752 | 528.777 | 0.003 | 192.721 | -0.099 | 1.091 | **Questionable** Goodness of fit p-value < 0.1 |
| Exponential 5 | 22.24 | 66.165 | 195.824 | 0.005 | 191.459 | 0.452 | -0.172 | **Questionable** Goodness of fit p-value < 0.1 |
| Hill^ab^ | 15.422 | 34.6 | 90.326 | 0.107 | 184.194 | 1.041 | -0.415 | **Recommended - Lowest AIC** |
| Polynomial 2 | 155.678 | 239.878 | 503.32 | 0.003 | 192.381 | -0.057 | 1.01 | **Questionable** Goodness of fit p-value < 0.1 |
| Polynomial 3 | 155.678 | 239.878 | 503.32 | 0.003 | 192.381 | -0.057 | 1.01 | **Questionable** Goodness of fit p-value < 0.1 |
| Power | 155.678 | 239.878 | 503.32 | 0.003 | 192.381 | -0.057 | 1.01 | **Questionable** Goodness of fit p-value < 0.1 |
| Linear | 155.678 | 239.878 | 503.32 | 0.003 | 192.381 | -0.057 | 1.01 | **Questionable** Goodness of fit p-value < 0.1 |

^a^ BMDS recommended best fitting model

^b^ User selected best fitting model


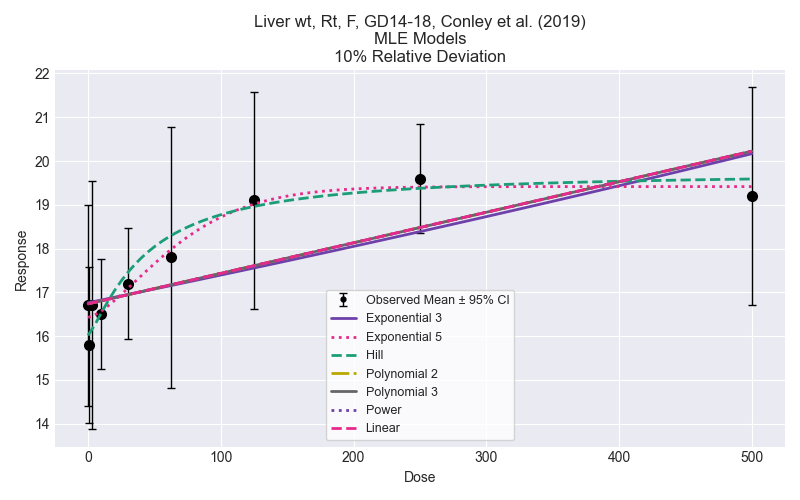


## Selected Model: Hill


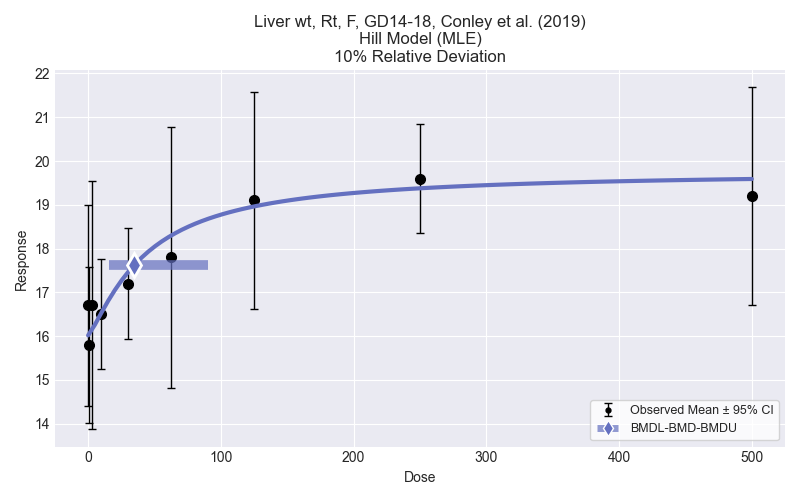


Hill Model
══════════════════════════════

Version: pybmds 25.1 (bmdscore 25.1)

Input Summary:
╒══════════════════════════════╤═══════════════════════════════╕
│ BMR │ 10% Relative Deviation │
│ Distribution │ Normal + Nonconstant variance │
│ Modeling Direction │ Up (↑) │
│ Confidence Level (one sided) │ 0.95 │
│ Modeling Approach │ MLE │
╘══════════════════════════════╧═══════════════════════════════╛

Parameter Settings:
╒═════════════╤═══════════╤═══════╤═══════╕
│ Parameter │ Initial │ Min │ Max │
╞═════════════╪═══════════╪═══════╪═══════╡
│ g │ 0 │ -100 │ 100 │
│ v │ 0 │ -100 │ 100 │
│ k │ 0 │ 0 │ 5 │
│ n │ 1 │ 1 │ 18 │
│ rho │ 0 │ -18 │ 18 │
│ alpha │ 0 │ -18 │ 18 │
╘═════════════╧═══════════╧═══════╧═══════╛

Modeling Summary:
╒════════════════╤════════════╕
│ BMD │ 34.5999 │
│ BMDL │ 15.4216 │
│ BMDU │ 90.3258 │
│ AIC │ 184.194 │
│ Log-Likelihood │ -86.0968 │
│ P-Value │ 0.106723 │
│ Model d.f. │ 5 │
╘════════════════╧════════════╛

Model Parameters:
╒════════════╤════════════╤════════════╤══════════════╕
│ Variable │ Estimate │ On Bound │ Std Error │
╞════════════╪════════════╪════════════╪══════════════╡
│ g │ 16.016 │ no │ 0.531118 │
│ v │ 3.74765 │ no │ 1.07577 │
│ k │ 43.7991 │ no │ 23.5213 │
│ n │ 1.24118 │ no │ 0.954747 │
│ rho │ -6.00022 │ no │ 0.0820279 │
│ alpha │ 6.566e+07 │ no │ 3.36084e+11 │
╘════════════╧════════════╧════════════╧══════════════╛

Goodness of Fit:
╒════════╤═════╤═══════════════╤═════════════════════╤═══════════════════╕
│ Dose │ N │ Sample Mean │ Model Fitted Mean │ Scaled Residual │
╞════════╪═════╪═══════════════╪═════════════════════╪═══════════════════╡
│ 0 │ 9 │ 16.7 │ 16.016 │ 1.04067 │
│ 1 │ 6 │ 15.8 │ 16.0501 │ -0.312676 │
│ 3 │ 6 │ 16.7 │ 16.1458 │ 0.705327 │
│ 10 │ 6 │ 16.5 │ 16.5326 │ -0.0445914 │
│ 30 │ 6 │ 17.2 │ 17.4577 │ -0.414623 │
│ 62.5 │ 3 │ 17.8 │ 18.2967 │ -0.650565 │
│ 125 │ 3 │ 19.1 │ 18.9621 │ 0.201065 │
│ 250 │ 3 │ 19.6 │ 19.3768 │ 0.347154 │
│ 500 │ 3 │ 19.2 │ 19.5897 │ -0.626371 │
╘════════╧═════╧═══════════════╧═════════════════════╧═══════════════════╛
╒════════╤═════╤═════════════╤═══════════════════╕
│ Dose │ N │ Sample SD │ Model Fitted SD │
╞════════╪═════╪═════════════╪═══════════════════╡
│ 0 │ 9 │ 3 │ 1.97175 │
│ 1 │ 6 │ 1.7 │ 1.95922 │
│ 3 │ 6 │ 2.7 │ 1.92457 │
│ 10 │ 6 │ 1.2 │ 1.79262 │
│ 30 │ 6 │ 1.2 │ 1.52247 │
│ 62.5 │ 3 │ 1.2 │ 1.32248 │
│ 125 │ 3 │ 1 │ 1.18809 │
│ 250 │ 3 │ 0.5 │ 1.11342 │
│ 500 │ 3 │ 1 │ 1.07752 │
╘════════╧═════╧═════════════╧═══════════════════╛

Likelihoods:
╒═════════╤══════════════════╤════════════╤═════════╕
│ Model │ Log-Likelihood │ # Params │ AIC │
╞═════════╪══════════════════╪════════════╪═════════╡
│ A1 │ -90.1291 │ 10 │ 200.258 │
│ A2 │ -78.4034 │ 18 │ 192.807 │
│ A3 │ -81.5671 │ 11 │ 185.134 │
│ fitted │ -86.0968 │ 6 │ 184.194 │
│ reduced │ -97.8944 │ 2 │ 199.789 │
╘═════════╧══════════════════╧════════════╧═════════╛

Tests of Mean and Variance Fits:
╒════════╤══════════════════════════════╤═════════════╤════════════╕
│ Name │ -2 * Log(Likelihood Ratio) │ Test d.f. │ P-Value │
╞════════╪══════════════════════════════╪═════════════╪════════════╡
│ Test 1 │ 38.982 │ 16 │ 0.00109414 │
│ Test 2 │ 23.4514 │ 8 │ 0.00283053 │
│ Test 3 │ 6.32744 │ 7 │ 0.502078 │
│ Test 4 │ 9.05927 │ 5 │ 0.106723 │
╘════════╧══════════════════════════════╧═════════════╧════════════╛
Test 1: Test the null hypothesis that responses and variances don't differ among dose levels
(A2 vs R). If this test fails to reject the null hypothesis (p-value > 0.05), there may not be
a dose-response.

Test 2: Test the null hypothesis that variances are homogenous (A1 vs A2). If this test fails to
reject the null hypothesis (p-value > 0.05), the simpler constant variance model may be appropriate.

Test 3: Test the null hypothesis that the variances are adequately modeled (A3 vs A2). If this test
fails to reject the null hypothesis (p-value > 0.05), it may be inferred that the variances have
been modeled appropriately.

Test 4: Test the null hypothesis that the model for the mean fits the data (Fitted vs A3). If this
test fails to reject the null hypothesis (p-value > 0.1), the user has support for use of the
selected model.

# Serum T3, Rt, F, GD14-18, Conley et al. (2019)

## Dataset

**Name:** Serum T3, Rt, F, GD14-18, Conley et al. (2019)

| Dose | N | Mean | Std. Dev. |
| --- | --- | --- | --- |
| 0 | 6 | 0.62 | 0.1 |
| 1 | 3 | 0.67 | 0.12 |
| 3 | 3 | 0.61 | 0.05 |
| 10 | 3 | 0.52 | 0.12 |
| 30 | 3 | 0.45 | 0.1 |
| 62.5 | 3 | 0.38 | 0.1 |
| 125 | 3 | 0.32 | 0.05 |

Test 1 Dose Response: 0.0013

Test 2 Homogeneity of Variance: 0.6199

Test 3 Variance Model Selection: 0.6199

## Settings

| Setting | Value |
| --- | --- |
| BMR | 1.0 Standard Deviation |
| Distribution | Normal + Constant variance |
| Adverse Direction | Down (↓) |
| Maximum Polynomial Degree | 3 |
| Confidence Level (one sided) | 0.95 |

## Maximum Likelihood Approach

| Model | BMDL | BMD | BMDU | *P*-Value | AIC | Scaled Residual at Control | Scaled Residual near BMD | Recommendation and Notes |
| --- | --- | --- | --- | --- | --- | --- | --- | --- |
| Exponential 3 | 16.292 | 25.159 | 42.955 | 0.381 | -41.216 | 0.257 | -0.974 | **Viable** |
| Exponential 5 | 4.308 | 9.934 | 25.566 | 0.803 | -42.879 | -0.425 | -0.624 | **Viable** |
| Hill^ab^ | 3.154 | 8.633 | 24.07 | 0.708 | -41.117 | -0.498 | -0.471 | **Recommended - Lowest BMDL** |
| Polynomial 2 | 26.256 | 36.263 | 58.313 | 0.175 | -38.832 | 0.525 | -1.293 | **Viable** |
| Polynomial 3 | 26.256 | 36.263 | 58.629 | 0.175 | -38.832 | 0.525 | -1.293 | **Viable** |
| Power | 26.257 | 36.262 | 58.003 | 0.175 | -38.832 | 0.525 | -1.293 | **Viable** |
| Linear | 26.255 | 36.262 | 57.848 | 0.175 | -38.832 | 0.525 | -1.293 | **Viable** |

^a^ BMDS recommended best fitting model

^b^ User selected best fitting model


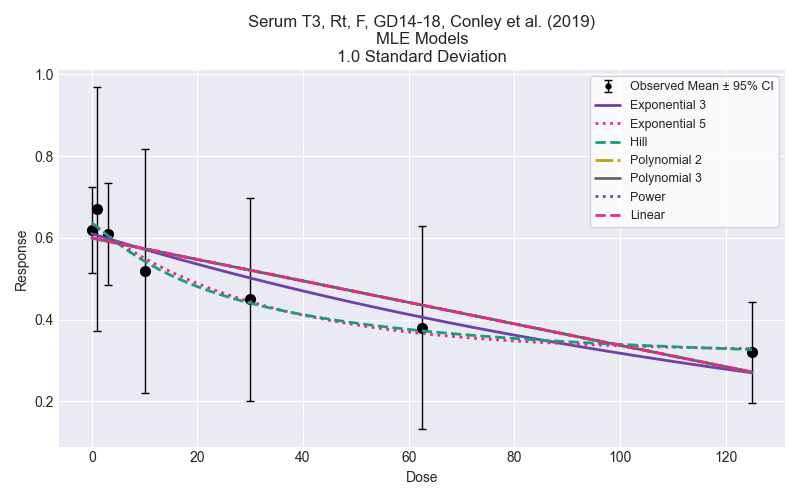


## Selected Model: Hill


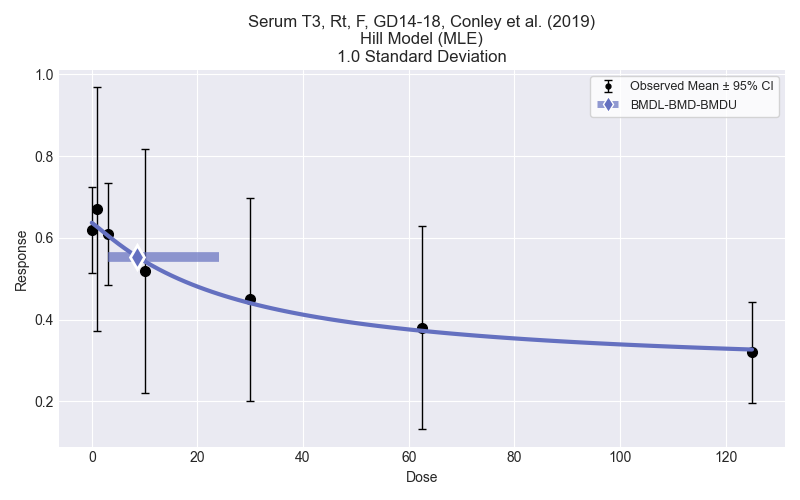


Hill Model
══════════════════════════════

Version: pybmds 25.1 (bmdscore 25.1)

Input Summary:
╒══════════════════════════════╤════════════════════════════╕
│ BMR │ 1.0 Standard Deviation │
│ Distribution │ Normal + Constant variance │
│ Modeling Direction │ Down (↓) │
│ Confidence Level (one sided) │ 0.95 │
│ Modeling Approach │ MLE │
╘══════════════════════════════╧════════════════════════════╛

Parameter Settings:
╒═════════════╤═══════════╤═══════╤═══════╕
│ Parameter │ Initial │ Min │ Max │
╞═════════════╪═══════════╪═══════╪═══════╡
│ g │ 0 │ -100 │ 100 │
│ v │ 0 │ -100 │ 100 │
│ k │ 0 │ 0 │ 5 │
│ n │ 1 │ 1 │ 18 │
│ alpha │ 0 │ -18 │ 18 │
╘═════════════╧═══════════╧═══════╧═══════╛

Modeling Summary:
╒════════════════╤════════════╕
│ BMD │ 8.63343 │
│ BMDL │ 3.15371 │
│ BMDU │ 24.07 │
│ AIC │ -41.1174 │
│ Log-Likelihood │ 25.5587 │
│ P-Value │ 0.707533 │
│ Model d.f. │ 3 │
╘════════════════╧════════════╛

Model Parameters:
╒════════════╤═════════════╤════════════╤══════════════╕
│ Variable │ Estimate │ On Bound │ Std Error │
╞════════════╪═════════════╪════════════╪══════════════╡
│ g │ 0.636949 │ no │ 0.0284373 │
│ v │ -0.365314 │ no │ 0.154129 │
│ k │ 26.1242 │ no │ 26.906 │
│ n │ 1.09975 │ no │ 0.621126 │
│ alpha │ 0.00695864 │ no │ 1.39784e-05 │
╘════════════╧═════════════╧════════════╧══════════════╛

Goodness of Fit:
╒════════╤═════╤═══════════════╤═════════════════════╤═══════════════════╕
│ Dose │ N │ Sample Mean │ Model Fitted Mean │ Scaled Residual │
╞════════╪═════╪═══════════════╪═════════════════════╪═══════════════════╡
│ 0 │ 6 │ 0.62 │ 0.636949 │ -0.497687 │
│ 1 │ 3 │ 0.67 │ 0.627122 │ 0.890299 │
│ 3 │ 3 │ 0.61 │ 0.606007 │ 0.0829135 │
│ 10 │ 3 │ 0.52 │ 0.542675 │ -0.470816 │
│ 30 │ 3 │ 0.45 │ 0.440425 │ 0.198812 │
│ 62.5 │ 3 │ 0.38 │ 0.372833 │ 0.148813 │
│ 125 │ 3 │ 0.32 │ 0.327041 │ -0.146187 │
╘════════╧═════╧═══════════════╧═════════════════════╧═══════════════════╛
╒════════╤═════╤═════════════╤═══════════════════╕
│ Dose │ N │ Sample SD │ Model Fitted SD │
╞════════╪═════╪═════════════╪═══════════════════╡
│ 0 │ 6 │ 0.1 │ 0.0834185 │
│ 1 │ 3 │ 0.12 │ 0.0834185 │
│ 3 │ 3 │ 0.05 │ 0.0834185 │
│ 10 │ 3 │ 0.12 │ 0.0834185 │
│ 30 │ 3 │ 0.1 │ 0.0834185 │
│ 62.5 │ 3 │ 0.1 │ 0.0834185 │
│ 125 │ 3 │ 0.05 │ 0.0834185 │
╘════════╧═════╧═════════════╧═══════════════════╛

Likelihoods:
╒═════════╤══════════════════╤════════════╤══════════╕
│ Model │ Log-Likelihood │ # Params │ AIC │
╞═════════╪══════════════════╪════════════╪══════════╡
│ A1 │ 26.2545 │ 8 │ -36.5089 │
│ A2 │ 28.4649 │ 14 │ -28.9299 │
│ A3 │ 26.2545 │ 8 │ -36.5089 │
│ fitted │ 25.5587 │ 5 │ -41.1174 │
│ reduced │ 12.3385 │ 2 │ -20.6771 │
╘═════════╧══════════════════╧════════════╧══════════╛

Tests of Mean and Variance Fits:
╒════════╤══════════════════════════════╤═════════════╤════════════╕
│ Name │ -2 * Log(Likelihood Ratio) │ Test d.f. │ P-Value │
╞════════╪══════════════════════════════╪═════════════╪════════════╡
│ Test 1 │ 32.2528 │ 12 │ 0.00126475 │
│ Test 2 │ 4.42096 │ 6 │ 0.619906 │
│ Test 3 │ 4.42096 │ 6 │ 0.619906 │
│ Test 4 │ 1.39148 │ 3 │ 0.707533 │
╘════════╧══════════════════════════════╧═════════════╧════════════╛
Test 1: Test the null hypothesis that responses and variances don't differ among dose levels
(A2 vs R). If this test fails to reject the null hypothesis (p-value > 0.05), there may not be
a dose-response.

Test 2: Test the null hypothesis that variances are homogenous (A1 vs A2). If this test fails to
reject the null hypothesis (p-value > 0.05), the simpler constant variance model may be appropriate.

Test 3: Test the null hypothesis that the variances are adequately modeled (A3 vs A2). If this test
fails to reject the null hypothesis (p-value > 0.05), it may be inferred that the variances have
been modeled appropriately.

Test 4: Test the null hypothesis that the model for the mean fits the data (Fitted vs A3). If this
test fails to reject the null hypothesis (p-value > 0.1), the user has support for use of the
selected model.

# Serum T4, Rt, F, GD14-18, Conley et al. (2019)_drop

## Dataset

**Name:** Serum T4, Rt, F, GD14-18, Conley et al. (2019)_drop

| Dose | N | Mean | Std. Dev. |
| --- | --- | --- | --- |
| 0 | 6 | 22.8 | 4.7 |
| 1 | 3 | 27.9 | 4.2 |
| 3 | 3 | 23 | 7.8 |
| 10 | 3 | 17.5 | 1.9 |
| 30 | 3 | 28.4 | 12.5 |
| 62.5 | 3 | 20.3 | 4.2 |
| 125 | 3 | 11.6 | 1.2 |
| 250 | 3 | 8.4 | 3.1 |

Test 1 Dose Response: <0.0001

Test 2 Homogeneity of Variance: 0.1519

Test 3 Variance Model Selection: 0.1519

## Settings

| Setting | Value |
| --- | --- |
| BMR | 1.0 Standard Deviation |
| Distribution | Lognormal + Constant variance |
| Adverse Direction | Down (↓) |
| Maximum Polynomial Degree | 3 |
| Confidence Level (one sided) | 0.95 |

## Maximum Likelihood Approach

| Model | BMDL | BMD | BMDU | *P*-Value | AIC | Scaled Residual at Control | Scaled Residual near BMD | Recommendation and Notes |
| --- | --- | --- | --- | --- | --- | --- | --- | --- |
| Exponential 3 | 43.719 | 84.417 | 86.385 | 0.062 | 168.612 | -0.798 | 2.985 | **Questionable** \|Residual near BMD\| > 2.0 Goodness of fit p-value < 0.1 |
| Exponential 5^ab^ | 49.188 | 93.049 | 95.202 | 0.103 | 167.803 | 0.302 | -0.269 | **Recommended - Lowest AIC** |

^a^ BMDS recommended best fitting model

^b^ User selected best fitting model


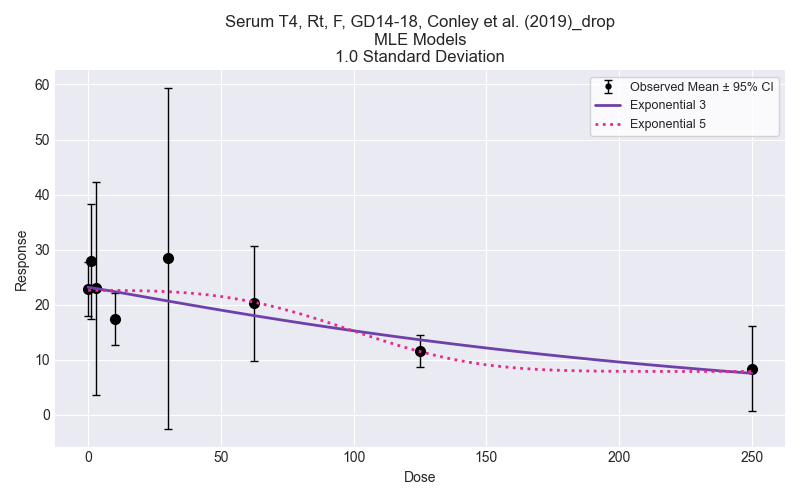


## Selected Model: Exponential 5


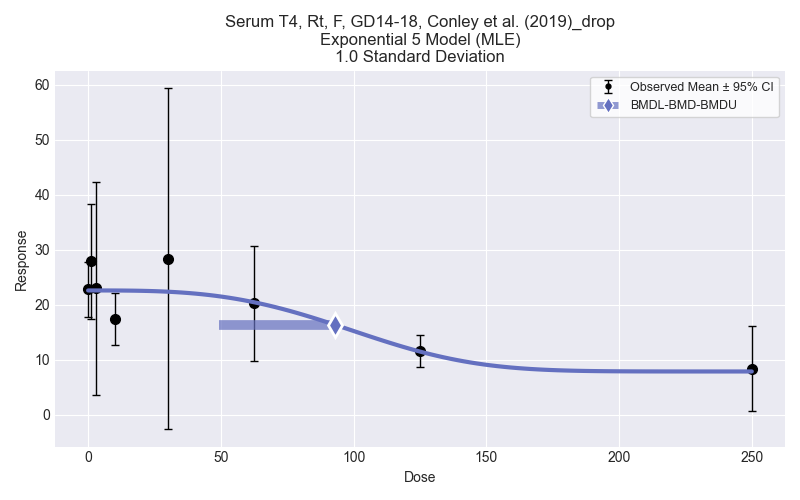


Exponential 5 Model
══════════════════════════════

Version: pybmds 25.1 (bmdscore 25.1)

Input Summary:
╒══════════════════════════════╤═══════════════════════════════╕
│ BMR │ 1.0 Standard Deviation │
│ Distribution │ Lognormal + Constant variance │
│ Modeling Direction │ Down (↓) │
│ Confidence Level (one sided) │ 0.95 │
│ Modeling Approach │ MLE │
╘══════════════════════════════╧═══════════════════════════════╛

Parameter Settings:
╒═════════════╤═══════════╤═══════╤═══════╕
│ Parameter │ Initial │ Min │ Max │
╞═════════════╪═══════════╪═══════╪═══════╡
│ a │ 0 │ 0 │ 100 │
│ b │ 0 │ 0 │ 100 │
│ c │ 0 │ -20 │ 0 │
│ d │ 1 │ 1 │ 18 │
│ log-alpha │ 0 │ -18 │ 18 │
╘═════════════╧═══════════╧═══════╧═══════╛

Modeling Summary:
╒════════════════╤════════════╕
│ BMD │ 93.049 │
│ BMDL │ 49.1885 │
│ BMDU │ 95.2016 │
│ AIC │ 167.803 │
│ Log-Likelihood │ -78.9013 │
│ P-Value │ 0.103051 │
│ Model d.f. │ 4 │
╘════════════════╧════════════╛

Model Parameters:
╒════════════╤═════════════╤════════════╤═════════════╕
│ Variable │ Estimate │ On Bound │ Std Error │
╞════════════╪═════════════╪════════════╪═════════════╡
│ a │ 22.6027 │ no │ 1.3199 │
│ b │ 0.00890643 │ no │ 0.00113237 │
│ c │ 0.348914 │ no │ 0.15256 │
│ d │ 3.15835 │ no │ 1.98886 │
│ log-alpha │ -2.82096 │ no │ 0.272164 │
╘════════════╧═════════════╧════════════╧═════════════╛

Goodness of Fit:
╒════════╤═════╤═══════════════╤═════════════════════════════╤═══════════════════════╤═══════════════════╕
│ Dose │ N │ Sample Mean │ Approximate Sample Median │ Model Fitted Median │ Scaled Residual │
╞════════╪═════╪═══════════════╪═════════════════════════════╪═══════════════════════╪═══════════════════╡
│ 0 │ 6 │ 22.8 │ 22.3305 │ 22.6428 │ 0.301719 │
│ 1 │ 3 │ 27.9 │ 27.5891 │ 22.6428 │ 7.13408 │
│ 3 │ 3 │ 23 │ 21.7815 │ 22.6426 │ 0.484963 │
│ 10 │ 3 │ 17.5 │ 17.3978 │ 22.6357 │ -6.96914 │
│ 30 │ 3 │ 28.4 │ 25.9936 │ 22.4164 │ 8.11984 │
│ 62.5 │ 3 │ 20.3 │ 19.879 │ 20.4982 │ -0.268951 │
│ 125 │ 3 │ 11.6 │ 11.5384 │ 11.523 │ 0.104494 │
│ 250 │ 3 │ 8.4 │ 7.88048 │ 7.90043 │ 0.677919 │
╘════════╧═════╧═══════════════╧═════════════════════════════╧═══════════════════════╧═══════════════════╛
╒════════╤═════╤═════════════╤══════════════════════════╤════════════════════╤═══════════════════╕
│ Dose │ N │ Sample SD │ Approximate Sample GSD │ Model Fitted GSD │ Scaled Residual │
╞════════╪═════╪═════════════╪══════════════════════════╪════════════════════╪═══════════════════╡
│ 0 │ 6 │ 4.7 │ 1.2263 │ 1.27638 │ 0.301719 │
│ 1 │ 3 │ 4.2 │ 1.16148 │ 1.27638 │ 7.13408 │
│ 3 │ 3 │ 7.8 │ 1.39089 │ 1.27638 │ 0.484963 │
│ 10 │ 3 │ 1.9 │ 1.11433 │ 1.27638 │ -6.96914 │
│ 30 │ 3 │ 12.5 │ 1.52319 │ 1.27638 │ 8.11984 │
│ 62.5 │ 3 │ 4.2 │ 1.2272 │ 1.27638 │ -0.268951 │
│ 125 │ 3 │ 1.2 │ 1.10868 │ 1.27638 │ 0.104494 │
│ 250 │ 3 │ 3.1 │ 1.42951 │ 1.27638 │ 0.677919 │
╘════════╧═════╧═════════════╧══════════════════════════╧════════════════════╧═══════════════════╛

Likelihoods:
╒═════════╤══════════════════╤════════════╤═════════╕
│ Model │ Log-Likelihood │ # Params │ AIC │
╞═════════╪══════════════════╪════════════╪═════════╡
│ A1 │ -75.0494 │ 9 │ 168.099 │
│ A2 │ -69.6956 │ 16 │ 171.391 │
│ A3 │ -75.0494 │ 9 │ 168.099 │
│ fitted │ -78.9013 │ 5 │ 167.803 │
│ reduced │ -94.7658 │ 2 │ 193.532 │
╘═════════╧══════════════════╧════════════╧═════════╛

Tests of Mean and Variance Fits:
╒════════╤══════════════════════════════╤═════════════╤═════════════╕
│ Name │ -2 * Log(Likelihood Ratio) │ Test d.f. │ P-Value │
╞════════╪══════════════════════════════╪═════════════╪═════════════╡
│ Test 1 │ 50.1403 │ 14 │ 5.78454e-06 │
│ Test 2 │ 10.7076 │ 7 │ 0.151889 │
│ Test 3 │ 10.7076 │ 7 │ 0.151889 │
│ Test 4 │ 7.70381 │ 4 │ 0.103051 │
╘════════╧══════════════════════════════╧═════════════╧═════════════╛
Test 1: Test the null hypothesis that responses and variances don't differ among dose levels
(A2 vs R). If this test fails to reject the null hypothesis (p-value > 0.05), there may not be
a dose-response.

Test 2: Test the null hypothesis that variances are homogenous (A1 vs A2). If this test fails to
reject the null hypothesis (p-value > 0.05), the simpler constant variance model may be appropriate.

Test 3: Test the null hypothesis that the variances are adequately modeled (A3 vs A2). If this test
fails to reject the null hypothesis (p-value > 0.05), it may be inferred that the variances have
been modeled appropriately.

Test 4: Test the null hypothesis that the model for the mean fits the data (Fitted vs A3). If this
test fails to reject the null hypothesis (p-value > 0.1), the user has support for use of the
selected model.

# Serum cholesterol, Rt, F, GD14-18, Conley et al. (2019)

## Dataset

**Name:** Serum cholesterol, Rt, F, GD14-18, Conley et al. (2019)

| Dose | N | Mean | Std. Dev. |
| --- | --- | --- | --- |
| 0 | 6 | 79.3 | 10 |
| 1 | 3 | 86.3 | 24.1 |
| 3 | 3 | 74.3 | 8.7 |
| 10 | 3 | 69.7 | 6.4 |
| 30 | 3 | 68 | 15.8 |
| 62.5 | 3 | 72 | 8.5 |
| 125 | 3 | 69.3 | 14.5 |
| 250 | 3 | 54.3 | 15.8 |
| 500 | 3 | 42.3 | 15.2 |

Test 1 Dose Response: 0.0155

Test 2 Homogeneity of Variance: 0.4502

Test 3 Variance Model Selection: 0.4502

## Settings

| Setting | Value |
| --- | --- |
| BMR | 1.0 Standard Deviation |
| Distribution | Normal + Constant variance |
| Adverse Direction | Down (↓) |
| Maximum Polynomial Degree | 3 |
| Confidence Level (one sided) | 0.95 |

## Maximum Likelihood Approach

| Model | BMDL | BMD | BMDU | *P*-Value | AIC | Scaled Residual at Control | Scaled Residual near BMD | Recommendation and Notes |
| --- | --- | --- | --- | --- | --- | --- | --- | --- |
| Exponential 3^ab^ | 90.577 | 137.995 | 282.736 | 0.748 | 241.595 | 0.404 | 0.456 | **Recommended - Lowest AIC** |
| Exponential 5 | 54.411 | 125.513 | 280.953 | 0.646 | 243.547 | 0.351 | 0.562 | **Viable** |
| Hill | 42.412 | 123.858 | 282.904 | 0.647 | 243.542 | 0.342 | 0.576 | **Viable** |
| Polynomial 2 | 120.501 | 184.125 | 311.553 | 0.574 | 244.085 | 0.569 | 0.214 | **Viable** |
| Polynomial 3 | 121.379 | 169.373 | 316.416 | 0.705 | 241.951 | 0.528 | 0.249 | **Viable** |
| Power | 121.38 | 169.348 | 301.017 | 0.705 | 241.951 | 0.528 | 0.249 | **Viable** |
| Linear | 121.378 | 169.348 | 277.932 | 0.705 | 241.951 | 0.528 | 0.249 | **Viable** |

^a^ BMDS recommended best fitting model

^b^ User selected best fitting model


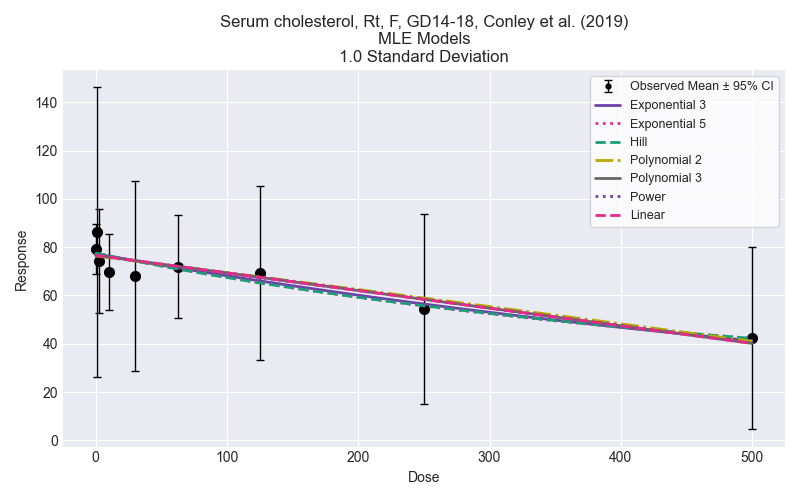


## Selected Model: Exponential 3


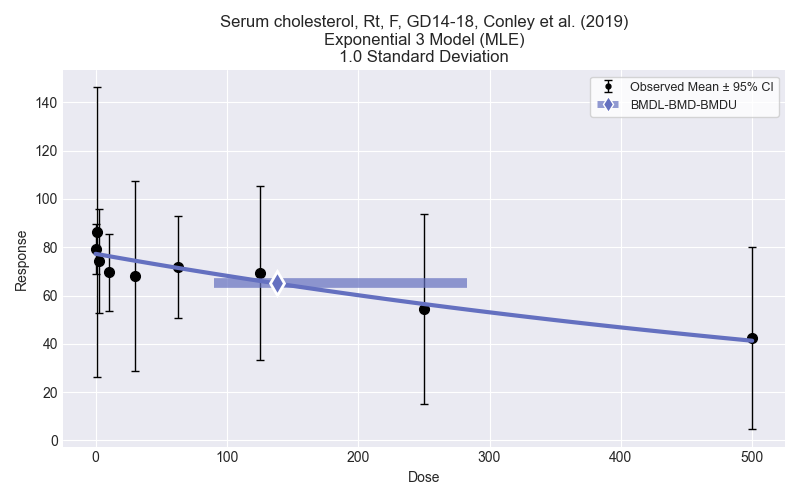


Exponential 3 Model
══════════════════════════════

Version: pybmds 25.1 (bmdscore 25.1)

Input Summary:
╒══════════════════════════════╤════════════════════════════╕
│ BMR │ 1.0 Standard Deviation │
│ Distribution │ Normal + Constant variance │
│ Modeling Direction │ Down (↓) │
│ Confidence Level (one sided) │ 0.95 │
│ Modeling Approach │ MLE │
╘══════════════════════════════╧════════════════════════════╛

Parameter Settings:
╒═════════════╤═══════════╤═══════╤═══════╕
│ Parameter │ Initial │ Min │ Max │
╞═════════════╪═══════════╪═══════╪═══════╡
│ a │ 0 │ 0 │ 100 │
│ b │ 0 │ 0 │ 100 │
│ c │ 0 │ -20 │ 0 │
│ d │ 1 │ 1 │ 18 │
│ log-alpha │ 0 │ -18 │ 18 │
╘═════════════╧═══════════╧═══════╧═══════╛

Modeling Summary:
╒════════════════╤═════════════╕
│ BMD │ 137.995 │
│ BMDL │ 90.5774 │
│ BMDU │ 282.736 │
│ AIC │ 241.595 │
│ Log-Likelihood │ -117.797 │
│ P-Value │ 0.747628 │
│ Model d.f. │ 7 │
╘════════════════╧═════════════╛

Model Parameters:
╒════════════╤═════════════╤════════════╤══════════════╕
│ Variable │ Estimate │ On Bound │ Std Error │
╞════════════╪═════════════╪════════════╪══════════════╡
│ a │ 77.2735 │ no │ 2.77842 │
│ b │ 0.00125368 │ no │ 0.000299629 │
│ d │ 1 │ yes │ Not Reported │
│ log-alpha │ 5.01529 │ no │ 0.258199 │
╘════════════╧═════════════╧════════════╧══════════════╛
Standard errors estimates are not generated for parameters estimated on corresponding bounds,
although sampling error is present for all parameters, as a rule. Standard error estimates may not
be reliable as a basis for confidence intervals or tests when one or more parameters are on bounds.


Goodness of Fit:
╒════════╤═════╤═══════════════╤═════════════════════╤═══════════════════╕
│ Dose │ N │ Sample Mean │ Model Fitted Mean │ Scaled Residual │
╞════════╪═════╪═══════════════╪═════════════════════╪═══════════════════╡
│ 0 │ 6 │ 79.3 │ 77.2735 │ 0.404355 │
│ 1 │ 3 │ 86.3 │ 77.1767 │ 1.28723 │
│ 3 │ 3 │ 74.3 │ 76.9834 │ -0.378613 │
│ 10 │ 3 │ 69.7 │ 76.3108 │ -0.932736 │
│ 30 │ 3 │ 68 │ 74.4212 │ -0.905984 │
│ 62.5 │ 3 │ 72 │ 71.4499 │ 0.0776194 │
│ 125 │ 3 │ 69.3 │ 66.0651 │ 0.456419 │
│ 250 │ 3 │ 54.3 │ 56.4825 │ -0.307931 │
│ 500 │ 3 │ 42.3 │ 41.2854 │ 0.14315 │
╘════════╧═════╧═══════════════╧═════════════════════╧═══════════════════╛
╒════════╤═════╤═════════════╤═══════════════════╕
│ Dose │ N │ Sample SD │ Model Fitted SD │
╞════════╪═════╪═════════════╪═══════════════════╡
│ 0 │ 6 │ 10 │ 12.276 │
│ 1 │ 3 │ 24.1 │ 12.276 │
│ 3 │ 3 │ 8.7 │ 12.276 │
│ 10 │ 3 │ 6.4 │ 12.276 │
│ 30 │ 3 │ 15.8 │ 12.276 │
│ 62.5 │ 3 │ 8.5 │ 12.276 │
│ 125 │ 3 │ 14.5 │ 12.276 │
│ 250 │ 3 │ 15.8 │ 12.276 │
│ 500 │ 3 │ 15.2 │ 12.276 │
╘════════╧═════╧═════════════╧═══════════════════╛

Likelihoods:
╒═════════╤══════════════════╤════════════╤═════════╕
│ Model │ Log-Likelihood │ # Params │ AIC │
╞═════════╪══════════════════╪════════════╪═════════╡
│ A1 │ -115.66 │ 10 │ 251.32 │
│ A2 │ -111.745 │ 18 │ 259.49 │
│ A3 │ -115.66 │ 10 │ 251.32 │
│ fitted │ -117.797 │ 3 │ 241.595 │
│ reduced │ -127.002 │ 2 │ 258.005 │
╘═════════╧══════════════════╧════════════╧═════════╛

Tests of Mean and Variance Fits:
╒════════╤══════════════════════════════╤═════════════╤═══════════╕
│ Name │ -2 * Log(Likelihood Ratio) │ Test d.f. │ P-Value │
╞════════╪══════════════════════════════╪═════════════╪═══════════╡
│ Test 1 │ 30.5145 │ 16 │ 0.0155102 │
│ Test 2 │ 7.83006 │ 8 │ 0.450244 │
│ Test 3 │ 7.83006 │ 8 │ 0.450244 │
│ Test 4 │ 4.27488 │ 7 │ 0.747628 │
╘════════╧══════════════════════════════╧═════════════╧═══════════╛
Test 1: Test the null hypothesis that responses and variances don't differ among dose levels
(A2 vs R). If this test fails to reject the null hypothesis (p-value > 0.05), there may not be
a dose-response.

Test 2: Test the null hypothesis that variances are homogenous (A1 vs A2). If this test fails to
reject the null hypothesis (p-value > 0.05), the simpler constant variance model may be appropriate.

Test 3: Test the null hypothesis that the variances are adequately modeled (A3 vs A2). If this test
fails to reject the null hypothesis (p-value > 0.05), it may be inferred that the variances have
been modeled appropriately.

Test 4: Test the null hypothesis that the model for the mean fits the data (Fitted vs A3). If this
test fails to reject the null hypothesis (p-value > 0.1), the user has support for use of the
selected model.

# Serum HDL, Rt, F, GD14-18, Conley et al. (2019)

## Dataset

**Name:** Serum HDL, Rt, F, GD14-18, Conley et al. (2019)

| Dose | N | Mean | Std. Dev. |
| --- | --- | --- | --- |
| 0 | 6 | 37.3 | 4.2 |
| 1 | 3 | 36.3 | 6.4 |
| 3 | 3 | 36 | 4.3 |
| 10 | 3 | 36 | 4.3 |
| 30 | 3 | 33 | 6.2 |
| 62.5 | 3 | 37 | 2.1 |
| 125 | 3 | 36.7 | 8.1 |
| 250 | 3 | 28.7 | 5.5 |
| 500 | 3 | 19.3 | 4.7 |

Test 1 Dose Response: 0.0056

Test 2 Homogeneity of Variance: 0.6924

Test 3 Variance Model Selection: 0.6924

## Settings

| Setting | Value |
| --- | --- |
| BMR | 1.0 Standard Deviation |
| Distribution | Normal + Constant variance |
| Adverse Direction | Down (↓) |
| Maximum Polynomial Degree | 3 |
| Confidence Level (one sided) | 0.95 |

## Maximum Likelihood Approach

| Model | BMDL | BMD | BMDU | *P*-Value | AIC | Scaled Residual at Control | Scaled Residual near BMD | Recommendation and Notes |
| --- | --- | --- | --- | --- | --- | --- | --- | --- |
| Exponential 3 | 111.235 | 207.571 | 328.993 | 0.787 | 184.411 | 0.489 | -0.552 | **Viable** |
| Exponential 5^ab^ | 131.795 | 241.269 | 297.733 | 0.911 | 183.336 | 0.599 | -0. | **Recommended - Lowest AIC** |
| Hill | 224.598 | 239.281 | 259.558 | 0.836 | 185.336 | 0.599 | -0. | **Viable** |
| Polynomial 2 | 110.885 | 210.882 | 310.698 | 0.751 | 184.686 | 0.441 | -0.707 | **Viable** |
| Polynomial 3 | 110.891 | 204.027 | 208.25 | 0.751 | 184.687 | 0.41 | -0.645 | **Viable** |
| Power | 111.633 | 207.292 | 339.723 | 0.764 | 184.589 | 0.465 | -0.616 | **Viable** |
| Linear | 103.913 | 140.184 | 213.533 | 0.709 | 183.838 | 0.135 | 1.418 | **Viable** |

^a^ BMDS recommended best fitting model

^b^ User selected best fitting model


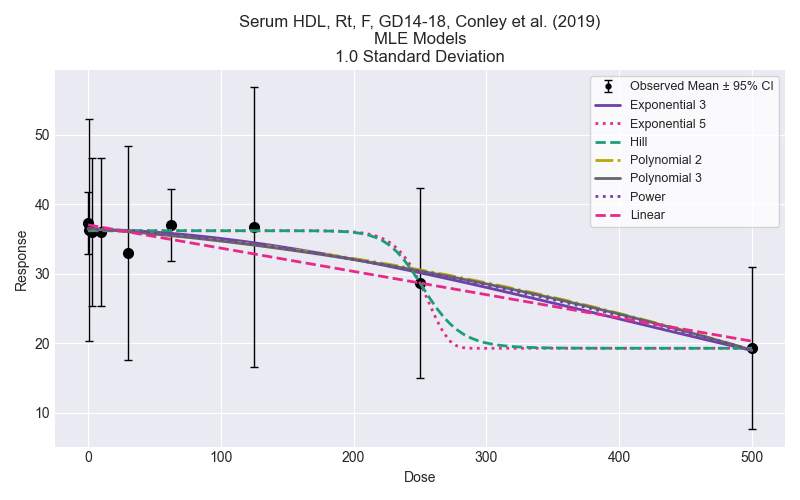


## Selected Model: Exponential 5


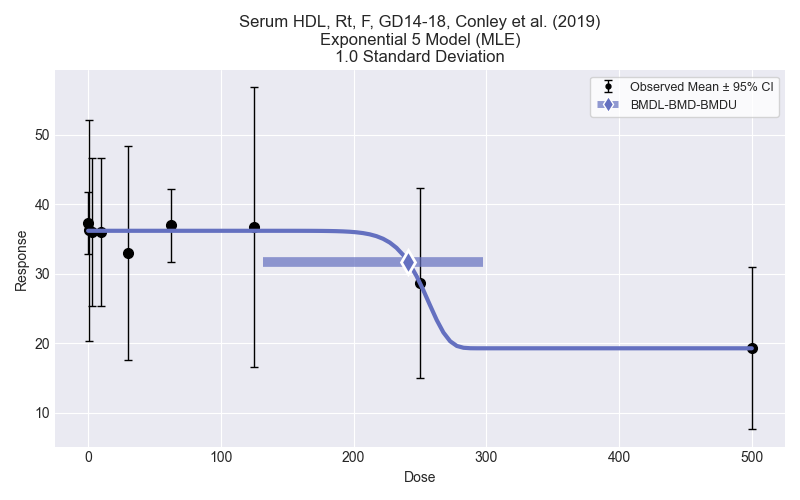


Exponential 5 Model
══════════════════════════════

Version: pybmds 25.1 (bmdscore 25.1)

Input Summary:
╒══════════════════════════════╤════════════════════════════╕
│ BMR │ 1.0 Standard Deviation │
│ Distribution │ Normal + Constant variance │
│ Modeling Direction │ Down (↓) │
│ Confidence Level (one sided) │ 0.95 │
│ Modeling Approach │ MLE │
╘══════════════════════════════╧════════════════════════════╛

Parameter Settings:
╒═════════════╤═══════════╤═══════╤═══════╕
│ Parameter │ Initial │ Min │ Max │
╞═════════════╪═══════════╪═══════╪═══════╡
│ a │ 0 │ 0 │ 100 │
│ b │ 0 │ 0 │ 100 │
│ c │ 0 │ -20 │ 0 │
│ d │ 1 │ 1 │ 18 │
│ log-alpha │ 0 │ -18 │ 18 │
╘═════════════╧═══════════╧═══════╧═══════╛

Modeling Summary:
╒════════════════╤════════════╕
│ BMD │ 241.269 │
│ BMDL │ 131.795 │
│ BMDU │ 297.733 │
│ AIC │ 183.336 │
│ Log-Likelihood │ -87.6682 │
│ P-Value │ 0.910734 │
│ Model d.f. │ 6 │
╘════════════════╧════════════╛

Model Parameters:
╒════════════╤════════════╤════════════╤══════════════╕
│ Variable │ Estimate │ On Bound │ Std Error │
╞════════════╪════════════╪════════════╪══════════════╡
│ a │ 36.2 │ no │ 0.917876 │
│ b │ 0.0038832 │ no │ 0.000112905 │
│ c │ 0.533149 │ no │ 0.136877 │
│ d │ 18 │ yes │ Not Reported │
│ log-alpha │ 3.00667 │ no │ 0.258198 │
╘════════════╧════════════╧════════════╧══════════════╛
Standard errors estimates are not generated for parameters estimated on corresponding bounds,
although sampling error is present for all parameters, as a rule. Standard error estimates may not
be reliable as a basis for confidence intervals or tests when one or more parameters are on bounds.


Goodness of Fit:
╒════════╤═════╤═══════════════╤═════════════════════╤═══════════════════╕
│ Dose │ N │ Sample Mean │ Model Fitted Mean │ Scaled Residual │
╞════════╪═════╪═══════════════╪═════════════════════╪═══════════════════╡
│ 0 │ 6 │ 37.3 │ 36.2 │ 0.599206 │
│ 1 │ 3 │ 36.3 │ 36.2 │ 0.0385169 │
│ 3 │ 3 │ 36 │ 36.2 │ -0.0770388 │
│ 10 │ 3 │ 36 │ 36.2 │ -0.0770388 │
│ 30 │ 3 │ 33 │ 36.2 │ -1.2326 │
│ 62.5 │ 3 │ 37 │ 36.2 │ 0.308147 │
│ 125 │ 3 │ 36.7 │ 36.2 │ 0.192606 │
│ 250 │ 3 │ 28.7 │ 28.7 │ -1.32368e-06 │
│ 500 │ 3 │ 19.3 │ 19.3 │ 2.83989e-07 │
╘════════╧═════╧═══════════════╧═════════════════════╧═══════════════════╛
╒════════╤═════╤═════════════╤═══════════════════╕
│ Dose │ N │ Sample SD │ Model Fitted SD │
╞════════╪═════╪═════════════╪═══════════════════╡
│ 0 │ 6 │ 4.2 │ 4.49667 │
│ 1 │ 3 │ 6.4 │ 4.49667 │
│ 3 │ 3 │ 4.3 │ 4.49667 │
│ 10 │ 3 │ 4.3 │ 4.49667 │
│ 30 │ 3 │ 6.2 │ 4.49667 │
│ 62.5 │ 3 │ 2.1 │ 4.49667 │
│ 125 │ 3 │ 8.1 │ 4.49667 │
│ 250 │ 3 │ 5.5 │ 4.49667 │
│ 500 │ 3 │ 4.7 │ 4.49667 │
╘════════╧═════╧═════════════╧═══════════════════╛

Likelihoods:
╒═════════╤══════════════════╤════════════╤═════════╕
│ Model │ Log-Likelihood │ # Params │ AIC │
╞═════════╪══════════════════╪════════════╪═════════╡
│ A1 │ -86.6206 │ 10 │ 193.241 │
│ A2 │ -83.8228 │ 18 │ 203.646 │
│ A3 │ -86.6206 │ 10 │ 193.241 │
│ fitted │ -87.6682 │ 4 │ 183.336 │
│ reduced │ -100.777 │ 2 │ 205.554 │
╘═════════╧══════════════════╧════════════╧═════════╛

Tests of Mean and Variance Fits:
╒════════╤══════════════════════════════╤═════════════╤════════════╕
│ Name │ -2 * Log(Likelihood Ratio) │ Test d.f. │ P-Value │
╞════════╪══════════════════════════════╪═════════════╪════════════╡
│ Test 1 │ 33.9082 │ 16 │ 0.00558991 │
│ Test 2 │ 5.59563 │ 8 │ 0.692423 │
│ Test 3 │ 5.59563 │ 8 │ 0.692423 │
│ Test 4 │ 2.09524 │ 6 │ 0.910734 │
╘════════╧══════════════════════════════╧═════════════╧════════════╛
Test 1: Test the null hypothesis that responses and variances don't differ among dose levels
(A2 vs R). If this test fails to reject the null hypothesis (p-value > 0.05), there may not be
a dose-response.

Test 2: Test the null hypothesis that variances are homogenous (A1 vs A2). If this test fails to
reject the null hypothesis (p-value > 0.05), the simpler constant variance model may be appropriate.

Test 3: Test the null hypothesis that the variances are adequately modeled (A3 vs A2). If this test
fails to reject the null hypothesis (p-value > 0.05), it may be inferred that the variances have
been modeled appropriately.

Test 4: Test the null hypothesis that the model for the mean fits the data (Fitted vs A3). If this
test fails to reject the null hypothesis (p-value > 0.1), the user has support for use of the
selected model.

# Serum triglycerides, Rt, F, GD16-20, Conley et al. (2021)

## Dataset

**Name:** Serum triglycerides, Rt, F, GD16-20, Conley et al. (2021)

| Dose | N | Mean | Std. Dev. |
| --- | --- | --- | --- |
| 0 | 6 | 390 | 157 |
| 1 | 4 | 268 | 102 |
| 3 | 4 | 256 | 74 |
| 10 | 4 | 197 | 114 |
| 30 | 4 | 181 | 40 |
| 62.5 | 4 | 196 | 54 |
| 125 | 4 | 165 | 30 |

Test 1 Dose Response: 0.0004

Test 2 Homogeneity of Variance: 0.0086

Test 3 Variance Model Selection: 0.5513

## Settings

| Setting | Value |
| --- | --- |
| BMR | 1.0 Standard Deviation |
| Distribution | Normal + Nonconstant variance |
| Adverse Direction | Down (↓) |
| Maximum Polynomial Degree | 3 |
| Confidence Level (one sided) | 0.95 |

## Maximum Likelihood Approach

| Model | BMDL | BMD | BMDU | *P*-Value | AIC | Scaled Residual at Control | Scaled Residual near BMD | Recommendation and Notes |
| --- | --- | --- | --- | --- | --- | --- | --- | --- |
| Exponential 3 | 66.408 | 97.649 | 99.67 | 0.011 | 362.645 | 2.126 | 0.943 | **Questionable** Residual at control > 2.0 Goodness of fit p-value < 0.1 |
| Exponential 5 | 0.946 | 20.567 | - | 0.244 | 355.208 | 1.083 | 0.07 | **Questionable** BMD/BMDL ratio > 3.0 BMD/BMDL ratio > 20.0 |
| Hill^ab^ | 0.662 | 5.872 | - | 0.421 | 353.646 | 0.407 | 0.125 | **Recommended - Lowest AIC** BMD/BMDL ratio > 3.0 |
| Polynomial 2 | 92.676 | 126.716 | 129.339 | 0.014 | 362.08 | 2.302 | 0.633 | **Questionable** Residual at control > 2.0 Goodness of fit p-value < 0.1 BMD/highest dose ratio > 1.0 |
| Polynomial 3 | 110.052 | 127.544 | 186.466 | 0.014 | 362.027 | 2.307 | 0.621 | **Questionable** Residual at control > 2.0 Goodness of fit p-value < 0.1 BMD/highest dose ratio > 1.0 |
| Power | 92.981 | 127.409 | 160.963 | 0.014 | 362.03 | 2.303 | 0.619 | **Questionable** Residual at control > 2.0 Goodness of fit p-value < 0.1 BMD/highest dose ratio > 1.0 |
| Linear | 92.996 | 127.544 | 186.466 | 0.014 | 362.027 | 2.307 | 0.621 | **Questionable** Residual at control > 2.0 Goodness of fit p-value < 0.1 BMD/highest dose ratio > 1.0 |

^a^ BMDS recommended best fitting model

^b^ User selected best fitting model


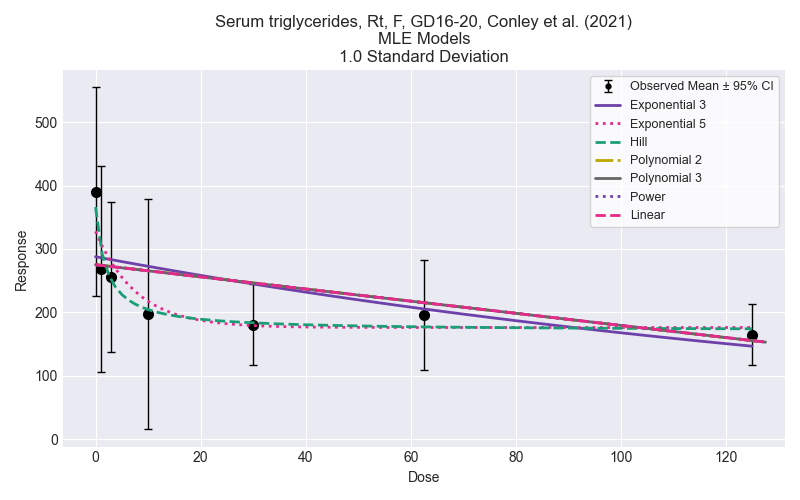


## Selected Model: Hill


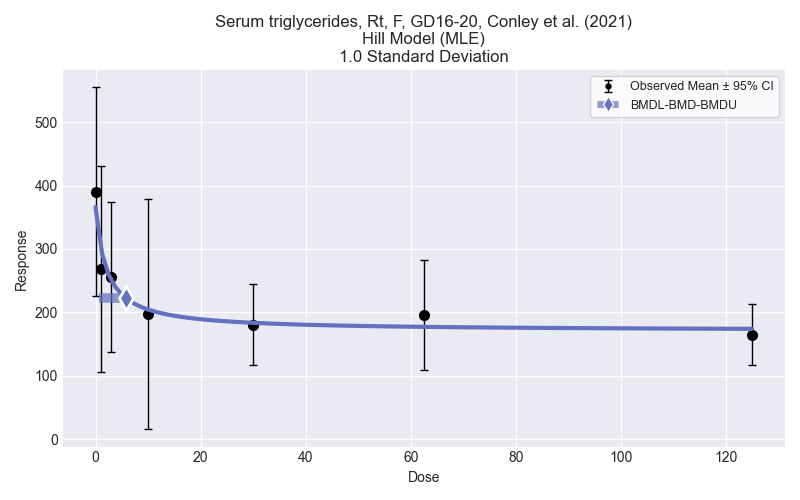


Hill Model
══════════════════════════════

Version: pybmds 25.1 (bmdscore 25.1)

Input Summary:
╒══════════════════════════════╤═══════════════════════════════╕
│ BMR │ 1.0 Standard Deviation │
│ Distribution │ Normal + Nonconstant variance │
│ Modeling Direction │ Down (↓) │
│ Confidence Level (one sided) │ 0.95 │
│ Modeling Approach │ MLE │
╘══════════════════════════════╧═══════════════════════════════╛

Parameter Settings:
╒═════════════╤═══════════╤═══════╤═══════╕
│ Parameter │ Initial │ Min │ Max │
╞═════════════╪═══════════╪═══════╪═══════╡
│ g │ 0 │ -100 │ 100 │
│ v │ 0 │ -100 │ 100 │
│ k │ 0 │ 0 │ 5 │
│ n │ 1 │ 1 │ 18 │
│ rho │ 0 │ -18 │ 18 │
│ alpha │ 0 │ -18 │ 18 │
╘═════════════╧═══════════╧═══════╧═══════╛

Modeling Summary:
╒════════════════╤══════════════╕
│ BMD │ 5.87209 │
│ BMDL │ 0.661727 │
│ BMDU │ -9999 │
│ AIC │ 353.646 │
│ Log-Likelihood │ -171.823 │
│ P-Value │ 0.420919 │
│ Model d.f. │ 4 │
╘════════════════╧══════════════╛

Model Parameters:
╒════════════╤════════════════╤════════════╤══════════════╕
│ Variable │ Estimate │ On Bound │ Std Error │
╞════════════╪════════════════╪════════════╪══════════════╡
│ g │ 366.089 │ no │ 63.4142 │
│ v │ -195.069 │ no │ 60.9608 │
│ k │ 2.08007 │ no │ 2.43521 │
│ n │ 1 │ yes │ Not Reported │
│ rho │ 3.00111 │ no │ 1.41508 │
│ alpha │ 0.000420135 │ no │ 1.36095e-06 │
╘════════════╧════════════════╧════════════╧══════════════╛
Standard errors estimates are not generated for parameters estimated on corresponding bounds,
although sampling error is present for all parameters, as a rule. Standard error estimates may not
be reliable as a basis for confidence intervals or tests when one or more parameters are on bounds.


Goodness of Fit:
╒════════╤═════╤═══════════════╤═════════════════════╤═══════════════════╕
│ Dose │ N │ Sample Mean │ Model Fitted Mean │ Scaled Residual │
╞════════╪═════╪═══════════════╪═════════════════════╪═══════════════════╡
│ 0 │ 6 │ 390 │ 366.089 │ 0.406613 │
│ 1 │ 4 │ 268 │ 302.756 │ -0.641727 │
│ 3 │ 4 │ 256 │ 250.892 │ 0.125033 │
│ 10 │ 4 │ 197 │ 204.609 │ -0.252913 │
│ 30 │ 4 │ 181 │ 183.668 │ -0.104277 │
│ 62.5 │ 4 │ 196 │ 177.303 │ 0.770544 │
│ 125 │ 4 │ 165 │ 174.212 │ -0.389807 │
╘════════╧═════╧═══════════════╧═════════════════════╧═══════════════════╛
╒════════╤═════╤═════════════╤═══════════════════╕
│ Dose │ N │ Sample SD │ Model Fitted SD │
╞════════╪═════╪═════════════╪═══════════════════╡
│ 0 │ 6 │ 157 │ 144.044 │
│ 1 │ 4 │ 102 │ 108.32 │
│ 3 │ 4 │ 74 │ 81.7064 │
│ 10 │ 4 │ 114 │ 60.1675 │
│ 30 │ 4 │ 40 │ 51.1682 │
│ 62.5 │ 4 │ 54 │ 48.5305 │
│ 125 │ 4 │ 30 │ 47.2669 │
╘════════╧═════╧═════════════╧═══════════════════╛

Likelihoods:
╒═════════╤══════════════════╤════════════╤═════════╕
│ Model │ Log-Likelihood │ # Params │ AIC │
╞═════════╪══════════════════╪════════════╪═════════╡
│ A1 │ -176.483 │ 8 │ 368.965 │
│ A2 │ -167.884 │ 14 │ 363.767 │
│ A3 │ -169.877 │ 9 │ 357.754 │
│ fitted │ -171.823 │ 5 │ 353.646 │
│ reduced │ -185.613 │ 2 │ 375.226 │
╘═════════╧══════════════════╧════════════╧═════════╛

Tests of Mean and Variance Fits:
╒════════╤══════════════════════════════╤═════════════╤═════════════╕
│ Name │ -2 * Log(Likelihood Ratio) │ Test d.f. │ P-Value │
╞════════╪══════════════════════════════╪═════════════╪═════════════╡
│ Test 1 │ 35.4586 │ 12 │ 0.000395685 │
│ Test 2 │ 17.1978 │ 6 │ 0.00858315 │
│ Test 3 │ 3.98696 │ 5 │ 0.551296 │
│ Test 4 │ 3.89128 │ 4 │ 0.420919 │
╘════════╧══════════════════════════════╧═════════════╧═════════════╛
Test 1: Test the null hypothesis that responses and variances don't differ among dose levels
(A2 vs R). If this test fails to reject the null hypothesis (p-value > 0.05), there may not be
a dose-response.

Test 2: Test the null hypothesis that variances are homogenous (A1 vs A2). If this test fails to
reject the null hypothesis (p-value > 0.05), the simpler constant variance model may be appropriate.

Test 3: Test the null hypothesis that the variances are adequately modeled (A3 vs A2). If this test
fails to reject the null hypothesis (p-value > 0.05), it may be inferred that the variances have
been modeled appropriately.

Test 4: Test the null hypothesis that the model for the mean fits the data (Fitted vs A3). If this
test fails to reject the null hypothesis (p-value > 0.1), the user has support for use of the
selected model.

# Serum cholesterol, Rt, F, GD16-20, Conley et al. (2021)_drop

## Dataset

**Name:** Serum cholesterol, Rt, F, GD16-20, Conley et al. (2021)_drop

| Dose | N | Mean | Std. Dev. |
| --- | --- | --- | --- |
| 0 | 6 | 94 | 12 |
| 1 | 4 | 89 | 20 |
| 3 | 4 | 81 | 4 |
| 10 | 4 | 87 | 6 |
| 30 | 4 | 72 | 10 |
| 62.5 | 4 | 69 | 10 |

Test 1 Dose Response: 0.0026

Test 2 Homogeneity of Variance: 0.0461

Test 3 Variance Model Selection: 0.0508

## Settings

| Setting | Value |
| --- | --- |
| BMR | 1.0 Standard Deviation |
| Distribution | Normal + Nonconstant variance |
| Adverse Direction | Down (↓) |
| Maximum Polynomial Degree | 3 |
| Confidence Level (one sided) | 0.95 |

## Maximum Likelihood Approach

| Model | BMDL | BMD | BMDU | *P*-Value | AIC | Scaled Residual at Control | Scaled Residual near BMD | Recommendation and Notes |
| --- | --- | --- | --- | --- | --- | --- | --- | --- |
| Exponential 3 | 18.259 | 31.519 | 63.123 | 0.243 | 205.905 | 1.049 | -1.097 | **Viable** |
| Exponential 5 | 4.718 | 14.611 | 48.407 | 0.325 | 205.901 | 0.538 | 0.98 | **Viable** BMD/BMDL ratio > 3.0 |
| Hill^ab^ | 2.046 | 13.501 | 48.365 | 0.326 | 205.896 | 0.465 | 1.097 | **Recommended - Lowest BMDL** BMD/BMDL ratio > 3.0 |
| Polynomial 2 | 21.228 | 34.345 | 65.361 | 0.21 | 206.289 | 1.116 | -1.201 | **Viable** |
| Polynomial 3 | 21.21 | 33.924 | 34.627 | 0.21 | 206.298 | 1.116 | -1.189 | **Viable** |
| Power | 21.222 | 34.091 | 34.797 | 0.21 | 206.293 | 1.122 | -1.218 | **Viable** |
| Linear | 21.227 | 34.569 | 65.353 | 0.21 | 206.289 | 1.12 | -1.205 | **Viable** |

^a^ BMDS recommended best fitting model

^b^ User selected best fitting model


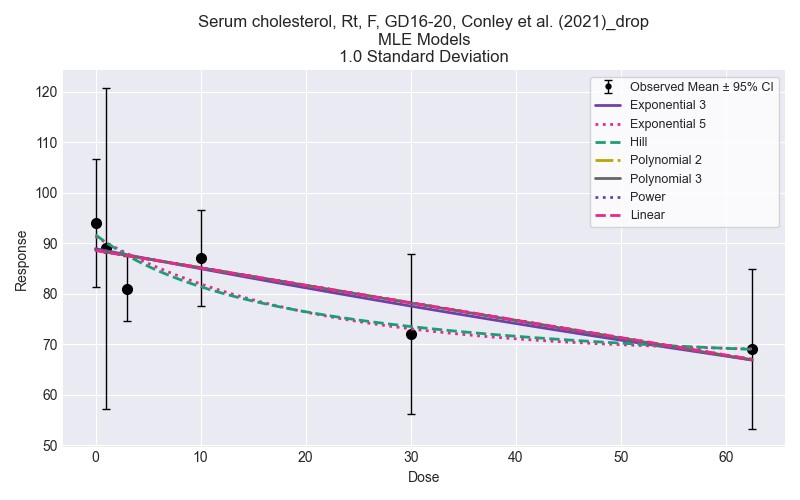


## Selected Model: Hill


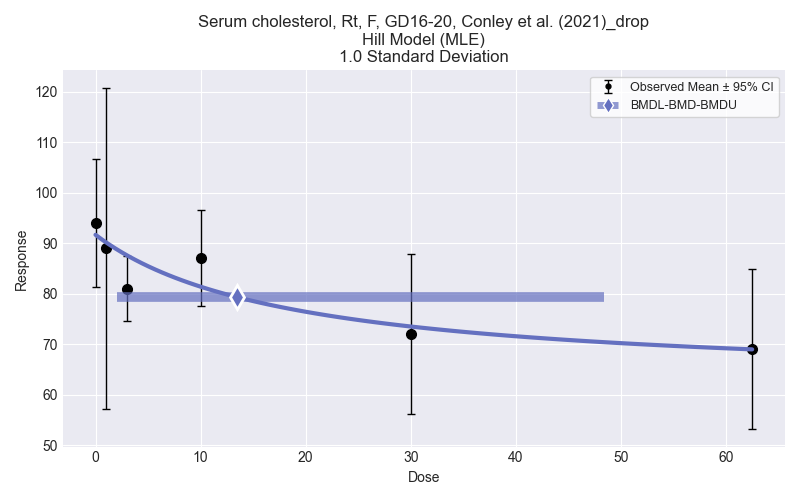


Hill Model
══════════════════════════════

Version: pybmds 25.1 (bmdscore 25.1)

Input Summary:
╒══════════════════════════════╤═══════════════════════════════╕
│ BMR │ 1.0 Standard Deviation │
│ Distribution │ Normal + Nonconstant variance │
│ Modeling Direction │ Down (↓) │
│ Confidence Level (one sided) │ 0.95 │
│ Modeling Approach │ MLE │
╘══════════════════════════════╧═══════════════════════════════╛

Parameter Settings:
╒═════════════╤═══════════╤═══════╤═══════╕
│ Parameter │ Initial │ Min │ Max │
╞═════════════╪═══════════╪═══════╪═══════╡
│ g │ 0 │ -100 │ 100 │
│ v │ 0 │ -100 │ 100 │
│ k │ 0 │ 0 │ 5 │
│ n │ 1 │ 1 │ 18 │
│ rho │ 0 │ -18 │ 18 │
│ alpha │ 0 │ -18 │ 18 │
╘═════════════╧═══════════╧═══════╧═══════╛

Modeling Summary:
╒════════════════╤════════════╕
│ BMD │ 13.5006 │
│ BMDL │ 2.04579 │
│ BMDU │ 48.3653 │
│ AIC │ 205.896 │
│ Log-Likelihood │ -97.9482 │
│ P-Value │ 0.326036 │
│ Model d.f. │ 3 │
╘════════════════╧════════════╛

Model Parameters:
╒════════════╤═══════════════╤════════════╤══════════════╕
│ Variable │ Estimate │ On Bound │ Std Error │
╞════════════╪═══════════════╪════════════╪══════════════╡
│ g │ 91.6532 │ no │ 3.90357 │
│ v │ -29.4858 │ no │ 10.5383 │
│ k │ 18.6866 │ no │ 22.7646 │
│ n │ 1 │ yes │ Not Reported │
│ rho │ 3.14841 │ no │ 2.53826 │
│ alpha │ 0.000101605 │ no │ 1.15642e-07 │
╘════════════╧═══════════════╧════════════╧══════════════╛
Standard errors estimates are not generated for parameters estimated on corresponding bounds,
although sampling error is present for all parameters, as a rule. Standard error estimates may not
be reliable as a basis for confidence intervals or tests when one or more parameters are on bounds.


Goodness of Fit:
╒════════╤═════╤═══════════════╤═════════════════════╤═══════════════════╕
│ Dose │ N │ Sample Mean │ Model Fitted Mean │ Scaled Residual │
╞════════╪═════╪═══════════════╪═════════════════════╪═══════════════════╡
│ 0 │ 6 │ 94 │ 91.6532 │ 0.46481 │
│ 1 │ 4 │ 89 │ 90.1554 │ -0.191755 │
│ 3 │ 4 │ 81 │ 87.5743 │ -1.14214 │
│ 10 │ 4 │ 87 │ 81.3746 │ 1.09704 │
│ 30 │ 4 │ 72 │ 73.4844 │ -0.339903 │
│ 62.5 │ 4 │ 69 │ 68.9541 │ 0.0116273 │
╘════════╧═════╧═══════════════╧═════════════════════╧═══════════════════╛
╒════════╤═════╤═════════════╤═══════════════════╕
│ Dose │ N │ Sample SD │ Model Fitted SD │
╞════════╪═════╪═════════════╪═══════════════════╡
│ 0 │ 6 │ 12 │ 12.3675 │
│ 1 │ 4 │ 20 │ 12.0509 │
│ 3 │ 4 │ 4 │ 11.5122 │
│ 10 │ 4 │ 6 │ 10.2556 │
│ 30 │ 4 │ 10 │ 8.73442 │
│ 62.5 │ 4 │ 10 │ 7.90188 │
╘════════╧═════╧═════════════╧═══════════════════╛

Likelihoods:
╒═════════╤══════════════════╤════════════╤═════════╕
│ Model │ Log-Likelihood │ # Params │ AIC │
╞═════════╪══════════════════╪════════════╪═════════╡
│ A1 │ -97.1342 │ 7 │ 208.268 │
│ A2 │ -91.4939 │ 12 │ 206.988 │
│ A3 │ -96.2184 │ 8 │ 208.437 │
│ fitted │ -97.9482 │ 5 │ 205.896 │
│ reduced │ -104.982 │ 2 │ 213.963 │
╘═════════╧══════════════════╧════════════╧═════════╛

Tests of Mean and Variance Fits:
╒════════╤══════════════════════════════╤═════════════╤════════════╕
│ Name │ -2 * Log(Likelihood Ratio) │ Test d.f. │ P-Value │
╞════════╪══════════════════════════════╪═════════════╪════════════╡
│ Test 1 │ 26.9756 │ 10 │ 0.00262758 │
│ Test 2 │ 11.2806 │ 5 │ 0.0460923 │
│ Test 3 │ 9.4491 │ 4 │ 0.0508037 │
│ Test 4 │ 3.45963 │ 3 │ 0.326036 │
╘════════╧══════════════════════════════╧═════════════╧════════════╛
Test 1: Test the null hypothesis that responses and variances don't differ among dose levels
(A2 vs R). If this test fails to reject the null hypothesis (p-value > 0.05), there may not be
a dose-response.

Test 2: Test the null hypothesis that variances are homogenous (A1 vs A2). If this test fails to
reject the null hypothesis (p-value > 0.05), the simpler constant variance model may be appropriate.

Test 3: Test the null hypothesis that the variances are adequately modeled (A3 vs A2). If this test
fails to reject the null hypothesis (p-value > 0.05), it may be inferred that the variances have
been modeled appropriately.

Test 4: Test the null hypothesis that the model for the mean fits the data (Fitted vs A3). If this
test fails to reject the null hypothesis (p-value > 0.1), the user has support for use of the
selected model.

# Serum T4, Rt, F, GD16-20, Conley et al. (2021)

## Dataset

**Name:** Serum T4, Rt, F, GD16-20, Conley et al. (2021)

| Dose | N | Mean | Std. Dev. |
| --- | --- | --- | --- |
| 0 | 6 | 18.6 | 4.4 |
| 1 | 4 | 16.7 | 2.4 |
| 3 | 4 | 15.3 | 4.2 |
| 10 | 4 | 14.2 | 5.8 |
| 30 | 4 | 15.3 | 2.8 |
| 62.5 | 4 | 12.1 | 1.4 |
| 125 | 4 | 8.4 | 1.2 |

Test 1 Dose Response: 0.0005

Test 2 Homogeneity of Variance: 0.0243

Test 3 Variance Model Selection: 0.2877

## Settings

| Setting | Value |
| --- | --- |
| BMR | 1.0 Standard Deviation |
| Distribution | Normal + Nonconstant variance |
| Adverse Direction | Down (↓) |
| Maximum Polynomial Degree | 3 |
| Confidence Level (one sided) | 0.95 |

## Maximum Likelihood Approach

| Model | BMDL | BMD | BMDU | *P*-Value | AIC | Scaled Residual at Control | Scaled Residual near BMD | Recommendation and Notes |
| --- | --- | --- | --- | --- | --- | --- | --- | --- |
| Exponential 3^ab^ | 34.56 | 49.392 | 81.517 | 0.863 | 155.945 | 0.943 | 0.123 | **Recommended - Lowest AIC** |
| Exponential 5 | 28.264 | 49.916 | 81.347 | 0.599 | 159.922 | 1.028 | 0.302 | **Viable** |
| Hill | 27.181 | 49.934 | 82.529 | 0.593 | 159.947 | 1.038 | 0.287 | **Viable** |
| Polynomial 2 | 44.159 | 63.882 | 65.204 | 0.566 | 158.996 | 1.497 | -0.201 | **Viable** |
| Polynomial 3 | 45.036 | 60.324 | 89.488 | 0.769 | 156.594 | 1.198 | -0.376 | **Viable** |
| Power | 44.826 | 58.488 | 59.698 | 0.755 | 156.687 | 1.18 | -0.375 | **Viable** |
| Linear | 45.039 | 60.583 | 87.224 | 0.77 | 156.593 | 1.2 | -0.376 | **Viable** |

^a^ BMDS recommended best fitting model

^b^ User selected best fitting model


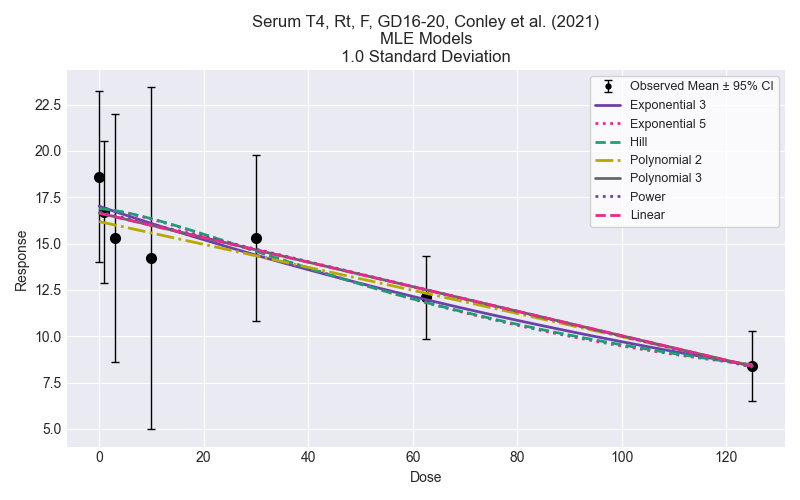


## Selected Model: Exponential 3


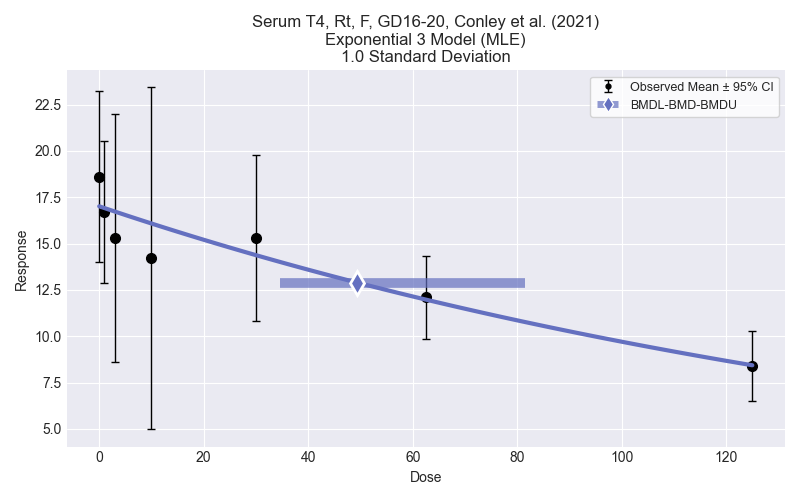


Exponential 3 Model
══════════════════════════════

Version: pybmds 25.1 (bmdscore 25.1)

Input Summary:
╒══════════════════════════════╤═══════════════════════════════╕
│ BMR │ 1.0 Standard Deviation │
│ Distribution │ Normal + Nonconstant variance │
│ Modeling Direction │ Down (↓) │
│ Confidence Level (one sided) │ 0.95 │
│ Modeling Approach │ MLE │
╘══════════════════════════════╧═══════════════════════════════╛

Parameter Settings:
╒═════════════╤═══════════╤═══════╤═══════╕
│ Parameter │ Initial │ Min │ Max │
╞═════════════╪═══════════╪═══════╪═══════╡
│ a │ 0 │ 0 │ 100 │
│ b │ 0 │ 0 │ 100 │
│ c │ 0 │ -20 │ 0 │
│ d │ 1 │ 1 │ 18 │
│ rho │ 0 │ 0 │ 18 │
│ log-alpha │ 0 │ -18 │ 18 │
╘═════════════╧═══════════╧═══════╧═══════╛

Modeling Summary:
╒════════════════╤════════════╕
│ BMD │ 49.3924 │
│ BMDL │ 34.5597 │
│ BMDU │ 81.517 │
│ AIC │ 155.945 │
│ Log-Likelihood │ -73.9723 │
│ P-Value │ 0.863135 │
│ Model d.f. │ 5 │
╘════════════════╧════════════╛

Model Parameters:
╒════════════╤═════════════╤════════════╤══════════════╕
│ Variable │ Estimate │ On Bound │ Std Error │
╞════════════╪═════════════╪════════════╪══════════════╡
│ a │ 17.0144 │ no │ 0.863112 │
│ b │ 0.00561268 │ no │ 0.000627897 │
│ d │ 1 │ yes │ Not Reported │
│ rho │ 4.2589 │ no │ 1.11373 │
│ log-alpha │ -9.23856 │ no │ 2.94771 │
╘════════════╧═════════════╧════════════╧══════════════╛
Standard errors estimates are not generated for parameters estimated on corresponding bounds,
although sampling error is present for all parameters, as a rule. Standard error estimates may not
be reliable as a basis for confidence intervals or tests when one or more parameters are on bounds.


Goodness of Fit:
╒════════╤═════╤═══════════════╤═════════════════════╤═══════════════════╕
│ Dose │ N │ Sample Mean │ Model Fitted Mean │ Scaled Residual │
╞════════╪═════╪═══════════════╪═════════════════════╪═══════════════════╡
│ 0 │ 6 │ 18.6 │ 17.0144 │ 0.942806 │
│ 1 │ 4 │ 16.7 │ 16.9192 │ -0.1077 │
│ 3 │ 4 │ 15.3 │ 16.7303 │ -0.719781 │
│ 10 │ 4 │ 14.2 │ 16.0858 │ -1.03178 │
│ 30 │ 4 │ 15.3 │ 14.3777 │ 0.64086 │
│ 62.5 │ 4 │ 12.1 │ 11.9804 │ 0.122592 │
│ 125 │ 4 │ 8.4 │ 8.43573 │ -0.077286 │
╘════════╧═════╧═══════════════╧═════════════════════╧═══════════════════╛
╒════════╤═════╤═════════════╤═══════════════════╕
│ Dose │ N │ Sample SD │ Model Fitted SD │
╞════════╪═════╪═════════════╪═══════════════════╡
│ 0 │ 6 │ 4.4 │ 4.11945 │
│ 1 │ 4 │ 2.4 │ 4.07051 │
│ 3 │ 4 │ 4.2 │ 3.97436 │
│ 10 │ 4 │ 5.8 │ 3.65538 │
│ 30 │ 4 │ 2.8 │ 2.87819 │
│ 62.5 │ 4 │ 1.4 │ 1.95175 │
│ 125 │ 4 │ 1.2 │ 0.924714 │
╘════════╧═════╧═════════════╧═══════════════════╛

Likelihoods:
╒═════════╤══════════════════╤════════════╤═════════╕
│ Model │ Log-Likelihood │ # Params │ AIC │
╞═════════╪══════════════════╪════════════╪═════════╡
│ A1 │ -77.1887 │ 8 │ 170.377 │
│ A2 │ -69.9259 │ 14 │ 167.852 │
│ A3 │ -73.0235 │ 9 │ 164.047 │
│ fitted │ -73.9723 │ 4 │ 155.945 │
│ reduced │ -87.467 │ 2 │ 178.934 │
╘═════════╧══════════════════╧════════════╧═════════╛

Tests of Mean and Variance Fits:
╒════════╤══════════════════════════════╤═════════════╤═════════════╕
│ Name │ -2 * Log(Likelihood Ratio) │ Test d.f. │ P-Value │
╞════════╪══════════════════════════════╪═════════════╪═════════════╡
│ Test 1 │ 35.0821 │ 12 │ 0.000454424 │
│ Test 2 │ 14.5255 │ 6 │ 0.0242859 │
│ Test 3 │ 6.19515 │ 5 │ 0.287691 │
│ Test 4 │ 1.89753 │ 5 │ 0.863135 │
╘════════╧══════════════════════════════╧═════════════╧═════════════╛
Test 1: Test the null hypothesis that responses and variances don't differ among dose levels
(A2 vs R). If this test fails to reject the null hypothesis (p-value > 0.05), there may not be
a dose-response.

Test 2: Test the null hypothesis that variances are homogenous (A1 vs A2). If this test fails to
reject the null hypothesis (p-value > 0.05), the simpler constant variance model may be appropriate.

Test 3: Test the null hypothesis that the variances are adequately modeled (A3 vs A2). If this test
fails to reject the null hypothesis (p-value > 0.05), it may be inferred that the variances have
been modeled appropriately.

Test 4: Test the null hypothesis that the model for the mean fits the data (Fitted vs A3). If this
test fails to reject the null hypothesis (p-value > 0.1), the user has support for use of the
selected model.

# Serum T3, Rt, F, GD16-20, Conley et al. (2021)

## Dataset

**Name:** Serum T3, Rt, F, GD16-20, Conley et al. (2021)

| Dose | N | Mean | Std. Dev. |
| --- | --- | --- | --- |
| 0 | 6 | 0.69 | 0.37 |
| 1 | 4 | 0.57 | 0.3 |
| 3 | 4 | 0.84 | 0.32 |
| 10 | 4 | 0.63 | 0.44 |
| 30 | 4 | 0.33 | 0.04 |
| 62.5 | 4 | 0.23 | 0.08 |

Test 1 Dose Response: 0.0002

Test 2 Homogeneity of Variance: 0.0006

Test 3 Variance Model Selection: 0.2139

## Settings

| Setting | Value |
| --- | --- |
| BMR | 1.0 Standard Deviation |
| Distribution | Normal + Nonconstant variance |
| Adverse Direction | Down (↓) |
| Maximum Polynomial Degree | 3 |
| Confidence Level (one sided) | 0.95 |

## Maximum Likelihood Approach

| Model | BMDL | BMD | BMDU | *P*-Value | AIC | Scaled Residual at Control | Scaled Residual near BMD | Recommendation and Notes |
| --- | --- | --- | --- | --- | --- | --- | --- | --- |
| Exponential 3 | 21.314 | 38.689 | 98.517 | 0.127 | 4.718 | -0.067 | -0.93 | **Viable** |
| Exponential 5 | 18.112 | 29.207 | - | 0.572 | 2.659 | -0.017 | 0.651 | **Viable** |
| Hill^ab^ | 14.356 | 28.599 | - | 0.773 | 0.659 | -0.017 | 0.651 | **Recommended - Lowest AIC** |
| Polynomial 2 | 33.185 | 49.275 | 86.145 | 0.063 | 6.464 | 0.205 | 0.159 | **Questionable** Goodness of fit p-value < 0.1 |
| Polynomial 3 | 33.185 | 49.274 | 86.146 | 0.063 | 6.464 | 0.205 | 0.159 | **Questionable** Goodness of fit p-value < 0.1 |
| Power | 33.188 | 48.969 | 86.152 | 0.063 | 6.464 | 0.196 | 0.166 | **Questionable** Goodness of fit p-value < 0.1 |
| Linear | 33.183 | 49.274 | 86.146 | 0.063 | 6.464 | 0.205 | 0.159 | **Questionable** Goodness of fit p-value < 0.1 |

^a^ BMDS recommended best fitting model

^b^ User selected best fitting model


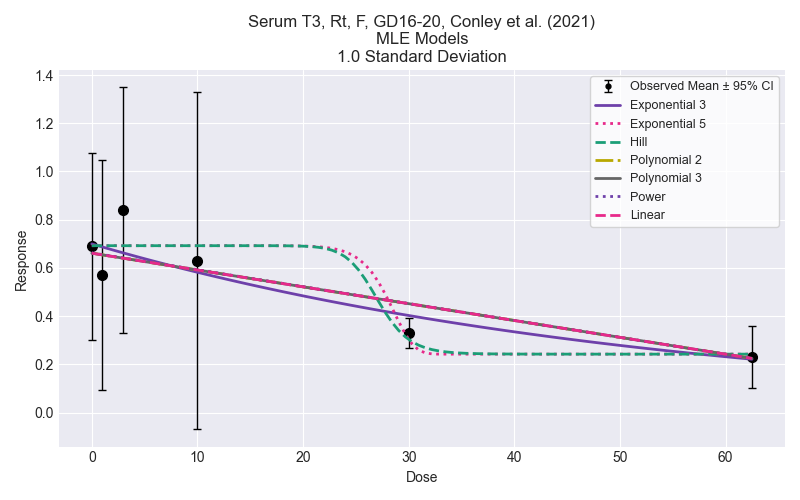


## Selected Model: Hill


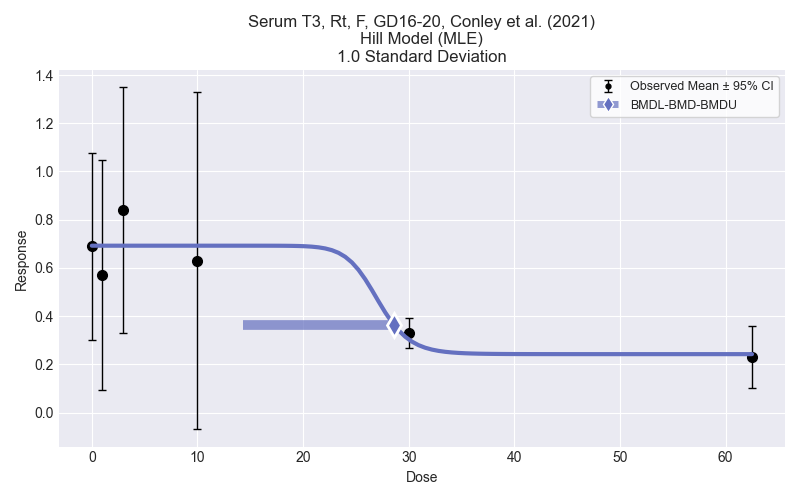


Hill Model
══════════════════════════════

Version: pybmds 25.1 (bmdscore 25.1)

Input Summary:
╒══════════════════════════════╤═══════════════════════════════╕
│ BMR │ 1.0 Standard Deviation │
│ Distribution │ Normal + Nonconstant variance │
│ Modeling Direction │ Down (↓) │
│ Confidence Level (one sided) │ 0.95 │
│ Modeling Approach │ MLE │
╘══════════════════════════════╧═══════════════════════════════╛

Parameter Settings:
╒═════════════╤═══════════╤═══════╤═══════╕
│ Parameter │ Initial │ Min │ Max │
╞═════════════╪═══════════╪═══════╪═══════╡
│ g │ 0 │ -100 │ 100 │
│ v │ 0 │ -100 │ 100 │
│ k │ 0 │ 0 │ 5 │
│ n │ 1 │ 1 │ 18 │
│ rho │ 0 │ -18 │ 18 │
│ alpha │ 0 │ -18 │ 18 │
╘═════════════╧═══════════╧═══════╧═══════╛

Modeling Summary:
╒════════════════╤══════════════╕
│ BMD │ 28.599 │
│ BMDL │ 14.3557 │
│ BMDU │ -9999 │
│ AIC │ 0.658723 │
│ Log-Likelihood │ 4.67064 │
│ P-Value │ 0.773294 │
│ Model d.f. │ 3 │
╘════════════════╧══════════════╛

Model Parameters:
╒════════════╤════════════╤════════════╤══════════════╕
│ Variable │ Estimate │ On Bound │ Std Error │
╞════════════╪════════════╪════════════╪══════════════╡
│ g │ 0.692224 │ no │ 0.0764106 │
│ v │ -0.44986 │ no │ 0.082697 │
│ k │ 27.0645 │ no │ 1.14212 │
│ n │ 18 │ yes │ Not Reported │
│ rho │ 3.36001 │ no │ 0.751982 │
│ alpha │ 0.370781 │ no │ 0.0952609 │
╘════════════╧════════════╧════════════╧══════════════╛
Standard errors estimates are not generated for parameters estimated on corresponding bounds,
although sampling error is present for all parameters, as a rule. Standard error estimates may not
be reliable as a basis for confidence intervals or tests when one or more parameters are on bounds.


Goodness of Fit:
╒════════╤═════╤═══════════════╤═════════════════════╤═══════════════════╕
│ Dose │ N │ Sample Mean │ Model Fitted Mean │ Scaled Residual │
╞════════╪═════╪═══════════════╪═════════════════════╪═══════════════════╡
│ 0 │ 6 │ 0.69 │ 0.692224 │ -0.0165999 │
│ 1 │ 4 │ 0.57 │ 0.692224 │ -0.744757 │
│ 3 │ 4 │ 0.84 │ 0.692224 │ 0.900451 │
│ 10 │ 4 │ 0.63 │ 0.692224 │ -0.379156 │
│ 30 │ 4 │ 0.33 │ 0.303301 │ 0.650765 │
│ 62.5 │ 4 │ 0.23 │ 0.242365 │ -0.439296 │
╘════════╧═════╧═══════════════╧═════════════════════╧═══════════════════╛
╒════════╤═════╤═════════════╤═══════════════════╕
│ Dose │ N │ Sample SD │ Model Fitted SD │
╞════════╪═════╪═════════════╪═══════════════════╡
│ 0 │ 6 │ 0.37 │ 0.328226 │
│ 1 │ 4 │ 0.3 │ 0.328226 │
│ 3 │ 4 │ 0.32 │ 0.328226 │
│ 10 │ 4 │ 0.44 │ 0.328226 │
│ 30 │ 4 │ 0.04 │ 0.0820549 │
│ 62.5 │ 4 │ 0.08 │ 0.0562945 │
╘════════╧═════╧═════════════╧═══════════════════╛

Likelihoods:
╒═════════╤══════════════════╤════════════╤═══════════╕
│ Model │ Log-Likelihood │ # Params │ AIC │
╞═════════╪══════════════════╪════════════╪═══════════╡
│ A1 │ -2.64999 │ 7 │ 19.3 │
│ A2 │ 8.13287 │ 12 │ 7.73427 │
│ A3 │ 5.22847 │ 8 │ 5.54306 │
│ fitted │ 4.67064 │ 5 │ 0.658723 │
│ reduced │ -8.59482 │ 2 │ 21.1896 │
╘═════════╧══════════════════╧════════════╧═══════════╛

Tests of Mean and Variance Fits:
╒════════╤══════════════════════════════╤═════════════╤═════════════╕
│ Name │ -2 * Log(Likelihood Ratio) │ Test d.f. │ P-Value │
╞════════╪══════════════════════════════╪═════════════╪═════════════╡
│ Test 1 │ 33.4554 │ 10 │ 0.000228308 │
│ Test 2 │ 21.5657 │ 5 │ 0.000633062 │
│ Test 3 │ 5.80879 │ 4 │ 0.21389 │
│ Test 4 │ 1.11567 │ 3 │ 0.773294 │
╘════════╧══════════════════════════════╧═════════════╧═════════════╛
Test 1: Test the null hypothesis that responses and variances don't differ among dose levels
(A2 vs R). If this test fails to reject the null hypothesis (p-value > 0.05), there may not be
a dose-response.

Test 2: Test the null hypothesis that variances are homogenous (A1 vs A2). If this test fails to
reject the null hypothesis (p-value > 0.05), the simpler constant variance model may be appropriate.

Test 3: Test the null hypothesis that the variances are adequately modeled (A3 vs A2). If this test
fails to reject the null hypothesis (p-value > 0.05), it may be inferred that the variances have
been modeled appropriately.

Test 4: Test the null hypothesis that the model for the mean fits the data (Fitted vs A3). If this
test fails to reject the null hypothesis (p-value > 0.1), the user has support for use of the
selected model.

# Relative liver wt, Rt, F, GD16-20, Conley et al. (2021)

## Dataset

**Name:** Relative liver wt, Rt, F, GD16-20, Conley et al. (2021)

| Dose | N | Mean | Std. Dev. |
| --- | --- | --- | --- |
| 0 | 6 | 40.8 | 1.71 |
| 1 | 4 | 40.6 | 2.6 |
| 3 | 4 | 39.6 | 1.8 |
| 10 | 4 | 39.9 | 1.6 |
| 30 | 4 | 42.1 | 1.6 |
| 62.5 | 4 | 45 | 2.6 |
| 125 | 4 | 45 | 2.2 |

Test 1 Dose Response: 0.0058

Test 2 Homogeneity of Variance: 0.895

Test 3 Variance Model Selection: 0.895

## Settings

| Setting | Value |
| --- | --- |
| BMR | 1.0 Standard Deviation |
| Distribution | Normal + Constant variance |
| Adverse Direction | Up (↑) |
| Maximum Polynomial Degree | 3 |
| Confidence Level (one sided) | 0.95 |

## Maximum Likelihood Approach

| Model | BMDL | BMD | BMDU | *P*-Value | AIC | Scaled Residual at Control | Scaled Residual near BMD | Recommendation and Notes |
| --- | --- | --- | --- | --- | --- | --- | --- | --- |
| Exponential 3 | 35.241 | 48.328 | 78.057 | 0.189 | 133.106 | 0.396 | 1.892 | **Viable** |
| Exponential 5^ab^ | 18.487 | 30.01 | 48.559 | 0.711 | 131.026 | 0.688 | <0.001 | **Recommended - Lowest AIC** |
| Hill | 27.449 | 30.013 | 34.994 | 0.711 | 131.026 | 0.688 | <0.001 | **Viable** |
| Polynomial 2 | 32.951 | 45.803 | 75.04 | 0.209 | 132.806 | 0.444 | 0.351 | **Viable** |
| Polynomial 3 | 32.951 | 45.751 | 75.143 | 0.209 | 132.806 | 0.446 | 0.351 | **Viable** |
| Power | 32.951 | 45.836 | 75.542 | 0.209 | 132.806 | 0.443 | 0.351 | **Viable** |
| Linear | 32.951 | 45.836 | 74.717 | 0.209 | 132.806 | 0.443 | 0.351 | **Viable** |

^a^ BMDS recommended best fitting model

^b^ User selected best fitting model


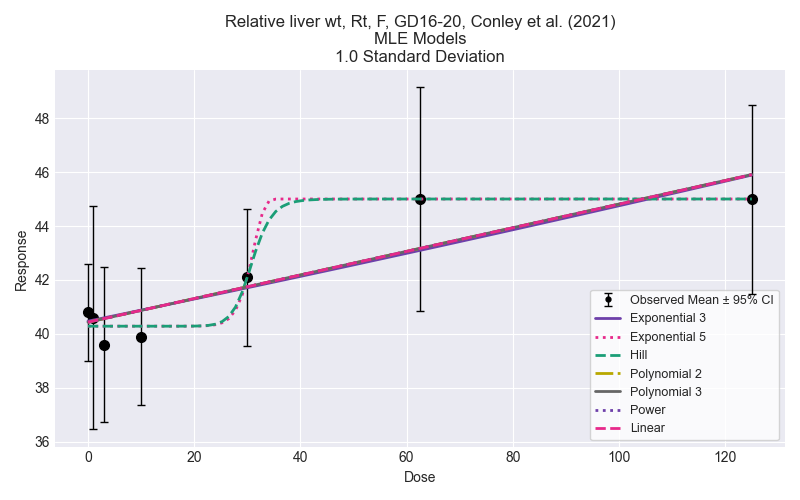


## Selected Model: Exponential 5


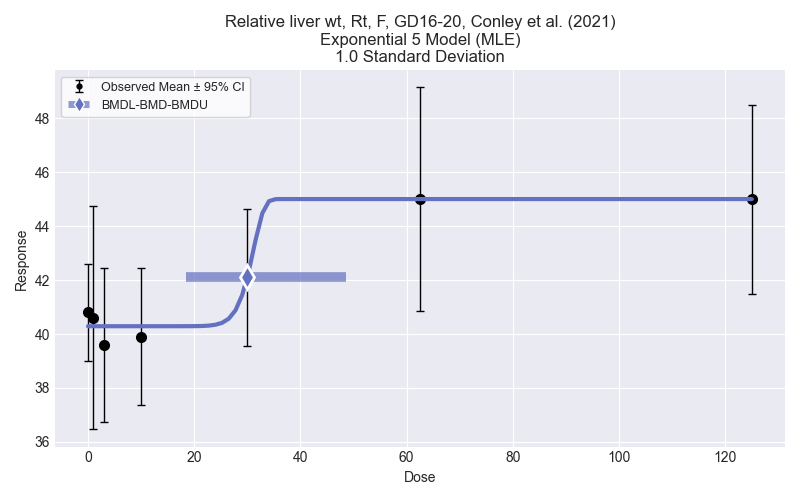


Exponential 5 Model
══════════════════════════════

Version: pybmds 25.1 (bmdscore 25.1)

Input Summary:
╒══════════════════════════════╤════════════════════════════╕
│ BMR │ 1.0 Standard Deviation │
│ Distribution │ Normal + Constant variance │
│ Modeling Direction │ Up (↑) │
│ Confidence Level (one sided) │ 0.95 │
│ Modeling Approach │ MLE │
╘══════════════════════════════╧════════════════════════════╛

Parameter Settings:
╒═════════════╤═══════════╤═══════╤═══════╕
│ Parameter │ Initial │ Min │ Max │
╞═════════════╪═══════════╪═══════╪═══════╡
│ a │ 0 │ 0 │ 100 │
│ b │ 0 │ 0 │ 100 │
│ c │ 0 │ 0 │ 20 │
│ d │ 1 │ 1 │ 18 │
│ log-alpha │ 0 │ -18 │ 18 │
╘═════════════╧═══════════╧═══════╧═══════╛

Modeling Summary:
╒════════════════╤════════════╕
│ BMD │ 30.0097 │
│ BMDL │ 18.4873 │
│ BMDU │ 48.5588 │
│ AIC │ 131.026 │
│ Log-Likelihood │ -60.5131 │
│ P-Value │ 0.710511 │
│ Model d.f. │ 3 │
╘════════════════╧════════════╛

Model Parameters:
╒════════════╤════════════╤════════════╤═════════════╕
│ Variable │ Estimate │ On Bound │ Std Error │
╞════════════╪════════════╪════════════╪═════════════╡
│ a │ 40.2889 │ no │ 0.428688 │
│ b │ 0.0319243 │ no │ 0.0375439 │
│ c │ 1.11693 │ no │ 0.0178159 │
│ d │ 16.7432 │ no │ 455.604 │
│ log-alpha │ 1.19633 │ no │ 0.258195 │
╘════════════╧════════════╧════════════╧═════════════╛

Goodness of Fit:
╒════════╤═════╤═══════════════╤═════════════════════╤═══════════════════╕
│ Dose │ N │ Sample Mean │ Model Fitted Mean │ Scaled Residual │
╞════════╪═════╪═══════════════╪═════════════════════╪═══════════════════╡
│ 0 │ 6 │ 40.8 │ 40.2889 │ 0.688354 │
│ 1 │ 4 │ 40.6 │ 40.2889 │ 0.342111 │
│ 3 │ 4 │ 39.6 │ 40.2889 │ -0.757531 │
│ 10 │ 4 │ 39.9 │ 40.2889 │ -0.427638 │
│ 30 │ 4 │ 42.1 │ 42.1 │ 7.3765e-09 │
│ 62.5 │ 4 │ 45 │ 45 │ -4.26235e-08 │
│ 125 │ 4 │ 45 │ 45 │ -4.26235e-08 │
╘════════╧═════╧═══════════════╧═════════════════════╧═══════════════════╛
╒════════╤═════╤═════════════╤═══════════════════╕
│ Dose │ N │ Sample SD │ Model Fitted SD │
╞════════╪═════╪═════════════╪═══════════════════╡
│ 0 │ 6 │ 1.71 │ 1.81878 │
│ 1 │ 4 │ 2.6 │ 1.81878 │
│ 3 │ 4 │ 1.8 │ 1.81878 │
│ 10 │ 4 │ 1.6 │ 1.81878 │
│ 30 │ 4 │ 1.6 │ 1.81878 │
│ 62.5 │ 4 │ 2.6 │ 1.81878 │
│ 125 │ 4 │ 2.2 │ 1.81878 │
╘════════╧═════╧═════════════╧═══════════════════╛

Likelihoods:
╒═════════╤══════════════════╤════════════╤═════════╕
│ Model │ Log-Likelihood │ # Params │ AIC │
╞═════════╪══════════════════╪════════════╪═════════╡
│ A1 │ -59.8237 │ 8 │ 135.647 │
│ A2 │ -58.6971 │ 14 │ 145.394 │
│ A3 │ -59.8237 │ 8 │ 135.647 │
│ fitted │ -60.5131 │ 5 │ 131.026 │
│ reduced │ -72.6319 │ 2 │ 149.264 │
╘═════════╧══════════════════╧════════════╧═════════╛

Tests of Mean and Variance Fits:
╒════════╤══════════════════════════════╤═════════════╤════════════╕
│ Name │ -2 * Log(Likelihood Ratio) │ Test d.f. │ P-Value │
╞════════╪══════════════════════════════╪═════════════╪════════════╡
│ Test 1 │ 27.8696 │ 12 │ 0.00578021 │
│ Test 2 │ 2.25317 │ 6 │ 0.895005 │
│ Test 3 │ 2.25317 │ 6 │ 0.895005 │
│ Test 4 │ 1.3788 │ 3 │ 0.710511 │
╘════════╧══════════════════════════════╧═════════════╧════════════╛
Test 1: Test the null hypothesis that responses and variances don't differ among dose levels
(A2 vs R). If this test fails to reject the null hypothesis (p-value > 0.05), there may not be
a dose-response.

Test 2: Test the null hypothesis that variances are homogenous (A1 vs A2). If this test fails to
reject the null hypothesis (p-value > 0.05), the simpler constant variance model may be appropriate.

Test 3: Test the null hypothesis that the variances are adequately modeled (A3 vs A2). If this test
fails to reject the null hypothesis (p-value > 0.05), it may be inferred that the variances have
been modeled appropriately.

Test 4: Test the null hypothesis that the model for the mean fits the data (Fitted vs A3). If this
test fails to reject the null hypothesis (p-value > 0.1), the user has support for use of the
selected model.

# Serum albumin, Rt, F, GD8-PND2, Conley et al. (2021)

## Dataset

**Name:** Serum albumin, Rt, F, GD8-PND2, Conley et al. (2021)

| Dose | N | Mean | Std. Dev. |
| --- | --- | --- | --- |
| 0 | 5 | 3.92 | 0.18 |
| 10 | 5 | 3.81 | 0.16 |
| 30 | 5 | 3.86 | 0.31 |
| 62.5 | 4 | 3.73 | 0.14 |
| 125 | 5 | 3.7 | 0.22 |
| 250 | 5 | 3.18 | 0.16 |

Test 1 Dose Response: 0.0001

Test 2 Homogeneity of Variance: 0.4651

Test 3 Variance Model Selection: 0.4651

## Settings

| Setting | Value |
| --- | --- |
| BMR | 1.0 Standard Deviation |
| Distribution | Normal + Constant variance |
| Adverse Direction | Down (↓) |
| Maximum Polynomial Degree | 3 |
| Confidence Level (one sided) | 0.95 |

## Maximum Likelihood Approach

| Model | BMDL | BMD | BMDU | *P*-Value | AIC | Scaled Residual at Control | Scaled Residual near BMD | Recommendation and Notes |
| --- | --- | --- | --- | --- | --- | --- | --- | --- |
| Exponential 3 | 60.524 | 125.169 | 226.99 | 0.628 | -6.462 | 0.753 | 0.375 | **Viable** |
| Exponential 5 | 60.524 | 125.169 | 217.109 | 0.419 | -4.462 | 0.753 | 0.375 | **Viable** |
| Hill | 62.467 | 125.256 | 231.708 | 0.424 | -4.484 | 0.749 | 0.367 | **Viable** |
| Polynomial 2 | 61.169 | 110.946 | 113.243 | 0.656 | -6.586 | 0.523 | 0.591 | **Viable** |
| Polynomial 3^ab^ | 62.638 | 128.338 | 181.682 | 0.849 | -8.831 | 0.658 | 0.177 | **Recommended - Lowest AIC** |
| Power | 60.677 | 124.402 | 230.868 | 0.638 | -6.504 | 0.736 | 0.381 | **Viable** |
| Linear | 53.777 | 71.692 | 106.365 | 0.464 | -6.611 | 0.038 | -0.174 | **Viable** |

^a^ BMDS recommended best fitting model

^b^ User selected best fitting model


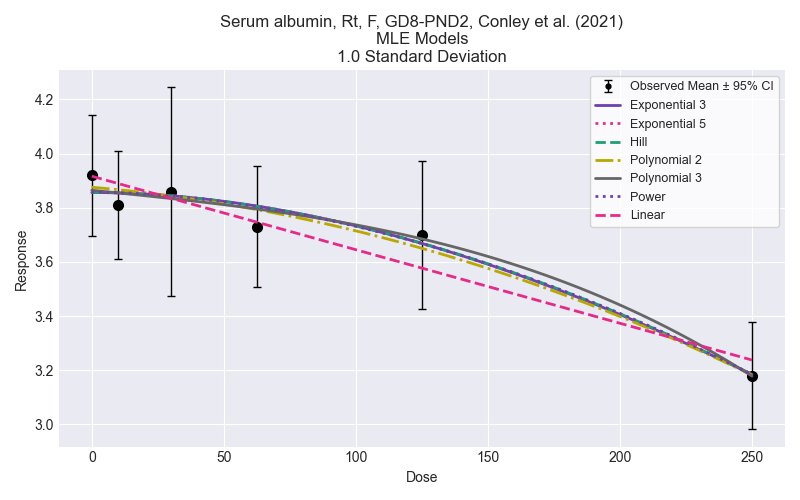


## Selected Model: Polynomial 3


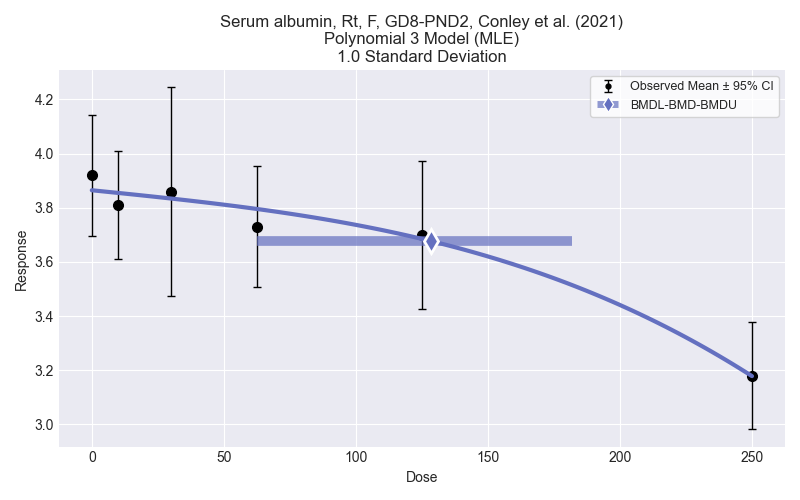


Polynomial 3 Model
══════════════════════════════

Version: pybmds 25.1 (bmdscore 25.1)

Input Summary:
╒══════════════════════════════╤════════════════════════════╕
│ BMR │ 1.0 Standard Deviation │
│ Distribution │ Normal + Constant variance │
│ Modeling Direction │ Down (↓) │
│ Confidence Level (one sided) │ 0.95 │
│ Modeling Approach │ MLE │
│ Degree │ 3 │
╘══════════════════════════════╧════════════════════════════╛

Parameter Settings:
╒═════════════╤═══════════╤═════════╤════════╕
│ Parameter │ Initial │ Min │ Max │
╞═════════════╪═══════════╪═════════╪════════╡
│ g │ 0 │ -1e+06 │ 1e+06 │
│ b1 │ 0 │ -1e+06 │ 0 │
│ b2 │ 0 │ -1e+06 │ 0 │
│ b3 │ 0 │ -1e+06 │ 0 │
│ alpha │ 0 │ -18 │ 18 │
╘═════════════╧═══════════╧═════════╧════════╛

Modeling Summary:
╒════════════════╤════════════╕
│ BMD │ 128.338 │
│ BMDL │ 62.6378 │
│ BMDU │ 181.682 │
│ AIC │ -8.83067 │
│ Log-Likelihood │ 7.41534 │
│ P-Value │ 0.849404 │
│ Model d.f. │ 4 │
╘════════════════╧════════════╛

Model Parameters:
╒════════════╤══════════════╤════════════╤══════════════╕
│ Variable │ Estimate │ On Bound │ Std Error │
╞════════════╪══════════════╪════════════╪══════════════╡
│ g │ 3.8648 │ no │ 0.055252 │
│ b1 │ -0.000990723 │ no │ 0.000960459 │
│ b2 │ -1.1873e-07 │ yes │ Not Reported │
│ b3 │ -2.7604e-08 │ yes │ Not Reported │
│ alpha │ 0.0351381 │ no │ 0.000324768 │
╘════════════╧══════════════╧════════════╧══════════════╛
Standard errors estimates are not generated for parameters estimated on corresponding bounds,
although sampling error is present for all parameters, as a rule. Standard error estimates may not
be reliable as a basis for confidence intervals or tests when one or more parameters are on bounds.


Goodness of Fit:
╒════════╤═════╤═══════════════╤═════════════════════╤═══════════════════╕
│ Dose │ N │ Sample Mean │ Model Fitted Mean │ Scaled Residual │
╞════════╪═════╪═══════════════╪═════════════════════╪═══════════════════╡
│ 0 │ 5 │ 3.92 │ 3.8648 │ 0.658464 │
│ 10 │ 5 │ 3.81 │ 3.85485 │ -0.535049 │
│ 30 │ 5 │ 3.86 │ 3.83423 │ 0.307446 │
│ 62.5 │ 4 │ 3.73 │ 3.79568 │ -0.700737 │
│ 125 │ 5 │ 3.7 │ 3.68519 │ 0.176657 │
│ 250 │ 5 │ 3.18 │ 3.17839 │ 0.019251 │
╘════════╧═════╧═══════════════╧═════════════════════╧═══════════════════╛
╒════════╤═════╤═════════════╤═══════════════════╕
│ Dose │ N │ Sample SD │ Model Fitted SD │
╞════════╪═════╪═════════════╪═══════════════════╡
│ 0 │ 5 │ 0.18 │ 0.187452 │
│ 10 │ 5 │ 0.16 │ 0.187452 │
│ 30 │ 5 │ 0.31 │ 0.187452 │
│ 62.5 │ 4 │ 0.14 │ 0.187452 │
│ 125 │ 5 │ 0.22 │ 0.187452 │
│ 250 │ 5 │ 0.16 │ 0.187452 │
╘════════╧═════╧═════════════╧═══════════════════╛

Likelihoods:
╒═════════╤══════════════════╤════════════╤══════════╕
│ Model │ Log-Likelihood │ # Params │ AIC │
╞═════════╪══════════════════╪════════════╪══════════╡
│ A1 │ 8.1003 │ 7 │ -2.20061 │
│ A2 │ 10.4063 │ 12 │ 3.18749 │
│ A3 │ 8.1003 │ 7 │ -2.20061 │
│ fitted │ 7.41534 │ 3 │ -8.83067 │
│ reduced │ -7.02992 │ 2 │ 18.0598 │
╘═════════╧══════════════════╧════════════╧══════════╛

Tests of Mean and Variance Fits:
╒════════╤══════════════════════════════╤═════════════╤═════════════╕
│ Name │ -2 * Log(Likelihood Ratio) │ Test d.f. │ P-Value │
╞════════╪══════════════════════════════╪═════════════╪═════════════╡
│ Test 1 │ 34.8723 │ 10 │ 0.000131285 │
│ Test 2 │ 4.6119 │ 5 │ 0.465053 │
│ Test 3 │ 4.6119 │ 5 │ 0.465053 │
│ Test 4 │ 1.36993 │ 4 │ 0.849404 │
╘════════╧══════════════════════════════╧═════════════╧═════════════╛
Test 1: Test the null hypothesis that responses and variances don't differ among dose levels
(A2 vs R). If this test fails to reject the null hypothesis (p-value > 0.05), there may not be
a dose-response.

Test 2: Test the null hypothesis that variances are homogenous (A1 vs A2). If this test fails to
reject the null hypothesis (p-value > 0.05), the simpler constant variance model may be appropriate.

Test 3: Test the null hypothesis that the variances are adequately modeled (A3 vs A2). If this test
fails to reject the null hypothesis (p-value > 0.05), it may be inferred that the variances have
been modeled appropriately.

Test 4: Test the null hypothesis that the model for the mean fits the data (Fitted vs A3). If this
test fails to reject the null hypothesis (p-value > 0.1), the user has support for use of the
selected model.

# Serum T4, Rt, F, GD8-PND2, Conley et al. (2021)

## Dataset

**Name:** Serum T4, Rt, F, GD8-PND2, Conley et al. (2021)

| Dose | N | Mean | Std. Dev. |
| --- | --- | --- | --- |
| 0 | 5 | 32.4 | 8.05 |
| 10 | 5 | 29.7 | 5.37 |
| 30 | 5 | 26.2 | 3.35 |
| 62.5 | 4 | 16 | 3 |
| 125 | 5 | 13.7 | 3.58 |
| 250 | 5 | 10.8 | 2.24 |

Test 1 Dose Response: <0.0001

Test 2 Homogeneity of Variance: 0.0523

Test 3 Variance Model Selection: 0.0523

## Settings

| Setting | Value |
| --- | --- |
| BMR | 1.0 Standard Deviation |
| Distribution | Normal + Constant variance |
| Adverse Direction | Down (↓) |
| Maximum Polynomial Degree | 3 |
| Confidence Level (one sided) | 0.95 |

## Maximum Likelihood Approach

| Model | BMDL | BMD | BMDU | *P*-Value | AIC | Scaled Residual at Control | Scaled Residual near BMD | Recommendation and Notes |
| --- | --- | --- | --- | --- | --- | --- | --- | --- |
| Exponential 3 | 20.192 | 29.417 | 47.728 | 0.047 | 181.508 | 0.682 | 0.18 | **Questionable** Goodness of fit p-value < 0.1 |
| Exponential 5 | 8.971 | 24.479 | 43.015 | 0.426 | 177.592 | 0.374 | 0.3 | **Viable** |
| Hill^ab^ | 8.791 | 25.186 | 40.713 | 0.487 | 177.322 | 0.465 | 0.339 | **Recommended - Lowest AIC** |
| Polynomial 2 | 52.02 | 68.935 | 101.281 | 0.001 | 190.139 | 1.55 | -2.467 | **Questionable** \|Residual near BMD\| > 2.0 Goodness of fit p-value < 0.1 |
| Polynomial 3 | 52.018 | 68.983 | 101.28 | 0.001 | 190.139 | 1.552 | -2.466 | **Questionable** \|Residual near BMD\| > 2.0 Goodness of fit p-value < 0.1 |
| Power | 52.019 | 68.971 | 101.216 | 0.001 | 190.139 | 1.551 | -2.466 | **Questionable** \|Residual near BMD\| > 2.0 Goodness of fit p-value < 0.1 |
| Linear | 52.019 | 68.971 | 101.28 | 0.001 | 190.139 | 1.551 | -2.466 | **Questionable** \|Residual near BMD\| > 2.0 Goodness of fit p-value < 0.1 |

^a^ BMDS recommended best fitting model

^b^ User selected best fitting model


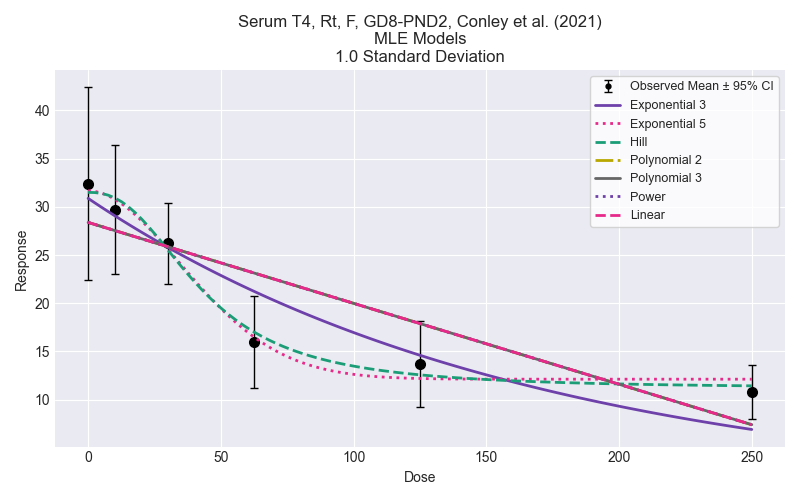


## Selected Model: Hill


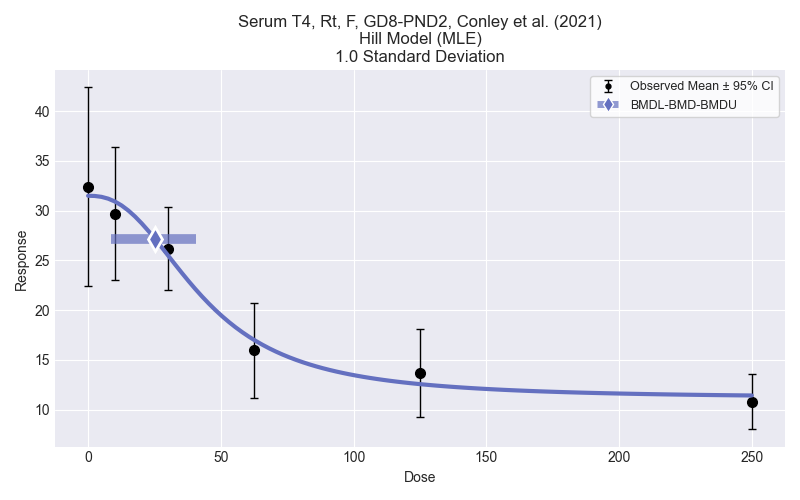


Hill Model
══════════════════════════════

Version: pybmds 25.1 (bmdscore 25.1)

Input Summary:
╒══════════════════════════════╤════════════════════════════╕
│ BMR │ 1.0 Standard Deviation │
│ Distribution │ Normal + Constant variance │
│ Modeling Direction │ Down (↓) │
│ Confidence Level (one sided) │ 0.95 │
│ Modeling Approach │ MLE │
╘══════════════════════════════╧════════════════════════════╛

Parameter Settings:
╒═════════════╤═══════════╤═══════╤═══════╕
│ Parameter │ Initial │ Min │ Max │
╞═════════════╪═══════════╪═══════╪═══════╡
│ g │ 0 │ -100 │ 100 │
│ v │ 0 │ -100 │ 100 │
│ k │ 0 │ 0 │ 5 │
│ n │ 1 │ 1 │ 18 │
│ alpha │ 0 │ -18 │ 18 │
╘═════════════╧═══════════╧═══════╧═══════╛

Modeling Summary:
╒════════════════╤════════════╕
│ BMD │ 25.1862 │
│ BMDL │ 8.79129 │
│ BMDU │ 40.7131 │
│ AIC │ 177.322 │
│ Log-Likelihood │ -83.6612 │
│ P-Value │ 0.487157 │
│ Model d.f. │ 2 │
╘════════════════╧════════════╛

Model Parameters:
╒════════════╤════════════╤════════════╤═════════════╕
│ Variable │ Estimate │ On Bound │ Std Error │
╞════════════╪════════════╪════════════╪═════════════╡
│ g │ 31.4996 │ no │ 1.71457 │
│ v │ -20.3441 │ no │ 3.61361 │
│ k │ 43.1035 │ no │ 8.52857 │
│ n │ 2.43332 │ no │ 1.58596 │
│ alpha │ 18.7625 │ no │ 92.4465 │
╘════════════╧════════════╧════════════╧═════════════╛

Goodness of Fit:
╒════════╤═════╤═══════════════╤═════════════════════╤═══════════════════╕
│ Dose │ N │ Sample Mean │ Model Fitted Mean │ Scaled Residual │
╞════════╪═════╪═══════════════╪═════════════════════╪═══════════════════╡
│ 0 │ 5 │ 32.4 │ 31.4996 │ 0.464821 │
│ 10 │ 5 │ 29.7 │ 30.9343 │ -0.637199 │
│ 30 │ 5 │ 26.2 │ 25.5429 │ 0.339188 │
│ 62.5 │ 4 │ 16 │ 17.0187 │ -0.47037 │
│ 125 │ 5 │ 13.7 │ 12.5742 │ 0.581176 │
│ 250 │ 5 │ 10.8 │ 11.434 │ -0.327275 │
╘════════╧═════╧═══════════════╧═════════════════════╧═══════════════════╛
╒════════╤═════╤═════════════╤═══════════════════╕
│ Dose │ N │ Sample SD │ Model Fitted SD │
╞════════╪═════╪═════════════╪═══════════════════╡
│ 0 │ 5 │ 8.05 │ 4.33157 │
│ 10 │ 5 │ 5.37 │ 4.33157 │
│ 30 │ 5 │ 3.35 │ 4.33157 │
│ 62.5 │ 4 │ 3 │ 4.33157 │
│ 125 │ 5 │ 3.58 │ 4.33157 │
│ 250 │ 5 │ 2.24 │ 4.33157 │
╘════════╧═════╧═════════════╧═══════════════════╛

Likelihoods:
╒═════════╤══════════════════╤════════════╤═════════╕
│ Model │ Log-Likelihood │ # Params │ AIC │
╞═════════╪══════════════════╪════════════╪═════════╡
│ A1 │ -82.942 │ 7 │ 179.884 │
│ A2 │ -77.4655 │ 12 │ 178.931 │
│ A3 │ -82.942 │ 7 │ 179.884 │
│ fitted │ -83.6612 │ 5 │ 177.322 │
│ reduced │ -106.09 │ 2 │ 216.18 │
╘═════════╧══════════════════╧════════════╧═════════╛

Tests of Mean and Variance Fits:
╒════════╤══════════════════════════════╤═════════════╤═════════════╕
│ Name │ -2 * Log(Likelihood Ratio) │ Test d.f. │ P-Value │
╞════════╪══════════════════════════════╪═════════════╪═════════════╡
│ Test 1 │ 57.2492 │ 10 │ 1.19671e-08 │
│ Test 2 │ 10.953 │ 5 │ 0.0523196 │
│ Test 3 │ 10.953 │ 5 │ 0.0523196 │
│ Test 4 │ 1.43834 │ 2 │ 0.487157 │
╘════════╧══════════════════════════════╧═════════════╧═════════════╛
Test 1: Test the null hypothesis that responses and variances don't differ among dose levels
(A2 vs R). If this test fails to reject the null hypothesis (p-value > 0.05), there may not be
a dose-response.

Test 2: Test the null hypothesis that variances are homogenous (A1 vs A2). If this test fails to
reject the null hypothesis (p-value > 0.05), the simpler constant variance model may be appropriate.

Test 3: Test the null hypothesis that the variances are adequately modeled (A3 vs A2). If this test
fails to reject the null hypothesis (p-value > 0.05), it may be inferred that the variances have
been modeled appropriately.

Test 4: Test the null hypothesis that the model for the mean fits the data (Fitted vs A3). If this
test fails to reject the null hypothesis (p-value > 0.1), the user has support for use of the
selected model.

# Serum T3, Rt, F, GD8-PND2, Conley et al. (2021)

## Dataset

**Name:** Serum T3, Rt, F, GD8-PND2, Conley et al. (2021)

| Dose | N | Mean | Std. Dev. |
| --- | --- | --- | --- |
| 0 | 5 | 1.03 | 0.25 |
| 10 | 5 | 1.03 | 0.34 |
| 30 | 5 | 0.68 | 0.29 |
| 62.5 | 4 | 0.5 | 0.36 |
| 125 | 5 | 0.52 | 0.47 |

Test 1 Dose Response: 0.1083

Test 2 Homogeneity of Variance: 0.6801

Test 3 Variance Model Selection: 0.6801

## Settings

| Setting | Value |
| --- | --- |
| BMR | 1.0 Standard Deviation |
| Distribution | Normal + Constant variance |
| Adverse Direction | Down (↓) |
| Maximum Polynomial Degree | 3 |
| Confidence Level (one sided) | 0.95 |

## Maximum Likelihood Approach

| Model | BMDL | BMD | BMDU | *P*-Value | AIC | Scaled Residual at Control | Scaled Residual near BMD | Recommendation and Notes |
| --- | --- | --- | --- | --- | --- | --- | --- | --- |
| Exponential 3 | 25.237 | 52.995 | 171.056 | 0.447 | 20.707 | 0.168 | -0.792 | **Viable** |
| Exponential 5 | 9.982 | 29.421 | 23858.771 | 0.924 | 22.055 | -0. | -0. | **Viable** |
| Hill^ab^ | 9.879 | 29.462 | - | 0.995 | 20.055 | -0. | <0.001 | **Recommended - Lowest BMDL** |
| Polynomial 2 | 48.479 | 78.86 | 208.053 | 0.287 | 21.822 | 0.508 | -1.111 | **Viable** |
| Polynomial 3 | 48.476 | 78.985 | 208.068 | 0.287 | 21.823 | 0.51 | -1.112 | **Viable** |
| Power | 48.48 | 78.747 | 208.034 | 0.287 | 21.822 | 0.506 | -1.111 | **Viable** |
| Linear | 48.48 | 78.747 | 208.047 | 0.287 | 21.822 | 0.506 | -1.111 | **Viable** |

^a^ BMDS recommended best fitting model

^b^ User selected best fitting model


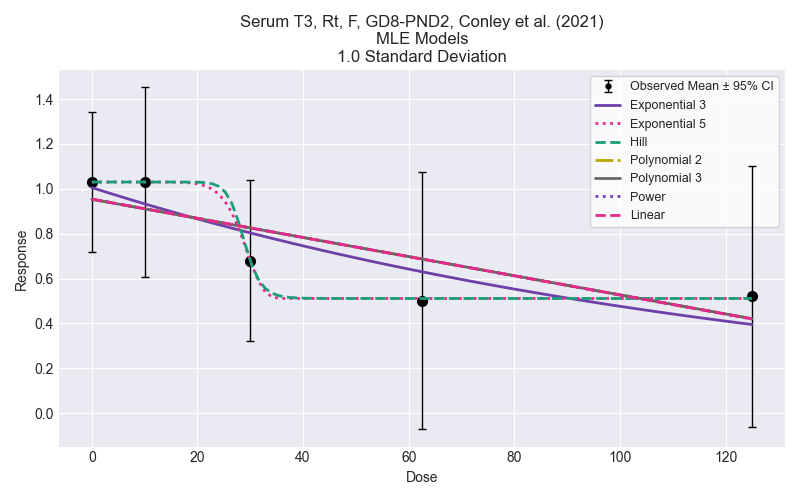


## Selected Model: Hill


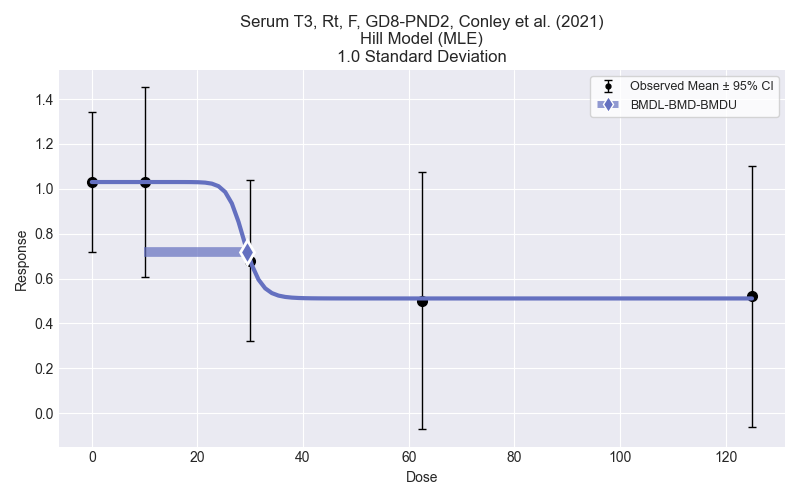


Hill Model
══════════════════════════════

Version: pybmds 25.1 (bmdscore 25.1)

Input Summary:
╒══════════════════════════════╤════════════════════════════╕
│ BMR │ 1.0 Standard Deviation │
│ Distribution │ Normal + Constant variance │
│ Modeling Direction │ Down (↓) │
│ Confidence Level (one sided) │ 0.95 │
│ Modeling Approach │ MLE │
╘══════════════════════════════╧════════════════════════════╛

Parameter Settings:
╒═════════════╤═══════════╤═══════╤═══════╕
│ Parameter │ Initial │ Min │ Max │
╞═════════════╪═══════════╪═══════╪═══════╡
│ g │ 0 │ -100 │ 100 │
│ v │ 0 │ -100 │ 100 │
│ k │ 0 │ 0 │ 5 │
│ n │ 1 │ 1 │ 18 │
│ alpha │ 0 │ -18 │ 18 │
╘═════════════╧═══════════╧═══════╧═══════╛

Modeling Summary:
╒════════════════╤══════════════╕
│ BMD │ 29.4624 │
│ BMDL │ 9.879 │
│ BMDU │ -9999 │
│ AIC │ 20.055 │
│ Log-Likelihood │ -6.02749 │
│ P-Value │ 0.995416 │
│ Model d.f. │ 2 │
╘════════════════╧══════════════╛

Model Parameters:
╒════════════╤════════════╤════════════╤══════════════╕
│ Variable │ Estimate │ On Bound │ Std Error │
╞════════════╪════════════╪════════════╪══════════════╡
│ g │ 1.03 │ no │ 0.0983631 │
│ v │ -0.518889 │ no │ 0.142918 │
│ k │ 28.8098 │ no │ 2.23331 │
│ n │ 18 │ yes │ Not Reported │
│ alpha │ 0.0967537 │ no │ 0.00270236 │
╘════════════╧════════════╧════════════╧══════════════╛
Standard errors estimates are not generated for parameters estimated on corresponding bounds,
although sampling error is present for all parameters, as a rule. Standard error estimates may not
be reliable as a basis for confidence intervals or tests when one or more parameters are on bounds.


Goodness of Fit:
╒════════╤═════╤═══════════════╤═════════════════════╤═══════════════════╕
│ Dose │ N │ Sample Mean │ Model Fitted Mean │ Scaled Residual │
╞════════╪═════╪═══════════════╪═════════════════════╪═══════════════════╡
│ 0 │ 5 │ 1.03 │ 1.03 │ -1.75498e-08 │
│ 10 │ 5 │ 1.03 │ 1.03 │ 2.40327e-09 │
│ 30 │ 5 │ 0.68 │ 0.68 │ 1.99959e-07 │
│ 62.5 │ 4 │ 0.5 │ 0.511111 │ -0.0714438 │
│ 125 │ 5 │ 0.52 │ 0.511111 │ 0.063901 │
╘════════╧═════╧═══════════════╧═════════════════════╧═══════════════════╛
╒════════╤═════╤═════════════╤═══════════════════╕
│ Dose │ N │ Sample SD │ Model Fitted SD │
╞════════╪═════╪═════════════╪═══════════════════╡
│ 0 │ 5 │ 0.25 │ 0.311053 │
│ 10 │ 5 │ 0.34 │ 0.311053 │
│ 30 │ 5 │ 0.29 │ 0.311053 │
│ 62.5 │ 4 │ 0.36 │ 0.311053 │
│ 125 │ 5 │ 0.47 │ 0.311053 │
╘════════╧═════╧═════════════╧═══════════════════╛

Likelihoods:
╒═════════╤══════════════════╤════════════╤═════════╕
│ Model │ Log-Likelihood │ # Params │ AIC │
╞═════════╪══════════════════╪════════════╪═════════╡
│ A1 │ -6.02289 │ 6 │ 24.0458 │
│ A2 │ -4.87112 │ 10 │ 29.7422 │
│ A3 │ -6.02289 │ 6 │ 24.0458 │
│ fitted │ -6.02749 │ 4 │ 20.055 │
│ reduced │ -11.4232 │ 2 │ 26.8463 │
╘═════════╧══════════════════╧════════════╧═════════╛

Tests of Mean and Variance Fits:
╒════════╤══════════════════════════════╤═════════════╤═══════════╕
│ Name │ -2 * Log(Likelihood Ratio) │ Test d.f. │ P-Value │
╞════════╪══════════════════════════════╪═════════════╪═══════════╡
│ Test 1 │ 13.1041 │ 8 │ 0.108318 │
│ Test 2 │ 2.30355 │ 4 │ 0.680123 │
│ Test 3 │ 2.30355 │ 4 │ 0.680123 │
│ Test 4 │ 0.00918931 │ 2 │ 0.995416 │
╘════════╧══════════════════════════════╧═════════════╧═══════════╛
Test 1: Test the null hypothesis that responses and variances don't differ among dose levels
(A2 vs R). If this test fails to reject the null hypothesis (p-value > 0.05), there may not be
a dose-response.

Test 2: Test the null hypothesis that variances are homogenous (A1 vs A2). If this test fails to
reject the null hypothesis (p-value > 0.05), the simpler constant variance model may be appropriate.

Test 3: Test the null hypothesis that the variances are adequately modeled (A3 vs A2). If this test
fails to reject the null hypothesis (p-value > 0.05), it may be inferred that the variances have
been modeled appropriately.

Test 4: Test the null hypothesis that the model for the mean fits the data (Fitted vs A3). If this
test fails to reject the null hypothesis (p-value > 0.1), the user has support for use of the
selected model.

# Liver wt, Rt, F, GD8-PND2, Conley et al. (2021)

## Dataset

**Name:** Liver wt, Rt, F, GD8-PND2, Conley et al. (2021)

| Dose | N | Mean | Std. Dev. |
| --- | --- | --- | --- |
| 0 | 5 | 11.9 | 1.34 |
| 10 | 5 | 13.4 | 1.12 |
| 30 | 5 | 15.6 | 1.57 |
| 62.5 | 4 | 16.2 | 1.8 |
| 125 | 5 | 16.9 | 2.01 |
| 250 | 5 | 18.1 | 1.79 |

Test 1 Dose Response: 0.0001

Test 2 Homogeneity of Variance: 0.8351

Test 3 Variance Model Selection: 0.8351

## Settings

| Setting | Value |
| --- | --- |
| BMR | 10% Relative Deviation |
| Distribution | Normal + Constant variance |
| Adverse Direction | Up (↑) |
| Maximum Polynomial Degree | 3 |
| Confidence Level (one sided) | 0.95 |

## Maximum Likelihood Approach

| Model | BMDL | BMD | BMDU | *P*-Value | AIC | Scaled Residual at Control | Scaled Residual near BMD | Recommendation and Notes |
| --- | --- | --- | --- | --- | --- | --- | --- | --- |
| Exponential 3 | 59.345 | 77.876 | 115.916 | 0.005 | 124.444 | -2.289 | 1.387 | **Questionable** Residual at control > 2.0 Goodness of fit p-value < 0.1 |
| Exponential 5 | 3.834 | 8.598 | 21.477 | 0.573 | 113.743 | -0.169 | 0.023 | **Viable** |
| Hill^ab^ | 2.637 | 6.419 | 19.233 | 0.818 | 112.678 | 0.036 | -0.242 | **Recommended - Lowest AIC** lowest dose/BMDL ratio > 3.0 |
| Polynomial 2 | 48.387 | 66.341 | 103.101 | 0.009 | 123.335 | -2.162 | 1.359 | **Questionable** Residual at control > 2.0 Goodness of fit p-value < 0.1 |
| Polynomial 3 | 103.594 | 105.763 | 107.929 | <0.001 | 129.844 | -2.321 | 1.268 | **Questionable** Residual at control > 2.0 Goodness of fit p-value < 0.1 Control stdev. fit > 1.5 |
| Power | 48.385 | 66.435 | 102.348 | 0.009 | 123.335 | -2.164 | 1.359 | **Questionable** Residual at control > 2.0 Goodness of fit p-value < 0.1 |
| Linear | 48.385 | 66.435 | 100.955 | 0.009 | 123.335 | -2.164 | 1.359 | **Questionable** Residual at control > 2.0 Goodness of fit p-value < 0.1 |

^a^ BMDS recommended best fitting model

^b^ User selected best fitting model


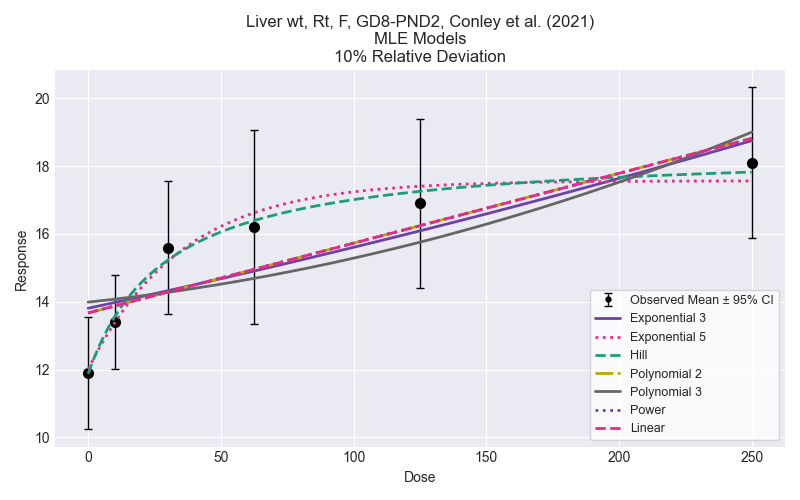


## Selected Model: Hill


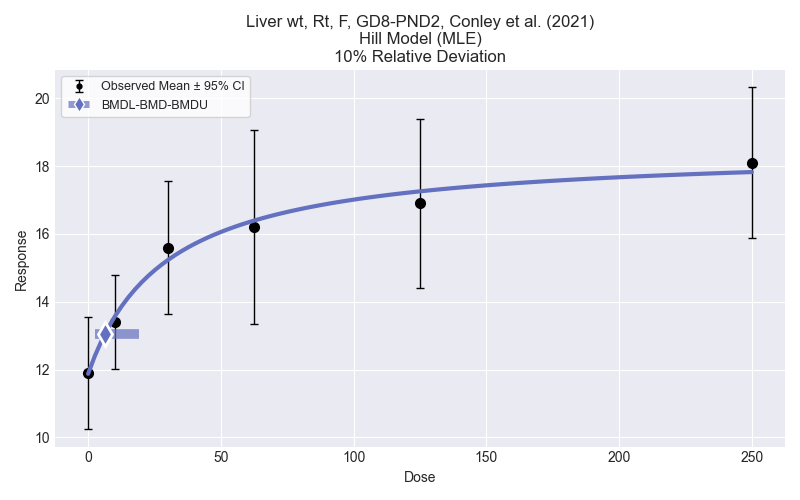


Hill Model
══════════════════════════════

Version: pybmds 25.1 (bmdscore 25.1)

Input Summary:
╒══════════════════════════════╤════════════════════════════╕
│ BMR │ 10% Relative Deviation │
│ Distribution │ Normal + Constant variance │
│ Modeling Direction │ Up (↑) │
│ Confidence Level (one sided) │ 0.95 │
│ Modeling Approach │ MLE │
╘══════════════════════════════╧════════════════════════════╛

Parameter Settings:
╒═════════════╤═══════════╤═══════╤═══════╕
│ Parameter │ Initial │ Min │ Max │
╞═════════════╪═══════════╪═══════╪═══════╡
│ g │ 0 │ -100 │ 100 │
│ v │ 0 │ -100 │ 100 │
│ k │ 0 │ 0 │ 5 │
│ n │ 1 │ 1 │ 18 │
│ alpha │ 0 │ -18 │ 18 │
╘═════════════╧═══════════╧═══════╧═══════╛

Modeling Summary:
╒════════════════╤════════════╕
│ BMD │ 6.41851 │
│ BMDL │ 2.63715 │
│ BMDU │ 19.233 │
│ AIC │ 112.678 │
│ Log-Likelihood │ -52.3389 │
│ P-Value │ 0.817762 │
│ Model d.f. │ 3 │
╘════════════════╧════════════╛

Model Parameters:
╒════════════╤════════════╤════════════╤══════════════╕
│ Variable │ Estimate │ On Bound │ Std Error │
╞════════════╪════════════╪════════════╪══════════════╡
│ g │ 11.8766 │ no │ 0.63192 │
│ v │ 6.65023 │ no │ 0.922443 │
│ k │ 29.5214 │ no │ 15.808 │
│ n │ 1 │ yes │ Not Reported │
│ alpha │ 2.16345 │ no │ 1.22907 │
╘════════════╧════════════╧════════════╧══════════════╛
Standard errors estimates are not generated for parameters estimated on corresponding bounds,
although sampling error is present for all parameters, as a rule. Standard error estimates may not
be reliable as a basis for confidence intervals or tests when one or more parameters are on bounds.


Goodness of Fit:
╒════════╤═════╤═══════════════╤═════════════════════╤═══════════════════╕
│ Dose │ N │ Sample Mean │ Model Fitted Mean │ Scaled Residual │
╞════════╪═════╪═══════════════╪═════════════════════╪═══════════════════╡
│ 0 │ 5 │ 11.9 │ 11.8766 │ 0.0355183 │
│ 10 │ 5 │ 13.4 │ 13.5593 │ -0.242211 │
│ 30 │ 5 │ 15.6 │ 15.2285 │ 0.564792 │
│ 62.5 │ 4 │ 16.2 │ 16.3934 │ -0.262974 │
│ 125 │ 5 │ 16.9 │ 17.2563 │ -0.541708 │
│ 250 │ 5 │ 18.1 │ 17.8245 │ 0.41882 │
╘════════╧═════╧═══════════════╧═════════════════════╧═══════════════════╛
╒════════╤═════╤═════════════╤═══════════════════╕
│ Dose │ N │ Sample SD │ Model Fitted SD │
╞════════╪═════╪═════════════╪═══════════════════╡
│ 0 │ 5 │ 1.34 │ 1.47087 │
│ 10 │ 5 │ 1.12 │ 1.47087 │
│ 30 │ 5 │ 1.57 │ 1.47087 │
│ 62.5 │ 4 │ 1.8 │ 1.47087 │
│ 125 │ 5 │ 2.01 │ 1.47087 │
│ 250 │ 5 │ 1.79 │ 1.47087 │
╘════════╧═════╧═════════════╧═══════════════════╛

Likelihoods:
╒═════════╤══════════════════╤════════════╤═════════╕
│ Model │ Log-Likelihood │ # Params │ AIC │
╞═════════╪══════════════════╪════════════╪═════════╡
│ A1 │ -51.8731 │ 7 │ 117.746 │
│ A2 │ -50.8228 │ 12 │ 125.646 │
│ A3 │ -51.8731 │ 7 │ 117.746 │
│ fitted │ -52.3389 │ 4 │ 112.678 │
│ reduced │ -68.5785 │ 2 │ 141.157 │
╘═════════╧══════════════════╧════════════╧═════════╛

Tests of Mean and Variance Fits:
╒════════╤══════════════════════════════╤═════════════╤═════════════╕
│ Name │ -2 * Log(Likelihood Ratio) │ Test d.f. │ P-Value │
╞════════╪══════════════════════════════╪═════════════╪═════════════╡
│ Test 1 │ 35.5114 │ 10 │ 0.000102096 │
│ Test 2 │ 2.1005 │ 5 │ 0.835071 │
│ Test 3 │ 2.1005 │ 5 │ 0.835071 │
│ Test 4 │ 0.931741 │ 3 │ 0.817762 │
╘════════╧══════════════════════════════╧═════════════╧═════════════╛
Test 1: Test the null hypothesis that responses and variances don't differ among dose levels
(A2 vs R). If this test fails to reject the null hypothesis (p-value > 0.05), there may not be
a dose-response.

Test 2: Test the null hypothesis that variances are homogenous (A1 vs A2). If this test fails to
reject the null hypothesis (p-value > 0.05), the simpler constant variance model may be appropriate.

Test 3: Test the null hypothesis that the variances are adequately modeled (A3 vs A2). If this test
fails to reject the null hypothesis (p-value > 0.05), it may be inferred that the variances have
been modeled appropriately.

Test 4: Test the null hypothesis that the model for the mean fits the data (Fitted vs A3). If this
test fails to reject the null hypothesis (p-value > 0.1), the user has support for use of the
selected model.

# Gestational BW gain, Rt, F, GD8-PND2, Conley et al. (2021)

## Dataset

**Name:** Gestational BW gain, Rt, F, GD8-PND2, Conley et al. (2021)

| Dose | N | Mean | Std. Dev. |
| --- | --- | --- | --- |
| 0 | 5 | 150 | 20 |
| 10 | 5 | 156 | 16 |
| 30 | 5 | 150 | 16 |
| 62.5 | 4 | 125 | 8 |
| 125 | 5 | 103 | 16 |
| 250 | 4 | 96 | 26 |

Test 1 Dose Response: <0.0001

Test 2 Homogeneity of Variance: 0.4044

Test 3 Variance Model Selection: 0.4044

## Settings

| Setting | Value |
| --- | --- |
| BMR | 10% Relative Deviation |
| Distribution | Normal + Constant variance |
| Adverse Direction | Down (↓) |
| Maximum Polynomial Degree | 3 |
| Confidence Level (one sided) | 0.95 |

## Maximum Likelihood Approach

| Model | BMDL | BMD | BMDU | *P*-Value | AIC | Scaled Residual at Control | Scaled Residual near BMD | Recommendation and Notes |
| --- | --- | --- | --- | --- | --- | --- | --- | --- |
| Exponential 3 | 34.764 | 45.255 | 71.251 | 0.16 | 246.199 | -0.421 | 0.89 | **Viable** |
| Exponential 5 | 25.681 | 49.963 | 69.353 | 0.669 | 244.429 | -0.42 | -0.002 | **Viable** |
| Hill^ab^ | 30.583 | 47.227 | 65.858 | 0.798 | 244.077 | -0.476 | -0.101 | **Recommended - Lowest AIC** |
| Polynomial 2 | 47.021 | 57.962 | 80.318 | 0.07 | 248.302 | -0.08 | -1.027 | **Questionable** Goodness of fit p-value < 0.1 |
| Polynomial 3 | 46.787 | 59.787 | 81.379 | 0.032 | 250.434 | -0.013 | -1.015 | **Questionable** Goodness of fit p-value < 0.1 |
| Power | 47.023 | 57.926 | 83.861 | 0.07 | 248.302 | -0.082 | -1.028 | **Questionable** Goodness of fit p-value < 0.1 |
| Linear | 47.023 | 57.926 | 76.709 | 0.07 | 248.302 | -0.082 | -1.028 | **Questionable** Goodness of fit p-value < 0.1 |

^a^ BMDS recommended best fitting model

^b^ User selected best fitting model


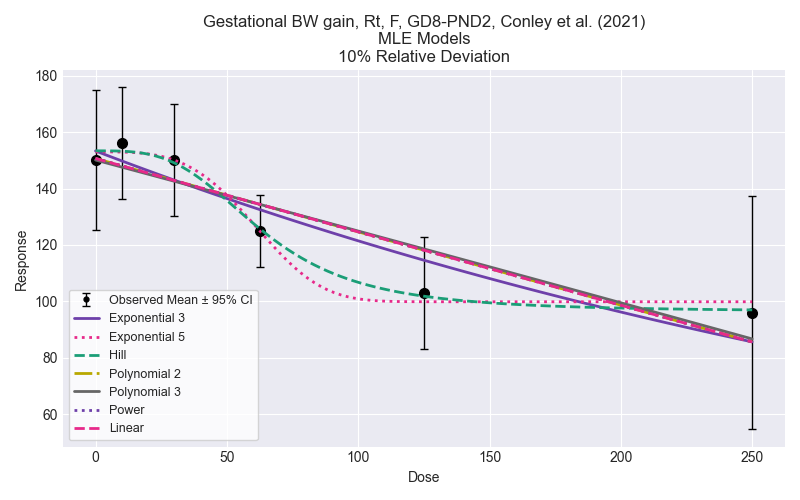


## Selected Model: Hill


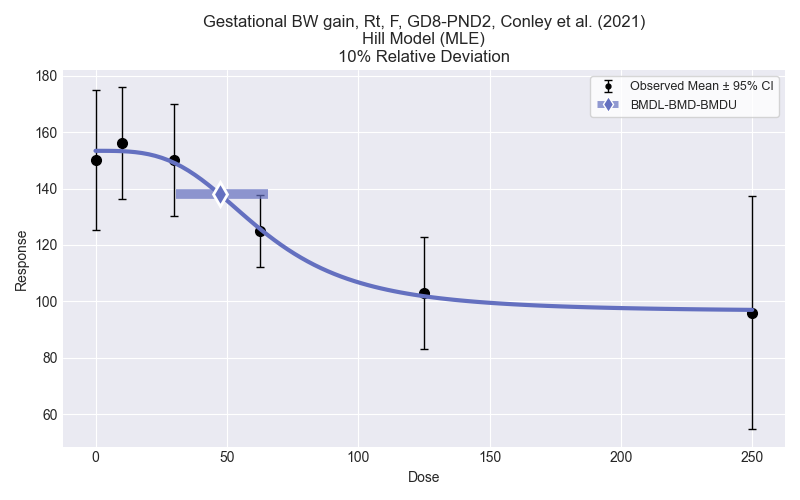


Hill Model
══════════════════════════════

Version: pybmds 25.1 (bmdscore 25.1)

Input Summary:
╒══════════════════════════════╤════════════════════════════╕
│ BMR │ 10% Relative Deviation │
│ Distribution │ Normal + Constant variance │
│ Modeling Direction │ Down (↓) │
│ Confidence Level (one sided) │ 0.95 │
│ Modeling Approach │ MLE │
╘══════════════════════════════╧════════════════════════════╛

Parameter Settings:
╒═════════════╤═══════════╤═══════╤═══════╕
│ Parameter │ Initial │ Min │ Max │
╞═════════════╪═══════════╪═══════╪═══════╡
│ g │ 0 │ -100 │ 100 │
│ v │ 0 │ -100 │ 100 │
│ k │ 0 │ 0 │ 5 │
│ n │ 1 │ 1 │ 18 │
│ alpha │ 0 │ -18 │ 18 │
╘═════════════╧═══════════╧═══════╧═══════╛

Modeling Summary:
╒════════════════╤═════════════╕
│ BMD │ 47.2272 │
│ BMDL │ 30.583 │
│ BMDU │ 65.8579 │
│ AIC │ 244.077 │
│ Log-Likelihood │ -117.038 │
│ P-Value │ 0.798319 │
│ Model d.f. │ 2 │
╘════════════════╧═════════════╛

Model Parameters:
╒════════════╤════════════╤════════════╤═════════════╕
│ Variable │ Estimate │ On Bound │ Std Error │
╞════════════╪════════════╪════════════╪═════════════╡
│ g │ 153.364 │ no │ 4.7906 │
│ v │ -56.9463 │ no │ 10.7854 │
│ k │ 63.7063 │ no │ 13.5512 │
│ n │ 3.33465 │ no │ 2.19273 │
│ alpha │ 250.135 │ no │ 16721.8 │
╘════════════╧════════════╧════════════╧═════════════╛

Goodness of Fit:
╒════════╤═════╤═══════════════╤═════════════════════╤═══════════════════╕
│ Dose │ N │ Sample Mean │ Model Fitted Mean │ Scaled Residual │
╞════════╪═════╪═══════════════╪═════════════════════╪═══════════════════╡
│ 0 │ 5 │ 150 │ 153.364 │ -0.475547 │
│ 10 │ 5 │ 156 │ 153.245 │ 0.389474 │
│ 30 │ 5 │ 150 │ 149.089 │ 0.128867 │
│ 62.5 │ 4 │ 125 │ 125.798 │ -0.100873 │
│ 125 │ 5 │ 103 │ 101.859 │ 0.161376 │
│ 250 │ 4 │ 96 │ 97.0074 │ -0.127396 │
╘════════╧═════╧═══════════════╧═════════════════════╧═══════════════════╛
╒════════╤═════╤═════════════╤═══════════════════╕
│ Dose │ N │ Sample SD │ Model Fitted SD │
╞════════╪═════╪═════════════╪═══════════════════╡
│ 0 │ 5 │ 20 │ 15.8157 │
│ 10 │ 5 │ 16 │ 15.8157 │
│ 30 │ 5 │ 16 │ 15.8157 │
│ 62.5 │ 4 │ 8 │ 15.8157 │
│ 125 │ 5 │ 16 │ 15.8157 │
│ 250 │ 4 │ 26 │ 15.8157 │
╘════════╧═════╧═════════════╧═══════════════════╛

Likelihoods:
╒═════════╤══════════════════╤════════════╤═════════╕
│ Model │ Log-Likelihood │ # Params │ AIC │
╞═════════╪══════════════════╪════════════╪═════════╡
│ A1 │ -116.813 │ 7 │ 247.626 │
│ A2 │ -114.266 │ 12 │ 252.532 │
│ A3 │ -116.813 │ 7 │ 247.626 │
│ fitted │ -117.038 │ 5 │ 244.077 │
│ reduced │ -133.431 │ 2 │ 270.862 │
╘═════════╧══════════════════╧════════════╧═════════╛

Tests of Mean and Variance Fits:
╒════════╤══════════════════════════════╤═════════════╤════════════╕
│ Name │ -2 * Log(Likelihood Ratio) │ Test d.f. │ P-Value │
╞════════╪══════════════════════════════╪═════════════╪════════════╡
│ Test 1 │ 38.3305 │ 10 │ 3.3238e-05 │
│ Test 2 │ 5.09458 │ 5 │ 0.404447 │
│ Test 3 │ 5.09458 │ 5 │ 0.404447 │
│ Test 4 │ 0.450493 │ 2 │ 0.798319 │
╘════════╧══════════════════════════════╧═════════════╧════════════╛
Test 1: Test the null hypothesis that responses and variances don't differ among dose levels
(A2 vs R). If this test fails to reject the null hypothesis (p-value > 0.05), there may not be
a dose-response.

Test 2: Test the null hypothesis that variances are homogenous (A1 vs A2). If this test fails to
reject the null hypothesis (p-value > 0.05), the simpler constant variance model may be appropriate.

Test 3: Test the null hypothesis that the variances are adequately modeled (A3 vs A2). If this test
fails to reject the null hypothesis (p-value > 0.05), it may be inferred that the variances have
been modeled appropriately.

Test 4: Test the null hypothesis that the model for the mean fits the data (Fitted vs A3). If this
test fails to reject the null hypothesis (p-value > 0.1), the user has support for use of the
selected model.

# BW PND2, Rt, F, GD8-PND2, Conley et al. (2021)

## Dataset

**Name:** BW PND2, Rt, F, GD8-PND2, Conley et al. (2021)

| Dose | N | Mean | Std. Dev. |
| --- | --- | --- | --- |
| 0 | 5 | 298 | 22 |
| 10 | 5 | 292 | 25 |
| 30 | 5 | 292 | 22 |
| 62.5 | 4 | 275 | 14 |
| 125 | 5 | 256 | 18 |
| 250 | 5 | 240 | 18 |

Test 1 Dose Response: 0.0022

Test 2 Homogeneity of Variance: 0.8602

Test 3 Variance Model Selection: 0.8602

## Settings

| Setting | Value |
| --- | --- |
| BMR | 10% Relative Deviation |
| Distribution | Normal + Constant variance |
| Adverse Direction | Down (↓) |
| Maximum Polynomial Degree | 3 |
| Confidence Level (one sided) | 0.95 |

## Maximum Likelihood Approach

| Model | BMDL | BMD | BMDU | *P*-Value | AIC | Scaled Residual at Control | Scaled Residual near BMD | Recommendation and Notes |
| --- | --- | --- | --- | --- | --- | --- | --- | --- |
| Exponential 3^ab^ | 89.443 | 115.876 | 166.248 | 0.774 | 258.21 | 0.308 | -0.918 | **Recommended - Lowest AIC** |
| Exponential 5 | 46.403 | 88.583 | 145.445 | 0.847 | 260.75 | 0.183 | -0.194 | **Viable** |
| Hill | 49.388 | 87.737 | 141.965 | 0.856 | 260.728 | 0.242 | -0.154 | **Viable** |
| Polynomial 2 | 98.365 | 123.929 | 172.224 | 0.694 | 258.643 | 0.406 | -1.051 | **Viable** |
| Polynomial 3 | 180.277 | 184.051 | 187.819 | 0.03 | 267.435 | 1.005 | -1.935 | **Questionable** Goodness of fit p-value < 0.1 |
| Power | 98.363 | 123.968 | 172.104 | 0.694 | 258.643 | 0.407 | -1.051 | **Viable** |
| Linear | 98.363 | 123.968 | 169.651 | 0.694 | 258.643 | 0.407 | -1.051 | **Viable** |

^a^ BMDS recommended best fitting model

^b^ User selected best fitting model


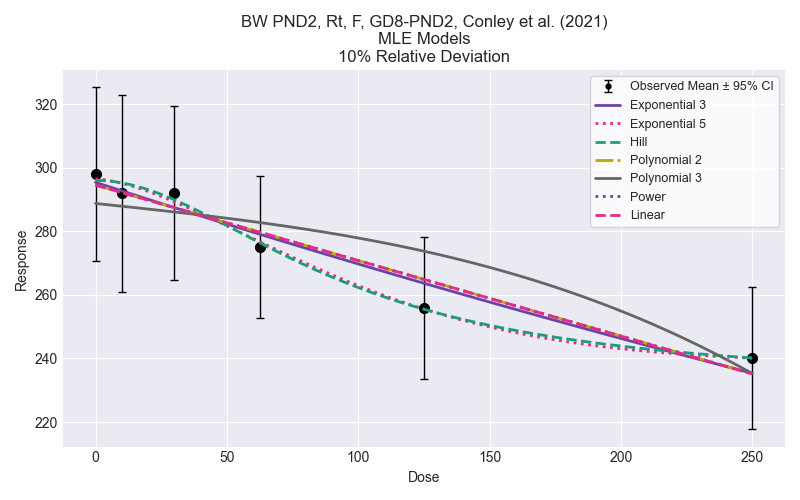


## Selected Model: Exponential 3


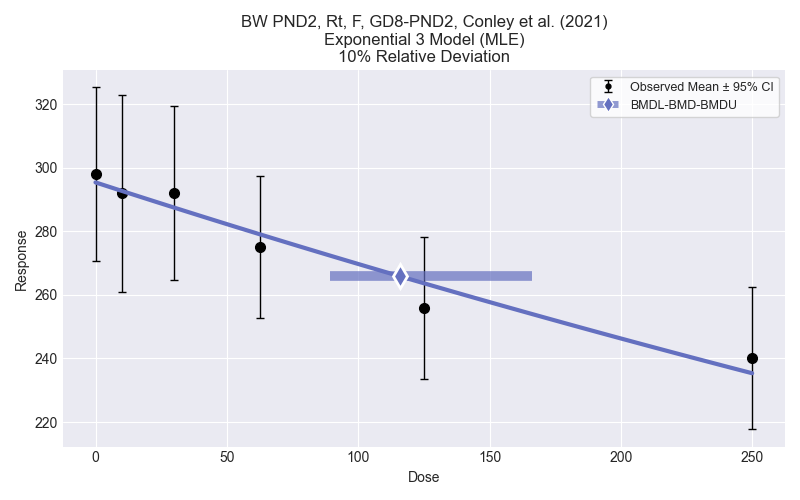


Exponential 3 Model
══════════════════════════════

Version: pybmds 25.1 (bmdscore 25.1)

Input Summary:
╒══════════════════════════════╤════════════════════════════╕
│ BMR │ 10% Relative Deviation │
│ Distribution │ Normal + Constant variance │
│ Modeling Direction │ Down (↓) │
│ Confidence Level (one sided) │ 0.95 │
│ Modeling Approach │ MLE │
╘══════════════════════════════╧════════════════════════════╛

Parameter Settings:
╒═════════════╤═══════════╤═══════╤═══════╕
│ Parameter │ Initial │ Min │ Max │
╞═════════════╪═══════════╪═══════╪═══════╡
│ a │ 0 │ 0 │ 100 │
│ b │ 0 │ 0 │ 100 │
│ c │ 0 │ -20 │ 0 │
│ d │ 1 │ 1 │ 18 │
│ log-alpha │ 0 │ -18 │ 18 │
╘═════════════╧═══════════╧═══════╧═══════╛

Modeling Summary:
╒════════════════╤═════════════╕
│ BMD │ 115.876 │
│ BMDL │ 89.4428 │
│ BMDU │ 166.248 │
│ AIC │ 258.21 │
│ Log-Likelihood │ -126.105 │
│ P-Value │ 0.773933 │
│ Model d.f. │ 4 │
╘════════════════╧═════════════╛

Model Parameters:
╒════════════╤═══════════════╤════════════╤══════════════╕
│ Variable │ Estimate │ On Bound │ Std Error │
╞════════════╪═══════════════╪════════════╪══════════════╡
│ a │ 295.419 │ no │ 4.85216 │
│ b │ 0.000909249 │ no │ 0.000157101 │
│ d │ 1 │ yes │ Not Reported │
│ log-alpha │ 5.85901 │ no │ 0.262613 │
╘════════════╧═══════════════╧════════════╧══════════════╛
Standard errors estimates are not generated for parameters estimated on corresponding bounds,
although sampling error is present for all parameters, as a rule. Standard error estimates may not
be reliable as a basis for confidence intervals or tests when one or more parameters are on bounds.


Goodness of Fit:
╒════════╤═════╤═══════════════╤═════════════════════╤═══════════════════╕
│ Dose │ N │ Sample Mean │ Model Fitted Mean │ Scaled Residual │
╞════════╪═════╪═══════════════╪═════════════════════╪═══════════════════╡
│ 0 │ 5 │ 298 │ 295.419 │ 0.308311 │
│ 10 │ 5 │ 292 │ 292.745 │ -0.0890184 │
│ 30 │ 5 │ 292 │ 287.47 │ 0.541181 │
│ 62.5 │ 4 │ 275 │ 279.099 │ -0.437979 │
│ 125 │ 5 │ 256 │ 263.681 │ -0.917528 │
│ 250 │ 5 │ 240 │ 235.352 │ 0.555229 │
╘════════╧═════╧═══════════════╧═════════════════════╧═══════════════════╛
╒════════╤═════╤═════════════╤═══════════════════╕
│ Dose │ N │ Sample SD │ Model Fitted SD │
╞════════╪═════╪═════════════╪═══════════════════╡
│ 0 │ 5 │ 22 │ 18.7183 │
│ 10 │ 5 │ 25 │ 18.7183 │
│ 30 │ 5 │ 22 │ 18.7183 │
│ 62.5 │ 4 │ 14 │ 18.7183 │
│ 125 │ 5 │ 18 │ 18.7183 │
│ 250 │ 5 │ 18 │ 18.7183 │
╘════════╧═════╧═════════════╧═══════════════════╛

Likelihoods:
╒═════════╤══════════════════╤════════════╤═════════╕
│ Model │ Log-Likelihood │ # Params │ AIC │
╞═════════╪══════════════════╪════════════╪═════════╡
│ A1 │ -125.209 │ 7 │ 264.418 │
│ A2 │ -124.249 │ 12 │ 272.499 │
│ A3 │ -125.209 │ 7 │ 264.418 │
│ fitted │ -126.105 │ 3 │ 258.21 │
│ reduced │ -137.956 │ 2 │ 279.913 │
╘═════════╧══════════════════╧════════════╧═════════╛

Tests of Mean and Variance Fits:
╒════════╤══════════════════════════════╤═════════════╤════════════╕
│ Name │ -2 * Log(Likelihood Ratio) │ Test d.f. │ P-Value │
╞════════╪══════════════════════════════╪═════════════╪════════════╡
│ Test 1 │ 27.4141 │ 10 │ 0.00223881 │
│ Test 2 │ 1.91891 │ 5 │ 0.860248 │
│ Test 3 │ 1.91891 │ 5 │ 0.860248 │
│ Test 4 │ 1.79207 │ 4 │ 0.773933 │
╘════════╧══════════════════════════════╧═════════════╧════════════╛
Test 1: Test the null hypothesis that responses and variances don't differ among dose levels
(A2 vs R). If this test fails to reject the null hypothesis (p-value > 0.05), there may not be
a dose-response.

Test 2: Test the null hypothesis that variances are homogenous (A1 vs A2). If this test fails to
reject the null hypothesis (p-value > 0.05), the simpler constant variance model may be appropriate.

Test 3: Test the null hypothesis that the variances are adequately modeled (A3 vs A2). If this test
fails to reject the null hypothesis (p-value > 0.05), it may be inferred that the variances have
been modeled appropriately.

Test 4: Test the null hypothesis that the model for the mean fits the data (Fitted vs A3). If this
test fails to reject the null hypothesis (p-value > 0.1), the user has support for use of the
selected model.
